# Genome-wide expression profiling in leaves and roots of date palm (*Phoenix dactylifera* L.) exposed to salinity

**DOI:** 10.1186/s12864-017-3633-6

**Published:** 2017-03-22

**Authors:** Mahmoud W. Yaish, Himanshu V. Patankar, Dekoum V. M. Assaha, Yun Zheng, Rashid Al-Yahyai, Ramanjulu Sunkar

**Affiliations:** 1 Department of Biology, College of Science, Sultan Qaboos University, Muscat, Oman; 2 Faculty of Life Science and Technology, Kunming University of Science and Technology, Kunming, Yunnan 650500 China; 3 Department of Crop Science, College of Agriculture and Marine Sciences, Sultan Qaboos University, Muscat, Oman; 4 Department of Biochemistry and Molecular Biology, Oklahoma State University, Stillwater, OK 74078 USA

**Keywords:** date palm, differentially expressed genes, DEGs, leaves, RNA-seq, roots, salinity

## Abstract

**Background:**

Date palm, as one of the most important fruit crops in North African and West Asian countries including Oman, is facing serious growth problems due to salinity, arising from persistent use of saline water for irrigation. Although date palm is a relatively salt-tolerant plant species, its adaptive mechanisms to salt stress are largely unknown.

**Results:**

In order to get an insight into molecular mechanisms of salt tolerance, RNA was profiled in leaves and roots of date palm seedlings subjected to NaCl for 10 days. Under salt stress, photosynthetic parameters were differentially affected; all gas exchange parameters were decreased but the quantum yield of PSII was unaffected while non-photochemical quenching was increased. Analyses of gene expression profiles revealed 2630 and 4687 genes were differentially expressed in leaves and roots, respectively, under salt stress. Of these, 194 genes were identified as commonly responding in both the tissue sources. Gene ontology (GO) analysis in leaves revealed enrichment of transcripts involved in metabolic pathways including photosynthesis, sucrose and starch metabolism, and oxidative phosphorylation, while in roots genes involved in membrane transport, phenylpropanoid biosynthesis, purine, thiamine, and tryptophan metabolism, and casparian strip development were enriched. Differentially expressed genes (DEGs) common to both tissues included the auxin responsive gene, GH3, a putative potassium transporter 8 and vacuolar membrane proton pump.

**Conclusions:**

Leaf and root tissues respond differentially to salinity stress and this study has revealed genes and pathways that are associated with responses to elevated NaCl levels and thus may play important roles in salt tolerance providing a foundation for functional characterization of salt stress-responsive genes in the date palm.

**Electronic supplementary material:**

The online version of this article (doi:10.1186/s12864-017-3633-6) contains supplementary material, which is available to authorized users.

## Background

Soil salinity is a global problem, which severely affects the agricultural production and the total organic stock on our planet [1, 2]. This problem becomes greater in regions with dry climates, sporadic rainfall and high temperatures, leading to high evapotranspiration rates [3]. All of these agriculturally devastating factors are common in some arid and semi-arid regions of the Mideast and North Africa, where date palm (*Phoenix dactylifera* L.) is considered as a major fruit crop. For example, in Oman 70% of the total agricultural land area has been affected by salinity [4–6] due to high evaporation rates from the soil surface and the shortage of precipitation and fresh water. This situation has led to the farmers using saline groundwater, resulting from seawater intrusion, for irrigation, which ultimately leads to accumulation of salts in the soil, consequently leading to the salinization of vast agricultural regions.

Saline environments can severely affect plants primarily by exerting osmotic and ionic effects [7, 8]. Osmotic effects lead to cellular dehydration and reduced accessibility of soil water by the root system, whereas toxic ionic effect results from accumulation of Na^+^ and/or Cl^-^ ions in the cell. High levels of Na^+^ concentration can interfere with K^+^ and Ca^2+^ flux across the guard cell membranes, thereby interrupting their turgidity and the regular gas exchange across the stoma, while high Cl^–^ ions can decrease photosynthesis through the degradation of chlorophyll [9]. Additionally, the accumulation of Na^+^ and Cl^-^ ions in the cytosol causes cellular toxicity through the production of reactive oxygen species (ROS) [10] which in turn causes protein and lipid degradation and also intercellular destructions through autophagy [11]. However, salt-tolerant plants have evolved several mechanisms to cope with the osmotic and ion toxicity effects that include extrusion of the salts and compartmentation of Na^+^ ions in the vacuole [12, 13] and the production of osmolytes such as carnitine, fructans, glutamate, glycine-betaine, inorganic ions like K^+^, mannitol, oligosaccharides, proline, polyols, sorbitol, sucrose and trehalose [14] as well as enhanced enzymatic (SOD, catalase, peroxidases) and non-enzymatic (glutathione, ascorbic acid, anthocyanins, etc) antioxidants [6, 7].

Salinity induces changes in gene expression at the global scale. For example, active salt-adapted cells are able to activate or repress a set of genes coding for a family of ion transporter proteins including the Na^+^/H^+^ antiporters located within the plasma and tonoplast membranes [15–17]. Although these proteins may be involved in the salinity adaptive mechanisms of some known plant species, the mechanisms of a large number of plant species remains unknown [18].

Although some date palm cultivars can sustain growth even up to 12.8 dS.m^-1^ [19], some others can only tolerate moderate salinity range (between 4 and 10 dS.m^−1^) [20, 21]. Despite salt tolerance levels among cultivars being highly varied, date palm is generally considered as a relatively salt tolerant species [19–22], but the molecular basis of the salt tolerance is poorly understood in this plant species [23]. The date palm cultivar, *Khalas,* is one of the most important date palm cultivars in the Middle East owing to its large, and energy rich fruits, qualities that are being exploited in date palm breeding programs in the region [24, 25]. It is relatively tolerant to different abiotic stress including salinity [26, 27]. The only transcriptomic analysis in response to salt stress in date palm in general was recently conducted [28]. In that study seedlings of date palm were shocked after removal from the soil with 150 mM NaCl for 2 or 4 h and the differentially expressed genes (DEGs) were analyzed in the root. This duration of treatment elicits mostly responses to the osmotic effects of salt stress, leaving out the salt-specific DEGs that show up after several days or weeks of treatment [29–31]. Secondly, the transcriptomic data for the leaves in the study were not evaluated. Thus transcriptomic studies to reveal salt-specific changes in gene expression, required for adaption to salinity in date palm is still required. Therefore, global differential gene expression analyses in salt-treated and untreated date palm will lay the foundation for understanding salinity response and salt tolerance mechanisms in this plant species, and has the potential to reveal novel gene(s) or pathway(s) responsible for salt-adaptation phenotype.

In the present study, four mRNA libraries prepared from leaves and roots of NaCl-treated and untreated seedlings of *Khalas* were sequenced, *de novo* assembled and analyzed. Sequence analyses revealed differential expression of a large number of genes in date palm seedlings exposed to soil salinity. Based on sequence similarities, genes related to different known salt response and pathways are strongly represented within the DEGs.

## Results

### Effect of salinity on growth and photosynthetic gas exchange

**Fig. 1:**
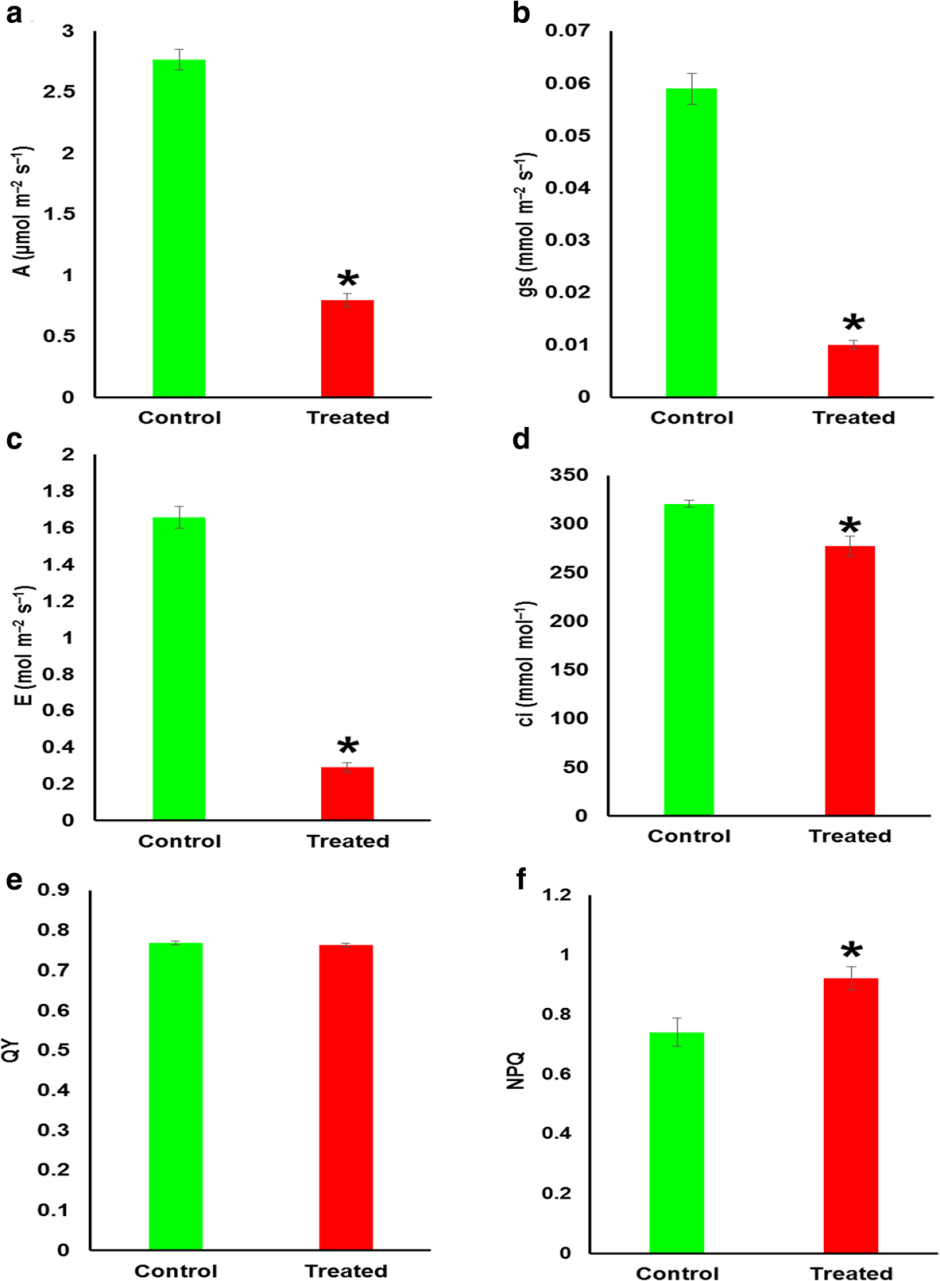
Changes in gas exchange parameters and chlorophyll fluorescence of plants subjected to salt stress and control conditions for 9 days. **a** Photosynthetic rate (A), **b**) Stomatal conductance (gs), **c**) Transpiration rate (E), **d**) Internal CO_2_ concentration (ci), **e**) Quantum Yield (QY), and **f**) Non-Photochemical Quenching (NPQ). The values are means (*n* = 9) and error bars represent SE (*p* < 0.05), significant differences are marked by asterisk

Eight-week-old date palm seedlings were irrigated with 300 mM NaCl solution every two days for 10 days. At the end of the treatment, the average EC of the treated and control soil was 18.2 (±0.5 S.D.) and 0.9 (±0.2 S.D.) dS.m^-1^, respectively. Morphologically, salt-treated seedlings were not different from untreated controls, which could be due to short duration of the NaCl stress. However, salinity significantly (*p* < 0.05) reduced the photosynthetic rate (A) in date palm (Fig. 1). The average net photosynthetic rate decreased by 71% in salt-stressed plants (Fig. 1a), while stomatal conductance (gs) and transpiration rate (E) decreased by 80% and 82%, respectively (Fig. 1b and c). Also, the internal carbon dioxide concentration (ci) was significantly (*p* < 0.05) lowered by ~9% in the treated plants (Fig. 1d). However, salinity did not alter the quantum yield of PSII efficiency (QY) (Fig. 1e), but significantly raised the non-photochemical quenching (NPQ) (Fig. 1f).

### RNA Sequencing and assembly

Plants have acquired several mechanisms to adapt to salinity conditions. While roots are directly exposed to the saline environments within the soil, leaves may also directly or indirectly be affected by this environment, thus it is important to examine the tissue-specific responses to salt stress. To understand the molecular basis of salt tolerance/adaptation in date palm, RNA-seq libraries were generated from leaves and roots of NaCl-treated and control seedlings were sequenced and assembled. A total of 43,243 raw nucleotide reads accounting for a total length of 31,717,837 bp of contigs, with a length range from 201 to 12,301 bp obtained from the leaf libraries, including 89,382,824 and 143,071,624 contigs, were sequenced from the control and NaCl-treated leaves, respectively. On the other hand, a total of 85,526 raw nucleotide reads accounting for 67,808,169 bp total length of contigs, with a length ranged from 201 to 7671 bp obtained from the root libraries including 114,677,846 and 95,910,068 contigs were sequenced from the control and NaCl-treated roots, respectively (Table 1).

**Table 1:** Transcriptomic sequences obtained from the analysis of total RNA isolated from NaCl treated and untreated leaf and root tissues

Sample	Number of reads	Total (bp)	Total Length of contigs	Total number of contigs	Maximum length (bp)	Minimum length (bp)	Differentially expressed (≥2 fold change, *p-*value, FDR < 0.05)	
Control-leaf	89,382,824	9,027,665,224	31,717,837	43,243	12,301	201	Up-regulated	1270
Treated-leaf	143,071,624	14,450,234,024					Down-regulated	1360
Control-root	114,677,846	11,582,462,446	67,808,169	85,526	7671	201	Up-regulated	2297
Treated-root	95,910,068	9,686,916,868					Down-regulated	2387
Total	443,042,362	44,747,278,562	99,526,006	128,769				7314

### Sequence analysis and functional annotation of the transcripts sequenced from the leaf and root tissues

To obtain a general impression of the identity of the total mRNA sequenced in this study, transcripts from leaves and roots were searched against the public databases and classified based on their predicted biological functions. Deduced amino acid translation of the coding regions of the sequenced transcripts from leaves yielded a total of 31,823 protein sequences. Interestingly, 11,420 of the sequences (26%) were not similar to proteins available in the databases. The functional annotation revealed that each of the biological processes and molecular function categories occupied 27% of the total sequence, while only 20% of the sequences were involved in cellular component category (Additional file 1: Figure S1). Functional annotation analysis showed that these proteins were mainly involved in the biological processes such as response to stress, transport and signal transduction (Additional file 2: Figure S2), whereas other proteins were mainly found in specific cellular components, such as the plasma membrane, chloroplast and nucleus. Classification of these proteins, based on the molecular function, showed that they were mainly involved in binding, transportation and hydrolysis processes. In roots, a total of 50,990 transcripts were assigned to protein coding sequences (Additional file 1: Figure S1B). Gene ontology (GO) and functional annotation revealed that the majority of the proteins were involved in cellular, metabolic and response to stimulus processes; in chloroplast, nucleus and plasma membrane cellular components; and molecularly involved in the binding, hydrolysis and transport functions (Additional file 3: Figure S3).

### DEGs in leaves

**Fig. 2:**
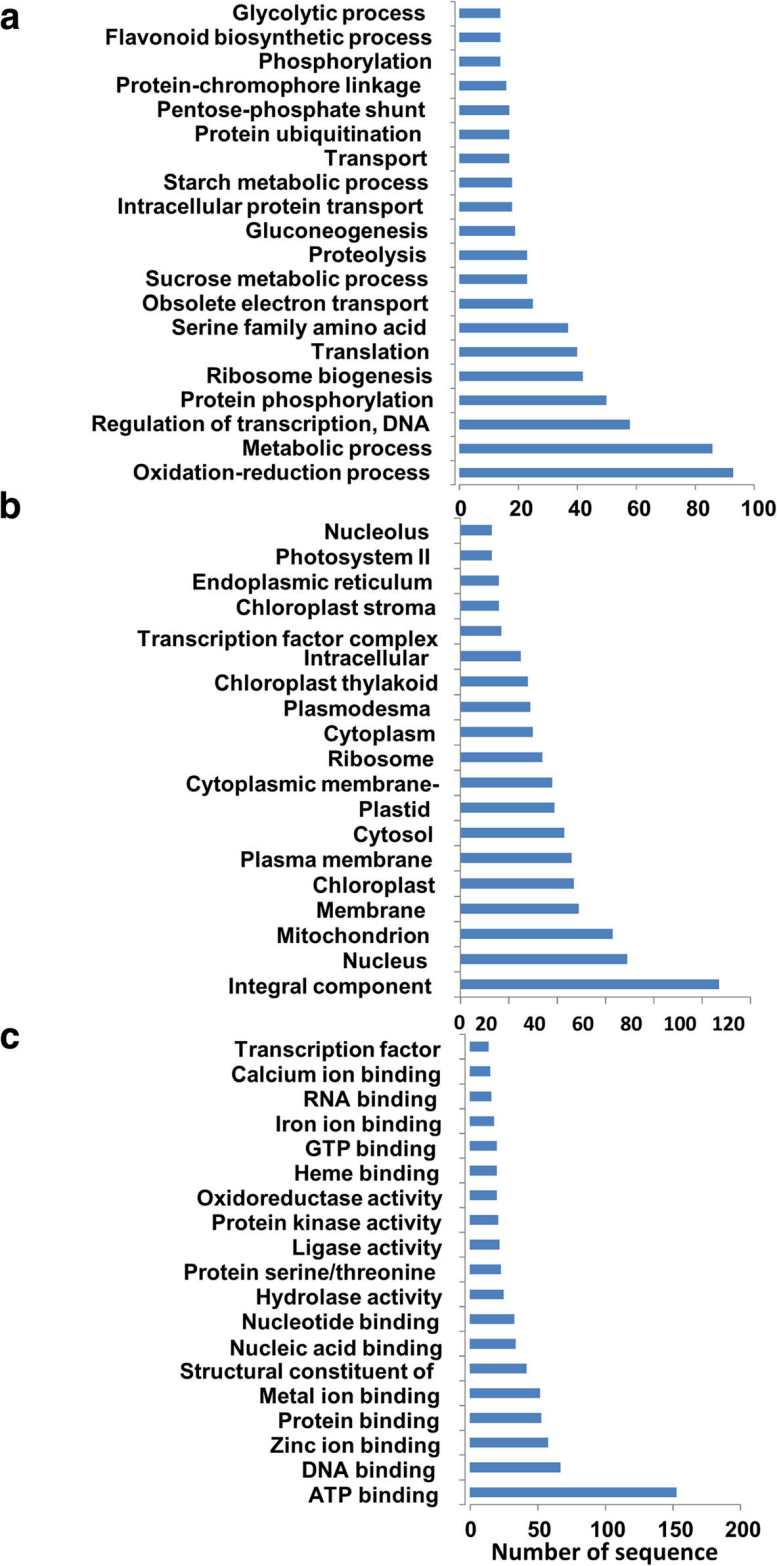
The Distribution of GO categories between the major three GO domains under salinity stress condition in date palm leaves. The genes were classified based on the biological processes (**a**), cellular components (**b**), and molecular functions (**c**). The bar represents the number of sequences in different GO categories

**Fig. 3:**
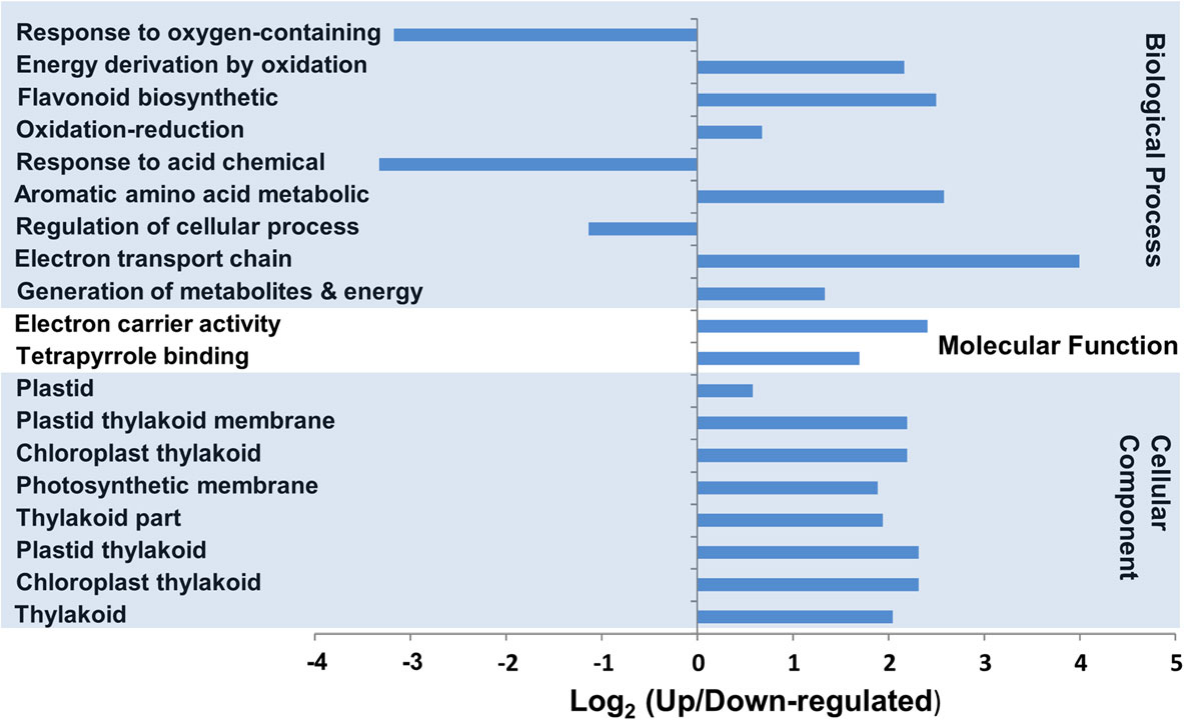
The distribution of DEGs among the GO categories in leaf tissues obtained using the Fisher’s exact test. The bar represents the log_2_ transformed ratio between category portions in the differentially expressed gene sets. The ratio greater than zero indicates that there were more genes up regulated due to salinity stress in the particular category. Significantly (*p* ≤ 0.001) enrich categories are shown

In order to determine the potential function of differentially expressed transcripts, a biological function was assigned to them and tested for their annotation enrichments based on the variation in gene expression levels between the control and the NaCl-treated plants. Differential gene expression analysis in leaves revealed a total of 2630 transcripts whose expression levels were significantly (*p*, *FDR* < 0.05) altered when the plants were exposed to salinity (Additional file 4: Table S1). These included 1360 upregulated and 1270 downregulated transcripts with an expression value ≥ 2 fold (*p*, *FDR* < 0.05) (Table 1). Functional annotation revealed that salinity affected the expression levels of genes involved in the oxidation-reduction, regulation of transcription and proteolysis processes (Fig. 2a); the integral component of the plasma membranes and the organellar membranes (Fig. 2b); and the binding to ATP, DNA, minerals and ions (Fig. 2c). The Fisher’s exact test was employed to determine the significant (*p* < 0.01) GO term enrichments among the DEGs using the up-regulated transcripts as a test group against the downregulated genes which were used as a reference group. The enrichment analysis of the GO terms of these transcripts showed that 19 were significantly enriched GOs (*p* < 0.01), including three GOs in the leaves grown under normal conditions, and 16 GOs in the leaves treated with NaCl (Fig. 3). Among the differentially expressed transcripts in leaves, were genes involved in photosynthetic and photorespiration pathways, electron transport chain, oxidation–reduction and energy derivation by oxidation processes. Other photosynthesis-related GOs were also enriched by salinity treatment, such as those involved in the molecular functions, electron carrier and tetrapyrrole photoreceptors binding activities. Additionally, GOs involved in different plastid components were overrepresented in the library (Fig. 3). Mapping of the leaf enzymes on the metabolic pathways using the KEGG tool revealed the distribution of these sequences on 98 different metabolic pathways (Additional file 5: Table S2). The list included 26 genes coding for 17 enzymes mapped on starch and sucrose metabolic pathways, 15 genes coding for six enzymes mapped on the oxidative phosphorylation, and 10 genes coding for 10 enzymes in the carbon fixation pathways (Additional file 6: Figure S4).

The differentially expressed gene list included a group of transcript orthologues previously characterized in response to salt stress in other plant species. The short list included ten salt-induced genes, a putative sodium hydrogen exchanger-8 and two genes coding for a probable sodium-coupled neutral amino acid transporter-6, two potassium channel AKT-2 and 3 analogues, a probable potassium transporter-9, a cation-chloride cotransporter 1-like, a potassium efflux antiporter 4-like isoform x3 and abscisic acid receptor pyl-1, 4 and 8 (Additional file 4: Table S1).

### DEGs in roots

**Fig. 4:**
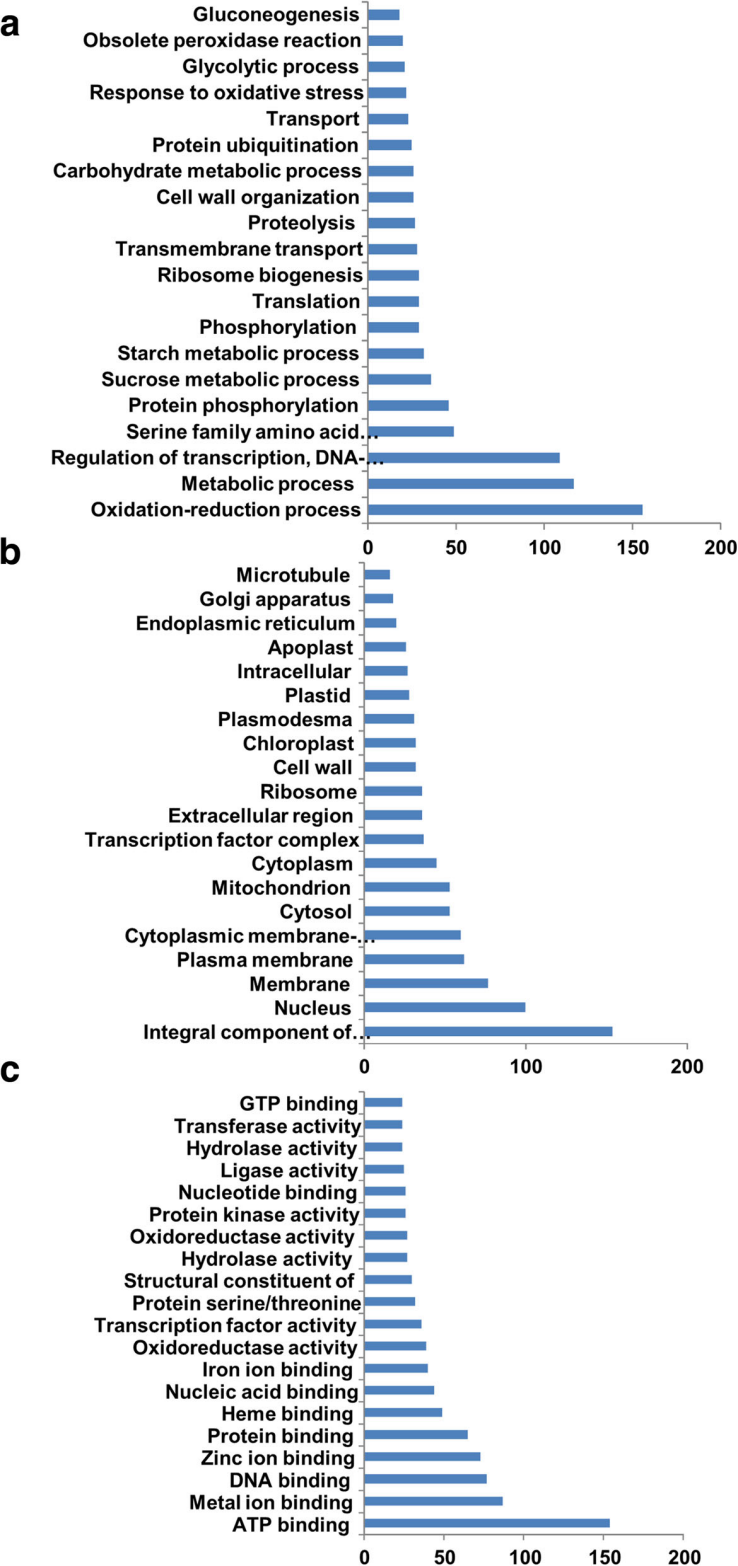
The Distribution of GO categories between the major three GO domains under salinity stress condition in date palm roots. The genes were classified based on the biological processes (**a**), cellular components (**b**), and molecular functions (**c**). The bar represents the number of sequences in different GO categories

**Fig. 5:**
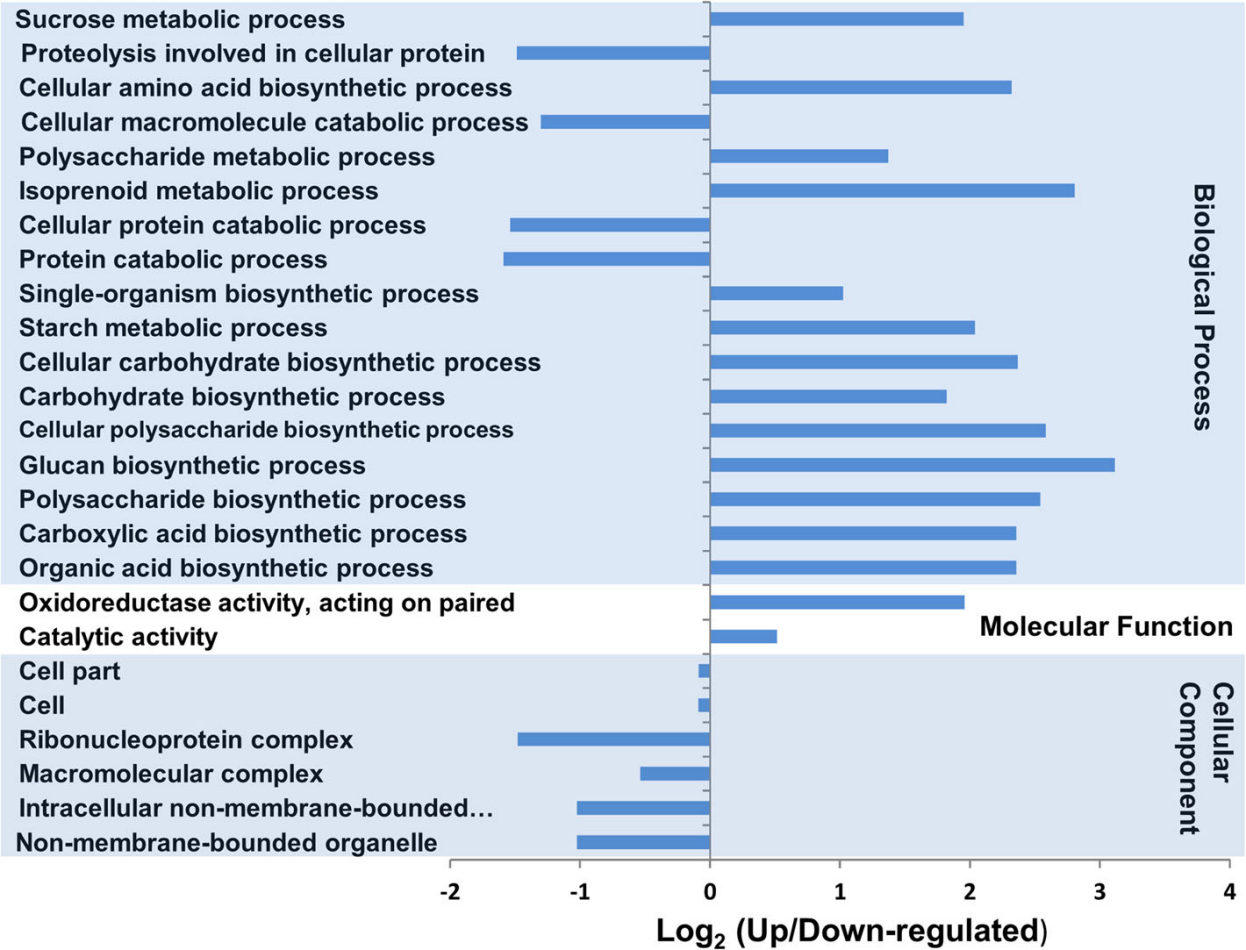
The distribution of DEGs among the GO categories in root tissues obtained using the Fisher’s exact test. The bar represents the log_2_ transformed ratio between category portions in the differentially expressed gene sets. The ratio greater than zero indicates that there were more genes upregulated by salinity stress in the particular category. Significantly (*p* ≤ 0.001) enrich categories are shown

Differential gene expression analysis of the roots revealed a total of 4684 (2297 upregulated and 2387 downregulated) transcripts whose relative expression level was significantly changed ≥2 fold (*p*, FDR < 0.05) during salinity (Additional file 7: Table S3). Functional annotation of these differentially regulated genes indicated that genes falling into several GO categories were altered due to salinity stress. These include oxidation-reduction, regulation of transcription, transmembrane transport and proteolysis processes (Fig. 4a); plasma membrane, transcription factor complex, apoplast cellular components (Fig. 4b); and ATP, ions, metal, nucleic acid binding and transfer molecular functions (Fig. 4c). Fisher’s extract test for statistical significance (*p* < 0.01) assessment of GO term enrichment showed that there were 28 differentially enriched gene ontologies in the libraries, including 15 GO terms enriched in salinity-treated roots and 13 GO terms enriched in the control plants (Fig. 5). Mapping of the differentially expressed enzymes on the metabolic pathways revealed the presence of 166 different mappeable enzymes on 111 metabolic pathways, some of which are important in salinity tolerance in plants (Additional file 8: Table S4). These metabolic pathways include purine, thiamine, inositol phosphate and tryptophan metabolism and phenylpropanoid biosynthesis pathways (Additional file 9: Figure S5).

A list of candidate genes that are potentially important for salinity tolerance are included in the differentially expressed transcripts. This short list included genes whose expression was previously shown to be affected by salinity stress: 5 sodium ion transporter coding genes, including a HKT1 sodium ion transporter; 10 transmembrane potassium ion transporters, a chloride ion transporter, 8 H^+^/K^+^ antiporters, and four abscisic acid receptors, two abscisic acid insensitivity-5 proteins and an abscisic acid-inducible kinase (Additional file 7: Table S3).

### Transcripts commonly expressed in leaves and roots upon exposure to salinity

Sequence analysis revealed 194 differentially expressed transcripts in both leaf and root tissues. These transcripts included a putative potassium transporter 8, an abscisic acid receptor PYR1 and 4, an indole-3-acetic acid-amido synthetase GH3, a pyrophosphate-energized vacuolar membrane proton pump previously shown to be induced by salt stress (Additional file 10: Table S5).

### Verification of the expression level of some transcripts using qPCR

**Fig. 6:**
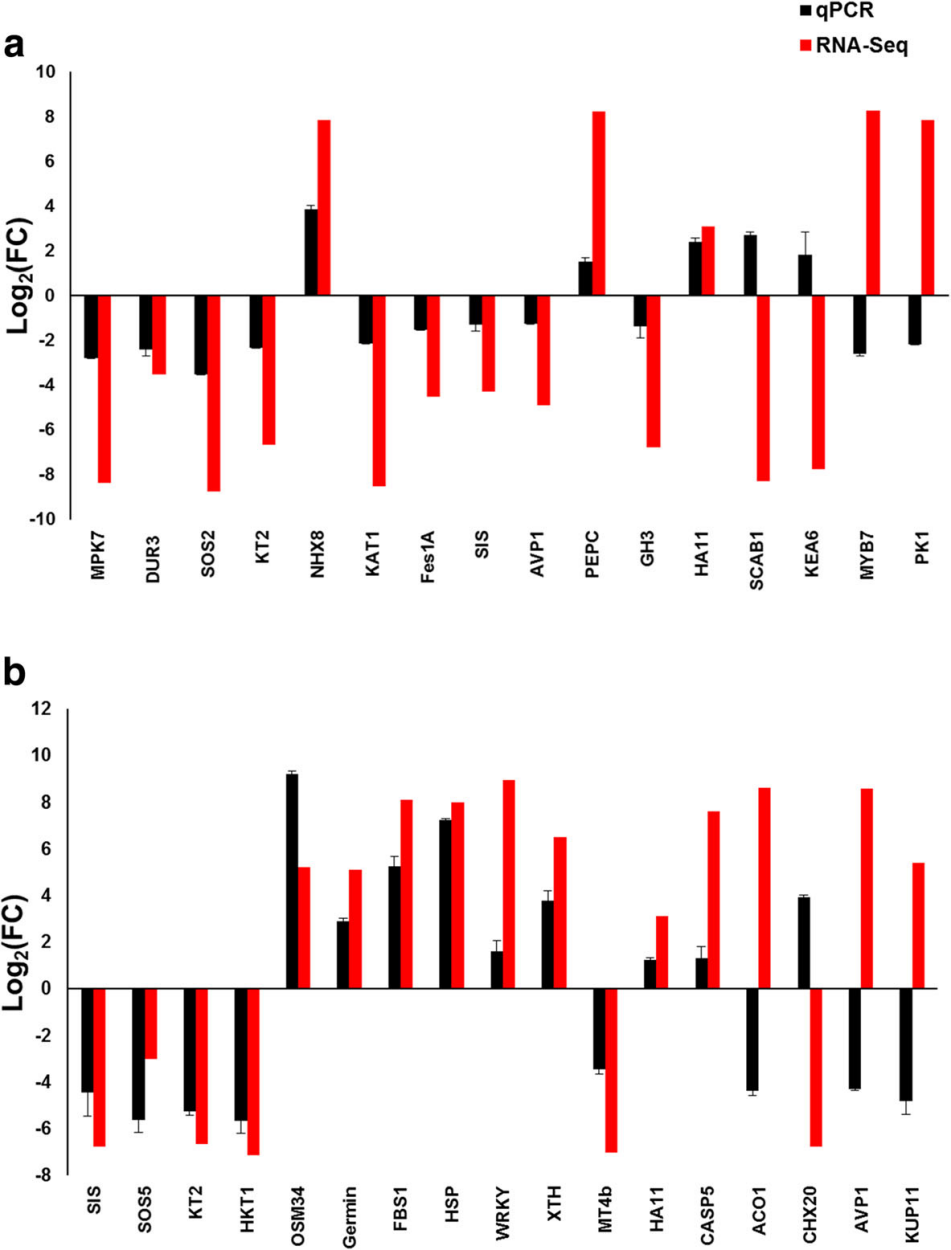
Differential gene expression value of selected genes obtained by total RNA sequencing (RNA-seq) and qPCR in leaf (**a**) and root (**b**) tissues of plants treated with 300 mM NaCl for 10 days. Bars represent mean log_2_ fold change ± SE (*n* = 3)

Stable housekeeping (reference) genes in response to salinity in leaf and root tissues were selected and used to verify the expression level of a group of differentially expressed transcripts identified using the total RNA sequencing method, and which have a potential role in salt tolerance, using the qPCR. The results indicated that the salt-stress induced changes in gene expression for majority (~70%) of the tested genes was consistent between both approaches (RNA-seq and qPCR), and in leaves (Fig. 6a) and roots (Fig. 6b). The correlation coefficient (r) between qPCR- and FPKM-derived expressions for leaf and root were 0.91 and 0.86, respectively (Additional file 11: Figure S6), indicating high reliability of the RNA-seq data.

## Discussion

Date palm (*Phoenix dactylifera* L., Arecaceae) is a relatively salt-tolerant plant capable of growing up to 9 dS/m salinity level with no significant growth penalties [32], but the molecular bases of this salinity tolerance have not been investigated. It is only recently that the genome of date palm has been sequenced [33–35] alongside the mitochondrial [36] and chloroplastic [37] genomes, with the latter two revealing intra- and inter-varietal variations in the genome sequences. These variations could be useful in understanding cultivar differences in stress tolerance. Recently, in an attempt to elucidate the molecular mechanisms of salt tolerance in date palm, Radwan et al., [28] studied the transcriptomic changes in the root system of date palm (cv. *Deglet Beida*) seedlings after removed from the soil and exposed to 150 mM NaCl solution for only 2 and 4 h. The results yielded stress-responsive genes that can aid in understanding stress tolerance pathways, but the study was limited by the fact that the duration of the stress for a few hours and so genes that are induced mostly represent osmotic stress genes rather than salt-specific genes, which can only be identified by relatively prolonged stress treatments, usually after several days [30]. Moreover, the study by Radwan et al., [28] only analyzed responses in the root system hence the responses in leaves were not analyzed. The leaf is the hub of most metabolic processes in plants, including photosynthesis. The goal of the present study was to analyze transcriptomic changes in roots and leaves of the date palm cv. *Khalas*, exposed to salinity (irrigated with 300 mM NaCl to the soil) over a relatively longer period of time (10 days) in order to obtain salt-specific genes, in addition to osmotic stress ones. The transcriptomic data in the present study indicated that stress-responsive genes are mainly associated with photosynthetic and photorespiratory pathways in leaves, while transcription regulation, transmembrane transport, apoplastic component, proteolysis, and ATP and metal binding pathways were found in the roots. DEGs from these pathways that are involved in stress tolerance were selected from the leaf and root profiles and validated by qPCR (Fig. 6).

### DEGs in leaves

In the leaf, 52% of the DEGs were upregulated while 48% were downregulated (Additional file 4: Table S1).

Most of the DEGs in the leaf (52%) were upregulated (Additional file 4: Table S1). GO enrichment analysis revealed that most of these upregulated genes are involved in photosynthetic and respiratory metabolisms. For specific cases, the chlorophyll a and b binding proteins, for example CP47, of the light harvesting complex II, which serve as conduits for excitation energy to the reaction centers of PSII [36], were upregulated in the present study. In addition, photosystem I and II proteins were also upregulated, suggesting an enhanced photosynthetic efficiency. The photosynthetic electron transport chain is one of the main sources of reactive oxygen species (ROS) production in plants as a result of stress-induced leakage of electrons to oxygen (O_2_), which can seriously affect photosynthesis by damaging the photosynthetic machinery. NADH plastoquinone oxidoreductase was previously shown to reduce photoinhibition of photosynthesis induced by light and ozone in barley [38]. The upregulation of this enzyme, as observed in this study, may protect the photosynthetic machinery against stress. Furthermore, enzymes that are involved in glycolytic pathways (which use hexoses from starch degradation to generate ATP, reducing power, pyruvate and various anabolic products), such as the chloroplastic and cytosolic glyceraldehyde-3-phosphate dehydrogenase (GAPDH), were enhanced in this study (Additional file 6: Figure S4). The rice cytosolic GAPDH (OsGAPC3) was shown to improve salt tolerance in rice by mainly reducing ROS accumulation [38], while the chloroplastic GAPC3 was shown to be important for root development, through serine synthesis and transport in *Arabidopsis* [39]. This glycolytic process appears to have been unperturbed by salt stress, since hexose carrier genes were also upregulated.

Surprisingly, phosphoenol pyruvate carboxylase (PEPC) was also upregulated in the current study. This enzyme catalyzes the first carboxylation reaction in C_4_ and CAM photosynthetic pathways. Given that date palm is a C_3_ plant, this occurrence raises the possibility of salinity-induced C_4_ or CAM pathways, which will be worthwhile investigating. The induction of these pathways has been shown to be important in salt tolerance [40]. However, although the genes of the photosynthetic and associated pathways were upregulated, the photosynthetic rate was severely reduced by salt stress. This reduction can be attributed to processes including damage to photosynthetic machinery, as a result of photoinhibition, and to reduced stomatal conductance. To determine whether salt stress compromised the photosynthetic machinery, QY and NPQ were measured (Fig. 1). The results showed that the photosynthetic machinery was not affected, as QY was not altered by salt stress, while NPQ increased, indicating sufficient energy dissipation in the system, similar to the observation made by Sperling and his coworkers [20] on another date palm cultivar. It is therefore likely that the drastic reduction in stomatal conductance in the present study (Fig. 1) should be the cause of the reduced photosynthetic rate, corroborating results on other date palm cultivars [20, 41]. In these other studies, no damage to the photosynthetic apparatus, alteration of electron transfer or affected carboxylation, was observed suggesting that stomatal conductance generally limits photosynthesis in date palm under salt stress [42]. 

Furthermore, genes linked to ion transport in the leaf tissue, for example *KT2* and *KAT*, were downregulated except for *NHX8. NHX8* is plasma membrane H^+^/cation antiporter similar TO SALT OVERLY SENSITIVE 1 (SOS1), a Na^+^ extruding antiporter, however contrary to SOS1, its function has been shown to involve Li^+^ and not Na^+^ extrusion out of cell [43]. KAT1 is a guard cell membrane-localized potassium channel that is one of the main channels involved in K^+^ influx in guard cells during stomatal opening [44]. Therefore, its down regulation in the present study will imply a defect in proper stomatal aperture regulation and consequently related metabolic activities such as stomatal conductance to CO_2_ diffusion for photosynthesis, although it has been shown that KAT1 is not essential for stomatal opening [45]. In addition, K^+^ distribution may be generally affected, as other K^+^ transporters such as KT2 is also down regulated. Accordingly, it is possible that vacuolar K^+^ pools may also be affected, following the downregulation of serine threonine protein kinase (SOS2), which is involved in both SOS1 and NHX activation by phosphorylation to extrude Na^+^ out of the cell, and for vacuolar Na^+^ and K^+^ sequestration, respectively [46]. This is further supported by the downregulation of AVP1, a vacuolar pyrophosphatase that is involved in the regulation of vacuolar pH, and hence in regulating the activity of NHX in Na^+^ or K^+^ compartmentation [47]. These results suggest that high salinity may negatively affect ion homeostasis in date palm leaves. Furthermore, kinases such as the mitogen-activated protein kinase, MAPK (MPK7), and protein kinases PK (PK1) were also downregulated, indicating that posttranslational regulation of many proteins will be affected. Nitrogen metabolism in the leaf may also be affected by the downregulation of DUR3, a urea transporter [48]. Under salt stress, high temperature can also account for sensitivity. Therefore enhanced thermotolerance can also lead to enhanced salt tolerance. The Arabidopsis AtFes1A protein is involved in thermotolerance by stabilizing Hsp70 proteins, as shown in *atfes1a* knock out mutants, which had increased salt sensitivity and reduced thermotolerance [49]. This susceptibility was accounted for by increased ubiquitination of Hsp70 proteins, which are involved in thermotolerance. In the present study, *Fes1A* is downregulated (Fig. 6a), suggesting that high temperature would affect date palm growth under high salinity.

### DEGs in roots

Using next generation sequencing, salt stress-induced changes in gene expression profiles in roots of date palm were reported recently [28]. In the reported study, 6-week-old seedlings were exposed to hydroponic medium containing 150 mM NaCl for 2 and 4 h and analyzed the transcriptomic changes. The authors observed that after 2 h of stress, only 10% of the DEGs were upregulated and that after 4 h some of the previously upregulated genes were downregulated [28]. Thirty to 60% of these DEGs were grouped into the functional category, catalysis. Contrarily, in the present study, after subjecting date palm seedlings to a longer period (10 days) of stress 49% of the DEGs were upregulated. This difference is not surprising given the fact that the differences in stress intensity and duration could affect expression of salt-responsive genes differently [50]. The differences could also be due to differences in genotype/cultivar used for the study [51]. Date palm is relatively salt-tolerant suggesting that it would normally require stronger and gradual stress imposition to activate stress-responsive genes.

In the present study, genes of the transmembrane transport category that are important for stress tolerance were upregulated (Additional file 5: Table S2). They include the potassium transporter (KT2) and the potassium uptake transporter 11 (KUP11), which are important for K^+^ homeostasis under salt stress, the nitrate transporter, which is important for root to shoot nitrate transport in plants [52], sulphate transporter, and ABC (ATP binding cassette) transporters. However, some isoforms of genes coding transmembrane proteins, such as KT2, KUP11 and HKT1 are downregulated (Fig. 6b). The downregulation of *KT2* suggests inhibition of K^+^ uptake and therefore altered K^+^ homeostasis under the present stress condition. The downregulation of *HKT1* on the other hand would indicate unregulated Na^+^ transport from root to shoot of *Khalas*. HKT1s (e.g. AtHKT1;1 and OsHKT1;5) are mainly Na^+^-selective transporters localized to the xylem/symplast boundary, where they mediate Na^+^ retrieval from xylem into xylem parenchyma cells, thus reducing leaf Na^+^ accumulation [53, 54]. These results suggest that under high salinity, cation transporters may not be very crucial for stress tolerance at least in this date palm cultivar.

Beside ion transport, genes related to osmoregulation are also induced in the root of date palm. These include osmotin34 (OSM34) (Fig. 6b). *OSM34* is localized to endomembranes, whose product is secreted in the apoplast and have roles in osmotic adaptation [55]. In the same light, the F-Box stress induced 1 (FBS1, a member of the SCF ubiquitin E3 ligase complex), for example, the Arabidopsis *AtFBS1*, was shown to be induced by salt stress and sorbitol [56], suggesting a role in osmotic stress tolerance. Similarly *FBS1* was upregulated in the present study, indicating enhanced adaptation to osmotic stress by date palm under high salinity.

Furthermore, induction of heat shock proteins (HSPs) could be of potential benefit to date palm under high salinity. HSPs are involved in stabilization of proteins and membranes as well as in protein folding, assembly and translocation, and have been shown to impart tolerance to high temperature, water deficit, salinity and osmotic stress [57]. For example Augustine and his coworkers [58] showed that when *Erianthus arundinaceus* Hsp70 (*EaHsp70*) was overexpressed in a sugarcane, the transformed plants acquired resistance to salinity and drought.

In a root bending assay to determine stress tolerance pathways in Arabidopsis, Shi et al. [59] discovered the SOS5 gene, encoding a protein similar to arabinoglycan-like protein (AGP, involved in cell-to-cell adhesion), whose mutation exhibited distorted root tip phenotype, and hence poor root development compared to WT. This gene was found to play an essential role in cell wall formation and cell expansion in root tip. It was later found that in addition to its role in root development, it is also implicated in the formation of the mucilage layer of the seed coat in Arabidopsis, where mutants showed alteration in cellulosic rays of the mucilage layer [60]. In the current study, SOS5 appears downregulated in the root, suggesting an aberration in cell wall integrity and a possible increased susceptibility of root cells to high salt concentrations.

These results indicate that under high salinity, osmotic and oxidative stresses would be major challenges for date palm, which it counteracts by upregulating the corresponding genes.

In addition, protein kinases involved in signal transductions eliciting stress defense, such as the SOS2, which is involved in the SOS1-mediated Na^+^ extrusion pathway [15], the Ca-dependent protein kinases, and the mitogen-activated protein kinase were also upregulated [61, 62]. Furthermore, proton ATPases are important in generating electrochemical gradients across membranes, which are important for ion uptake, solute transport, and cell wall growth [38]. Various types of ATPases such as the tonoplast v-type ATPase involved in Na^+^ sequestration into vacuoles and plasma membrane Ca-transporting ATPases, which are implicated in salt stress tolerance [50] were upregulated in this study (Additional file 5: Table S2). Also of significance was the induction of casparian strip membrane protein 5 (CASP5). CASPs have been shown to be involved in buildup of the casparian strip [51, 63], which is a barrier to apoplastic flow of solutes to the stellar region of the root and is important in salt stress tolerance [64]. Salt stress often induces osmotic effects that inhibit water uptake by reducing hydraulic conductance [30]. The upregulation of aquaporin genes in root of date palm in this study suggests a potential increase in water uptake and regulated hydraulic conductance [62].

Various transcription factors and transcription regulation genes were also strongly induced by salt stress (Additional file 5: Table S2). Among these genes is the homeobox leucine zipper protein. The Arabidopsis homeobox leucine zipper protein, Athb-12, has been shown to improve resistance to salt stress by regulating Na^+^ exclusion in yeast [65]. The MADS box transcription factor is also implicated in salt tolerance [66], while the base helix-loop-helix (bHLH) transcription factor that controls root growth, hence increasing tolerance in *Medicago truncatula* to salt stress [67] was also upregulated in this study. In addition, the WRKY transcription factor was also significantly induced. The overexpression of soybean WRKY (*GmWRKY*) in *Arabidopsis* improved tolerance to salinity by enhancing lateral root development [68].

DEGs coding antioxidants that are known to be important in salt stress tolerance were significantly upregulated in the current study (Additional file 5: Table S2). These include phenylalanine ammonium lyase, peroxidase and superoxide dismutase. This enhanced antioxidant gene expression indicates enhanced tolerance to oxidative stress induced by high salt concentrations in the root. Also, the upregulation of germin-like protein8-5 in the present study is an indication of induction of oxidative stress defense mechanisms. Germin-like proteins of *Arachis hypogoea* were shown to have SOD enzyme activity as well as capacity to induce non-enzyme antioxidant genes when overexpressed in Arabidopsis [69]. In addition, some of the DEGs in root tissues were mapped on the pathways associated with antioxidant metabolites such as thiamine and ascorbate (Additional file 9: Figure S5 and Additional file 8: Table S4).

Other upregulated genes that have been shown to enhance tolerance to salinity include the enzyme xyloglucan endotransglycosylase (XTH), which is involved in cell wall loosening, root elongation and hypocotyl elongation. The overexpression of the *Capsicum annum* XTH (*CaXTH*) in *Arabidopsis* was shown to improve tolerance to drought and high salinity [70].

Most of the upregulated genes in the present study were previously shown to be downregulated in date palm seedlings shocked with 150 mM NaCl for 2 and 4 h [28]. This marked difference in response might be due to the shock that the plants received from the high salt concentration and probably consequences such as deactivation of enzymes and damage to membranes [71]. Moreover, the difference between these DEGs could also be due to a low stringent filtration strategy used in that study, as these DEGs are included within a list of 1939 genes whose expression value was altered by ±15 folds in response to salinity [34]. For example, all antioxidant enzymes (SOD, CAT, APX, and POD) were downregulated in that study, indicating insufficient scavenging capacity [72]. Also, the downregulation of SOS2, which is an important signaling protein involved in Na^+^ exclusion at the root, indicates the possibility of excess accumulation of Na^+^ in the root tissues. Furthermore, many transmembrane transporters were down regulated, indicating a consequential nutrient deficiency in the plant. For example, the downregulation of the nitrate transporter will impair nitrate uptake and subsequently all biosynthetic pathways requiring N, such as protein and nucleic acid synthesis. These results suggest the relevance of subjecting plants to gradually increasing levels of salinity to enable adaptation.

However, common features between the two studies include the downregulation of the chloroplastic K^+^ efflux antiporters and metallothioneins. Metallothioneins are involved in ROS scavenging as well as metal chelation, hence improving tolerance to salt and heavy metal stress [68].

### Common DEGs between leaves and roots

Other genes that have previously been shown to be important for salt stress adaptation such as the auxin-responsive gene, Gretchen Hagen3 (GH3), are also downregulated in the current study in both leaves and roots. Different classes of these genes were found to be upregulated in the leaf of *Sorghum bicolor* under salt and drought stress and thought to be important for growth and development [70]. Another common salt stress-responsive gene between the two tissues is glutathione-S-transferase, which has been shown to promote root and shoot growth under salt and drought stress [41]. In addition, the vacuolar proton ATPase, which pumps H^+^ into the vacuoles, thus generating an electrochemical H^+^ gradient, which serves as a driving force for vacuolar sequestration of ions and solutes was also induced. The induced expression of this gene has been shown to be important for salt stress tolerance in cotton [47]. Also, remorin, which was induced in both tissues, has been shown to improve drought and salt tolerance, by mediating responses to osmotic stress [40]. Furthermore, Protein ubiquitination (ubiquitin mediated degradation of selected proteins) is important for abiotic stress adaptation as it helps prevent accumulation of proteins damaged by the stresses. SIS (salt-induced serine-rich) is an E3 ubiquitin protein ligase that is induced by salt and drought stress and involved in ABA-dependent/independent pathways [73]. *SIS* was downregulated in both tissues in the present study, suggesting altered ABA signaling for proper stomatal regulation under high salinity.

## Conclusions

The photosynthetic parameters (A, gs, E and ci) were negatively affected by the salinity treatments in date palm seedlings. The reduction in A has been mainly attributed to reduced stomatal conductance to CO_2_. In this regard, the leaf transcriptome data revealed the downregulation of *SIS*, an E3 ubiquitin ligase that is involved in ABA signaling, which could negatively alter stomatal functions. Still in relation to photosynthesis, an interesting observation in the leaf transcriptome data was the upregulation of *PEPC*, a C4 photosynthetic pathway enzyme that catalyzes the first carboxylation in mesophyll cells. This pathway has not been reported in date palm and therefore needs to be investigated, especially to understand its role in salt stress adaptation in the plant. The root transcriptomic data was essentially different from that of the leaf in that the main salt stress tolerance genes were associated with osmotic stress (e.g. *FBS1*, *OSM34*), oxidative stress (e.g., germin-like protein8-5), and heat stress (e.g., *HSP*) components. Further studies are required to functionally characterize these genes and to establish the salt tolerance mechanism in date palm and subsequently to utilize those genes or gene-associated markers to improve date palm’s production on saline soils, where they commonly grow, like in Oman.

## Methods

### Plant growth conditions and salt treatment

Date palm seeds (*Phoenix dactylifera* L. cv. *Khalas*) were surface sterilized, planted, salinity treated and incubated under the same environmental conditions previously described [27]. Briefly, seeds were sown in artificial nutrient soils and regularly irrigated to field capacity with deionized water for eight weeks. Subsequently, two groups of 9 plants each were either continually watered with deionized water (control plants) or with 300 mM NaCl solution every 2 days for one more week (salinity-treated plants). Three days after the last irrigation, leaf and root tissues were harvested, thoroughly cleaned with tap water, dried by tissue papers and flash frozen in liquid nitrogen.

### Photosynthesis and chlorophyll fluorescence measurements

In order to determine the effect of the salinity treatments on photosynthetic processes, the photosynthesis parameters of the plants under control and salinity treatments were measured 9 days after start of treatment. Net photosynthetic rate (A), stomatal conductance (gs), transpiration (E), and internal CO_2_ concentration (ci) were measured, using the LCpro-SD instrument (ADC Bioscientific Ltd., United Kingdom). The chlorophyll fluorescence of dark-adapted leaves was measured, using FluoroPen FP100 (Photon System Instruments, Czech Republic), and the quantum yield of PSII (QY), and non-photochemical quenching (NPQ) were recorded.

### RNA extraction, library construction and RNA sequencing

Leaf and root tissues, obtained from six seedlings grown under control or salt stress conditions, were pooled. The total RNA from these tissues was extracted using RNeasy Plant Mini Kit (Qiagen) following the manufacturer’s instructions. The resulting RNA was treated with DNase (Qiagen, USA) and the quality and quantity of the extracted RNA were assessed using an Agilent 2100 Bioanalyzer (Agilent). Next generation sequencing was performed by Macrogen (South Korea). The mRNA libraries were constructed, using the reagents provided in the Illumina ® TruSeq™ RNA Sample Preparation Kit, and following the manufacturer’s instructions. Briefly, RNA with poly‐A containing mRNA molecules was isolated using poly‐T oligo‐attached Ampure XP magnetic beads. After isolation and fragmentation, the cleaved RNA fragments were copied into first strand cDNA, using reverse transcriptase and random hexamer primers. Subsequently, a second cDNA strand was prepared using DNA Polymerase I and RNase H. After the end repair process, using end repair (ERP) mix and 3' ends adenylation, adaptors were ligated and the products were then purified and enriched with PCR, using PCR primer cocktail to generate the final cDNA library. After passing the quality control, cDNA molecules were clustered and sequenced, using the HiSeq 2000 Illumina Solexa’s sequencing system.

### RNA sequence assembly and differential gene expression analysis

The sequence quality was verified using SolexaQA Perl-based and FastQC software packages (www.bioinformatics.bbsrc.ac.uk/projects/fastqc/). Because of the lack of a completely assembled genome of date palm, *de novo* sequence assembling strategy was used in this protocol. Spliced junctions of the RNA-Seq reads were mapped, assembled (paired-end 101 bp), estimated for their relative abundances, and tested for differential expression and regulation, using TopHat (version 1.3.3) [74], Trinty and Cufflinks (version 1.2.1) [75] software packages, set on default options and following the protocol [76]. The transcripts were counted at isoform level and the relative transcript abundances were measured and expressed in Fragments per kilobase pair of exon per million fragments mapped (FPKM).

The expression values of each transcript were normalized using the following equation:

$$ {s}_j\kern0.5em =\kern0.5em \underset{i}{\mathrm{median}}\kern0.5em \left[\frac{c_{i j}}{{\left({\displaystyle {\prod}_{k=1}^m{c}_{i j}}\right)}^{1/ m}}\right] $$

Where *S*_*j*_ is the library size parameter; *c*_*ij*_ is the count number of sequence *i* of sample *j*; and *m* is the total number of samples associated with the analysis.

After eliminating the sequences that did not show read counts in all the libraries, the Differential Expression Analysis of Digital Gene Expression Data software (edgeR) [77] was used to statistically validate the differentially normalized expression level of the transcripts in response to salinity treatment. The Biological coefficient of variation (BCV) value was set to 0.2 according to the software’s instructions. Differentially expressed transcripts were identified based on the multiple test corrected *P*-value and the False Discovery Rate (FDR) was < 0.05.

### Functional annotation and analysis of the transcripts

Differentially expressed transcripts (FC ≥ 2) with a significant value (*p*-value and FDR < 0.05) in roots and leaves were searched against the non-redundant protein databases of the Basic Local Alignment Search Tool BLASTX (cut off E^-6^ ≤) and InterPro motifs using the tools implemented in Blast2Go software package [78, 79]. The results obtained from BLASTX and InterPro motifs databases were combined and sequences were assigned to certain classes of GO. Subsequently, GO annotations were augmented by annex [80], using the tools implemented within the Blast2Go software. Annotations were classified into biological processes, molecular functions and cellular components. GO enrichment analysis was performed using Fisher’s exact test and the data were filtered based on *p* ≤ 0.01. Transcript coding enzymes were mapped on the Kyoto Encyclopedia of Genes and Genomes (KEGG) [81] software implemented within the Blast2Go software.

### Validation of the gene expression using reverse transcriptase real time PCR (qPCR)

The cDNA was synthesized from the total RNA of three independent biological replicates, isolated from leaf and root tissues, using SuperScript™ IV First-Strand Synthesis System (Life Technologies, USA). The synthesized cDNA was used in a qPCR reaction containing Fast SYBR® Green Master Mix (Applied Biosystem, USA) and a pair of primers (Table 2), and the reaction carried out using the CFX96 Touch™ Real-Time PCR Detection System (BioRad, USA). The gene expression was normalized using 3 different stable reference genes for each tissue. Reference genes used for qPCR normalization were selected based on the previously published work [82]. The ribosomal subunit genes 18S rRNA and 25S rRNA, and the splicing-associated factor (YT521-B) were used as stable reference genes in leaves. However, the elongation factor 1A (eEF1a), ubiquitin (UBQ) and actin genes were used as stable reference genes in roots. The data from the qPCR reaction was analyzed and gene expression was calculated using the 2^-∆∆CT^ method [83]. The Pearson correlation coefficient was used to correlated the qPCR-derived expression and RNA-seq-derived expression (FPKM) [84].

**Table 2:** Oligo nucleotides used in the qPCR

Names	Forward (5’ – 3’)	Reverse (5’ – 3’)
*MPK7*	CCCGGTGGGCTTAAGTTGA	CATTTGGCAATGTGGGATTCT
*DUR3*	CACAACATGGGCAGCAGTTC	AGCACATACTGTTTGCGAGATTG
*SOS2*	GGTTGGGTAACGGGTTTATGG	CCCGGCACTCCCACTTC
*KT2*	CGGTCCAAAGCATCATATGGT	CATTCACCCCCATCCATTGA
*NHX8*	CCAGGTCCAGCAAGTAGTAGCA	GCCATGGAAGTGCACCAAAT
*KAT1*	TCTGAGATACCTGCCCACGAA	AACCGCAAAAAAGCTGTTGAC
*Fes1A*	CGAGGCTCCACTTGAGCAA	AAGAATCGATGGCGAACGAT
*SIS*	GCCGGTCCCTTATTTCCTTCT	GCGGTGAGCGCCTGAA
*AVP1*	GGTGGAGGTTGAGTTTTCGTTT	CGCCGCTCCCATTGC
*KEA6*	GCGACCAGATCGATGGAGAA	TCTCGGCAACGCTGTTGTT
*MYB7*	TGCTGTCCAAGCTCCTCTATTG	CAGCATGGGAAGGAAACCA
*PK1*	TCAAACAACAGGATGCCAACA	CTCGGAAAAGGCCACGATAA
*PEPC*	TGAAAATTCTGCAGCGACTGA	AGCTGCACCACAAGCCTCTT
*SCAB1*	CAGGATCTATCGGACTGGTTGTT	GCAAGCAGAGATCGTTTTAAATGA
*GH3*	CACCGCCGCAACGTAGTC	CTTGAGGAGGTCTTCTTCATTGG
*HA11*	CAAAAGGACTGCTCTAACATATATCGA	TGGTGCACCTTTGCTGACA
*SIS*	GCCGGTCCCTTATTTCCTTCT	GCGGTGAGCGCCTGAA
*SOS5*	GAGGAGGGCTGTGACATTAACC	CCTATTCTCCTCTTTCTTTTACTTCTTCTT
*HKT1*	CCCGGTGGGCTTAAGTTGA	CATTTGGCAATGTGGGATTCT
*OSM34*	CCCTGAACGAGGGAGATGTC	GGAATTCGCCCTCAACCAGTA
*Germin*	CGGTCTTCTTGTCCAATTGAAAG	GCGAAGCCACCAATCTCTGA
*FBS1*	GTGGACCGTTGCAAAGGAA	AGCAACTCGAGGCGATTAGATC
*HSP*	AACTGTGACCGTGCCCAAA	TGGAGATCTCAATGGCCTTCA
*ACO1*	TGGGCAGTGAGTTGCAATATCT	TGGGATCTCCATTTTCTCCAA
*CHX20*	GGCCGGAACAAGACGTACCT	ATCGGCGTGCTCCATGA
*AVP1*	AGCTTCGGAGCATGCTAGGA	GCAGCCTTGTGGGCATCT
*KUP11*	GCTCTTGCACTTGGCTGTTTT	CCGAGGAACTTTTTTGATGTATGA
*WRKY*	GACGGTCACGTTGGATCTGA	GCTGGTAGCCTTTGGAATTGG
*XTH*	AGCTTGATCTTCATGCTGACATG	GCGGGTTCGCTTCGAAT
*MT4b*	AGACACAACCTGTGCTCGTG	ACCGAGACTGCTCCTCTCAA
*18S rRNA*	CGAACCACTGCGAAAGCAT	CCCCCAACTTTCGTTCTTGA
*25S rRNA*	CCACCGTCCTGCTGTCTTAATC	CGCGCCAACCCAGATC
*YT521-β*	CATTCGCAATAGCTCGCTTCT	GCAGCTGGTGATGCCTTGA
*eEF1α*	GATCCCTTCCTACACTCGAATCC	TCCTTTCCCATTGGTATTTGCT
*UBQ*	CCGACACCATCGACAACGT	GCGGGATGCCCTCCTT
*Actin*	TCAATGTGCCTGCCATGTATGT	GCGGCCGCTAGCATAGAG

## Additional files

Additional file 1: **Figure S1.** The percent distribution of global transcriptome abundance according to the three GO domains in salinity-stressed date palm leaves (A) and roots (B). (TIF 737 kb)

Additional file 2: **Figure S2.** The percent distribution of functionally annotated and DEGs across the transcriptome specifically involved in the biological process, cellular components and molecular functions in salinity-stressed date palm leaves. (TIF 1762 kb)

Additional file 3: **Figure S3.** The percent distribution of functionally annotated and DEGs across the transcriptome specifically involved in the biological process, cellular components and molecular functions in salinity-stressed date palm roots. (TIF 1514 kb)

Additional file 4: **Table S1.** DEGs and their predicted function in leaves. Only significantly (*p*, FDR < 0.05) altered genes are shown. (XLSX 468 kb)

Additional file 5: **Table S2.** Mapping of the DEGs in isolated from leaf tissues on the metabolic pathways using the KEGG database. (XLSX 27 kb)

Additional file 6: **Figure S4.** Mapping of differentially expressed enzymes in leaves due to salinity stress on the KEGG. (PDF 5590 kb)

Additional file 7: **Table S3.** DEGs and their predicted function in roots. Only significantly (*p*, FDR < 0.05) altered genes are shown. (XLSX 670 kb)

Additional file 8: **Table S4.** Mapping of the DEGs in isolated from root tissues on the metabolic pathways using the KEGG database. (XLSX 32 kb)

Additional file 9: **Figure S5.** Mapping of differentially expressed enzymes in roots due to salinity stress on the KEGG. (PDF 6204 kb)

Additional file 10: **Table S5.** DEGs expressed in both root and leaf tissues and their predicted function. Only significantly (*p*, FDR < 0.05) altered genes are shown. (XLSX 35 kb)

Additional file 11: **Figure S6**. Correlation between RNA-seq and qPCR-derived expression of selected DEGs from leaves (A) and roots (B). r is the Pearson correlation coefficient. (TIF 194 kb)

## Abbreviations

ATP: Adenosine triphosphate
DEGs: Differentially Expressed Genes
dS.m^-1^: deci Siemens per meter
FRD: False Discovery Rate
GAPDH: Glyceraldehyde 3-phosphate dehydrogenase
GO: Gene Ontology
KEGG: Kyoto Encyclopedia of Genes and Genomes
NADH: Nicotinamide adenine dinucleotide
NPQ: Non-photochemical quenching
PEPC: Phosphoenol pyruvate carboxylase
PSII: photosystem II
qPCR: Reverse transcriptase-quantitative polymerase chain reaction
RNA-seq: RNA sequencing
ROS: Reactive oxygen species
SOD: Superoxide dismutase
