# Supplementary figures and images for: Genome-wide expression profiling in leaves and roots of date palm (Phoenix dactylifera L.) exposed to salinity
Source: BMC Genomics. 2017 Mar 22;18:246. doi: 10.1186/s12864-017-3633-6 (PMC5423419; doi:10.1186/s12864-017-3633-6)

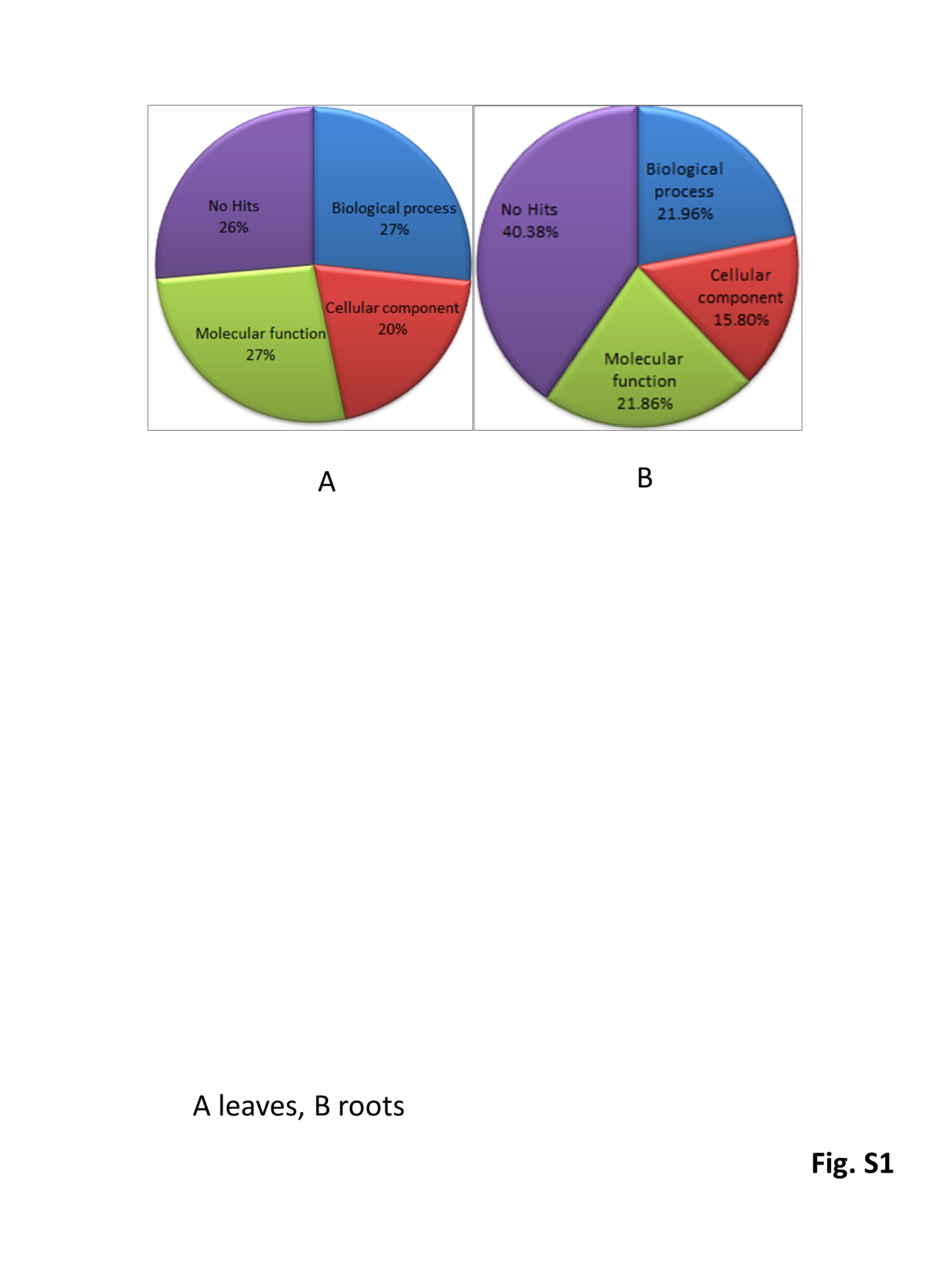

Supplement: Supplementary file 1 — Figure S1. The percent distribution of global transcriptome abundance according to the three GO domains in salinity-stressed date palm leaves (A) and roots (B). (TIF 737 kb) [file 12864_2017_3633_MOESM1_ESM.tif]

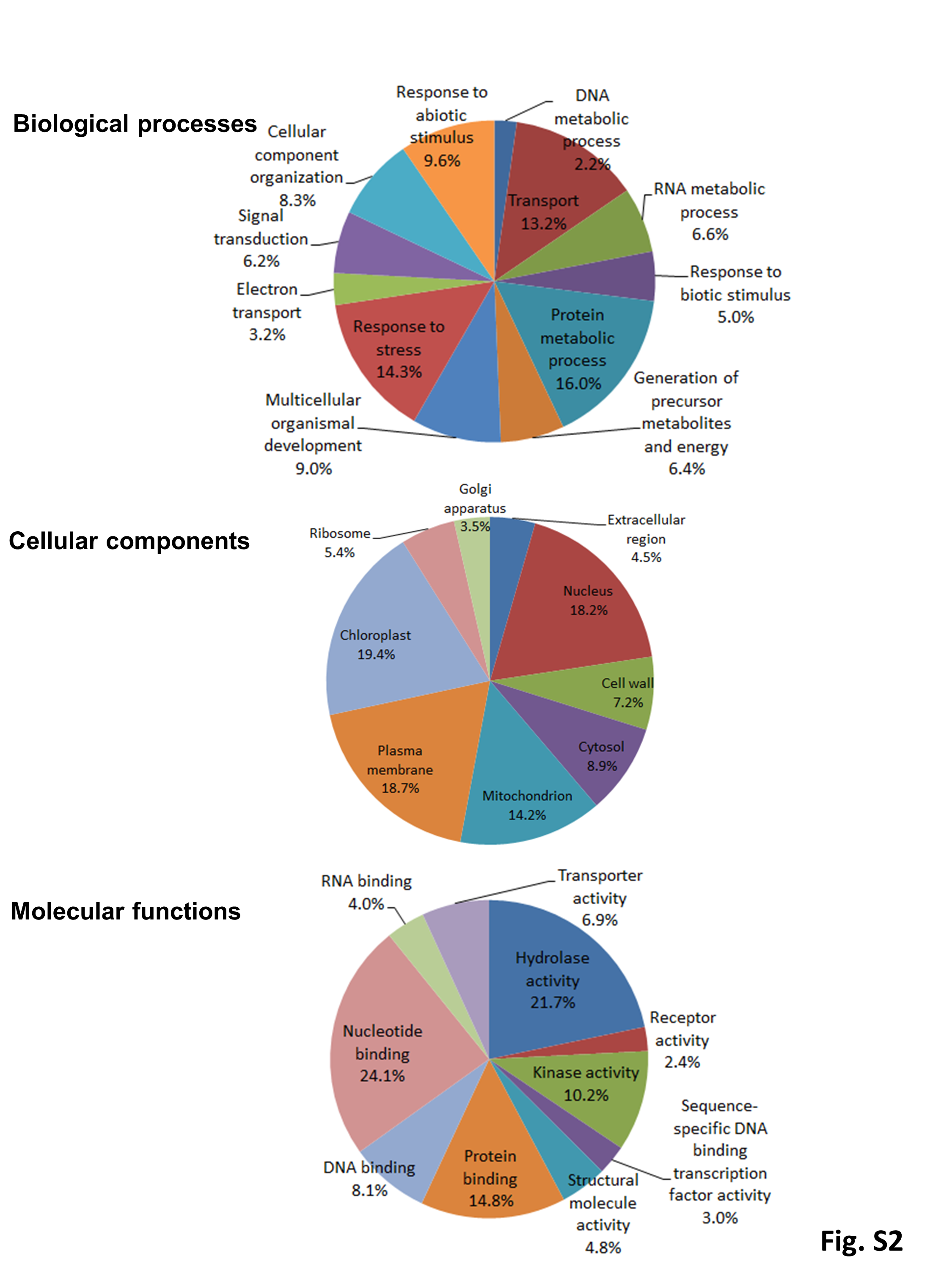

Supplement: Supplementary file 2 — Figure S2. The percent distribution of functionally annotated and DEGs across the transcriptome specifically involved in the biological process, cellular components and molecular functions in salinity-stressed date palm leaves. (TIF 1762 kb) [file 12864_2017_3633_MOESM2_ESM.tif]

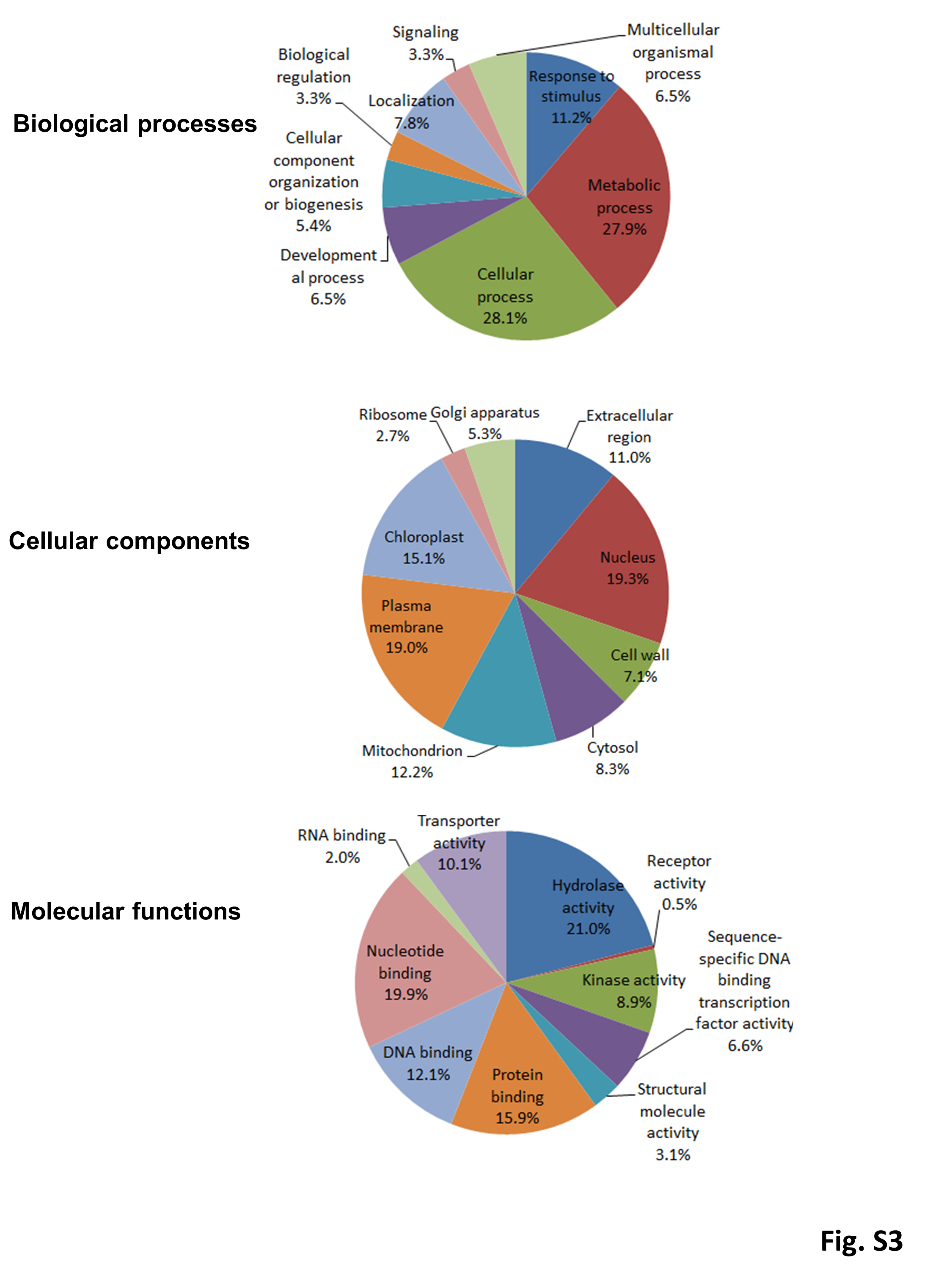

Supplement: Supplementary file 3 — Figure S3. The percent distribution of functionally annotated and DEGs across the transcriptome specifically involved in the biological process, cellular components and molecular functions in salinity-stressed date palm roots. (TIF 1514 kb) [file 12864_2017_3633_MOESM3_ESM.tif]

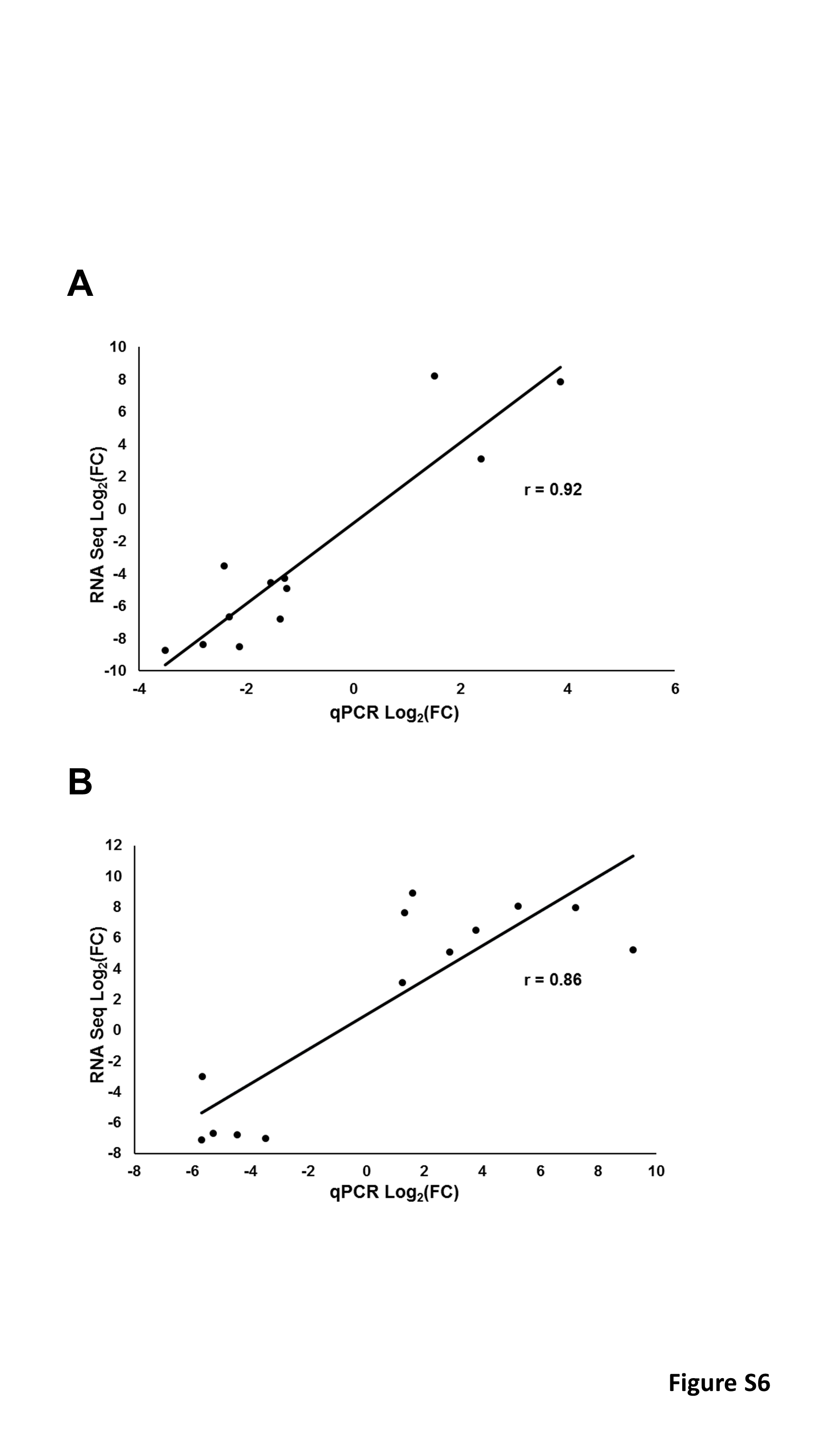

Supplement: Supplementary file 11 — Figure S6. Correlation between RNA-seq and qPCR-derived expression of selected DEGs from leaves (A) and roots (B). r is the Pearson correlation coefficient. (TIF 194 kb) [file 12864_2017_3633_MOESM11_ESM.tif]
