# Supplementary material for: Genome-wide expression profiling in leaves and roots of date palm (Phoenix dactylifera L.) exposed to salinity
Source: BMC Genomics. 2017 Mar 22;18:246. doi: 10.1186/s12864-017-3633-6 (PMC5423419; doi:10.1186/s12864-017-3633-6)
Supplement: Supplementary file 6 — Figure S4. Mapping of differentially expressed enzymes in leaves due to salinity stress on the KEGG. (PDF 5590 kb) [file 12864_2017_3633_MOESM6_ESM.pdf]

Figure S4. Mapping of differentially expressed enzymes in leaves due to salinity stress on the KEGG.

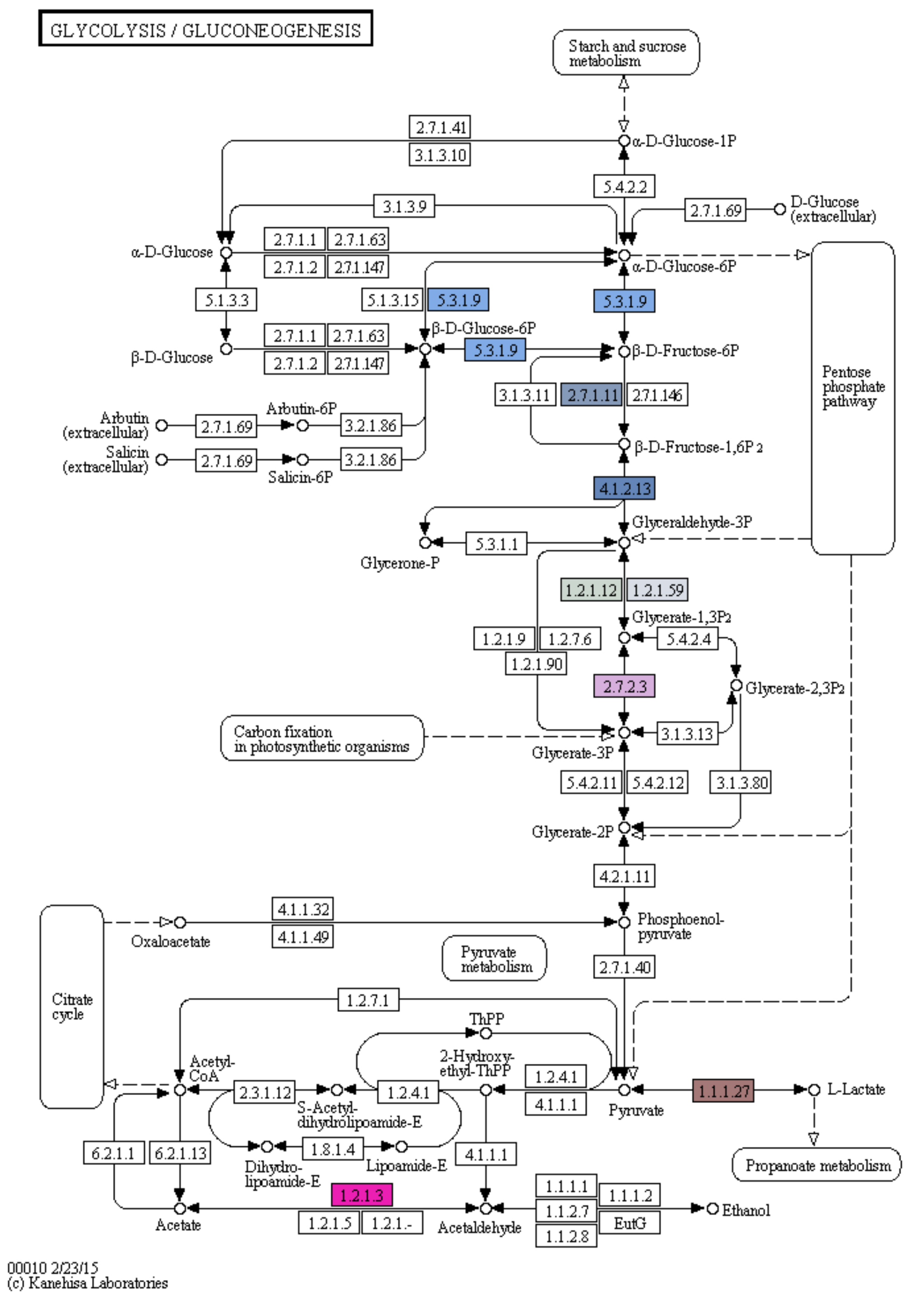
GLYCOLYSIS / GLUCONEOGENESIS
Starch and sucrose metabolism
α-D-Glucose-1P
2.7.1.41
3.1.3.10
5.4.2.2
3.1.3.9
2.7.1.69
D-Glucose (extracellular)
α-D-Glucose
2.7.1.1
2.7.1.63
2.7.1.2
2.7.1.147
α-D-Glucose-6P
5.1.3.3
5.1.3.15
5.3.1.9
β-D-Glucose-6P
β-D-Glucose
β-D-Fructose-6P
Pentose phosphate pathway
3.1.3.11
2.7.1.11
2.7.1.146
Arbutin (extracellular)
Arbutin-6P
3.2.1.86
Salicin (extracellular)
Salicin-6P
β-D-Fructose-1,6P2
4.1.2.13
Glyceraldehyde-3P
5.3.1.1
Glycerone-P
1.2.1.12
1.2.1.59
Glycerate-1,3P2
1.2.1.9
1.2.7.6
5.4.2.4
1.2.1.90
2.7.2.3
Glycerate-2,3P2
Carbon fixation in photosynthetic organisms
3.1.3.13
Glycerate-3P
5.4.2.11
5.4.2.12
3.1.3.80
Glycerate-2P
4.2.1.11
4.1.1.32
4.1.1.49
Oxaloacetate
Phosphoenol-pyruvate
Citrate cycle
Pyruvate metabolism
2.7.1.40
1.2.7.1
ThPP
2-Hydroxy-ethyl-ThPP
Acetyl-CoA
2.3.1.12
1.2.4.1
4.1.1.1
Pyruvate
1.1.1.27
L-Lactate
S-Acetyl-dihydrolipoamide-E
6.2.1.1
6.2.1.13
1.8.1.4
Dihydro-lipoamide-E
Lipoamide-E
Propanoate metabolism
1.2.1.3
1.1.1.1
1.1.1.2
1.1.2.7
EutG
1.1.2.8
Acetate
1.2.1.5
1.2.1.-
Acetaldehyde
Ethanol
00010 2/23/15
(c) Kanehisa Laboratories

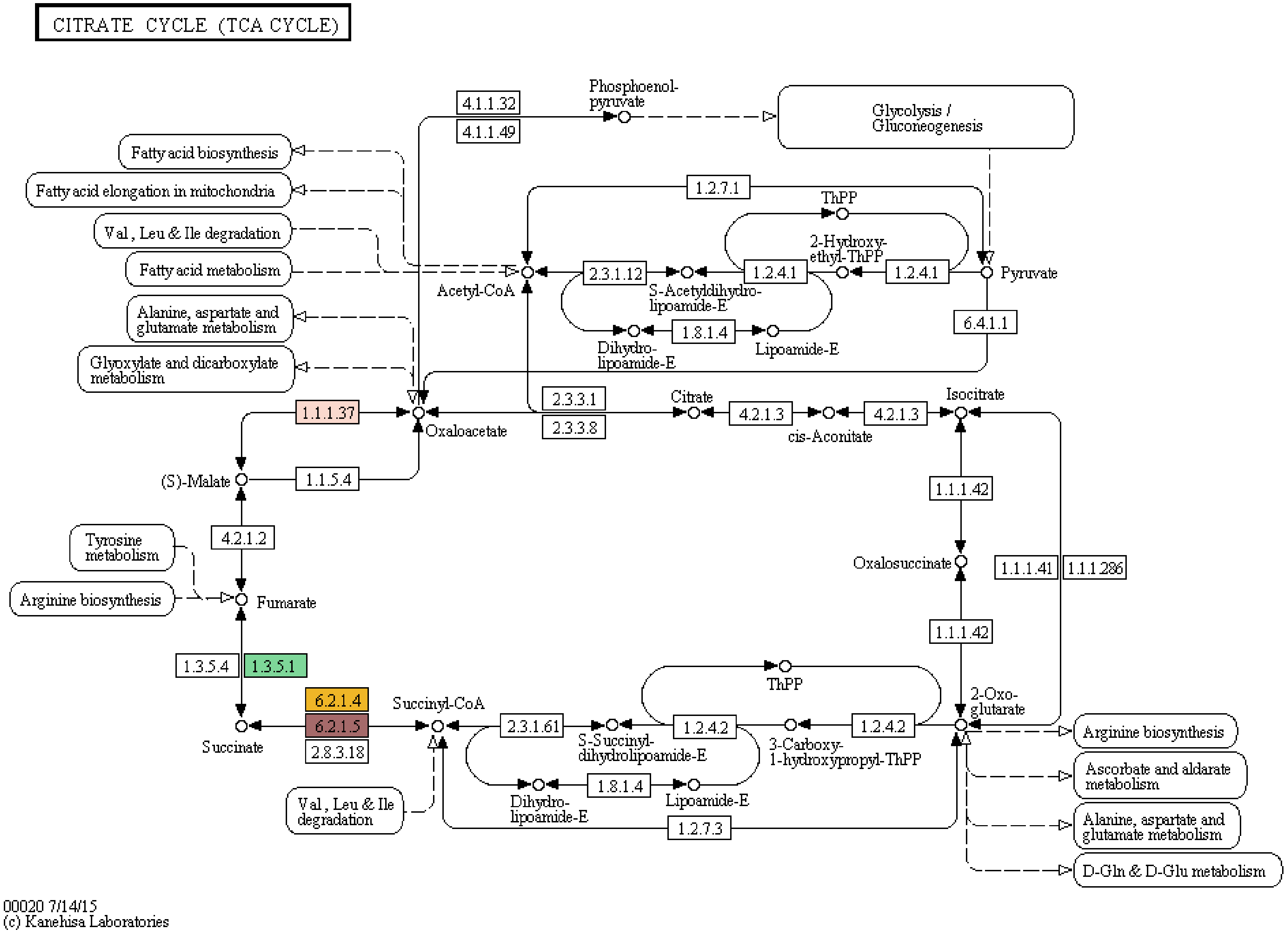
CITRATE CYCLE (TCA CYCLE)
4.1.1.32
4.1.1.49
Phosphoenol-pyruvate
Glycolysis / Gluconeogenesis
Fatty acid biosynthesis
Fatty acid elongation in mitochondria
Val, Leu & Ile degradation
Fatty acid metabolism
Alanine, aspartate and glutamate metabolism
Glyoxylate and dicarboxylate metabolism
1.2.7.1
ThPP
2-Hydroxy-ethyl-ThPP
Acetyl-CoA
2.3.1.12
1.2.4.1
1.2.4.1
Pyruvate
S-Acetyldihydro-lipoamide-E
1.8.1.4
Dihydro-lipoamide-E
Lipoamide-E
6.4.1.1
2.3.3.1
2.3.3.8
Citrate
4.2.1.3
4.2.1.3
cis-Aconitate
Isocitrate
1.1.1.37
Oxaloacetate
(S)-Malate
1.1.5.4
1.1.1.42
4.2.1.2
Tyrosine metabolism
Oxalosuccinate
1.1.1.41
1.1.1.286
Arginine biosynthesis
Fumarate
1.1.1.42
1.3.5.4
1.3.5.1
ThPP
6.2.1.4
Succinyl-CoA
2-Oxo-glutarate
6.2.1.5
2.3.1.61
1.2.4.2
1.2.4.2
Succinate
2.8.3.18
S-Succinyl-dihydrolipoamide-E
3-Carboxy-1-hydroxypropyl-ThPP
Arginine biosynthesis
Ascorbate and aldarate metabolism
Val, Leu & Ile degradation
Dihydro-lipoamide-E
1.8.1.4
Lipoamide-E
1.2.7.3
Alanine, aspartate and glutamate metabolism
D-Gln & D-Glu metabolism
00020 7/14/15
(c) Kanehisa Laboratories

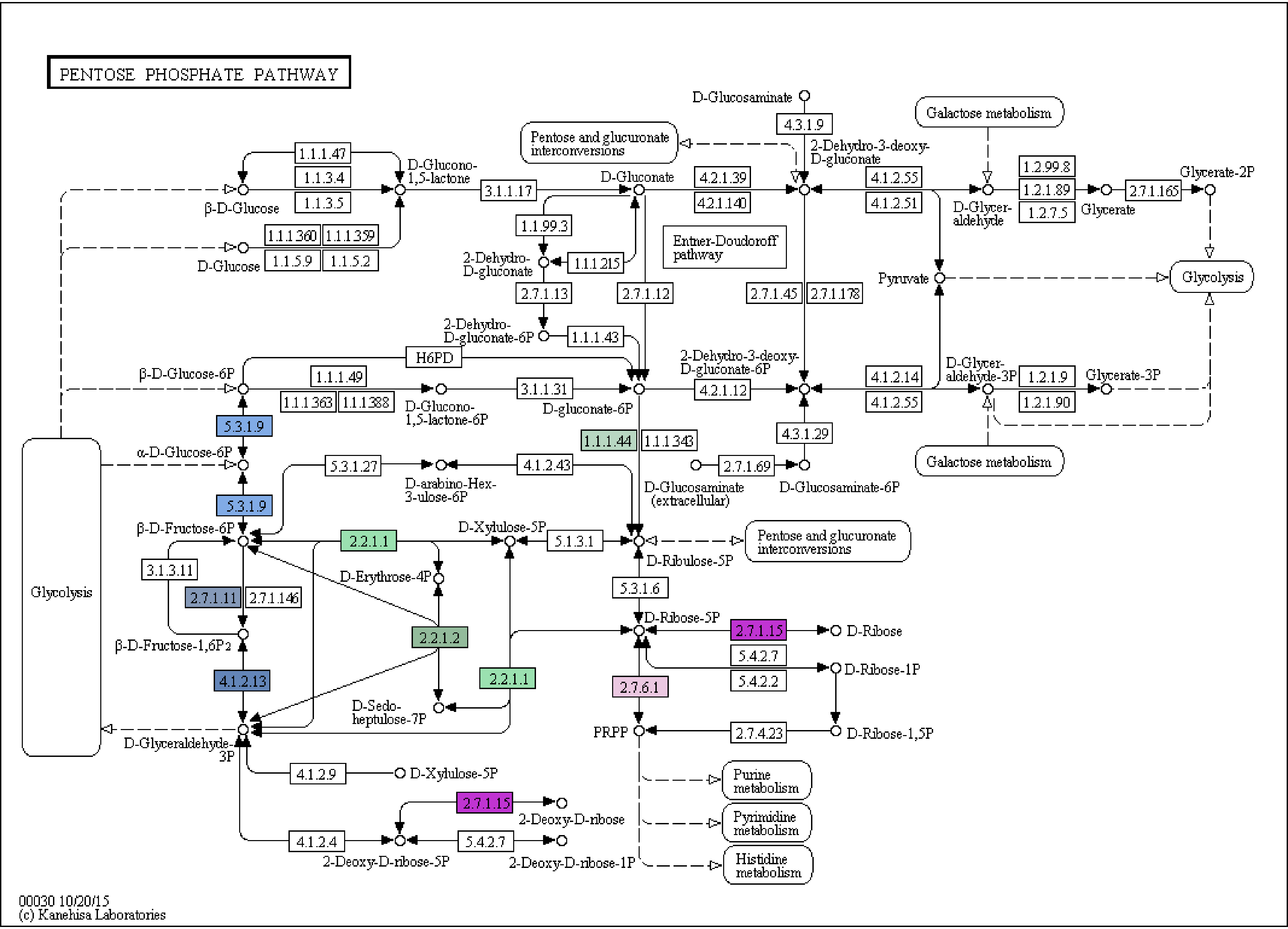
PENTOSE PHOSPHATE PATHWAY
D-Glucosaminate
4.3.1.9
Galactose metabolism
Pentose and glucuronate interconversions
2-Dehydro-3-deoxy-D-gluconate
1.1.1.47
D-Glucono-1,5-lactone
1.1.3.4
3.1.1.17
D-Gluconate
4.2.1.39
4.1.2.55
1.2.99.8
Glycerate-2P
1.1.3.5
4.2.1.140
4.1.2.51
1.2.1.89
2.7.1.165
β-D-Glucose
D-Glycer-aldehyde
1.2.7.5
Glycerate
1.1.1.360
1.1.1.359
1.1.99.3
Entner-Doudoroff pathway
D-Glucose
1.1.5.9
1.1.5.2
2-Dehydro-D-gluconate
1.1.1.215
Pyruvate
Glycolysis
2.7.1.13
2.7.1.12
2.7.1.45
2.7.1.178
2-Dehydro-D-gluconate-6P
1.1.1.43
H6PD
2-Dehydro-3-deoxy-D-gluconate-6P
D-Glycer-aldehyde-3P
β-D-Glucose-6P
1.1.1.49
4.1.2.14
1.2.1.9
Glycerate-3P
3.1.1.31
4.2.1.12
1.1.1.363
1.1.1.388
D-Glucono-1,5-lactone-6P
D-gluconate-6P
4.1.2.55
1.2.1.90
5.3.1.9
1.1.1.44
1.1.1.343
4.3.1.29
α-D-Glucose-6P
5.3.1.27
4.1.2.43
2.7.1.69
Galactose metabolism
D-arabino-Hex-3-ulose-6P
D-Glucosaminate (extracellular)
D-Glucosaminate-6P
5.3.1.9
β-D-Fructose-6P
2.2.1.1
D-Xylulose-5P
5.1.3.1
Pentose and glucuronate interconversions
D-Ribulose-5P
3.1.3.11
D-Erythrose-4P
Glycolysis
2.7.1.11
2.7.1.146
5.3.1.6
D-Ribose-5P
2.7.1.15
D-Ribose
β-D-Fructose-1,6P2
2.2.1.2
5.4.2.7
2.2.1.1
D-Ribose-1P
4.1.2.13
5.4.2.2
2.7.6.1
D-Sedo-heptulose-7P
PRPP
2.7.4.23
D-Ribose-1,5P
D-Glyceraldehyde-3P
4.1.2.9
D-Xylulose-5P
Purine metabolism
2.7.1.15
2-Deoxy-D-ribose
Pyrimidine metabolism
4.1.2.4
5.4.2.7
2-Deoxy-D-ribose-5P
2-Deoxy-D-ribose-1P
Histidine metabolism
00030 10/20/15
(c) Kanehisa Laboratories

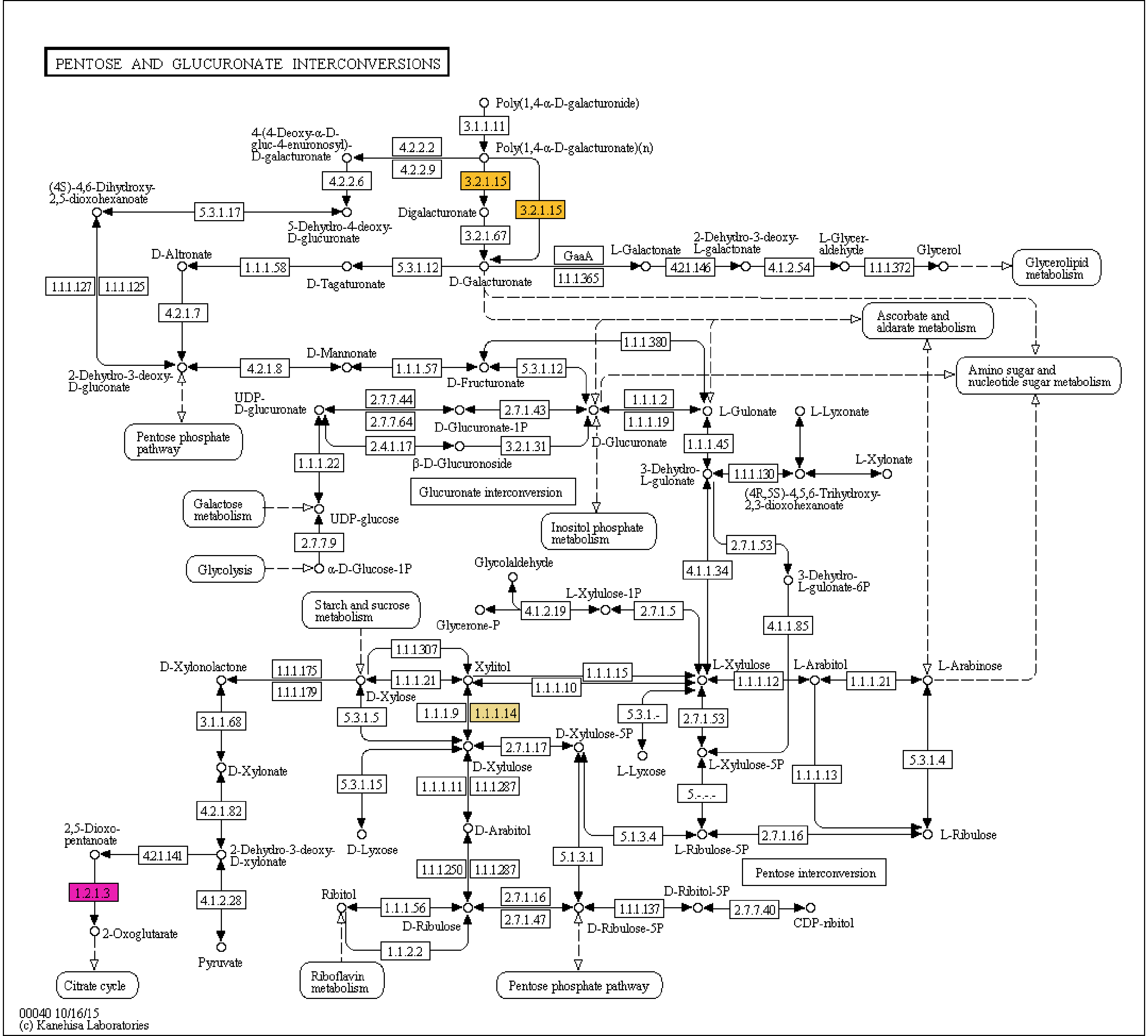
PENTOSE AND GLUCURONATE INTERCONVERSIONS
Poly(1,4-α-D-galacturonide)
3.1.1.11
Poly(1,4-α-D-galacturonate)(n)
4-(4-Deoxy-α-D-gluc-4-enuronosyl)-D-galacturonate
4.2.2.2
4.2.2.9
3.2.1.15
3.2.1.15
4.2.2.6
(4S)-4,6-Dihydroxy-2,5-dioxohexanoate
5.3.1.17
5-Dehydro-4-deoxy-D-glucuronate
Digalacturonate
3.2.1.67
D-Altronate
1.1.1.58
D-Tagaturonate
5.3.1.12
D-Galacturonate
GaaA
1.1.1.365
L-Galactonate
2-Dehydro-3-deoxy-L-galactonate
4.2.1.146
4.1.2.54
L-Glyceraldehyde
1.1.1.372
Glycerol
Glycerolipid metabolism
1.1.1.127
1.1.1.125
4.2.1.7
Ascorbate and aldarate metabolism
1.1.1.380
2-Dehydro-3-deoxy-D-gluconate
4.2.1.8
D-Mannonate
1.1.1.57
D-Fructuronate
5.3.1.12
Amino sugar and nucleotide sugar metabolism
UDP-D-glucuronate
2.7.7.44
2.7.7.64
D-Glucuronate-1P
2.7.1.43
1.1.1.2
1.1.1.19
L-Gulonate
L-Lyxonate
Pentose phosphate pathway
2.4.1.17
β-D-Glucuronoside
3.2.1.31
D-Glucuronate
1.1.1.45
1.1.1.22
3-Dehydro-L-gulonate
1.1.1.130
L-Xylonate
(4R,5S)-4,5,6-Trihydroxy-2,3-dioxohexanoate
Galactose metabolism
UDP-glucose
Glucuronate interconversion
Inositol phosphate metabolism
2.7.7.9
2.7.1.53
Glycolysis
α-D-Glucose-1P
Glycolaldehyde
4.1.1.34
3-Dehydro-L-gulonate-6P
Starch and sucrose metabolism
L-Xylulose-1P
4.1.2.19
2.7.1.5
Glycerone-P
4.1.1.85
1.1.1.307
D-Xylonolactone
1.1.1.175
1.1.1.179
Xylitol
1.1.1.15
L-Xylulose
L-Arabitol
L-Arabinose
1.1.1.21
1.1.1.10
1.1.1.12
1.1.1.21
D-Xylose
1.1.1.9
1.1.1.14
5.3.1.5
3.1.1.68
5.3.1.-
2.7.1.53
2.7.1.17
D-Xylulose-5P
D-Xylonate
D-Xylulose
L-Lyxose
L-Xylulose-5P
5.3.1.4
5.3.1.15
1.1.1.11
1.1.1.287
1.1.1.13
4.2.1.82
5.-.-.-
2,5-Dioxopentanoate
D-Lyxose
D-Arabitol
5.1.3.4
2.7.1.16
L-Ribulose
4.2.1.141
2-Dehydro-3-deoxy-D-xylonate
L-Ribulose-5P
5.1.3.1
Pentose interconversion
1.2.1.3
1.1.1.250
1.1.1.287
4.1.2.28
Ribitol
1.1.1.56
2.7.1.16
2.7.1.47
D-Ribitol-5P
1.1.1.137
2.7.7.40
CDP-ribitol
2-Oxoglutarate
D-Ribulose
D-Ribulose-5P
Pyruvate
1.1.2.2
Citrate cycle
Riboflavin metabolism
Pentose phosphate pathway
00040 10/16/15
(c) Kanehisa Laboratories

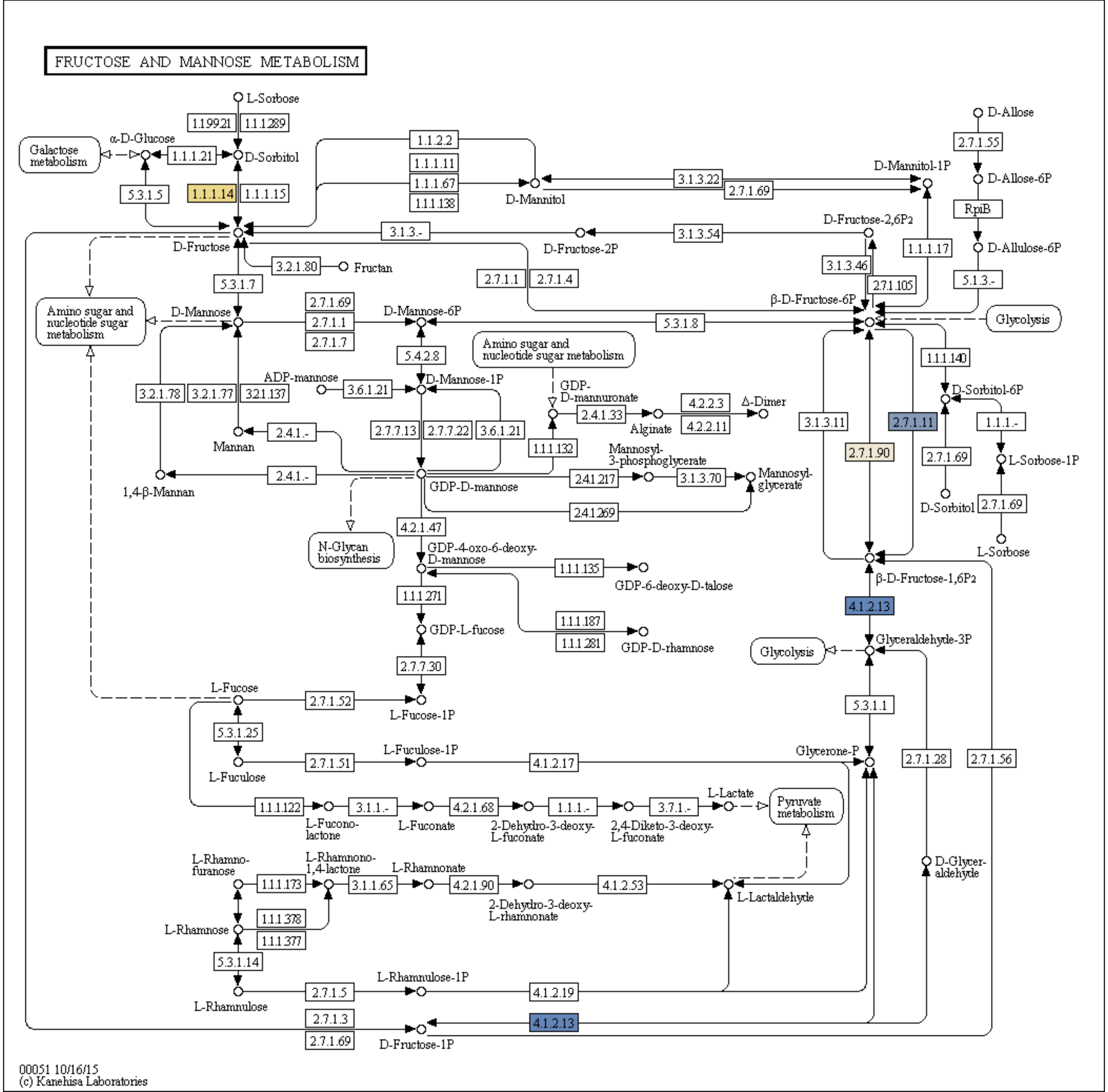
FRUCTOSE AND MANNOSE METABOLISM
L-Sorbose
1.1.99.21
1.1.1.289
α-D-Glucose
Galactose metabolism
1.1.1.21
D-Sorbitol
5.3.1.5
1.1.1.14
1.1.1.15
1.1.2.2
1.1.1.11
1.1.1.67
1.1.1.138
D-Mannitol
3.1.3.22
2.7.1.69
D-Mannitol-1P
D-Allose
2.7.1.55
D-Allose-6P
RpiB
D-Allulose-6P
5.1.3.-
D-Fructose
3.1.3.-
D-Fructose-2P
3.1.3.54
D-Fructose-2,6P2
3.1.3.46
2.7.1.105
1.1.1.17
3.2.1.80
Fructan
5.3.1.7
2.7.1.1
2.7.1.4
β-D-Fructose-6P
Glycolysis
Amino sugar and nucleotide sugar metabolism
D-Mannose
2.7.1.69
2.7.1.1
2.7.1.7
D-Mannose-6P
5.3.1.8
5.4.2.8
1.1.1.140
D-Sorbitol-6P
ADP-mannose
3.6.1.21
D-Mannose-1P
GDP-D-mannuronate
4.2.2.3
Δ-Dimer
2.4.1.33
Alginate
4.2.2.11
3.2.1.78
3.2.1.77
3.2.1.137
3.1.3.11
2.7.1.11
1.1.1.-
Mannan
2.4.1.-
2.7.7.13
2.7.7.22
3.6.1.21
1.1.1.132
2.7.1.90
2.7.1.69
L-Sorbose-1P
Mannosyl-3-phosphoglycerate
1,4-β-Mannan
2.4.1.-
GDP-D-mannose
2.4.1.217
3.1.3.70
Mannosyl-glycerate
2.4.1.269
D-Sorbitol
2.7.1.69
L-Sorbose
N-Glycan biosynthesis
4.2.1.47
GDP-4-oxo-6-deoxy-D-mannose
1.1.1.135
GDP-6-deoxy-D-talose
β-D-Fructose-1,6P2
4.1.2.13
1.1.1.271
1.1.1.187
1.1.1.281
GDP-D-rhamnose
GDP-L-fucose
Glyceraldehyde-3P
2.7.7.30
5.3.1.1
L-Fucose
2.7.1.52
L-Fucose-1P
5.3.1.25
L-Fuculose-1P
2.7.1.51
4.1.2.17
Glycerone-P
2.7.1.28
2.7.1.56
L-Fuculose
1.1.1.122
3.1.1.-
4.2.1.68
1.1.1.-
3.7.1.-
L-Lactate
Pyruvate metabolism
L-Fucono-lactone
L-Fuconate
2-Dehydro-3-deoxy-L-fuconate
2,4-Diketo-3-deoxy-L-fuconate
L-Rhamno-furanose
L-Rhamnono-1,4-lactone
L-Rhamnonate
1.1.1.173
3.1.1.65
4.2.1.90
4.1.2.53
L-Lactaldehyde
D-Glycer-aldehyde
2-Dehydro-3-deoxy-L-rhamnonate
1.1.1.378
L-Rhamnose
1.1.1.377
5.3.1.14
L-Rhamnulose-1P
2.7.1.5
4.1.2.19
L-Rhamnulose
2.7.1.3
4.1.2.13
2.7.1.69
D-Fructose-1P
00051 10/16/15
(c) Kanehisa Laboratories

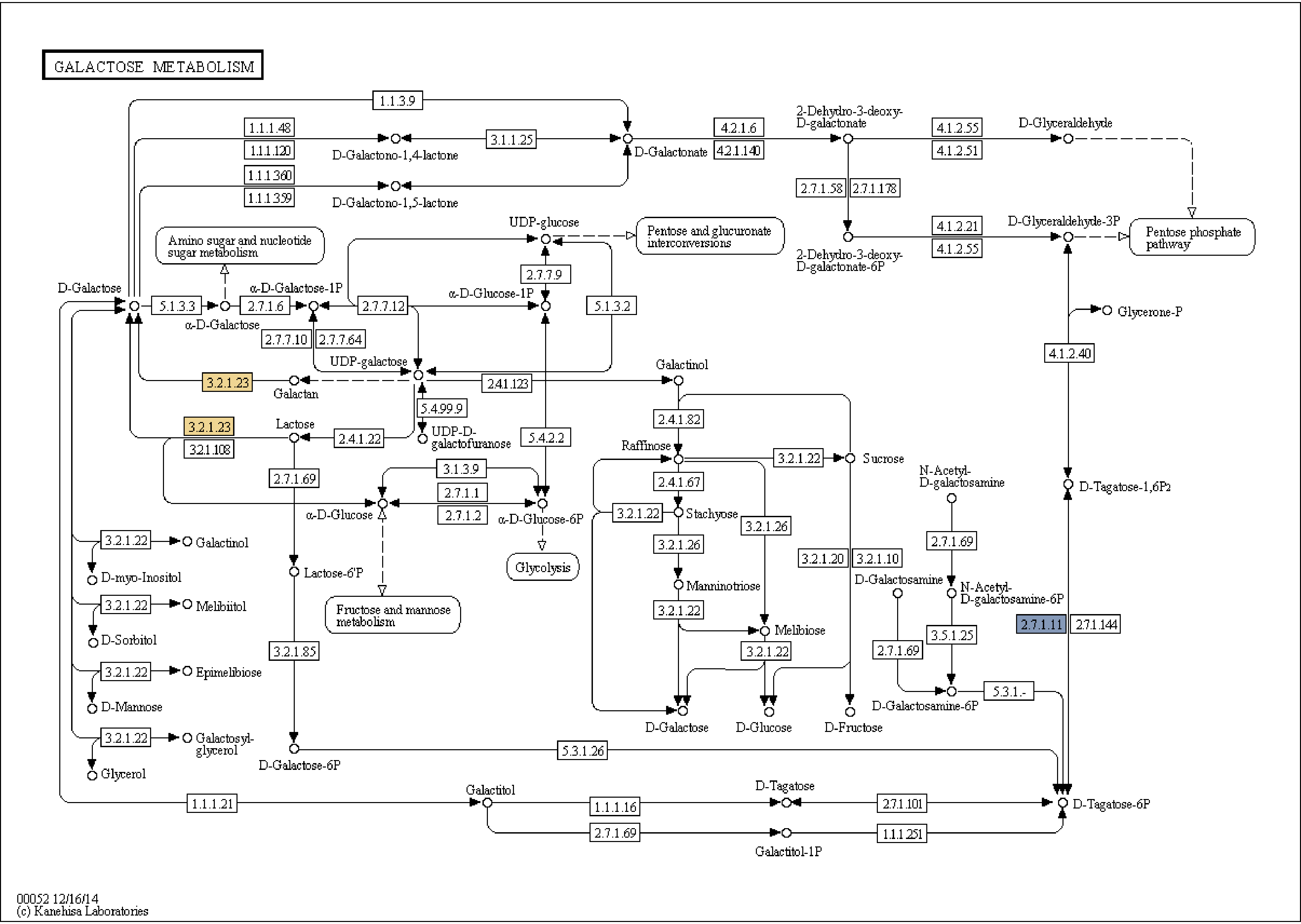

GALACTOSE METABOLISM
1.1.3.9
1.1.1.48
1.1.1.120
3.1.1.25
D-Galactono-1,4-lactone
1.1.1.360
1.1.1.359
D-Galactono-1,5-lactone
D-Galactonate
4.2.1.6
4.2.1.140
2-Dehydro-3-deoxy-D-galactonate
4.1.2.55
4.1.2.51
D-Glyceraldehyde
2.7.1.58
2.7.1.178
2-Dehydro-3-deoxy-D-galactonate-6P
4.1.2.21
4.1.2.55
D-Glyceraldehyde-3P
Pentose phosphate pathway
UDP-glucose
Pentose and glucuronate interconversions
Amino sugar and nucleotide sugar metabolism
D-Galactose
5.1.3.3
α-D-Galactose
α-D-Galactose-1P
2.7.1.6
2.7.7.12
2.7.7.10
2.7.7.64
2.7.7.9
α-D-Glucose-1P
5.1.3.2
UDP-galactose
Galactinol
Glycerone-P
4.1.2.40
3.2.1.23
Galactan
2.4.1.123
5.4.99.9
UDP-D-galactofuranose
3.2.1.23
3.2.1.108
Lactose
2.4.1.22
5.4.2.2
2.4.1.82
Raffinose
3.2.1.22
Sucrose
2.7.1.69
3.1.3.9
2.7.1.1
2.7.1.2
α-D-Glucose
α-D-Glucose-6P
2.4.1.67
3.2.1.22
Stachyose
3.2.1.26
N-Acetyl-D-galactosamine
D-Tagatose-1,6P2
3.2.1.22
Galactinol
D-myo-Inositol
Glycolysis
3.2.1.26
2.7.1.69
3.2.1.20
3.2.1.10
Lactose-6'P
Manninotriose
D-Galactosamine
N-Acetyl-D-galactosamine-6P
3.2.1.22
Melibiitol
D-Sorbitol
Fructose and mannose metabolism
3.2.1.22
Melibiose
2.7.1.11
2.7.1.144
3.2.1.85
3.2.1.22
2.7.1.69
3.5.1.25
3.2.1.22
Epimelibiose
D-Mannose
5.3.1.-
D-Galactosamine-6P
D-Galactose
D-Glucose
D-Fructose
3.2.1.22
Galactosyl-glycerol
Glycerol
D-Galactose-6P
5.3.1.26
Galactitol
D-Tagatose
1.1.1.21
1.1.1.16
2.7.1.101
D-Tagatose-6P
2.7.1.69
1.1.1.251
Galactitol-1P
00052 12/16/14
(c) Kanehisa Laboratories

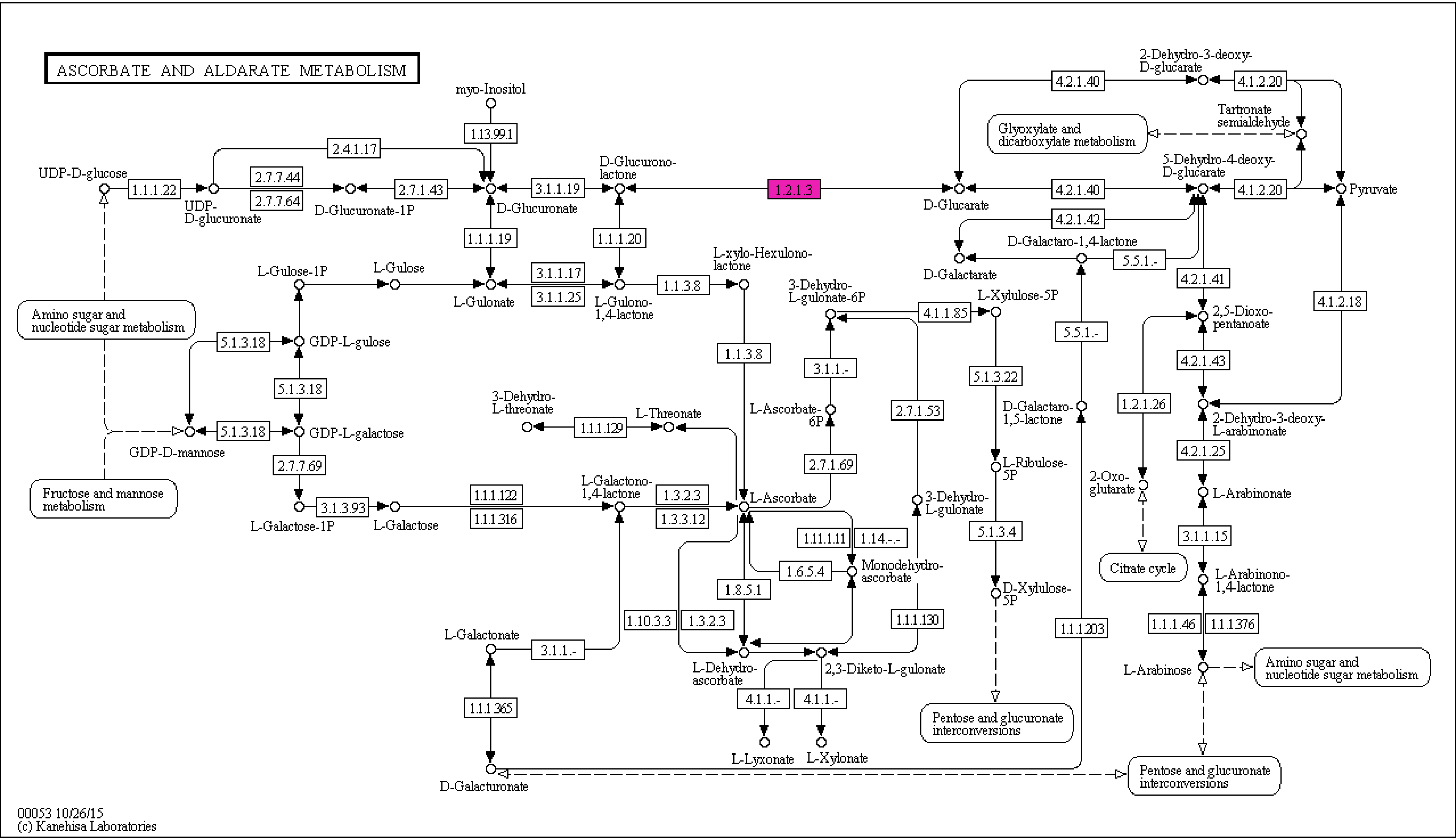
ASCORBATE AND ALDARATE METABOLISM
myo-Inositol
1.13.99.1
2.4.1.17
UDP-D-glucose
1.1.1.22
UDP-D-glucuronate
2.7.7.44
2.7.7.64
D-Glucuronate-1P
2.7.1.43
D-Glucuronate
3.1.1.19
D-Glucurono-lactone
1.2.1.3
D-Glucarate
4.2.1.40
2-Dehydro-3-deoxy-D-glucarate
4.1.2.20
Tartronate semialdehyde
Glyoxylate and dicarboxylate metabolism
5-Dehydro-4-deoxy-D-glucarate
Pyruvate
4.2.1.42
D-Galactaro-1,4-lactone
5.5.1.-
D-Galactarate
1.1.1.19
1.1.1.20
L-Gulose-1P
L-Gulose
3.1.1.17
3.1.1.25
L-Gulonate
L-Gulono-1,4-lactone
1.1.3.8
L-xylo-Hexulono-lactone
3-Dehydro-L-gulonate-6P
4.1.1.85
L-Xylulose-5P
4.2.1.41
2,5-Dioxo-pentanoate
4.1.2.18
Amino sugar and nucleotide sugar metabolism
5.1.3.18
GDP-L-gulose
3.1.1.-
5.1.3.22
4.2.1.43
1.2.1.26
D-Galactaro-1,5-lactone
2-Dehydro-3-deoxy-L-arabinonate
3-Dehydro-L-threonate
L-Threonate
1.1.1.129
L-Ascorbate-6P
2.7.1.53
GDP-D-mannose
GDP-L-galactose
2.7.7.69
2.7.1.69
L-Ribulose-5P
4.2.1.25
2-Oxo-glutarate
L-Arabinonate
Fructose and mannose metabolism
3.1.3.93
L-Galactose-1P
L-Galactose
1.1.1.122
1.1.1.316
L-Galactono-1,4-lactone
1.3.2.3
1.3.3.12
L-Ascorbate
3-Dehydro-L-gulonate
5.1.3.4
3.1.1.15
1.11.1.11
1.14.-.-
Citrate cycle
1.6.5.4
Monodehydro-ascorbate
L-Arabinono-1,4-lactone
1.8.5.1
D-Xylulose-5P
1.10.3.3
1.3.2.3
1.1.1.130
1.1.1.203
1.1.1.46
1.1.1.376
L-Galactonate
3.1.1.-
L-Dehydro-ascorbate
2,3-Diketo-L-gulonate
L-Arabinose
Amino sugar and nucleotide sugar metabolism
4.1.1.-
4.1.1.-
1.1.1.365
Pentose and glucuronate interconversions
L-Lyxonate
L-Xylonate
D-Galacturonate
Pentose and glucuronate interconversions
00053 10/26/15
(c) Kanehisa Laboratories

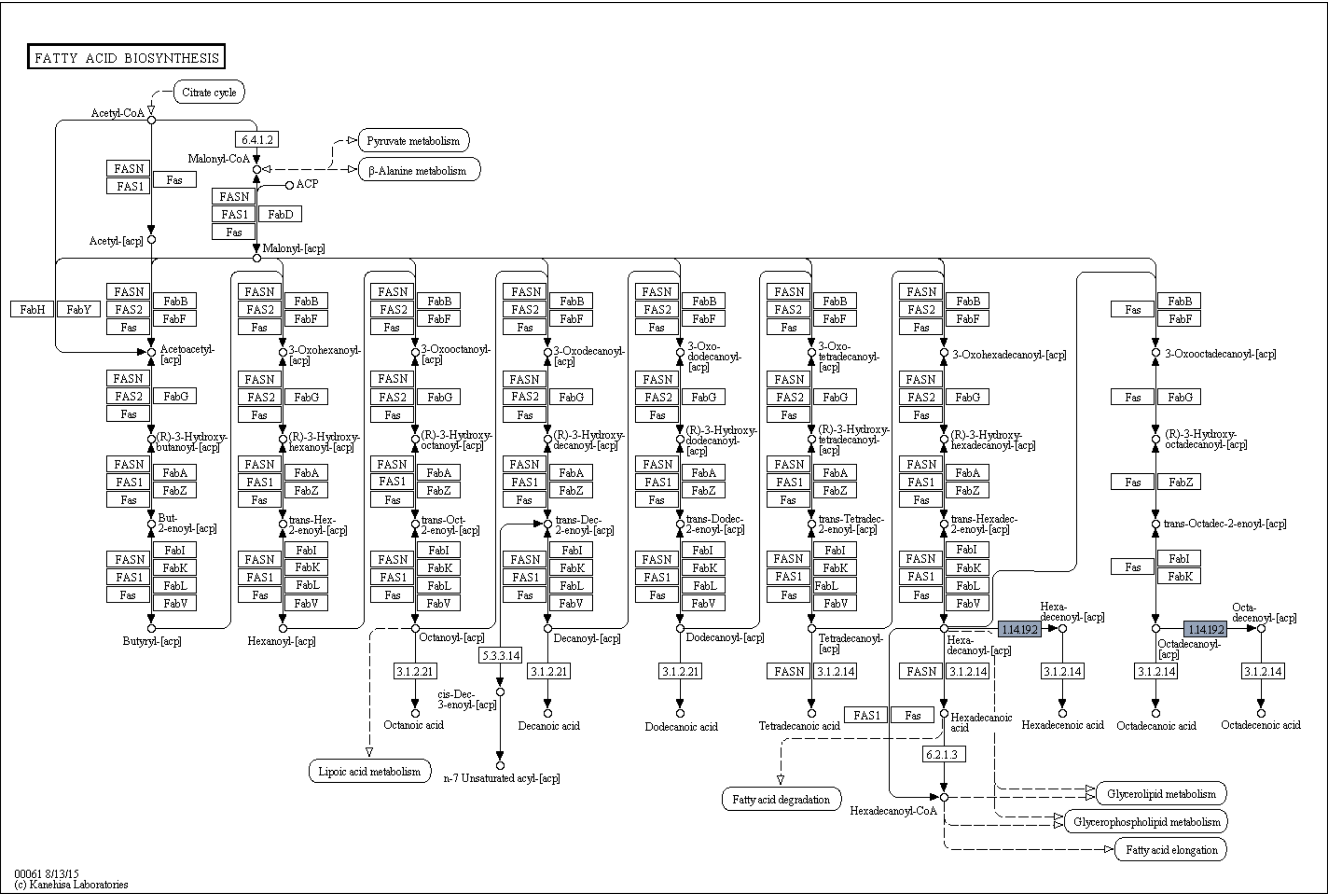
FATTY ACID BIOSYNTHESIS
Citrate cycle
Acetyl-CoA
6.4.1.2
Malonyl-CoA
Pyruvate metabolism
β-Alanine metabolism
ACP
FASN
FAS1
FAS2
Fas
FabD
FabH
FabY
FabB
FabF
FabG
FabA
FabZ
FabI
FabK
FabL
FabV
Acetyl-[acp]
Malonyl-[acp]
Acetoacetyl-[acp]
(R)-3-Hydroxy-butanoyl-[acp]
But-2-enoyl-[acp]
Butyryl-[acp]
3-Oxohexanoyl-[acp]
(R)-3-Hydroxy-hexanoyl-[acp]
trans-Hex-2-enoyl-[acp]
Hexanoyl-[acp]
3-Oxooctanoyl-[acp]
(R)-3-Hydroxy-octanoyl-[acp]
trans-Oct-2-enoyl-[acp]
Octanoyl-[acp]
3-Oxodecanoyl-[acp]
(R)-3-Hydroxy-decanoyl-[acp]
trans-Dec-2-enoyl-[acp]
Decanoyl-[acp]
3-Oxo-dodecanoyl-[acp]
(R)-3-Hydroxy-dodecanoyl-[acp]
trans-Dodec-2-enoyl-[acp]
Dodecanoyl-[acp]
3-Oxo-tetradecanoyl-[acp]
(R)-3-Hydroxy-tetradecanoyl-[acp]
trans-Tetradec-2-enoyl-[acp]
Tetradecanoyl-[acp]
3-Oxohexadecanoyl-[acp]
(R)-3-Hydroxy-hexadecanoyl-[acp]
trans-Hexadec-2-enoyl-[acp]
Hexa-decanoyl-[acp]
3-Oxooctadecanoyl-[acp]
(R)-3-Hydroxy-octadecanoyl-[acp]
trans-Octadec-2-enoyl-[acp]
Octadecanoyl-[acp]
1.14.19.2
Hexa-decenoyl-[acp]
Octa-decenoyl-[acp]
3.1.2.21
3.1.2.14
5.3.3.14
6.2.1.3
cis-Dec-3-enoyl-[acp]
n-7 Unsaturated acyl-[acp]
Octanoic acid
Decanoic acid
Dodecanoic acid
Tetradecanoic acid
Hexadecanoic acid
Hexadecenoic acid
Octadecanoic acid
Octadecenoic acid
Hexadecanoyl-CoA
Lipoic acid metabolism
Fatty acid degradation
Glycerolipid metabolism
Glycerophospholipid metabolism
Fatty acid elongation
00061 8/13/15
(c) Kanehisa Laboratories

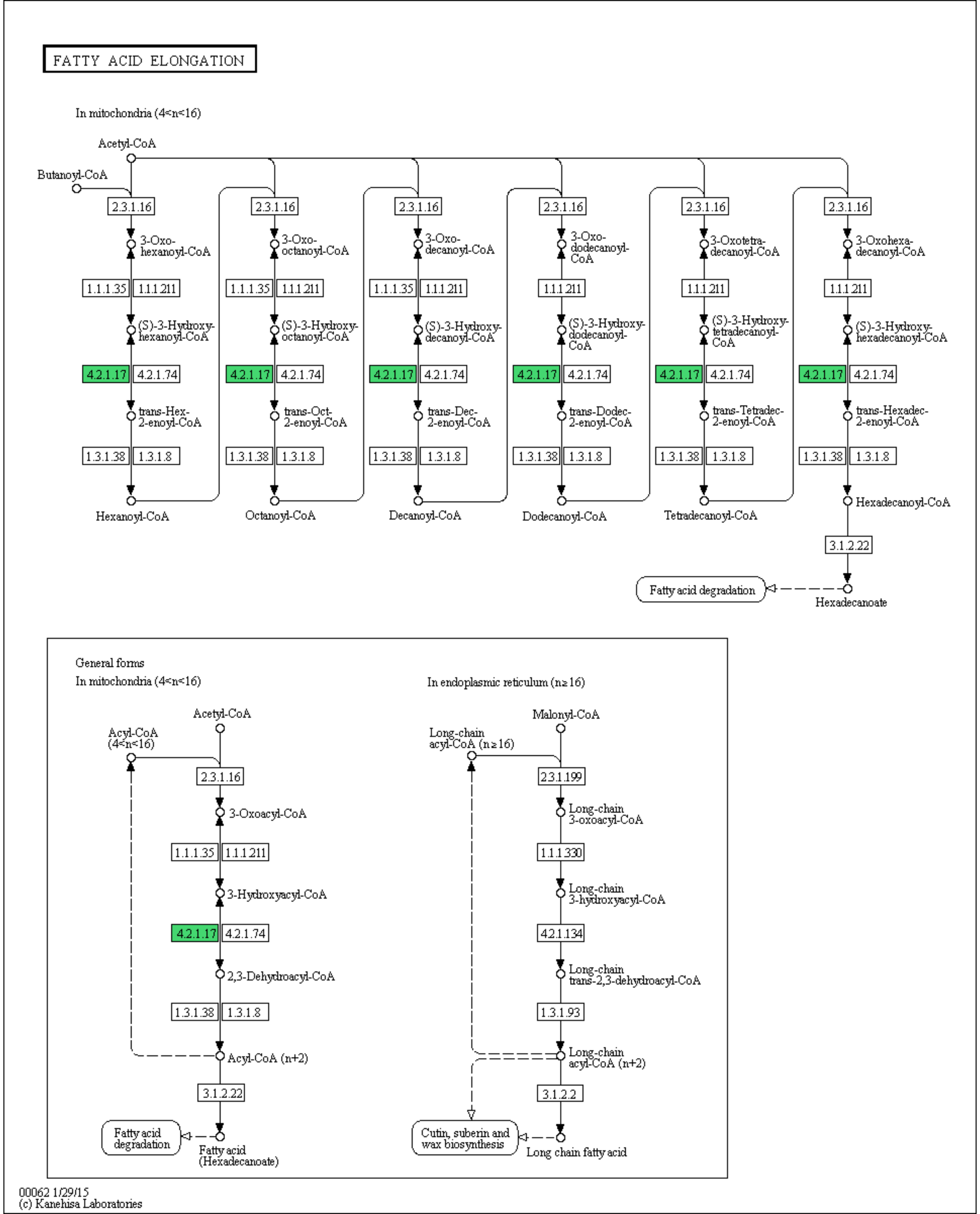
FATTY ACID ELONGATION
In mitochondria (4<n<16)
Acetyl-CoA
Butanoyl-CoA
2.3.1.16
3-Oxo-hexanoyl-CoA
3-Oxo-octanoyl-CoA
3-Oxo-decanoyl-CoA
3-Oxo-dodecanoyl-CoA
3-Oxotetra-decanoyl-CoA
3-Oxohexa-decanoyl-CoA
1.1.1.35
1.1.1.211
(S)-3-Hydroxy-hexanoyl-CoA
(S)-3-Hydroxy-octanoyl-CoA
(S)-3-Hydroxy-decanoyl-CoA
(S)-3-Hydroxy-dodecanoyl-CoA
(S)-3-Hydroxy-tetradecanoyl-CoA
(S)-3-Hydroxy-hexadecanoyl-CoA
4.2.1.17
4.2.1.74
trans-Hex-2-enoyl-CoA
trans-Oct-2-enoyl-CoA
trans-Dec-2-enoyl-CoA
trans-Dodec-2-enoyl-CoA
trans-Tetradec-2-enoyl-CoA
trans-Hexadec-2-enoyl-CoA
1.3.1.38
1.3.1.8
Hexanoyl-CoA
Octanoyl-CoA
Decanoyl-CoA
Dodecanoyl-CoA
Tetradecanoyl-CoA
Hexadecanoyl-CoA
3.1.2.22
Fatty acid degradation
Hexadecanoate
General forms
In mitochondria (4<n<16)
Acetyl-CoA
Acyl-CoA (4<n<16)
2.3.1.16
3-Oxoacyl-CoA
1.1.1.35
1.1.1.211
3-Hydroxyacyl-CoA
4.2.1.17
4.2.1.74
2,3-Dehydroacyl-CoA
1.3.1.38
1.3.1.8
Acyl-CoA (n+2)
3.1.2.22
Fatty acid degradation
Fatty acid (Hexadecanoate)
In endoplasmic reticulum (n≥16)
Malonyl-CoA
Long-chain acyl-CoA (n≥16)
2.3.1.199
Long-chain 3-oxoacyl-CoA
1.1.1.330
Long-chain 3-hydroxyacyl-CoA
4.2.1.134
Long-chain trans-2,3-dehydroacyl-CoA
1.3.1.93
Long-chain acyl-CoA (n+2)
3.1.2.2
Cutin, suberin and wax biosynthesis
Long chain fatty acid
00062 1/29/15
(c) Kanehisa Laboratories

FATTY ACID DEGRADATION

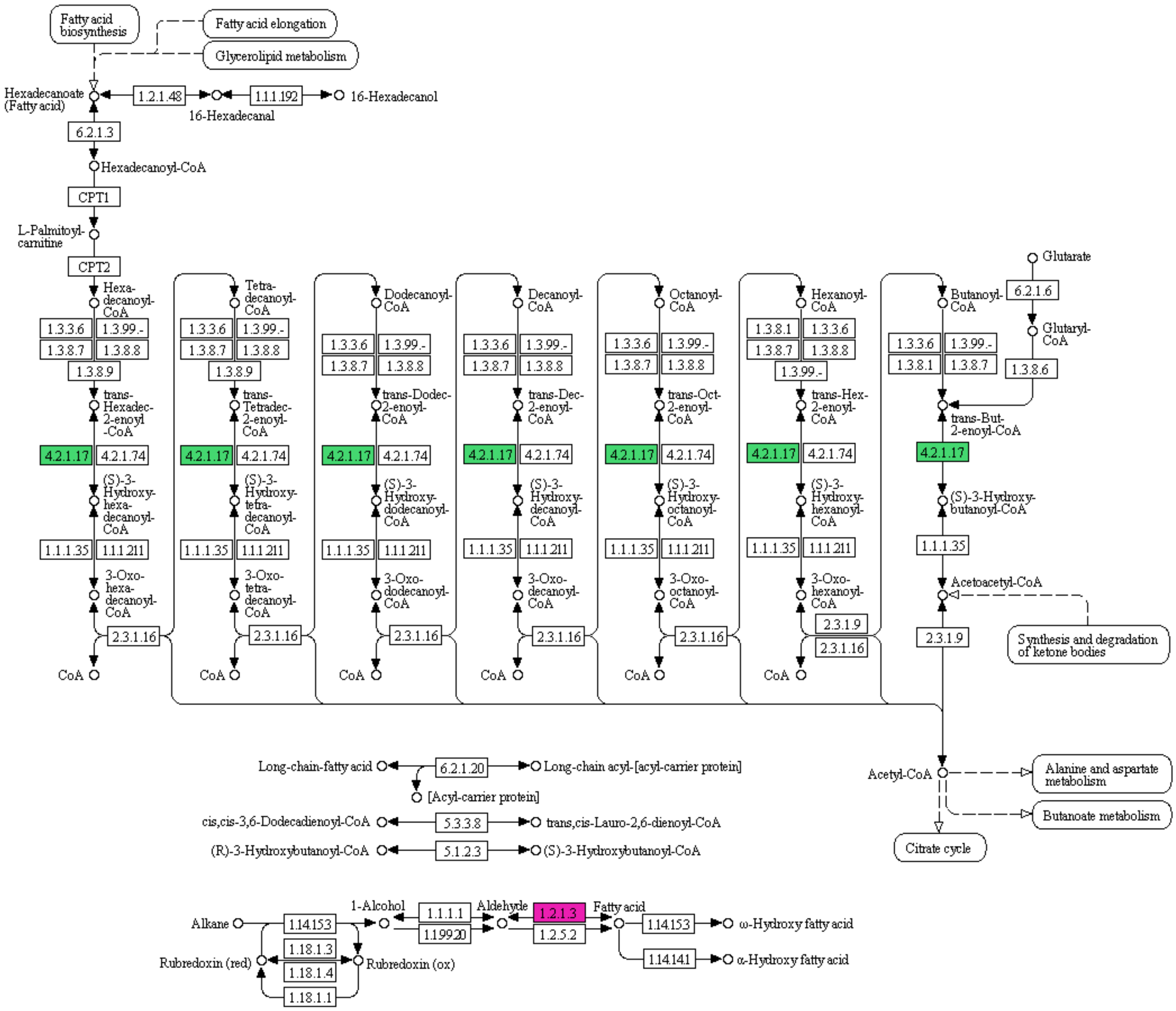

00071 8/10/15
(c) Kanehisa Laboratories

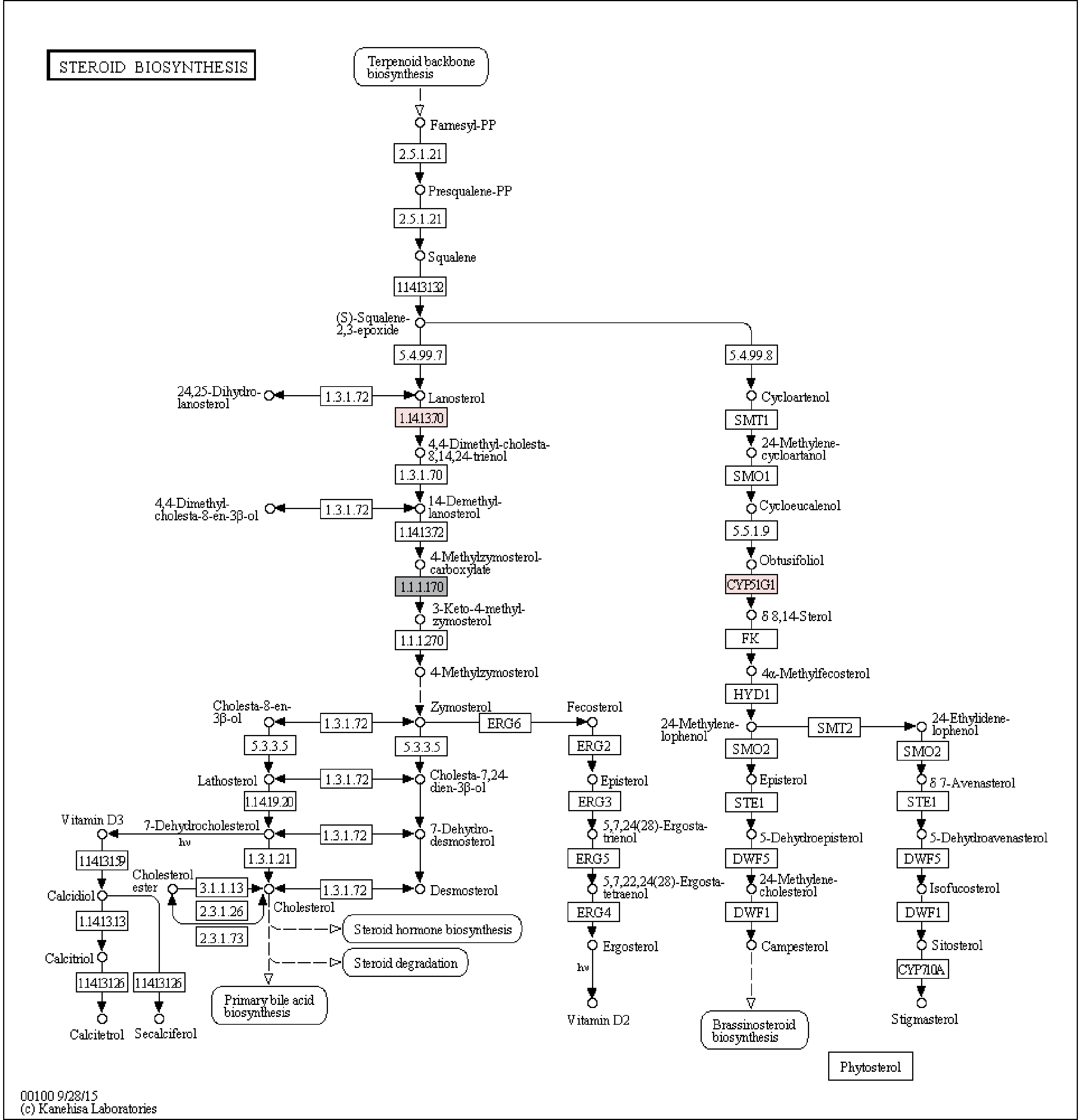

STEROID BIOSYNTHESIS
Terpenoid backbone biosynthesis
Farnesyl-PP
2.5.1.21
Presqualene-PP
2.5.1.21
Squalene
1.14.13.132
(S)-Squalene-2,3-epoxide
5.4.99.7
5.4.99.8
24,25-Dihydro-lanosterol
1.3.1.72
Lanosterol
1.14.13.70
4,4-Dimethyl-cholesta-8,14,24-trienol
1.3.1.70
4,4-Dimethyl-cholesta-8-en-3β-ol
1.3.1.72
14-Demethyl-lanosterol
1.14.13.72
4-Methylzymosterol-carboxylate
1.1.1.170
3-Keto-4-methyl-zymosterol
1.1.1.270
4-Methylzymosterol
Cholesta-8-en-3β-ol
1.3.1.72
Zymosterol
ERG6
Fecosterol
5.3.3.5
5.3.3.5
ERG2
Lathosterol
1.3.1.72
Cholesta-7,24-dien-3β-ol
Episterol
1.14.19.20
ERG3
Vitamin D3
7-Dehydrocholesterol
hν
1.3.1.72
7-Dehydro-desmosterol
5,7,24(28)-Ergosta-trienol
1.14.13.159
1.3.1.21
ERG5
Cholesterol ester
3.1.1.13
1.3.1.72
Desmosterol
5,7,22,24(28)-Ergosta-tetraenol
Calcidiol
2.3.1.26
Cholesterol
ERG4
1.14.13.13
2.3.1.73
Steroid hormone biosynthesis
Calcitriol
Steroid degradation
Ergosterol
hν
1.14.13.126
1.14.13.126
Primary bile acid biosynthesis
Vitamin D2
Calcitetrol
Secalciferol
Cycloartenol
SMT1
24-Methylene-cycloartanol
SMO1
Cycloeucalenol
5.5.1.9
Obtusifoliol
CYP51G1
δ 8,14-Sterol
FK
4α-Methylfecosterol
HYD1
24-Methylene-lophenol
SMT2
24-Ethylidene-lophenol
SMO2
SMO2
Episterol
δ 7-Avenasterol
STE1
STE1
5-Dehydroepisterol
5-Dehydroavenasterol
DWF5
DWF5
24-Methylene-cholesterol
Isofucosterol
DWF1
DWF1
Campesterol
Sitosterol
CYP710A
Brassinosteroid biosynthesis
Stigmasterol
Phytosterol
00100 9/28/15
(c) Kanehisa Laboratories

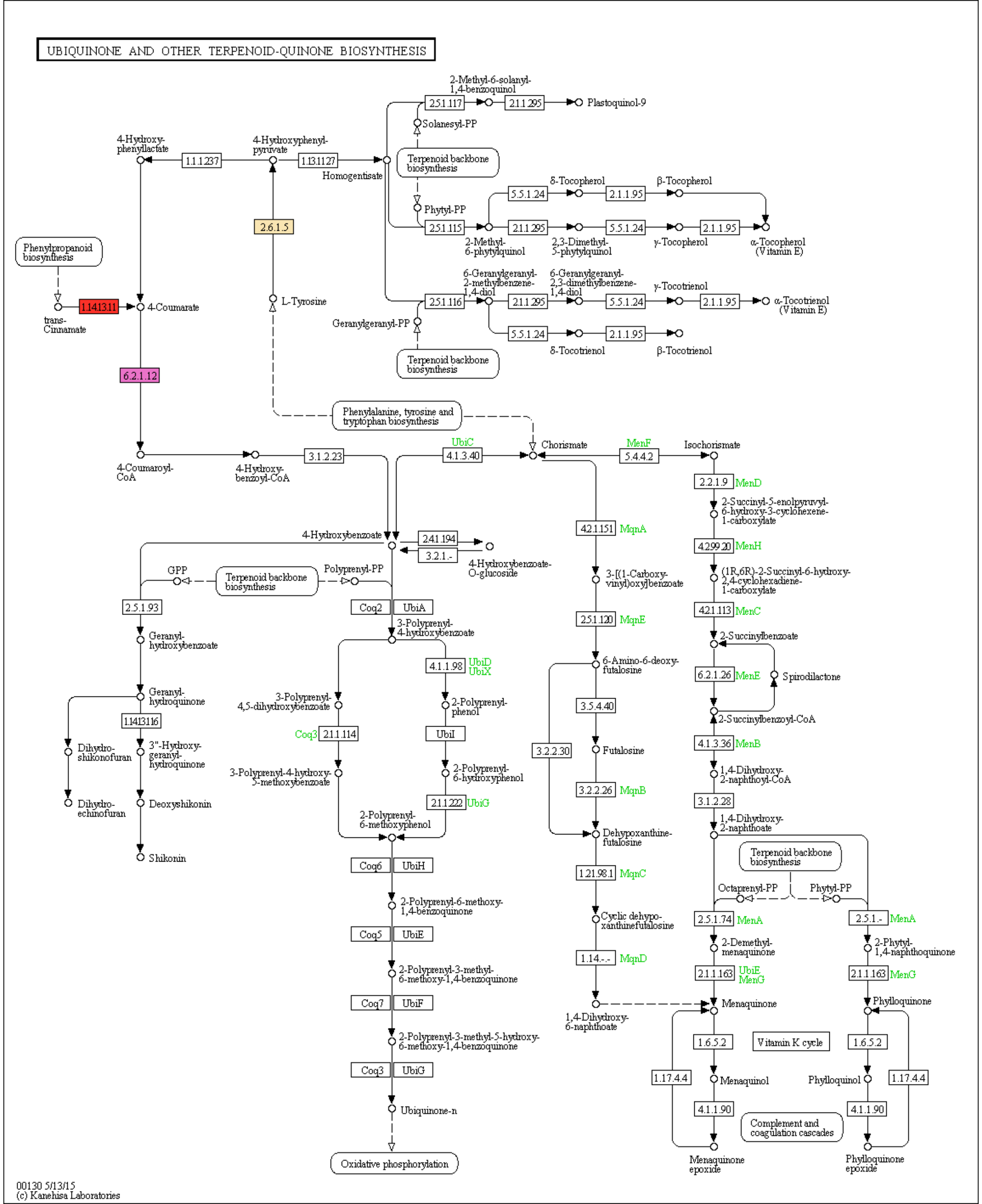
UBIQUINONE AND OTHER TERPENOID-QUINONE BIOSYNTHESIS
2-Methyl-6-solanyl-1,4-benzoquinol
2.5.1.117
2.1.1.295
Plastoquinol-9
Solanesyl-PP
4-Hydroxy-phenyllactate
1.1.1.237
4-Hydroxyphenyl-pyruvate
1.13.11.27
Homogentisate
Terpenoid backbone biosynthesis
δ-Tocopherol
β-Tocopherol
5.5.1.24
2.1.1.95
Phytyl-PP
2.5.1.115
2.1.1.295
5.5.1.24
2.1.1.95
2-Methyl-6-phytylquinol
2,3-Dimethyl-5-phytylquinol
γ-Tocopherol
α-Tocopherol (Vitamin E)
2.6.1.5
Phenylpropanoid biosynthesis
1.14.13.11
trans-Cinnamate
4-Coumarate
L-Tyrosine
6-Geranylgeranyl-2-methylbenzene-1,4-diol
6-Geranylgeranyl-2,3-dimethylbenzene-1,4-diol
γ-Tocotrienol
2.5.1.116
2.1.1.295
5.5.1.24
2.1.1.95
α-Tocotrienol (Vitamin E)
Geranylgeranyl-PP
5.5.1.24
2.1.1.95
δ-Tocotrienol
β-Tocotrienol
Terpenoid backbone biosynthesis
6.2.1.12
Phenylalanine, tyrosine and tryptophan biosynthesis
UbiC
MenF
Chorismate
Isochorismate
4-Coumaroyl-CoA
4-Hydroxy-benzoyl-CoA
3.1.2.23
4.1.3.40
5.4.4.2
2.2.1.9
MenD
2-Succinyl-5-enolpyruvyl-6-hydroxy-3-cyclohexene-1-carboxylate
4.2.1.151
MqnA
4.2.99.20
MenH
4-Hydroxybenzoate
2.4.1.194
3.2.1.-
4-Hydroxybenzoate-O-glucoside
(1R,6R)-2-Succinyl-6-hydroxy-2,4-cyclohexadiene-1-carboxylate
3-[(1-Carboxy-vinyl)oxy]benzoate
GPP
Terpenoid backbone biosynthesis
Polyprenyl-PP
2.5.1.93
Coq2
UbiA
2.5.1.120
MqnE
4.2.1.113
MenC
Geranyl-hydroxybenzoate
3-Polyprenyl-4-hydroxybenzoate
2-Succinylbenzoate
6-Amino-6-deoxy-futalosine
4.1.1.98
UbiD
UbiX
6.2.1.26
MenE
Spirodilactone
Geranyl-hydroquinone
3-Polyprenyl-4,5-dihydroxybenzoate
2-Polyprenyl-phenol
3.5.4.40
2-Succinylbenzoyl-CoA
1.14.13.16
Coq3
2.1.1.114
UbiI
Futalosine
3.2.2.30
4.1.3.36
MenB
Dihydro-shikonofuran
3''-Hydroxy-geranyl-hydroquinone
3-Polyprenyl-4-hydroxy-5-methoxybenzoate
2-Polyprenyl-6-hydroxyphenol
1,4-Dihydroxy-2-naphthoyl-CoA
3.2.2.26
MqnB
3.1.2.28
Dihydro-echinofuran
Deoxyshikonin
2.1.1.222
UbiG
2-Polyprenyl-6-methoxyphenol
1,4-Dihydroxy-2-naphthoate
Dehypoxanthine-futalosine
Terpenoid backbone biosynthesis
Shikonin
Coq6
UbiH
1.21.98.1
MqnC
Octaprenyl-PP
Phytyl-PP
2-Polyprenyl-6-methoxy-1,4-benzoquinone
Cyclic dehypo-xanthinefutalosine
2.5.1.74
MenA
2.5.1.-
MenA
Coq5
UbiE
2-Demethyl-menaquinone
2-Phytyl-1,4-naphthoquinone
2-Polyprenyl-3-methyl-6-methoxy-1,4-benzoquinone
1.14.-.-
MqnD
2.1.1.163
UbiE
MenG
2.1.1.163
MenG
Coq7
UbiF
Menaquinone
Phylloquinone
1,4-Dihydroxy-6-naphthoate
2-Polyprenyl-3-methyl-5-hydroxy-6-methoxy-1,4-benzoquinone
1.6.5.2
Vitamin K cycle
1.6.5.2
Coq3
UbiG
1.17.4.4
Menaquinol
Phylloquinol
1.17.4.4
4.1.1.90
4.1.1.90
Ubiquinone-n
Complement and coagulation cascades
Menaquinone epoxide
Phylloquinone epoxide
Oxidative phosphorylation
00130 5/13/15
(c) Kanehisa Laboratories

STEROID HORMONE BIOSYNTHESIS

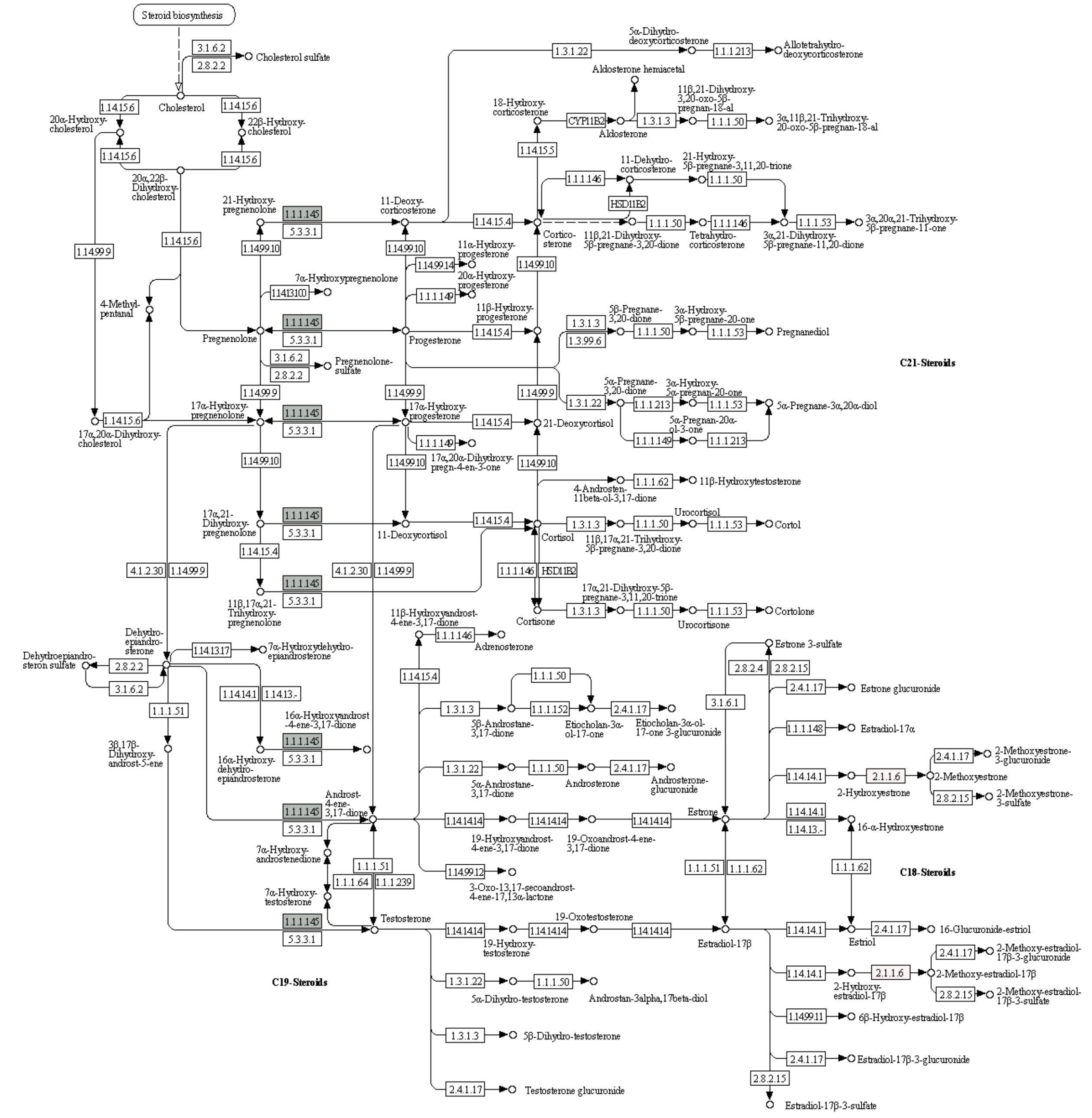

00140 12/18/13
(c) Kanehisa Laboratories

OXIDATIVE PHOSPHORYLATION

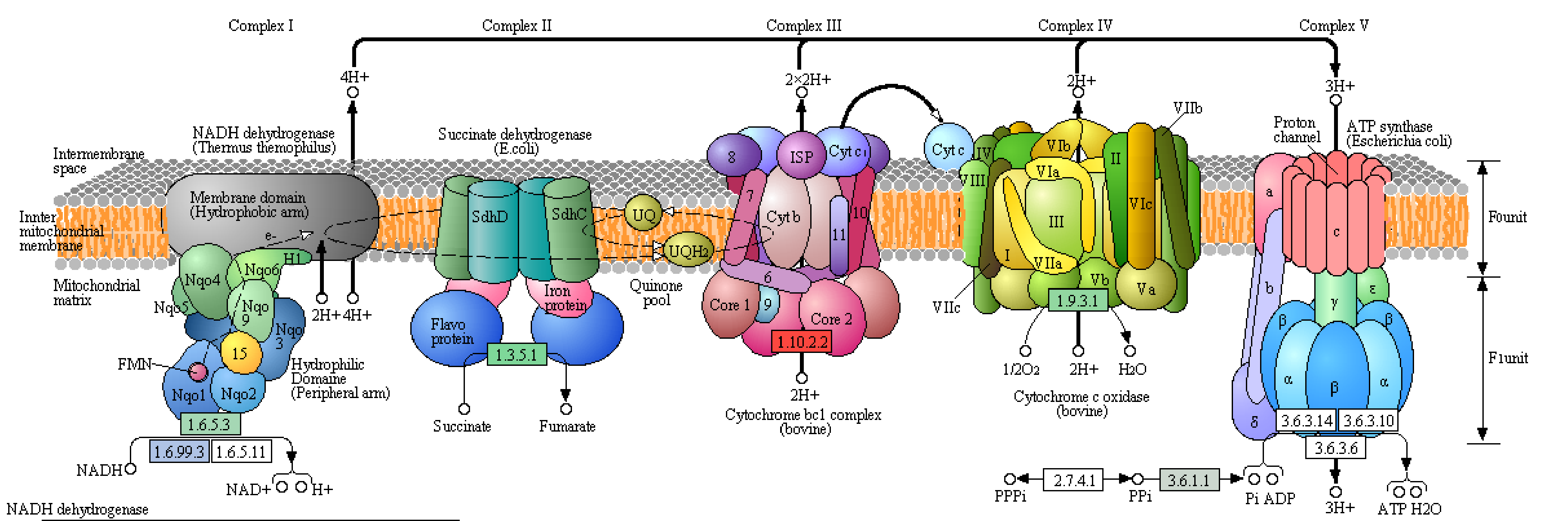

NADH dehydrogenase

E	ND1	ND2	ND3	ND4	ND4L	ND5	ND6										
E	Ndufs1	Ndufs2	Ndufs3	Ndufs4	Ndufs5	Ndufs6	Ndufs7	Ndufs8	Ndufv1	Ndufv2	Ndufv3						
B/A	NuoA	NuoB	NuoC	NuoD	NuoE	NuoF	NuoG	NuoH	NuoI	NuoJ	NuoK	NuoL	NuoM	NuoN			
B/A	NdhC	NdhK	NdhJ	NdhH	NdhA	NdhI	NdhG	NdhE	NdhF	NdhD	NdhB	NdhL	NdhM	NdhN	HoxE	HoxF	HoxU
E	Ndufa1	Ndufa2	Ndufa3	Ndufa4	Ndufa5	Ndufa6	Ndufa7	Ndufa8	Ndufa9	Ndufa10	Ndufab1	Ndufa11	Ndufa12	Ndufa13			
E	Ndufb1	Ndufb2	Ndufb3	Ndufb4	Ndufb5	Ndufb6	Ndufb7	Ndufb8	Ndufb9	Ndufb10	Ndufb11	Ndufc1	Ndufc2				

Succinate dehydrogenase / Fumarate reductase

E	SDHC	SDHD	SDHA	SDHB		
B/A	SdhC	SdhD	SdhA	SdhB		
			FrdA	FrdB	FrdC	FrdD

Cytochrome c reductase

E/B/A	ISP	Cytb	Cyt1							
E				COR1	QCR2	QCR6	QCR7	QCR8	QCR9	QCR10

Cytochrome c oxidase

E	COX10		COX3	COX1	COX2	COX4	COX5A	COX5B	COX6A	COX6B	COX6C	COX7A	COX7B	COX7C	COX8	COX17
E/B/A															COX11, COX15	
B/A	CyoE	CyoD	CyoC	CyoB	CyoA											
		CoxD	CoxC	CoxA	CoxB											
		QoxD	QoxC	QoxB	QoxA											

Cytochrome c oxidase , cbb3-type

B	I	II	IV	III

Cytochrome bd complex

B/A	CydA	CydB

F-type ATPase (Bacteria)

alpha	beta	gamma	delta	epsilon
a	b	c		

F-type ATPase (Eukaryotes)

alpha	beta	gamma	delta	epsilon	
OSCP	a	b	c	d	e
f	g	f6/h	j	k	8

V/A-type ATPase (Bacteria, Archaeas)

A	B	C	D	E	F	G/H
I	K					

V-type ATPase (Eukaryotes)

A	B	C	D	E	F	G	H
a	c	d	e	S1			

00190 6/16/15

(c) Kanehisa Laboratories

PHOTOSYNTHESIS

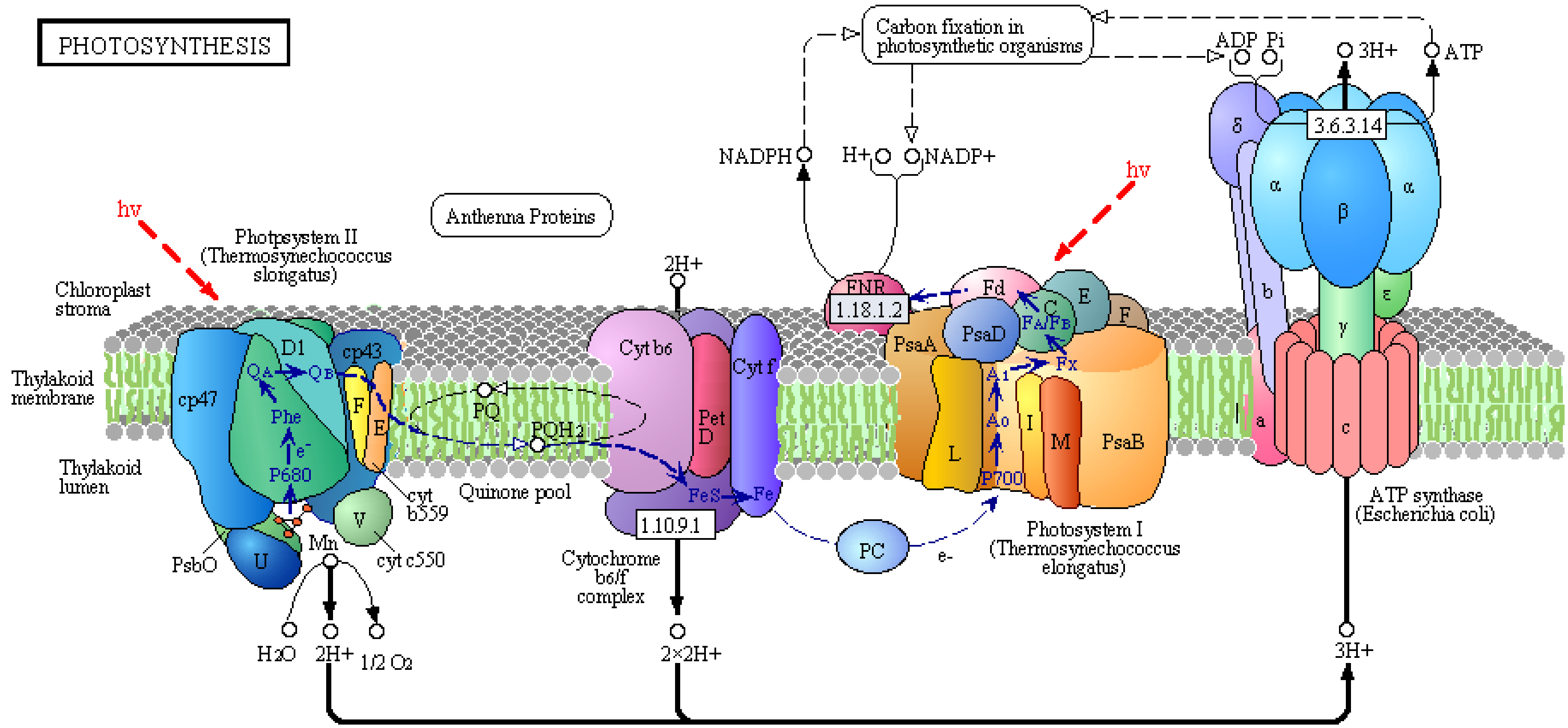

Photosystem II

D1	D2	cp43	cp47	cyt b559	
PsbA	PsbD	PsbC	PsbB	PsbE	PsbF

						MSP	OEC
PsbL	PsbJ	PsbK	PsbM	PsbH	PsbI	PsbO	PsbP
PsbQ	PsbR	PsbS	PsbT	PsbU	PsbV	PsbW	PsbX
PsbY	PsbZ	Psb27	Psb28	Psb28-2			

Photosystem I

PsaA	PsaB	PsaC	PsaD	PsaE	PsaF	PsaG	PsaH
PsaI	PsaJ	PsaK	PsaL	PsaM	PsaN	PsaO	PsaX

Cytochrome b6/f complex

PetB	PetD	PetA	PetC	PetL	PetM	PetN	PetG

Photosynthethic electron transport

PC	Fd	FNR	cyt c6
PetE	PetF	PetH	PetJ

F-type ATPase

beta	alpha	gamma	delta	epsilon	c	a	b

00195 1/15/14

(c) Kanehisa Laboratories

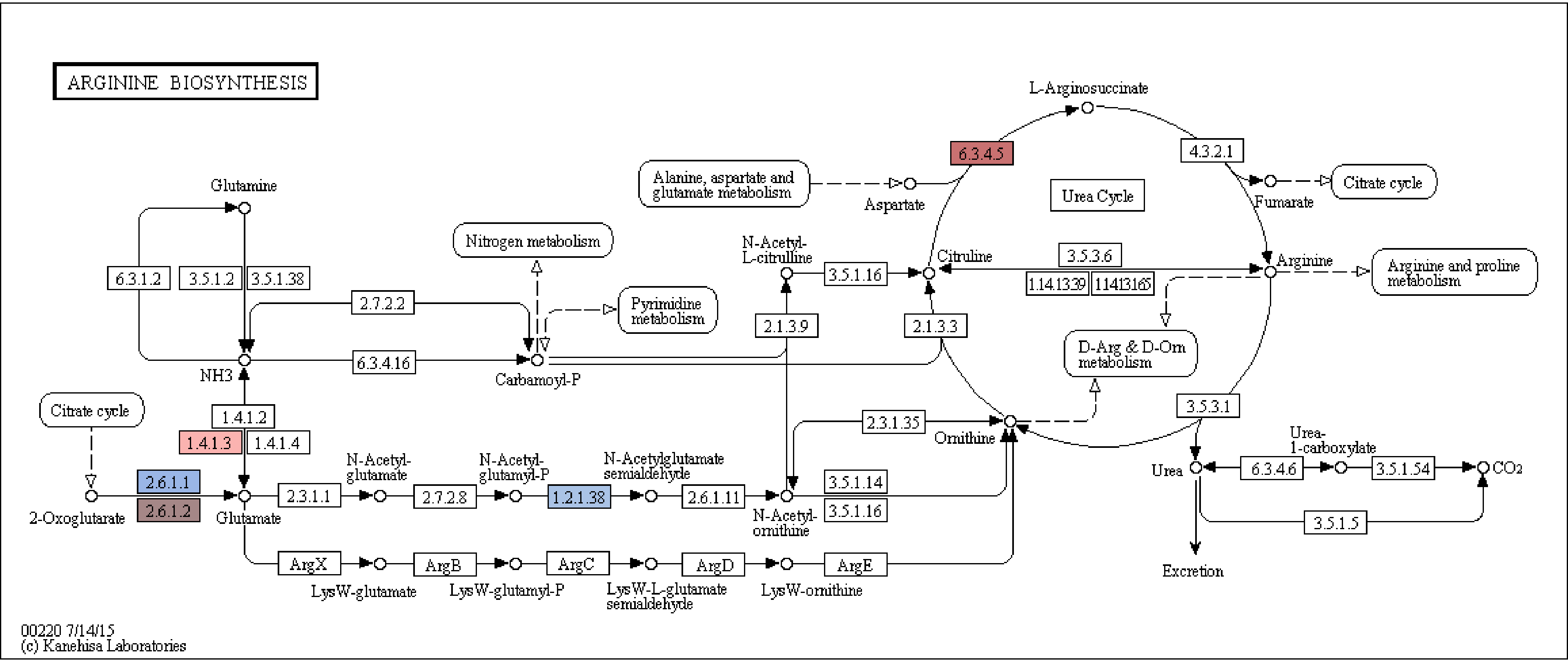

ARGININE BIOSYNTHESIS
L-Arginosuccinate
6.3.4.5
4.3.2.1
Alanine, aspartate and glutamate metabolism
Aspartate
Urea Cycle
Citrate cycle
Fumarate
Glutamine
Nitrogen metabolism
N-Acetyl-L-citrulline
Citruline
3.5.3.6
Arginine
Arginine and proline metabolism
6.3.1.2
3.5.1.2
3.5.1.38
3.5.1.16
1.14.13.39
1.14.13.165
2.7.2.2
Pyrimidine metabolism
2.1.3.9
2.1.3.3
D-Arg & D-Orn metabolism
NH3
6.3.4.16
Carbamoyl-P
Citrate cycle
1.4.1.2
3.5.3.1
2.3.1.35
1.4.1.3
1.4.1.4
Ornithine
Urea-1-carboxylate
N-Acetyl-glutamate
N-Acetyl-glutamyl-P
N-Acetylglutamate semialdehyde
2.6.1.1
3.5.1.14
Urea
6.3.4.6
3.5.1.54
CO2
2.3.1.1
2.7.2.8
1.2.1.38
2.6.1.11
2-Oxoglutarate
2.6.1.2
Glutamate
N-Acetyl-ornithine
3.5.1.16
3.5.1.5
ArgX
ArgB
ArgC
ArgD
ArgE
Excretion
LysW-glutamate
LysW-glutamyl-P
LysW-L-glutamate semialdehyde
LysW-ornithine
00220 7/14/15
(c) Kanehisa Laboratories

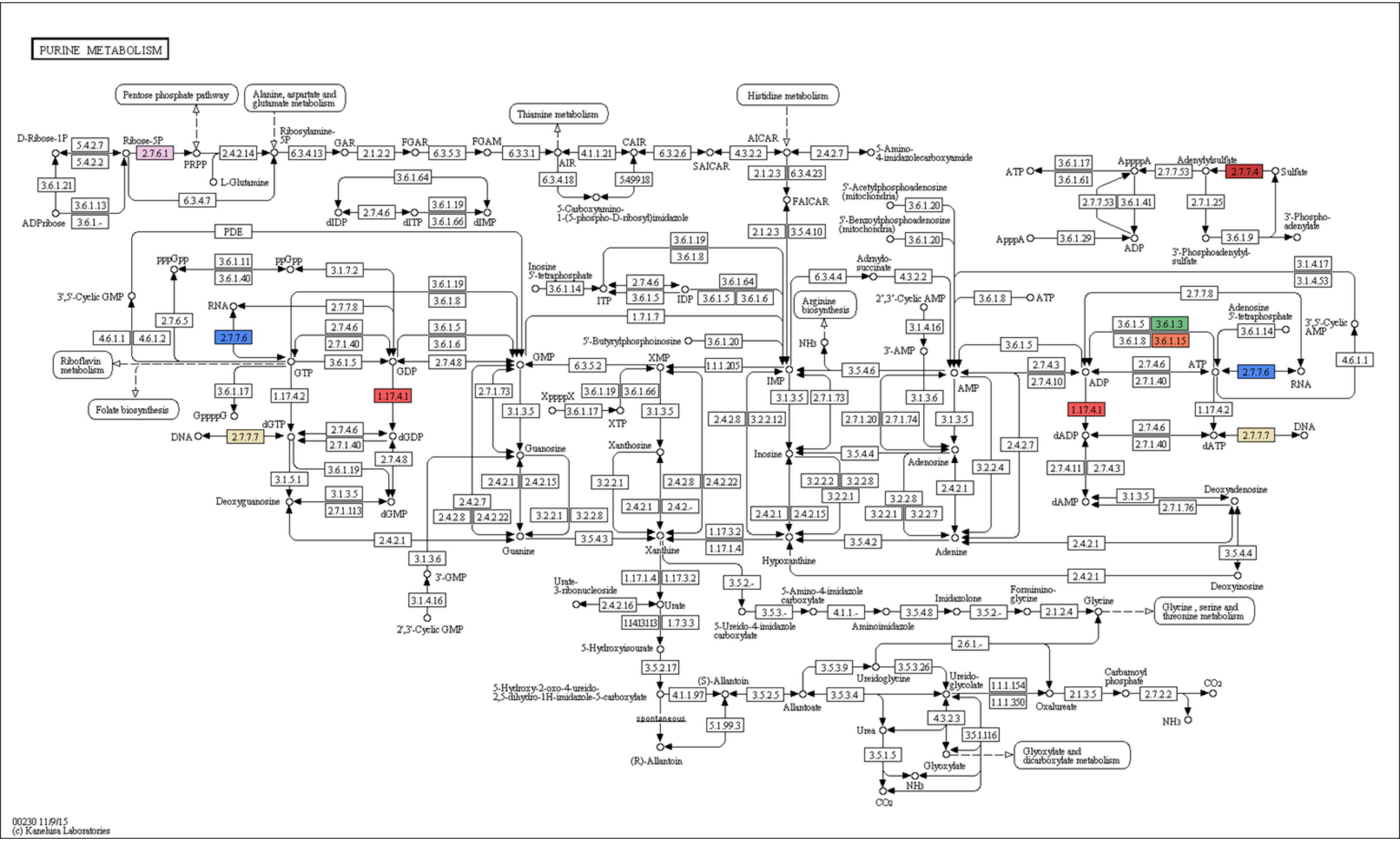
PURINE METABOLISM
Pentose phosphate pathway
Alanine, aspartate and glutamate metabolism
Thiamine metabolism
Histidine metabolism
D-Ribose-1P
Ribose-5P
PRPP
L-Glutamine
Ribosylamine-5P
GAR
FGAR
FGAM
AIR
CAIR
SAICAR
AICAR
FAICAR
5-Amino-4-imidazolecarboxyamide
ADPribose
5-Carboxyamino-1-(5-phospho-D-ribosyl)imidazole
dIDP
dITP
dIMP
PDE
pppGpp
ppGpp
3',5'-Cyclic GMP
RNA
Riboflavin metabolism
Folate biosynthesis
GTP
GDP
GMP
GppppG
dGTP
dGDP
dGMP
DNA
Deoxyguanosine
Guanosine
Guanine
3'-GMP
2',3'-Cyclic GMP
Inosine 5'-tetraphosphate
ITP
IDP
XppppX
XTP
XMP
Xanthosine
Xanthine
5'-Butyrylphosphoinosine
IMP
Inosine
Hypoxanthine
Adenylo-succinate
Arginine biosynthesis
NH3
2',3'-Cyclic AMP
3'-AMP
AMP
Adenosine
Adenine
5'-Acetylphosphoadenosine (mitochondria)
5'-Benzoylphosphoadenosine (mitochondria)
ApppA
ApppA
Adenylylsulfate
Sulfate
3'-Phospho-adenylate
3'-Phosphoadenylyl-sulfate
ATP
ADP
RNA
Adenosine 5'-tetraphosphate
3',5'-Cyclic AMP
dADP
dATP
dAMP
Deoxyadenosine
Deoxyinosine
Urate-3-ribonucleoside
Urate
5-Hydroxyisourate
5-Hydroxy-2-oxo-4-ureido-2,5-dihydro-1H-imidazole-5-carboxylate
(S)-Allantoin
(R)-Allantoin
spontaneous
Allantoate
5-Amino-4-imidazole carboxylate
5-Ureido-4-imidazole carboxylate
Aminoimidazole
Imidazolone
Formimino-glycine
Glycine
Glycine, serine and threonine metabolism
Ureidoglycine
Ureido-glycolate
Oxalureate
Carbamoyl phosphate
CO2
Urea
Glyoxylate
Glyoxylate and dicarboxylate metabolism
00230 11/9/15
(c) Kanehisa Laboratories

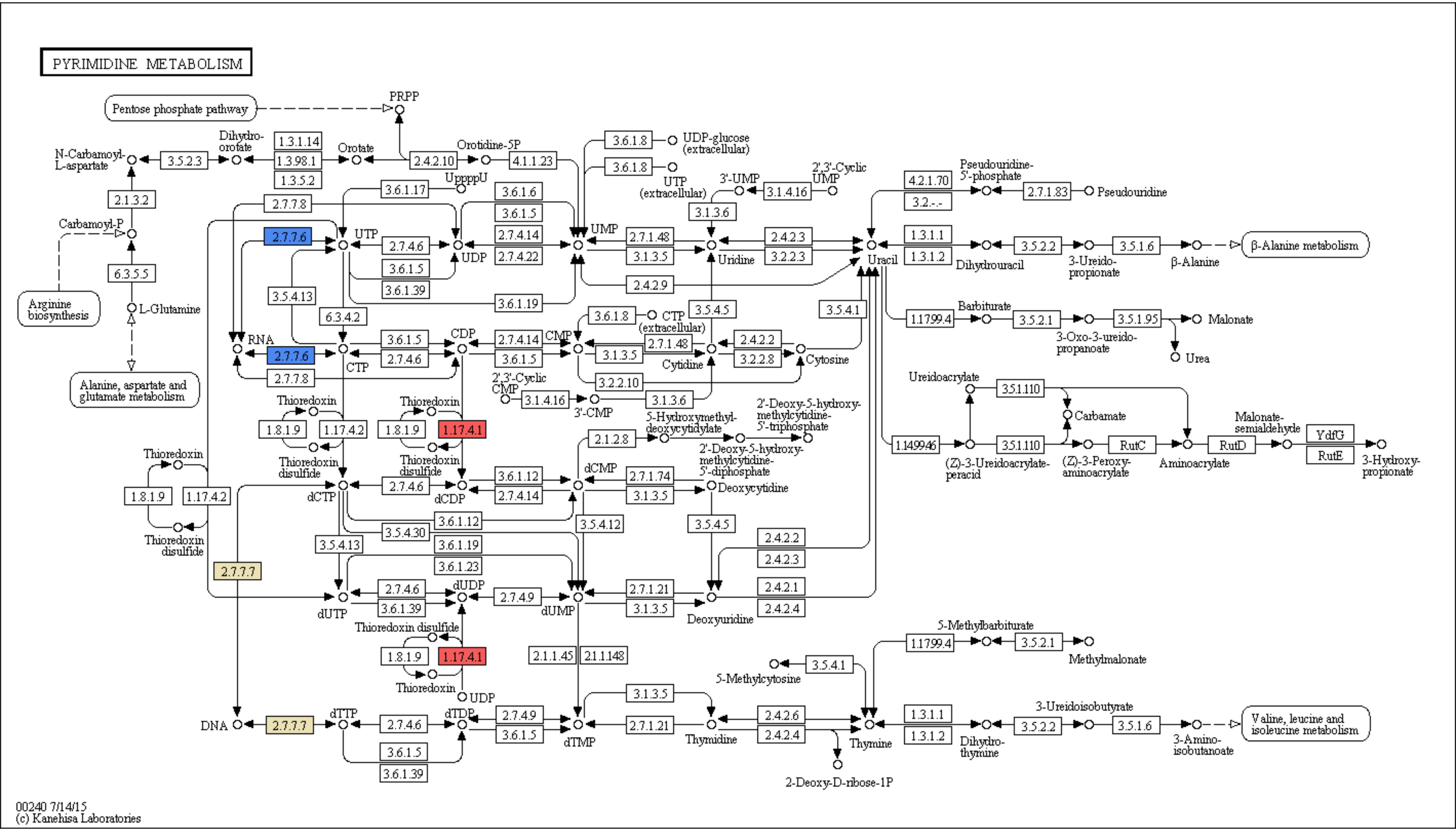
PYRIMIDINE METABOLISM
Pentose phosphate pathway
PRPP
N-Carbamoyl-L-aspartate
3.5.2.3
Dihydro-orotate
1.3.1.14
1.3.98.1
1.3.5.2
Orotate
2.4.2.10
Orotidine-5P
4.1.1.23
2.1.3.2
Carbamoyl-P
6.3.5.5
L-Glutamine
Arginine biosynthesis
Alanine, aspartate and glutamate metabolism
3.6.1.17
UppppU
2.7.7.8
2.7.7.6
UTP
2.7.4.6
UDP
3.6.1.5
3.6.1.39
3.6.1.6
2.7.4.14
2.7.4.22
3.6.1.19
3.5.4.13
6.3.4.2
RNA
CTP
CDP
CMP
3.6.1.8
UDP-glucose (extracellular)
UTP (extracellular)
UMP
2.7.1.48
3.1.3.5
3'-UMP
3.1.4.16
2',3'-Cyclic UMP
3.1.3.6
Uridine
2.4.2.3
3.2.2.3
2.4.2.9
Uracil
CTP (extracellular)
3.5.4.5
Cytidine
2.4.2.2
3.2.2.8
Cytosine
3.2.2.10
2',3'-Cyclic CMP
3'-CMP
3.5.4.1
Thioredoxin
Thioredoxin disulfide
1.8.1.9
1.17.4.2
1.17.4.1
dCTP
dCDP
3.6.1.12
dCMP
2.7.1.74
Deoxycytidine
2.1.2.8
5-Hydroxymethyl-deoxycytidylate
2'-Deoxy-5-hydroxy-methylcytidine-5'-triphosphate
2'-Deoxy-5-hydroxy-methylcytidine-5'-diphosphate
3.5.4.30
3.5.4.12
3.6.1.23
2.7.7.7
dUTP
dUDP
2.7.4.9
dUMP
2.7.1.21
Deoxyuridine
2.4.2.1
2.4.2.4
Thioredoxin disulfide
UDP
2.1.1.45
2.1.1.148
DNA
dTTP
dTDP
dTMP
Thymidine
2.4.2.6
2-Deoxy-D-ribose-1P
Thymine
5-Methylcytosine
5-Methylbarbiturate
1.17.99.4
3.5.2.1
Methylmalonate
1.3.1.1
1.3.1.2
Dihydro-thymine
3-Ureidoisobutyrate
3.5.2.2
3.5.1.6
3-Amino-isobutanoate
Valine, leucine and isoleucine metabolism
4.2.1.70
3.2.-.-
Pseudouridine-5'-phosphate
2.7.1.83
Pseudouridine
Dihydrouracil
3-Ureido-propionate
β-Alanine
β-Alanine metabolism
Barbiturate
3-Oxo-3-ureido-propanoate
3.5.1.95
Malonate
Urea
Ureidoacrylate
3.5.1.110
Carbamate
1.14.99.46
(Z)-3-Ureidoacrylate-peracid
(Z)-3-Peroxy-aminoacrylate
RutC
Aminoacrylate
Malonate-semialdehyde
RutD
YdfG
RutE
3-Hydroxy-propionate
00240 7/14/15
(c) Kanehisa Laboratories

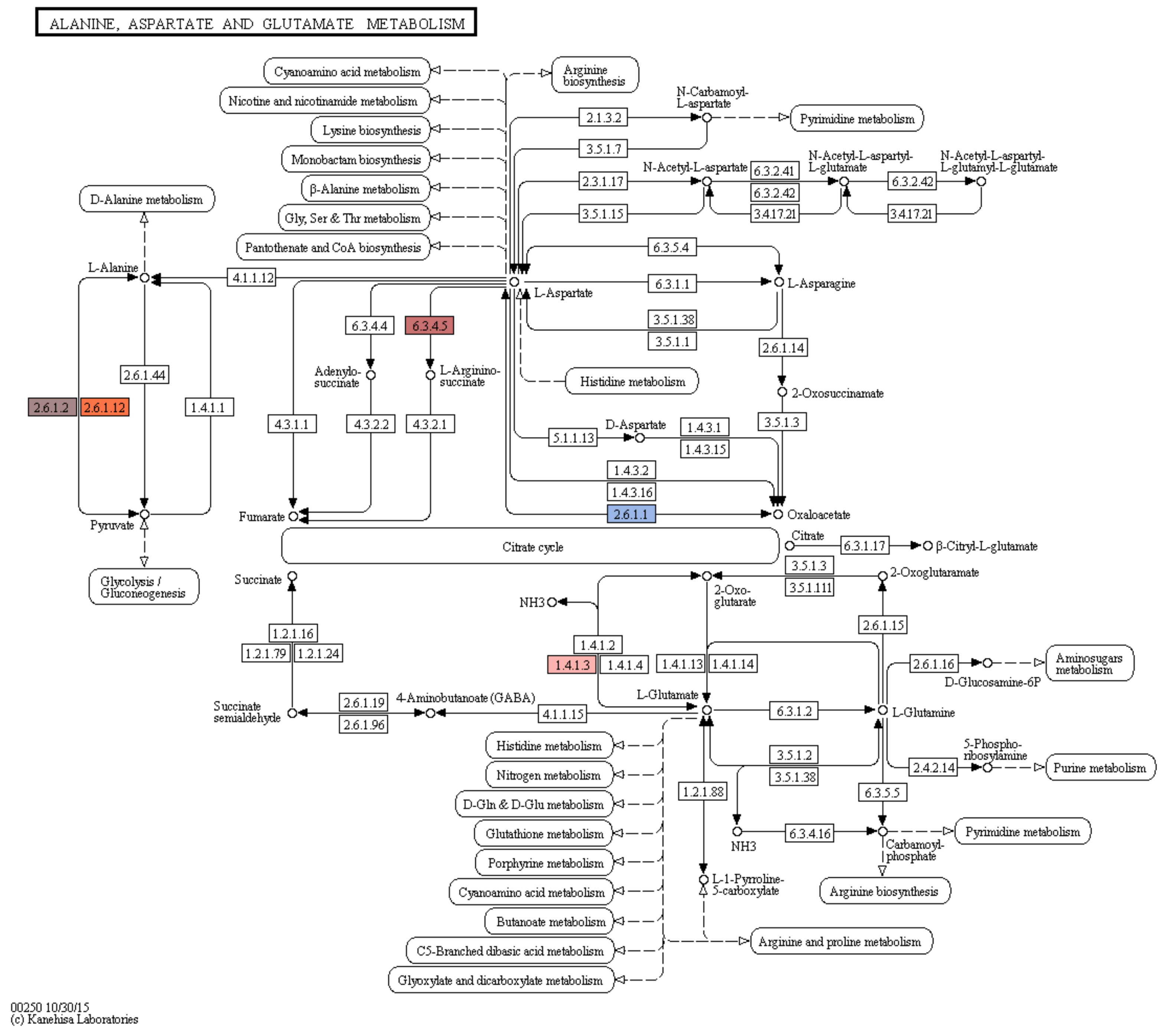
ALANINE, ASPARTATE AND GLUTAMATE METABOLISM
Cyanoamino acid metabolism
Nicotine and nicotinamide metabolism
Lysine biosynthesis
Monobactam biosynthesis
β-Alanine metabolism
Gly, Ser & Thr metabolism
Pantothenate and CoA biosynthesis
Arginine biosynthesis
N-Carbamoyl-L-aspartate
Pyrimidine metabolism
2.1.3.2
3.5.1.7
2.3.1.17
3.5.1.15
N-Acetyl-L-aspartate
6.3.2.41
6.3.2.42
3.4.17.21
N-Acetyl-L-aspartyl-L-glutamate
N-Acetyl-L-aspartyl-L-glutamyl-L-glutamate
D-Alanine metabolism
L-Alanine
4.1.1.12
L-Aspartate
6.3.5.4
6.3.1.1
L-Asparagine
3.5.1.38
3.5.1.1
2.6.1.14
2-Oxosuccinamate
3.5.1.3
6.3.4.4
6.3.4.5
Adenylo-succinate
L-Arginino-succinate
Histidine metabolism
2.6.1.44
2.6.1.2
2.6.1.12
1.4.1.1
4.3.1.1
4.3.2.2
4.3.2.1
D-Aspartate
5.1.1.13
1.4.3.1
1.4.3.15
1.4.3.2
1.4.3.16
2.6.1.1
Oxaloacetate
Fumarate
Pyruvate
Glycolysis / Gluconeogenesis
Citrate cycle
Citrate
6.3.1.17
β-Citryl-L-glutamate
Succinate
3.5.1.3
3.5.1.111
2-Oxoglutaramate
NH3
2-Oxo-glutarate
2.6.1.15
1.2.1.16
1.2.1.79
1.2.1.24
1.4.1.2
1.4.1.3
1.4.1.4
1.4.1.13
1.4.1.14
2.6.1.16
D-Glucosamine-6P
Aminosugars metabolism
Succinate semialdehyde
2.6.1.19
2.6.1.96
4-Aminobutanoate (GABA)
4.1.1.15
L-Glutamate
6.3.1.2
L-Glutamine
5-Phospho-ribosylamine
2.4.2.14
Purine metabolism
3.5.1.2
3.5.1.38
6.3.5.5
1.2.1.88
NH3
6.3.4.16
Carbamoyl-phosphate
Pyrimidine metabolism
Histidine metabolism
Nitrogen metabolism
D-Gln & D-Glu metabolism
Glutathione metabolism
Porphyrine metabolism
Cyanoamino acid metabolism
Butanoate metabolism
C5-Branched dibasic acid metabolism
Glyoxylate and dicarboxylate metabolism
L-1-Pyrroline-5-carboxylate
Arginine biosynthesis
Arginine and proline metabolism
00250 10/30/15
(c) Kanehisa Laboratories

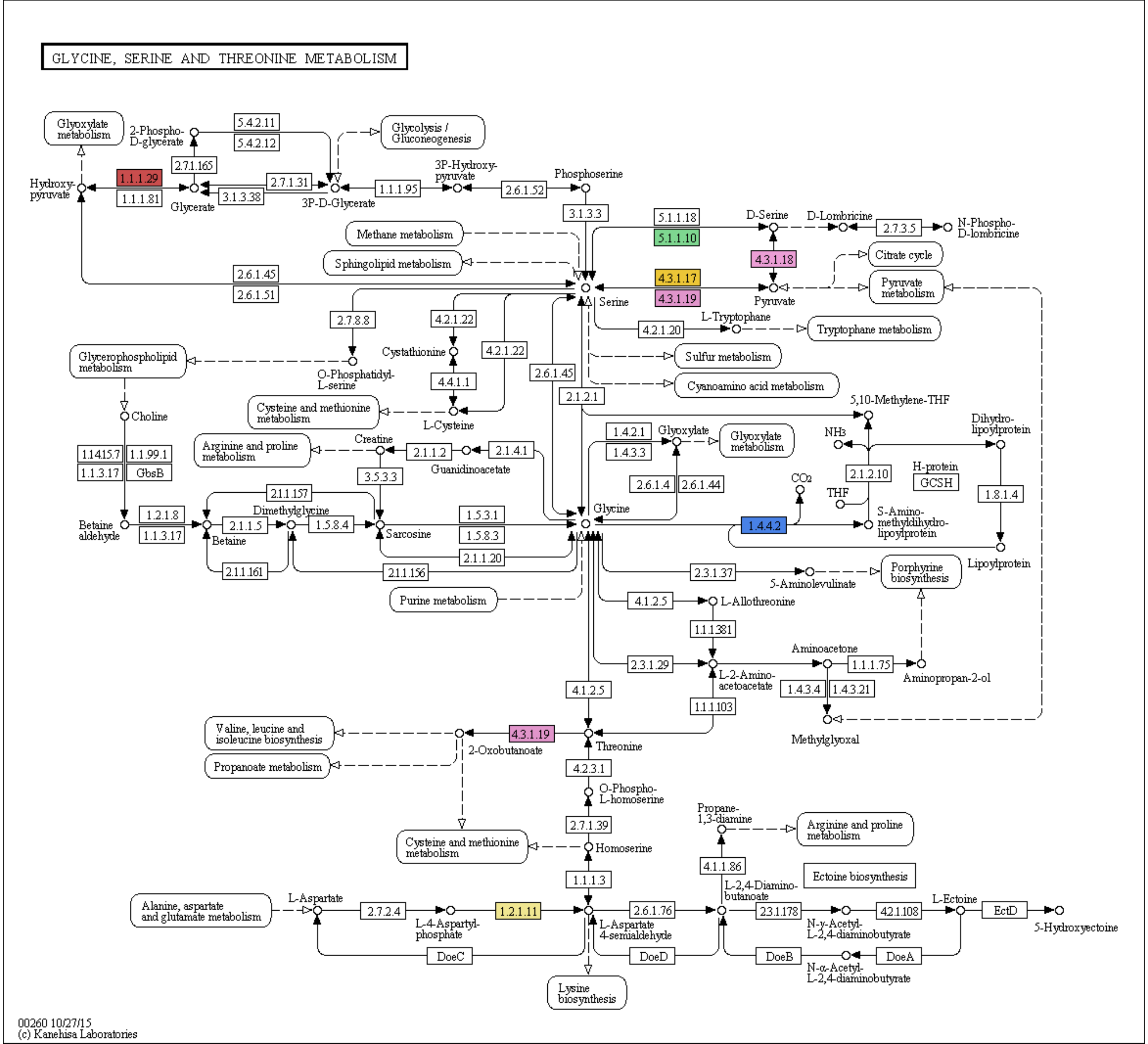
GLYCINE, SERINE AND THREONINE METABOLISM
Glyoxylate metabolism
2-Phospho-D-glycerate
5.4.2.11
5.4.2.12
Glycolysis / Gluconeogenesis
2.7.1.165
Hydroxy-pyruvate
1.1.1.29
1.1.1.81
Glycerate
2.7.1.31
3.1.3.38
3P-D-Glycerate
1.1.1.95
3P-Hydroxy-pyruvate
2.6.1.52
Phosphoserine
3.1.3.3
Methane metabolism
Sphingolipid metabolism
5.1.1.18
5.1.1.10
D-Serine
D-Lombricine
2.7.3.5
N-Phospho-D-lombricine
4.3.1.18
Citrate cycle
2.6.1.45
2.6.1.51
4.3.1.17
4.3.1.19
Serine
Pyruvate
Pyruvate metabolism
2.7.8.8
4.2.1.22
4.2.1.20
L-Tryptophane
Tryptophane metabolism
Glycerophospholipid metabolism
O-Phosphatidyl-L-serine
Cystathionine
4.2.1.22
2.6.1.45
Sulfur metabolism
Cyanoamino acid metabolism
4.4.1.1
2.1.2.1
Cysteine and methionine metabolism
L-Cysteine
5,10-Methylene-THF
Choline
Arginine and proline metabolism
Creatine
2.1.1.2
Guanidinoacetate
2.1.4.1
1.4.2.1
1.4.3.3
Glyoxylate
Glyoxylate metabolism
NH3
Dihydro-lipoylprotein
1.14.15.7
1.1.99.1
1.1.3.17
GbsB
3.5.3.3
2.1.2.10
H-protein
GCSH
2.1.1.157
2.6.1.4
2.6.1.44
CO2
THF
1.8.1.4
Dimethylglycine
1.2.1.8
1.1.3.17
Betaine aldehyde
2.1.1.5
1.5.8.4
Betaine
Sarcosine
1.5.3.1
1.5.8.3
Glycine
1.4.4.2
S-Amino-methyldihydro-lipoylprotein
2.1.1.20
2.1.1.161
2.1.1.156
2.3.1.37
5-Aminolevulinate
Porphyrine biosynthesis
Lipoylprotein
Purine metabolism
4.1.2.5
L-Allothreonine
1.1.1.381
Aminoacetone
2.3.1.29
L-2-Amino-acetoacetate
1.1.1.75
Aminopropan-2-ol
4.1.2.5
1.4.3.4
1.4.3.21
1.1.1.103
Methylglyoxal
Valine, leucine and isoleucine biosynthesis
4.3.1.19
2-Oxobutanoate
Threonine
Propanoate metabolism
4.2.3.1
O-Phospho-L-homoserine
2.7.1.39
Propane-1,3-diamine
Arginine and proline metabolism
Cysteine and methionine metabolism
Homoserine
4.1.1.86
Ectoine biosynthesis
1.1.1.3
L-2,4-Diamino-butanoate
Alanine, aspartate and glutamate metabolism
L-Aspartate
2.7.2.4
1.2.1.11
2.6.1.76
2.3.1.178
4.2.1.108
L-Ectoine
EctD
L-4-Aspartyl-phosphate
L-Aspartate 4-semialdehyde
N-γ-Acetyl-L-2,4-diaminobutyrate
5-Hydroxyectoine
DoeC
DoeD
DoeB
DoeA
N-α-Acetyl-L-2,4-diaminobutyrate
Lysine biosynthesis
00260 10/27/15
(c) Kanehisa Laboratories

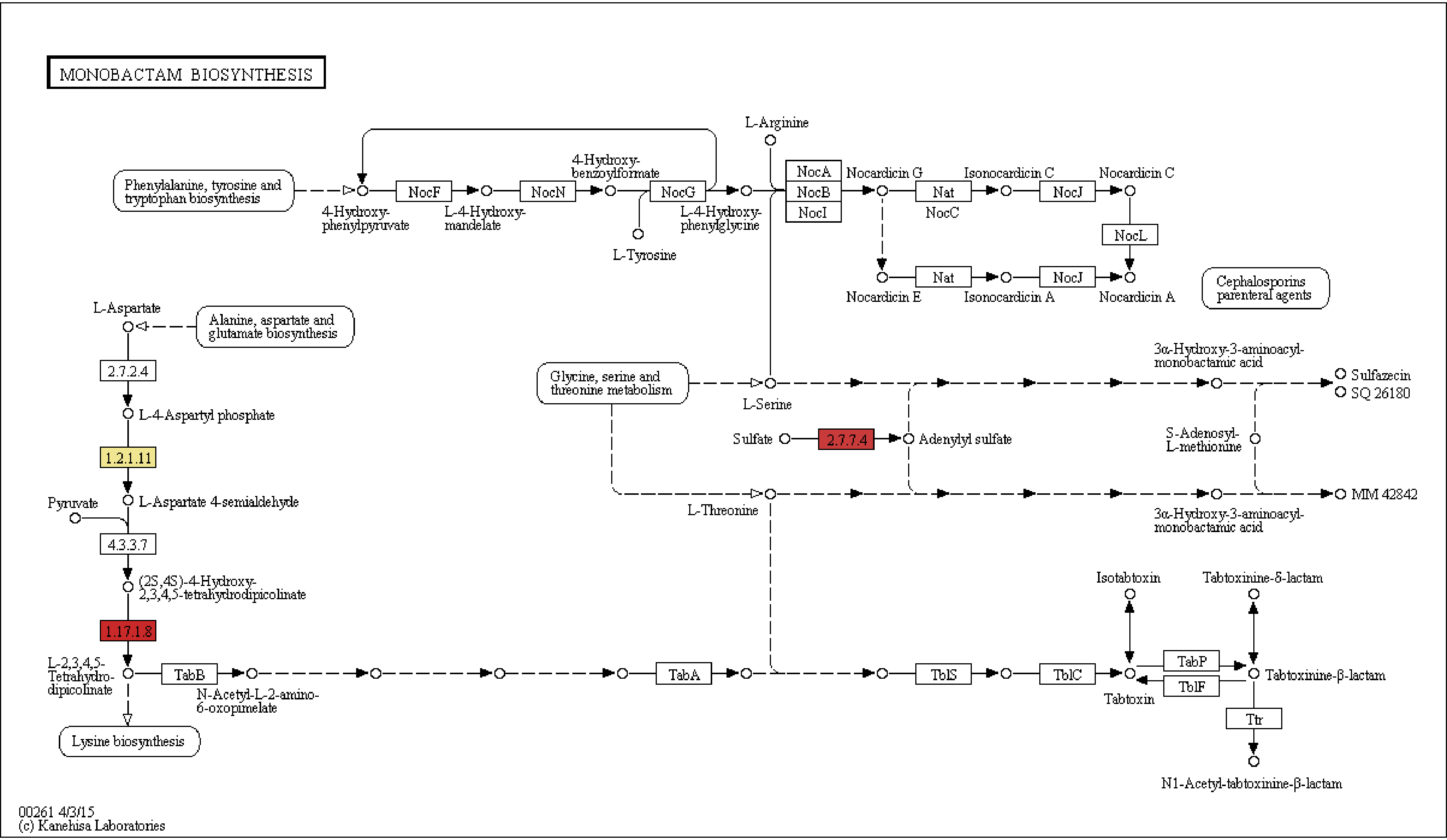
MONOBACTAM BIOSYNTHESIS
Phenylalanine, tyrosine and tryptophan biosynthesis
4-Hydroxy-phenylpyruvate
NocF
L-4-Hydroxy-mandelate
NocN
4-Hydroxy-benzoylformate
NocG
L-Tyrosine
L-4-Hydroxy-phenylglycine
L-Arginine
NocA
NocB
NocI
Nocardicin G
Nat
NocC
Isonocardicin C
NocJ
Nocardicin C
NocL
Nocardicin E
Nat
Isonocardicin A
NocJ
Nocardicin A
Cephalosporins parenteral agents
L-Aspartate
Alanine, aspartate and glutamate biosynthesis
2.7.2.4
L-4-Aspartyl phosphate
1.2.1.11
L-Aspartate 4-semialdehyde
Pyruvate
4.3.3.7
(2S,4S)-4-Hydroxy-2,3,4,5-tetrahydrodipicolinate
1.17.1.8
L-2,3,4,5-Tetrahydro-dipicolinate
Lysine biosynthesis
TabB
N-Acetyl-L-2-amino-6-oxopimelate
TabA
Glycine, serine and threonine metabolism
L-Serine
Sulfate
2.7.7.4
Adenylyl sulfate
L-Threonine
3α-Hydroxy-3-aminoacyl-monobactamic acid
Sulfazecin
SQ 26180
S-Adenosyl-L-methionine
3α-Hydroxy-3-aminoacyl-monobactamic acid
MM 42842
TblS
TblC
Isotabtoxin
Tabtoxin
TabP
TblF
Tabtoxinine-δ-lactam
Tabtoxinine-β-lactam
Ttr
N1-Acetyl-tabtoxinine-β-lactam
00261 4/3/15
(c) Kanehisa Laboratories

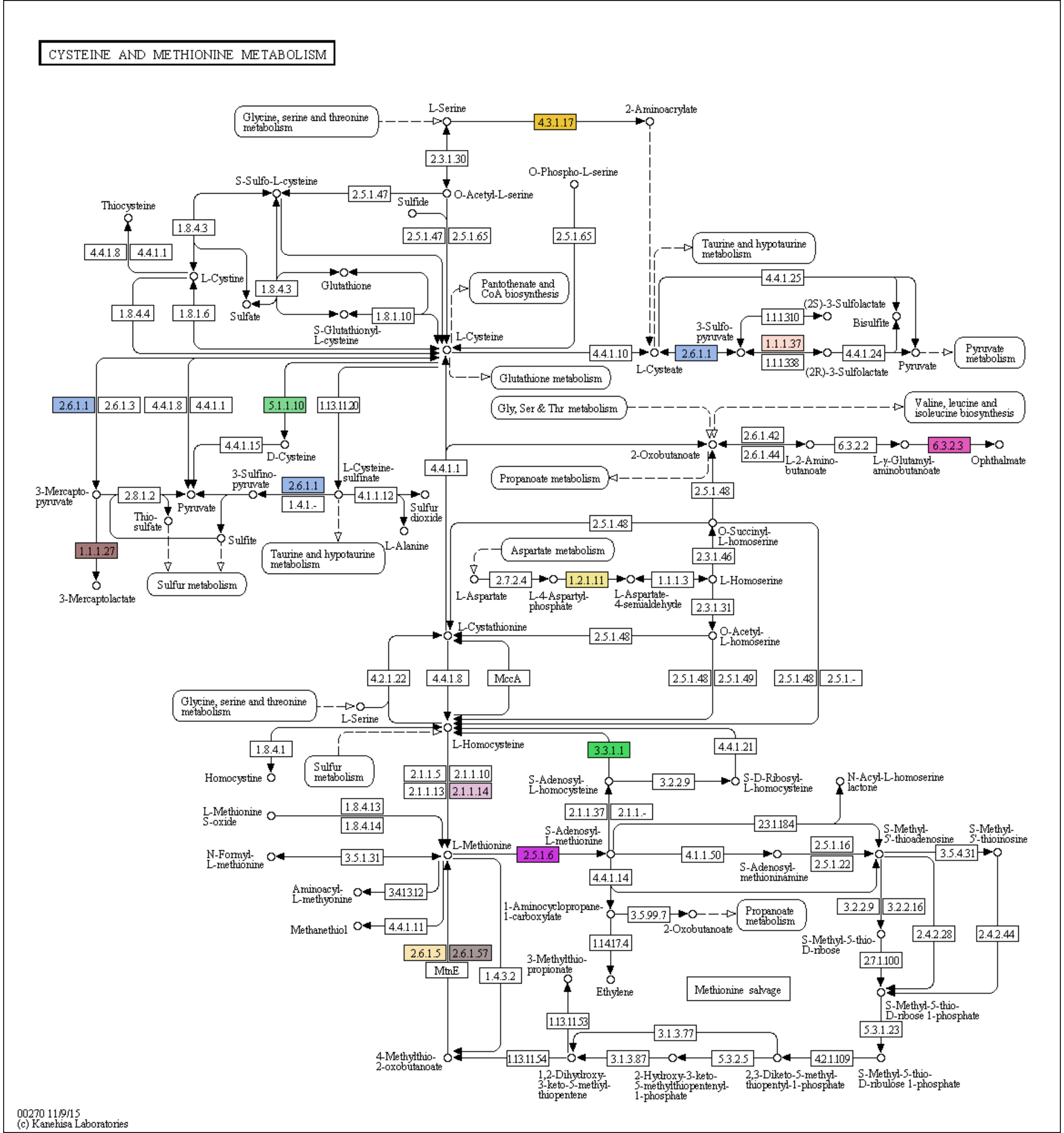
CYSTEINE AND METHIONINE METABOLISM
Glycine, serine and threonine metabolism
L-Serine
4.3.1.17
2-Aminoacrylate
2.3.1.30
S-Sulfo-L-cysteine
2.5.1.47
O-Acetyl-L-serine
O-Phospho-L-serine
Thiocysteine
Sulfide
1.8.4.3
2.5.1.47
2.5.1.65
2.5.1.65
4.4.1.8
4.4.1.1
Taurine and hypotaurine metabolism
L-Cystine
1.8.4.3
Glutathione
Pantothenate and CoA biosynthesis
4.4.1.25
1.8.4.4
1.8.1.6
Sulfate
1.8.1.10
(2S)-3-Sulfolactate
1.1.1.310
Bisulfite
S-Glutathionyl-L-cysteine
L-Cysteine
3-Sulfopyruvate
1.1.1.37
4.4.1.10
2.6.1.1
4.4.1.24
Pyruvate metabolism
1.1.1.338
L-Cysteate
(2R)-3-Sulfolactate
Pyruvate
Glutathione metabolism
2.6.1.1
2.6.1.3
4.4.1.8
4.4.1.1
5.1.1.10
1.13.11.20
Gly, Ser & Thr metabolism
Valine, leucine and isoleucine biosynthesis
2.6.1.42
4.4.1.15
6.3.2.2
6.3.2.3
D-Cysteine
2.6.1.44
2-Oxobutanoate
L-2-Aminobutanoate
L-γ-Glutamylaminobutanoate
Ophthalmate
3-Sulfinopyruvate
L-Cysteine-sulfinate
4.4.1.1
Propanoate metabolism
3-Mercaptopyruvate
2.8.1.2
2.6.1.1
4.1.1.12
Pyruvate
1.4.1.-
Sulfur dioxide
2.5.1.48
Thiosulfate
Sulfite
2.5.1.48
O-Succinyl-L-homoserine
1.1.1.27
Taurine and hypotaurine metabolism
L-Alanine
Aspartate metabolism
2.3.1.46
Sulfur metabolism
2.7.2.4
1.2.1.11
1.1.1.3
L-Homoserine
3-Mercaptolactate
L-Aspartate
L-4-Aspartyl-phosphate
L-Aspartate-4-semialdehyde
2.3.1.31
L-Cystathionine
2.5.1.48
O-Acetyl-L-homoserine
4.2.1.22
4.4.1.8
MccA
2.5.1.48
2.5.1.49
2.5.1.48
2.5.1.-
Glycine, serine and threonine metabolism
L-Serine
L-Homocysteine
1.8.4.1
3.3.1.1
4.4.1.21
Homocystine
Sulfur metabolism
2.1.1.5
2.1.1.10
S-Adenosyl-L-homocysteine
3.2.2.9
S-D-Ribosyl-L-homocysteine
2.1.1.13
2.1.1.14
N-Acyl-L-homoserine lactone
L-Methionine S-oxide
1.8.4.13
2.1.1.37
2.1.1.-
1.8.4.14
2.3.1.184
S-Adenosyl-L-methionine
S-Methyl-5'-thioadenosine
S-Methyl-5'-thioinosine
2.5.1.16
N-Formyl-L-methionine
3.5.1.31
L-Methionine
2.5.1.6
4.1.1.50
3.5.4.31
2.5.1.22
S-Adenosylmethioninamine
4.4.1.14
Aminoacyl-L-methionine
3.4.13.12
1-Aminocyclopropane-1-carboxylate
3.5.99.7
Propanoate metabolism
3.2.2.9
3.2.2.16
2-Oxobutanoate
Methanethiol
4.4.1.11
1.14.17.4
2.4.2.28
2.4.2.44
S-Methyl-5-thio-D-ribose
2.6.1.5
2.6.1.57
3-Methylthiopropionate
2.7.1.100
MtnE
1.4.3.2
Ethylene
Methionine salvage
S-Methyl-5-thio-D-ribose 1-phosphate
1.13.11.53
3.1.3.77
5.3.1.23
4-Methylthio-2-oxobutanoate
1.13.11.54
3.1.3.87
5.3.2.5
4.2.1.109
1,2-Dihydroxy-3-keto-5-methylthiopentene
2-Hydroxy-3-keto-5-methylthiopentenyl-1-phosphate
2,3-Diketo-5-methylthiopentyl-1-phosphate
S-Methyl-5-thio-D-ribulose 1-phosphate
00270 11/9/15
(c) Kanehisa Laboratories

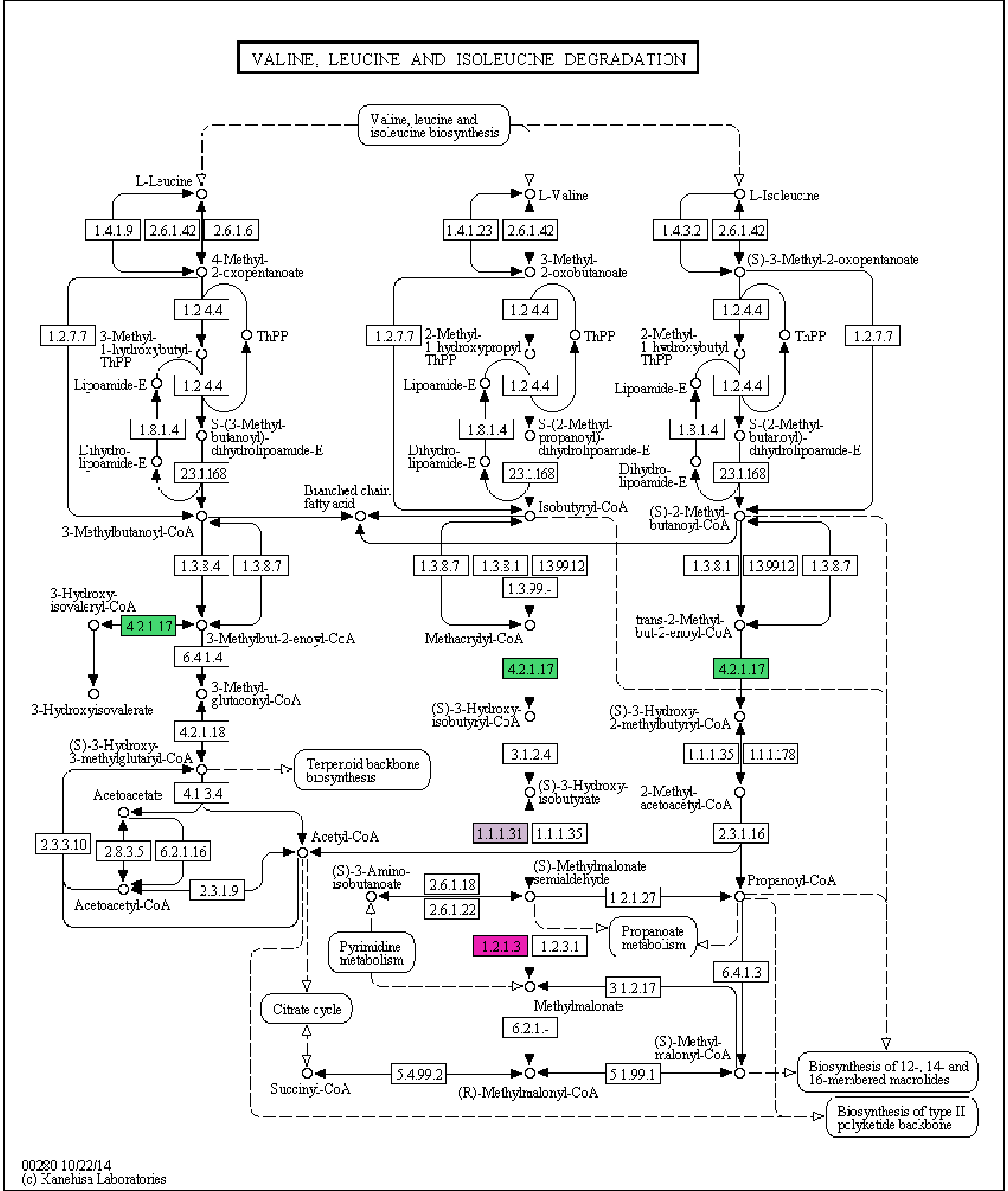
VALINE, LEUCINE AND ISOLEUCINE DEGRADATION
Valine, leucine and isoleucine biosynthesis
L-Leucine
L-Valine
L-Isoleucine
1.4.1.9
2.6.1.42
2.6.1.6
1.4.1.23
2.6.1.42
1.4.3.2
2.6.1.42
4-Methyl-2-oxopentanoate
3-Methyl-2-oxobutanoate
(S)-3-Methyl-2-oxopentanoate
1.2.4.4
1.2.7.7
ThPP
3-Methyl-1-hydroxybutyl-ThPP
2-Methyl-1-hydroxypropyl-ThPP
2-Methyl-1-hydroxybutyl-ThPP
Lipoamide-E
1.8.1.4
Dihydro-lipoamide-E
S-(3-Methylbutanoyl)-dihydrolipoamide-E
S-(2-Methylpropanoyl)-dihydrolipoamide-E
S-(2-Methylbutanoyl)-dihydrolipoamide-E
2.3.1.168
Branched chain fatty acid
3-Methylbutanoyl-CoA
Isobutyryl-CoA
(S)-2-Methylbutanoyl-CoA
1.3.8.4
1.3.8.7
1.3.8.1
1.3.99.12
1.3.99.-
3-Hydroxy-isovaleryl-CoA
4.2.1.17
3-Methylbut-2-enoyl-CoA
Methacrylyl-CoA
trans-2-Methyl-but-2-enoyl-CoA
3-Hydroxyisovalerate
6.4.1.4
3-Methyl-glutaconyl-CoA
4.2.1.18
(S)-3-Hydroxy-3-methylglutaryl-CoA
Terpenoid backbone biosynthesis
(S)-3-Hydroxy-isobutyryl-CoA
(S)-3-Hydroxy-2-methylbutyryl-CoA
3.1.2.4
1.1.1.35
1.1.1.178
(S)-3-Hydroxy-isobutyrate
2-Methyl-acetoacetyl-CoA
Acetoacetate
4.1.3.4
2.3.3.10
2.8.3.5
6.2.1.16
Acetyl-CoA
1.1.1.31
2.3.1.16
Acetoacetyl-CoA
2.3.1.9
(S)-3-Amino-isobutanoate
2.6.1.18
2.6.1.22
(S)-Methylmalonate semialdehyde
1.2.1.27
Propanoyl-CoA
Pyrimidine metabolism
Propanoate metabolism
1.2.1.3
1.2.3.1
6.4.1.3
3.1.2.17
Methylmalonate
Citrate cycle
6.2.1.-
(S)-Methyl-malonyl-CoA
5.4.99.2
5.1.99.1
Succinyl-CoA
(R)-Methylmalonyl-CoA
Biosynthesis of 12-, 14- and 16-membered macrolides
Biosynthesis of type II polyketide backbone
00280 10/22/14
(c) Kanehisa Laboratories

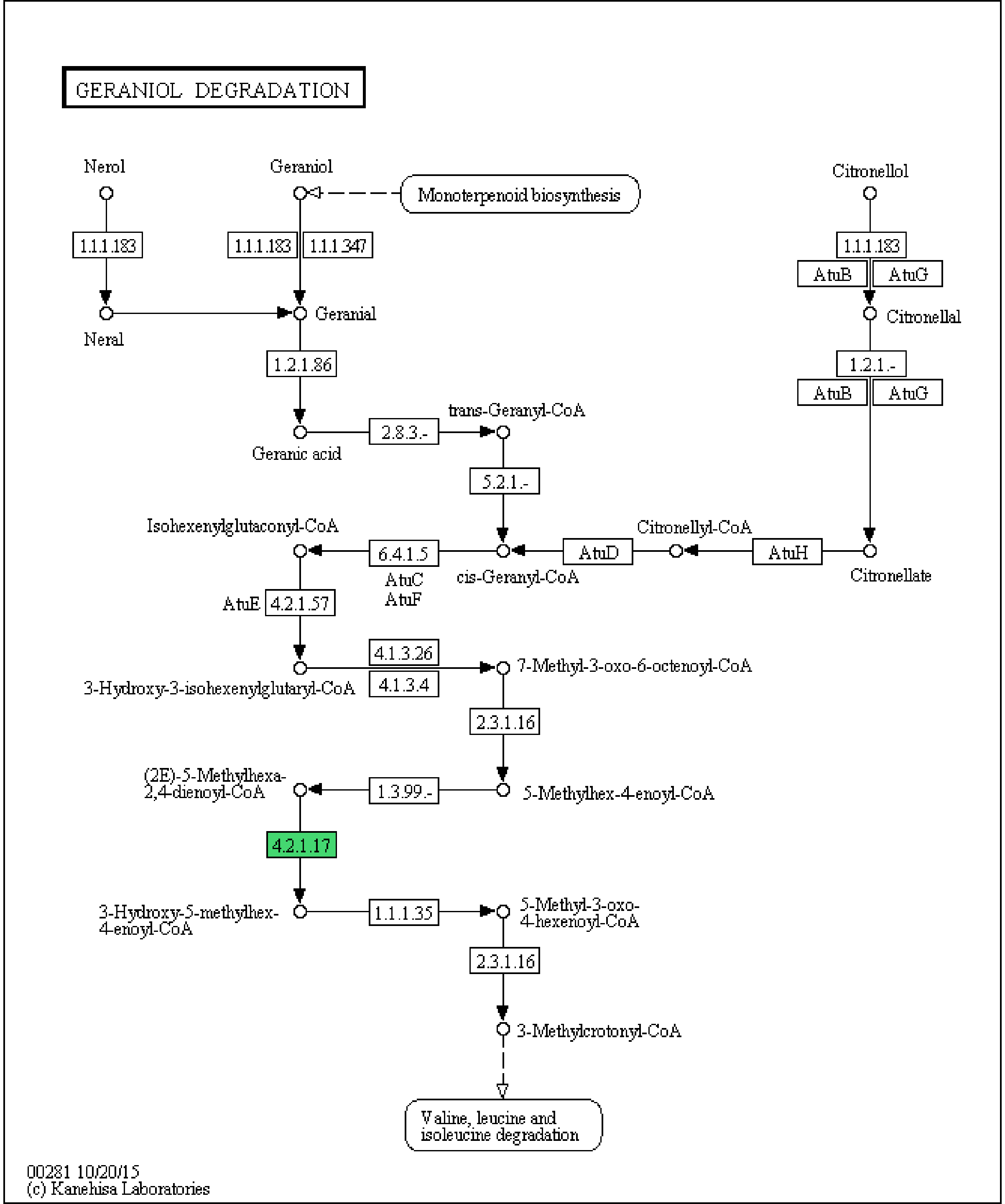
GERANIOL DEGRADATION
Nerol
Geraniol
Monoterpenoid biosynthesis
Citronellol
1.1.1.183
1.1.1.183
1.1.1.347
1.1.1.183
AtuB
AtuG
Geranial
Neral
Citronellal
1.2.1.86
1.2.1.-
AtuB
AtuG
trans-Geranyl-CoA
2.8.3.-
Geranic acid
5.2.1.-
Isohexenylglutaconyl-CoA
Citronellyl-CoA
6.4.1.5
AtuD
AtuH
AtuC
AtuF
cis-Geranyl-CoA
Citronellate
AtuE
4.2.1.57
4.1.3.26
7-Methyl-3-oxo-6-octenoyl-CoA
4.1.3.4
3-Hydroxy-3-isohexenylglutaryl-CoA
2.3.1.16
(2E)-5-Methylhexa-
2,4-dienoyl-CoA
1.3.99.-
5-Methylhex-4-enoyl-CoA
4.2.1.17
3-Hydroxy-5-methylhex-
4-enoyl-CoA
1.1.1.35
5-Methyl-3-oxo-
4-hexenoyl-CoA
2.3.1.16
3-Methylcrotonyl-CoA
Valine, leucine and
isoleucine degradation
00281 10/20/15
(c) Kanehisa Laboratories

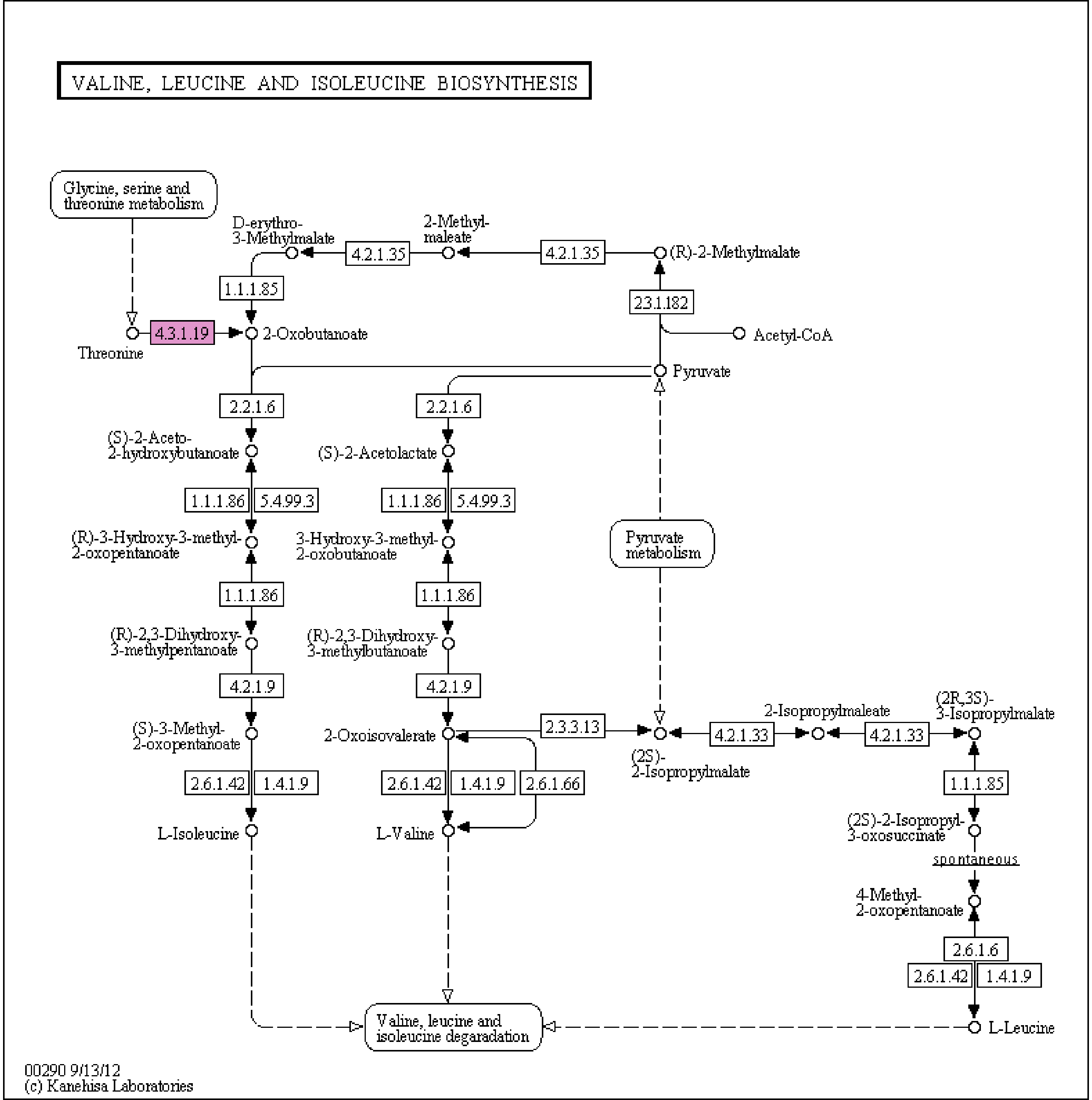
VALINE, LEUCINE AND ISOLEUCINE BIOSYNTHESIS
Glycine, serine and threonine metabolism
D-erythro-3-Methylmalate
2-Methyl-maleate
4.2.1.35
4.2.1.35
(R)-2-Methylmalate
1.1.1.85
2.3.1.182
Threonine
4.3.1.19
2-Oxobutanoate
Acetyl-CoA
Pyruvate
2.2.1.6
2.2.1.6
(S)-2-Aceto-2-hydroxybutanoate
(S)-2-Acetolactate
1.1.1.86
5.4.99.3
1.1.1.86
5.4.99.3
(R)-3-Hydroxy-3-methyl-2-oxopentanoate
3-Hydroxy-3-methyl-2-oxobutanoate
Pyruvate metabolism
1.1.1.86
1.1.1.86
(R)-2,3-Dihydroxy-3-methylpentanoate
(R)-2,3-Dihydroxy-3-methylbutanoate
4.2.1.9
4.2.1.9
(S)-3-Methyl-2-oxopentanoate
2-Oxoisovalerate
2.3.3.13
(2S)-2-Isopropylmalate
4.2.1.33
2-Isopropylmaleate
4.2.1.33
(2R,3S)-3-Isopropylmalate
2.6.1.42
1.4.1.9
2.6.1.42
1.4.1.9
2.6.1.66
1.1.1.85
L-Isoleucine
L-Valine
(2S)-2-Isopropyl-3-oxosuccinate
spontaneous
4-Methyl-2-oxopentanoate
2.6.1.6
2.6.1.42
1.4.1.9
Valine, leucine and isoleucine degaradation
L-Leucine
00290 9/13/12
(c) Kanehisa Laboratories

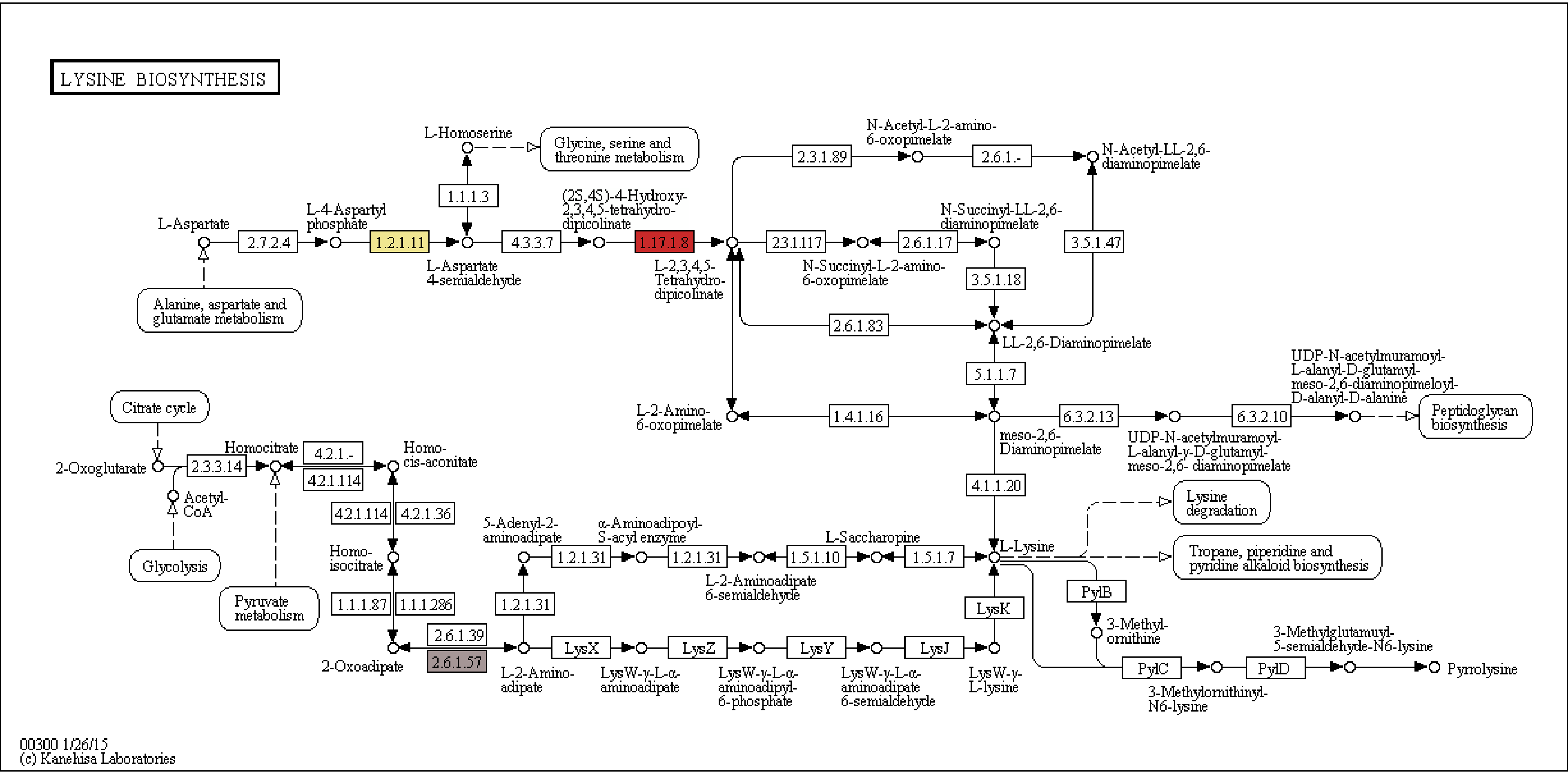
LYSINE BIOSYNTHESIS
L-Homoserine
Glycine, serine and threonine metabolism
1.1.1.3
L-Aspartate
2.7.2.4
L-4-Aspartyl phosphate
1.2.1.11
L-Aspartate 4-semialdehyde
4.3.3.7
(2S,4S)-4-Hydroxy-2,3,4,5-tetrahydro-dipicolinate
1.17.1.8
L-2,3,4,5-Tetrahydro-dipicolinate
Alanine, aspartate and glutamate metabolism
2.3.1.89
N-Acetyl-L-2-amino-6-oxopimelate
2.6.1.-
N-Acetyl-LL-2,6-diaminopimelate
2.3.1.117
2.6.1.17
N-Succinyl-LL-2,6-diaminopimelate
N-Succinyl-L-2-amino-6-oxopimelate
3.5.1.47
3.5.1.18
2.6.1.83
LL-2,6-Diaminopimelate
5.1.1.7
L-2-Amino-6-oxopimelate
1.4.1.16
meso-2,6-Diaminopimelate
6.3.2.13
UDP-N-acetylmuramoyl-L-alanyl-γ-D-glutamyl-meso-2,6- diaminopimelate
6.3.2.10
UDP-N-acetylmuramoyl-L-alanyl-D-glutamyl-meso-2,6-diaminopimeloyl-D-alanyl-D-alanine
Peptidoglycan biosynthesis
4.1.1.20
Lysine degradation
Tropane, piperidine and pyridine alkaloid biosynthesis
Citrate cycle
2-Oxoglutarate
2.3.3.14
Homocitrate
4.2.1.-
4.2.1.114
Homo-cis-aconitate
Acetyl-CoA
Glycolysis
Pyruvate metabolism
4.2.1.114
4.2.1.36
Homo-isocitrate
1.1.1.87
1.1.1.286
2-Oxoadipate
2.6.1.39
2.6.1.57
5-Adenyl-2-aminoadipate
1.2.1.31
α-Aminoadipoyl-S-acyl enzyme
1.2.1.31
L-2-Aminoadipate 6-semialdehyde
1.5.1.10
L-Saccharopine
1.5.1.7
L-Lysine
1.2.1.31
L-2-Amino-adipate
LysX
LysW-γ-L-α-aminoadipate
LysZ
LysW-γ-L-α-aminoadipyl-6-phosphate
LysY
LysW-γ-L-α-aminoadipate 6-semialdehyde
LysJ
LysW-γ-L-lysine
LysK
PylB
3-Methyl-ornithine
PylC
3-Methylornithinyl-N6-lysine
PylD
3-Methylglutamuyl-5-semialdehyde-N6-lysine
Pyrrolysine
00300 1/26/15
(c) Kanehisa Laboratories

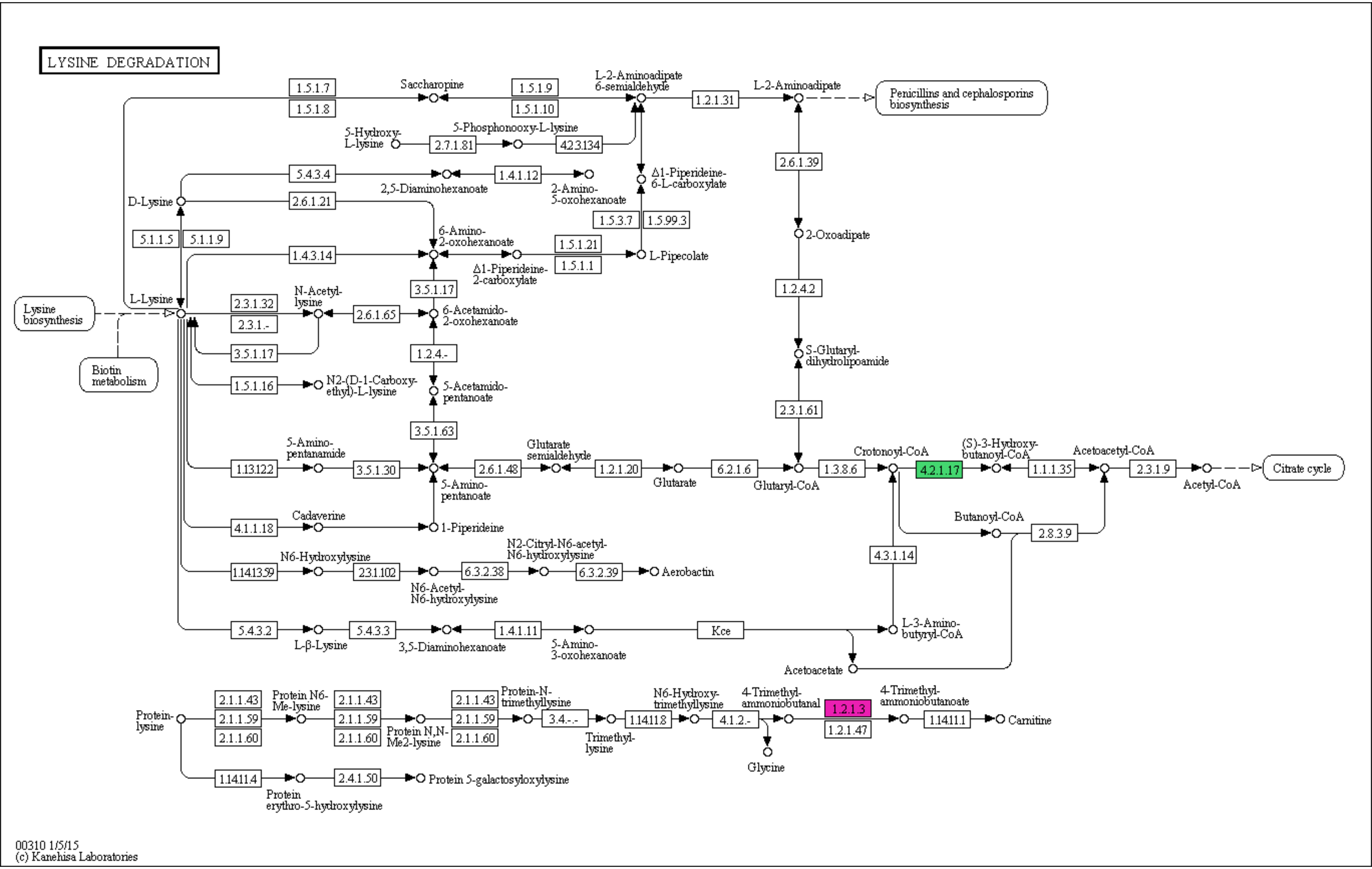
LYSINE DEGRADATION
1.5.1.7
1.5.1.8
Saccharopine
1.5.1.9
1.5.1.10
L-2-Aminoadipate 6-semialdehyde
1.2.1.31
L-2-Aminoadipate
Penicillins and cephalosporins biosynthesis
5-Hydroxy-L-lysine
2.7.1.81
5-Phosphonooxy-L-lysine
4.2.3.134
5.4.3.4
2,5-Diaminohexanoate
1.4.1.12
2-Amino-5-oxohexanoate
Δ1-Piperideine-6-L-carboxylate
2.6.1.39
D-Lysine
2.6.1.21
1.5.3.7
1.5.99.3
2-Oxoadipate
5.1.1.5
5.1.1.9
6-Amino-2-oxohexanoate
1.5.1.21
1.5.1.1
L-Pipecolate
1.4.3.14
Δ1-Piperideine-2-carboxylate
3.5.1.17
1.2.4.2
L-Lysine
2.3.1.32
2.3.1.-
N-Acetyl-lysine
2.6.1.65
6-Acetamido-2-oxohexanoate
Lysine biosynthesis
3.5.1.17
1.2.4.-
S-Glutaryl-dihydrolipoamide
Biotin metabolism
1.5.1.16
N2-(D-1-Carboxy-ethyl)-L-lysine
5-Acetamido-pentanoate
2.3.1.61
3.5.1.63
5-Amino-pentanamide
Glutarate semialdehyde
Crotonoyl-CoA
(S)-3-Hydroxy-butanoyl-CoA
Acetoacetyl-CoA
1.13.12.2
3.5.1.30
2.6.1.48
1.2.1.20
6.2.1.6
1.3.8.6
4.2.1.17
1.1.1.35
2.3.1.9
Citrate cycle
5-Amino-pentanoate
Glutarate
Glutaryl-CoA
Acetyl-CoA
Cadaverine
Butanoyl-CoA
4.1.1.18
1-Piperideine
2.8.3.9
N2-Citryl-N6-acetyl-N6-hydroxylysine
N6-Hydroxylysine
4.3.1.14
1.14.13.59
2.3.1.102
6.3.2.38
6.3.2.39
Aerobactin
N6-Acetyl-N6-hydroxylysine
5.4.3.2
5.4.3.3
1.4.1.11
Kce
L-3-Amino-butyryl-CoA
L-β-Lysine
3,5-Diaminohexanoate
5-Amino-3-oxohexanoate
Acetoacetate
2.1.1.43
2.1.1.59
2.1.1.60
Protein N6-Me-lysine
Protein-N-trimethyllysine
N6-Hydroxy-trimethyllysine
4-Trimethyl-ammoniobutanal
4-Trimethyl-ammoniobutanoate
Protein-lysine
3.4.-.-
1.14.11.8
4.1.2.-
1.2.1.3
1.2.1.47
1.14.11.1
Carnitine
Protein N,N-Me2-lysine
Trimethyl-lysine
Glycine
1.14.11.4
2.4.1.50
Protein 5-galactosyloxylysine
Protein erythro-5-hydroxylysine
00310 1/5/15
(c) Kanehisa Laboratories

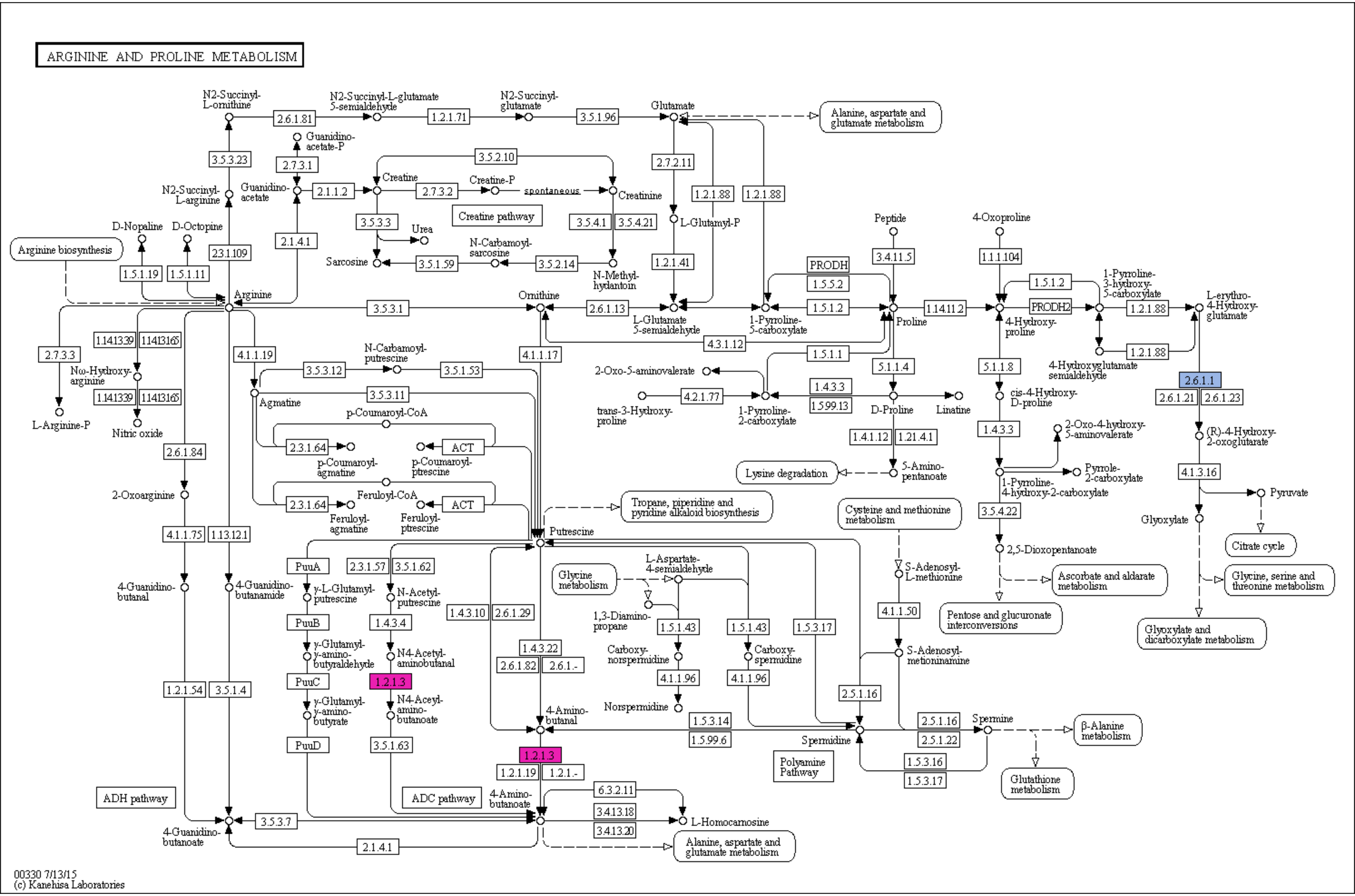

ARGININE AND PROLINE METABOLISM
N2-Succinyl-L-ornithine
2.6.1.81
N2-Succinyl-L-glutamate 5-semialdehyde
1.2.1.71
N2-Succinyl-glutamate
3.5.1.96
Glutamate
Alanine, aspartate and glutamate metabolism
3.5.3.23
Guanidino-acetate-P
2.7.3.1
3.5.2.10
N2-Succinyl-L-arginine
Guanidino-acetate
2.1.1.2
Creatine
2.7.3.2
Creatine-P
spontaneous
Creatinine
2.7.2.11
1.2.1.88
1.2.1.88
Creatine pathway
3.5.4.1
3.5.4.21
L-Glutamyl-P
Peptide
4-Oxoproline
D-Nopaline
D-Octopine
3.5.3.3
Urea
2.1.4.1
N-Carbamoyl-sarcosine
Arginine biosynthesis
23.1.109
Sarcosine
3.5.1.59
3.5.2.14
N-Methyl-hydantoin
1.2.1.41
PRODH
3.4.11.5
1.1.1.104
1.5.1.19
1.5.1.11
1.5.5.2
1.5.1.2
1-Pyrroline-3-hydroxy-5-carboxylate
Arginine
3.5.3.1
Ornithine
2.6.1.13
L-Glutamate 5-semialdehyde
1-Pyrroline-5-carboxylate
1.5.1.2
Proline
1.14.11.2
PRODH2
1.2.1.88
L-erythro-4-Hydroxy-glutamate
4-Hydroxy-proline
2.7.3.3
1.14.13.39
1.14.13.165
4.1.1.19
4.1.1.17
4.3.1.12
1.5.1.1
1.2.1.88
N-Carbamoyl-putrescine
4-Hydroxyglutamate semialdehyde
Nω-Hydroxy-arginine
3.5.3.12
3.5.1.53
2-Oxo-5-aminovalerate
5.1.1.4
5.1.1.8
2.6.1.1
1.4.3.3
1.14.13.39
1.14.13.165
Agmatine
3.5.3.11
trans-3-Hydroxy-proline
4.2.1.77
1.5.99.13
2.6.1.21
2.6.1.23
cis-4-Hydroxy-D-proline
L-Arginine-P
Nitric oxide
p-Coumaroyl-CoA
1-Pyrroline-2-carboxylate
D-Proline
Linatine
2-Oxo-4-hydroxy-5-aminovalerate
(R)-4-Hydroxy-2-oxoglutarate
2.6.1.84
2.3.1.64
ACT
1.4.3.3
1.4.1.12
1.21.4.1
p-Coumaroyl-agmatine
p-Coumaroyl-ptrescine
Lysine degradation
5-Amino-pentanoate
Pyrrole-2-carboxylate
1-Pyrroline-4-hydroxy-2-carboxylate
4.1.3.16
Pyruvate
2-Oxoarginine
Feruloyl-CoA
2.3.1.64
ACT
Tropane, piperidine and pyridine alkaloid biosynthesis
Cysteine and methionine metabolism
Feruloyl-agmatine
Feruloyl-ptrescine
3.5.4.22
Glyoxylate
Citrate cycle
Putrescine
4.1.1.75
1.13.12.1
PuuA
2.3.1.57
3.5.1.62
L-Aspartate-4-semialdehyde
Glycine metabolism
2,5-Dioxopentanoate
S-Adenosyl-L-methionine
Ascorbate and aldarate metabolism
Glycine, serine and threonine metabolism
4-Guanidino-butanal
4-Guanidino-butanamide
γ-L-Glutamyl-putrescine
N-Acetyl-putrescine
1,3-Diamino-propane
PuuB
1.4.3.4
1.4.3.10
2.6.1.29
1.5.1.43
1.5.1.43
1.5.3.17
4.1.1.50
Pentose and glucuronate interconversions
Glyoxylate and dicarboxylate metabolism
γ-Glutamyl-γ-amino-butyraldehyde
N4-Acetyl-aminobutanal
1.4.3.22
Carboxy-norspermidine
Carboxy-spermidine
S-Adenosyl-metioninamine
2.6.1.82
2.6.1.-
4.1.1.96
4.1.1.96
1.2.1.54
3.5.1.4
PuuC
1.2.1.3
2.5.1.16
γ-Glutamyl-γ-amino-butyrate
N4-Acetyl-amino-butanoate
Norspermidine
4-Amino-butanal
1.5.3.14
2.5.1.16
Spermine
β-Alanine metabolism
1.5.99.6
Spermidine
2.5.1.22
PuuD
3.5.1.63
1.2.1.3
Polyamine Pathway
1.5.3.16
1.2.1.19
1.2.1.-
1.5.3.17
Glutathione metabolism
ADH pathway
ADC pathway
4-Amino-butanoate
6.3.2.11
3.4.13.18
3.5.3.7
L-Homocarnosine
3.4.13.20
4-Guanidino-butanoate
2.1.4.1
Alanine, aspartate and glutamate metabolism
00330 7/13/15
(c) Kanehisa Laboratories

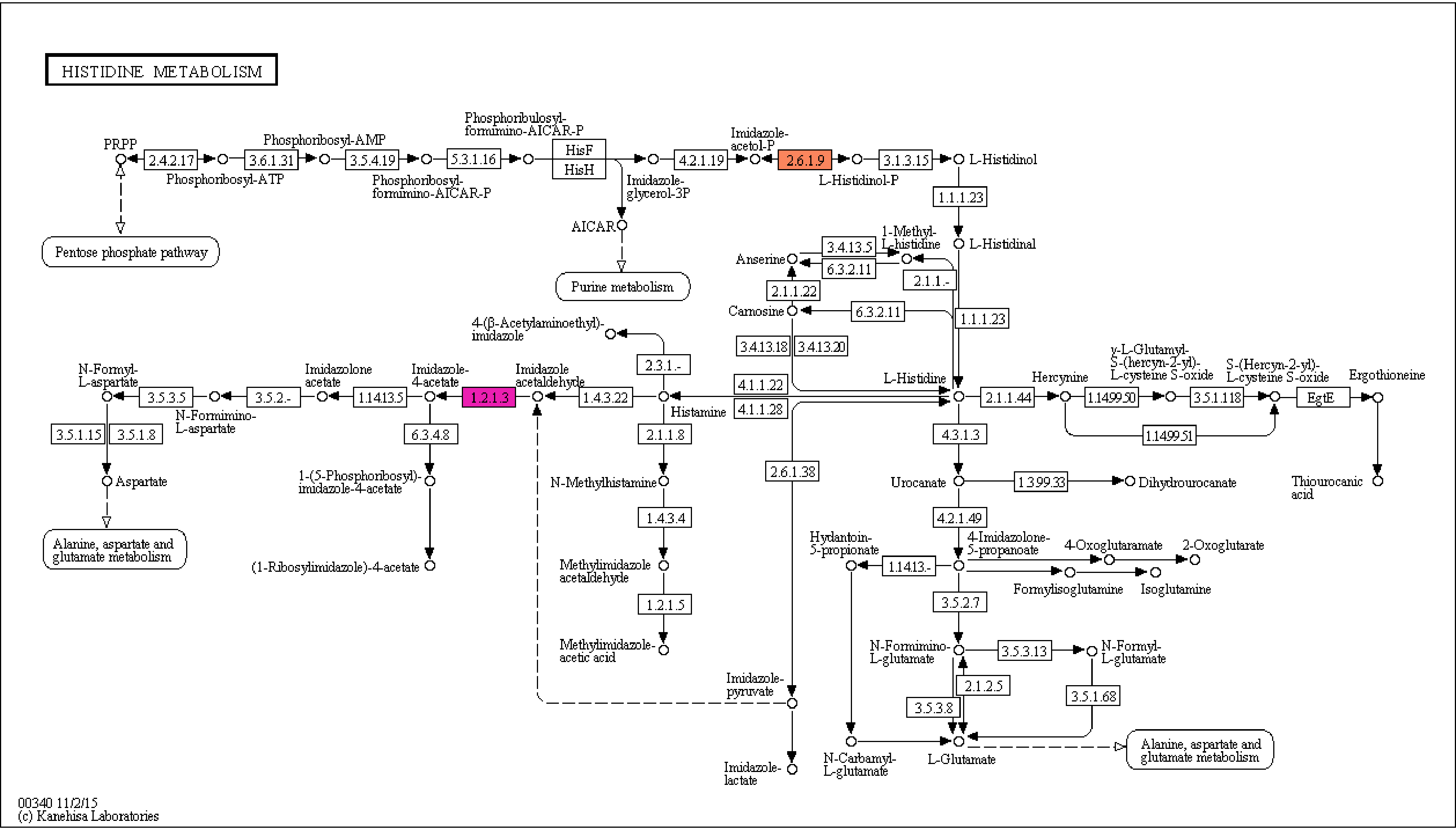
HISTIDINE METABOLISM
PRPP
2.4.2.17
Phosphoribosyl-ATP
3.6.1.31
Phosphoribosyl-AMP
3.5.4.19
Phosphoribosyl-formimino-AICAR-P
5.3.1.16
Phosphoribulosyl-formimino-AICAR-P
HisF
HisH
Imidazole-glycerol-3P
AICAR
Purine metabolism
Pentose phosphate pathway
4.2.1.19
Imidazole-acetol-P
2.6.1.9
L-Histidinol-P
3.1.3.15
L-Histidinol
1.1.1.23
L-Histidinal
1-Methyl-L-histidine
3.4.13.5
6.3.2.11
Anserine
2.1.1.-
2.1.1.22
Carnosine
6.3.2.11
1.1.1.23
3.4.13.18
3.4.13.20
4-(β-Acetylaminoethyl)-imidazole
2.3.1.-
N-Formyl-L-aspartate
3.5.3.5
N-Formimino-L-aspartate
3.5.2.-
Imidazolone acetate
1.14.13.5
Imidazole-4-acetate
1.2.1.3
Imidazole acetaldehyde
1.4.3.22
Histamine
4.1.1.22
4.1.1.28
L-Histidine
2.1.1.44
Hercynine
1.14.99.50
γ-L-Glutamyl-S-(hercyn-2-yl)-L-cysteine S-oxide
3.5.1.118
S-(Hercyn-2-yl)-L-cysteine S-oxide
EgtE
Ergothioneine
1.14.99.51
Thiourocanic acid
3.5.1.15
3.5.1.8
Aspartate
Alanine, aspartate and glutamate metabolism
6.3.4.8
1-(5-Phosphoribosyl)-imidazole-4-acetate
(1-Ribosylimidazole)-4-acetate
2.1.1.8
N-Methylhistamine
1.4.3.4
Methylimidazole acetaldehyde
1.2.1.5
Methylimidazole-acetic acid
2.6.1.38
Imidazole-pyruvate
Imidazole-lactate
4.3.1.3
Urocanate
1.3.99.33
Dihydrourocanate
4.2.1.49
4-Imidazolone-5-propanoate
Hydantoin-5-propionate
1.14.13.-
4-Oxoglutaramate
2-Oxoglutarate
Formylisoglutamine
Isoglutamine
3.5.2.7
N-Formimino-L-glutamate
3.5.3.13
N-Formyl-L-glutamate
2.1.2.5
3.5.3.8
3.5.1.68
N-Carbamyl-L-glutamate
L-Glutamate
Alanine, aspartate and glutamate metabolism
00340 11/2/15
(c) Kanehisa Laboratories

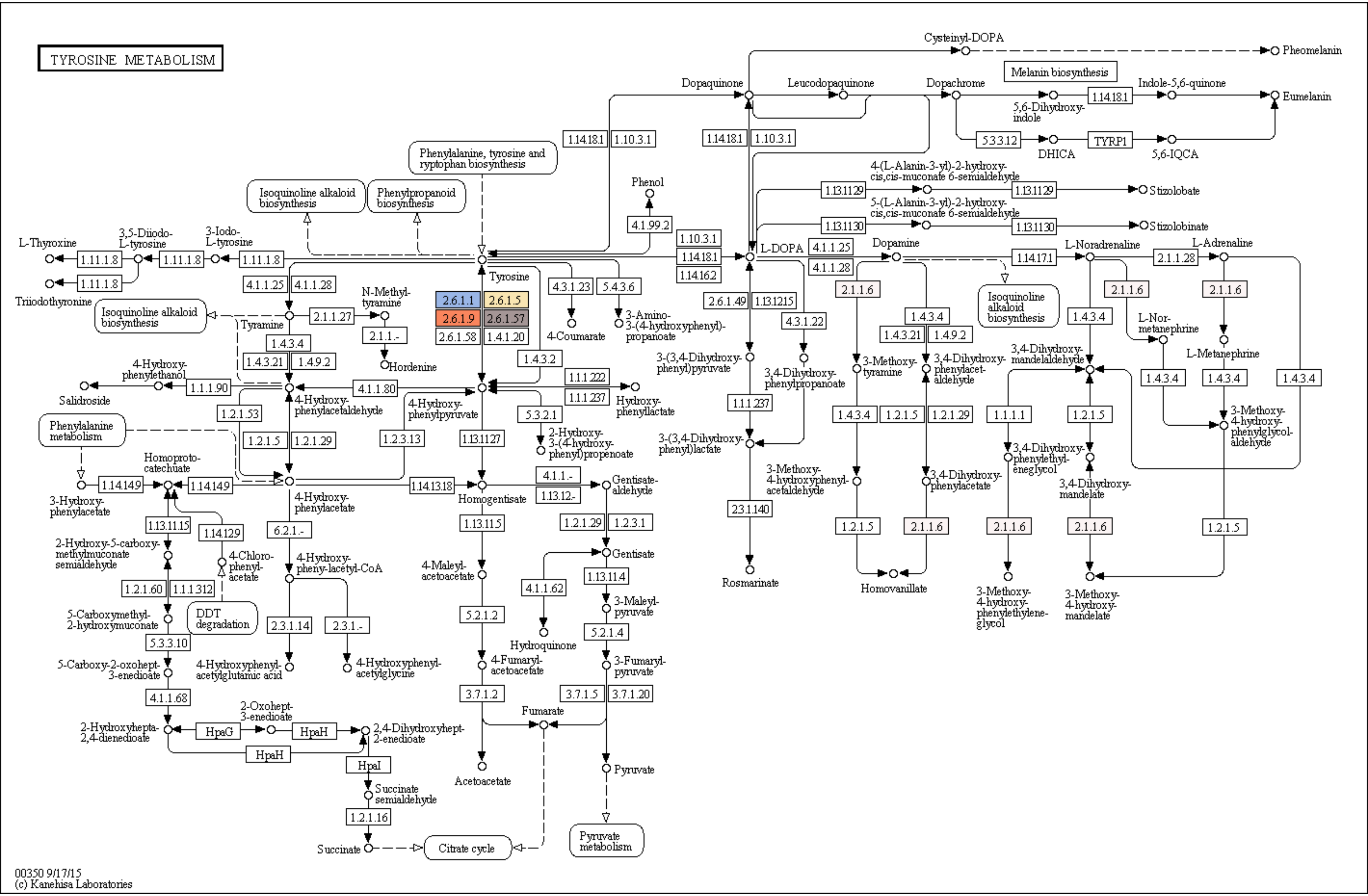
TYROSINE METABOLISM
Tyrosine
Dopaquinone
Leucodopaquinone
Dopachrome
Cysteinyl-DOPA
Pheomelanin
Melanin biosynthesis
Indole-5,6-quinone
Eumelanin
5,6-Dihydroxy-indole
DHICA
5,6-IQCA
L-DOPA
Dopamine
L-Noradrenaline
L-Adrenaline
L-Thyroxine
3,5-Diiodo-L-tyrosine
3-Iodo-L-tyrosine
Triiodothyronine
Tyramine
N-Methyl-tyramine
Hordenine
Phenol
4-Coumarate
3-Amino-3-(4-hydroxyphenyl)-propanoate
4-Hydroxy-phenylethanol
Salidroside
4-Hydroxy-phenylacetaldehyde
4-Hydroxy-phenylpyruvate
Hydroxy-phenyllactate
2-Hydroxy-3-(4-hydroxy-phenyl)propenoate
Homoproto-catechuate
3-Hydroxy-phenylacetate
4-Hydroxy-phenylacetate
Homogentisate
Gentisate-aldehyde
Gentisate
Hydroquinone
3-Maleyl-pyruvate
3-Fumaryl-pyruvate
Pyruvate
4-Maleyl-acetoacetate
4-Fumaryl-acetoacetate
Fumarate
Acetoacetate
4-Hydroxy-pheny-lacetyl-CoA
4-Hydroxyphenyl-acetylglutamic acid
4-Hydroxyphenyl-acetylglycine
2-Hydroxy-5-carboxy-methylmuconate semialdehyde
4-Chloro-phenyl-acetate
5-Carboxymethyl-2-hydroxymuconate
5-Carboxy-2-oxohept-3-enedioate
2-Oxohept-3-enedioate
2-Hydroxyhepta-2,4-dienedioate
2,4-Dihydroxyhept-2-enedioate
Succinate semialdehyde
Succinate
Citrate cycle
Pyruvate metabolism
DDT degradation
Phenylalanine metabolism
Phenylalanine, tyrosine and ryptophan biosynthesis
Isoquinoline alkaloid biosynthesis
Phenylpropanoid biosynthesis
4-(L-Alanin-3-yl)-2-hydroxy-cis,cis-muconate 6-semialdehyde
5-(L-Alanin-3-yl)-2-hydroxy-cis,cis-muconate 6-semialdehyde
Stizolobate
Stizolobinate
3-(3,4-Dihydroxy-phenyl)pyruvate
3,4-Dihydroxy-phenylpropanoate
3-(3,4-Dihydroxy-phenyl)lactate
Rosmarinate
3-Methoxy-tyramine
3,4-Dihydroxy-phenylacet-aldehyde
3-Methoxy-4-hydroxyphenyl-acetaldehyde
3,4-Dihydroxy-phenylacetate
Homovanillate
3,4-Dihydroxy-mandelaldehyde
L-Nor-metanephrine
L-Metanephrine
3-Methoxy-4-hydroxy-phenylglycol-aldehyde
3,4-Dihydroxy-phenylethyl-eneglycol
3,4-Dihydroxy-mandelate
3-Methoxy-4-hydroxy-phenylethylene-glycol
3-Methoxy-4-hydroxy-mandelate
2.6.1.1
2.6.1.5
2.6.1.9
2.6.1.57
2.6.1.58
1.4.1.20
1.14.18.1
1.10.3.1
1.14.16.2
1.13.11.29
1.13.11.30
4.1.1.25
4.1.1.28
1.14.17.1
2.1.1.28
2.1.1.6
5.3.3.12
TYRP1
1.11.1.8
2.1.1.27
2.1.1.-
1.4.3.4
1.4.3.21
1.4.9.2
1.1.1.90
4.1.1.80
4.3.1.23
5.4.3.6
4.1.99.2
1.4.3.2
1.1.1.222
1.1.1.237
5.3.2.1
1.2.1.53
1.2.1.5
1.2.1.29
1.2.3.13
1.13.11.27
1.14.13.18
1.14.14.9
1.13.11.15
1.14.12.9
1.2.1.60
1.1.1.312
5.3.3.10
4.1.1.68
HpaG
HpaH
HpaI
1.2.1.16
6.2.1.-
2.3.1.14
2.3.1.-
1.13.11.5
5.2.1.2
3.7.1.2
4.1.1.-
1.13.12.-
1.2.3.1
4.1.1.62
1.13.11.4
5.2.1.4
3.7.1.5
3.7.1.20
2.6.1.49
1.13.12.15
4.3.1.22
2.3.1.140
1.1.1.1
00350 9/17/15
(c) Kanehisa Laboratories

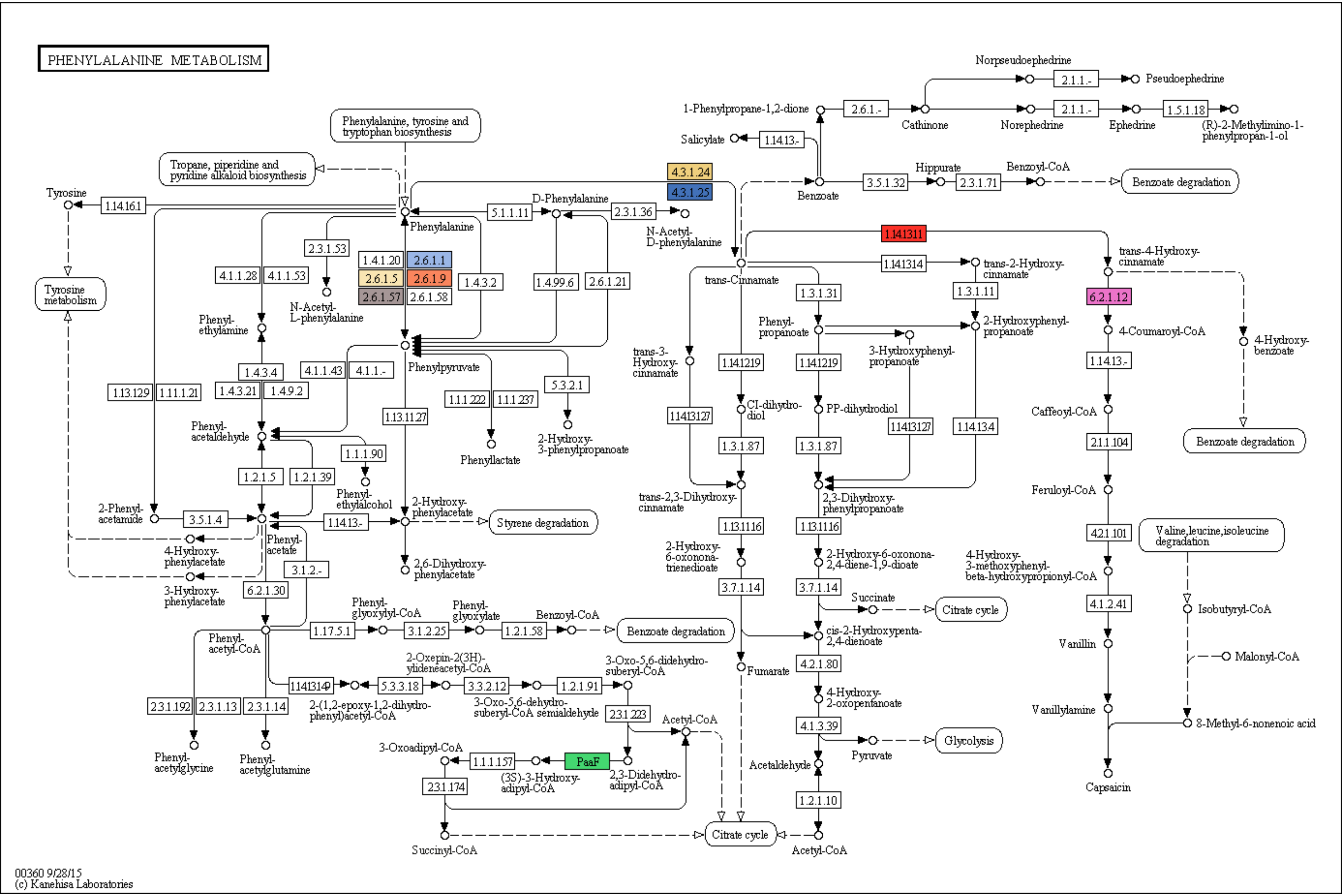
PHENYLALANINE METABOLISM
Phenylalanine, tyrosine and tryptophan biosynthesis
Tropane, piperidine and pyridine alkaloid biosynthesis
Tyrosine
1.14.16.1
Tyrosine metabolism
Phenylalanine
D-Phenylalanine
5.1.1.11
2.3.1.36
N-Acetyl-D-phenylalanine
4.3.1.24
4.3.1.25
2.3.1.53
N-Acetyl-L-phenylalanine
1.4.1.20
2.6.1.1
2.6.1.5
2.6.1.9
2.6.1.57
2.6.1.58
1.4.3.2
1.4.99.6
2.6.1.21
4.1.1.28
4.1.1.53
Phenyl-ethylamine
1.4.3.4
1.4.3.21
1.4.9.2
4.1.1.43
4.1.1.-
Phenylpyruvate
5.3.2.1
1.1.1.222
1.1.1.237
2-Hydroxy-3-phenylpropanoate
Phenyllactate
1.13.12.9
1.11.1.21
1.13.11.27
Phenyl-acetaldehyde
1.1.1.90
Phenyl-ethylalcohol
1.2.1.5
1.2.1.39
2-Phenyl-acetamide
3.5.1.4
1.14.13.-
2-Hydroxy-phenylacetate
Styrene degradation
2,6-Dihydroxy-phenylacetate
4-Hydroxy-phenylacetate
3-Hydroxy-phenylacetate
Phenyl-acetate
3.1.2.-
6.2.1.30
Phenyl-acetyl-CoA
1.17.5.1
Phenyl-glyoxylyl-CoA
3.1.2.25
Phenyl-glyoxylate
1.2.1.58
Benzoyl-CoA
Benzoate degradation
1.14.13.149
2-(1,2-epoxy-1,2-dihydro-phenyl)acetyl-CoA
5.3.3.18
2-Oxepin-2(3H)-ylideneacetyl-CoA
3.3.2.12
3-Oxo-5,6-dehydro-suberyl-CoA semialdehyde
1.2.1.91
3-Oxo-5,6-didehydro-suberyl-CoA
2.3.1.223
Acetyl-CoA
2.3.1.192
2.3.1.13
2.3.1.14
Phenyl-acetylglycine
Phenyl-acetylglutamine
3-Oxoadipyl-CoA
1.1.1.157
(3S)-3-Hydroxy-adipyl-CoA
PaaF
2,3-Didehydro-adipyl-CoA
2.3.1.174
Succinyl-CoA
Citrate cycle
1-Phenylpropane-1,2-dione
2.6.1.-
Cathinone
Norpseudoephedrine
2.1.1.-
Pseudoephedrine
Norephedrine
2.1.1.-
Ephedrine
1.5.1.18
(R)-2-Methylimino-1-phenylpropan-1-ol
Salicylate
1.14.13.-
Benzoate
3.5.1.32
Hippurate
2.3.1.71
Benzoyl-CoA
Benzoate degradation
1.14.13.11
trans-Cinnamate
1.14.13.14
trans-2-Hydroxy-cinnamate
trans-4-Hydroxy-cinnamate
6.2.1.12
4-Coumaroyl-CoA
4-Hydroxy-benzoate
1.14.13.-
Caffeoyl-CoA
2.1.1.104
Feruloyl-CoA
Benzoate degradation
1.3.1.31
1.3.1.11
Phenyl-propanoate
2-Hydroxyphenyl-propanoate
3-Hydroxyphenyl-propanoate
trans-3-Hydroxy-cinnamate
1.14.12.19
1.14.12.19
1.14.13.127
1.14.13.127
1.14.13.4
CI-dihydro-diol
PP-dihydrodiol
1.3.1.87
1.3.1.87
trans-2,3-Dihydroxy-cinnamate
2,3-Dihydroxy-phenylpropanoate
1.13.11.16
1.13.11.16
2-Hydroxy-6-oxonona-trienedioate
2-Hydroxy-6-oxonona-2,4-diene-1,9-dioate
3.7.1.14
3.7.1.14
Succinate
Citrate cycle
cis-2-Hydroxypenta-2,4-dienoate
Fumarate
4.2.1.80
4-Hydroxy-2-oxopentanoate
4.1.3.39
Pyruvate
Glycolysis
Acetaldehyde
1.2.1.10
Acetyl-CoA
Citrate cycle
4.2.1.101
4-Hydroxy-3-methoxyphenyl-beta-hydroxypropionyl-CoA
4.1.2.41
Vanillin
Vanillylamine
Capsaicin
Valine, leucine, isoleucine degradation
Isobutyryl-CoA
Malonyl-CoA
8-Methyl-6-nonenoic acid
00360 9/28/15
(c) Kanehisa Laboratories

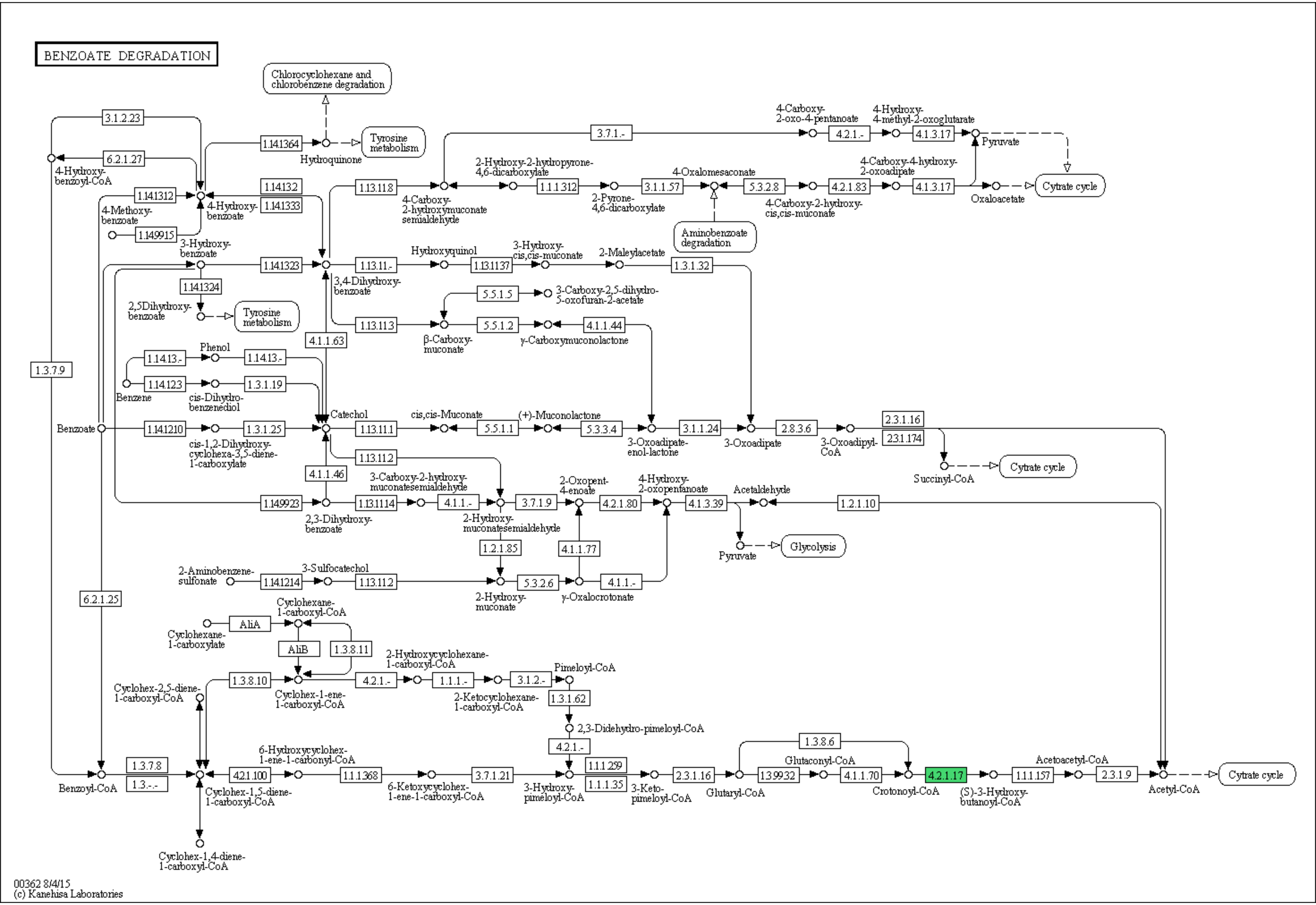
BENZOATE DEGRADATION
Chlorocyclohexane and chlorobenzene degradation
Tyrosine metabolism
Hydroquinone
3.1.2.23
6.2.1.27
1.14.13.64
4-Hydroxy-benzoyl-CoA
1.14.13.12
1.14.13.2
1.14.13.33
4-Hydroxy-benzoate
4-Methoxy-benzoate
1.14.99.15
3-Hydroxy-benzoate
1.14.13.23
1.14.13.24
2,5Dihydroxy-benzoate
Tyrosine metabolism
1.13.11.8
4-Carboxy-2-hydroxymuconate semialdehyde
2-Hydroxy-2-hydropyrone-4,6-dicarboxylate
1.1.1.312
2-Pyrone-4,6-dicarboxylate
3.1.1.57
4-Oxalomesaconate
5.3.2.8
4.2.1.83
4.1.3.17
4-Carboxy-2-hydroxy-cis,cis-muconate
4-Carboxy-4-hydroxy-2-oxoadipate
Oxaloacetate
Cytrate cycle
Aminobenzoate degradation
3.7.1.-
4-Carboxy-2-oxo-4-pentanoate
4.2.1.-
4-Hydroxy-4-methyl-2-oxoglutarate
4.1.3.17
Pyruvate
1.13.11.-
3,4-Dihydroxy-benzoate
Hydroxyquinol
1.13.11.37
3-Hydroxy-cis,cis-muconate
2-Maleylacetate
1.3.1.32
5.5.1.5
3-Carboxy-2,5-dihydro-5-oxofuran-2-acetate
1.13.11.3
β-Carboxy-muconate
5.5.1.2
γ-Carboxymuconolactone
4.1.1.44
4.1.1.63
Phenol
1.14.13.-
1.14.13.-
Benzene
1.14.12.3
cis-Dihydro-benzenediol
1.3.1.19
1.3.7.9
Benzoate
1.14.12.10
cis-1,2-Dihydroxy-cyclohexa-3,5-diene-1-carboxylate
1.3.1.25
Catechol
1.13.11.1
cis,cis-Muconate
5.5.1.1
(+)-Muconolactone
5.3.3.4
3-Oxoadipate-enol-lactone
3.1.1.24
3-Oxoadipate
2.8.3.6
3-Oxoadipyl-CoA
2.3.1.16
2.3.1.174
Succinyl-CoA
Cytrate cycle
1.13.11.2
4.1.1.46
3-Carboxy-2-hydroxy-muconatesemialdehyde
1.14.99.23
2,3-Dihydroxy-benzoate
1.13.11.14
4.1.1.-
2-Hydroxy-muconatesemialdehyde
3.7.1.9
2-Oxopent-4-enoate
4.2.1.80
4-Hydroxy-2-oxopentanoate
4.1.3.39
Acetaldehyde
1.2.1.10
Pyruvate
Glycolysis
1.2.1.85
4.1.1.77
2-Aminobenzene-sulfonate
1.14.12.14
3-Sulfocatechol
1.13.11.2
2-Hydroxy-muconate
5.3.2.6
γ-Oxalocrotonate
4.1.1.-
6.2.1.25
Cyclohexane-1-carboxylate
AliA
Cyclohexane-1-carboxyl-CoA
AliB
1.3.8.11
2-Hydroxycyclohexane-1-carboxyl-CoA
1.3.8.10
Cyclohex-1-ene-1-carboxyl-CoA
4.2.1.-
1.1.1.-
2-Ketocyclohexane-1-carboxyl-CoA
3.1.2.-
Pimeloyl-CoA
1.3.1.62
2,3-Didehydro-pimeloyl-CoA
4.2.1.-
Cyclohex-2,5-diene-1-carboxyl-CoA
Benzoyl-CoA
1.3.7.8
1.3.-.-
6-Hydroxycyclohex-1-ene-1-carbonyl-CoA
4.2.1.100
Cyclohex-1,5-diene-1-carboxyl-CoA
1.1.1.368
6-Ketoxycyclohex-1-ene-1-carboxyl-CoA
3.7.1.21
3-Hydroxy-pimeloyl-CoA
1.1.1.259
1.1.1.35
3-Keto-pimeloyl-CoA
2.3.1.16
Glutaryl-CoA
1.3.8.6
1.3.99.32
Glutaconyl-CoA
4.1.1.70
Crotonoyl-CoA
4.2.1.17
(S)-3-Hydroxy-butanoyl-CoA
1.1.1.157
Acetoacetyl-CoA
2.3.1.9
Acetyl-CoA
Cytrate cycle
Cyclohex-1,4-diene-1-carboxyl-CoA
00362 8/4/15
(c) Kanehisa Laboratories

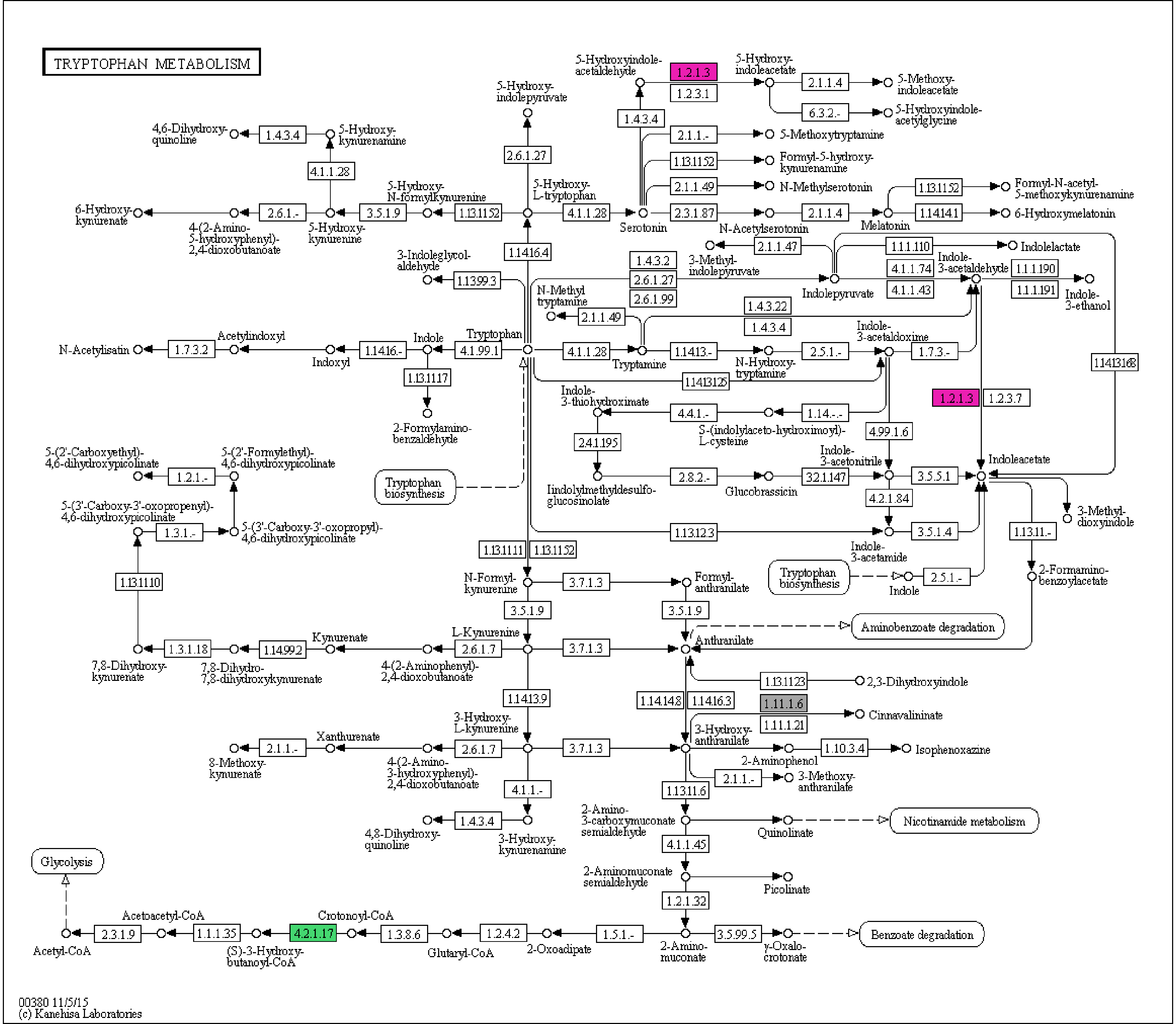

TRYPTOPHAN METABOLISM
5-Hydroxyindole-acetaldehyde
1.2.1.3
1.2.3.1
5-Hydroxy-indoleacetate
2.1.1.4
5-Methoxy-indoleacetate
6.3.2.-
5-Hydroxyindole-acetylglycine
1.4.3.4
2.1.1.-
5-Methoxytryptamine
1.13.11.52
Formyl-5-hydroxy-kynurenamine
2.1.1.49
N-Methylserotonin
2.3.1.87
2.1.4
1.13.11.52
Formyl-N-acetyl-5-methoxykynurenamine
1.14.14.1
6-Hydroxymelatonin
Serotonin
N-Acetylserotonin
Melatonin
5-Hydroxy-indolepyruvate
2.6.1.27
4,6-Dihydroxy-quinoline
1.4.3.4
5-Hydroxy-kynurenamine
4.1.1.28
5-Hydroxy-N-formylkynurenine
5-Hydroxy-L-tryptophan
4.1.1.28
6-Hydroxy-kynurenate
2.6.1.-
3.5.1.9
1.13.11.52
4-(2-Amino-5-hydroxyphenyl)-2,4-dioxobutanoate
5-Hydroxy-kynurenine
1.14.16.4
3-Indoleglycol-aldehyde
1.13.99.3
1.4.3.2
2.6.1.27
2.6.1.99
3-Methyl-indolepyruvate
2.1.1.47
1.1.1.110
Indolelactate
Indole-3-acetaldehyde
4.1.1.74
1.1.1.190
4.1.1.43
1.1.1.191
Indolepyruvate
Indole-3-ethanol
N-Methyl tryptamine
2.1.1.49
1.4.3.22
1.4.3.4
N-Acetylisatin
1.7.3.2
Acetylindoxyl
Indoxyl
1.14.16.-
Indole
Tryptophan
4.1.99.1
4.1.1.28
Tryptamine
1.14.13.-
N-Hydroxy-tryptamine
2.5.1.-
Indole-3-acetaldoxime
1.7.3.-
1.14.13.168
1.13.11.17
1.14.13.125
2-Formylamino-benzaldehyde
Indole-3-thiohydroximate
4.4.1.-
S-(indolylaceto-hydroximoyl)-L-cysteine
1.14.-.-
1.2.1.3
1.2.3.7
2.4.1.195
4.99.1.6
Indole-3-acetonitrile
Indoleacetate
5-(2'-Carboxyethyl)-4,6-dihydroxypicolinate
1.2.1.-
5-(2'-Formylethyl)-4,6-dihydroxypicolinate
Tryptophan biosynthesis
Indolylmethyldesulfo-glucosinolate
2.8.2.-
Glucobrassicin
3.2.1.147
3.5.5.1
4.2.1.84
5-(3'-Carboxy-3'-oxopropenyl)-4,6-dihydroxypicolinate
1.3.1.-
5-(3'-Carboxy-3'-oxopropyl)-4,6-dihydroxypicolinate
1.13.12.3
3.5.1.4
3-Methyl-dioxyindole
1.13.11.-
Indole-3-acetamide
1.13.11.11
1.13.11.52
1.13.11.10
N-Formyl-kynurenine
3.7.1.3
Formyl-anthranilate
Tryptophan biosynthesis
Indole
2.5.1.-
2-Formamino-benzoylacetate
3.5.1.9
3.5.1.9
Aminobenzoate degradation
7,8-Dihydroxy-kynurenate
1.3.1.18
7,8-Dihydro-7,8-dihydroxykynurenate
1.14.99.2
Kynurenate
4-(2-Aminophenyl)-2,4-dioxobutanoate
2.6.1.7
L-Kynurenine
3.7.1.3
Anthranilate
1.13.11.23
2,3-Dihydroxyindole
1.14.13.9
1.14.14.8
1.14.16.3
1.11.1.6
Cinnavalininate
1.11.1.21
3-Hydroxy-L-kynurenine
8-Methoxy-kynurenate
2.1.1.-
Xanthurenate
4-(2-Amino-3-hydroxyphenyl)-2,4-dioxobutanoate
2.6.1.7
3.7.1.3
3-Hydroxy-anthranilate
2-Aminophenol
1.10.3.4
Isophenoxazine
2.1.1.-
3-Methoxy-anthranilate
4.1.1.-
1.13.11.6
4,8-Dihydroxy-quinoline
1.4.3.4
3-Hydroxy-kynurenamine
2-Amino-3-carboxymuconate semialdehyde
Quinolinate
Nicotinamide metabolism
4.1.1.45
2-Aminomuconate semialdehyde
Picolinate
Glycolysis
1.2.1.32
Acetoacetyl-CoA
Crotonoyl-CoA
Acetyl-CoA
2.3.1.9
1.1.1.35
4.2.1.17
1.3.8.6
1.2.4.2
1.5.1.-
3.5.99.5
Benzoate degradation
(S)-3-Hydroxy-butanoyl-CoA
Glutaryl-CoA
2-Oxoadipate
2-Amino-muconate
γ-Oxalo-crotonate
00380 11/5/15
(c) Kanehisa Laboratories

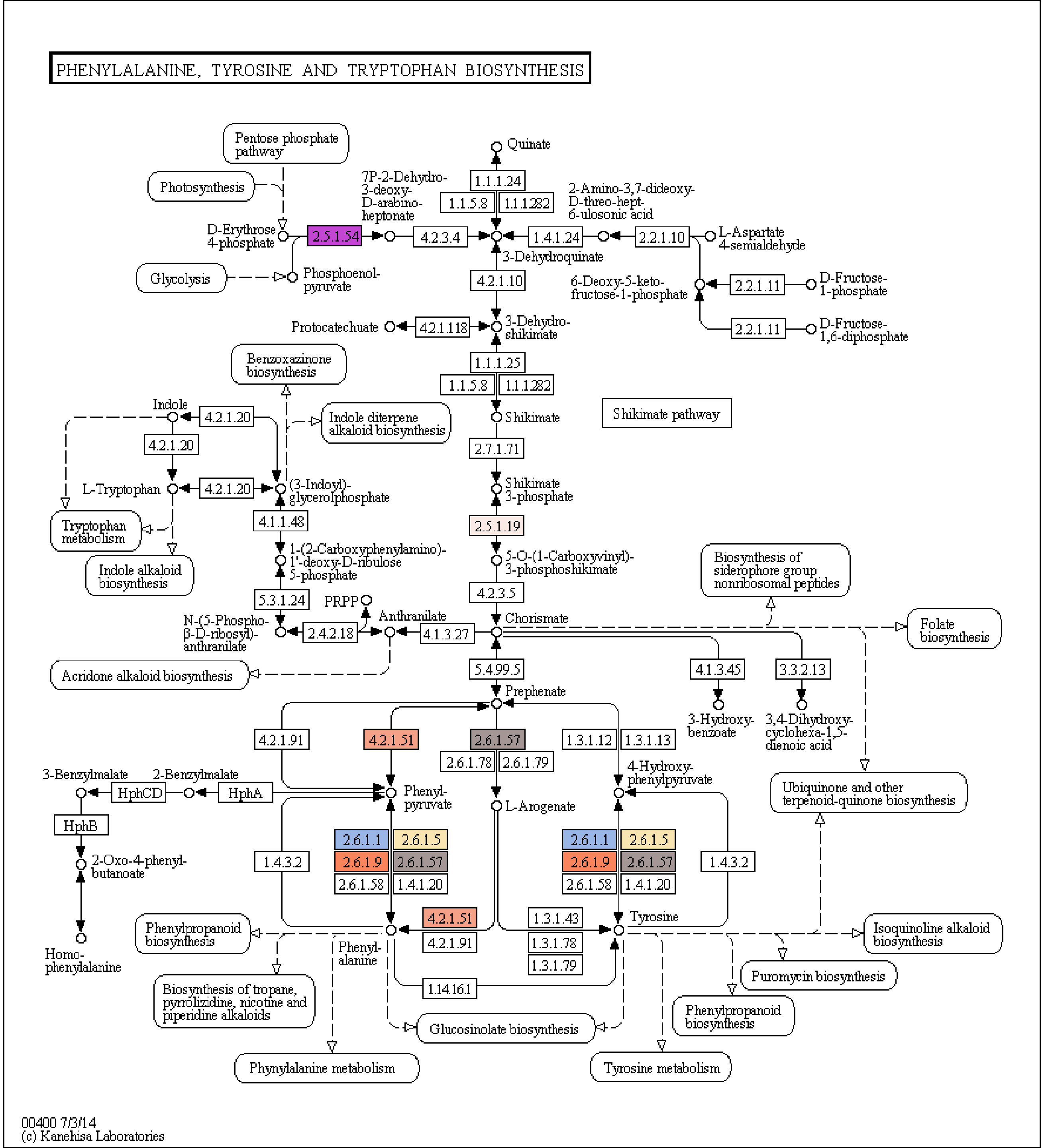
PHENYLALANINE, TYROSINE AND TRYPTOPHAN BIOSYNTHESIS
Pentose phosphate pathway
Photosynthesis
Glycolysis
D-Erythrose 4-phosphate
Phosphoenol-pyruvate
2.5.1.54
7P-2-Dehydro-3-deoxy-D-arabino-heptonate
4.2.3.4
Quinate
1.1.1.24
1.1.5.8
1.1.1.282
2-Amino-3,7-dideoxy-D-threo-hept-6-ulosonic acid
1.4.1.24
2.2.1.10
L-Aspartate 4-semialdehyde
3-Dehydroquinate
4.2.1.10
6-Deoxy-5-keto-fructose-1-phosphate
2.2.1.11
D-Fructose-1-phosphate
2.2.1.11
D-Fructose-1,6-diphosphate
Protocatechuate
4.2.1.118
3-Dehydro-shikimate
1.1.1.25
1.1.5.8
1.1.1.282
Shikimate
Shikimate pathway
2.7.1.71
Shikimate 3-phosphate
2.5.1.19
5-O-(1-Carboxyvinyl)-3-phosphoshikimate
4.2.3.5
Chorismate
Benzoxazinone biosynthesis
Indole diterpene alkaloid biosynthesis
Indole
4.2.1.20
4.2.1.20
L-Tryptophan
4.2.1.20
(3-Indoyl)-glycerolphosphate
Tryptophan metabolism
Indole alkaloid biosynthesis
4.1.1.48
1-(2-Carboxyphenylamino)-1'-deoxy-D-ribulose 5-phosphate
5.3.1.24
PRPP
Anthranilate
N-(5-Phospho-β-D-ribosyl)-anthranilate
2.4.2.18
4.1.3.27
Acridone alkaloid biosynthesis
Biosynthesis of siderophore group nonribosomal peptides
Folate biosynthesis
5.4.99.5
4.1.3.45
3.3.2.13
Prephenate
3-Hydroxy-benzoate
3,4-Dihydroxy-cyclohexa-1,5-dienoic acid
4.2.1.91
4.2.1.51
2.6.1.57
2.6.1.78
2.6.1.79
1.3.1.12
1.3.1.13
3-Benzylmalate
2-Benzylmalate
HphCD
HphA
HphB
Phenyl-pyruvate
L-Arogenate
4-Hydroxy-phenylpyruvate
Ubiquinone and other terpenoid-quinone biosynthesis
2-Oxo-4-phenyl-butanoate
1.4.3.2
2.6.1.1
2.6.1.5
2.6.1.9
2.6.1.57
2.6.1.58
1.4.1.20
2.6.1.1
2.6.1.5
2.6.1.9
2.6.1.57
2.6.1.58
1.4.1.20
1.4.3.2
Homo-phenylalanine
Phenylpropanoid biosynthesis
4.2.1.51
4.2.1.91
Phenyl-alanine
1.3.1.43
1.3.1.78
1.3.1.79
Tyrosine
Isoquinoline alkaloid biosynthesis
Puromycin biosynthesis
Biosynthesis of tropane, pyrrolizidine, nicotine and piperidine alkaloids
1.14.16.1
Phenylpropanoid biosynthesis
Glucosinolate biosynthesis
Phynylalanine metabolism
Tyrosine metabolism
00400 7/3/14
(c) Kanehisa Laboratories

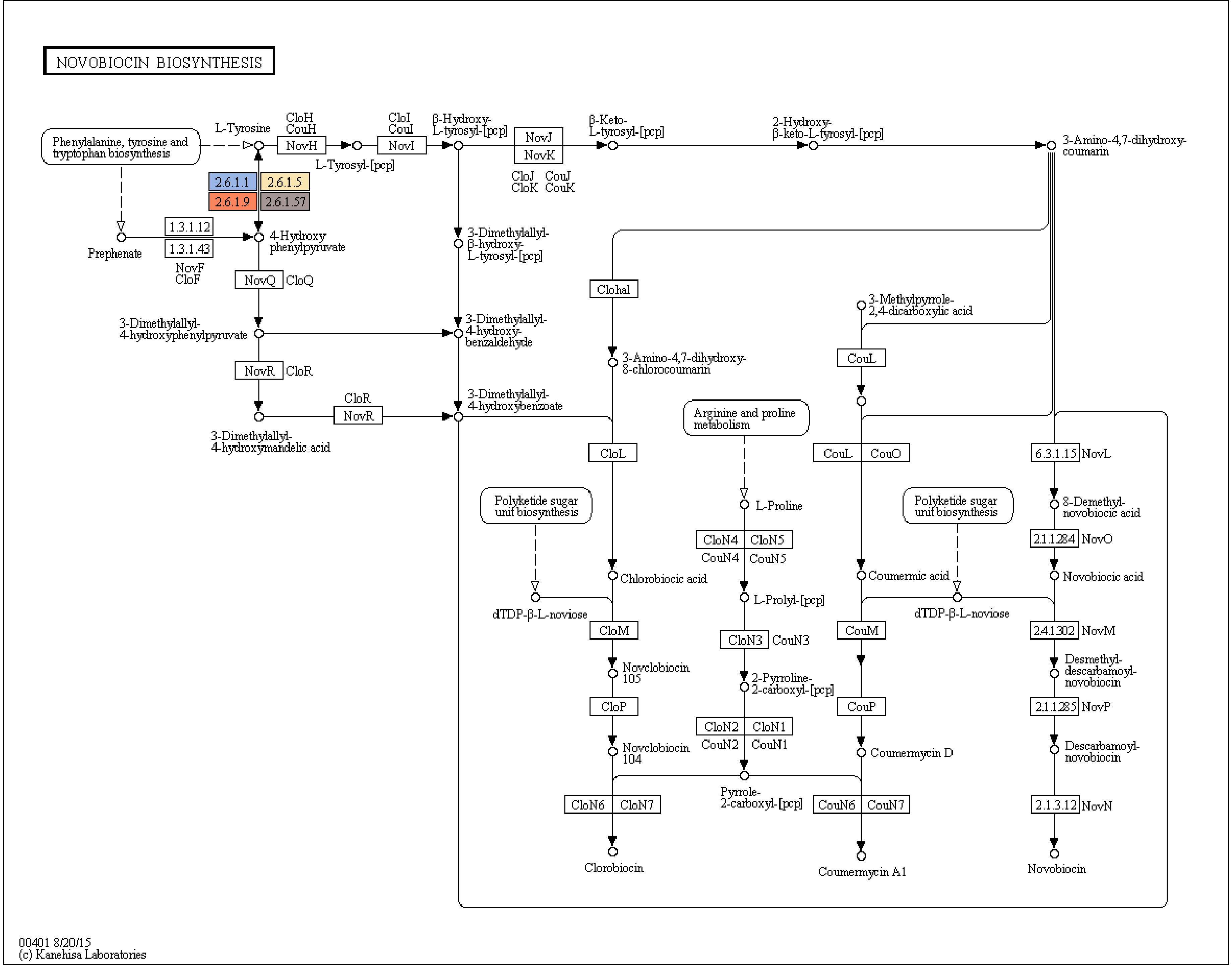
NOVOBIOCIN BIOSYNTHESIS
Phenylalanine, tyrosine and tryptophan biosynthesis
L-Tyrosine
CloH CouH
NovH
L-Tyrosyl-[pcp]
CloI CouI
NovI
β-Hydroxy-L-tyrosyl-[pcp]
NovJ
NovK
CloJ CouJ
CloK CouK
β-Keto-L-tyrosyl-[pcp]
2-Hydroxy-β-keto-L-tyrosyl-[pcp]
3-Amino-4,7-dihydroxy-coumarin
2.6.1.1
2.6.1.5
2.6.1.9
2.6.1.57
1.3.1.12
1.3.1.43
NovF CloF
Prephenate
4-Hydroxy phenylpyruvate
NovQ
CloQ
3-Dimethylallyl-β-hydroxy-L-tyrosyl-[pcp]
3-Dimethylallyl-4-hydroxyphenylpyruvate
3-Dimethylallyl-4-hydroxy-benzaldehyde
NovR
CloR
3-Dimethylallyl-4-hydroxybenzoate
3-Dimethylallyl-4-hydroxymandelic acid
Clohal
3-Amino-4,7-dihydroxy-8-chlorocoumarin
3-Methylpyrrole-2,4-dicarboxylic acid
CouL
Arginine and proline metabolism
CloL
CouL CouO
6.3.1.15 NovL
Polyketide sugar unit biosynthesis
L-Proline
CloN4 CloN5
CouN4 CouN5
8-Demethyl-novobiocic acid
2.1.1.284 NovO
Chlorobiocic acid
L-Prolyl-[pcp]
Coumermic acid
Novobiocic acid
dTDP-β-L-noviose
CloM
CloN3 CouN3
CouM
2.4.1.302 NovM
Novclobiocin 105
2-Pyrroline-2-carboxyl-[pcp]
Desmethyl-descarbamoyl-novobiocin
CloP
CouP
2.1.1.285 NovP
CloN2 CloN1
CouN2 CouN1
Novclobiocin 104
Coumermycin D
Descarbamoyl-novobiocin
Pyrrole-2-carboxyl-[pcp]
CloN6 CloN7
CouN6 CouN7
2.1.3.12 NovN
Clorobiocin
Coumermycin A1
Novobiocin
00401 8/20/15
(c) Kanehisa Laboratories

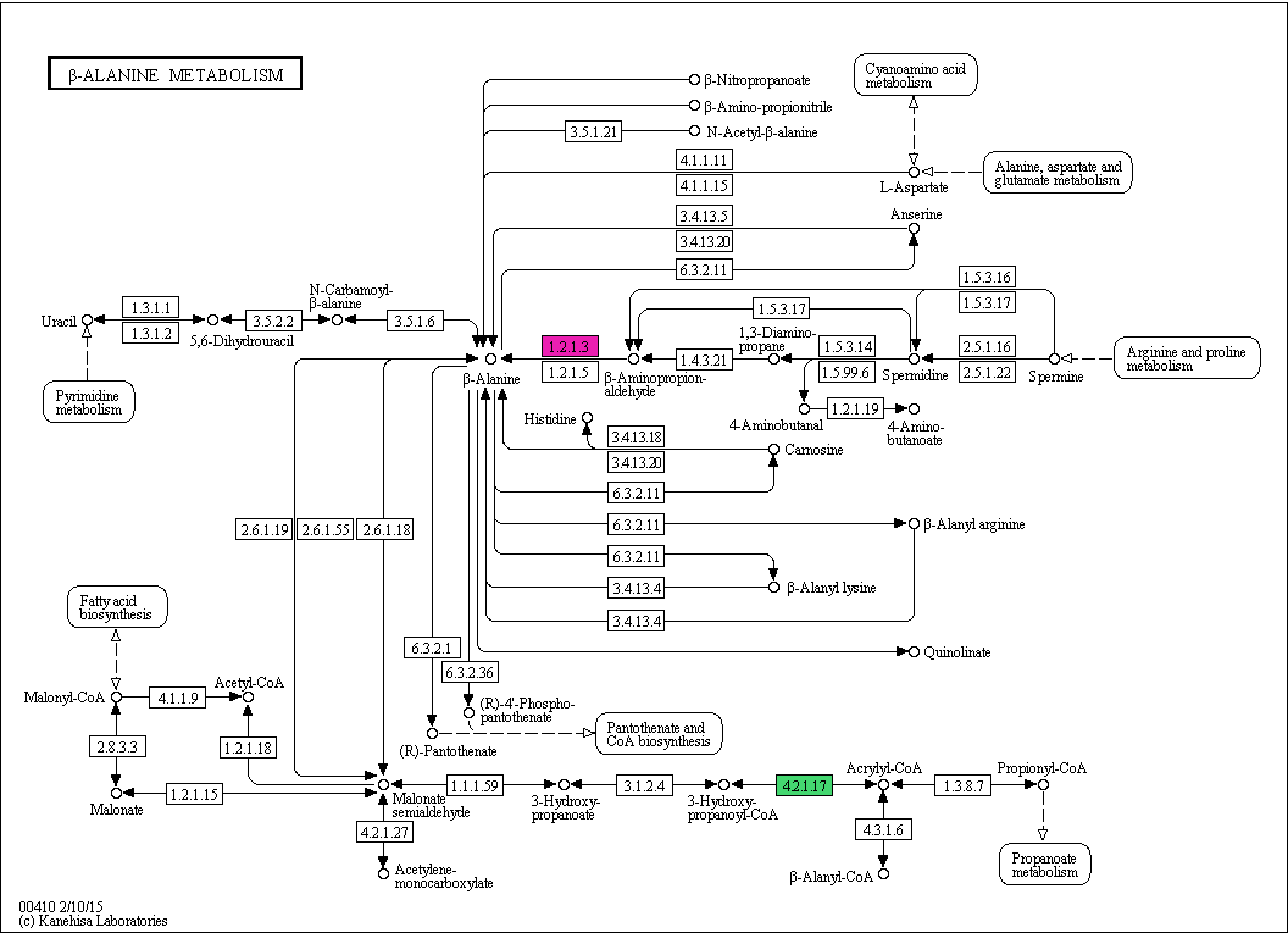
β-ALANINE METABOLISM
β-Nitropropanoate
β-Amino-propionitrile
N-Acetyl-β-alanine
3.5.1.21
Cyanoamino acid metabolism
4.1.1.11
4.1.1.15
L-Aspartate
Alanine, aspartate and glutamate metabolism
3.4.13.5
3.4.13.20
6.3.2.11
Anserine
1.5.3.16
1.5.3.17
1.5.3.17
Uracil
1.3.1.1
1.3.1.2
5,6-Dihydrouracil
3.5.2.2
N-Carbamoyl-β-alanine
3.5.1.6
1.2.1.3
1.2.1.5
β-Alanine
β-Aminopropion-aldehyde
1.4.3.21
1,3-Diamino-propane
1.5.3.14
1.5.99.6
Spermidine
2.5.1.16
2.5.1.22
Spermine
Arginine and proline metabolism
Pyrimidine metabolism
4-Aminobutanal
1.2.1.19
4-Amino-butanoate
Histidine
3.4.13.18
3.4.13.20
Carnosine
6.3.2.11
6.3.2.11
β-Alanyl arginine
6.3.2.11
3.4.13.4
β-Alanyl lysine
3.4.13.4
2.6.1.19
2.6.1.55
2.6.1.18
Quinolinate
Fatty acid biosynthesis
6.3.2.1
6.3.2.36
(R)-4'-Phospho-pantothenate
Malonyl-CoA
4.1.1.9
Acetyl-CoA
(R)-Pantothenate
Pantothenate and CoA biosynthesis
2.8.3.3
1.2.1.18
Malonate
1.2.1.15
Malonate semialdehyde
1.1.1.59
3-Hydroxy-propanoate
3.1.2.4
3-Hydroxy-propanoyl-CoA
4.2.1.17
Acrylyl-CoA
1.3.8.7
Propionyl-CoA
4.2.1.27
Acetylene-monocarboxylate
4.3.1.6
β-Alanyl-CoA
Propanoate metabolism
00410 2/10/15
(c) Kanehisa Laboratories

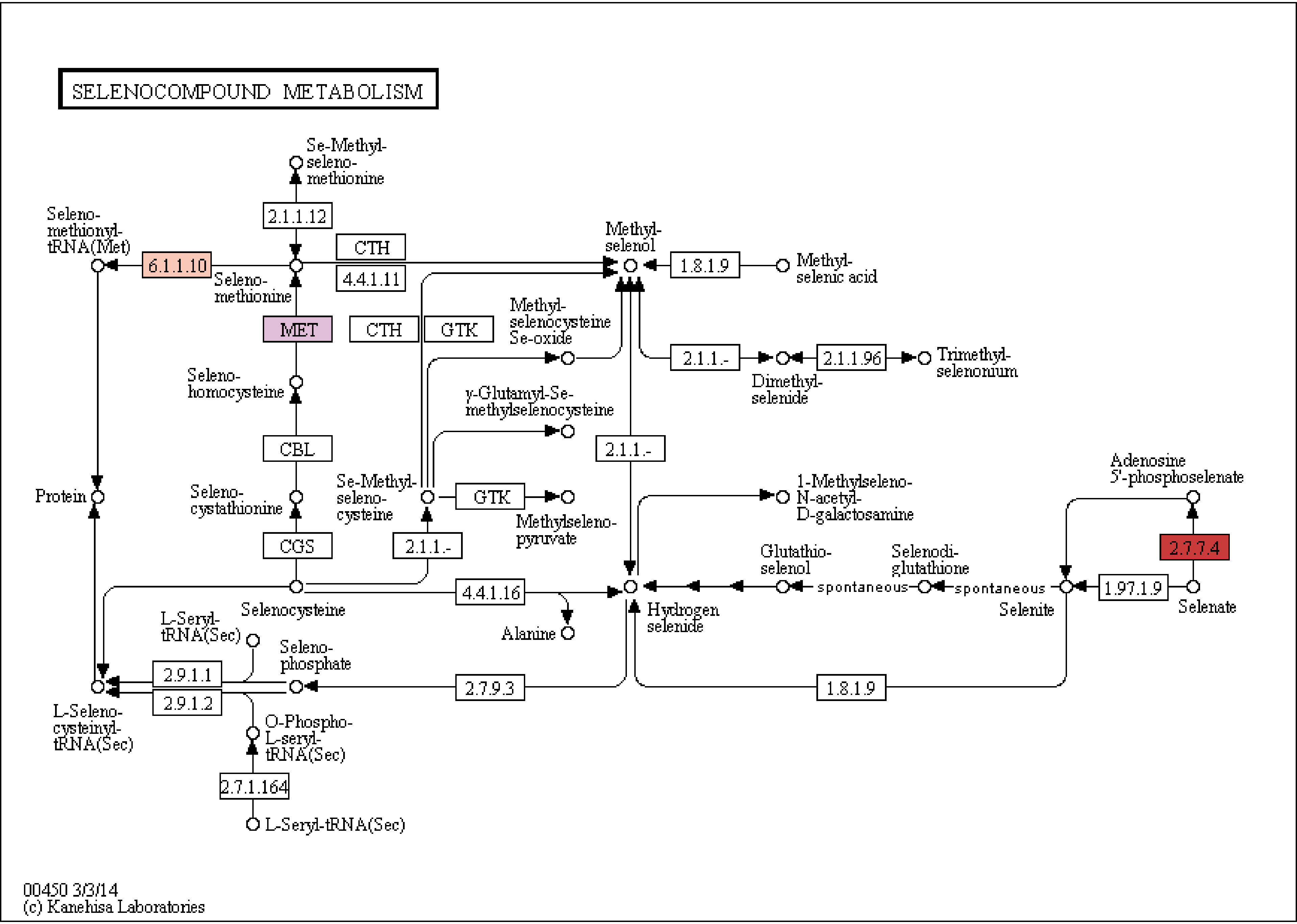
SELENOCOMPOUND METABOLISM
Se-Methyl-selenomethionine
2.1.1.12
Selenomethionyl-tRNA(Met)
6.1.1.10
Selenomethionine
CTH
4.4.1.11
Methylselenol
1.8.1.9
Methylselenic acid
MET
CTH
GTK
Methylselenocysteine Se-oxide
2.1.1.-
Dimethylselenide
2.1.1.96
Trimethylselenonium
Selenohomocysteine
γ-Glutamyl-Se-methylselenocysteine
CBL
2.1.1.-
Protein
Selenocystathionine
Se-Methylselenocysteine
GTK
Methylselenopyruvate
1-Methylseleno-N-acetyl-D-galactosamine
Adenosine 5'-phosphoselenate
CGS
2.1.1.-
2.7.7.4
Glutathioselenol
Selenodiglutathione
Selenocysteine
4.4.1.16
Alanine
Hydrogen selenide
spontaneous
spontaneous
Selenite
1.97.1.9
Selenate
L-Seryl-tRNA(Sec)
Selenophosphate
2.9.1.1
2.9.1.2
2.7.9.3
1.8.1.9
L-Selenocysteinyl-tRNA(Sec)
O-Phospho-L-seryl-tRNA(Sec)
2.7.1.164
L-Seryl-tRNA(Sec)
00450 3/3/14
(c) Kanehisa Laboratories

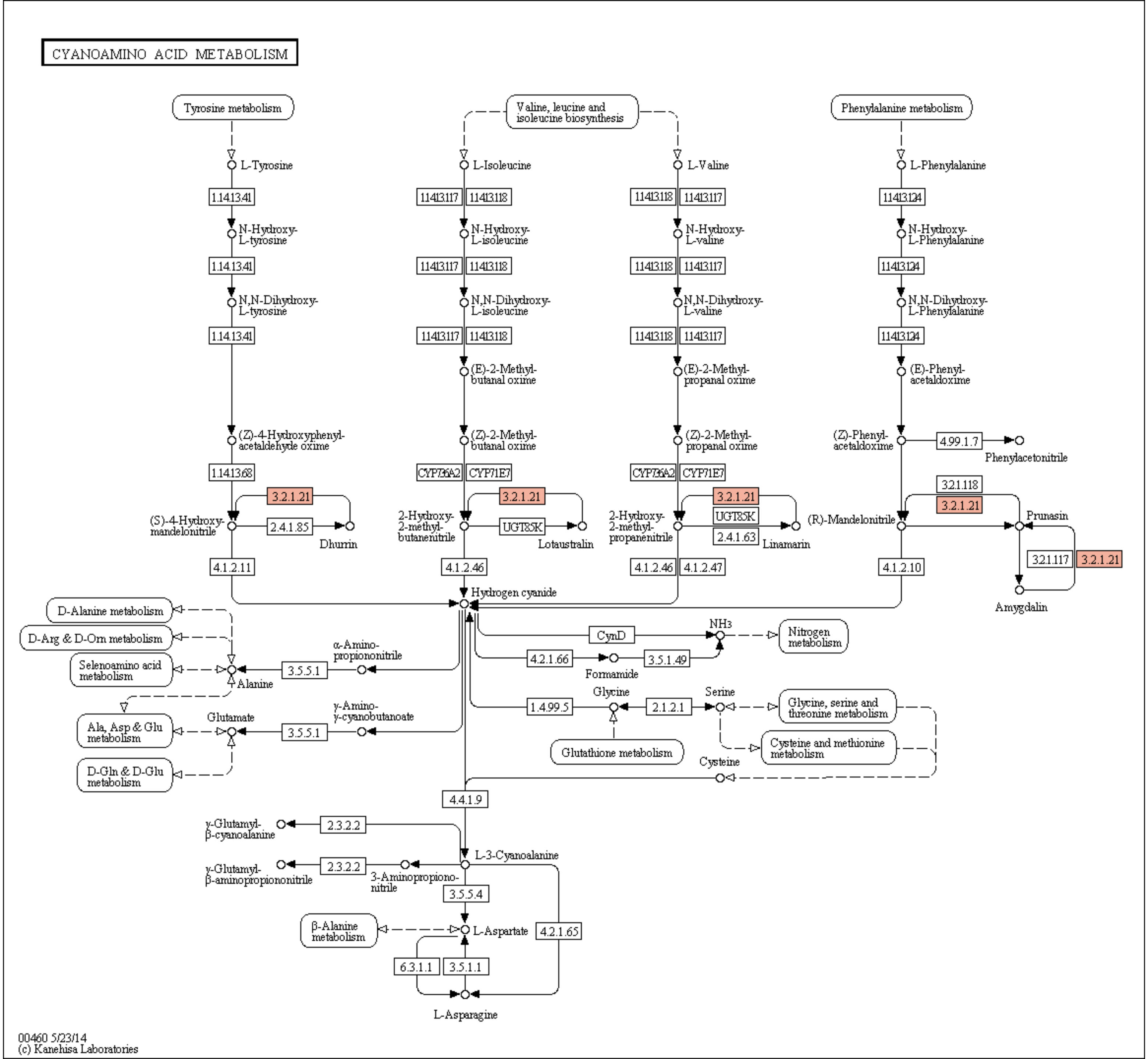
CYANOAMINO ACID METABOLISM
Tyrosine metabolism
Valine, leucine and isoleucine biosynthesis
Phenylalanine metabolism
L-Tyrosine
L-Isoleucine
L-Valine
L-Phenylalanine
1.14.13.41
1.14.13.117
1.14.13.118
1.14.13.124
N-Hydroxy-L-tyrosine
N-Hydroxy-L-isoleucine
N-Hydroxy-L-valine
N-Hydroxy-L-Phenylalanine
N,N-Dihydroxy-L-tyrosine
N,N-Dihydroxy-L-isoleucine
N,N-Dihydroxy-L-valine
N,N-Dihydroxy-L-Phenylalanine
(E)-2-Methyl-butanal oxime
(E)-2-Methyl-propanal oxime
(E)-Phenyl-acetaldoxime
(Z)-4-Hydroxyphenyl-acetaldehyde oxime
(Z)-2-Methyl-butanal oxime
(Z)-2-Methyl-propanal oxime
(Z)-Phenyl-acetaldoxime
4.99.1.7
Phenylacetonitrile
1.14.13.68
CYP736A2
CYP71E7
3.2.1.21
3.2.1.118
3.2.1.117
2.4.1.85
UGT85K
2.4.1.63
(S)-4-Hydroxy-mandelonitrile
2-Hydroxy-2-methyl-butanenitrile
2-Hydroxy-2-methyl-propanenitrile
(R)-Mandelonitrile
Dhurrin
Lotaustralin
Linamarin
Prunasin
Amygdalin
4.1.2.11
4.1.2.46
4.1.2.47
4.1.2.10
Hydrogen cyanide
D-Alanine metabolism
D-Arg & D-Orn metabolism
Selenoamino acid metabolism
Alanine
3.5.5.1
α-Amino-propiononitrile
CynD
NH3
Nitrogen metabolism
4.2.1.66
Formamide
3.5.1.49
Ala, Asp & Glu metabolism
D-Gln & D-Glu metabolism
Glutamate
γ-Amino-γ-cyanobutanoate
1.4.99.5
Glycine
2.1.2.1
Serine
Glycine, serine and threonine metabolism
Cysteine and methionine metabolism
Glutathione metabolism
Cysteine
4.4.1.9
γ-Glutamyl-β-cyanoalanine
2.3.2.2
L-3-Cyanoalanine
γ-Glutamyl-β-aminopropiononitrile
3-Aminopropiono-nitrile
3.5.5.4
β-Alanine metabolism
L-Aspartate
4.2.1.65
6.3.1.1
3.5.1.1
L-Asparagine
00460 5/23/14
(c) Kanehisa Laboratories

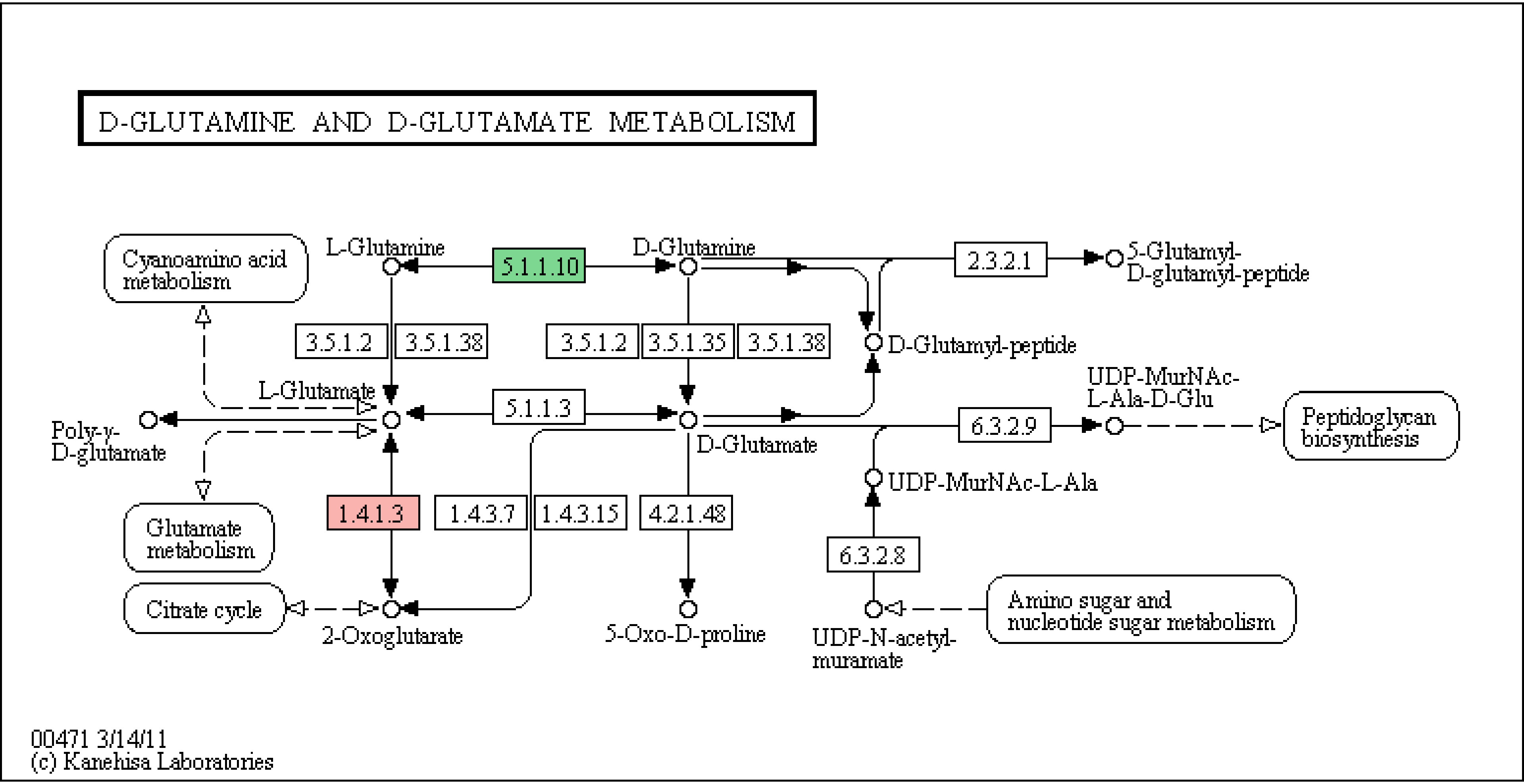
D-GLUTAMINE AND D-GLUTAMATE METABOLISM
Cyanoamino acid metabolism
L-Glutamine
5.1.1.10
D-Glutamine
2.3.2.1
5-Glutamyl-D-glutamyl-peptide
3.5.1.2
3.5.1.38
3.5.1.2
3.5.1.35
3.5.1.38
D-Glutamyl-peptide
L-Glutamate
5.1.1.3
UDP-MurNAc-L-Ala-D-Glu
Poly-γ-D-glutamate
D-Glutamate
6.3.2.9
Peptidoglycan biosynthesis
UDP-MurNAc-L-Ala
1.4.1.3
1.4.3.7
1.4.3.15
4.2.1.48
Glutamate metabolism
6.3.2.8
Citrate cycle
2-Oxoglutarate
5-Oxo-D-proline
UDP-N-acetyl-muramate
Amino sugar and nucleotide sugar metabolism
00471 3/14/11
(c) Kanehisa Laboratories

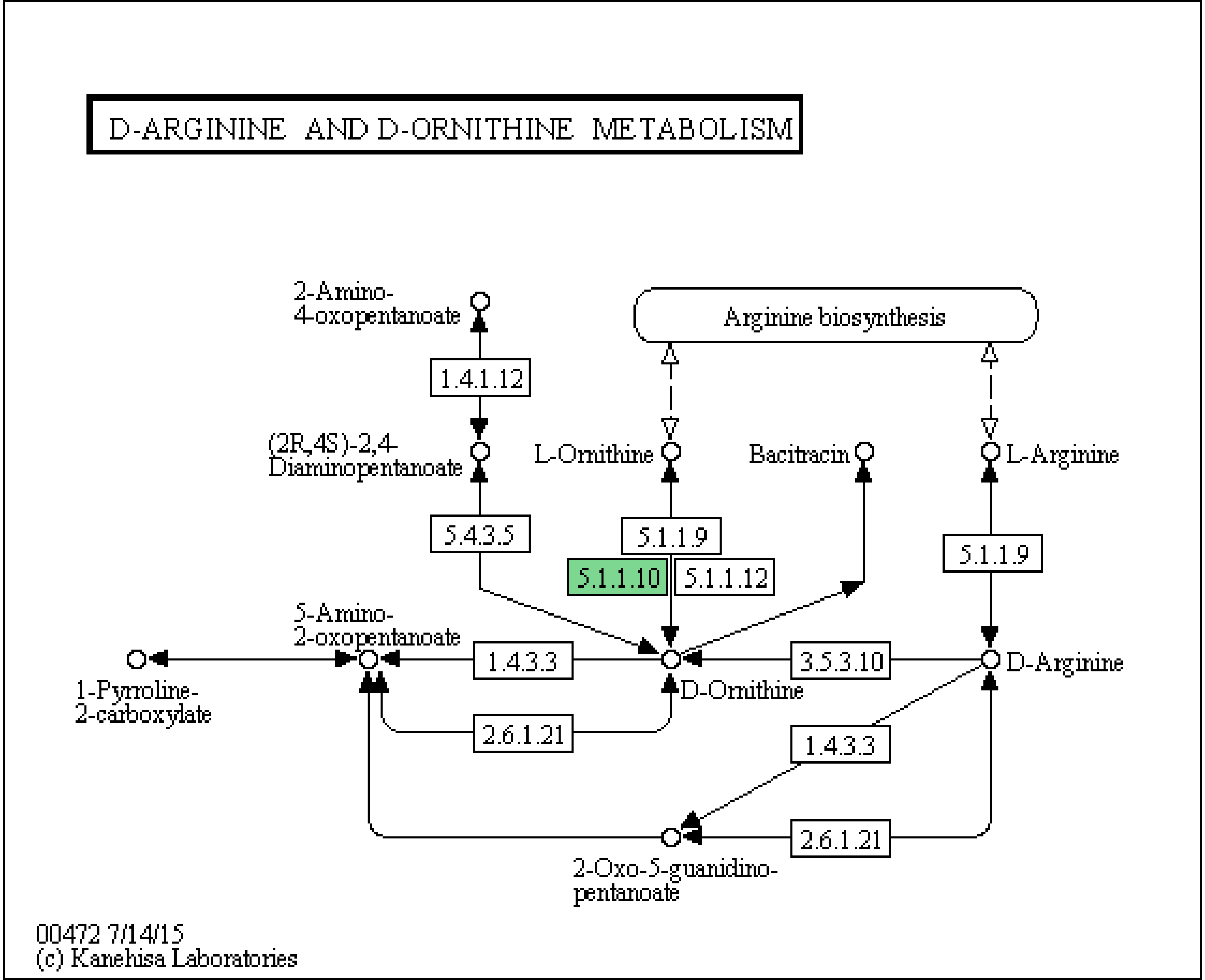

D-ARGININE AND D-ORNITHINE METABOLISM
2-Amino-
4-oxopentanoate
1.4.1.12
(2R,4S)-2,4-
Diaminopentanoate
5.4.3.5
Arginine biosynthesis
L-Ornithine
Bacitracin
L-Arginine
5.1.1.9
5.1.1.10
5.1.1.12
5.1.1.9
5-Amino-
2-oxopentanoate
1.4.3.3
3.5.3.10
D-Arginine
D-Ornithine
1-Pyrroline-
2-carboxylate
2.6.1.21
1.4.3.3
2.6.1.21
2-Oxo-5-guanidino-
pentanoate
00472 7/14/15
(c) Kanehisa Laboratories

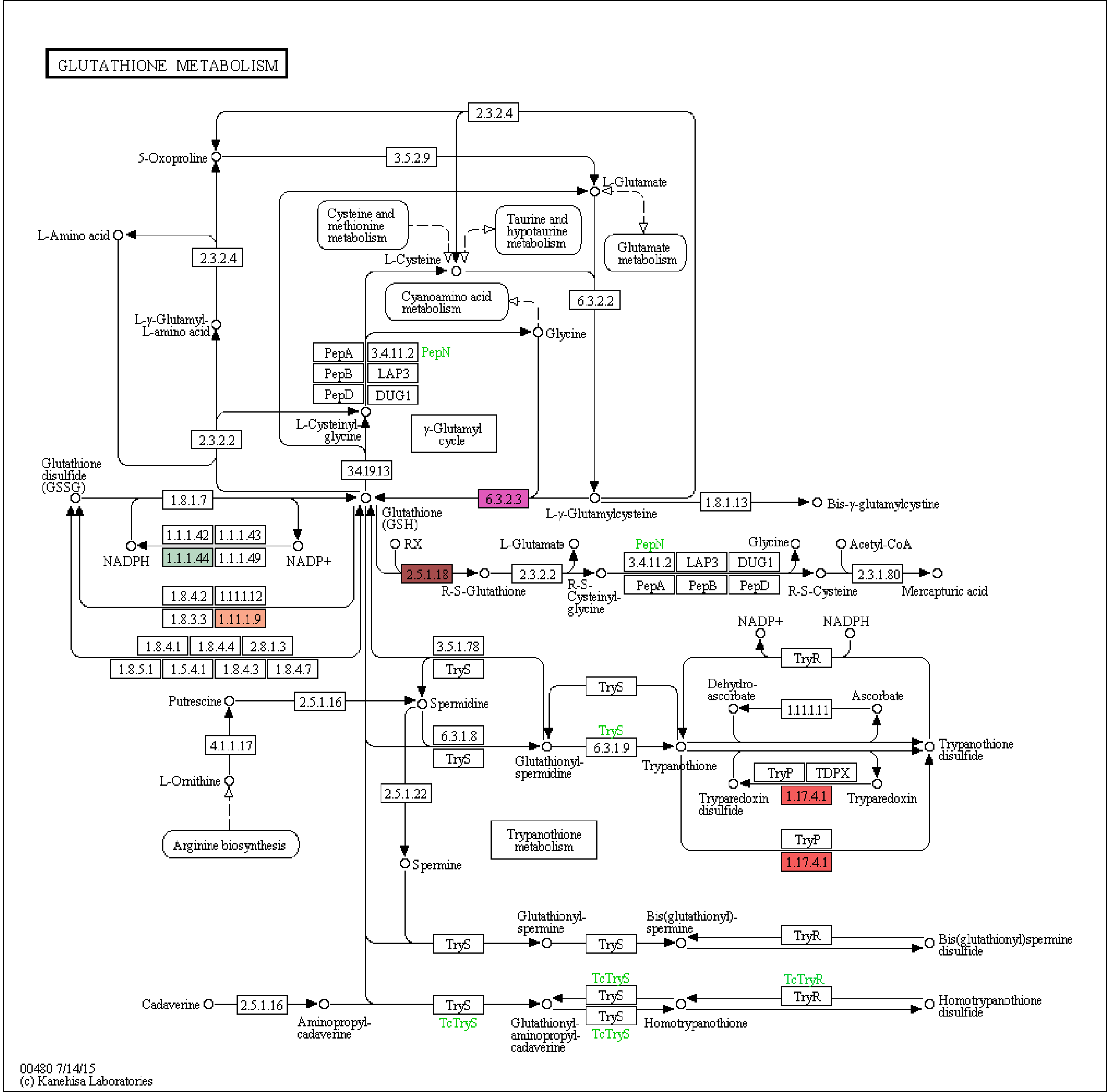
GLUTATHIONE METABOLISM
2.3.2.4
5-Oxoproline
3.5.2.9
L-Glutamate
Cysteine and methionine metabolism
Taurine and hypotaurine metabolism
Glutamate metabolism
L-Amino acid
2.3.2.4
L-Cysteine
Cyanoamino acid metabolism
6.3.2.2
L-γ-Glutamyl-L-amino acid
Glycine
PepA
3.4.11.2
PepN
PepB
LAP3
PepD
DUG1
L-Cysteinyl-glycine
γ-Glutamyl cycle
2.3.2.2
Glutathione disulfide (GSSG)
3.4.19.13
1.8.1.7
6.3.2.3
L-γ-Glutamylcysteine
1.8.1.13
Bis-γ-glutamylcystine
Glutathione (GSH)
1.1.1.42
1.1.1.43
1.1.1.44
1.1.1.49
NADPH
NADP+
RX
2.5.1.18
R-S-Glutathione
L-Glutamate
2.3.2.2
R-S-Cysteinyl-glycine
PepN
3.4.11.2
LAP3
DUG1
PepA
PepB
PepD
Glycine
Acetyl-CoA
R-S-Cysteine
2.3.1.80
Mercapturic acid
1.8.4.2
1.11.1.12
1.8.3.3
1.11.1.9
1.8.4.1
1.8.4.4
2.8.1.3
1.8.5.1
1.5.4.1
1.8.4.3
1.8.4.7
3.5.1.78
TryS
NADP+
NADPH
TryR
Putrescine
2.5.1.16
Spermidine
TryS
Dehydro-ascorbate
1.11.1.11
Ascorbate
4.1.1.17
6.3.1.8
TryS
Glutathionyl-spermidine
TryS
6.3.1.9
Trypanothione
Trypanothione disulfide
L-Ornithine
TryP
TDPX
Tryparedoxin disulfide
1.17.4.1
Tryparedoxin
2.5.1.22
Arginine biosynthesis
Trypanothione metabolism
TryP
1.17.4.1
Spermine
Glutathionyl-spermine
Bis(glutathionyl)-spermine
TryS
TryS
TryR
Bis(glutathionyl)spermine disulfide
TcTryS
TcTryR
Cadaverine
2.5.1.16
TryS
TryS
TryR
Homotrypanothione disulfide
TryS
Aminopropyl-cadaverine
TcTryS
Glutathionyl-aminopropyl-cadaverine
Homotrypanothione
TcTryS
00480 7/14/15
(c) Kanehisa Laboratories

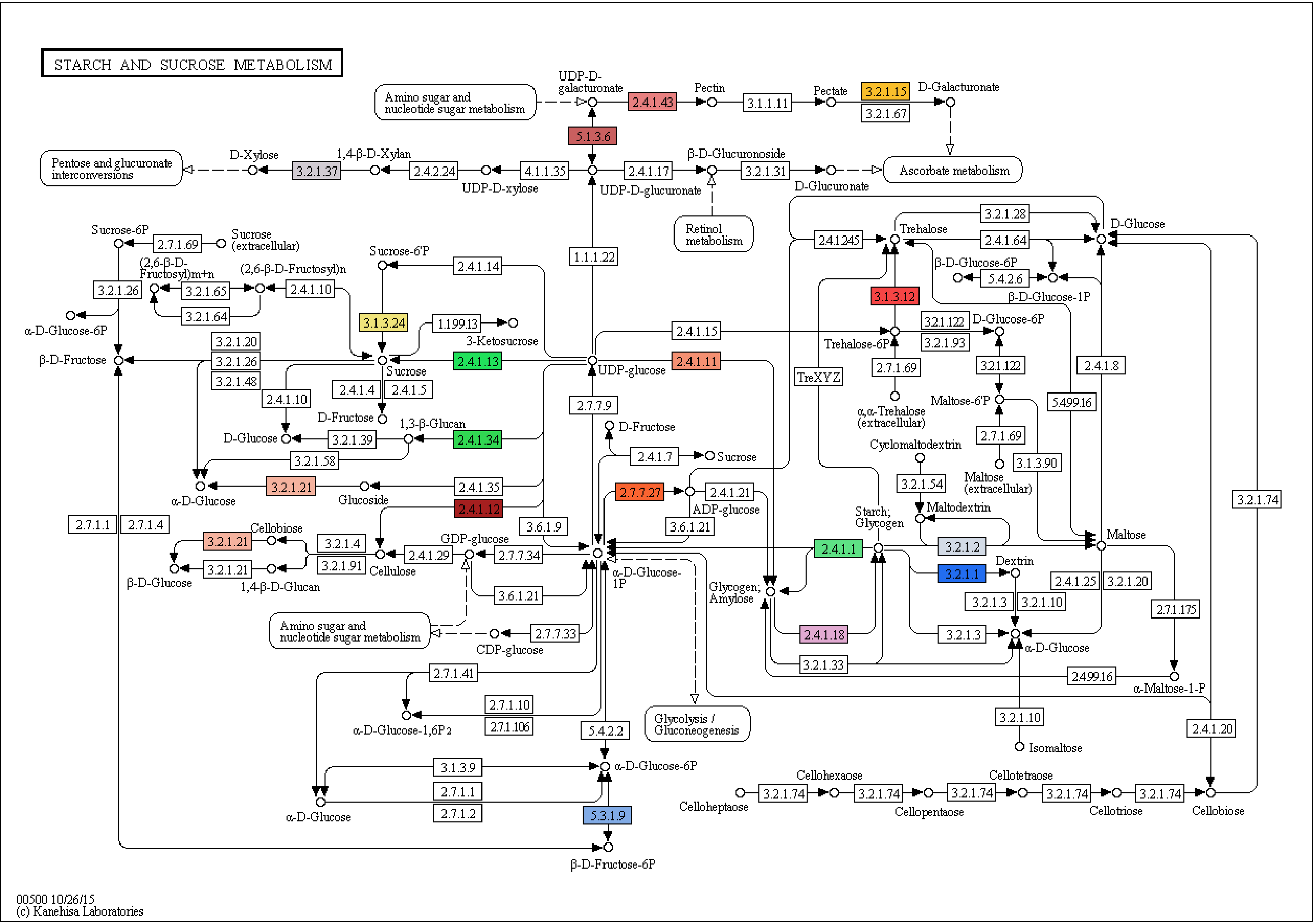
STARCH AND SUCROSE METABOLISM
Amino sugar and nucleotide sugar metabolism
UDP-D-galacturonate
2.4.1.43
Pectin
3.1.1.11
Pectate
3.2.1.15
3.2.1.67
D-Galacturonate
5.1.3.6
Pentose and glucuronate interconversions
D-Xylose
3.2.1.37
1,4-β-D-Xylan
2.4.2.24
UDP-D-xylose
4.1.1.35
2.4.1.17
UDP-D-glucuronate
β-D-Glucuronoside
3.2.1.31
D-Glucuronate
Ascorbate metabolism
Retinol metabolism
1.1.1.22
Sucrose-6P
2.7.1.69
Sucrose (extracellular)
(2,6-β-D-Fructosyl)m+n
3.2.1.65
(2,6-β-D-Fructosyl)n
2.4.1.10
3.2.1.26
3.2.1.64
α-D-Glucose-6P
β-D-Fructose
Sucrose-6'P
2.4.1.14
3.1.3.24
1.1.99.13
3-Ketosucrose
3.2.1.20
3.2.1.26
3.2.1.48
Sucrose
2.4.1.13
UDP-glucose
2.4.1.11
2.4.1.15
2.4.1.10
2.4.1.4
2.4.1.5
D-Fructose
D-Glucose
3.2.1.39
1,3-β-Glucan
2.4.1.34
3.2.1.58
α-D-Glucose
3.2.1.21
Glucoside
2.4.1.35
2.4.1.12
2.7.1.1
2.7.1.4
Cellobiose
3.2.1.21
3.2.1.21
3.2.1.4
3.2.1.91
β-D-Glucose
1,4-β-D-Glucan
Cellulose
2.4.1.29
GDP-glucose
2.7.7.34
3.6.1.9
3.6.1.21
Amino sugar and nucleotide sugar metabolism
CDP-glucose
2.7.7.33
2.7.7.9
D-Fructose
2.4.1.7
Sucrose
2.7.7.27
ADP-glucose
2.4.1.21
3.6.1.21
α-D-Glucose-1P
2.7.1.41
2.7.1.10
2.7.1.106
α-D-Glucose-1,6P2
5.4.2.2
α-D-Glucose-6P
3.1.3.9
2.7.1.1
2.7.1.2
α-D-Glucose
5.3.1.9
β-D-Fructose-6P
Glycolysis / Gluconeogenesis
3.2.1.28
Trehalose
2.4.1.245
2.4.1.64
D-Glucose
β-D-Glucose-6P
5.4.2.6
β-D-Glucose-1P
3.1.3.12
3.2.1.122
D-Glucose-6P
Trehalose-6P
3.2.1.93
TreXYZ
2.7.1.69
3.2.1.122
α,α-Trehalose (extracellular)
Maltose-6'P
2.7.1.69
2.4.1.8
5.4.99.16
Cyclomaltodextrin
3.1.3.90
Maltose (extracellular)
3.2.1.54
Maltodextrin
Starch; Glycogen
3.2.1.2
Maltose
2.4.1.1
Dextrin
3.2.1.1
Glycogen; Amylose
2.4.1.25
3.2.1.20
2.7.1.175
3.2.1.3
3.2.1.10
2.4.1.18
3.2.1.3
α-D-Glucose
3.2.1.33
2.4.99.16
α-Maltose-1-P
3.2.1.10
Isomaltose
3.2.1.74
2.4.1.20
Cellohexaose
Cellotetraose
3.2.1.74
3.2.1.74
3.2.1.74
3.2.1.74
3.2.1.74
Celloheptaose
Cellopentaose
Cellotriose
Cellobiose
00500 10/26/15
(c) Kanehisa Laboratories

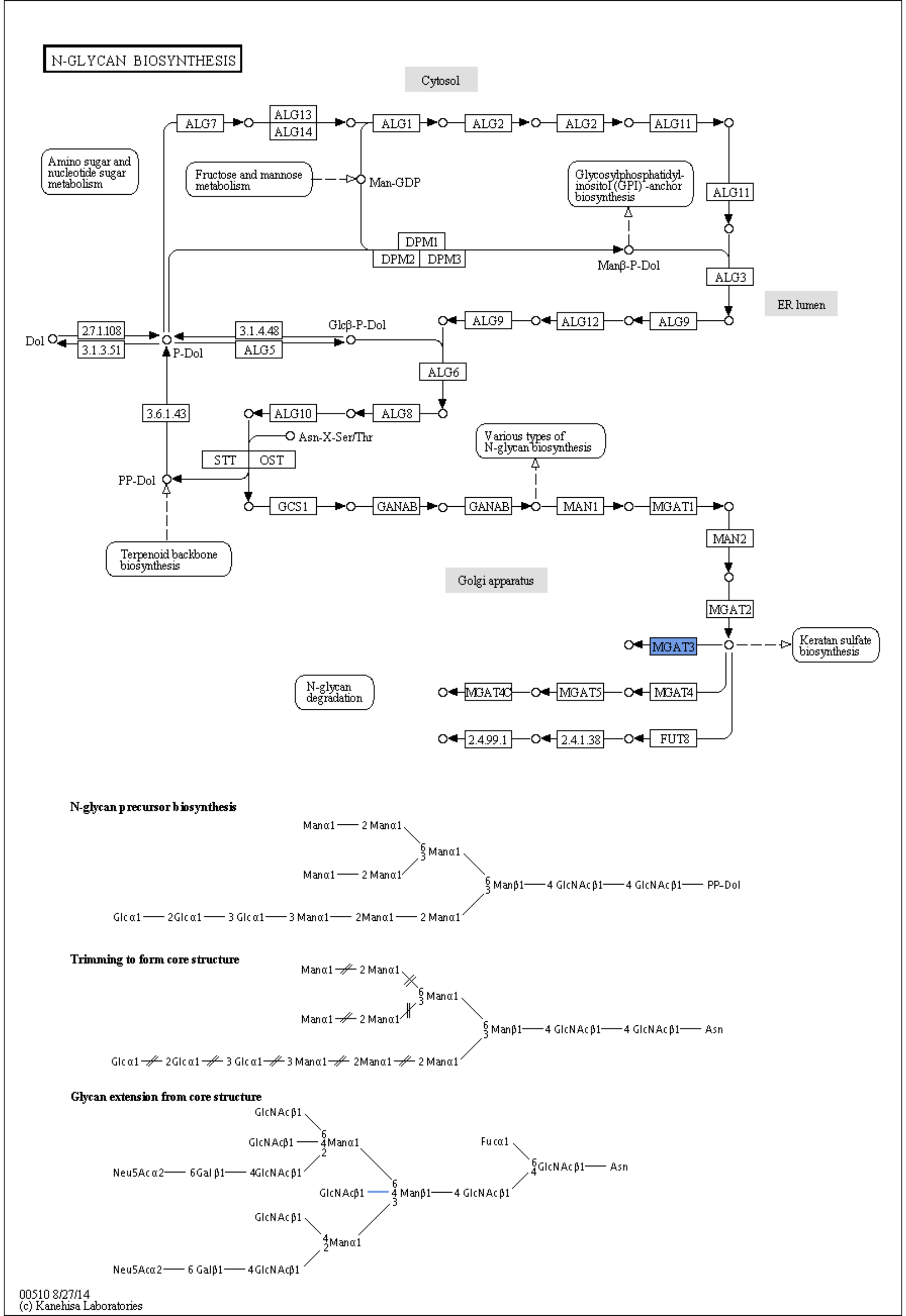
N-GLYCAN BIOSYNTHESIS
Cytosol
ER lumen
Golgi apparatus
ALG7
ALG13
ALG14
ALG1
ALG2
ALG2
ALG11
ALG11
ALG3
ALG9
ALG12
ALG9
ALG6
ALG8
ALG10
DPM1
DPM2
DPM3
Manβ-P-Dol
Man-GDP
Amino sugar and nucleotide sugar metabolism
Fructose and mannose metabolism
Glycosylphosphatidyl-inositol (GPI) -anchor biosynthesis
Dol
2.7.1.108
3.1.3.51
P-Dol
3.1.4.48
ALG5
Glcβ-P-Dol
3.6.1.43
Asn-X-Ser/Thr
STT
OST
PP-Dol
Terpenoid backbone biosynthesis
Various types of N-glycan biosynthesis
GCS1
GANAB
GANAB
MAN1
MGAT1
MAN2
MGAT2
MGAT3
Keratan sulfate biosynthesis
N-glycan degradation
MGAT4C
MGAT5
MGAT4
2.4.99.1
2.4.1.38
FUT8
N-glycan precursor biosynthesis
Manα1 — 2 Manα1
Manα1 — 2 Manα1
6 3 Manα1
6 3 Manβ1 — 4 GlcNAcβ1 — 4 GlcNAcβ1 — PP-Dol
Glcα1 — 2Glcα1 — 3 Glcα1 — 3 Manα1 — 2Manα1 — 2 Manα1
Trimming to form core structure
Manα1 — 2 Manα1
Manα1 — 2 Manα1
6 3 Manα1
6 3 Manβ1 — 4 GlcNAcβ1 — 4 GlcNAcβ1 — Asn
Glcα1 — 2Glcα1 — 3 Glcα1 — 3 Manα1 — 2Manα1 — 2 Manα1
Glycan extension from core structure
GlcNAcβ1
GlcNAcβ1 — 6 4 2 Manα1
Neu5Acα2 — 6Galβ1 — 4GlcNAcβ1
Fucα1
6 4 GlcNAcβ1 — Asn
GlcNAcβ1 — 6 4 3 Manβ1 — 4 GlcNAcβ1
GlcNAcβ1
4 2 Manα1
Neu5Acα2 — 6 Galβ1 — 4GlcNAcβ1
00510 8/27/14
(c) Kanehisa Laboratories

OTHER GLYCAN DEGRADATION

N-glycan

N-Glycan biosynthesis

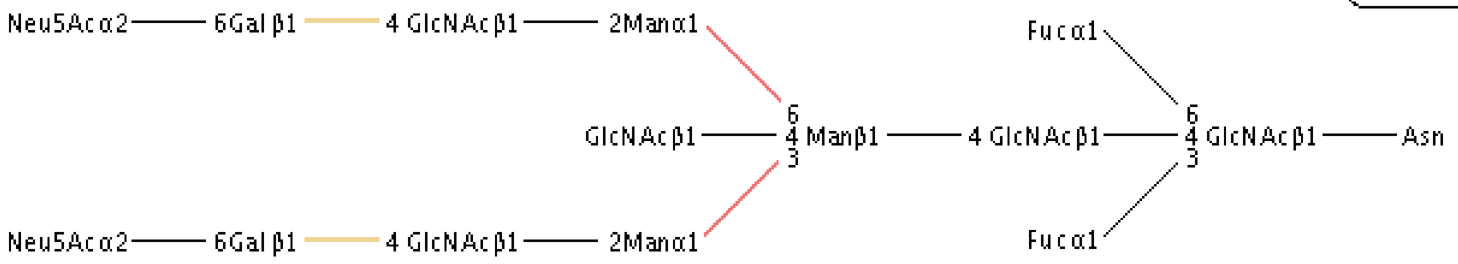

Ganglioside

Glycosphingolipid biosynthesis
- ganglio series

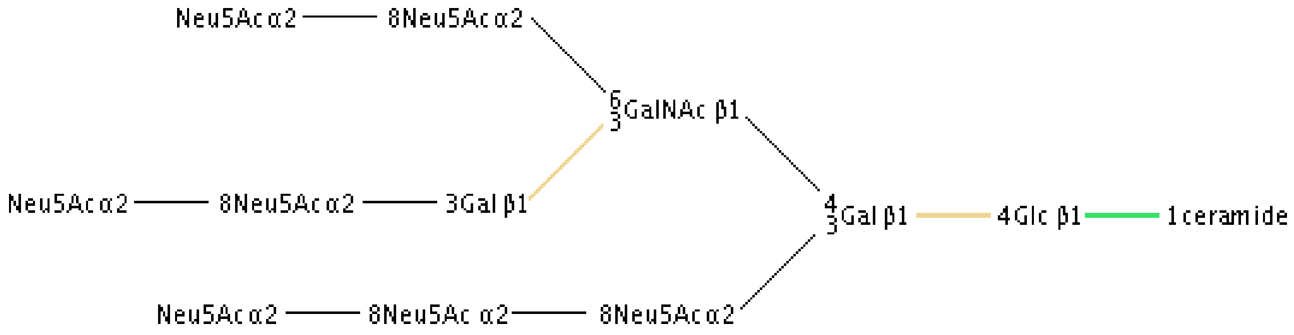

00511 6/3/13
(c) Kanehisa Laboratories

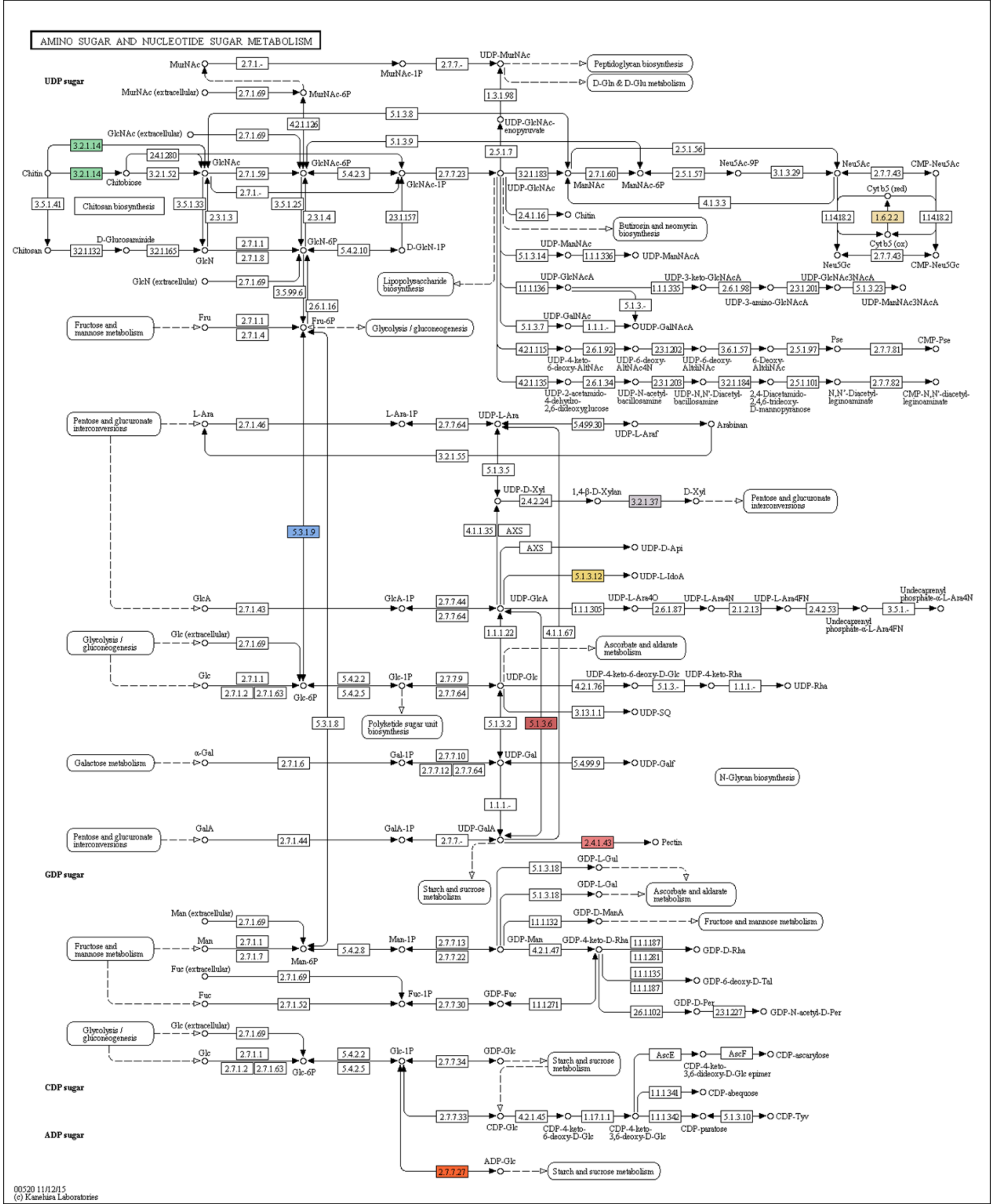
AMINO SUGAR AND NUCLEOTIDE SUGAR METABOLISM
UDP sugar
GDP sugar
CDP sugar
ADP sugar
MurNAc
MurNAc (extracellular)
MurNAc-1P
MurNAc-6P
UDP-MurNAc
Peptidoglycan biosynthesis
D-Gln & D-Glu metabolism
UDP-GlcNAc-enopyruvate
GlcNAc (extracellular)
GlcNAc
GlcNAc-6P
GlcNAc-1P
UDP-GlcNAc
Chitin
Chitobiose
Chitosan biosynthesis
Chitosan
D-Glucosaminide
GlcN
GlcN-6P
D-GlcN-1P
GlcN (extracellular)
ManNAc
ManNAc-6P
Neu5Ac-9P
Neu5Ac
CMP-Neu5Ac
Cyt b5 (red)
Cyt b5 (ox)
Neu5Gc
CMP-Neu5Gc
Butirosin and neomycin biosynthesis
UDP-ManNAc
UDP-ManNAcA
UDP-GlcNAcA
UDP-3-keto-GlcNAcA
UDP-GlcNAc3NAcA
UDP-3-amino-GlcNAcA
UDP-ManNAc3NAcA
UDP-GalNAc
UDP-GalNAcA
Lipopolysaccharide biosynthesis
Fructose and mannose metabolism
Fru
Fru-6P
Glycolysis / gluconeogenesis
Pse
CMP-Pse
UDP-4-keto-6-deoxy-AltNAc
UDP-6-deoxy-AltNAc4N
UDP-6-deoxy-AltdiNAc
6-Deoxy-AltdiNAc
UDP-2-acetamido-4-dehydro-2,6-dideoxyglucose
UDP-N-acetyl-bacillosamine
UDP-N,N'-Diacetyl-bacillosamine
2,4-Diacetamido-2,4,6-trideoxy-D-mannopyranose
N,N'-Diacetyl-legionaminate
CMP-N,N'-diacetyl-legionaminate
Pentose and glucuronate interconversions
L-Ara
L-Ara-1P
UDP-L-Ara
UDP-L-Araf
Arabinan
UDP-D-Xyl
1,4-β-D-Xylan
D-Xyl
UDP-D-Api
UDP-L-IdoA
GlcA
GlcA-1P
UDP-GlcA
UDP-L-Ara4O
UDP-L-Ara4N
UDP-L-Ara4FN
Undecaprenyl phosphate-α-L-Ara4FN
Undecaprenyl phosphate-α-L-Ara4N
Glc (extracellular)
Ascorbate and aldarate metabolism
Glc
Glc-6P
Glc-1P
UDP-Glc
UDP-4-keto-6-deoxy-D-Glc
UDP-4-keto-Rha
UDP-Rha
UDP-SQ
Polyketide sugar unit biosynthesis
Galactose metabolism
α-Gal
Gal-1P
UDP-Gal
UDP-Galf
N-Glycan biosynthesis
GalA
GalA-1P
UDP-GalA
Pectin
Starch and sucrose metabolism
GDP-L-Gul
GDP-L-Gal
GDP-D-ManA
Man (extracellular)
Man
Man-6P
Man-1P
GDP-Man
GDP-4-keto-D-Rha
GDP-D-Rha
GDP-6-deoxy-D-Tal
Fuc (extracellular)
Fuc
Fuc-1P
GDP-Fuc
GDP-D-Per
GDP-N-acetyl-D-Per
GDP-Glc
CDP-Glc
CDP-4-keto-6-deoxy-D-Glc
CDP-4-keto-3,6-deoxy-D-Glc
CDP-4-keto-3,6-dideoxy-D-Glc epimer
CDP-ascarylose
CDP-abequose
CDP-paratose
CDP-Tyv
AscE
AscF
AXS
ADP-Glc
00520 11/12/15
(c) Kanehisa Laboratories

STREPTOMYCIN BIOSYNTHESIS

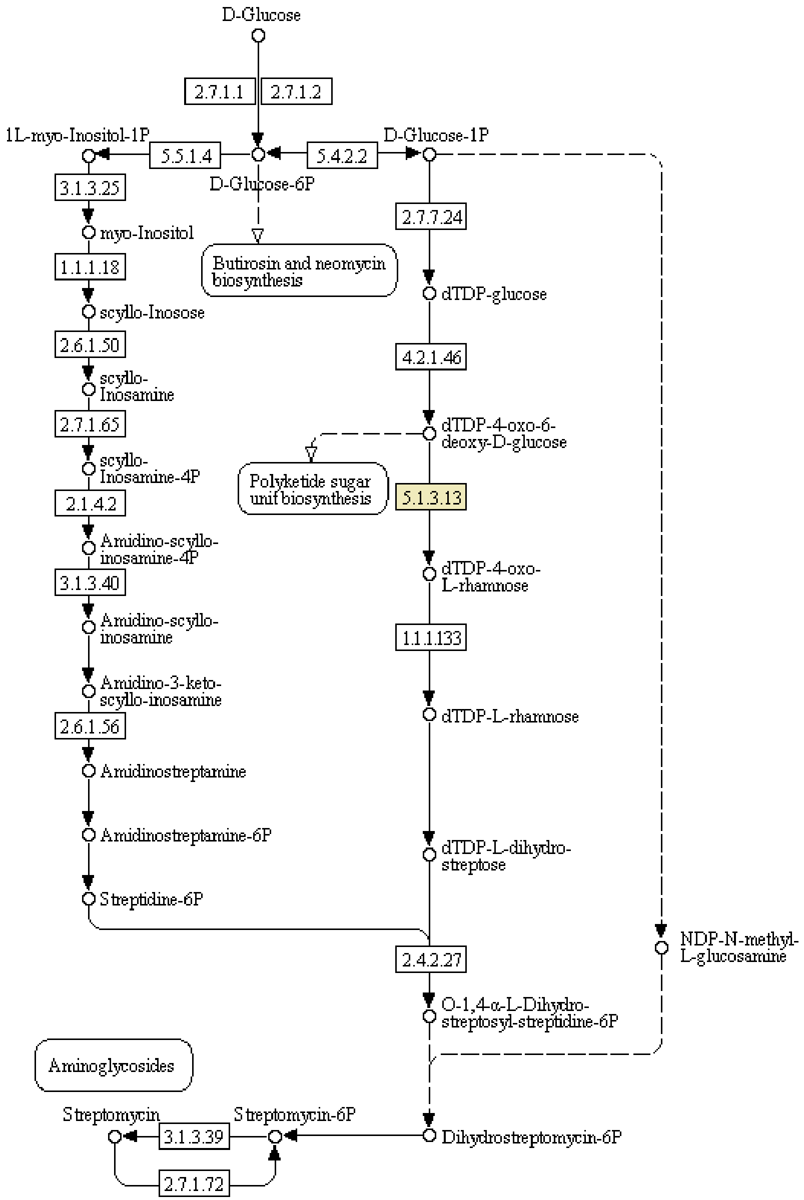

00521 12/27/10

(c) Kanehisa Laboratories

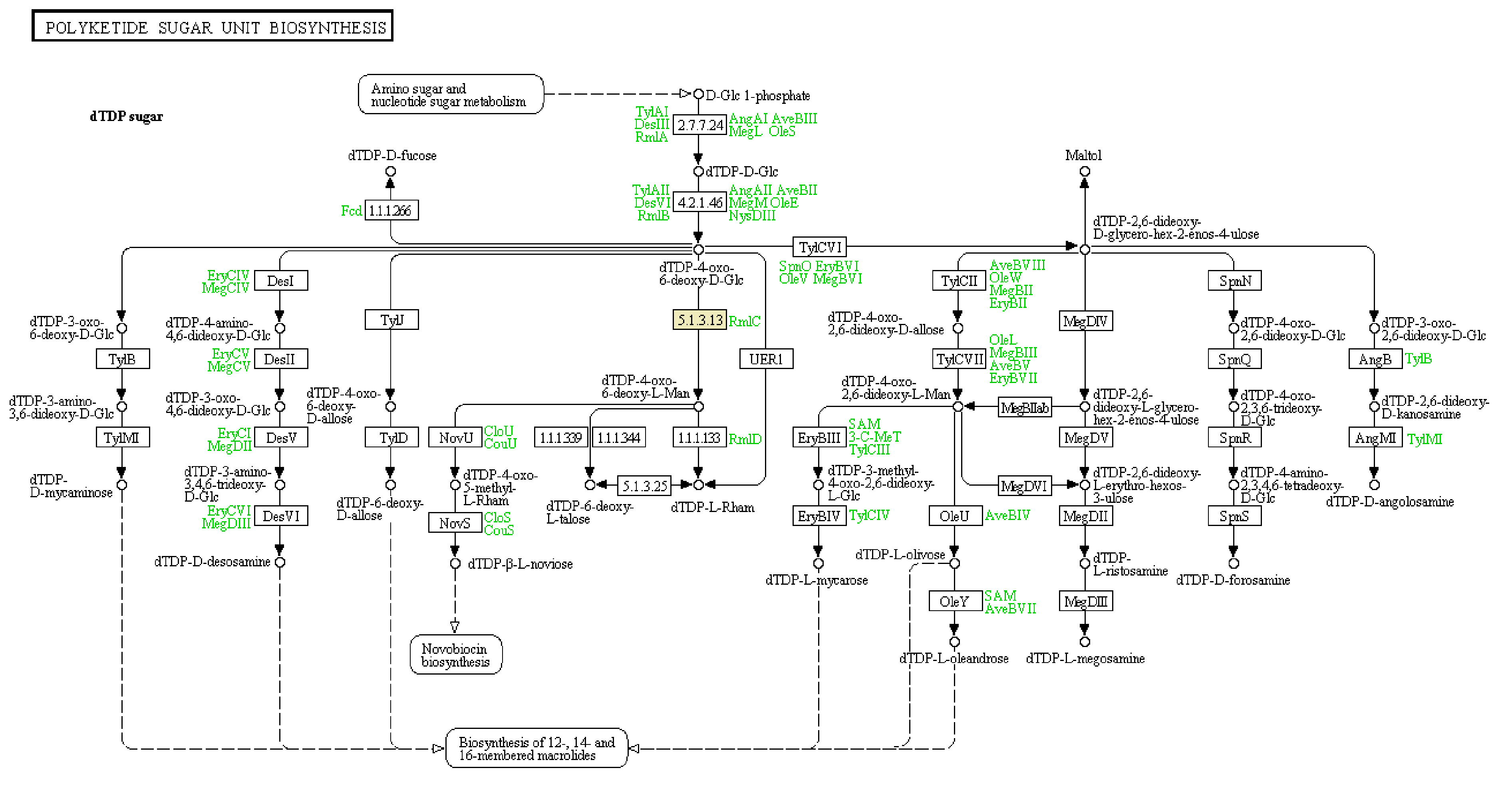

00523 10/20/15
(c) Kanehisa Laboratories

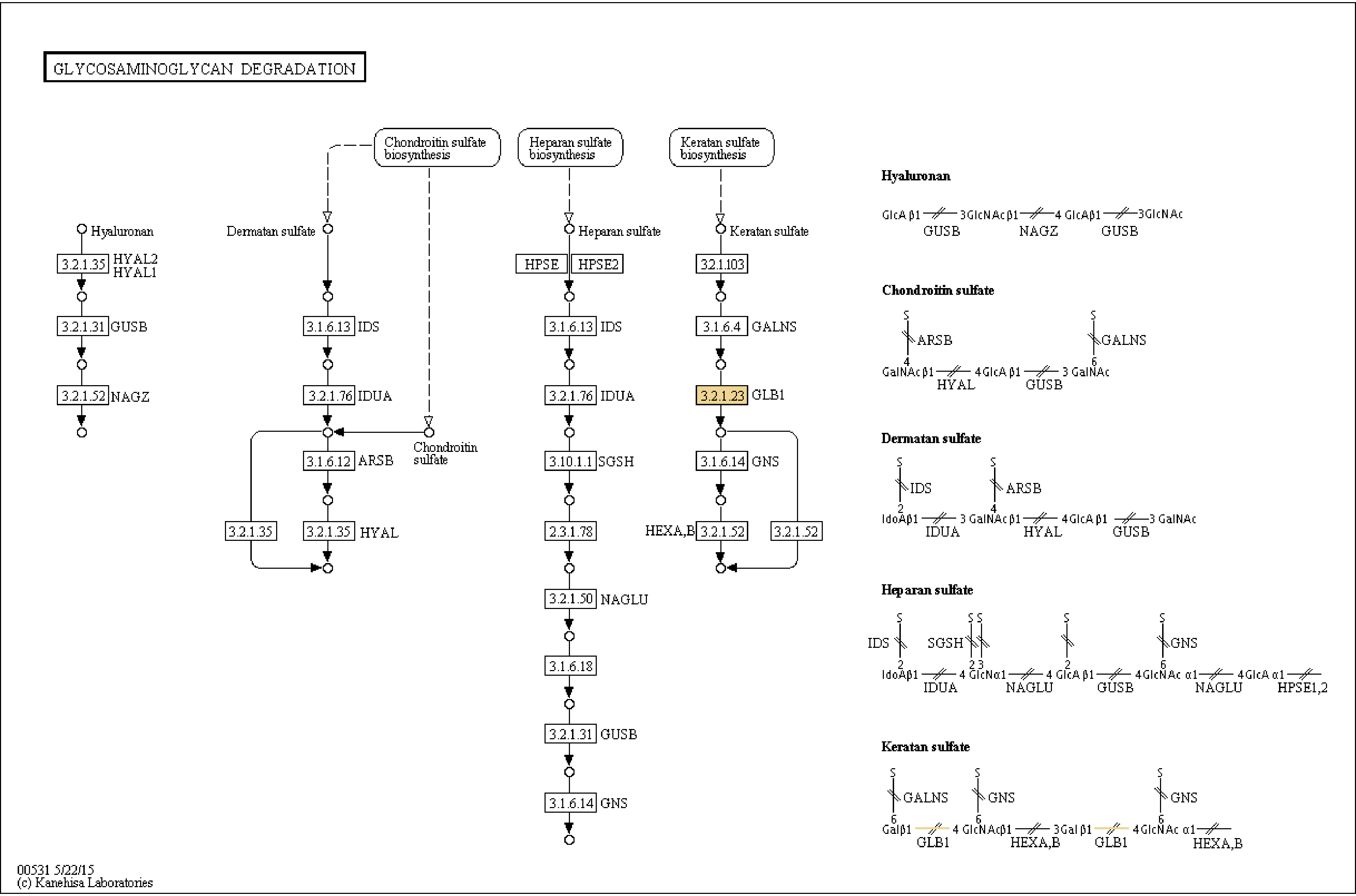

GLYCOSAMINOGLYCAN DEGRADATION
Chondroitin sulfate biosynthesis
Heparan sulfate biosynthesis
Keratan sulfate biosynthesis
Hyaluronan
3.2.1.35 HYAL2 HYAL1
3.2.1.31 GUSB
3.2.1.52 NAGZ
Dermatan sulfate
3.1.6.13 IDS
3.2.1.76 IDUA
3.1.6.12 ARSB
3.2.1.35
3.2.1.35 HYAL
Chondroitin sulfate
Heparan sulfate
HPSE
HPSE2
3.1.6.13 IDS
3.2.1.76 IDUA
3.10.1.1 SGSH
2.3.1.78
3.2.1.50 NAGLU
3.1.6.18
3.2.1.31 GUSB
3.1.6.14 GNS
Keratan sulfate
3.2.1.103
3.1.6.4 GALNS
3.2.1.23 GLB1
3.1.6.14 GNS
HEXA,B 3.2.1.52
3.2.1.52
Hyaluronan
GlcA β1 3GlcNAc β1 4 GlcA β1 3GlcNAc
GUSB NAGZ GUSB
Chondroitin sulfate
ARSB GALNS
GalNAc β1 4GlcA β1 3 GalNAc
HYAL GUSB
Dermatan sulfate
IDS ARSB
IdoAβ1 3 GalNAc β1 4GlcA β1 3 GalNAc
IDUA HYAL GUSB
Heparan sulfate
IDS SGSH GNS
IdoAβ1 4 GlcNα1 4 GlcA β1 4GlcNAc α1 4GlcA α1
IDUA NAGLU GUSB NAGLU HPSE1,2
Keratan sulfate
GALNS GNS GNS
Galβ1 4 GlcNAcβ1 3Gal β1 4GlcNAc α1
GLB1 HEXA,B GLB1 HEXA,B
00531 5/22/15
(c) Kanehisa Laboratories

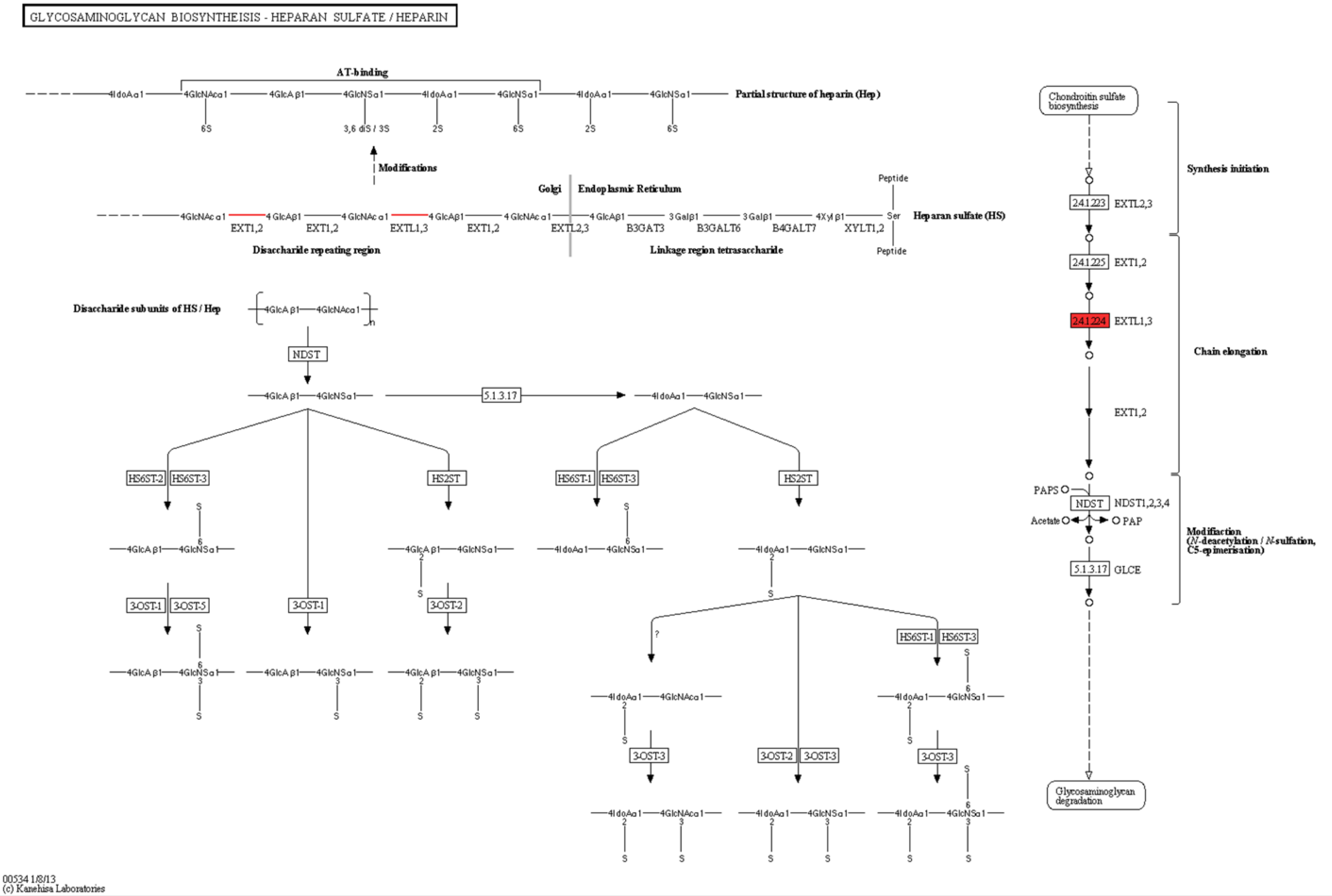
GLYCOSAMINOGLYCAN BIOSYNTHEISIS - HEPARAN SULFATE / HEPARIN
AT-binding
Partial structure of heparin (Hep)
Modifications
Golgi
Endoplasmic Reticulum
Heparan sulfate (HS)
Disaccharide repeating region
Linkage region tetrasaccharide
Peptide
Ser
EXT1,2
EXTL1,3
EXTL2,3
B3GAT3
B3GALT6
B4GALT7
XYLT1,2
Disaccharide subunits of HS / Hep
NDST
5.1.3.17
HS6ST-1
HS6ST-2
HS6ST-3
HS2ST
3-OST-1
3-OST-2
3-OST-3
3-OST-5
Chondroitin sulfate biosynthesis
2.4.1.223
EXTL2,3
Synthesis initiation
2.4.1.225
EXT1,2
2.4.1.224
EXTL1,3
Chain elongation
PAPS
NDST1,2,3,4
Acetate
PAP
Modifiaction (N-deacetylation / N-sulfation, C5-epimerisation)
GLCE
Glycosaminoglycan degradation
00534 1/8/13
(c) Kanehisa Laboratories

LIPOPOLYSACCHARIDE BIOSYNTHESIS

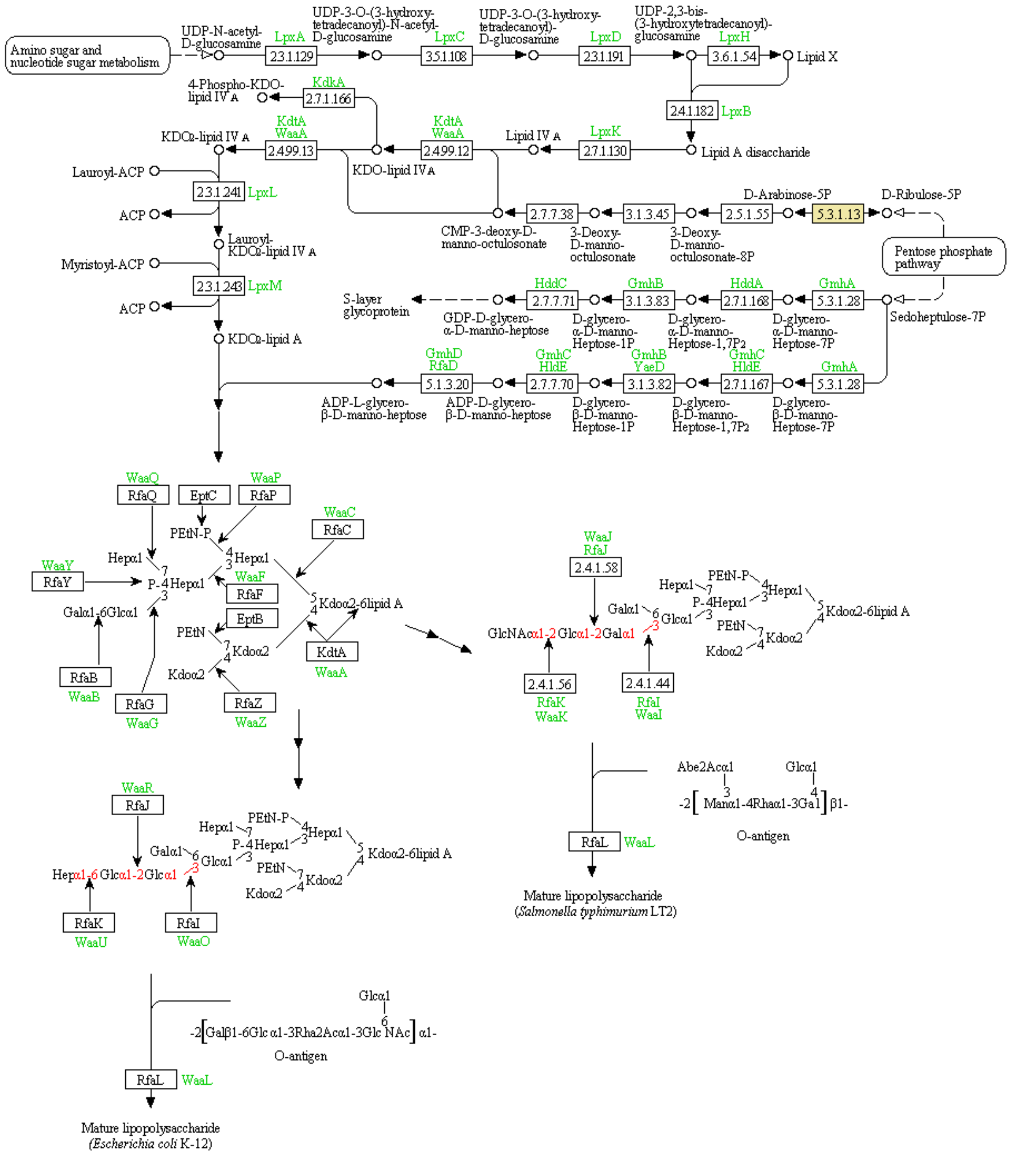

00540 6/16/15
(c) Kanehisa Laboratories

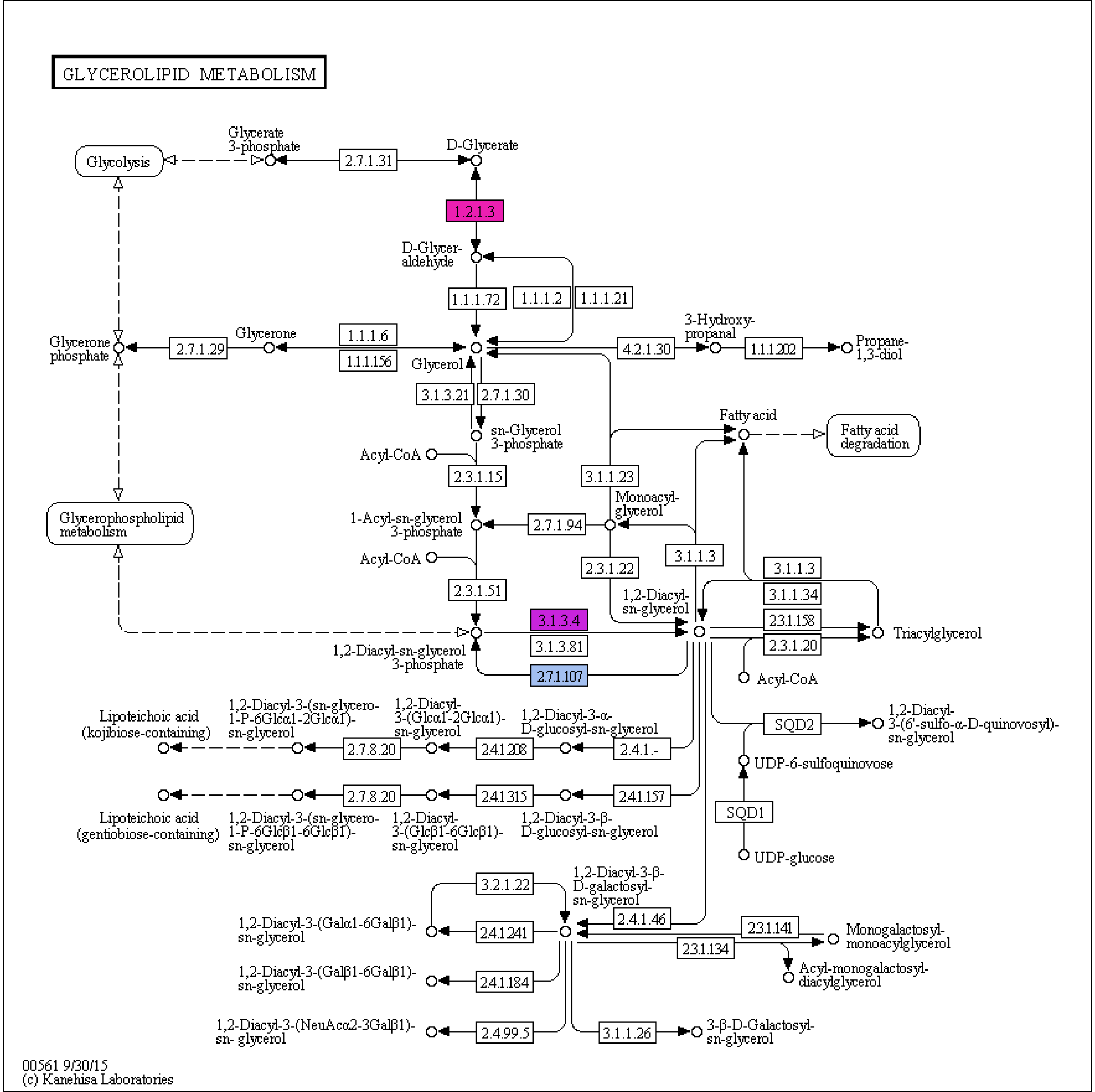
GLYCEROLIPID METABOLISM
Glycolysis
Glycerate 3-phosphate
2.7.1.31
D-Glycerate
1.2.1.3
D-Glycer-aldehyde
1.1.1.72
1.1.1.2
1.1.1.21
Glycerone phosphate
2.7.1.29
Glycerone
1.1.1.6
1.1.1.156
Glycerol
4.2.1.30
3-Hydroxy-propanal
1.1.1.202
Propane-1,3-diol
3.1.3.21
2.7.1.30
sn-Glycerol 3-phosphate
Fatty acid
Fatty acid degradation
Acyl-CoA
2.3.1.15
3.1.1.23
Glycerophospholipid metabolism
Monoacyl-glycerol
1-Acyl-sn-glycerol 3-phosphate
2.7.1.94
3.1.1.3
Acyl-CoA
2.3.1.22
3.1.1.3
3.1.1.34
2.3.1.51
1,2-Diacyl-sn-glycerol
2.3.1.158
3.1.3.4
3.1.3.81
Triacylglycerol
2.3.1.20
1,2-Diacyl-sn-glycerol 3-phosphate
Acyl-CoA
2.7.1.107
Lipoteichoic acid (kojibiose-containing)
1,2-Diacyl-3-(sn-glycero-1-P-6Glcα1-2Glcα1)-sn-glycerol
1,2-Diacyl-3-(Glcα1-2Glcα1)-sn-glycerol
1,2-Diacyl-3-α-D-glucosyl-sn-glycerol
SQD2
1,2-Diacyl-3-(6'-sulfo-α-D-quinovosyl)-sn-glycerol
2.7.8.20
2.4.1.208
2.4.1.-
UDP-6-sulfoquinovose
2.7.8.20
2.4.1.315
2.4.1.157
SQD1
Lipoteichoic acid (gentiobiose-containing)
1,2-Diacyl-3-(sn-glycero-1-P-6Glcβ1-6Glcβ1)-sn-glycerol
1,2-Diacyl-3-(Glcβ1-6Glcβ1)-sn-glycerol
1,2-Diacyl-3-β-D-glucosyl-sn-glycerol
UDP-glucose
3.2.1.22
1,2-Diacyl-3-β-D-galactosyl-sn-glycerol
2.4.1.46
2.3.1.141
1,2-Diacyl-3-(Galα1-6Galβ1)-sn-glycerol
2.4.1.241
Monogalactosyl-monoacylglycerol
2.3.1.134
Acyl-monogalactosyl-diacylglycerol
1,2-Diacyl-3-(Galβ1-6Galβ1)-sn-glycerol
2.4.1.184
1,2-Diacyl-3-(NeuAcα2-3Galβ1)-sn- glycerol
2.4.99.5
3.1.1.26
3-β-D-Galactosyl-sn-glycerol
00561 9/30/15
(c) Kanehisa Laboratories

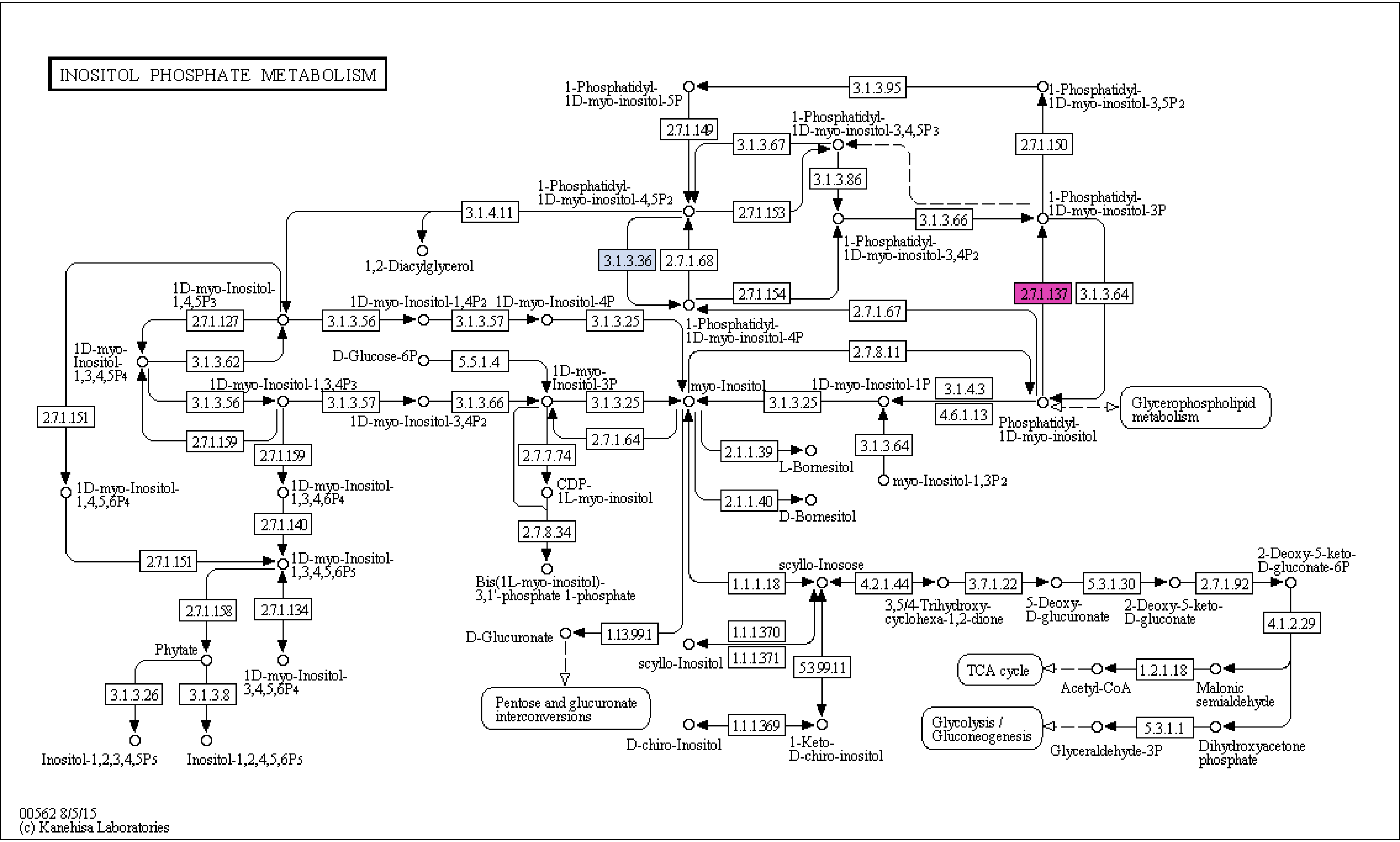
INOSITOL PHOSPHATE METABOLISM
1-Phosphatidyl-1D-myo-inositol-5P
3.1.3.95
1-Phosphatidyl-1D-myo-inositol-3,5P2
2.7.1.149
1-Phosphatidyl-1D-myo-inositol-3,4,5P3
3.1.3.67
2.7.1.150
3.1.3.86
1-Phosphatidyl-1D-myo-inositol-4,5P2
1-Phosphatidyl-1D-myo-inositol-3P
3.1.4.11
2.7.1.153
3.1.3.66
1,2-Diacylglycerol
1-Phosphatidyl-1D-myo-inositol-3,4P2
3.1.3.36
2.7.1.68
1D-myo-Inositol-1,4,5P3
2.7.1.154
2.7.1.137
3.1.3.64
1D-myo-Inositol-1,4P2
1D-myo-Inositol-4P
2.7.1.67
2.7.1.127
3.1.3.56
3.1.3.57
3.1.3.25
1-Phosphatidyl-1D-myo-inositol-4P
1D-myo-Inositol-1,3,4,5P4
3.1.3.62
D-Glucose-6P
5.5.1.4
2.7.8.11
1D-myo-Inositol-3P
1D-myo-Inositol-1,3,4P3
myo-Inositol
1D-myo-Inositol-1P
3.1.4.3
3.1.3.56
3.1.3.57
3.1.3.66
3.1.3.25
3.1.3.25
2.7.1.151
1D-myo-Inositol-3,4P2
4.6.1.13
Phosphatidyl-1D-myo-inositol
Glycerophospholipid metabolism
2.7.1.159
2.7.1.64
2.7.1.159
2.7.7.74
2.1.1.39
L-Bornesitol
3.1.3.64
myo-Inositol-1,3P2
1D-myo-Inositol-1,4,5,6P4
1D-myo-Inositol-1,3,4,6P4
CDP-1L-myo-inositol
2.1.1.40
D-Bornesitol
2.7.1.140
2.7.8.34
2.7.1.151
1D-myo-Inositol-1,3,4,5,6P5
scyllo-Inosose
2-Deoxy-5-keto-D-gluconate-6P
Bis(1L-myo-inositol)-3,1'-phosphate 1-phosphate
1.1.1.18
4.2.1.44
3.7.1.22
5.3.1.30
2.7.1.92
3,5/4-Trihydroxy-cyclohexa-1,2-dione
5-Deoxy-D-glucuronate
2-Deoxy-5-keto-D-gluconate
2.7.1.158
2.7.1.134
4.1.2.29
D-Glucuronate
1.13.99.1
1.1.1.370
scyllo-Inositol
1.1.1.371
Phytate
5.3.99.11
TCA cycle
Acetyl-CoA
1.2.1.18
Malonic semialdehyde
1D-myo-Inositol-3,4,5,6P4
3.1.3.26
3.1.3.8
Pentose and glucuronate interconversions
Glycolysis / Gluconeogenesis
5.3.1.1
1.1.1.369
D-chiro-Inositol
1-Keto-D-chiro-inositol
Glyceraldehyde-3P
Dihydroxyacetone phosphate
Inositol-1,2,3,4,5P5
Inositol-1,2,4,5,6P5
00562 8/5/15
(c) Kanehisa Laboratories

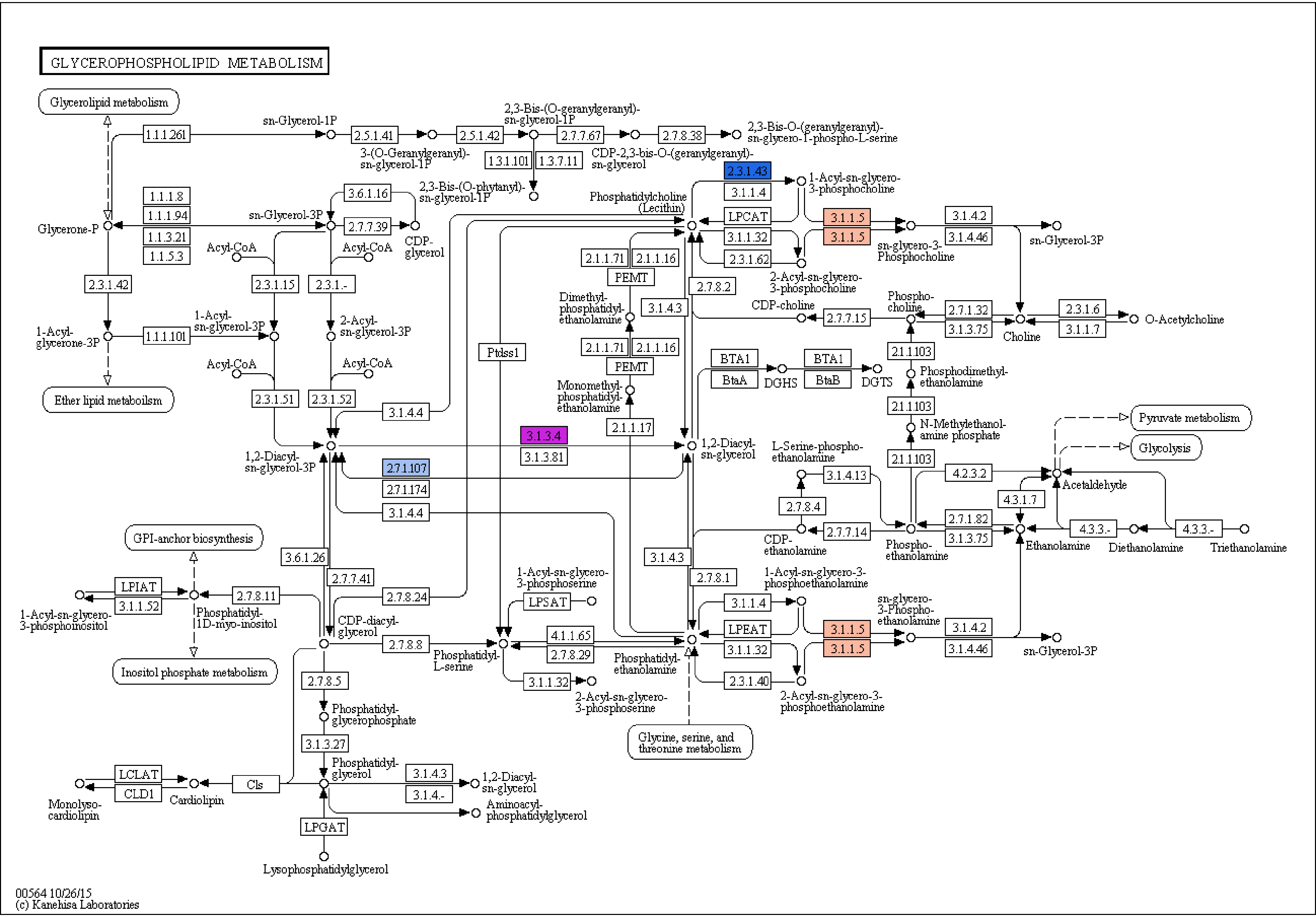
GLYCEROPHOSPHOLIPID METABOLISM
Glycerolipid metabolism
Ether lipid metaboilsm
GPI-anchor biosynthesis
Inositol phosphate metabolism
Pyruvate metabolism
Glycolysis
Glycine, serine, and threonine metabolism
sn-Glycerol-1P
3-(O-Geranylgeranyl)-sn-glycerol-1P
2,3-Bis-(O-geranylgeranyl)-sn-glycerol-1P
2,3-Bis-(O-phytanyl)-sn-glycerol-1P
CDP-2,3-bis-O-(geranylgeranyl)-sn-glycerol
2,3-Bis-O-(geranylgeranyl)-sn-glycero-1-phospho-L-serine
Glycerone-P
sn-Glycerol-3P
CDP-glycerol
Acyl-CoA
1-Acyl-glycerone-3P
1-Acyl-sn-glycerol-3P
2-Acyl-sn-glycerol-3P
1,2-Diacyl-sn-glycerol-3P
Phosphatidylcholine (Lecithin)
1-Acyl-sn-glycero-3-phosphocholine
2-Acyl-sn-glycero-3-phosphocholine
sn-glycero-3-Phosphocholine
sn-Glycerol-3P
Phosphocholine
CDP-choline
Choline
O-Acetylcholine
Dimethyl-phosphatidyl-ethanolamine
Monomethyl-phosphatidyl-ethanolamine
Phosphodimethyl-ethanolamine
N-Methylethanol-amine phosphate
DGHS
DGTS
1,2-Diacyl-sn-glycerol
L-Serine-phospho-ethanolamine
Acetaldehyde
CDP-ethanolamine
Phospho-ethanolamine
Ethanolamine
Diethanolamine
Triethanolamine
1-Acyl-sn-glycero-3-phosphoserine
1-Acyl-sn-glycero-3-phosphoethanolamine
sn-glycero-3-Phospho-ethanolamine
Phosphatidyl-L-serine
Phosphatidyl-ethanolamine
2-Acyl-sn-glycero-3-phosphoserine
2-Acyl-sn-glycero-3-phosphoethanolamine
1-Acyl-sn-glycero-3-phosphoinositol
Phosphatidyl-1D-myo-inositol
CDP-diacyl-glycerol
Phosphatidyl-glycerophosphate
Phosphatidyl-glycerol
1,2-Diacyl-sn-glycerol
Aminoacyl-phosphatidylglycerol
Lysophosphatidylglycerol
Cardiolipin
Monolyso-cardiolipin
1.1.1.261
2.5.1.41
2.5.1.42
2.7.7.67
2.7.8.38
1.3.1.101
1.3.7.11
1.1.1.8
1.1.1.94
1.1.3.21
1.1.5.3
3.6.1.16
2.7.7.39
2.3.1.42
2.3.1.15
2.3.1.-
1.1.1.101
2.3.1.51
2.3.1.52
3.1.4.4
2.3.1.43
3.1.1.4
LPCAT
3.1.1.32
2.3.1.62
3.1.1.5
3.1.4.2
3.1.4.46
2.1.1.71
2.1.1.16
PEMT
2.7.8.2
3.1.4.3
2.7.7.15
2.7.1.32
3.1.3.75
2.3.1.6
3.1.1.7
Ptdss1
2.1.1.17
BTA1
BtaA
BtaB
2.1.1.103
3.1.3.4
3.1.3.81
2.7.1.107
2.7.1.174
3.1.4.13
4.2.3.2
4.3.1.7
2.7.8.4
2.7.7.14
2.7.1.82
4.3.3.-
3.6.1.26
2.7.7.41
2.7.8.1
2.7.8.24
2.7.8.11
LPIAT
3.1.1.52
LPSAT
LPEAT
2.3.1.40
4.1.1.65
2.7.8.29
2.7.8.8
2.7.8.5
3.1.3.27
3.1.4.-
LCLAT
CLD1
Cls
LPGAT
00564 10/26/15
(c) Kanehisa Laboratories

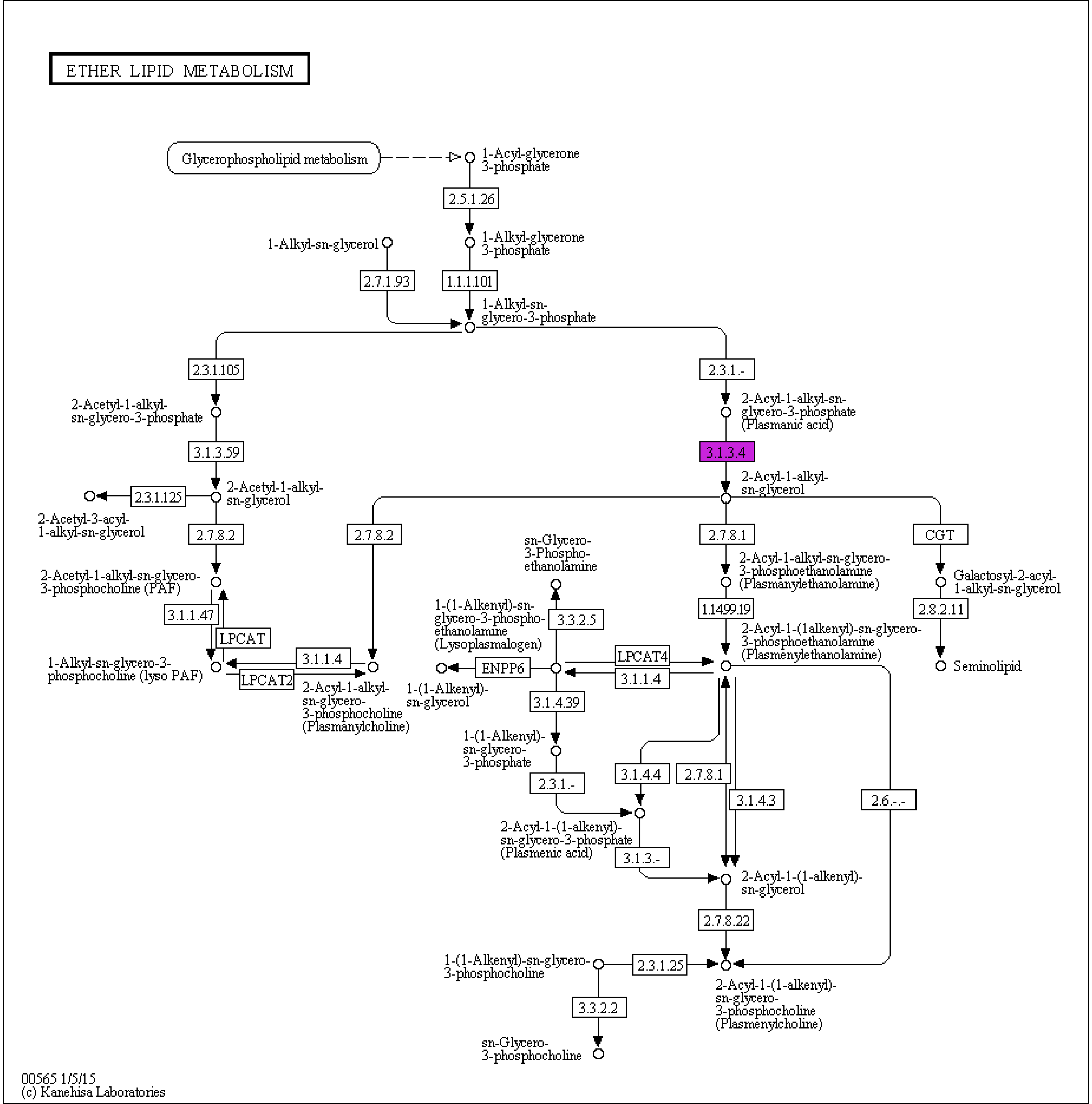
ETHER LIPID METABOLISM
Glycerophospholipid metabolism
1-Acyl-glycerone 3-phosphate
2.5.1.26
1-Alkyl-glycerone 3-phosphate
1-Alkyl-sn-glycerol
2.7.1.93
1.1.1.101
1-Alkyl-sn-glycero-3-phosphate
2.3.1.105
2.3.1.-
2-Acetyl-1-alkyl-sn-glycero-3-phosphate
2-Acyl-1-alkyl-sn-glycero-3-phosphate (Plasmanic acid)
3.1.3.59
3.1.3.4
2-Acetyl-1-alkyl-sn-glycerol
2.3.1.125
2-Acetyl-3-acyl-1-alkyl-sn-glycerol
2-Acyl-1-alkyl-sn-glycerol
2.7.8.2
2.7.8.2
2.7.8.1
CGT
2-Acetyl-1-alkyl-sn-glycero-3-phosphocholine (PAF)
sn-Glycero-3-Phospho-ethanolamine
2-Acyl-1-alkyl-sn-glycero-3-phosphoethanolamine (Plasmanylethanolamine)
Galactosyl-2-acyl-1-alkyl-sn-glycerol
3.1.1.47
LPCAT
1.14.99.19
2.8.2.11
1-(1-Alkenyl)-sn-glycero-3-phospho-ethanolamine (Lysoplasmalogen)
3.3.2.5
2-Acyl-1-(1alkenyl)-sn-glycero-3-phosphoethanolamine (Plasmenylethanolamine)
1-Alkyl-sn-glycero-3-phosphocholine (lyso PAF)
3.1.1.4
LPCAT2
ENPP6
LPCAT4
3.1.1.4
Seminolipid
2-Acyl-1-alkyl-sn-glycero-3-phosphocholine (Plasmanylcholine)
1-(1-Alkenyl)-sn-glycerol
3.1.4.39
1-(1-Alkenyl)-sn-glycero-3-phosphate
3.1.4.4
2.7.8.1
3.1.4.3
2.6.-.-
2.3.1.-
2-Acyl-1-(1-alkenyl)-sn-glycero-3-phosphate (Plasmenic acid)
3.1.3.-
2-Acyl-1-(1-alkenyl)-sn-glycerol
2.7.8.22
1-(1-Alkenyl)-sn-glycero-3-phosphocholine
2.3.1.25
2-Acyl-1-(1-alkenyl)-sn-glycero-3-phosphocholine (Plasmenylcholine)
3.3.2.2
sn-Glycero-3-phosphocholine
00565 1/5/15
(c) Kanehisa Laboratories

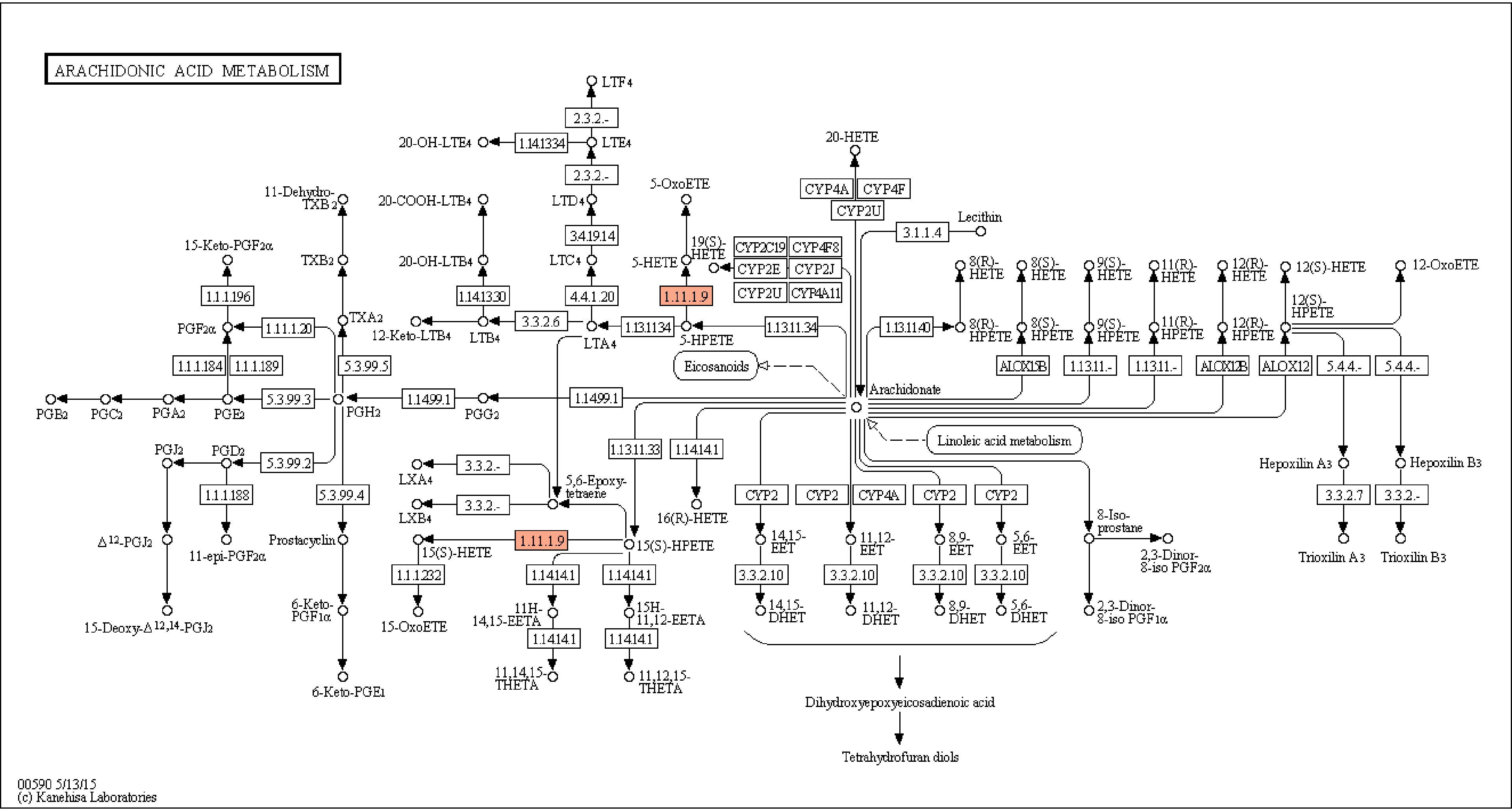

ARACHIDONIC ACID METABOLISM
LTF4
2.3.2.-
20-OH-LTE4
1.14.13.34
LTE4
2.3.2.-
11-Dehydro-TXB2
20-COOH-LTB4
LTD4
5-OxoETE
20-HETE
CYP4A
CYP4F
CYP2U
Lecithin
3.1.1.4
15-Keto-PGF2α
TXB2
20-OH-LTB4
3.4.19.14
LTC4
5-HETE
19(S)-HETE
CYP2C19
CYP4F8
CYP2E
CYP2J
CYP2U
CYP4A11
8(R)-HETE
8(S)-HETE
9(S)-HETE
11(R)-HETE
12(R)-HETE
12(S)-HETE
12-OxoETE
1.1.1.196
1.14.13.30
4.4.1.20
1.11.1.9
12(S)-HPETE
PGF2α
1.11.1.20
TXA2
12-Keto-LTB4
LTB4
3.3.2.6
LTA4
1.13.11.34
5-HPETE
1.13.11.34
1.13.11.40
8(R)-HPETE
8(S)-HPETE
9(S)-HPETE
11(R)-HPETE
12(R)-HPETE
1.1.1.184
1.1.1.189
5.3.99.5
Eicosanoids
ALOX15B
1.13.11.-
1.13.11.-
ALOX12B
ALOX12
5.4.4.-
5.4.4.-
PGB2
PGC2
PGA2
PGE2
5.3.99.3
PGH2
1.14.99.1
PGG2
1.14.99.1
Arachidonate
PGJ2
PGD2
5.3.99.2
3.3.2.-
LXA4
1.13.11.33
1.14.14.1
Linoleic acid metabolism
Hepoxilin A3
Hepoxilin B3
1.1.1.188
5.3.99.4
5,6-Epoxy-tetraene
3.3.2.-
LXB4
16(R)-HETE
CYP2
CYP2
CYP4A
CYP2
CYP2
3.3.2.7
3.3.2.-
Δ12-PGJ2
11-epi-PGF2α
Prostacyclin
1.11.1.9
15(S)-HETE
15(S)-HPETE
14,15-EET
11,12-EET
8,9-EET
5,6-EET
8-Iso-prostane
2,3-Dinor-8-iso PGF2α
Trioxilin A3
Trioxilin B3
1.1.1.232
1.14.14.1
1.14.14.1
3.3.2.10
3.3.2.10
3.3.2.10
3.3.2.10
6-Keto-PGF1α
15-OxoETE
11H-14,15-EETA
15H-11,12-EETA
14,15-DHET
11,12-DHET
8,9-DHET
5,6-DHET
2,3-Dinor-8-iso PGF1α
15-Deoxy-Δ12,14-PGJ2
1.14.14.1
1.14.14.1
11,14,15-THETA
11,12,15-THETA
6-Keto-PGE1
Dihydroxyepoxyeicosadienoic acid
Tetrahydrofuran diols
00590 5/13/15
(c) Kanehisa Laboratories

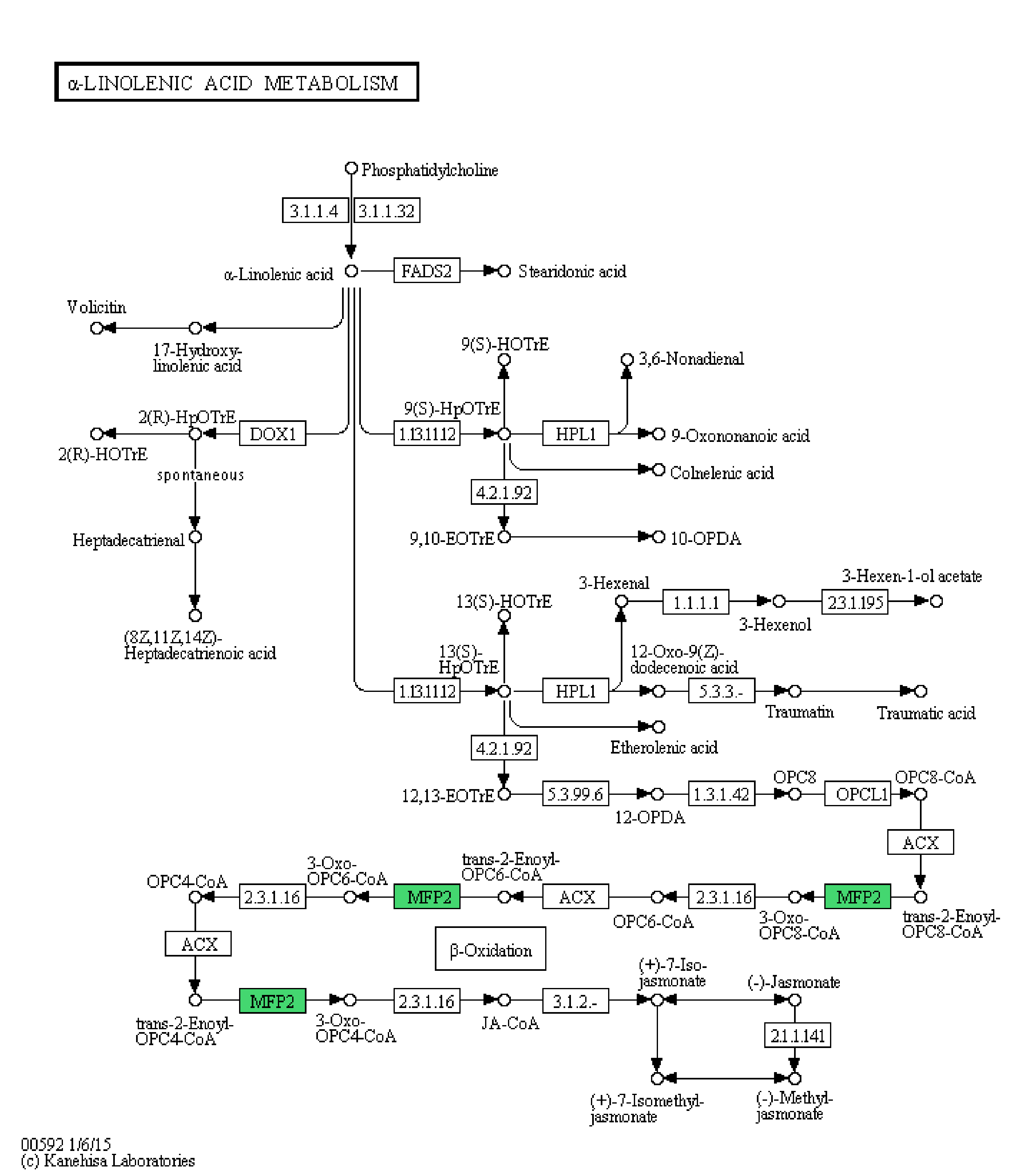
α-LINOLENIC ACID METABOLISM
Phosphatidylcholine
3.1.1.4
3.1.1.32
α-Linolenic acid
FADS2
Stearidonic acid
Volicitin
17-Hydroxy-linolenic acid
9(S)-HOTrE
3,6-Nonadienal
2(R)-HpOTrE
DOX1
2(R)-HOTrE
9(S)-HpOTrE
1.13.11.12
HPL1
9-Oxononanoic acid
Colnelenic acid
spontaneous
4.2.1.92
Heptadecatrienal
9,10-EOTrE
10-OPDA
(8Z,11Z,14Z)-Heptadecatrienoic acid
13(S)-HOTrE
3-Hexenal
1.1.1.1
3-Hexenol
3-Hexen-1-ol acetate
2.3.1.195
13(S)-HpOTrE
12-Oxo-9(Z)-dodecenoic acid
5.3.3.-
Traumatin
Traumatic acid
Etherolenic acid
12,13-EOTrE
5.3.99.6
12-OPDA
1.3.1.42
OPC8
OPCL1
OPC8-CoA
ACX
OPC4-CoA
2.3.1.16
3-Oxo-OPC6-CoA
MFP2
trans-2-Enoyl-OPC6-CoA
OPC6-CoA
3-Oxo-OPC8-CoA
trans-2-Enoyl-OPC8-CoA
β-Oxidation
trans-2-Enoyl-OPC4-CoA
3-Oxo-OPC4-CoA
JA-CoA
3.1.2.-
(+)-7-Iso-jasmonate
(-)-Jasmonate
2.1.1.141
(+)-7-Isomethyl-jasmonate
(-)-Methyl-jasmonate
00592 1/6/15
(c) Kanehisa Laboratories

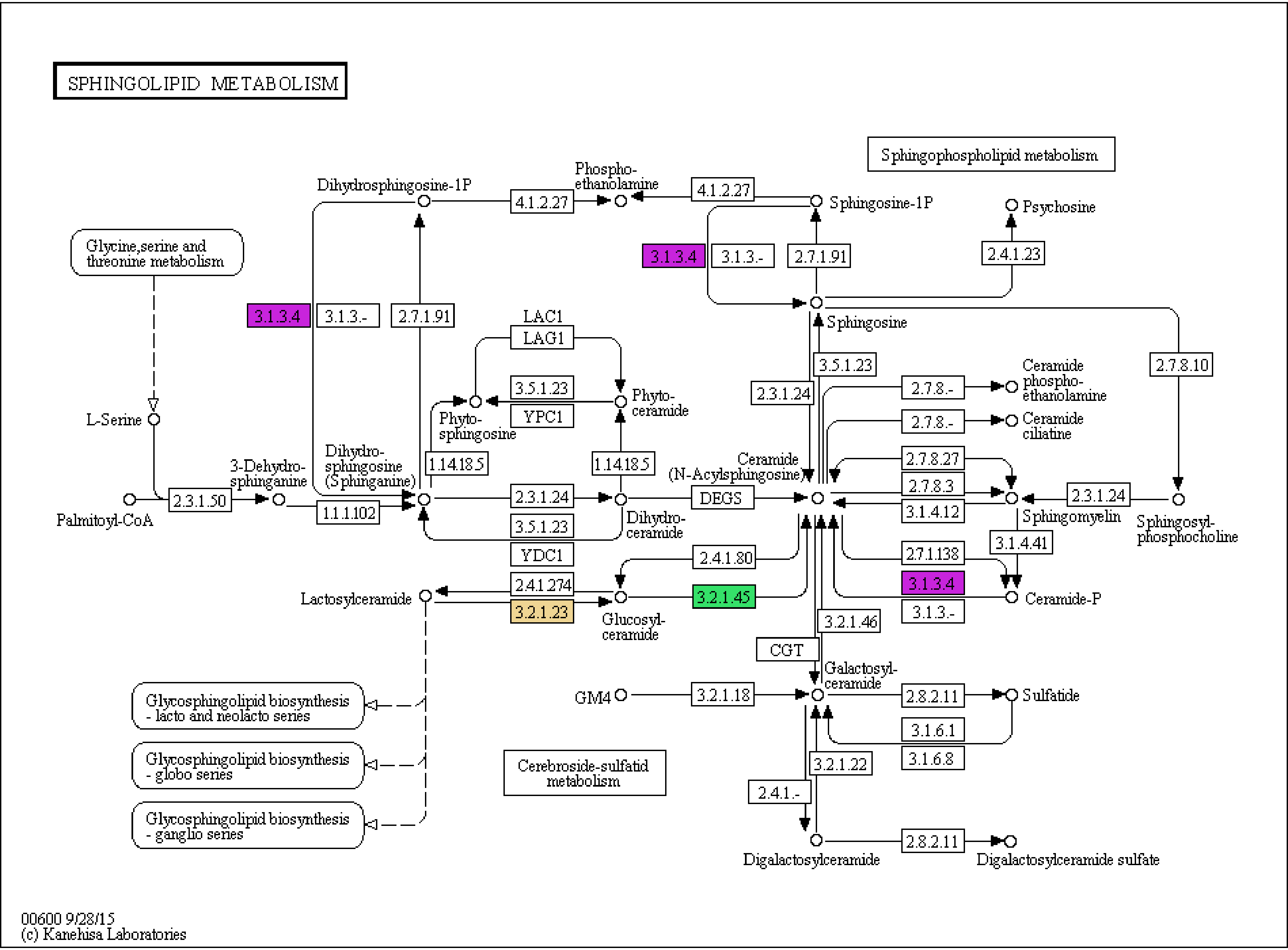
SPHINGOLIPID METABOLISM
Sphingophospholipid metabolism
Glycine,serine and threonine metabolism
Dihydrosphingosine-1P
Phospho-ethanolamine
Sphingosine-1P
Psychosine
4.1.2.27
4.1.2.27
3.1.3.4
3.1.3.-
2.7.1.91
2.4.1.23
3.1.3.4
3.1.3.-
2.7.1.91
Sphingosine
LAC1
LAG1
3.5.1.23
YPC1
Phyto-sphingosine
Phyto-ceramide
3.5.1.23
2.3.1.24
2.7.8.10
2.7.8.-
Ceramide phospho-ethanolamine
2.7.8.-
Ceramide ciliatine
L-Serine
3-Dehydro-sphinganine
Dihydro-sphingosine (Sphinganine)
1.14.18.5
1.14.18.5
Ceramide (N-Acylsphingosine)
2.7.8.27
2.7.8.3
3.1.4.12
2.3.1.24
Palmitoyl-CoA
2.3.1.50
1.1.1.102
2.3.1.24
3.5.1.23
YDC1
Dihydro-ceramide
DEGS
Sphingomyelin
Sphingosyl-phosphocholine
3.1.4.41
2.4.1.80
2.7.1.138
3.1.3.4
3.1.3.-
Lactosylceramide
2.4.1.274
3.2.1.23
Glucosyl-ceramide
3.2.1.45
3.2.1.46
Ceramide-P
CGT
Galactosyl-ceramide
GM4
3.2.1.18
2.8.2.11
Sulfatide
3.1.6.1
3.1.6.8
Glycosphingolipid biosynthesis - lacto and neolacto series
Glycosphingolipid biosynthesis - globo series
Glycosphingolipid biosynthesis - ganglio series
Cerebroside-sulfatid metabolism
3.2.1.22
2.4.1.-
Digalactosylceramide
2.8.2.11
Digalactosylceramide sulfate
00600 9/28/15
(c) Kanehisa Laboratories

GLYCOSPHINGOLIPID BIOSYNTHESIS - GANGLIO SERIES

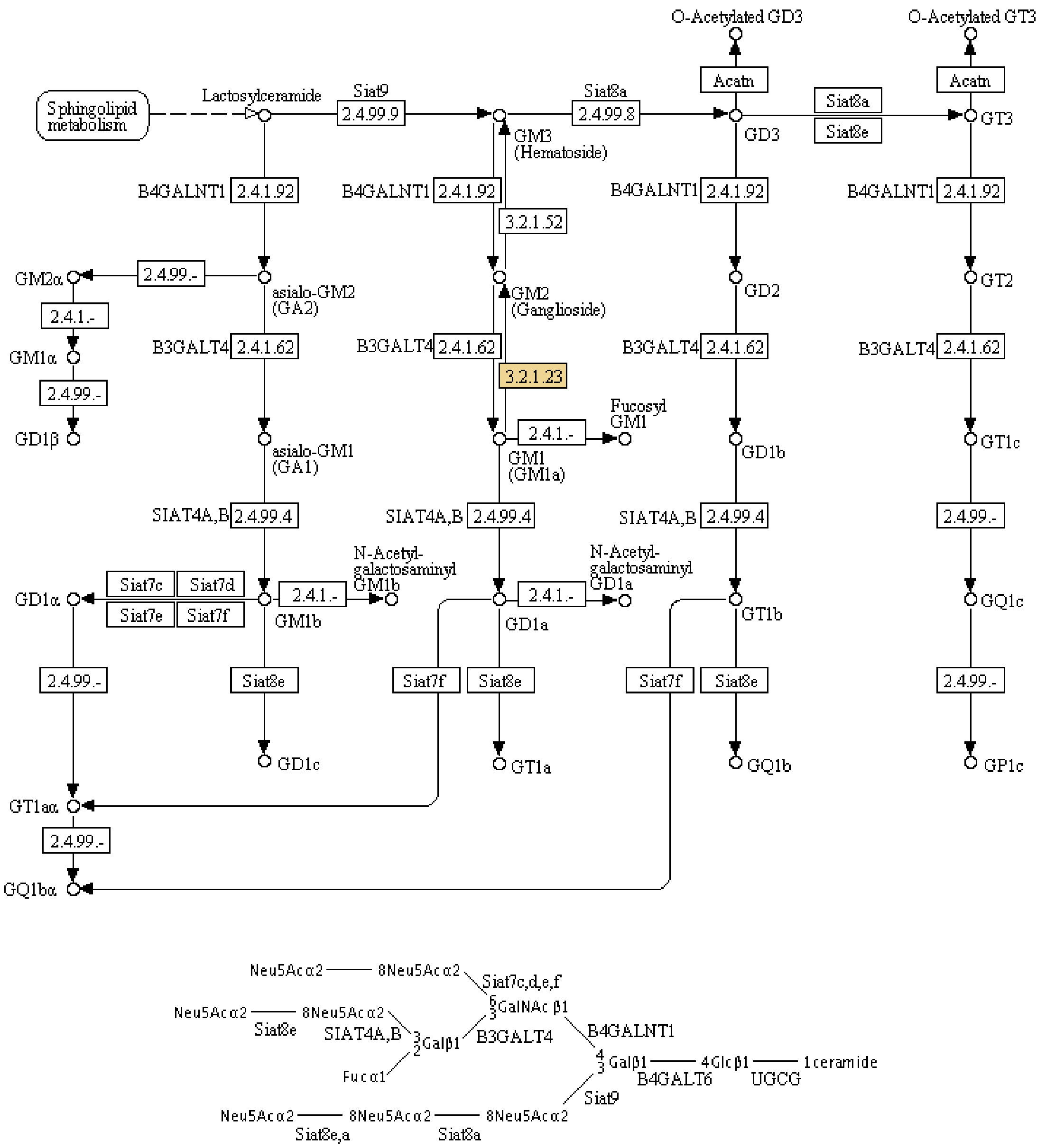

00604 7/2/12

(c) Kanehisa Laboratories

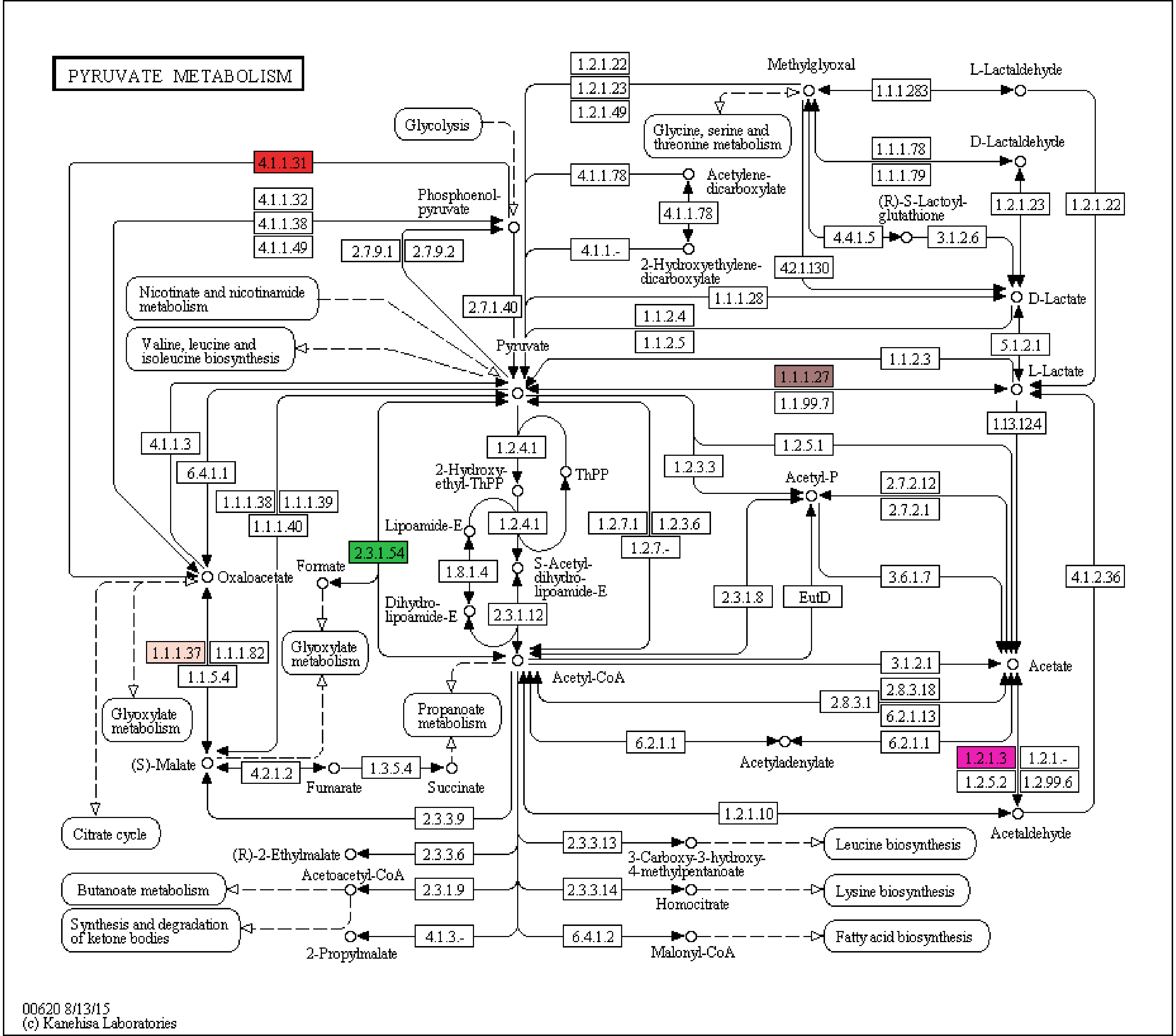
PYRUVATE METABOLISM
Glycolysis
Phosphoenol-pyruvate
Pyruvate
Methylglyoxal
L-Lactaldehyde
D-Lactaldehyde
(R)-S-Lactoyl-glutathione
D-Lactate
L-Lactate
Acetylene-dicarboxylate
2-Hydroxyethylene-dicarboxylate
Glycine, serine and threonine metabolism
Nicotinate and nicotinamide metabolism
Valine, leucine and isoleucine biosynthesis
2-Hydroxy-ethyl-ThPP
ThPP
Lipoamide-E
S-Acetyl-dihydro-lipoamide-E
Dihydro-lipoamide-E
Formate
Oxaloacetate
Glyoxylate metabolism
Acetyl-P
Acetyl-CoA
Acetate
Acetyladenylate
Acetaldehyde
Propanoate metabolism
(S)-Malate
Fumarate
Succinate
Citrate cycle
(R)-2-Ethylmalate
Acetoacetyl-CoA
2-Propylmalate
Butanoate metabolism
Synthesis and degradation of ketone bodies
3-Carboxy-3-hydroxy-4-methylpentanoate
Homocitrate
Malonyl-CoA
Leucine biosynthesis
Lysine biosynthesis
Fatty acid biosynthesis
4.1.1.31
4.1.1.32
4.1.1.38
4.1.1.49
2.7.9.1
2.7.9.2
2.7.1.40
1.2.1.22
1.2.1.23
1.2.1.49
4.1.1.78
4.1.1.-
1.1.1.283
1.1.1.78
1.1.1.79
4.4.1.5
3.1.2.6
4.2.1.130
1.1.1.28
1.1.2.4
1.1.2.5
5.1.2.1
1.1.2.3
1.1.1.27
1.1.99.7
1.13.12.4
1.2.4.1
1.2.5.1
1.2.3.3
2.7.2.12
2.7.2.1
1.2.7.1
1.2.3.6
1.2.7.-
1.8.1.4
2.3.1.12
2.3.1.54
2.3.1.8
EutD
3.6.1.7
4.1.2.36
4.1.1.3
6.4.1.1
1.1.1.38
1.1.1.39
1.1.1.40
1.1.1.37
1.1.1.82
1.1.5.4
4.2.1.2
1.3.5.4
2.3.3.9
2.3.3.6
2.3.1.9
4.1.3.-
3.1.2.1
2.8.3.18
2.8.3.1
6.2.1.13
6.2.1.1
1.2.1.3
1.2.1.-
1.2.5.2
1.2.99.6
1.2.1.10
2.3.3.13
2.3.3.14
6.4.1.2
00620 8/13/15
(c) Kanehisa Laboratories

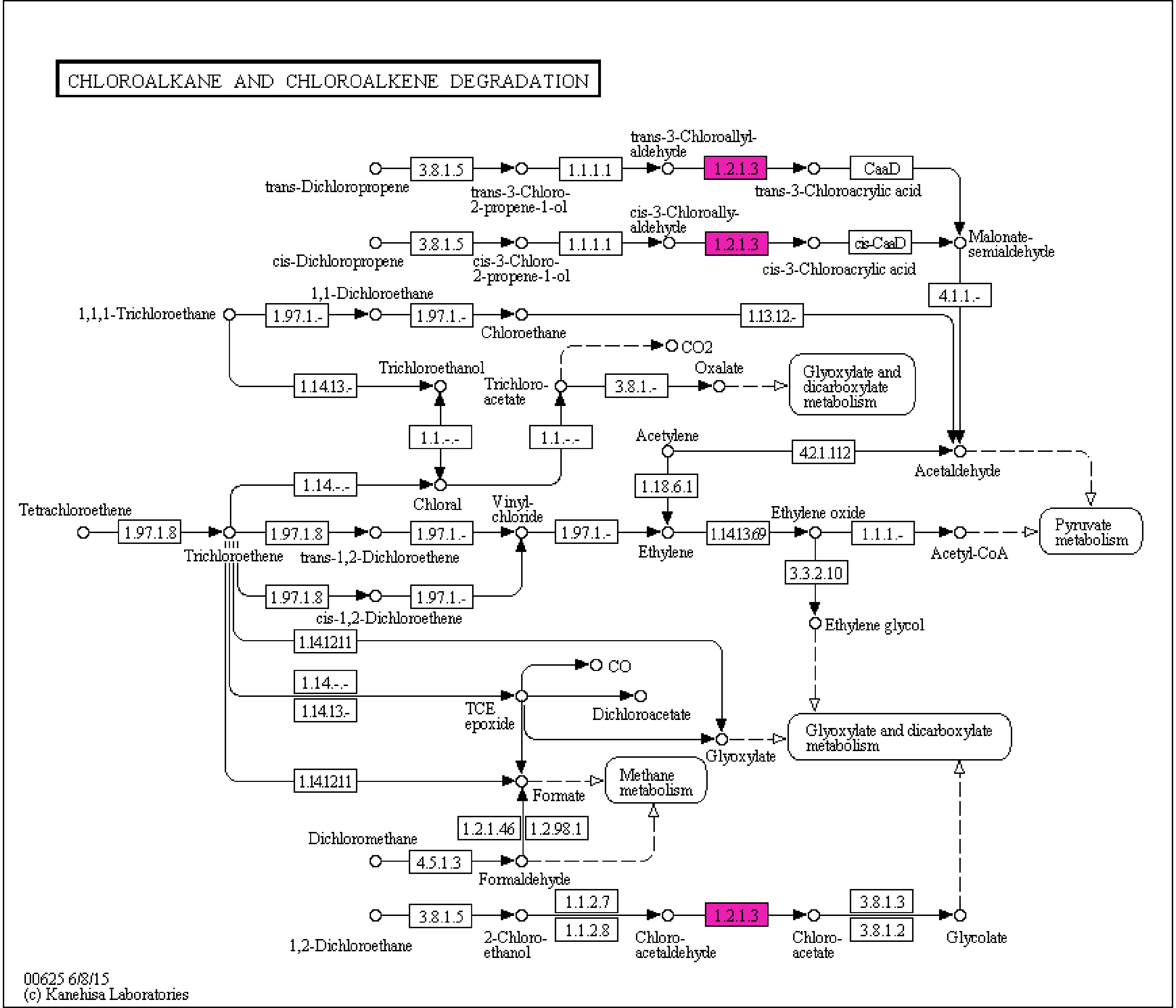
CHLOROALKANE AND CHLOROALKENE DEGRADATION
trans-Dichloropropene
3.8.1.5
trans-3-Chloro-2-propene-1-ol
1.1.1.1
trans-3-Chloroallyl-aldehyde
1.2.1.3
trans-3-Chloroacrylic acid
CaaD
cis-Dichloropropene
cis-3-Chloro-2-propene-1-ol
cis-3-Chloroally-aldehyde
cis-3-Chloroacrylic acid
cis-CaaD
Malonate-semialdehyde
4.1.1.-
1,1,1-Trichloroethane
1.97.1.-
1,1-Dichloroethane
Chloroethane
1.13.12.-
CO2
Trichloroethanol
1.14.13.-
Trichloro-acetate
3.8.1.-
Oxalate
Glyoxylate and dicarboxylate metabolism
1.1.-.-
Acetylene
4.2.1.112
Acetaldehyde
1.18.6.1
1.14.-.-
Chloral
Vinyl-chloride
Tetrachloroethene
1.97.1.8
Trichloroethene
trans-1,2-Dichloroethene
Ethylene
1.14.13.69
Ethylene oxide
1.1.1.-
Acetyl-CoA
Pyruvate metabolism
3.3.2.10
Ethylene glycol
cis-1,2-Dichloroethene
1.14.12.11
CO
TCE epoxide
Dichloroacetate
Glyoxylate
Formate
Methane metabolism
1.2.1.46
1.2.98.1
Dichloromethane
4.5.1.3
Formaldehyde
1,2-Dichloroethane
2-Chloro-ethanol
1.1.2.7
1.1.2.8
Chloro-acetaldehyde
Chloro-acetate
3.8.1.3
3.8.1.2
Glycolate
00625 6/8/15
(c) Kanehisa Laboratories

AMINOBENZOATE DEGRADATION

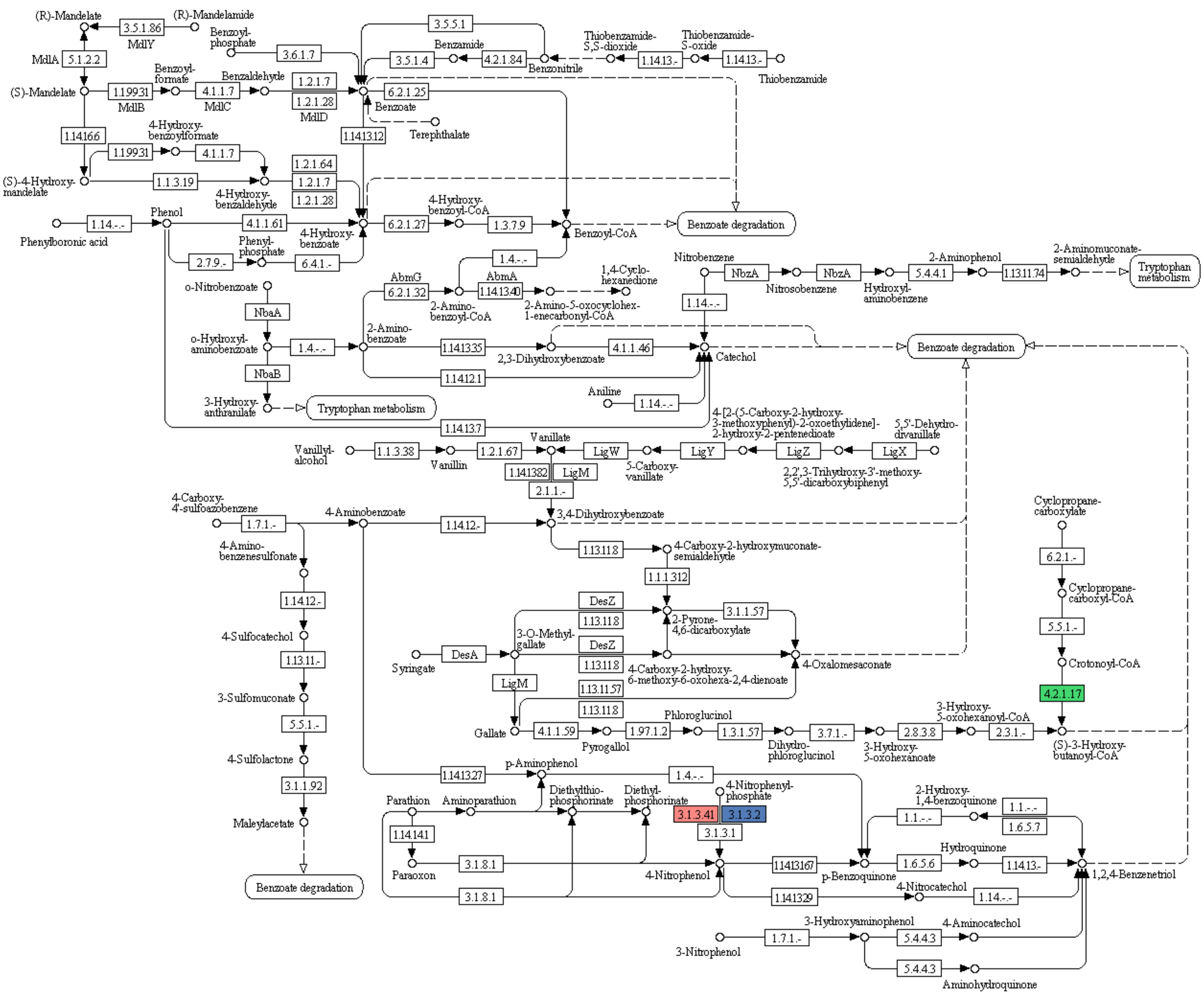

00627 9/12/14
(c) Kanehisa Laboratories

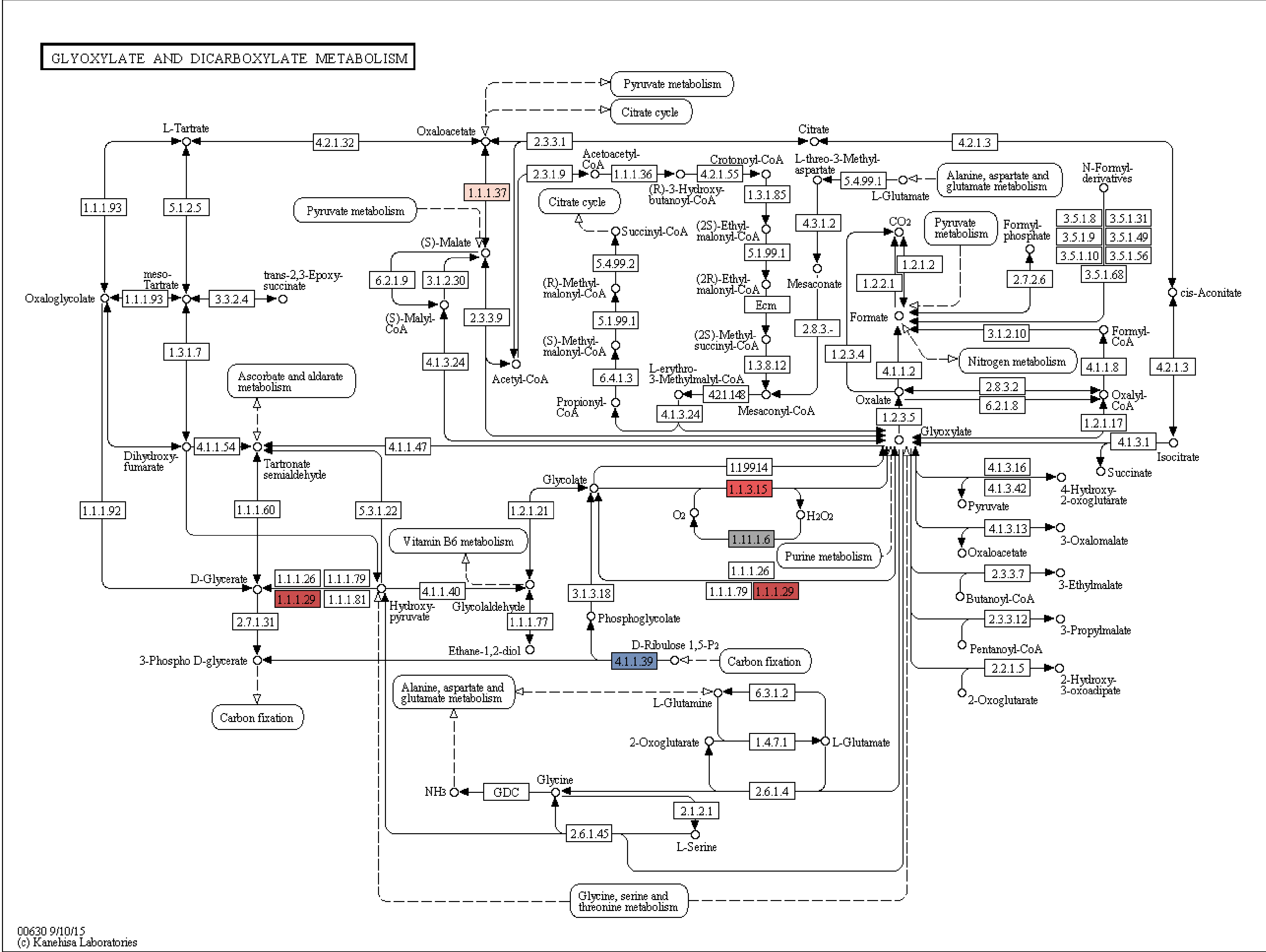
GLYOXYLATE AND DICARBOXYLATE METABOLISM
Pyruvate metabolism
Citrate cycle
L-Tartrate
4.2.1.32
Oxaloacetate
2.3.3.1
Citrate
4.2.1.3
Acetoacetyl-CoA
Crotonoyl-CoA
L-threo-3-Methyl-aspartate
2.3.1.9
1.1.1.36
4.2.1.55
5.4.99.1
L-Glutamate
Alanine, aspartate and glutamate metabolism
N-Formyl-derivatives
1.1.1.93
5.1.2.5
1.1.1.37
Pyruvate metabolism
Citrate cycle
(R)-3-Hydroxy-butanoyl-CoA
1.3.1.85
4.3.1.2
CO2
Pyruvate metabolism
Formyl-phosphate
3.5.1.8
3.5.1.31
3.5.1.9
3.5.1.49
3.5.1.10
3.5.1.56
3.5.1.68
(S)-Malate
Succinyl-CoA
(2S)-Ethyl-malonyl-CoA
5.1.99.1
1.2.1.2
meso-Tartrate
trans-2,3-Epoxy-succinate
6.2.1.9
3.1.2.30
5.4.99.2
Mesaconate
1.2.2.1
2.7.2.6
cis-Aconitate
Oxaloglycolate
1.1.1.93
3.3.2.4
(R)-Methyl-malonyl-CoA
(2R)-Ethyl-malonyl-CoA
Ecm
(S)-Malyl-CoA
2.3.3.9
5.1.99.1
Formate
(2S)-Methyl-succinyl-CoA
2.8.3.-
3.1.2.10
Formyl-CoA
1.3.1.7
(S)-Methyl-malonyl-CoA
1.2.3.4
4.1.3.24
Acetyl-CoA
Nitrogen metabolism
4.1.1.8
4.2.1.3
Ascorbate and aldarate metabolism
1.3.8.12
4.1.1.2
L-erythro-3-Methylmalyl-CoA
6.4.1.3
2.8.3.2
Oxalate
Oxalyl-CoA
4.2.1.148
Propionyl-CoA
6.2.1.8
Mesaconyl-CoA
4.1.3.24
1.2.3.5
1.2.1.17
4.1.1.54
4.1.1.47
Glyoxylate
4.1.3.1
Isocitrate
Dihydroxy-fumarate
Tartronate semialdehyde
1.1.99.14
4.1.3.16
Succinate
Glycolate
1.1.3.15
4-Hydroxy-2-oxoglutarate
4.1.3.42
Pyruvate
1.1.1.92
1.1.1.60
5.3.1.22
1.2.1.21
O2
H2O2
1.11.1.6
4.1.3.13
3-Oxalomalate
Vitamin B6 metabolism
Purine metabolism
Oxaloacetate
1.1.1.26
D-Glycerate
1.1.1.26
1.1.1.79
2.3.3.7
3-Ethylmalate
1.1.1.29
1.1.1.81
4.1.1.40
3.1.3.18
1.1.1.79
1.1.1.29
Butanoyl-CoA
Hydroxy-pyruvate
Glycolaldehyde
2.7.1.31
1.1.1.77
Phosphoglycolate
2.3.3.12
3-Propylmalate
Pentanoyl-CoA
Ethane-1,2-diol
D-Ribulose 1,5-P2
3-Phospho D-glycerate
4.1.1.39
Carbon fixation
2.2.1.5
2-Hydroxy-3-oxoadipate
2-Oxoglutarate
Carbon fixation
Alanine, aspartate and glutamate metabolism
6.3.1.2
L-Glutamine
2-Oxoglutarate
1.4.7.1
L-Glutamate
NH3
GDC
Glycine
2.6.1.4
2.1.2.1
2.6.1.45
L-Serine
Glycine, serine and threonine metabolism
00630 9/10/15
(c) Kanehisa Laboratories

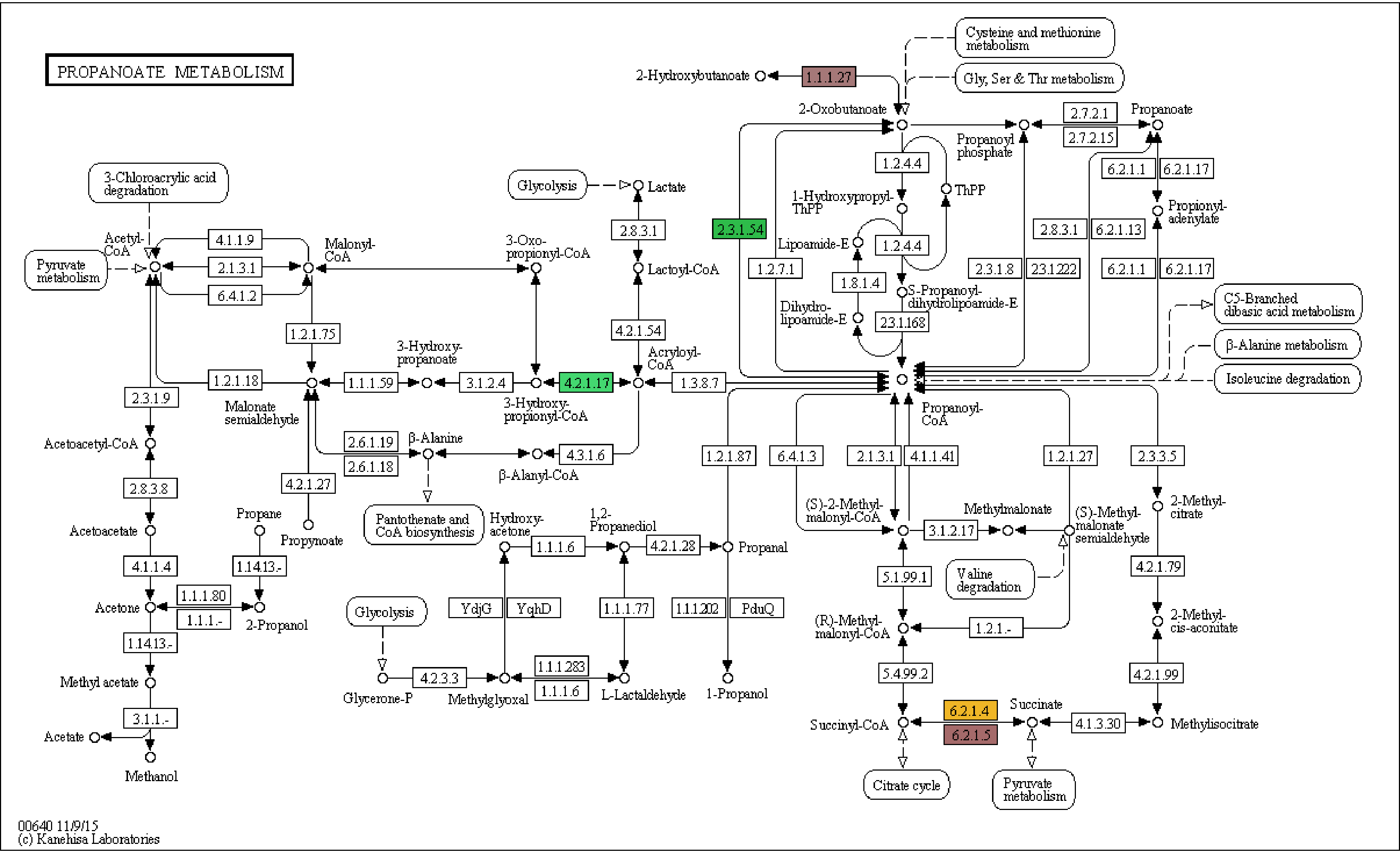
PROPANOATE METABOLISM
Cysteine and methionine metabolism
Gly, Ser & Thr metabolism
2-Hydroxybutanoate
1.1.1.27
2-Oxobutanoate
2.7.2.1
2.7.2.15
Propanoate
Propanoyl phosphate
1.2.4.4
ThPP
1-Hydroxypropyl-ThPP
6.2.1.1
6.2.1.17
Propionyl-adenylate
3-Chloroacrylic acid degradation
Glycolysis
Lactate
2.3.1.54
Lipoamide-E
1.2.4.4
2.8.3.1
2.8.3.1
6.2.1.13
Acetyl-CoA
4.1.1.9
Malonyl-CoA
3-Oxo-propionyl-CoA
Pyruvate metabolism
2.1.3.1
Lactoyl-CoA
1.2.7.1
1.8.1.4
2.3.1.8
2.3.1.222
6.2.1.1
6.2.1.17
6.4.1.2
S-Propanoyl-dihydrolipoamide-E
Dihydro-lipoamide-E
2.3.1.168
C5-Branched dibasic acid metabolism
1.2.1.75
4.2.1.54
3-Hydroxy-propanoate
Acryloyl-CoA
β-Alanine metabolism
1.2.1.18
1.1.1.59
3.1.2.4
4.2.1.17
1.3.8.7
Isoleucine degradation
2.3.1.9
Malonate semialdehyde
3-Hydroxy-propionyl-CoA
Propanoyl-CoA
2.6.1.19
β-Alanine
Acetoacetyl-CoA
2.6.1.18
4.3.1.6
1.2.1.87
6.4.1.3
2.1.3.1
4.1.1.41
1.2.1.27
2.3.3.5
β-Alanyl-CoA
2.8.3.8
4.2.1.27
(S)-2-Methyl-malonyl-CoA
Methylmalonate
(S)-Methyl-malonate semialdehyde
2-Methyl-citrate
Propane
Pantothenate and CoA biosynthesis
Hydroxy-acetone
1,2-Propanediol
Acetoacetate
Propynoate
3.1.2.17
1.1.1.6
4.2.1.28
Propanal
4.1.1.4
1.14.13.-
Valine degradation
5.1.99.1
4.2.1.79
1.1.1.80
Glycolysis
YdjG
YqhD
1.1.1.77
1.1.1.202
PduQ
Acetone
2-Propanol
1.1.1.-
(R)-Methyl-malonyl-CoA
1.2.1.-
2-Methyl-cis-aconitate
1.14.13.-
1.1.1.283
4.2.3.3
Methyl acetate
Glycerone-P
Methylglyoxal
1.1.1.6
L-Lactaldehyde
1-Propanol
5.4.99.2
4.2.1.99
Succinate
6.2.1.4
Succinyl-CoA
4.1.3.30
Methylisocitrate
6.2.1.5
3.1.1.-
Acetate
Methanol
Citrate cycle
Pyruvate metabolism
00640 11/9/15
(c) Kanehisa Laboratories

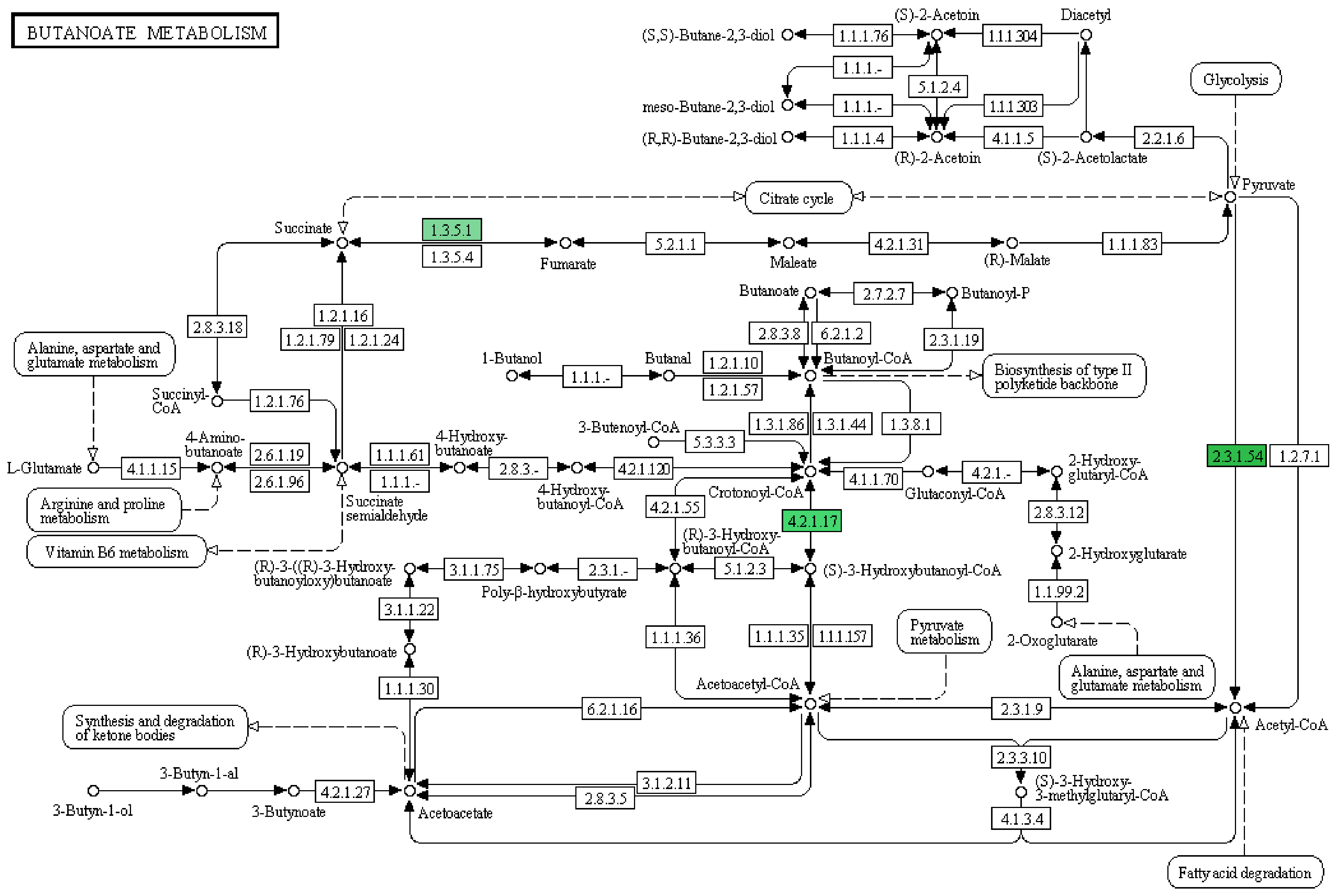

(c) Kanehisa Laboratories

C_5-BRANCHED DIBASIC ACID METABOLISM

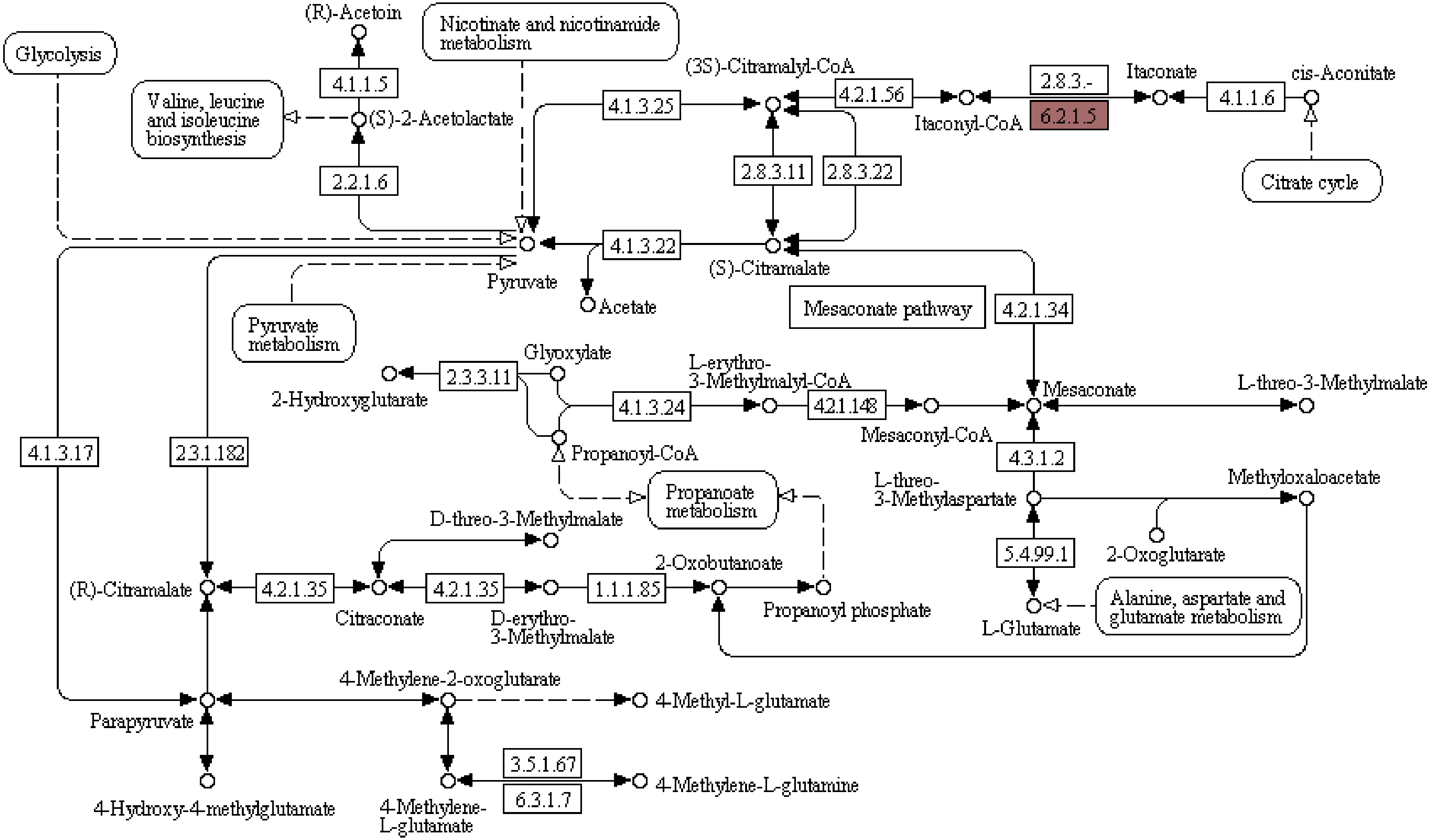

00660 5/20/15
(c) Kanehisa Laboratories

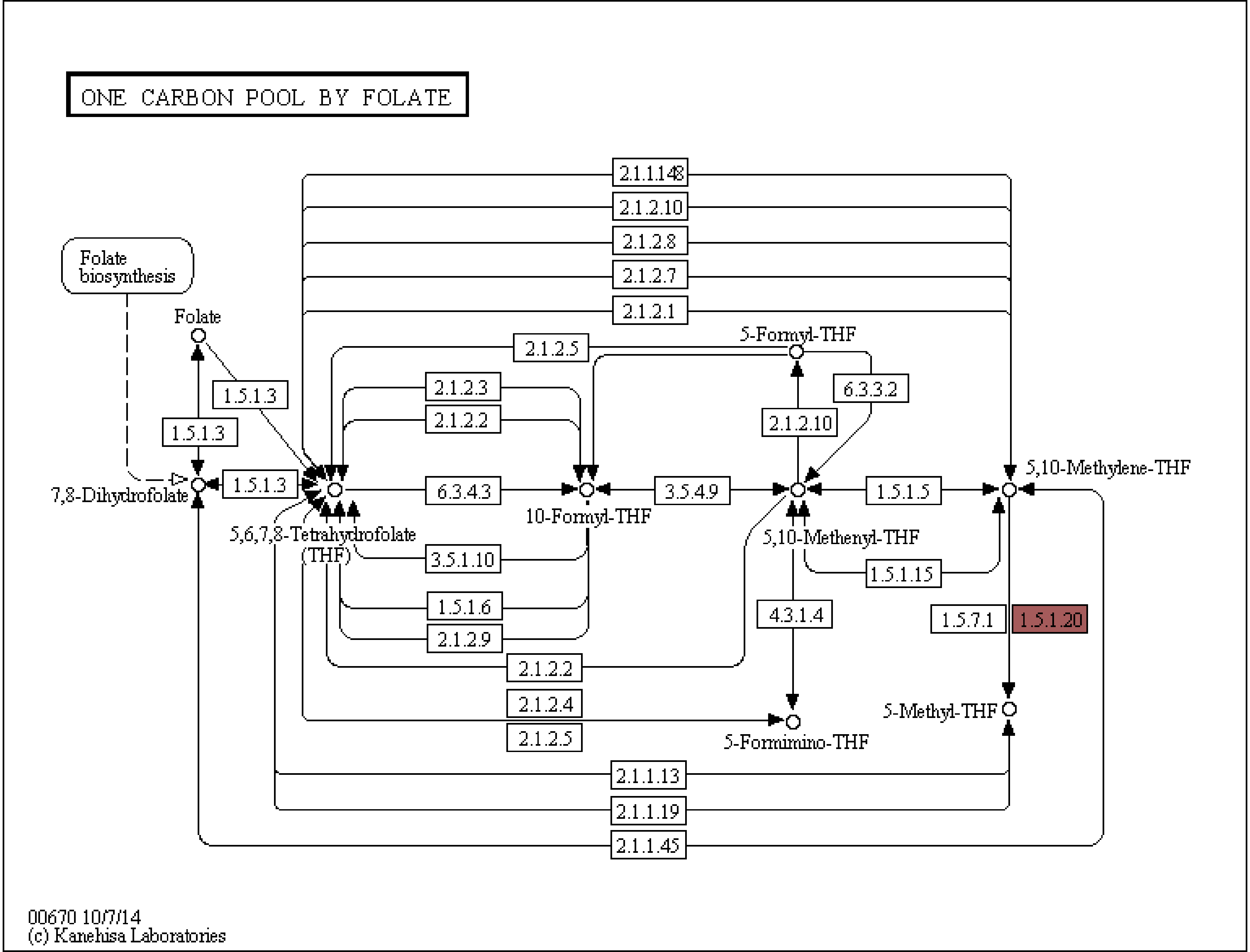
ONE CARBON POOL BY FOLATE
Folate biosynthesis
Folate
1.5.1.3
1.5.1.3
1.5.1.3
7,8-Dihydrofolate
5,6,7,8-Tetrahydrofolate (THF)
2.1.1.148
2.1.2.10
2.1.2.8
2.1.2.7
2.1.2.1
2.1.2.5
2.1.2.3
2.1.2.2
6.3.4.3
10-Formyl-THF
3.5.4.9
5-Formyl-THF
6.3.3.2
2.1.2.10
1.5.1.5
5,10-Methylene-THF
5,10-Methenyl-THF
3.5.1.10
1.5.1.6
2.1.2.9
2.1.2.2
2.1.2.4
2.1.2.5
1.5.1.15
4.3.1.4
1.5.7.1
1.5.1.20
5-Methyl-THF
5-Formimino-THF
2.1.1.13
2.1.1.19
2.1.1.45
00670 10/7/14
(c) Kanehisa Laboratories

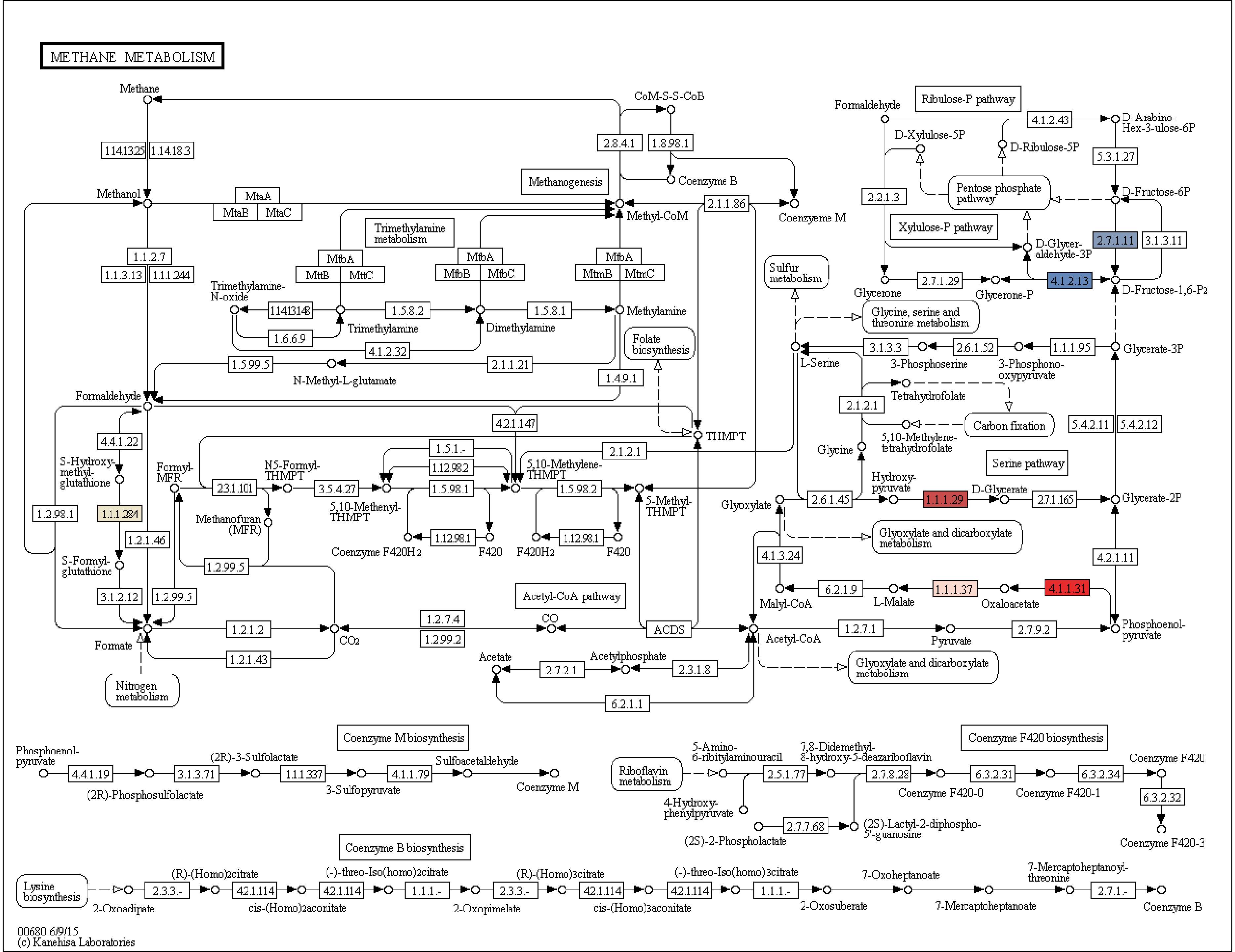

METHANE METABOLISM
Methane
CoM-S-S-CoB
1.14.13.25
1.14.18.3
2.8.4.1
1.8.98.1
Coenzyme B
Methanogenesis
Methanol
MtaA
MtaB
MtaC
Methyl-CoM
2.1.1.86
Coenzyme M
Trimethylamine metabolism
1.1.2.7
1.1.3.13
1.1.1.244
MttA
MttB
MttC
MtbA
MtbB
MtbC
MtmB
MtmC
Trimethylamine-N-oxide
1.14.13.148
1.5.8.2
1.5.8.1
Methylamine
Trimethylamine
Dimethylamine
1.6.6.9
4.1.2.32
Folate biosynthesis
1.5.99.5
2.1.1.21
N-Methyl-L-glutamate
1.4.9.1
Formaldehyde
4.2.1.147
THMPT
4.4.1.22
S-Hydroxy-methyl-glutathione
Formyl-MFR
N5-Formyl-THMPT
2.3.1.101
3.5.4.27
1.5.1.-
1.12.98.2
1.5.98.1
5,10-Methylene-THMPT
1.5.98.2
2.1.2.1
5-Methyl-THMPT
5,10-Methenyl-THMPT
Methanofuran (MFR)
1.2.98.1
1.1.1.284
1.2.1.46
1.12.98.1
Coenzyme F420H2
F420
F420H2
S-Formyl-glutathione
1.2.99.5
3.1.2.12
Acetyl-CoA pathway
1.2.7.4
1.2.99.2
CO
ACDS
Formate
1.2.1.2
CO2
1.2.1.43
Nitrogen metabolism
Acetate
2.7.2.1
Acetylphosphate
2.3.1.8
6.2.1.1
Formaldehyde
Ribulose-P pathway
4.1.2.43
D-Arabino-Hex-3-ulose-6P
D-Xylulose-5P
D-Ribulose-5P
5.3.1.27
2.2.1.3
Pentose phosphate pathway
D-Fructose-6P
Xylulose-P pathway
D-Glyceraldehyde-3P
2.7.1.11
3.1.3.11
Sulfur metabolism
Glycerone
2.7.1.29
Glycerone-P
4.1.2.13
D-Fructose-1,6-P2
Glycine, serine and threonine metabolism
3.1.3.3
2.6.1.52
1.1.1.95
Glycerate-3P
L-Serine
3-Phosphoserine
3-Phospho-oxypyruvate
2.1.2.1
Tetrahydrofolate
Carbon fixation
5,10-Methylene-tetrahydrofolate
5.4.2.11
5.4.2.12
Glycine
Serine pathway
Hydroxy-pyruvate
D-Glycerate
Glyoxylate
2.6.1.45
1.1.1.29
2.7.1.165
Glycerate-2P
Glyoxylate and dicarboxylate metabolism
4.1.3.24
4.2.1.11
6.2.1.9
1.1.1.37
4.1.1.31
Malyl-CoA
L-Malate
Oxaloacetate
Phosphoenol-pyruvate
Acetyl-CoA
1.2.7.1
Pyruvate
2.7.9.2
Glyoxylate and dicarboxylate metabolism
Coenzyme M biosynthesis
Phosphoenol-pyruvate
4.4.1.19
(2R)-3-Sulfolactate
3.1.3.71
1.1.1.337
4.1.1.79
Sulfoacetaldehyde
(2R)-Phosphosulfolactate
3-Sulfopyruvate
Coenzyme M
Coenzyme F420 biosynthesis
Riboflavin metabolism
5-Amino-6-ribitylaminouracil
7,8-Didemethyl-8-hydroxy-5-deazariboflavin
2.5.1.77
2.7.8.28
6.3.2.31
6.3.2.34
Coenzyme F420
6.3.2.32
Coenzyme F420-0
Coenzyme F420-1
4-Hydroxy-phenylpyruvate
2.7.7.68
(2S)-Lactyl-2-diphospho-5'-guanosine
(2S)-2-Phospholactate
Coenzyme F420-3
Coenzyme B biosynthesis
Lysine biosynthesis
(R)-(Homo)2citrate
(-)-threo-Iso(homo)2citrate
(R)-(Homo)3citrate
(-)-threo-Iso(homo)3citrate
7-Oxoheptanoate
7-Mercaptoheptanoyl-threonine
2.3.3.-
4.2.1.114
1.1.1.-
2.7.1.-
2-Oxoadipate
cis-(Homo)2aconitate
2-Oxopimelate
cis-(Homo)3aconitate
2-Oxosuberate
7-Mercaptoheptanoate
Coenzyme B
00680 6/9/15
(c) Kanehisa Laboratories

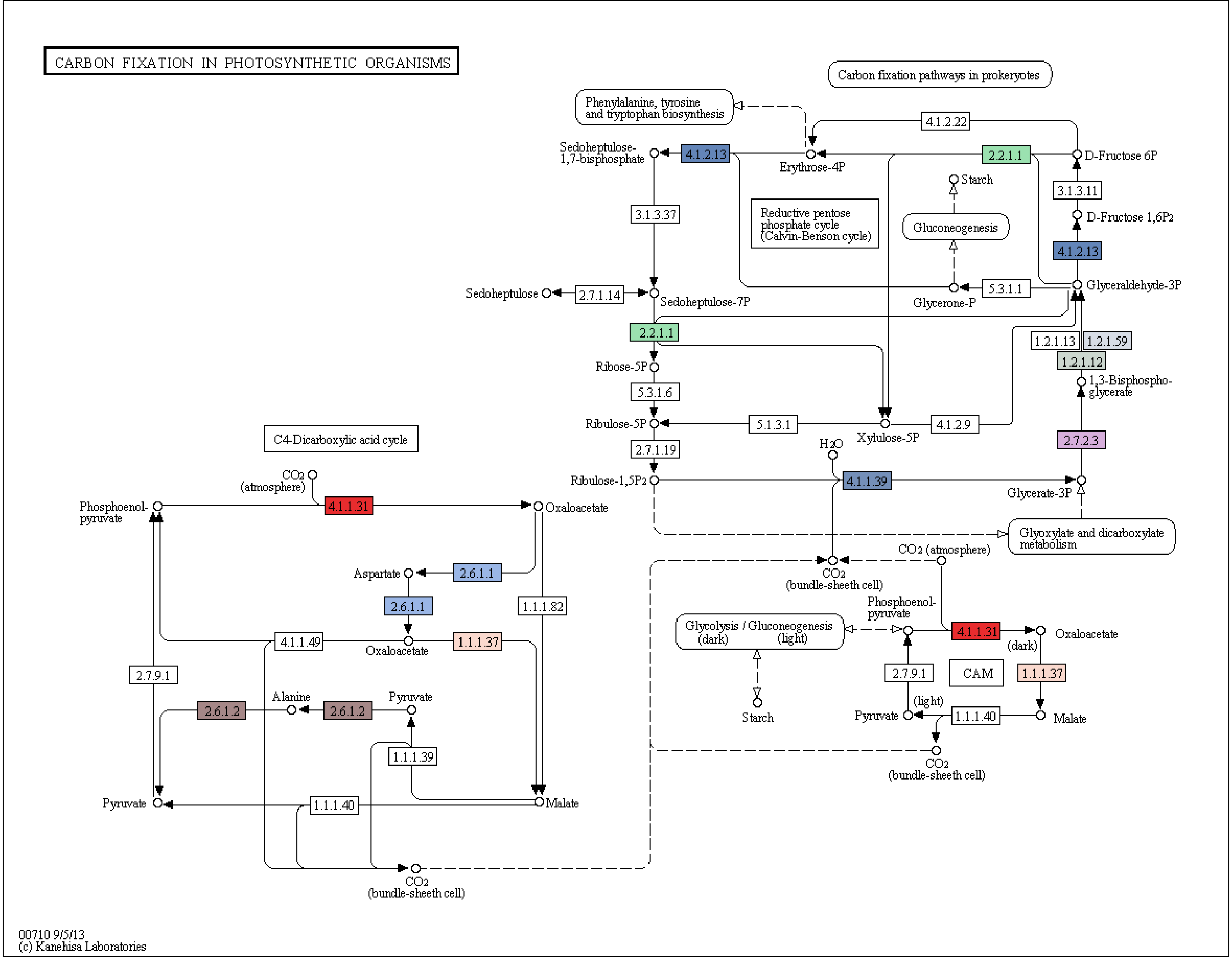
CARBON FIXATION IN PHOTOSYNTHETIC ORGANISMS
Carbon fixation pathways in prokeryotes
Phenylalanine, tyrosine and tryptophan biosynthesis
4.1.2.22
Sedoheptulose-1,7-bisphosphate
4.1.2.13
Erythrose-4P
2.2.1.1
D-Fructose 6P
Starch
3.1.3.11
3.1.3.37
Reductive pentose phosphate cycle (Calvin-Benson cycle)
Gluconeogenesis
D-Fructose 1,6P2
4.1.2.13
Sedoheptulose
2.7.1.14
Sedoheptulose-7P
5.3.1.1
Glyceraldehyde-3P
Glycerone-P
2.2.1.1
1.2.1.13
1.2.1.59
1.2.1.12
Ribose-5P
1,3-Bisphospho-glycerate
5.3.1.6
Ribulose-5P
5.1.3.1
Xylulose-5P
4.1.2.9
C4-Dicarboxylic acid cycle
2.7.1.19
H2O
2.7.2.3
CO2 (atmosphere)
Ribulose-1,5P2
4.1.1.39
Glycerate-3P
Phosphoenol-pyruvate
4.1.1.31
Oxaloacetate
Glyoxylate and dicarboxylate metabolism
CO2 (atmosphere)
CO2 (bundle-sheeth cell)
Aspartate
2.6.1.1
2.6.1.1
1.1.1.82
Phosphoenol-pyruvate
Glycolysis / Gluconeogenesis (dark) (light)
4.1.1.31
Oxaloacetate
(dark)
4.1.1.49
Oxaloacetate
1.1.1.37
2.7.9.1
2.7.9.1
CAM
1.1.1.37
(light)
Alanine
Pyruvate
2.6.1.2
2.6.1.2
Starch
Pyruvate
1.1.1.40
Malate
1.1.1.39
CO2 (bundle-sheeth cell)
Pyruvate
1.1.1.40
Malate
CO2 (bundle-sheeth cell)
00710 9/5/13
(c) Kanehisa Laboratories

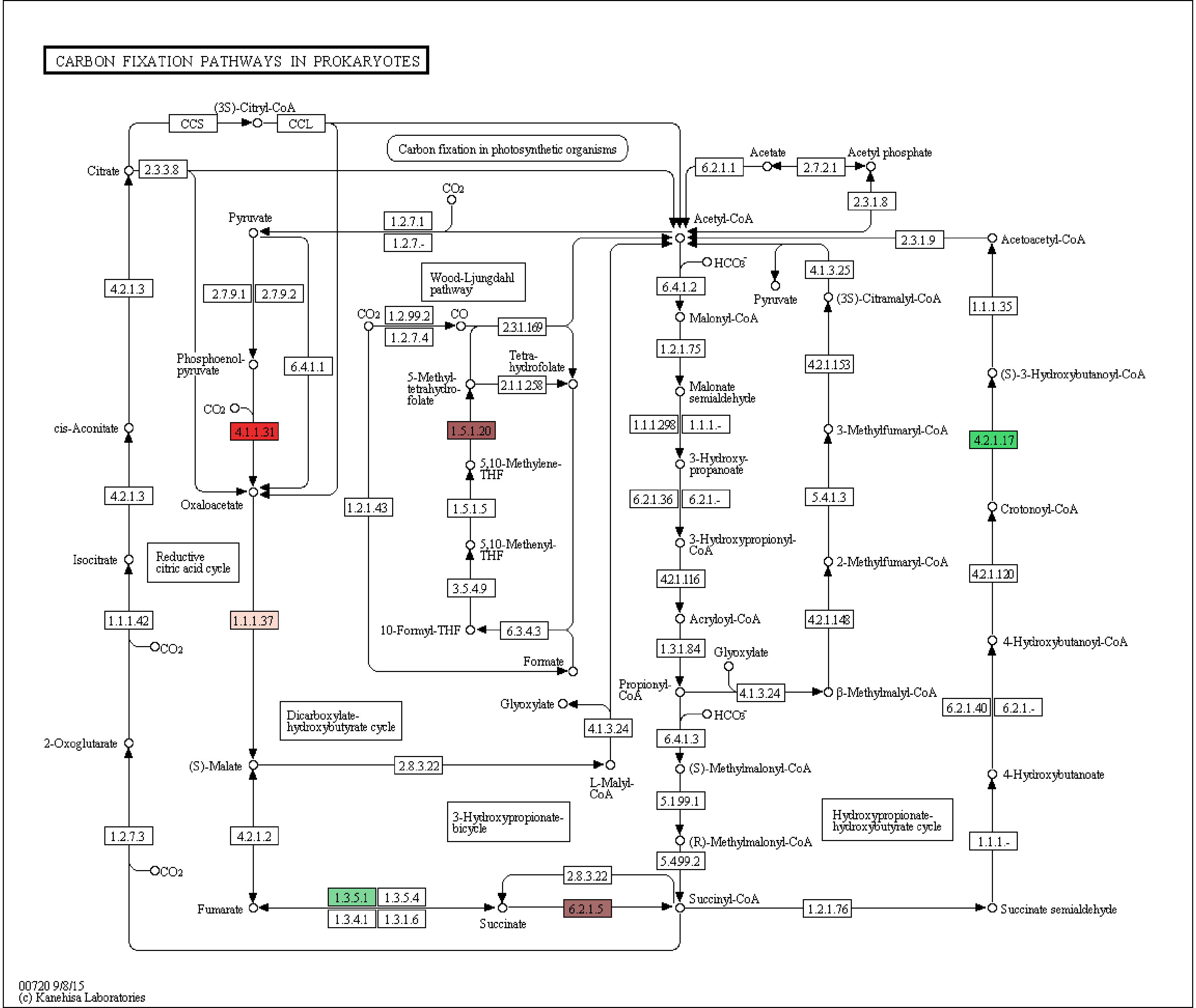
CARBON FIXATION PATHWAYS IN PROKARYOTES
(3S)-Citryl-CoA
CCS
CCL
Carbon fixation in photosynthetic organisms
Citrate
2.3.3.8
Acetate
Acetyl phosphate
6.2.1.1
2.7.2.1
2.3.1.8
CO2
1.2.7.1
1.2.7.-
Pyruvate
Acetyl-CoA
2.3.1.9
Acetoacetyl-CoA
HCO3-
4.2.1.3
2.7.9.1
2.7.9.2
Wood-Ljungdahl pathway
6.4.1.2
Pyruvate
4.1.3.25
(3S)-Citramalyl-CoA
1.1.1.35
Malonyl-CoA
CO2
1.2.99.2
CO
2.3.1.169
1.2.7.4
Phosphoenol-pyruvate
6.4.1.1
Tetra-hydrofolate
1.2.1.75
4.2.1.153
(S)-3-Hydroxybutanoyl-CoA
5-Methyl-tetrahydro-folate
2.1.1.258
Malonate semialdehyde
CO2
cis-Aconitate
4.1.1.31
1.5.1.20
1.1.1.298
1.1.1.-
3-Methylfumaryl-CoA
4.2.1.17
5,10-Methylene-THF
3-Hydroxy-propanoate
4.2.1.3
Oxaloacetate
1.2.1.43
1.5.1.5
6.2.1.36
6.2.1.-
5.4.1.3
Crotonoyl-CoA
3-Hydroxypropionyl-CoA
5,10-Methenyl-THF
Isocitrate
Reductive citric acid cycle
2-Methylfumaryl-CoA
3.5.4.9
4.2.1.116
4.2.1.120
1.1.1.42
1.1.1.37
10-Formyl-THF
6.3.4.3
Acryloyl-CoA
4.2.1.148
CO2
4-Hydroxybutanoyl-CoA
1.3.1.84
Glyoxylate
Formate
Propionyl-CoA
4.1.3.24
β-Methylmalyl-CoA
Glyoxylate
6.2.1.40
6.2.1.-
Dicarboxylate-hydroxybutyrate cycle
HCO3-
4.1.3.24
6.4.1.3
2-Oxoglutarate
(S)-Malate
2.8.3.22
(S)-Methylmalonyl-CoA
L-Malyl-CoA
4-Hydroxybutanoate
3-Hydroxypropionate-bicycle
5.1.99.1
Hydroxypropionate-hydroxybutyrate cycle
1.2.7.3
4.2.1.2
(R)-Methylmalonyl-CoA
1.1.1.-
CO2
5.4.99.2
2.8.3.22
1.3.5.1
1.3.5.4
Succinyl-CoA
Fumarate
6.2.1.5
1.2.1.76
Succinate semialdehyde
1.3.4.1
1.3.1.6
Succinate
00720 9/8/15
(c) Kanehisa Laboratories

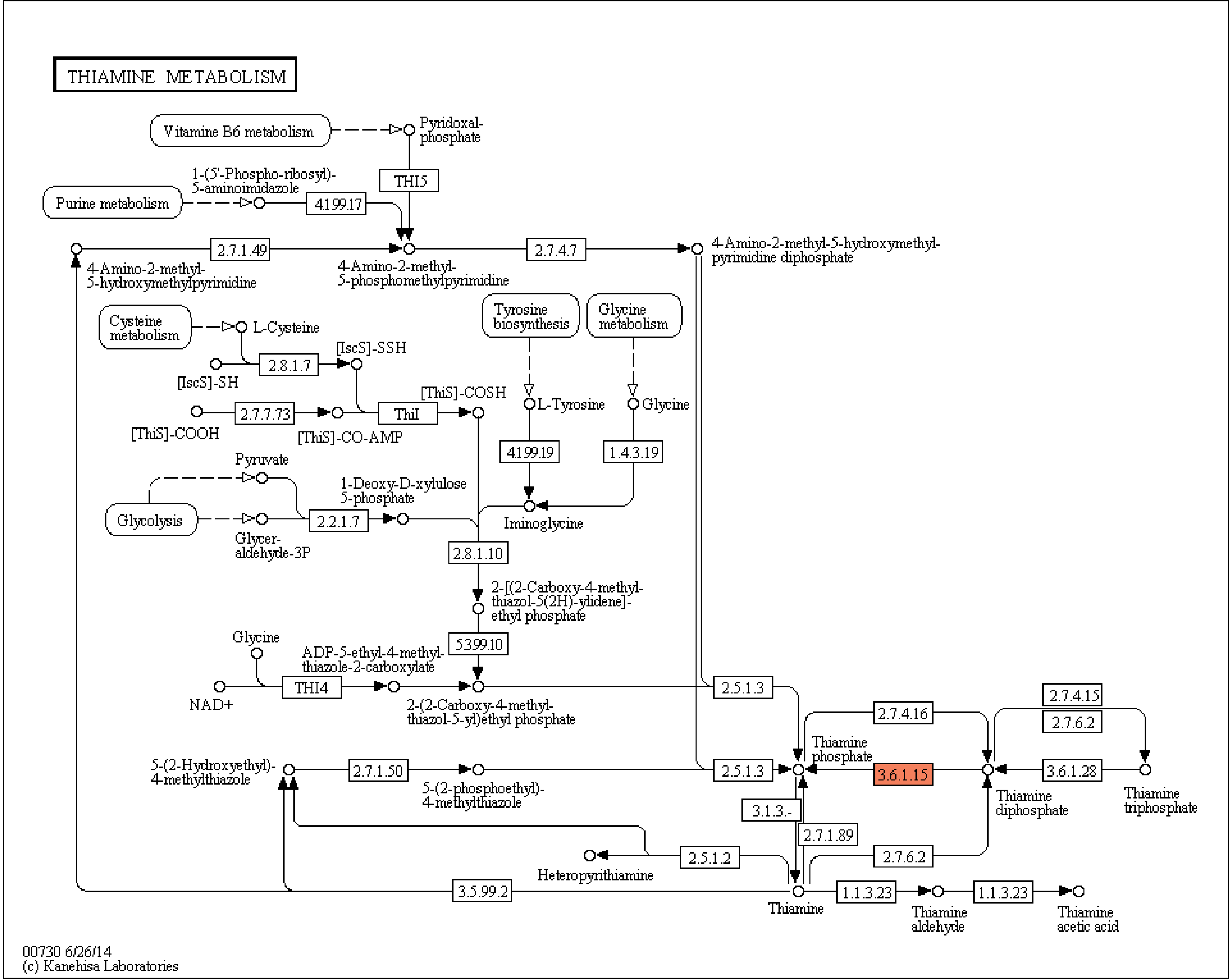
THIAMINE METABOLISM
Vitamine B6 metabolism
Pyridoxal-phosphate
THI5
1-(5'-Phospho-ribosyl)-5-aminoimidazole
Purine metabolism
4.1.99.17
2.7.1.49
2.7.4.7
4-Amino-2-methyl-5-hydroxymethylpyrimidine diphosphate
4-Amino-2-methyl-5-hydroxymethylpyrimidine
4-Amino-2-methyl-5-phosphomethylpyrimidine
Cysteine metabolism
L-Cysteine
Tyrosine biosynthesis
Glycine metabolism
2.8.1.7
[IscS]-SSH
[IscS]-SH
[ThiS]-COSH
2.7.7.73
ThiI
L-Tyrosine
Glycine
[ThiS]-COOH
[ThiS]-CO-AMP
4.1.99.19
1.4.3.19
Pyruvate
1-Deoxy-D-xylulose 5-phosphate
Glycolysis
2.2.1.7
Iminoglycine
Glyceraldehyde-3P
2.8.1.10
2-[(2-Carboxy-4-methyl-thiazol-5(2H)-ylidene]-ethyl phosphate
5.3.99.10
Glycine
ADP-5-ethyl-4-methyl-thiazole-2-carboxylate
NAD+
THI4
2-(2-Carboxy-4-methyl-thiazol-5-yl)ethyl phosphate
2.5.1.3
2.7.4.16
2.7.4.15
2.7.6.2
Thiamine phosphate
5-(2-Hydroxyethyl)-4-methylthiazole
2.7.1.50
2.5.1.3
3.6.1.15
3.6.1.28
5-(2-phosphoethyl)-4-methylthiazole
Thiamine diphosphate
Thiamine triphosphate
3.1.3.-
2.7.1.89
2.5.1.2
2.7.6.2
Heteropyrithiamine
3.5.99.2
1.1.3.23
1.1.3.23
Thiamine
Thiamine aldehyde
Thiamine acetic acid
00730 6/26/14
(c) Kanehisa Laboratories

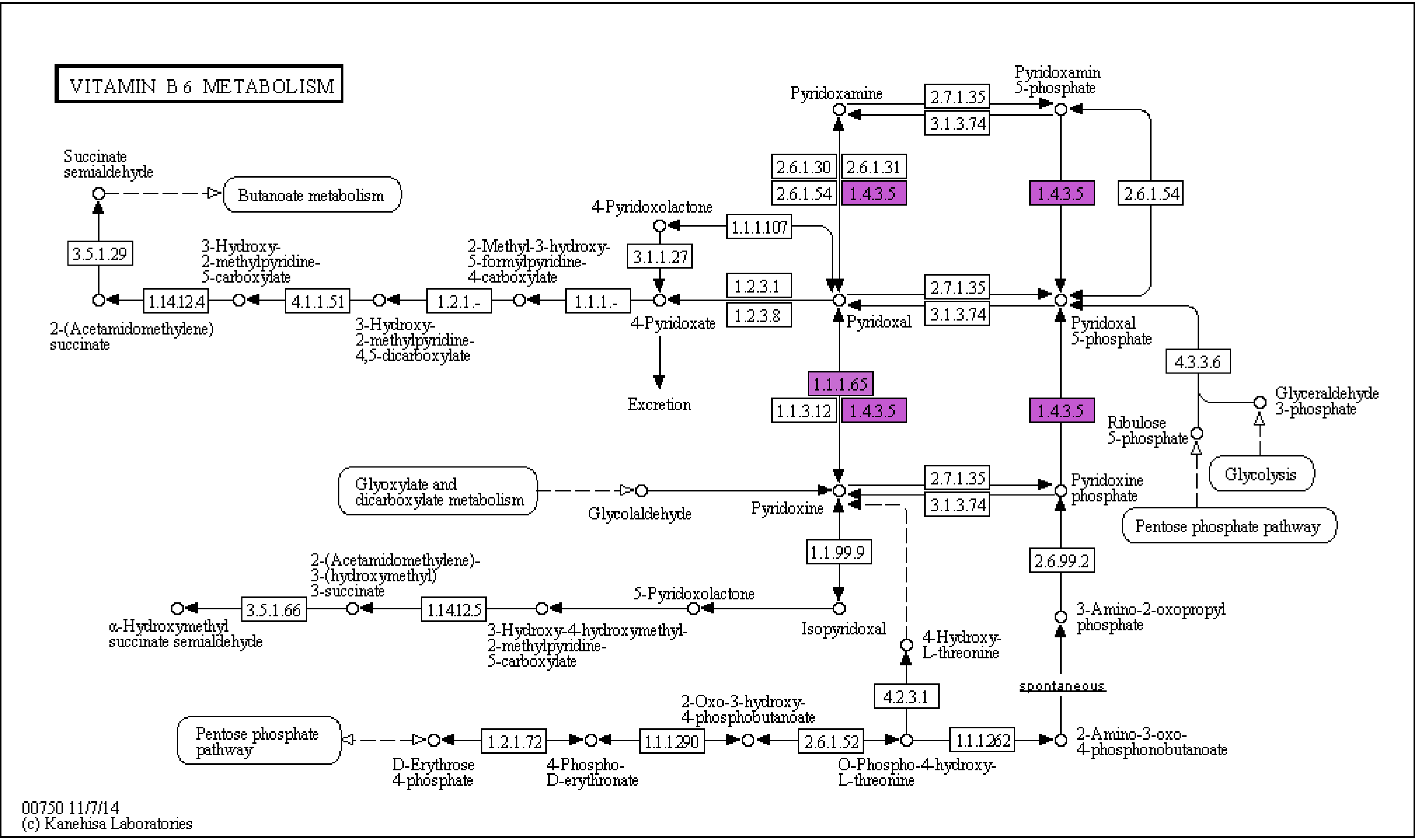

VITAMIN B6 METABOLISM
Succinate semialdehyde
Butanoate metabolism
3.5.1.29
2-(Acetamidomethylene) succinate
1.14.12.4
3-Hydroxy-2-methylpyridine-5-carboxylate
4.1.1.51
3-Hydroxy-2-methylpyridine-4,5-dicarboxylate
1.2.1.-
2-Methyl-3-hydroxy-5-formylpyridine-4-carboxylate
1.1.1.-
4-Pyridoxolactone
3.1.1.27
1.1.1.107
4-Pyridoxate
Excretion
1.2.3.1
1.2.3.8
Pyridoxamine
2.7.1.35
3.1.3.74
Pyridoxamin 5-phosphate
2.6.1.30
2.6.1.31
2.6.1.54
1.4.3.5
1.4.3.5
2.6.1.54
Pyridoxal
2.7.1.35
3.1.3.74
Pyridoxal 5-phosphate
1.1.1.65
1.1.3.12
1.4.3.5
1.4.3.5
4.3.3.6
Glyceraldehyde 3-phosphate
Ribulose 5-phosphate
Glycolysis
Glyoxylate and dicarboxylate metabolism
Glycolaldehyde
Pyridoxine
2.7.1.35
3.1.3.74
Pyridoxine phosphate
Pentose phosphate pathway
1.1.99.9
2.6.99.2
2-(Acetamidomethylene)-3-(hydroxymethyl)3-succinate
5-Pyridoxolactone
α-Hydroxymethyl succinate semialdehyde
3.5.1.66
1.14.12.5
3-Hydroxy-4-hydroxymethyl-2-methylpyridine-5-carboxylate
Isopyridoxal
4-Hydroxy-L-threonine
3-Amino-2-oxopropyl phosphate
4.2.3.1
spontaneous
2-Oxo-3-hydroxy-4-phosphobutanoate
Pentose phosphate pathway
1.2.1.72
1.1.1.290
2.6.1.52
1.1.1.262
D-Erythrose 4-phosphate
4-Phospho-D-erythronate
O-Phospho-4-hydroxy-L-threonine
2-Amino-3-oxo-4-phosphonobutanoate
00750 11/7/14
(c) Kanehisa Laboratories

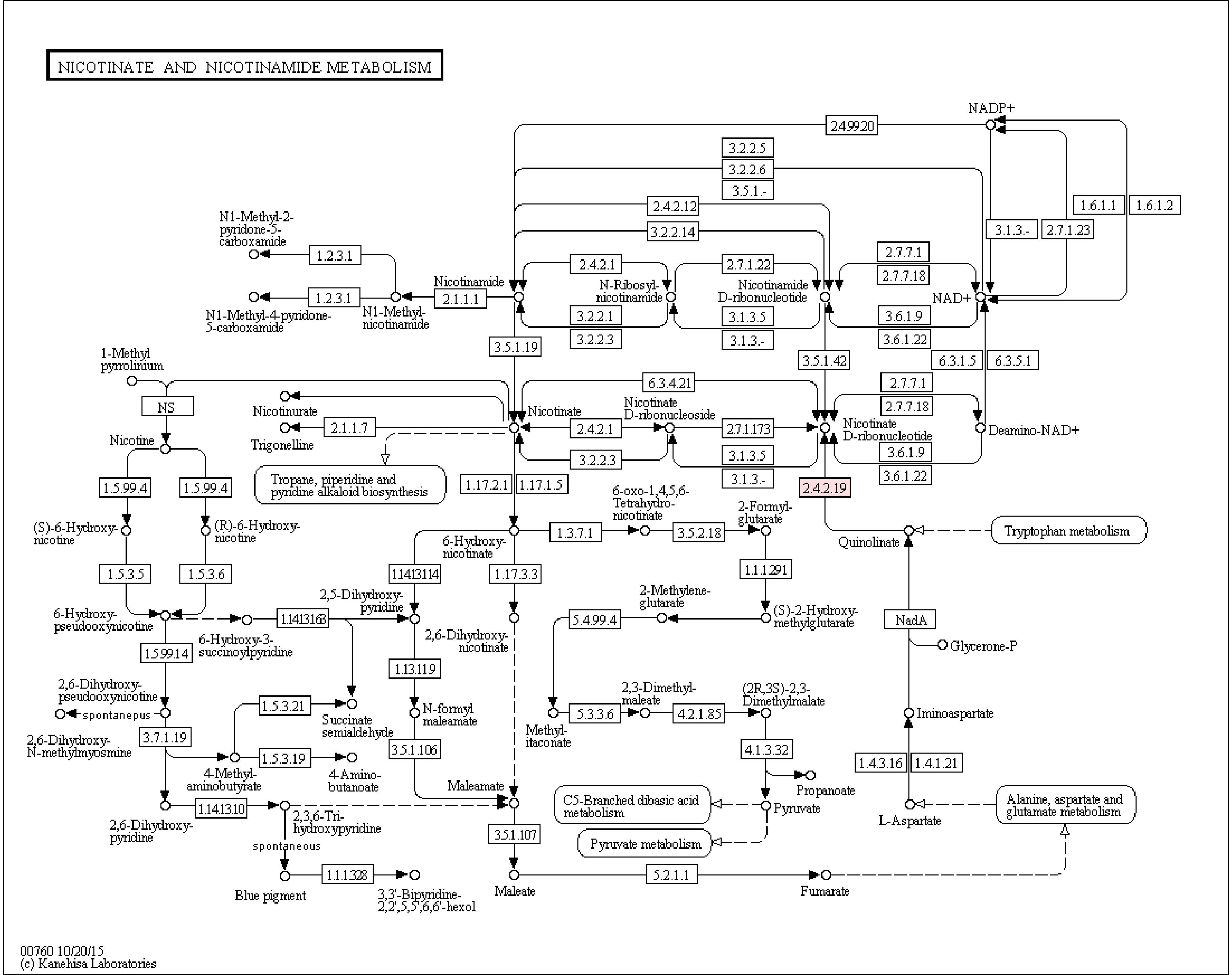
NICOTINATE AND NICOTINAMIDE METABOLISM
NADP+
2.4.99.20
3.2.2.5
3.2.2.6
3.5.1.-
2.4.2.12
3.2.2.14
1.6.1.1
1.6.1.2
3.1.3.-
2.7.1.23
N1-Methyl-2-pyridone-5-carboxamide
1.2.3.1
1.2.3.1
N1-Methyl-4-pyridone-5-carboxamide
N1-Methyl-nicotinamide
2.1.1.1
Nicotinamide
2.4.2.1
N-Ribosyl-nicotinamide
2.7.1.22
Nicotinamide D-ribonucleotide
2.7.7.1
2.7.7.18
NAD+
3.2.2.1
3.2.2.3
3.1.3.5
3.1.3.-
3.6.1.9
3.6.1.22
3.5.1.19
3.5.1.42
6.3.1.5
6.3.5.1
1-Methyl pyrrolinium
NS
Nicotinurate
Trigonelline
2.1.1.7
Nicotine
6.3.4.21
Nicotinate
Nicotinate D-ribonucleoside
2.4.2.1
2.7.1.173
2.7.7.1
2.7.7.18
Nicotinate D-ribonucleotide
Deamino-NAD+
3.2.2.3
3.1.3.5
3.1.3.-
3.6.1.9
3.6.1.22
Tropane, piperidine and pyridine alkaloid biosynthesis
1.5.99.4
1.5.99.4
1.17.2.1
1.17.1.5
6-oxo-1,4,5,6-Tetrahydro-nicotinate
2-Formyl-glutarate
2.4.2.19
(S)-6-Hydroxy-nicotine
(R)-6-Hydroxy-nicotine
6-Hydroxy-nicotinate
1.3.7.1
3.5.2.18
Quinolinate
Tryptophan metabolism
1.5.3.5
1.5.3.6
1.14.13.114
1.17.3.3
1.1.1.291
6-Hydroxy-pseudooxynicotine
2,5-Dihydroxy-pyridine
1.14.13.163
2-Methylene-glutarate
5.4.99.4
(S)-2-Hydroxy-methylglutarate
NadA
6-Hydroxy-3-succinoylpyridine
2,6-Dihydroxy-nicotinate
Glycerone-P
1.5.99.14
1.13.11.9
2,6-Dihydroxy-pseudooxynicotine
spontaneous
2,3-Dimethyl-maleate
(2R,3S)-2,3-Dimethylmalate
1.5.3.21
Succinate semialdehyde
N-formyl maleamate
Iminoaspartate
2,6-Dihydroxy-N-methylmyosmine
3.7.1.19
Methyl-itaconate
5.3.3.6
4.2.1.85
1.5.3.19
4.1.3.32
1.4.3.16
1.4.1.21
4-Methyl-aminobutyrate
4-Amino-butanoate
3.5.1.106
Propanoate
Maleamate
C5-Branched dibasic acid metabolism
Pyruvate
L-Aspartate
Alanine, aspartate and glutamate metabolism
2,6-Dihydroxy-pyridine
1.14.13.10
2,3,6-Tri-hydroxypyridine
3.5.1.107
Pyruvate metabolism
spontaneous
Blue pigment
1.1.1.328
3,3'-Bipyridine-2,2',5,5',6,6'-hexol
Maleate
5.2.1.1
Fumarate
00760 10/20/15
(c) Kanehisa Laboratories

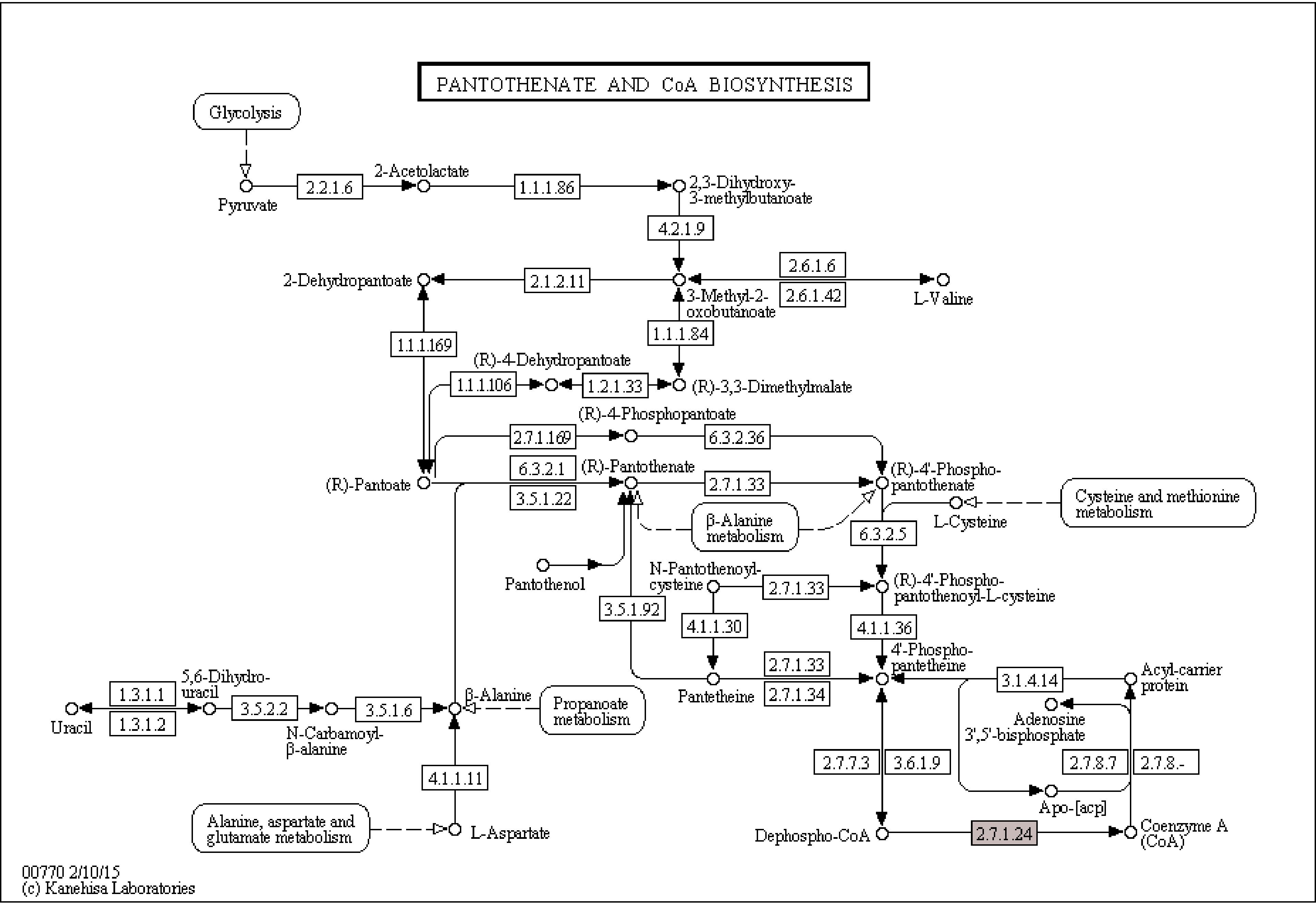
PANTOTHENATE AND CoA BIOSYNTHESIS
Glycolysis
Pyruvate
2.2.1.6
2-Acetolactate
1.1.1.86
2,3-Dihydroxy-
3-methylbutanoate
4.2.1.9
2-Dehydropantoate
2.1.2.11
2.6.1.6
2.6.1.42
L-Valine
3-Methyl-2-
oxobutanoate
1.1.1.169
1.1.1.84
(R)-4-Dehydropantoate
1.1.1.106
1.2.1.33
(R)-3,3-Dimethylmalate
(R)-4-Phosphopantoate
2.7.1.169
6.3.2.36
6.3.2.1
(R)-Pantothenate
(R)-Pantoate
3.5.1.22
2.7.1.33
(R)-4'-Phospho-
pantothenate
Cysteine and methionine
metabolism
β-Alanine
metabolism
L-Cysteine
6.3.2.5
Pantothenol
N-Pantothenoyl-
cysteine
2.7.1.33
(R)-4'-Phospho-
pantothenoyl-L-cysteine
3.5.1.92
4.1.1.30
4.1.1.36
4'-Phospho-
pantetheine
2.7.1.33
2.7.1.34
Pantetheine
3.1.4.14
Acyl-carrier
protein
1.3.1.1
1.3.1.2
Uracil
5,6-Dihydro-
uracil
3.5.2.2
N-Carbamoyl-
β-alanine
3.5.1.6
β-Alanine
Propanoate
metabolism
Adenosine
3',5'-bisphosphate
2.7.7.3
3.6.1.9
2.7.8.7
2.7.8.-
Apo-[acp]
4.1.1.11
Alanine, aspartate and
glutamate metabolism
L-Aspartate
Dephospho-CoA
2.7.1.24
Coenzyme A
(CoA)
00770 2/10/15
(c) Kanehisa Laboratories

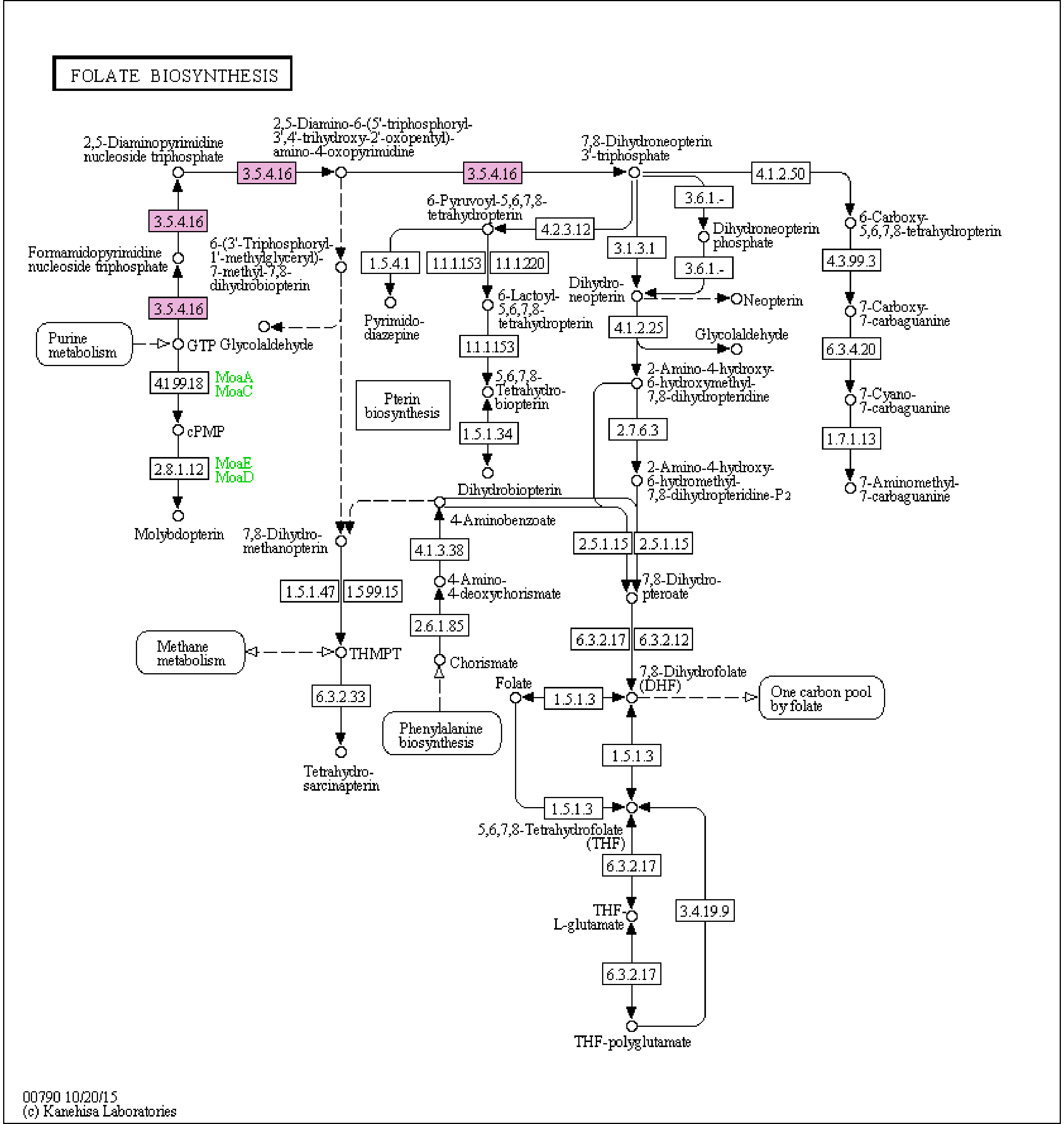
FOLATE BIOSYNTHESIS
2,5-Diaminopyrimidine nucleoside triphosphate
3.5.4.16
2,5-Diamino-6-(5'-triphosphoryl-3',4'-trihydroxy-2'-oxopentyl)-amino-4-oxopyrimidine
3.5.4.16
7,8-Dihydroneopterin 3'-triphosphate
4.1.2.50
3.6.1.-
6-Carboxy-5,6,7,8-tetrahydropterin
Dihydroneopterin phosphate
4.3.99.3
3.6.1.-
3.1.3.1
6-Pyruvoyl-5,6,7,8-tetrahydropterin
4.2.3.12
3.5.4.16
Formamidopyrimidine nucleoside triphosphate
6-(3'-Triphosphoryl-1'-methylglyceryl)-7-methyl-7,8-dihydrobiopterin
1.5.4.1
1.1.1.153
1.1.1.220
Dihydro-neopterin
Neopterin
7-Carboxy-7-carbaguanine
3.5.4.16
Pyrimido-diazepine
6-Lactoyl-5,6,7,8-tetrahydropterin
4.1.2.25
Purine metabolism
GTP Glycolaldehyde
Glycolaldehyde
6.3.4.20
1.1.1.153
4.1.99.18
MoaA
MoaC
2-Amino-4-hydroxy-6-hydroxymethyl-7,8-dihydropteridine
Pterin biosynthesis
5,6,7,8-Tetrahydro-biopterin
7-Cyano-7-carbaguanine
cPMP
1.5.1.34
2.7.6.3
2.8.1.12
MoaE
MoaD
1.7.1.13
2-Amino-4-hydroxy-6-hydromethyl-7,8-dihydropteridine-P2
7-Aminomethyl-7-carbaguanine
Dihydrobiopterin
4-Aminobenzoate
Molybdopterin
7,8-Dihydro-methanopterin
4.1.3.38
2.5.1.15
2.5.1.15
4-Amino-4-deoxychorismate
7,8-Dihydro-pteroate
1.5.1.47
1.5.99.15
2.6.1.85
6.3.2.17
6.3.2.12
Methane metabolism
THMPT
Chorismate
7,8-Dihydrofolate (DHF)
Folate
6.3.2.33
1.5.1.3
One carbon pool by folate
Phenylalanine biosynthesis
1.5.1.3
Tetrahydro-sarcinapterin
1.5.1.3
5,6,7,8-Tetrahydrofolate (THF)
6.3.2.17
THF-L-glutamate
3.4.19.9
6.3.2.17
THF-polyglutamate
00790 10/20/15
(c) Kanehisa Laboratories

PORPHYRIN AND CHLOROPHYLL METABOLISM

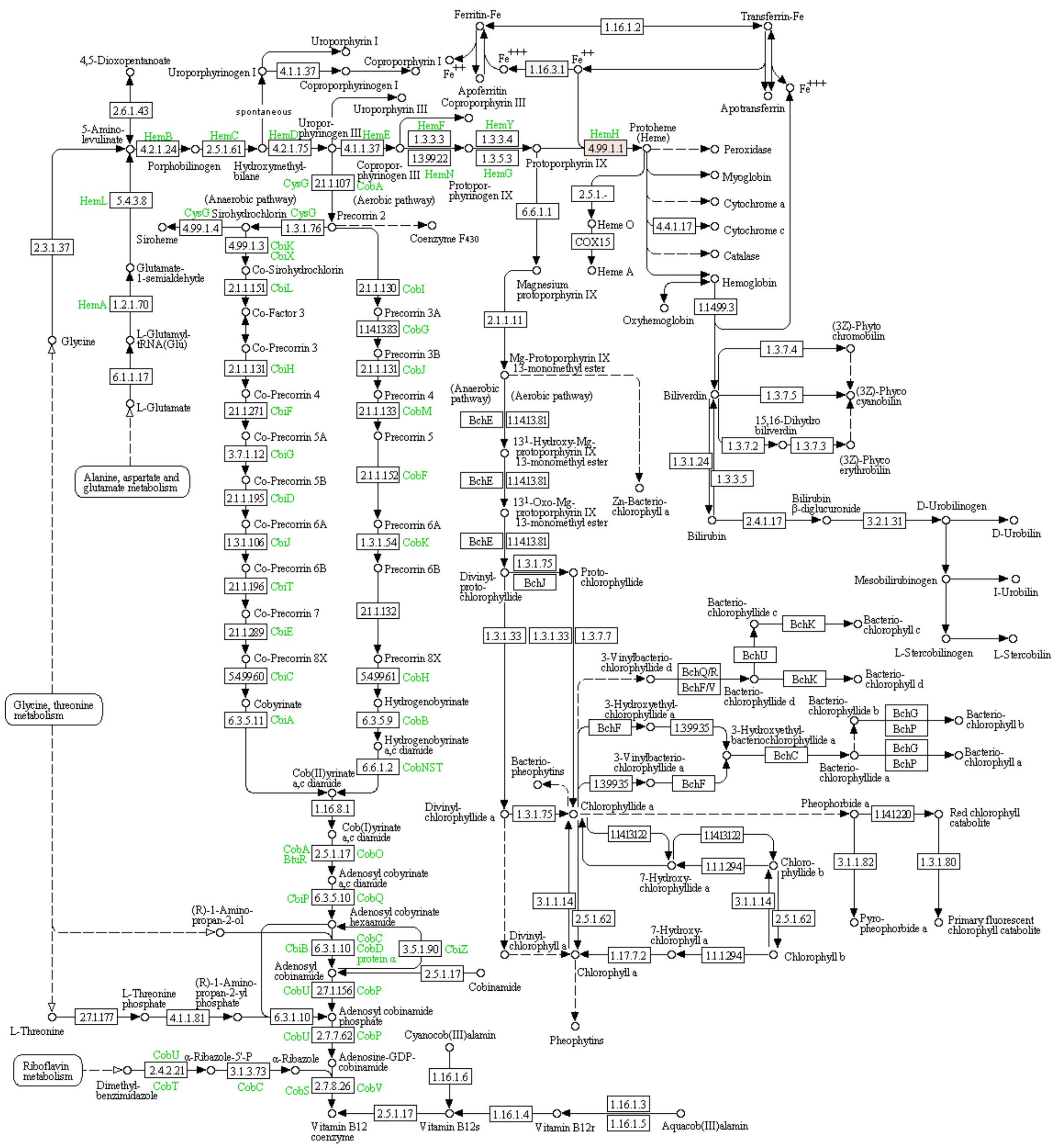

00860 4/20/15
(c) Kanehisa Laboratories

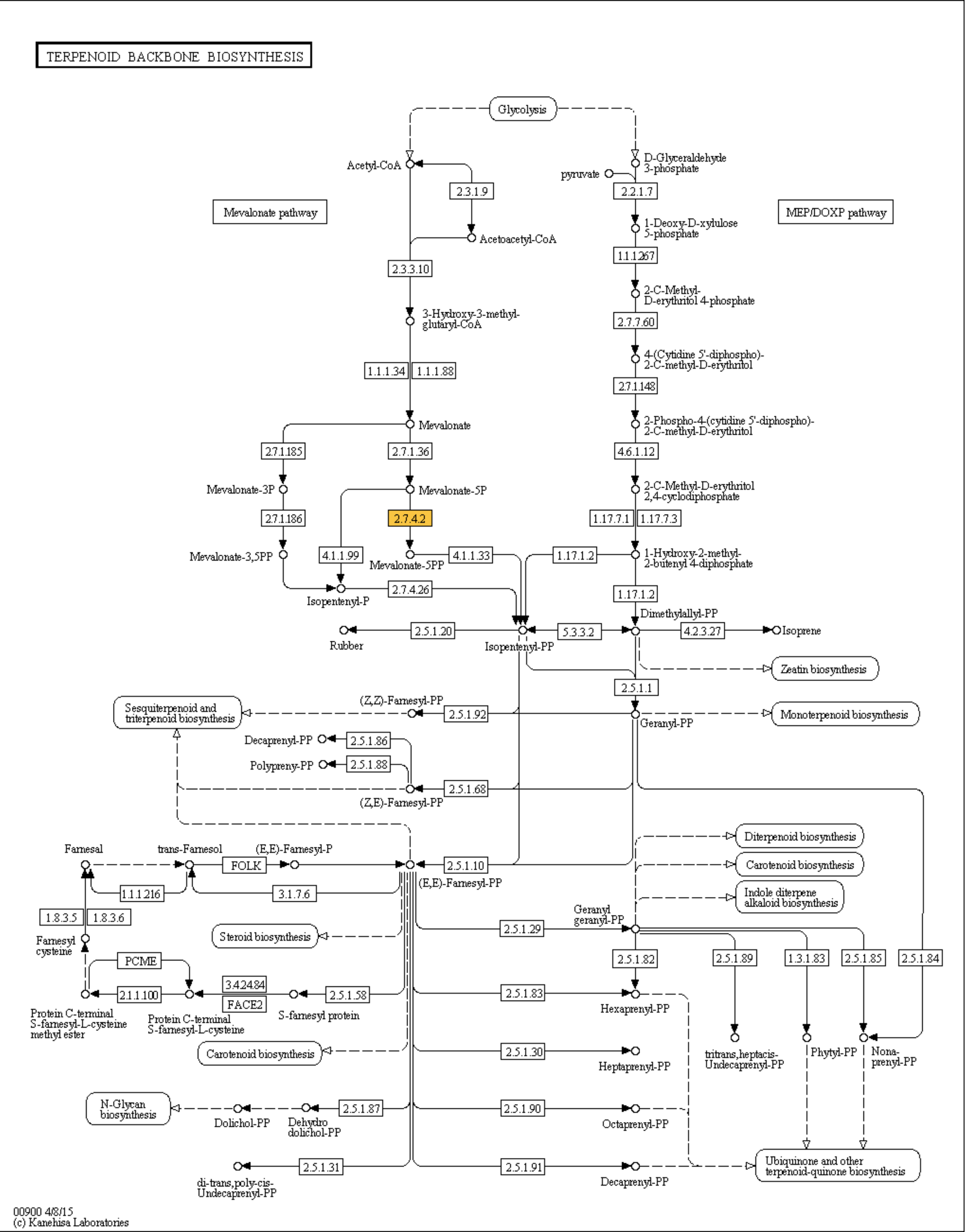

TERPENOID BACKBONE BIOSYNTHESIS
Glycolysis
Acetyl-CoA
2.3.1.9
Acetoacetyl-CoA
2.3.3.10
3-Hydroxy-3-methyl-glutaryl-CoA
1.1.1.34
1.1.1.88
Mevalonate
Mevalonate pathway
MEP/DOXP pathway
D-Glyceraldehyde 3-phosphate
pyruvate
2.2.1.7
1-Deoxy-D-xylulose 5-phosphate
1.1.1.267
2-C-Methyl-D-erythritol 4-phosphate
2.7.7.60
4-(Cytidine 5'-diphospho)-2-C-methyl-D-erythritol
2.7.1.148
2-Phospho-4-(cytidine 5'-diphospho)-2-C-methyl-D-erythritol
4.6.1.12
2-C-Methyl-D-erythritol 2,4-cyclodiphosphate
1.17.7.1
1.17.7.3
1-Hydroxy-2-methyl-2-butenyl 4-diphosphate
1.17.1.2
2.7.1.185
2.7.1.36
Mevalonate-3P
Mevalonate-5P
2.7.1.186
2.7.4.2
Mevalonate-3,5PP
4.1.1.99
Mevalonate-5PP
4.1.1.33
Isopentenyl-P
2.7.4.26
Dimethylallyl-PP
Rubber
2.5.1.20
Isopentenyl-PP
5.3.3.2
4.2.3.27
Isoprene
Zeatin biosynthesis
2.5.1.1
Sesquiterpenoid and triterpenoid biosynthesis
(Z,Z)-Farnesyl-PP
2.5.1.92
Geranyl-PP
Monoterpenoid biosynthesis
Decaprenyl-PP
2.5.1.86
Polypreny-PP
2.5.1.88
2.5.1.68
(Z,E)-Farnesyl-PP
Diterpenoid biosynthesis
Carotenoid biosynthesis
Indole diterpene alkaloid biosynthesis
Farnesal
trans-Farnesol
(E,E)-Farnesyl-P
FOLK
2.5.1.10
(E,E)-Farnesyl-PP
1.1.1.216
3.1.7.6
1.8.3.5
1.8.3.6
Farnesyl cysteine
Steroid biosynthesis
2.5.1.29
Geranyl geranyl-PP
PCME
3.4.24.84
FACE2
2.1.1.100
2.5.1.58
Protein C-terminal S-farnesyl-L-cysteine methyl ester
Protein C-terminal S-farnesyl-L-cysteine
S-farnesyl protein
2.5.1.83
2.5.1.82
2.5.1.89
1.3.1.83
2.5.1.85
2.5.1.84
Hexaprenyl-PP
tritrans,heptacis-Undecaprenyl-PP
Phytyl-PP
Nona-prenyl-PP
Carotenoid biosynthesis
2.5.1.30
Heptaprenyl-PP
N-Glycan biosynthesis
Dolichol-PP
Dehydro dolichol-PP
2.5.1.87
2.5.1.90
Octaprenyl-PP
2.5.1.31
di-trans,poly-cis-Undecaprenyl-PP
2.5.1.91
Decaprenyl-PP
Ubiquinone and other terpenoid-quinone biosynthesis
00900 4/8/15
(c) Kanehisa Laboratories

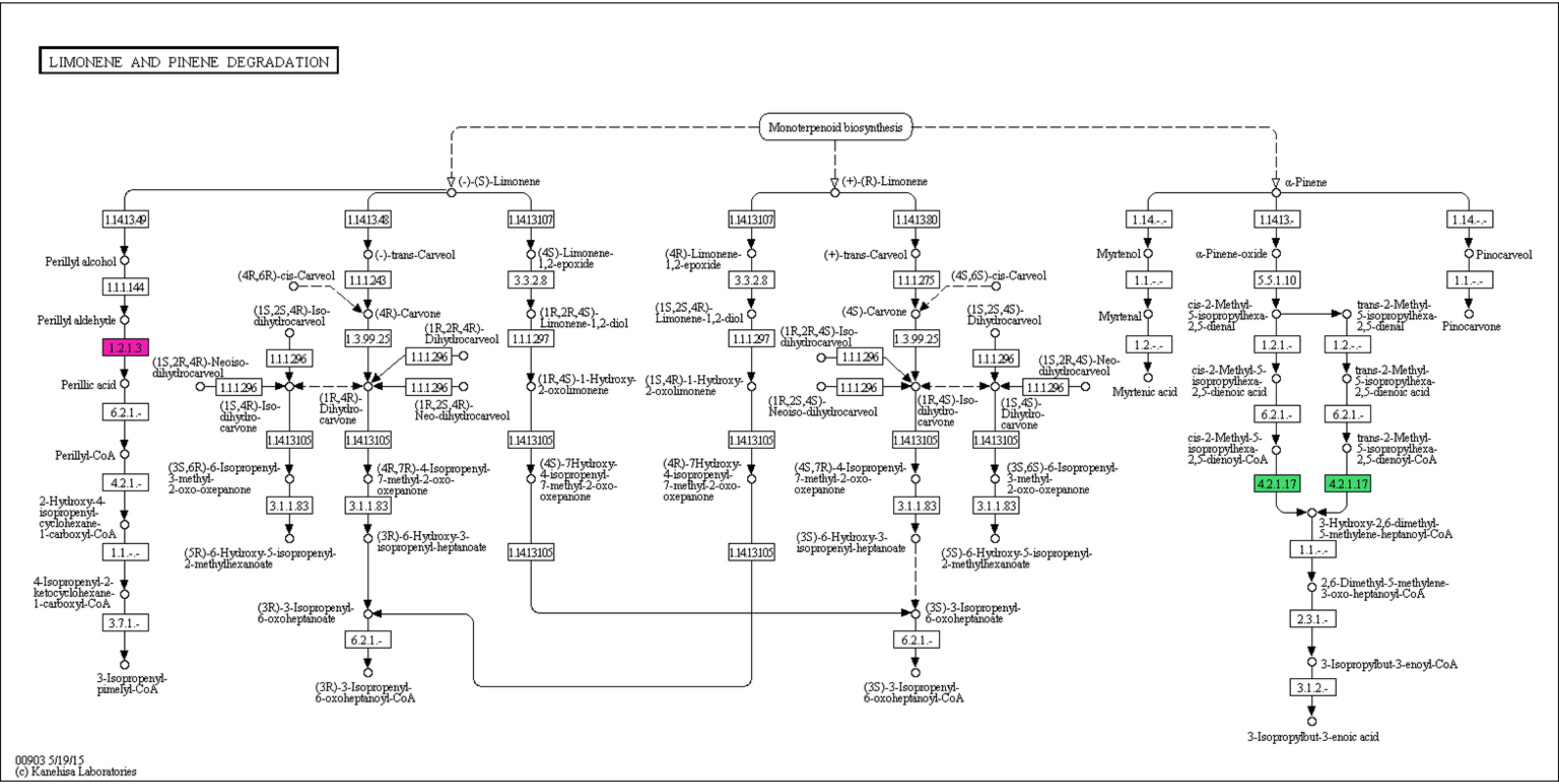
LIMONENE AND PINENE DEGRADATION
Monoterpenoid biosynthesis
(-)-(S)-Limonene
(+)-(R)-Limonene
α-Pinene
1.14.13.49
Perillyl alcohol
1.1.1.144
Perillyl aldehyde
1.2.1.3
Perillic acid
6.2.1.-
Perillyl-CoA
4.2.1.-
2-Hydroxy-4-isopropenyl-cyclohexane-1-carboxyl-CoA
1.1.-.-
4-Isopropenyl-2-ketocyclohexane-1-carboxyl-CoA
3.7.1.-
3-Isopropenyl-pimelyl-CoA
1.14.13.48
(-)-trans-Carveol
(4R,6R)-cis-Carveol
1.1.1.243
(4R)-Carvone
(1S,2S,4R)-Iso-dihydrocarveol
1.3.99.25
(1R,2R,4R)-Dihydrocarveol
1.1.1.296
(1S,2R,4R)-Neoiso-dihydrocarveol
(1S,4R)-Iso-dihydro-carvone
(1R,4R)-Dihydro-carvone
(1R,2S,4R)-Neo-dihydrocarveol
1.14.13.105
(3S,6R)-6-Isopropenyl-3-methyl-2-oxo-oxepanone
3.1.1.83
(5R)-6-Hydroxy-5-isopropenyl-2-methylhexanoate
(4R,7R)-4-Isopropenyl-7-methyl-2-oxo-oxepanone
(3R)-6-Hydroxy-3-isopropenyl-heptanoate
(3R)-3-Isopropenyl-6-oxoheptanoate
6.2.1.-
(3R)-3-Isopropenyl-6-oxoheptanoyl-CoA
1.14.13.107
(4S)-Limonene-1,2-epoxide
3.3.2.8
(1R,2R,4S)-Limonene-1,2-diol
1.1.1.297
(1R,4S)-1-Hydroxy-2-oxolimonene
(4S)-7Hydroxy-4-isopropenyl-7-methyl-2-oxo-oxepanone
(4R)-Limonene-1,2-epoxide
(1S,2S,4R)-Limonene-1,2-diol
(1S,4R)-1-Hydroxy-2-oxolimonene
(4R)-7Hydroxy-4-isopropenyl-7-methyl-2-oxo-oxepanone
1.14.13.80
(+)-trans-Carveol
(4S,6S)-cis-Carveol
1.1.1.275
(4S)-Carvone
(1S,2S,4S)-Dihydrocarveol
(1R,2R,4S)-Iso-dihydrocarveol
(1S,2R,4S)-Neo-dihydrocarveol
(1R,2S,4S)-Neoiso-dihydrocarveol
(1R,4S)-Iso-dihydro-carvone
(1S,4S)-Dihydro-carvone
(4S,7R)-4-Isopropenyl-7-methyl-2-oxo-oxepanone
(3S,6S)-6-Isopropenyl-3-methyl-2-oxo-oxepanone
(3S)-6-Hydroxy-3-isopropenyl-heptanoate
(5S)-6-Hydroxy-5-isopropenyl-2-methylhexanoate
(3S)-3-Isopropenyl-6-oxoheptanoate
(3S)-3-Isopropenyl-6-oxoheptanoyl-CoA
1.14.-.-
Myrtenol
1.1.-.-
Myrtenal
1.2.-.-
Myrtenic acid
1.14.13.-
α-Pinene-oxide
5.5.1.10
cis-2-Methyl-5-isopropylhexa-2,5-dienal
trans-2-Methyl-5-isopropylhexa-2,5-dienal
1.2.1.-
cis-2-Methyl-5-isopropylhexa-2,5-dienoic acid
trans-2-Methyl-5-isopropylhexa-2,5-dienoic acid
cis-2-Methyl-5-isopropylhexa-2,5-dienoyl-CoA
trans-2-Methyl-5-isopropylhexa-2,5-dienoyl-CoA
4.2.1.17
3-Hydroxy-2,6-dimethyl-5-methylene-heptanoyl-CoA
2,6-Dimethyl-5-methylene-3-oxo-heptanoyl-CoA
2.3.1.-
3-Isopropylbut-3-enoyl-CoA
3.1.2.-
3-Isopropylbut-3-enoic acid
Pinocarveol
Pinocarvone
00903 5/19/15
(c) Kanehisa Laboratories

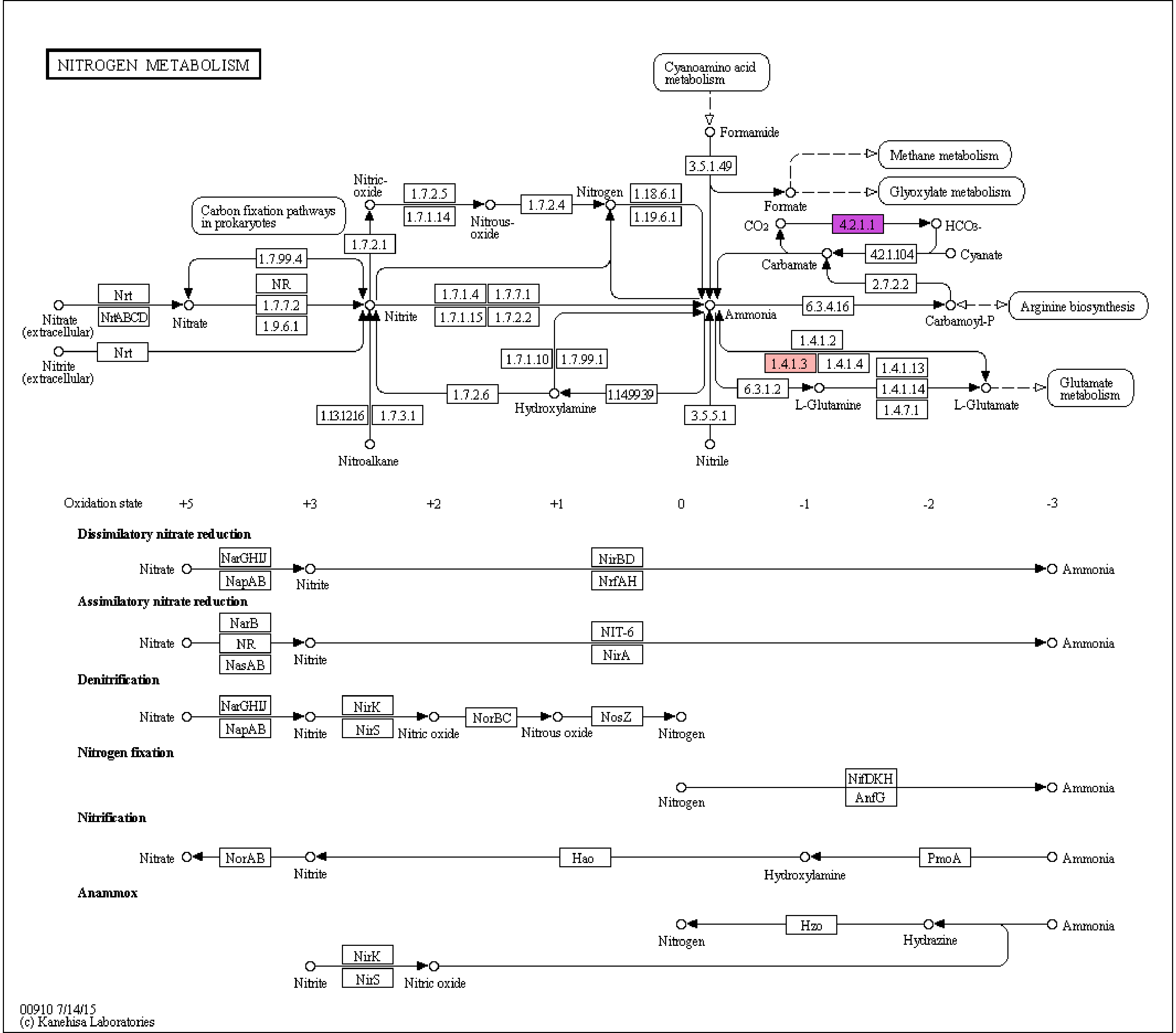

NITROGEN METABOLISM
Cyanoamino acid metabolism
Formamide
3.5.1.49
Methane metabolism
Glyoxylate metabolism
Formate
Carbon fixation pathways in prokaryotes
Nitric-oxide
1.7.2.5
1.7.1.14
Nitrous-oxide
1.7.2.4
Nitrogen
1.18.6.1
1.19.6.1
CO_2
4.2.1.1
HCO_3-
Carbamate
4.2.1.104
Cyanate
1.7.2.1
1.7.99.4
NR
2.7.2.2
Nrt
NrtABCD
Nitrate (extracellular)
Nitrate
1.7.7.2
1.9.6.1
Nitrite
1.7.1.4
1.7.7.1
1.7.1.15
1.7.2.2
Ammonia
6.3.4.16
Carbamoyl-P
Arginine biosynthesis
Nitrite (extracellular)
1.4.1.2
1.4.1.3
1.4.1.4
1.4.1.13
1.4.1.14
1.4.7.1
1.7.1.10
1.7.99.1
6.3.1.2
L-Glutamine
L-Glutamate
Glutamate metabolism
1.7.2.6
Hydroxylamine
1.14.99.39
1.13.12.16
1.7.3.1
3.5.5.1
Nitroalkane
Nitrile
Oxidation state +5 +3 +2 +1 0 -1 -2 -3
Dissimilatory nitrate reduction
Nitrate NarGHIJ NapAB Nitrite NirBD NrfAH Ammonia
Assimilatory nitrate reduction
Nitrate NarB NR NasAB Nitrite NIT-6 NirA Ammonia
Denitrification
Nitrate NarGHIJ NapAB Nitrite NirK NirS Nitric oxide NorBC Nitrous oxide NosZ Nitrogen
Nitrogen fixation
Nitrogen NifDKH AnfG Ammonia
Nitrification
Nitrate NorAB Nitrite Hao Hydroxylamine PmoA Ammonia
Anammox
Nitrogen Hzo Hydrazine Ammonia
Nitrite NirK NirS Nitric oxide
00910 7/14/15
(c) Kanehisa Laboratories

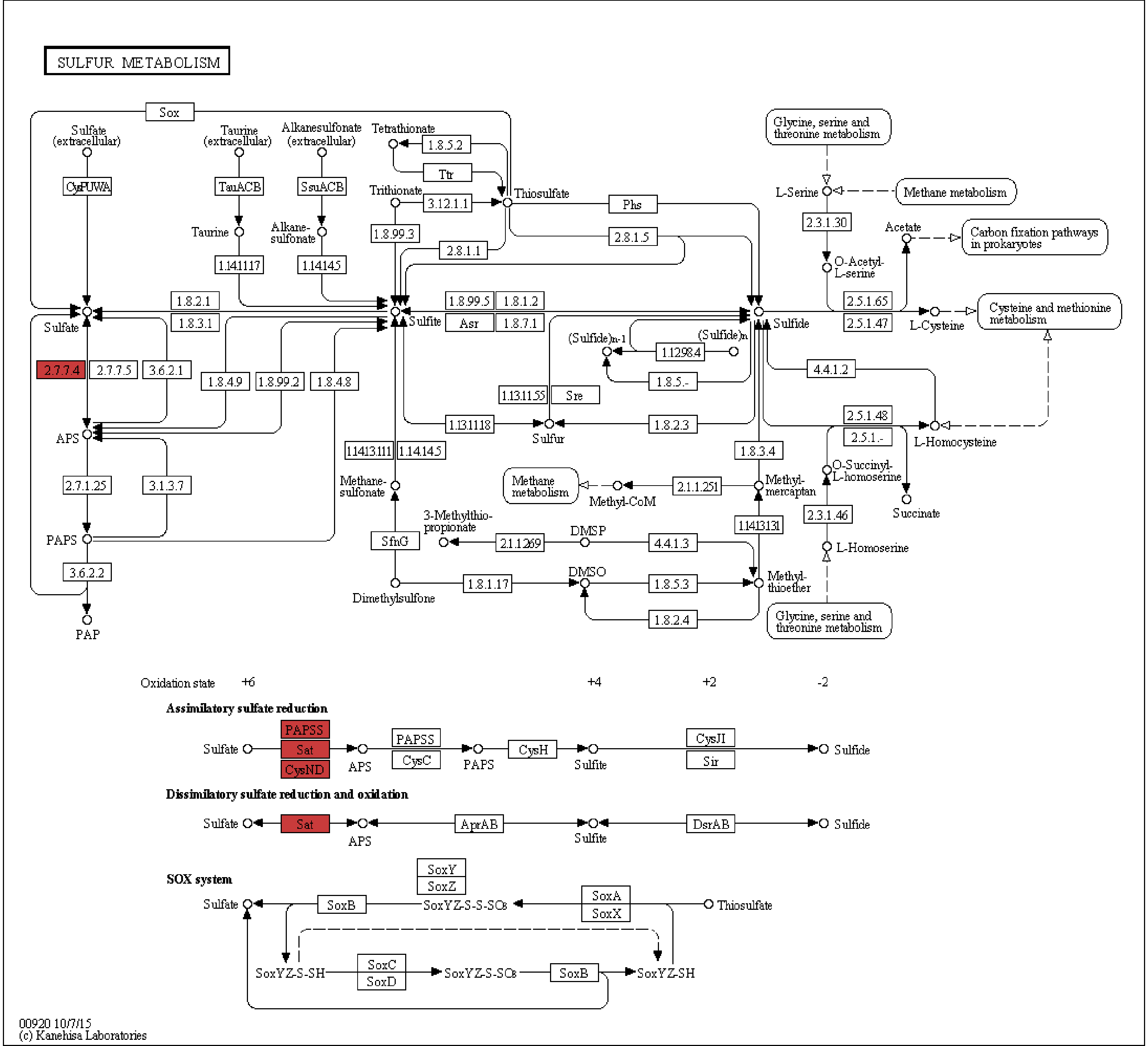
SULFUR METABOLISM
Sox
Sulfate (extracellular)
Taurine (extracellular)
Alkanesulfonate (extracellular)
Tetrathionate
1.8.5.2
Ttr
CysPUWA
TauACB
SsuACB
Trithionate
3.12.1.1
Thiosulfate
Phs
2.8.1.5
Taurine
Alkane-sulfonate
1.8.99.3
2.8.1.1
1.14.11.17
1.14.14.5
Glycine, serine and threonine metabolism
L-Serine
Methane metabolism
2.3.1.30
Acetate
Carbon fixation pathways in prokaryotes
O-Acetyl-L-serine
1.8.2.1
1.8.3.1
Sulfate
1.8.99.5
1.8.1.2
Asr
1.8.7.1
Sulfite
Sulfide
2.5.1.65
2.5.1.47
L-Cysteine
Cysteine and methionine metabolism
(Sulfide)n-1
(Sulfide)n
1.12.98.4
1.8.5.-
4.4.1.2
2.7.7.4
2.7.7.5
3.6.2.1
1.8.4.9
1.8.99.2
1.8.4.8
1.13.11.55
Sre
APS
1.13.11.18
Sulfur
1.8.2.3
2.5.1.48
L-Homocysteine
2.5.1.-
1.14.13.111
1.14.14.5
1.8.3.4
O-Succinyl-L-homoserine
Methane-sulfonate
Methane metabolism
Methyl-CoM
2.1.1.251
Methyl-mercaptan
2.7.1.25
3.1.3.7
Succinate
3-Methylthio-propionate
1.14.13.131
2.3.1.46
PAPS
SfnG
2.1.1.269
DMSP
4.4.1.3
L-Homoserine
3.6.2.2
DMSO
Methyl-thioether
1.8.1.17
1.8.5.3
Dimethylsulfone
PAP
1.8.2.4
Glycine, serine and threonine metabolism
Oxidation state
+6
+4
+2
-2
Assimilatory sulfate reduction
Sulfate
PAPSS
Sat
CysND
APS
PAPSS
CysC
PAPS
CysH
Sulfite
CysJI
Sir
Sulfide
Dissimilatory sulfate reduction and oxidation
Sulfate
Sat
APS
AprAB
Sulfite
DsrAB
Sulfide
SOX system
SoxY
SoxZ
Sulfate
SoxB
SoxYZ-S-S-SO3
SoxA
SoxX
Thiosulfate
SoxC
SoxD
SoxYZ-S-SH
SoxYZ-S-SO3
SoxB
SoxYZ-SH
00920 10/7/15
(c) Kanehisa Laboratories

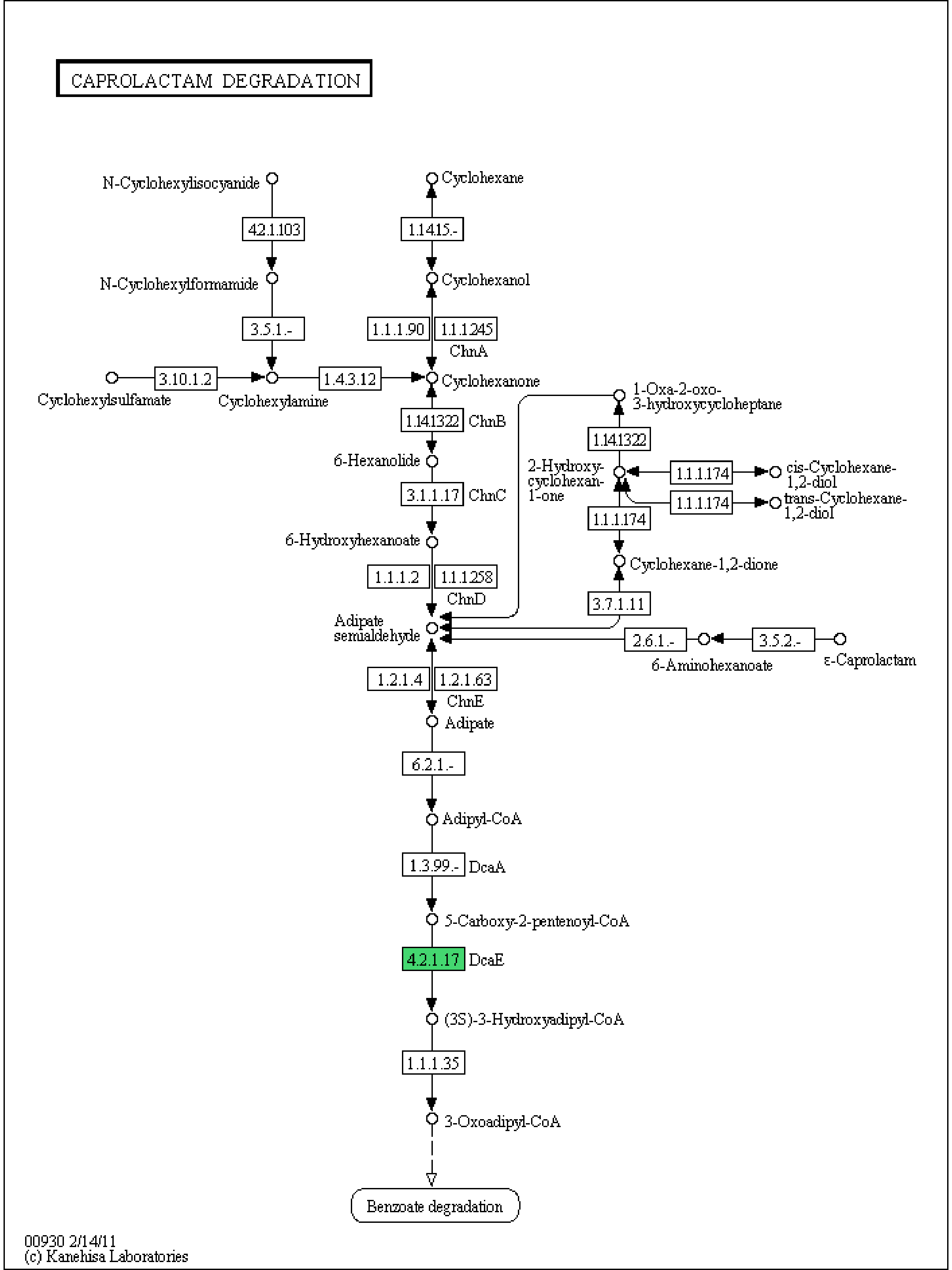
CAPROLACTAM DEGRADATION
N-Cyclohexylisocyanide
4.2.1.103
N-Cyclohexylformamide
3.5.1.-
Cyclohexane
1.14.15.-
Cyclohexanol
1.1.1.90
1.1.1.245
ChnA
Cyclohexylsulfamate
3.10.1.2
Cyclohexylamine
1.4.3.12
Cyclohexanone
1.14.13.22
ChnB
6-Hexanolide
3.1.1.17
ChnC
6-Hydroxyhexanoate
1.1.1.2
1.1.1.258
ChnD
Adipate semialdehyde
1-Oxa-2-oxo-3-hydroxycycloheptane
1.14.13.22
2-Hydroxy-cyclohexan-1-one
1.1.1.174
cis-Cyclohexane-1,2-diol
1.1.1.174
trans-Cyclohexane-1,2-diol
1.1.1.174
Cyclohexane-1,2-dione
3.7.1.11
2.6.1.-
6-Aminohexanoate
3.5.2.-
ε-Caprolactam
1.2.1.4
1.2.1.63
ChnE
Adipate
6.2.1.-
Adipyl-CoA
1.3.99.-
DcaA
5-Carboxy-2-pentenoyl-CoA
4.2.1.17
DcaE
(3S)-3-Hydroxyadipyl-CoA
1.1.1.35
3-Oxoadipyl-CoA
Benzoate degradation
00930 2/14/11
(c) Kanehisa Laboratories

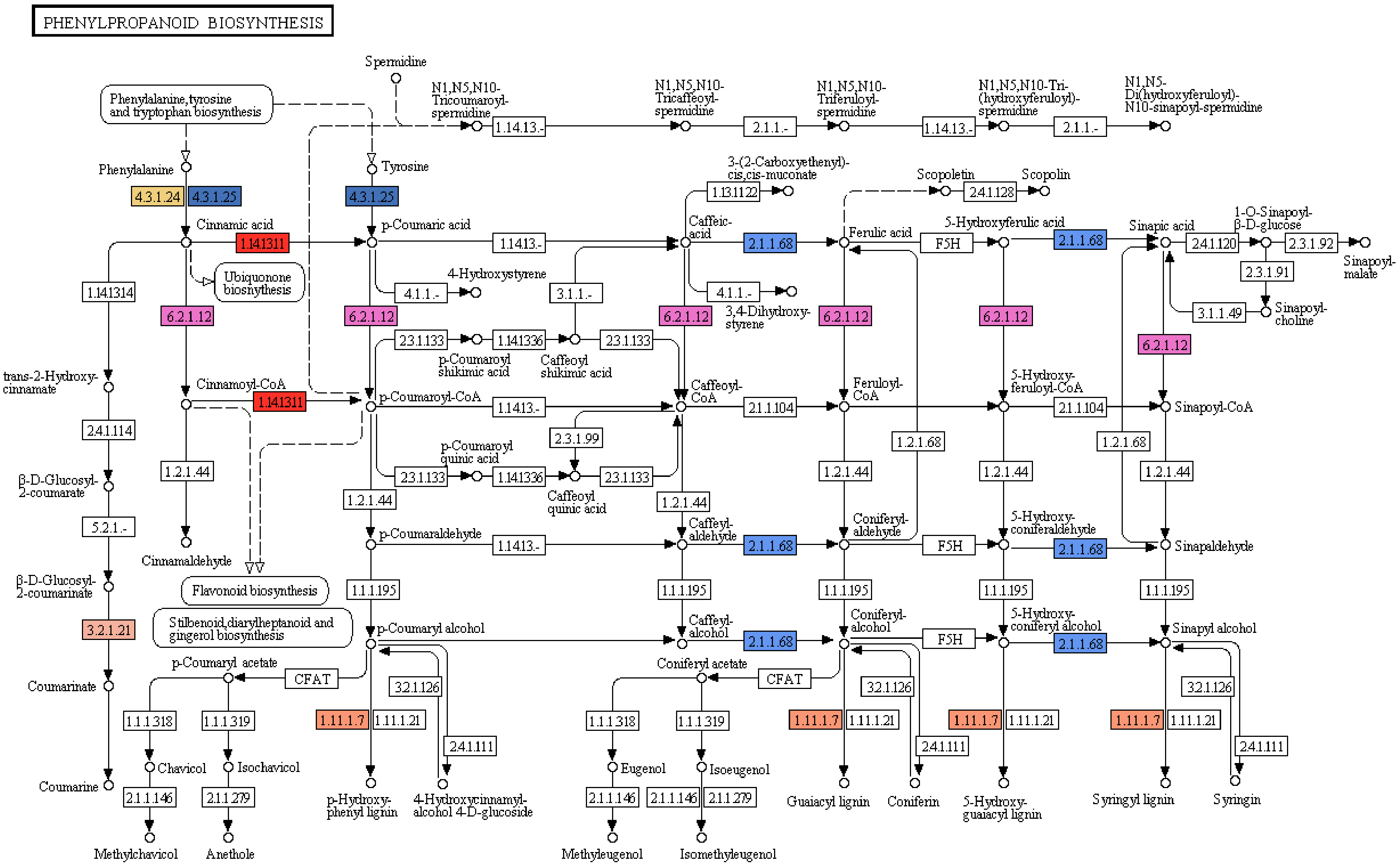

00940 8/22/14

(c) Kanehisa Laboratories

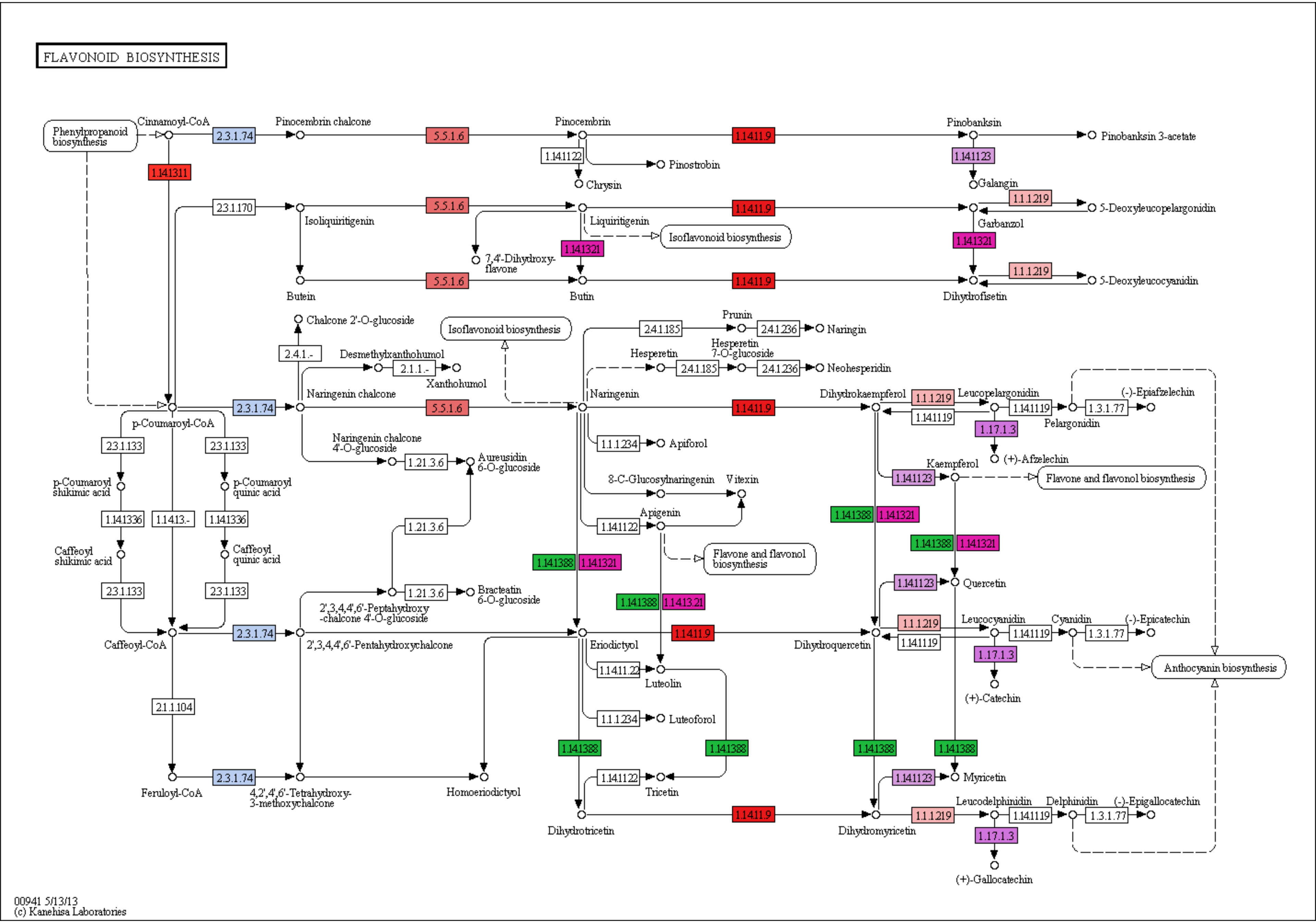
FLAVONOID BIOSYNTHESIS
Phenylpropanoid biosynthesis
Cinnamoyl-CoA
2.3.1.74
Pinocembrin chalcone
5.5.1.6
Pinocembrin
1.14.11.9
Pinobanksin
Pinobanksin 3-acetate
1.14.11.22
Pinostrobin
Chrysin
1.14.11.23
Galangin
1.14.13.11
2.3.1.170
Isoliquiritigenin
Liquiritigenin
Isoflavonoid biosynthesis
1.1.1.219
5-Deoxyleucopelargonidin
Garbanzol
1.14.13.21
7,4'-Dihydroxy-flavone
5-Deoxyleucocyanidin
Butein
Butin
Dihydrofisetin
Chalcone 2'-O-glucoside
2.4.1.-
Desmethylxanthohumol
2.1.1.-
Xanthohumol
2.4.1.185
Prunin
2.4.1.236
Naringin
Hesperetin
Hesperetin 7-O-glucoside
Neohesperidin
Naringenin chalcone
Naringenin
Dihydrokaempferol
Leucopelargonidin
1.14.11.19
1.3.1.77
(-)-Epiafzelechin
Pelargonidin
1.17.1.3
(+)-Afzelechin
p-Coumaroyl-CoA
2.3.1.133
Naringenin chalcone 4'-O-glucoside
1.21.3.6
Aureusidin 6-O-glucoside
1.1.1.234
Apiforol
Kaempferol
Flavone and flavonol biosynthesis
p-Coumaroyl shikimic acid
p-Coumaroyl quinic acid
8-C-Glucosylnaringenin
Vitexin
1.14.13.36
1.14.13.-
1.14.13.88
Apigenin
Caffeoyl shikimic acid
Caffeoyl quinic acid
Quercetin
Bracteatin 6-O-glucoside
2',3,4,4',6'-Peptahydroxy-chalcone 4'-O-glucoside
Caffeoyl-CoA
2',3,4,4',6'-Pentahydroxychalcone
Eriodictyol
Dihydroquercetin
Leucocyanidin
Cyanidin
(-)-Epicatechin
Luteolin
Anthocyanin biosynthesis
(+)-Catechin
Luteoforol
2.1.1.104
Myricetin
Feruloyl-CoA
4,2',4',6'-Tetrahydroxy-3-methoxychalcone
Homoeriodictyol
Tricetin
Dihydrotricetin
Dihydromyricetin
Leucodelphinidin
Delphinidin
(-)-Epigallocatechin
(+)-Gallocatechin
00941 5/13/13
(c) Kanehisa Laboratories

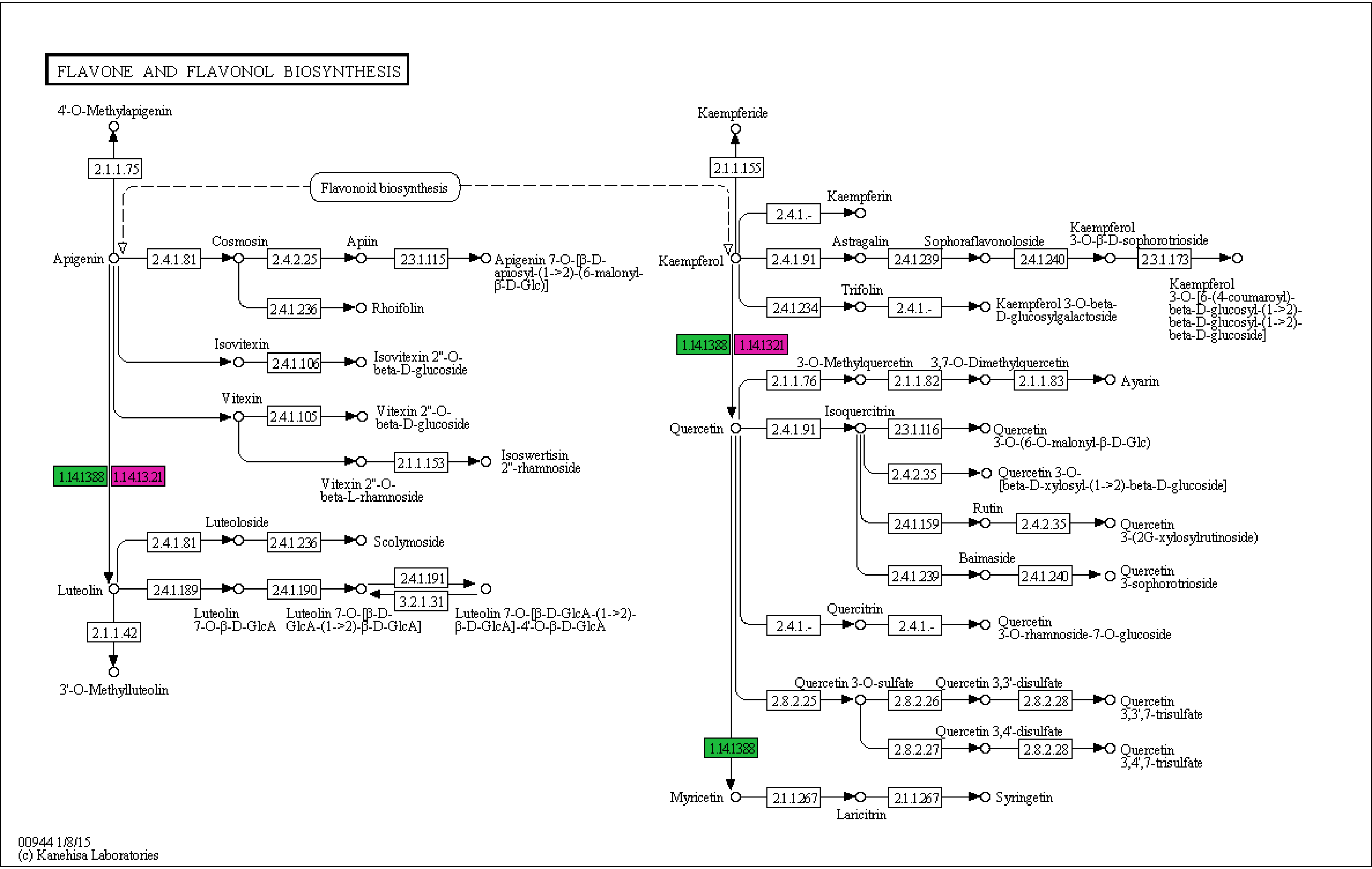
FLAVONE AND FLAVONOL BIOSYNTHESIS
4'-O-Methylapigenin
2.1.1.75
Flavonoid biosynthesis
Kaempferide
2.1.1.155
Apigenin
2.4.1.81
Cosmosin
2.4.2.25
Apiin
2.3.1.115
Apigenin 7-O-[β-D-apiosyl-(1->2)-(6-malonyl-β-D-Glc)]
2.4.1.236
Rhoifolin
Isovitexin
2.4.1.106
Isovitexin 2''-O-beta-D-glucoside
Vitexin
2.4.1.105
Vitexin 2''-O-beta-D-glucoside
2.1.1.153
Isoswertisin 2''-rhamnoside
Vitexin 2''-O-beta-L-rhamnoside
1.14.13.88
1.14.13.21
Luteoloside
2.4.1.81
2.4.1.236
Scolymoside
Luteolin
2.4.1.189
2.4.1.190
2.4.1.191
3.2.1.31
Luteolin 7-O-β-D-GlcA
Luteolin 7-O-[β-D-GlcA-(1->2)-β-D-GlcA]
Luteolin 7-O-[β-D-GlcA-(1->2)-β-D-GlcA]-4'-O-β-D-GlcA
2.1.1.42
3'-O-Methylluteolin
Kaempferin
2.4.1.-
Kaempferol
2.4.1.91
Astragalin
2.4.1.239
Sophoraflavonoloside
2.4.1.240
Kaempferol 3-O-β-D-sophorotrioside
2.3.1.173
Kaempferol 3-O-[6-(4-coumaroyl)-beta-D-glucosyl-(1->2)-beta-D-glucosyl-(1->2)-beta-D-glucoside]
Trifolin
2.4.1.234
2.4.1.-
Kaempferol 3-O-beta-D-glucosylgalactoside
1.14.13.88
1.14.13.21
3-O-Methylquercetin
3,7-O-Dimethylquercetin
2.1.1.76
2.1.1.82
2.1.1.83
Ayarin
Quercetin
2.4.1.91
Isoquercitrin
2.3.1.116
Quercetin 3-O-(6-O-malonyl-β-D-Glc)
2.4.2.35
Quercetin 3-O-[beta-D-xylosyl-(1->2)-beta-D-glucoside]
Rutin
2.4.1.159
2.4.2.35
Quercetin 3-(2G-xylosylrutinoside)
Baimaside
2.4.1.239
2.4.1.240
Quercetin 3-sophorotrioside
Quercitrin
2.4.1.-
2.4.1.-
Quercetin 3-O-rhamnoside-7-O-glucoside
Quercetin 3-O-sulfate
Quercetin 3,3'-disulfate
2.8.2.25
2.8.2.26
2.8.2.28
Quercetin 3,3',7-trisulfate
Quercetin 3,4'-disulfate
2.8.2.27
2.8.2.28
Quercetin 3,4',7-trisulfate
1.14.13.88
Myricetin
2.1.1.267
2.1.1.267
Syringetin
Laricitrin
00944 1/8/15
(c) Kanehisa Laboratories

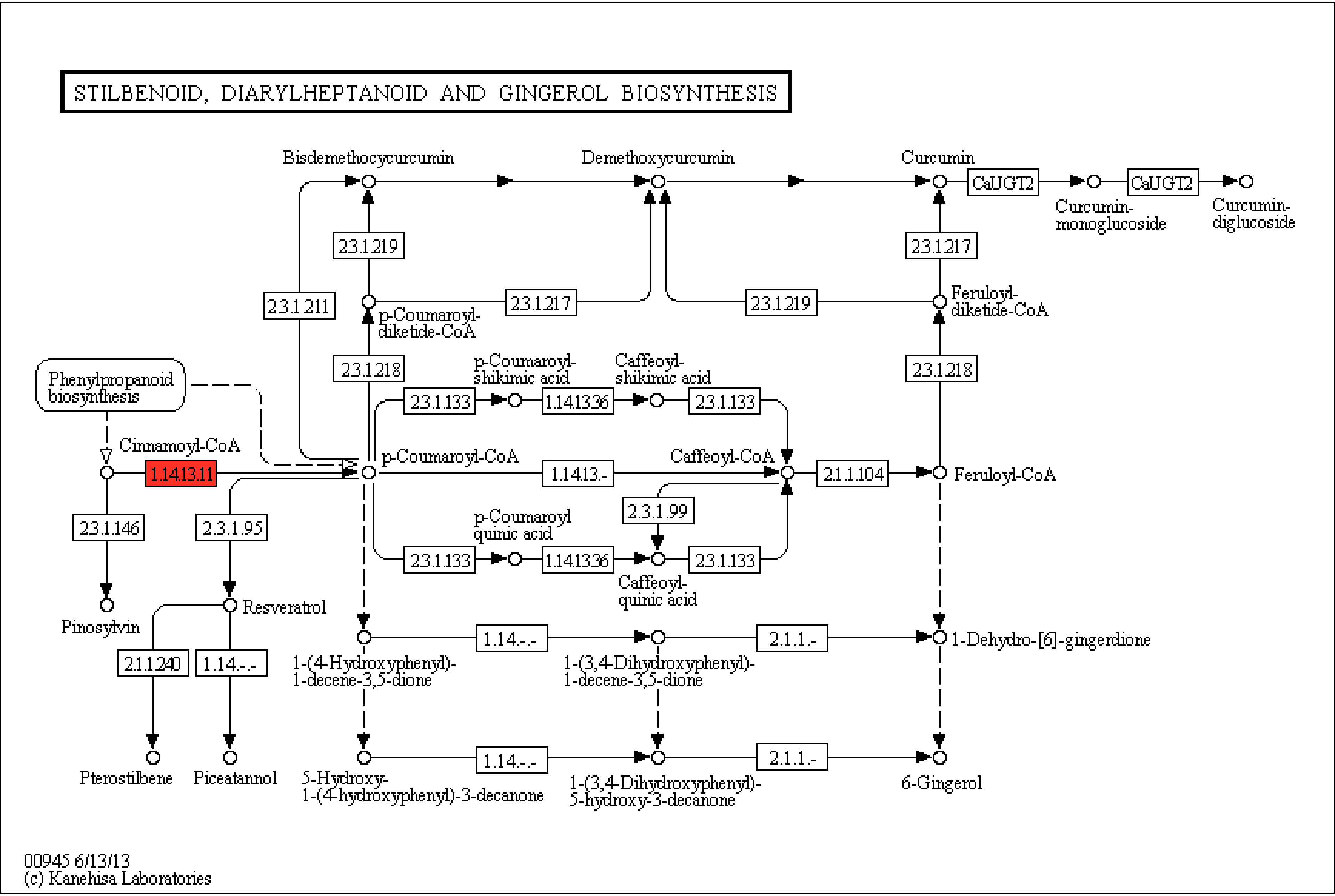

STILBENOID, DIARYLHEPTANOID AND GINGEROL BIOSYNTHESIS
Bisdemethoxycurcumin
Demethoxycurcumin
Curcumin
CaUGT2
CaUGT2
Curcumin-monoglucoside
Curcumin-diglucoside
2.3.1.219
2.3.1.217
2.3.1.211
2.3.1.217
2.3.1.219
p-Coumaroyl-diketide-CoA
Feruloyl-diketide-CoA
2.3.1.218
2.3.1.218
Phenylpropanoid biosynthesis
p-Coumaroyl-shikimic acid
Caffeoyl-shikimic acid
2.3.1.133
1.14.13.36
2.3.1.133
Cinnamoyl-CoA
p-Coumaroyl-CoA
Caffeoyl-CoA
1.14.13.11
1.14.13.-
2.1.1.104
Feruloyl-CoA
2.3.1.146
2.3.1.95
2.3.1.99
p-Coumaroyl quinic acid
2.3.1.133
1.14.13.36
2.3.1.133
Caffeoyl-quinic acid
Pinosylvin
Resveratrol
1.14.-.-
2.1.1.-
1-Dehydro-[6]-gingerdione
2.1.1.240
1.14.-.-
1-(4-Hydroxyphenyl)-1-decene-3,5-dione
1-(3,4-Dihydroxyphenyl)-1-decene-3,5-dione
1.14.-.-
2.1.1.-
Pterostilbene
Piceatannol
5-Hydroxy-1-(4-hydroxyphenyl)-3-decanone
1-(3,4-Dihydroxyphenyl)-5-hydroxy-3-decanone
6-Gingerol
00945 6/13/13
(c) Kanehisa Laboratories

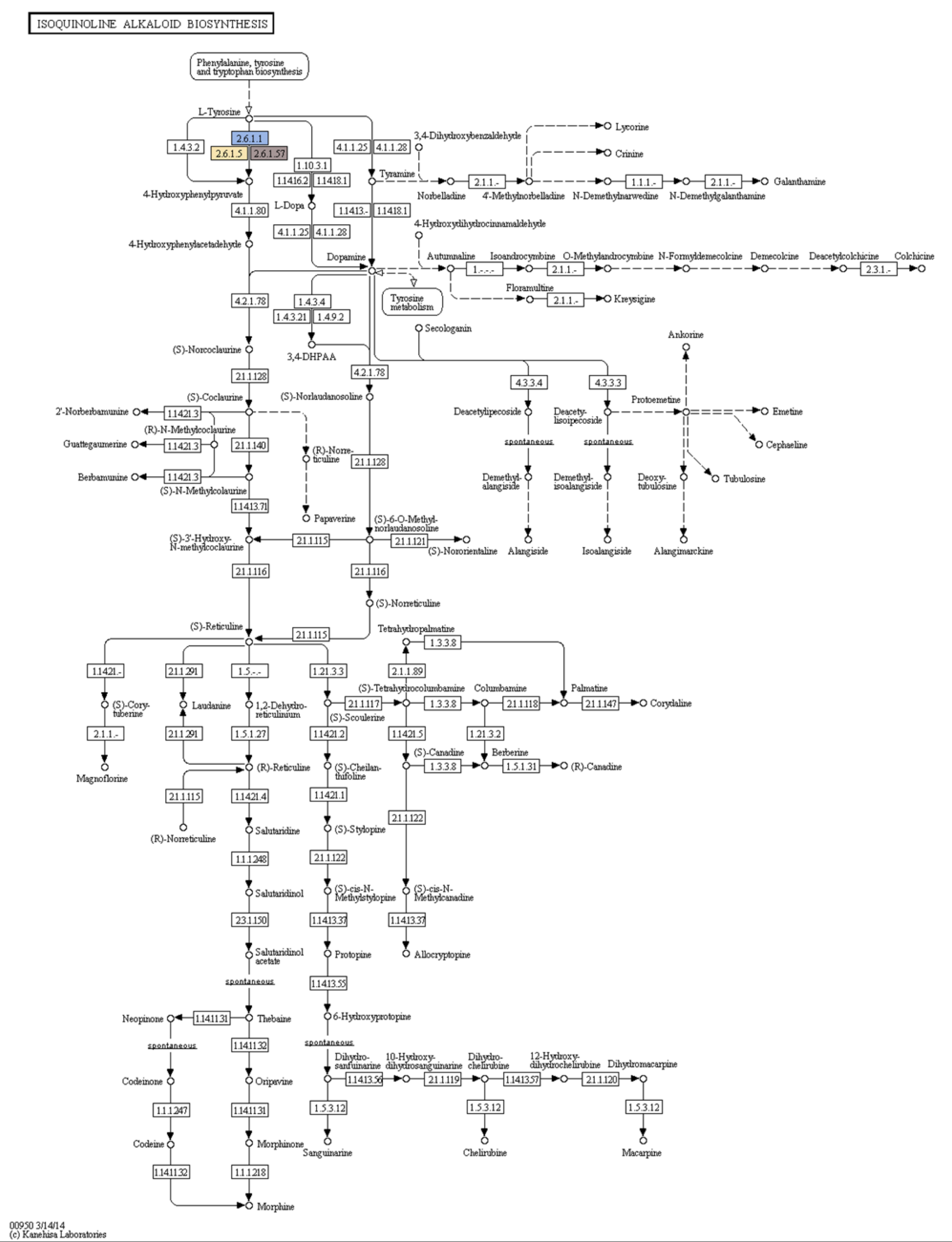
ISOQUINOLINE ALKALOID BIOSYNTHESIS
Phenylalanine, tyrosine and tryptophan biosynthesis
L-Tyrosine
1.4.3.2
2.6.1.1
2.6.1.5
2.6.1.57
1.10.3.1
1.14.16.2
1.14.18.1
4.1.1.25
4.1.1.28
Tyramine
4-Hydroxyphenylpyruvate
4.1.1.80
L-Dopa
1.14.13.-
1.14.18.1
4.1.1.25
4.1.1.28
4-Hydroxyphenylacetaldehyde
Dopamine
Tyrosine metabolism
1.4.3.4
1.4.3.21
1.4.9.2
4.2.1.78
3,4-DHPAA
(S)-Norcoclaurine
2.1.1.128
(S)-Coclaurine
2'-Norberbamunine
1.14.21.3
(R)-N-Methylcoclaurine
Guattegaumerine
1.14.21.3
2.1.1.140
Berbamunine
1.14.21.3
(S)-N-Methylcoclaurine
1.14.13.71
(R)-Norreticuline
Papaverine
4.2.1.78
(S)-Norlaudanosoline
2.1.1.128
(S)-6-O-Methylnorlaudanosoline
2.1.1.121
(S)-Nororientaline
(S)-3'-Hydroxy-N-methylcoclaurine
2.1.1.115
2.1.1.116
2.1.1.116
(S)-Norreticuline
(S)-Reticuline
2.1.1.115
3,4-Dihydroxybenzaldehyde
Norbelladine
2.1.1.-
4'-Methylnorbelladine
Lycorine
Crinine
N-Demethylnarwedine
1.1.1.-
N-Demethylgalanthamine
2.1.1.-
Galanthamine
4-Hydroxydihydrocinnamaldehyde
Autumnaline
1.-.-.-
Isoandrocymbine
2.1.1.-
O-Methylandrocymbine
N-Formyldemecolcine
Demecolcine
Deacetylcolchicine
2.3.1.-
Colchicine
Floramultine
2.1.1.-
Kreysigine
Secologanin
4.3.3.4
4.3.3.3
Ankorine
Deacetylipecoside
Deacetylisoipecoside
Protoemetine
Emetine
Cephaeline
spontaneous
spontaneous
Demethylalangiside
Demethylisoalangiside
Deoxytubulosine
Tubulosine
Alangiside
Isoalangiside
Alangimarckine
1.14.21.-
(S)-Corytuberine
2.1.1.-
Magnoflorine
2.1.1.291
Laudanine
2.1.1.291
1.5.-.-
1,2-Dehydroreticulinium
1.5.1.27
(R)-Reticuline
2.1.1.115
(R)-Norreticuline
1.14.21.4
Salutaridine
1.1.1.248
Salutaridinol
2.3.1.150
Salutaridinol acetate
spontaneous
Thebaine
1.14.11.31
Neopinone
spontaneous
Codeinone
1.1.1.247
Codeine
1.14.11.32
1.14.11.32
Oripavine
1.14.11.31
Morphinone
1.1.1.218
Morphine
1.21.3.3
2.1.1.117
(S)-Scoulerine
Tetrahydropalmatine
1.3.3.8
2.1.1.89
(S)-Tetrahydrocolumbamine
1.3.3.8
Columbamine
2.1.1.118
Palmatine
2.1.1.147
Corydaline
1.14.21.2
(S)-Cheilanthifoline
1.14.21.5
(S)-Canadine
1.3.3.8
1.21.3.2
Berberine
1.5.1.31
(R)-Canadine
1.14.21.1
(S)-Stylopine
2.1.1.122
2.1.1.122
(S)-cis-N-Methylstylopine
(S)-cis-N-Methylcanadine
1.14.13.37
1.14.13.37
Protopine
Allocryptopine
1.14.13.55
6-Hydroxyprotopine
spontaneous
Dihydrosanguinarine
1.14.13.56
10-Hydroxydihydrosanguinarine
2.1.1.119
Dihydrochelirubine
1.14.13.57
12-Hydroxydihydrochelirubine
2.1.1.120
Dihydromacarpine
1.5.3.12
Sanguinarine
1.5.3.12
Chelirubine
1.5.3.12
Macarpine
00950 3/14/14
(c) Kanehisa Laboratories

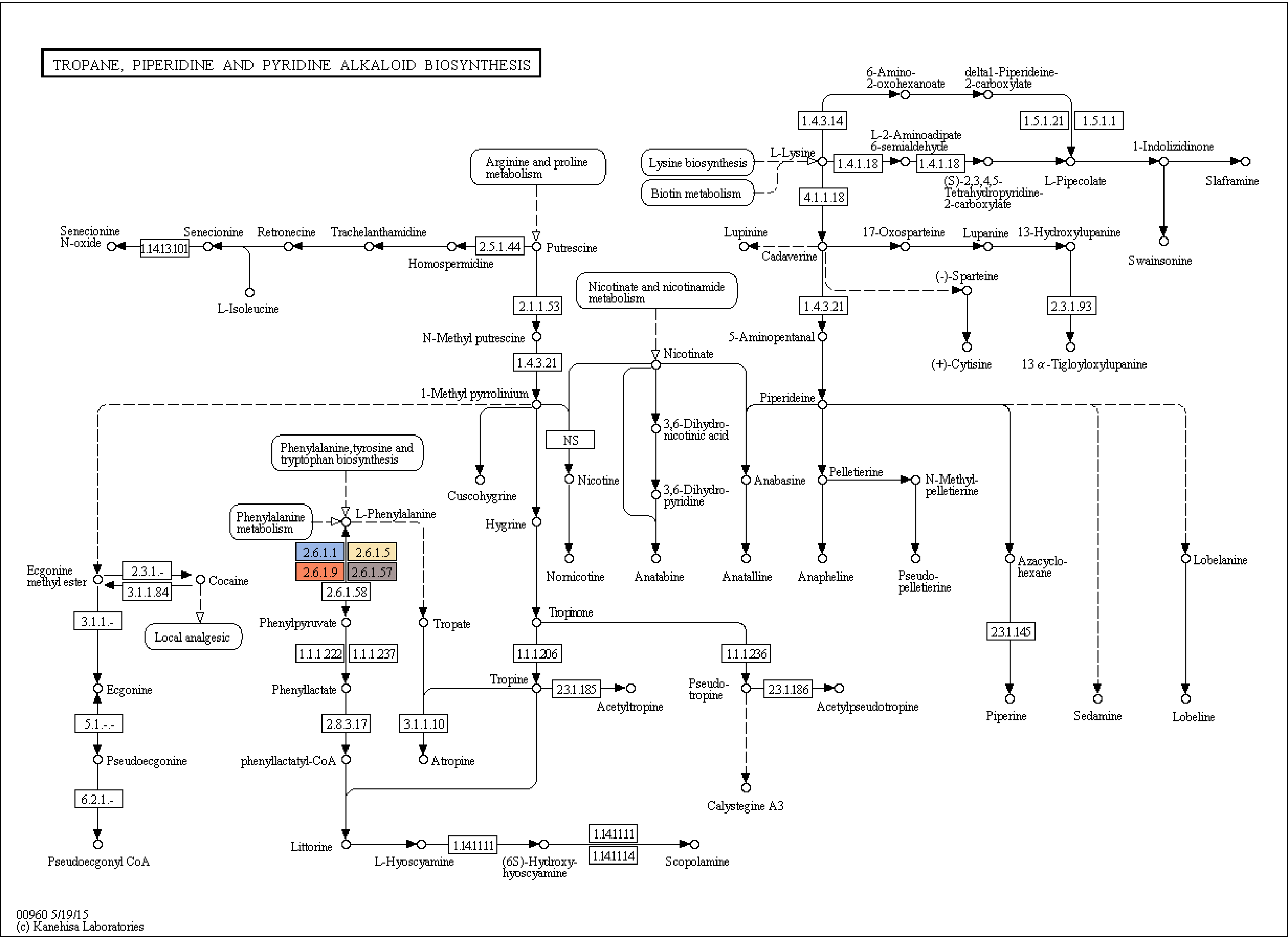
TROPANE, PIPERIDINE AND PYRIDINE ALKALOID BIOSYNTHESIS
6-Amino-
2-oxohexanoate
delta1-Piperideine-
2-carboxylate
1.4.3.14
1.5.1.21
1.5.1.1
Arginine and proline metabolism
Lysine biosynthesis
Biotin metabolism
L-Lysine
1.4.1.18
L-2-Aminoadipate
6-semialdehyde
1.4.1.18
(S)-2,3,4,5-
Tetrahydropyridine-
2-carboxylate
L-Pipecolate
1-Indolizidinone
Slaframine
Swainsonine
4.1.1.18
Senecionine
N-oxide
1.14.13.101
Senecionine
Retronecine
Trachelanthamidine
2.5.1.44
Putrescine
Homospermidine
L-Isoleucine
Lupinine
Cadaverine
17-Oxosparteine
Lupanine
13-Hydroxylupanine
(-)-Sparteine
Nicotinate and nicotinamide metabolism
2.1.1.53
1.4.3.21
2.3.1.93
N-Methyl putrescine
5-Aminopentanal
(+)-Cytisine
13 α-Tigloyloxylupanine
1.4.3.21
Nicotinate
1-Methyl pyrrolinium
Piperideine
NS
3,6-Dihydro-
nicotinic acid
Phenylalanine,tyrosine and tryptophan biosynthesis
Cuscohygrine
Nicotine
3,6-Dihydro-
pyridine
Anabasine
Pelletierine
N-Methyl-
pelletierine
Phenylalanine metabolism
L-Phenylalanine
Hygrine
2.6.1.1
2.6.1.5
2.6.1.9
2.6.1.57
2.6.1.58
Ecgonine
methyl ester
2.3.1.-
3.1.1.84
Cocaine
Nornicotine
Anatabine
Anatalline
Anapheline
Pseudo-
pelletierine
Azacyclo-
hexane
Lobelanine
3.1.1.-
Local analgesic
Phenylpyruvate
Tropate
Tropinone
1.1.1.222
1.1.1.237
1.1.1.206
1.1.1.236
2.3.1.145
Ecgonine
Phenyllactate
Tropine
2.3.1.185
Acetyltropine
Pseudo-
tropine
2.3.1.186
Acetylpseudotropine
Piperine
Sedamine
Lobeline
5.1.-.-
2.8.3.17
3.1.1.10
Pseudoecgonine
phenyllactatyl-CoA
Atropine
6.2.1.-
Calystegine A3
1.14.11.14
1.14.11.11
Littorine
L-Hyoscyamine
1.14.11.11
(6S)-Hydroxy-
hyoscyamine
Scopolamine
Pseudoecgonyl CoA
00960 5/19/15
(c) Kanehisa Laboratories

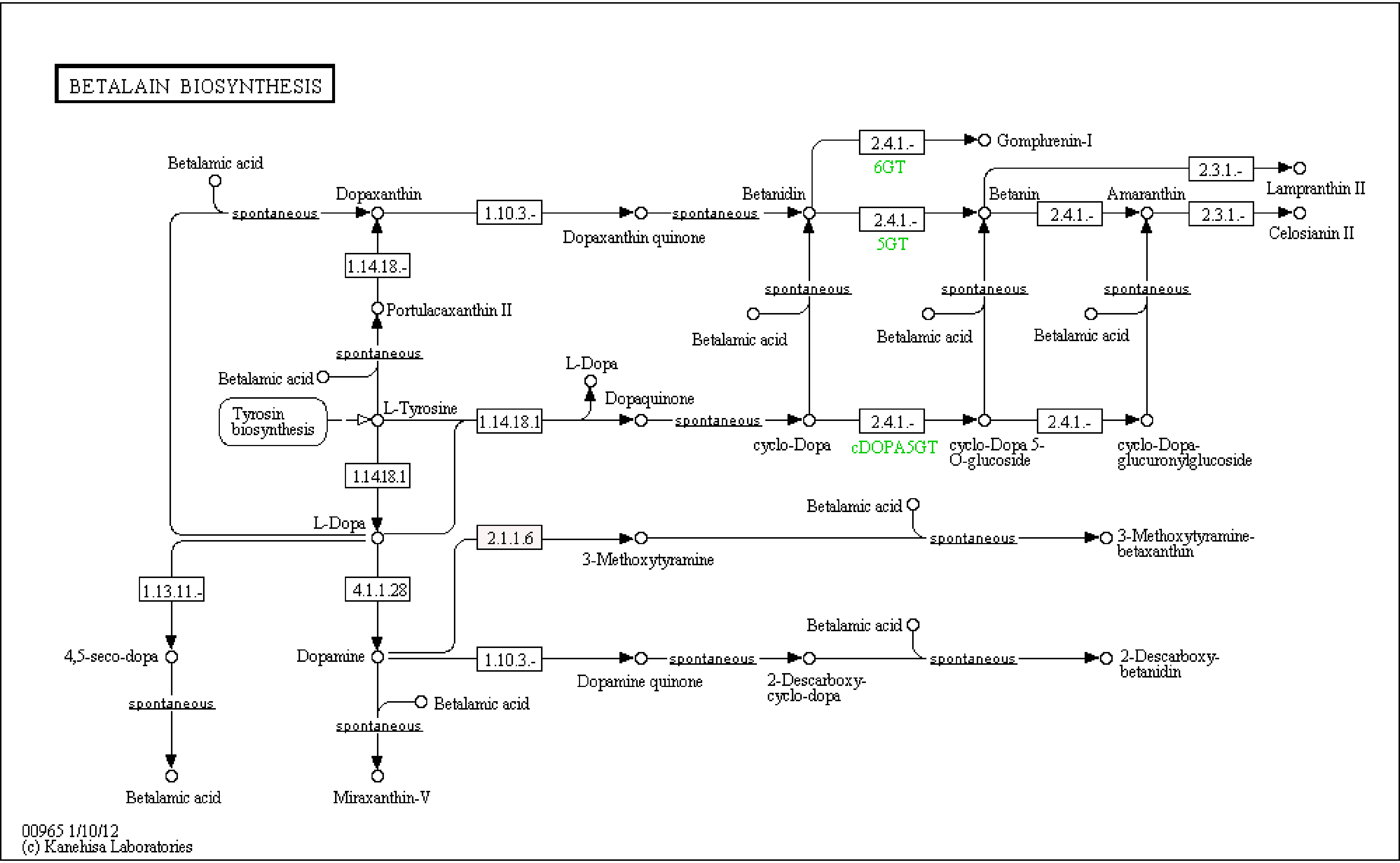
BETALAIN BIOSYNTHESIS
Betalamic acid
spontaneous
Dopaxanthin
1.10.3.-
Dopaxanthin quinone
Betanidin
2.4.1.-
6GT
Gomphrenin-I
5GT
Betanin
Amaranthin
2.3.1.-
Lamprianthin II
Celosianin II
1.14.18.-
Portulacaxanthin II
L-Tyrosine
Tyrosin biosynthesis
1.14.18.1
L-Dopa
Dopaquinone
cyclo-Dopa
cDOPA5GT
cyclo-Dopa 5-O-glucoside
cyclo-Dopa-glucuronylglucoside
2.1.1.6
3-Methoxytyramine
3-Methoxytyramine-betaxanthin
1.13.11.-
4.1.1.28
4,5-seco-dopa
Dopamine
Dopamine quinone
2-Descarboxy-cyclo-dopa
2-Descarboxy-betanidin
Miraxanthin-V
00965 1/10/12
(c) Kanehisa Laboratories

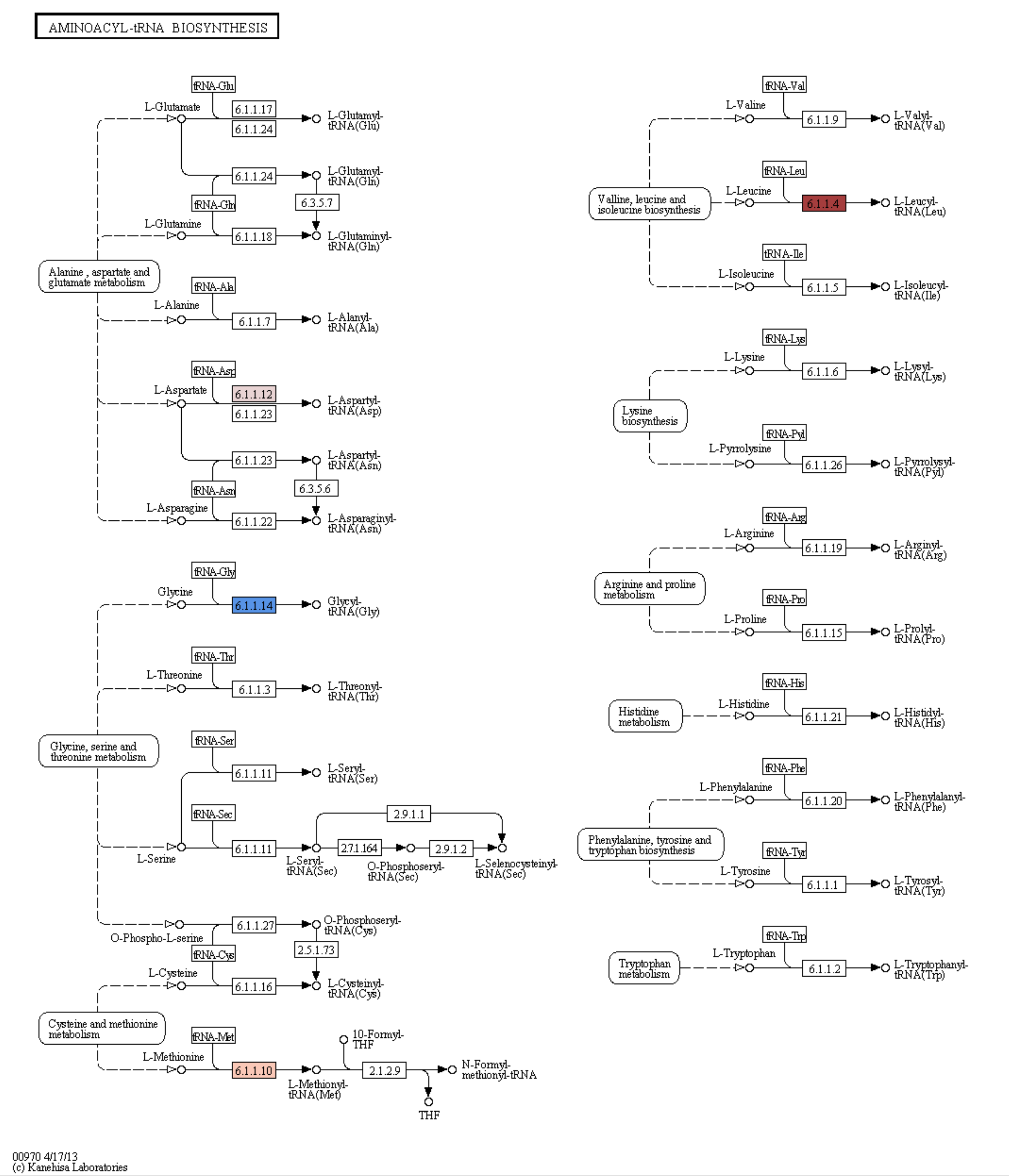
AMINOACYL-tRNA BIOSYNTHESIS
tRNA-Glu
L-Glutamate
6.1.1.17
6.1.1.24
L-Glutamyl-tRNA(Glu)
6.1.1.24
L-Glutamyl-tRNA(Gln)
6.3.5.7
tRNA-Gln
L-Glutamine
6.1.1.18
L-Glutaminyl-tRNA(Gln)
Alanine, aspartate and glutamate metabolism
tRNA-Ala
L-Alanine
6.1.1.7
L-Alanyl-tRNA(Ala)
tRNA-Asp
L-Aspartate
6.1.1.12
6.1.1.23
L-Aspartyl-tRNA(Asp)
6.1.1.23
L-Aspartyl-tRNA(Asn)
6.3.5.6
tRNA-Asn
L-Asparagine
6.1.1.22
L-Asparaginyl-tRNA(Asn)
tRNA-Gly
Glycine
6.1.1.14
Glycyl-tRNA(Gly)
tRNA-Thr
L-Threonine
6.1.1.3
L-Threonyl-tRNA(Thr)
Glycine, serine and threonine metabolism
tRNA-Ser
6.1.1.11
L-Seryl-tRNA(Ser)
tRNA-Sec
2.9.1.1
6.1.1.11
2.7.1.164
2.9.1.2
L-Serine
L-Seryl-tRNA(Sec)
O-Phosphoseryl-tRNA(Sec)
L-Selenocysteinyl-tRNA(Sec)
6.1.1.27
O-Phosphoseryl-tRNA(Cys)
O-Phospho-L-serine
2.5.1.73
tRNA-Cys
L-Cysteine
6.1.1.16
L-Cysteinyl-tRNA(Cys)
Cysteine and methionine metabolism
tRNA-Met
10-Formyl-THF
L-Methionine
6.1.1.10
2.1.2.9
N-Formyl-methionyl-tRNA
L-Methionyl-tRNA(Met)
THF
tRNA-Val
L-Valine
6.1.1.9
L-Valyl-tRNA(Val)
tRNA-Leu
L-Leucine
Valine, leucine and isoleucine biosynthesis
6.1.1.4
L-Leucyl-tRNA(Leu)
tRNA-Ile
L-Isoleucine
6.1.1.5
L-Isoleucyl-tRNA(Ile)
tRNA-Lys
L-Lysine
6.1.1.6
L-Lysyl-tRNA(Lys)
Lysine biosynthesis
tRNA-Pyl
L-Pyrrolysine
6.1.1.26
L-Pyrrolysyl-tRNA(Pyl)
tRNA-Arg
L-Arginine
6.1.1.19
L-Arginyl-tRNA(Arg)
Arginine and proline metabolism
tRNA-Pro
L-Proline
6.1.1.15
L-Prolyl-tRNA(Pro)
tRNA-His
Histidine metabolism
L-Histidine
6.1.1.21
L-Histidyl-tRNA(His)
tRNA-Phe
L-Phenylalanine
6.1.1.20
L-Phenylalanyl-tRNA(Phe)
Phenylalanine, tyrosine and tryptophan biosynthesis
tRNA-Tyr
L-Tyrosine
6.1.1.1
L-Tyrosyl-tRNA(Tyr)
tRNA-Trp
Tryptophan metabolism
L-Tryptophan
6.1.1.2
L-Tryptophanyl-tRNA(Trp)
00970 4/17/13
(c) Kanehisa Laboratories

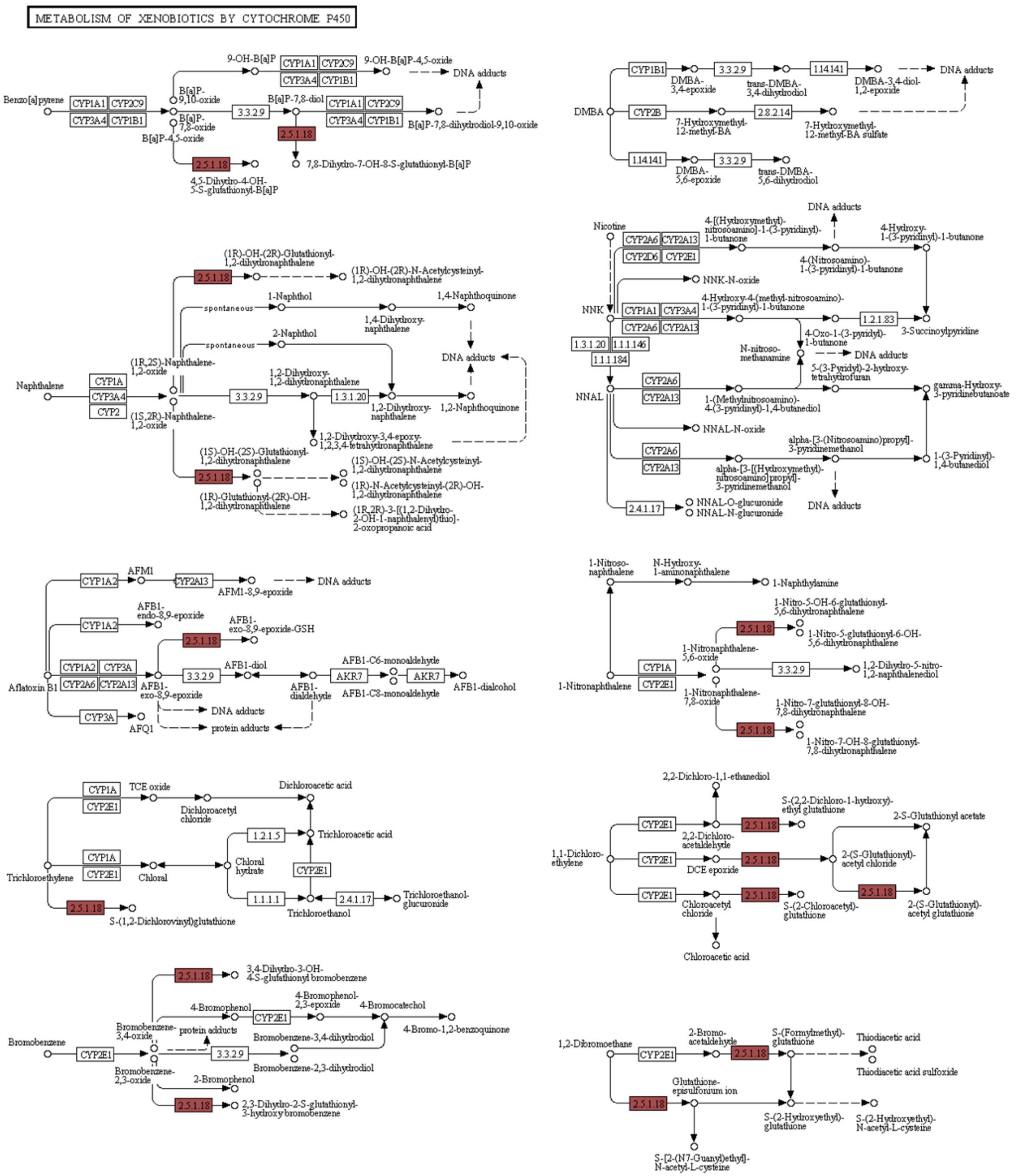

00980 11/18/15
(c) Kanehisa Laboratories

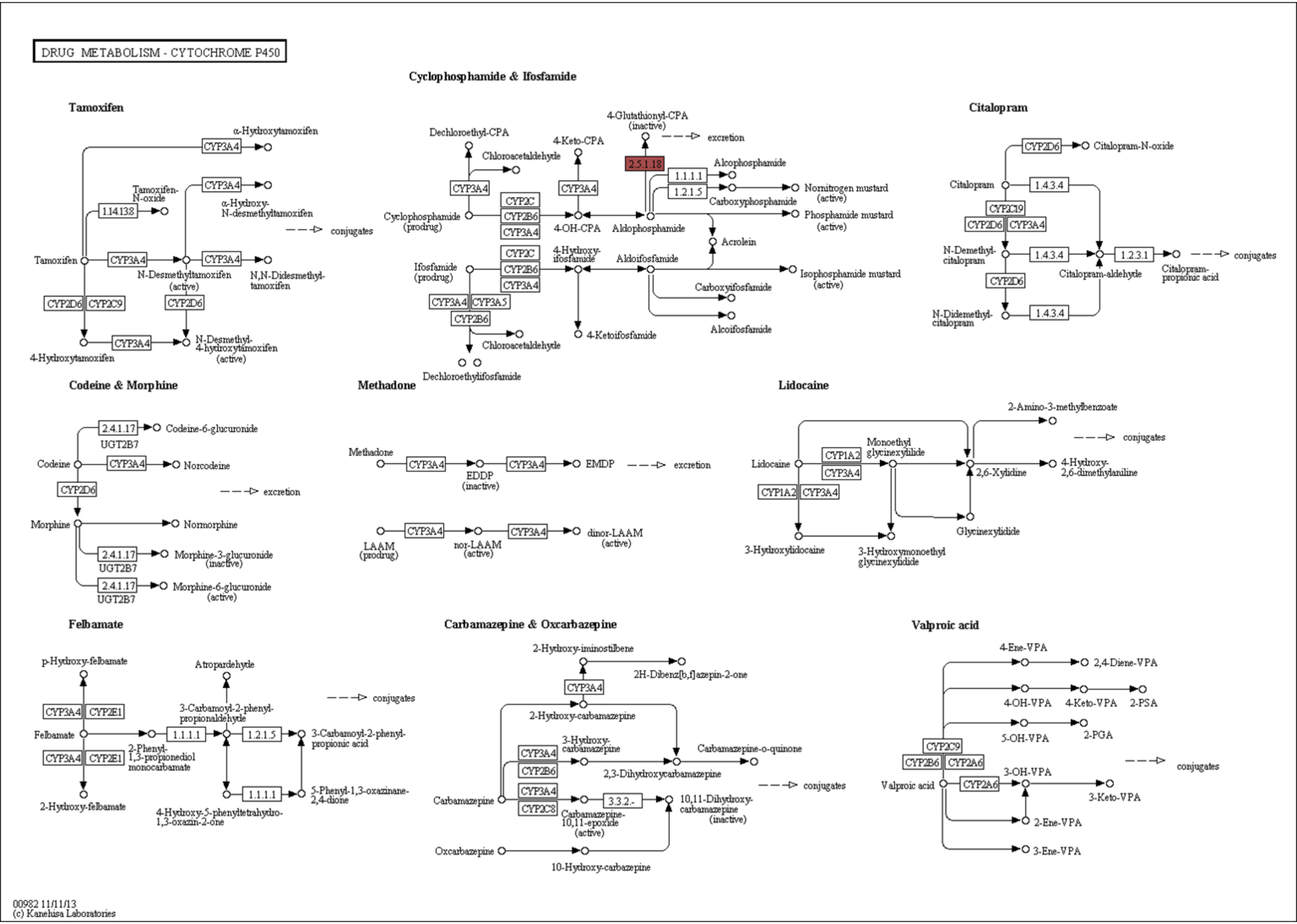
DRUG METABOLISM - CYTOCHROME P450
Cyclophosphamide & Ifosfamide
Tamoxifen
α-Hydroxytamoxifen
CYP3A4
Tamoxifen-N-oxide
1.14.13.8
α-Hydroxy-N-desmethyltamoxifen
conjugates
N-Desmethyltamoxifen (active)
N,N-Didesmethyl-tamoxifen
CYP2D6
CYP2C9
4-Hydroxytamoxifen
N-Desmethyl-4-hydroxytamoxifen (active)
Dechloroethyl-CPA
Chloroacetaldehyde
4-Keto-CPA
4-Glutathionyl-CPA (inactive)
excretion
2.5.1.18
1.1.1.1
1.2.1.5
Alcophosphamide
Nornitrogen mustard (active)
Carboxyphosphamide
Cyclophosphamide (prodrug)
CYP2C
CYP2B6
4-OH-CPA
Aldophosphamide
Phosphamide mustard (active)
Acrolein
Ifosfamide (prodrug)
4-Hydroxy-ifosfamide
Aldoifosfamide
Isophosphamide mustard (active)
Carboxyifosfamide
CYP3A5
Alcoifosfamide
4-Ketoifosfamide
Dechloroethylifosfamide
Citalopram
Citalopram-N-oxide
1.4.3.4
CYP2C19
N-Demethyl-citalopram
1.2.3.1
Citalopram-aldehyde
Citalopram-propionic acid
N-Didemethyl-citalopram
Codeine & Morphine
2.4.1.17
UGT2B7
Codeine-6-glucuronide
Codeine
Norcodeine
Morphine
Normorphine
Morphine-3-glucuronide (inactive)
Morphine-6-glucuronide (active)
Methadone
EDDP (inactive)
EMDP
LAAM (prodrug)
nor-LAAM (active)
dinor-LAAM (active)
Lidocaine
2-Amino-3-methylbenzoate
Monoethyl glycinexylidide
CYP1A2
2,6-Xylidine
4-Hydroxy-2,6-dimethylaniline
Glycinexylidide
3-Hydroxylidocaine
3-Hydroxymonoethyl glycinexylidide
Felbamate
p-Hydroxy-felbamate
Atropaldehyde
CYP2E1
3-Carbamoyl-2-phenyl-propionaldehyde
3-Carbamoyl-2-phenyl-propionic acid
2-Phenyl-1,3-propanediol monocarbamate
5-Phenyl-1,3-oxazinane-2,4-dione
2-Hydroxy-felbamate
4-Hydroxy-5-phenyltetrahydro-1,3-oxazin-2-one
Carbamazepine & Oxcarbazepine
2-Hydroxy-iminostilbene
2H-Dibenz[b,f]azepin-2-one
2-Hydroxy-carbamazepine
3-Hydroxy-carbamazepine
Carbamazepine-o-quinone
2,3-Dihydroxycarbamazepine
Carbamazepine
CYP2C8
3.3.2.-
Carbamazepine-10,11-epoxide (active)
10,11-Dihydroxy-carbamazepine (inactive)
Oxcarbazepine
10-Hydroxy-carbazepine
Valproic acid
4-Ene-VPA
2,4-Diene-VPA
4-OH-VPA
4-Keto-VPA
2-PSA
5-OH-VPA
2-PGA
CYP2A6
3-OH-VPA
3-Keto-VPA
2-Ene-VPA
3-Ene-VPA
00982 11/11/13
(c) Kanehisa Laboratories

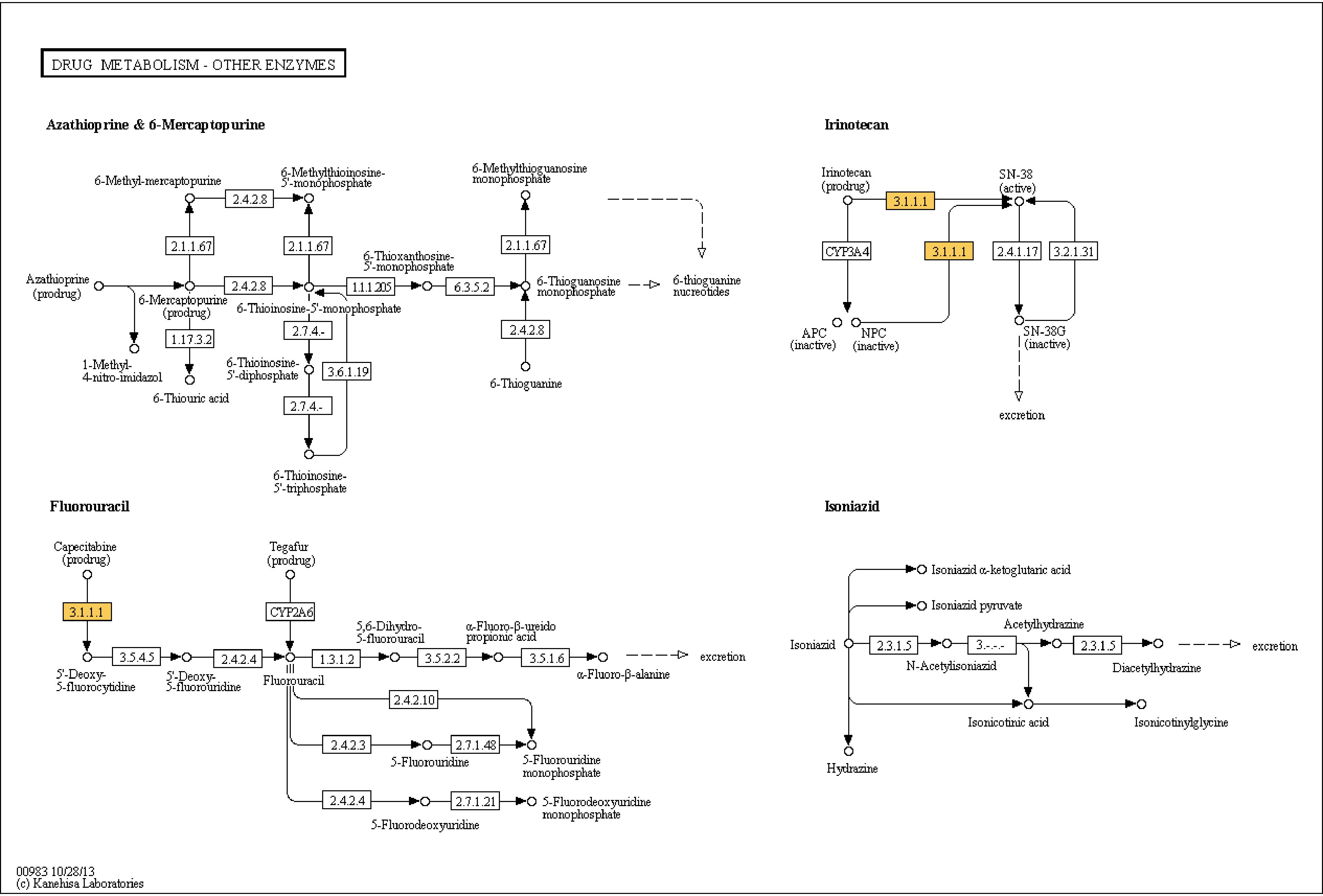

(c) Kanehisa Laboratories

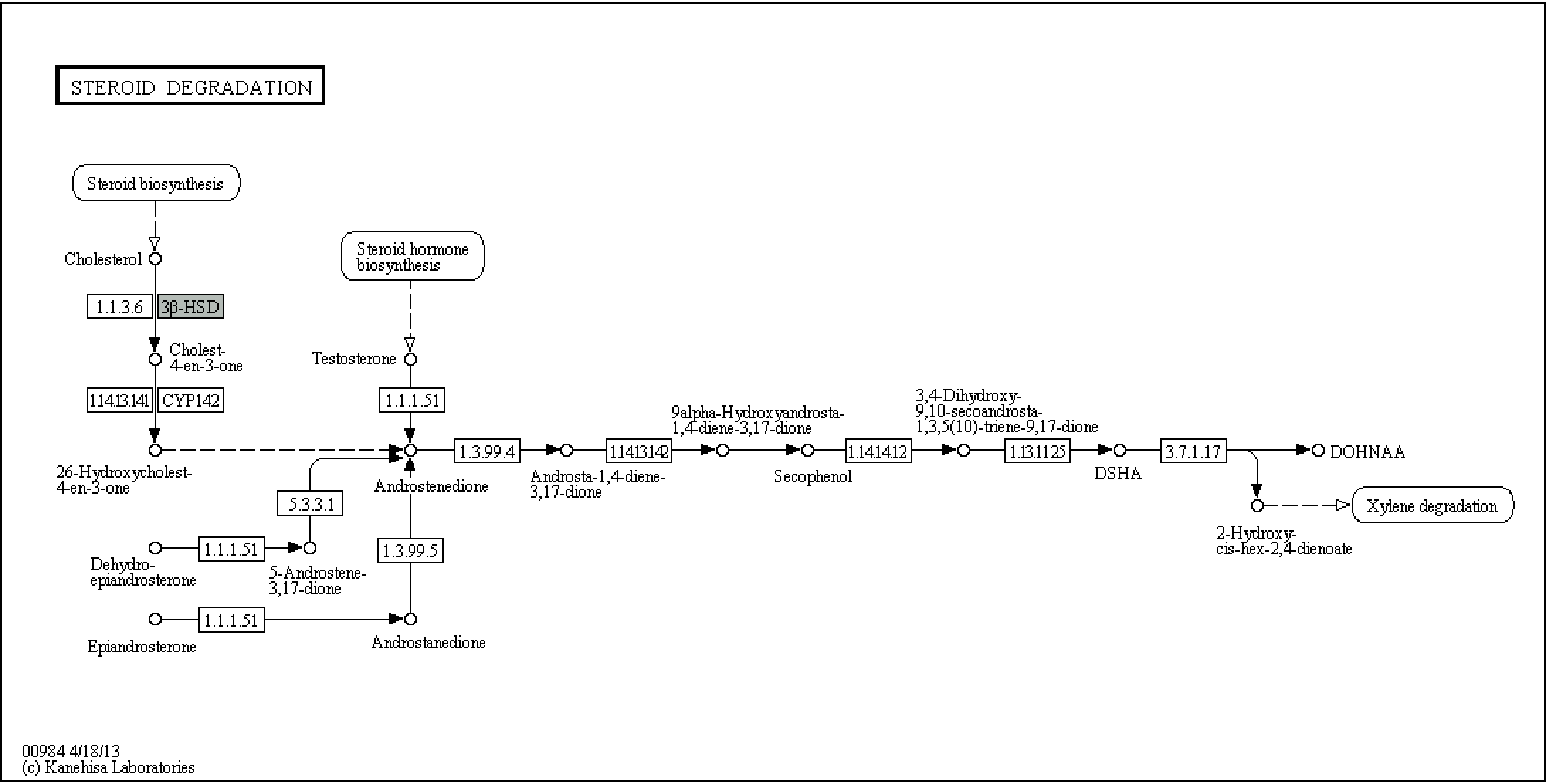

STEROID DEGRADATION
Steroid biosynthesis
Cholesterol
1.1.3.6
3β-HSD
Cholest-4-en-3-one
1.14.13.141
CYP142
26-Hydroxycholest-4-en-3-one
Steroid hormone biosynthesis
Testosterone
1.1.1.51
Androstenedione
5.3.3.1
Dehydro-epiandrosterone
1.1.1.51
5-Androstene-3,17-dione
1.3.99.5
Epiandrosterone
1.1.1.51
Androstanedione
1.3.99.4
Androsta-1,4-diene-3,17-dione
1.14.13.142
9alpha-Hydroxyandrosta-1,4-diene-3,17-dione
Secophenol
1.14.14.12
3,4-Dihydroxy-9,10-secoandrosta-1,3,5(10)-triene-9,17-dione
1.13.11.25
DSHA
3.7.1.17
DOHNAA
2-Hydroxy-cis-hex-2,4-dienoate
Xylene degradation
00984 4/18/13
(c) Kanehisa Laboratories

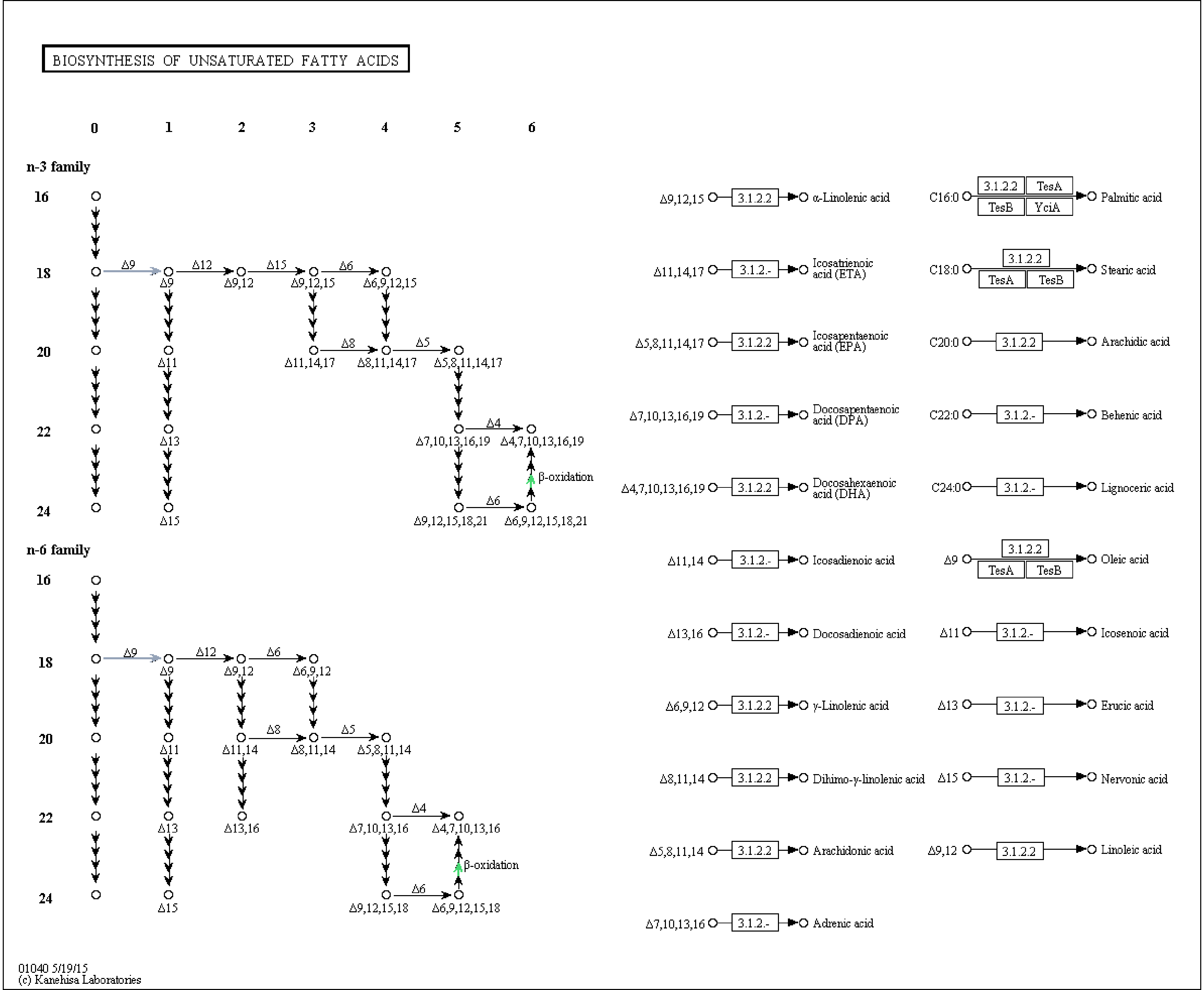
BIOSYNTHESIS OF UNSATURATED FATTY ACIDS
0 1 2 3 4 5 6
n-3 family
16
18
20
22
24
Δ9
Δ12
Δ15
Δ6
Δ9,12
Δ9,12,15
Δ6,9,12,15
Δ11
Δ8
Δ5
Δ11,14,17
Δ8,11,14,17
Δ5,8,11,14,17
Δ13
Δ4
Δ7,10,13,16,19
Δ4,7,10,13,16,19
β-oxidation
Δ15
Δ9,12,15,18,21
Δ6,9,12,15,18,21
n-6 family
Δ6,9,12
Δ11,14
Δ8,11,14
Δ5,8,11,14
Δ13,16
Δ7,10,13,16
Δ4,7,10,13,16
Δ9,12,15,18
Δ6,9,12,15,18
Δ9,12,15 3.1.2.2 α-Linolenic acid
Δ11,14,17 3.1.2.- Icosatrienoic acid (ETA)
Δ5,8,11,14,17 3.1.2.2 Icosapentaenoic acid (EPA)
Δ7,10,13,16,19 3.1.2.- Docosapentaenoic acid (DPA)
Δ4,7,10,13,16,19 3.1.2.2 Docosahexaenoic acid (DHA)
Δ11,14 3.1.2.- Icosadienoic acid
Δ13,16 3.1.2.- Docosadienoic acid
Δ6,9,12 3.1.2.2 γ-Linolenic acid
Δ8,11,14 3.1.2.2 Dihimo-γ-linolenic acid
Δ5,8,11,14 3.1.2.2 Arachidonic acid
Δ7,10,13,16 3.1.2.- Adrenic acid
C16:0 3.1.2.2 TesA TesB YciA Palmitic acid
C18:0 3.1.2.2 TesA TesB Stearic acid
C20:0 3.1.2.2 Arachidic acid
C22:0 3.1.2.- Behenic acid
C24:0 3.1.2.- Lignoceric acid
Δ9 3.1.2.2 TesA TesB Oleic acid
Δ11 3.1.2.- Icosenoic acid
Δ13 3.1.2.- Erucic acid
Δ15 3.1.2.- Nervonic acid
Δ9,12 3.1.2.2 Linoleic acid
01040 5/19/15
(c) Kanehisa Laboratories

BIOSYNTHESIS OF ANSAMYCINS

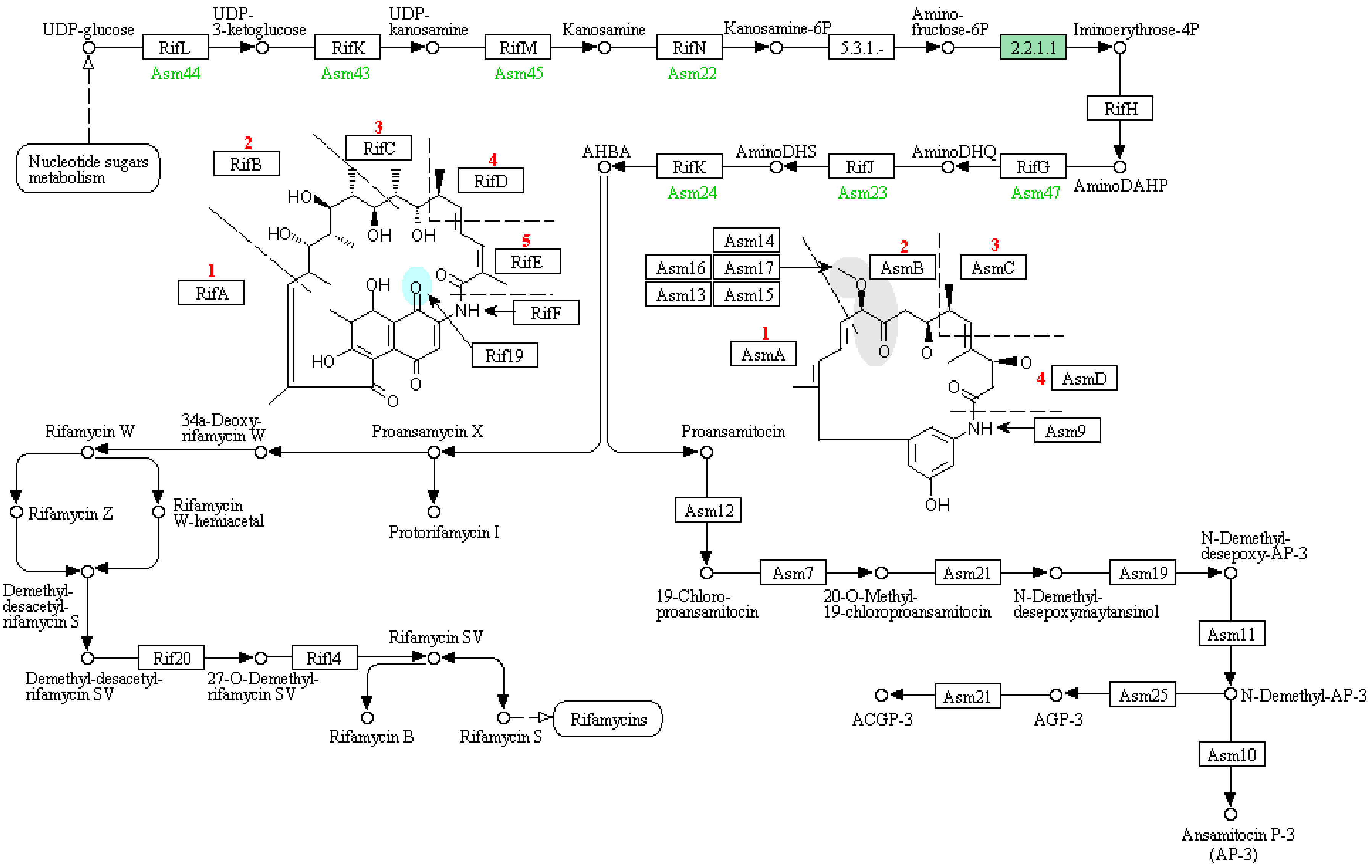

01051 7/29/13

(c) Kanehisa Laboratories

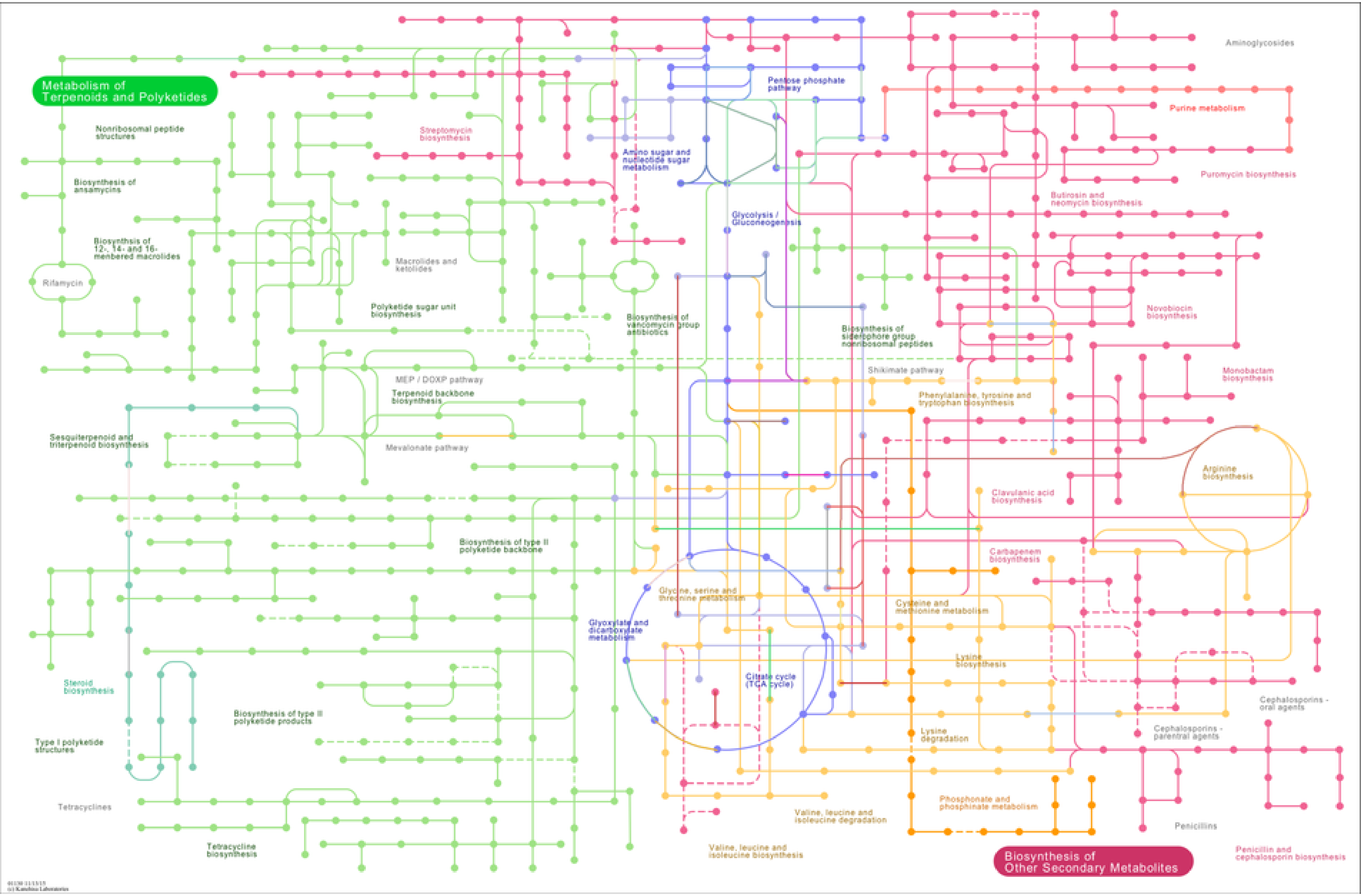
Metabolism of Terpenoids and Polyketides
Nonribosomal peptide structures
Biosynthesis of ansamycins
Biosynthesis of 12-, 14- and 16-membered macrolides
Rifamycin
Streptomycin biosynthesis
Macrolides and ketolides
Polyketide sugar unit biosynthesis
Amino sugar and nucleotide sugar metabolism
Pentose phosphate pathway
Glycolysis / Gluconeogenesis
Biosynthesis of vancomycin group antibiotics
Biosynthesis of siderophore group nonribosomal peptides
Shikimate pathway
Phenylalanine, tyrosine and tryptophan biosynthesis
Aminoglycosides
Purine metabolism
Puromycin biosynthesis
Butirosin and neomycin biosynthesis
Novobiocin biosynthesis
Monobactam biosynthesis
Arginine biosynthesis
Clavulanic acid biosynthesis
Carbapenem biosynthesis
MEP / DOXP pathway
Terpenoid backbone biosynthesis
Mevalonate pathway
Sesquiterpenoid and triterpenoid biosynthesis
Biosynthesis of type II polyketide backbone
Glycine, serine and threonine metabolism
Glyoxylate and dicarboxylate metabolism
Cysteine and methionine metabolism
Lysine biosynthesis
Citrate cycle (TCA cycle)
Steroid biosynthesis
Biosynthesis of type II polyketide products
Type I polyketide structures
Tetracyclines
Tetracycline biosynthesis
Lysine degradation
Phosphonate and phosphinate metabolism
Valine, leucine and isoleucine degradation
Valine, leucine and isoleucine biosynthesis
Cephalosporins - oral agents
Cephalosporins - parenteral agents
Penicillins
Biosynthesis of Other Secondary Metabolites
Penicillin and cephalosporin biosynthesis

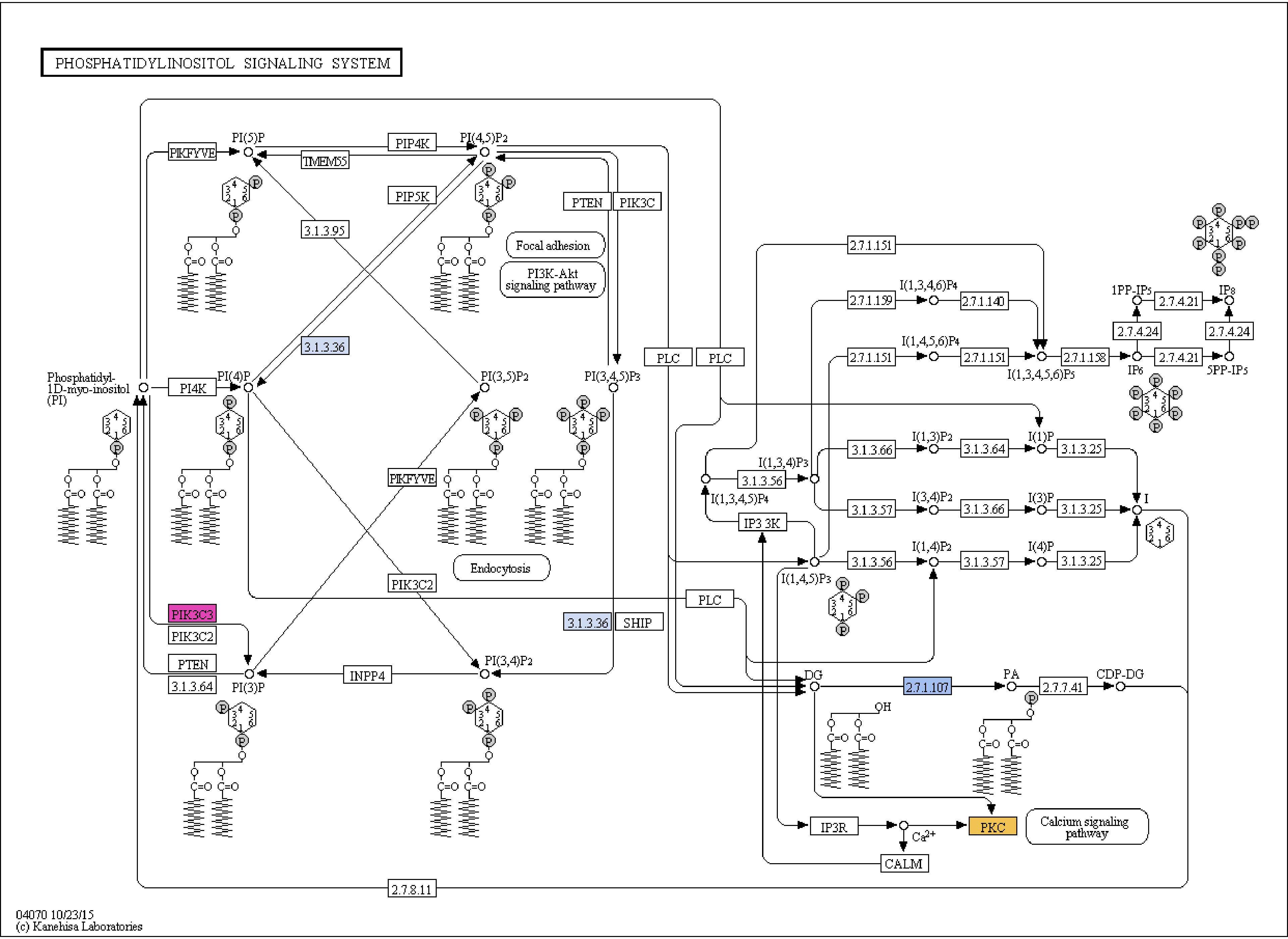
PHOSPHATIDYLINOSITOL SIGNALING SYSTEM
PI(5)P
PIKFYVE
PIP4K
PI(4,5)P2
TMEM55
PIP5K
3.1.3.95
PTEN
PIK3C
Focal adhesion
PI3K-Akt signaling pathway
3.1.3.36
Phosphatidyl-1D-myo-inositol (PI)
PI4K
PI(4)P
PI(3,5)P2
PI(3,4,5)P3
PLC
PLC
PIKFYVE
Endocytosis
PIK3C2
PLC
PIK3C3
PIK3C2
3.1.3.36
SHIP
PTEN
3.1.3.64
PI(3)P
INPP4
PI(3,4)P2
2.7.8.11
2.7.1.151
2.7.1.159
I(1,3,4,6)P4
2.7.1.140
2.7.1.151
I(1,4,5,6)P4
2.7.1.151
I(1,3,4,5,6)P5
2.7.1.158
IP6
2.7.4.21
5PP-IP5
1PP-IP5
2.7.4.21
IP8
2.7.4.24
2.7.4.24
I(1,3,4)P3
3.1.3.56
I(1,3,4,5)P4
IP3 3K
3.1.3.66
I(1,3)P2
3.1.3.64
I(1)P
3.1.3.25
3.1.3.57
I(3,4)P2
3.1.3.66
I(3)P
3.1.3.25
I
3.1.3.56
I(1,4)P2
3.1.3.57
I(4)P
3.1.3.25
I(1,4,5)P3
DG
2.7.1.107
PA
2.7.7.41
CDP-DG
IP3R
Ca2+
PKC
Calcium signaling pathway
CALM
04070 10/23/15
(c) Kanehisa Laboratories

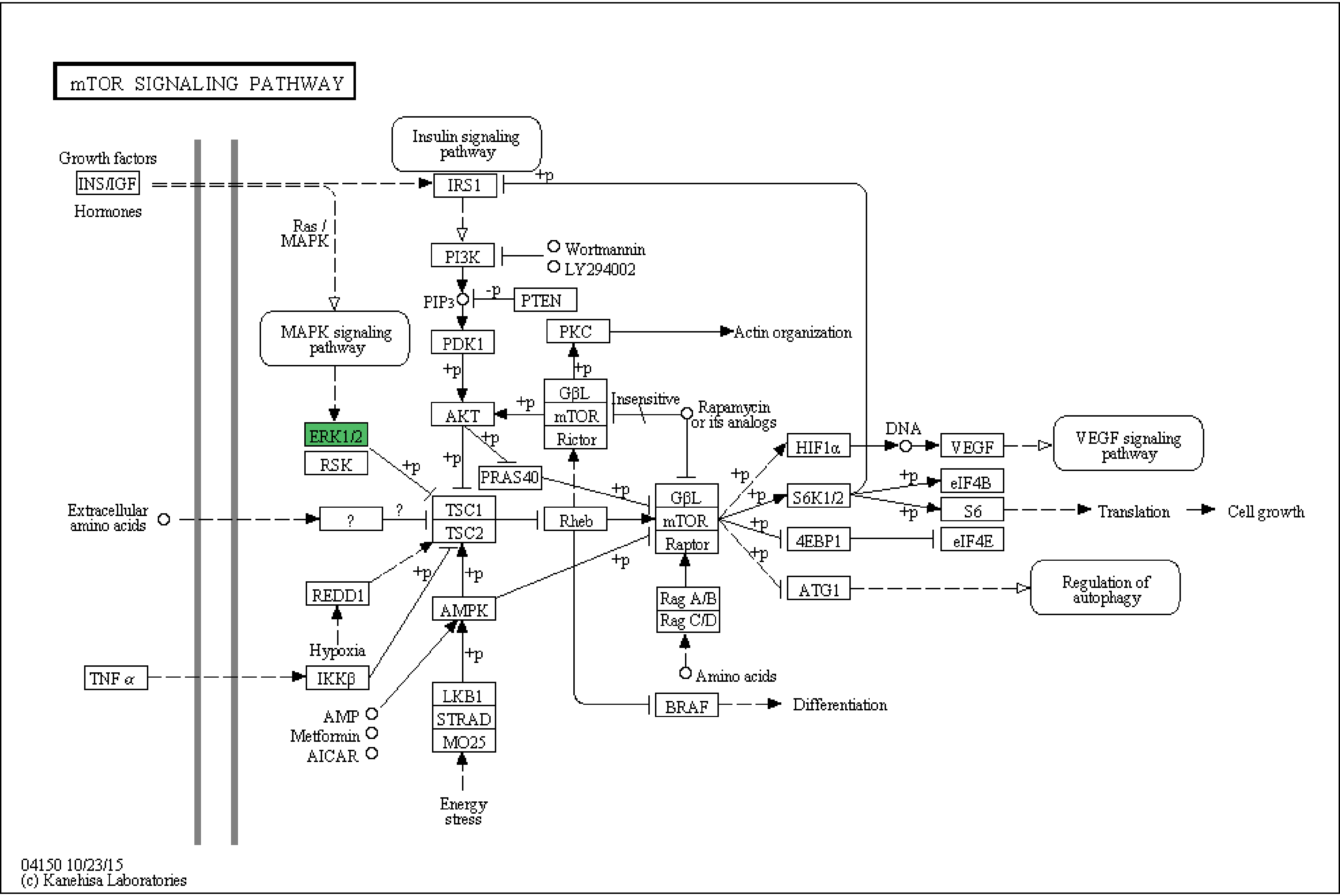
mTOR SIGNALING PATHWAY
Growth factors
INS/IGF
Hormones
Insulin signaling pathway
IRS1
+p
Ras / MAPK
PI3K
Wortmannin
LY294002
PIP3
-p
PTEN
MAPK signaling pathway
PDK1
PKC
Actin organization
+p
AKT
GβL
mTOR
Rictor
Insensitive
Rapamycin or its analogs
ERK1/2
RSK
PRAS40
DNA
HIF1α
VEGF
VEGF signaling pathway
S6K1/2
eIF4B
S6
Translation
Cell growth
Extracellular amino acids
?
TSC1
TSC2
Rheb
GβL
mTOR
Raptor
4EBP1
eIF4E
ATG1
Regulation of autophagy
REDD1
AMPK
Rag A/B
Rag C/D
Hypoxia
Amino acids
TNF α
IKKβ
LKB1
STRAD
MO25
BRAF
Differentiation
AMP
Metformin
AICAR
Energy stress
04150 10/23/15
(c) Kanehisa Laboratories

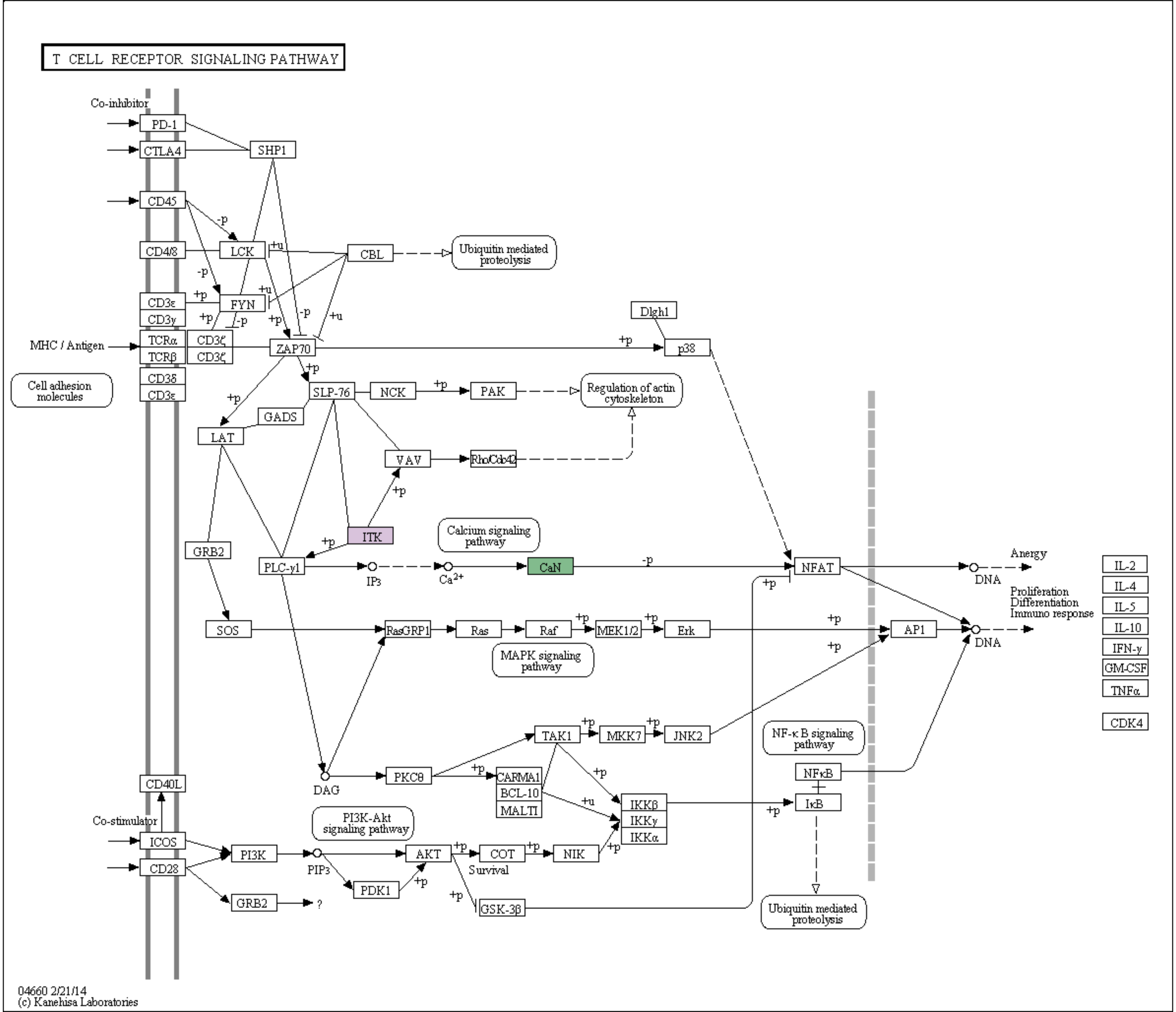
T CELL RECEPTOR SIGNALING PATHWAY
Co-inhibitor
PD-1
CTLA4
SHP1
CD45
CD4/8
LCK
CBL
Ubiquitin mediated proteolysis
CD3ε
CD3γ
FYN
Dlgh1
MHC / Antigen
TCRα
TCRβ
CD3ζ
CD3ζ
ZAP70
p38
CD3δ
CD3ε
Cell adhesion molecules
SLP-76
NCK
PAK
Regulation of actin cytoskeleton
GADS
LAT
VAV
Rho/Cdc42
ITK
Calcium signaling pathway
GRB2
PLC-γ1
IP3
Ca2+
CaN
NFAT
DNA
Anergy
Proliferation
Differentiation
Immuno response
IL-2
IL-4
IL-5
IL-10
IFN-γ
GM-CSF
TNFα
CDK4
SOS
RasGRP1
Ras
Raf
MEK1/2
Erk
AP1
MAPK signaling pathway
TAK1
MKK7
JNK2
NF-κB signaling pathway
NFκB
IκB
DAG
PKCθ
CARMA1
BCL-10
MALT1
IKKβ
IKKγ
IKKα
CD40L
Co-stimulator
PI3K-Akt signaling pathway
ICOS
CD28
PI3K
PIP3
AKT
COT
NIK
Survival
PDK1
GRB2
GSK-3β
Ubiquitin mediated proteolysis
04660 2/21/14
(c) Kanehisa Laboratories
