# Supplementary material for: Genome-wide expression profiling in leaves and roots of date palm (Phoenix dactylifera L.) exposed to salinity
Source: BMC Genomics. 2017 Mar 22;18:246. doi: 10.1186/s12864-017-3633-6 (PMC5423419; doi:10.1186/s12864-017-3633-6)
Supplement: Supplementary file 9 — Figure S5. Mapping of differentially expressed enzymes in roots due to salinity stress on the KEGG. (PDF 6204 kb) [file 12864_2017_3633_MOESM9_ESM.pdf]

Figure S5. Mapping of differentially expressed enzymes in roots due to salinity stress on the KEGG.

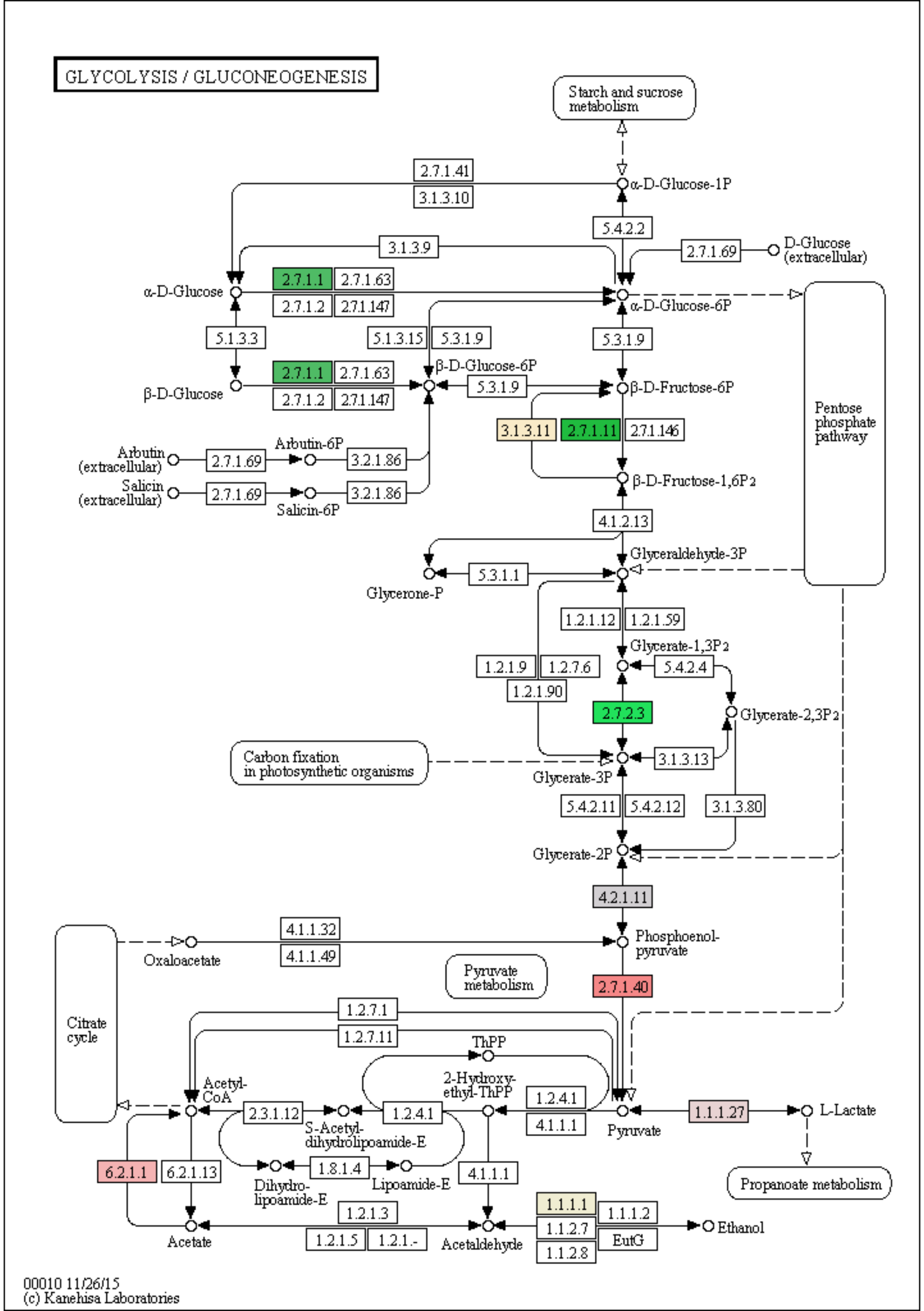
GLYCOLYSIS / GLUCONEOGENESIS
Starch and sucrose metabolism
2.7.1.41
3.1.3.10
α-D-Glucose-1P
5.4.2.2
3.1.3.9
2.7.1.69
D-Glucose (extracellular)
2.7.1.1
2.7.1.63
2.7.1.2
2.7.1.147
α-D-Glucose
α-D-Glucose-6P
5.1.3.3
5.1.3.15
5.3.1.9
5.3.1.9
2.7.1.1
2.7.1.63
β-D-Glucose-6P
2.7.1.2
2.7.1.147
β-D-Glucose
5.3.1.9
β-D-Fructose-6P
3.1.3.11
2.7.1.11
2.7.1.146
Pentose phosphate pathway
Arbutin (extracellular)
2.7.1.69
Arbutin-6P
3.2.1.86
Salicin (extracellular)
2.7.1.69
Salicin-6P
3.2.1.86
β-D-Fructose-1,6P2
4.1.2.13
Glyceraldehyde-3P
5.3.1.1
Glycerone-P
1.2.1.12
1.2.1.59
Glycerate-1,3P2
1.2.1.9
1.2.7.6
5.4.2.4
1.2.1.90
2.7.2.3
Glycerate-2,3P2
Carbon fixation in photosynthetic organisms
3.1.3.13
Glycerate-3P
5.4.2.11
5.4.2.12
3.1.3.80
Glycerate-2P
4.2.1.11
4.1.1.32
4.1.1.49
Oxaloacetate
Phosphoenol-pyruvate
Pyruvate metabolism
2.7.1.40
Citrate cycle
1.2.7.1
1.2.7.11
ThPP
2-Hydroxy-ethyl-ThPP
Acetyl-CoA
2.3.1.12
1.2.4.1
1.2.4.1
4.1.1.1
Pyruvate
1.1.1.27
L-Lactate
S-Acetyl-dihydrolipoamide-E
1.8.1.4
Lipoamide-E
Dihydro-lipoamide-E
6.2.1.1
6.2.1.13
4.1.1.1
Propanoate metabolism
1.2.1.3
1.1.1.1
1.1.1.2
Acetate
1.2.1.5
1.2.1.-
Acetaldehyde
1.1.2.7
EutG
Ethanol
1.1.2.8
00010 11/26/15
(c) Kanehisa Laboratories

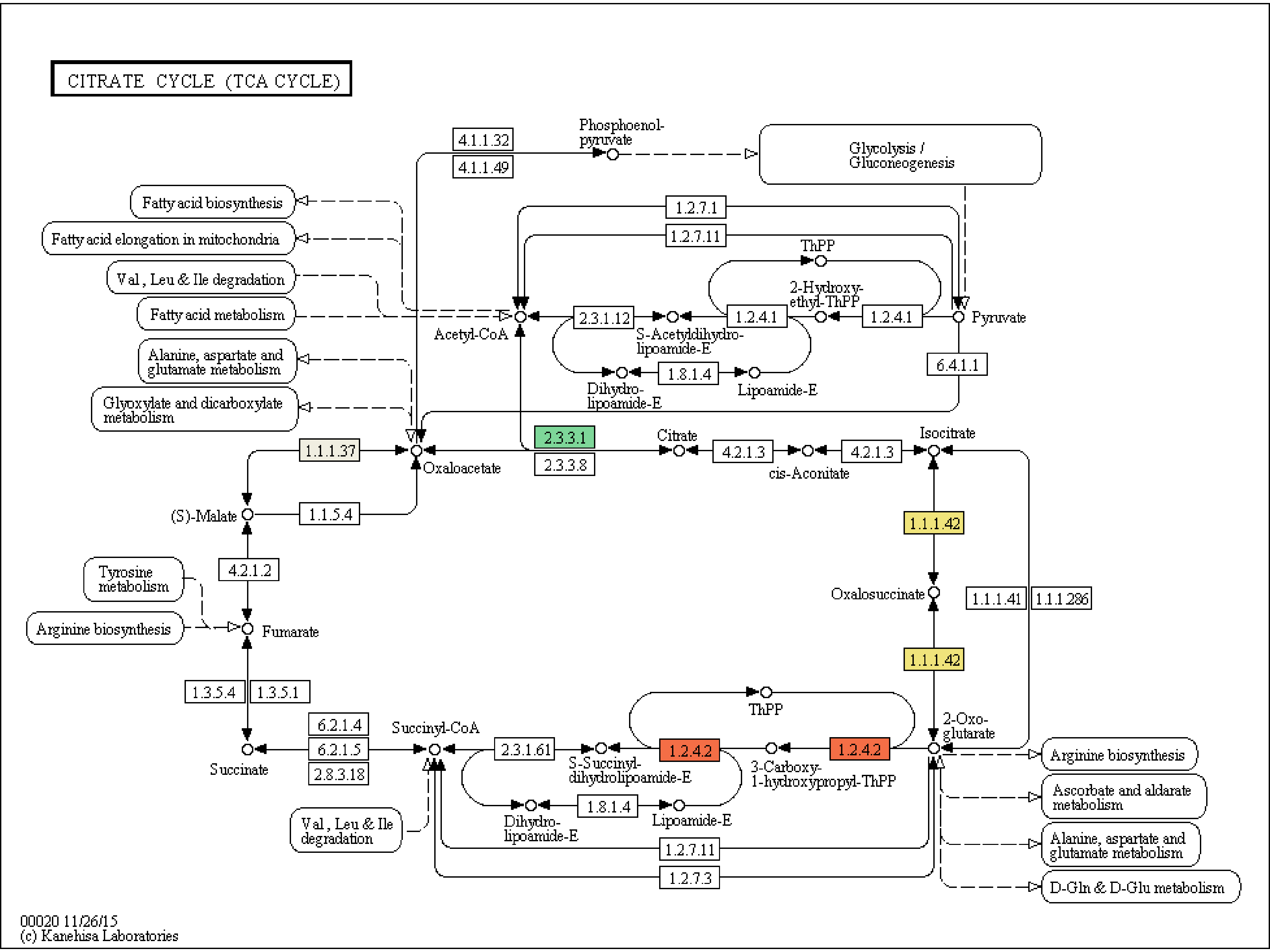
CITRATE CYCLE (TCA CYCLE)
4.1.1.32
4.1.1.49
Phosphoenol-pyruvate
Glycolysis / Gluconeogenesis
Fatty acid biosynthesis
Fatty acid elongation in mitochondria
Val, Leu & Ile degradation
Fatty acid metabolism
Alanine, aspartate and glutamate metabolism
Glyoxylate and dicarboxylate metabolism
1.2.7.1
1.2.7.11
ThPP
2-Hydroxy-ethyl-ThPP
2.3.1.12
1.2.4.1
1.2.4.1
Acetyl-CoA
S-Acetyldihydro-lipoamide-E
Pyruvate
1.8.1.4
Dihydro-lipoamide-E
Lipoamide-E
6.4.1.1
2.3.3.1
2.3.3.8
Citrate
4.2.1.3
4.2.1.3
cis-Aconitate
Isocitrate
1.1.1.37
Oxaloacetate
(S)-Malate
1.1.5.4
1.1.1.42
Oxalosuccinate
1.1.1.41
1.1.1.286
1.1.1.42
Tyrosine metabolism
Arginine biosynthesis
4.2.1.2
Fumarate
1.3.5.4
1.3.5.1
6.2.1.4
6.2.1.5
2.8.3.18
Succinate
Succinyl-CoA
2.3.1.61
S-Succinyl-dihydrolipoamide-E
1.2.4.2
1.2.4.2
ThPP
3-Carboxy-1-hydroxypropyl-ThPP
2-Oxo-glutarate
1.8.1.4
Dihydro-lipoamide-E
Lipoamide-E
Val, Leu & Ile degradation
1.2.7.11
1.2.7.3
Arginine biosynthesis
Ascorbate and aldarate metabolism
Alanine, aspartate and glutamate metabolism
D-Gln & D-Glu metabolism
00020 11/26/15
(c) Kanehisa Laboratories

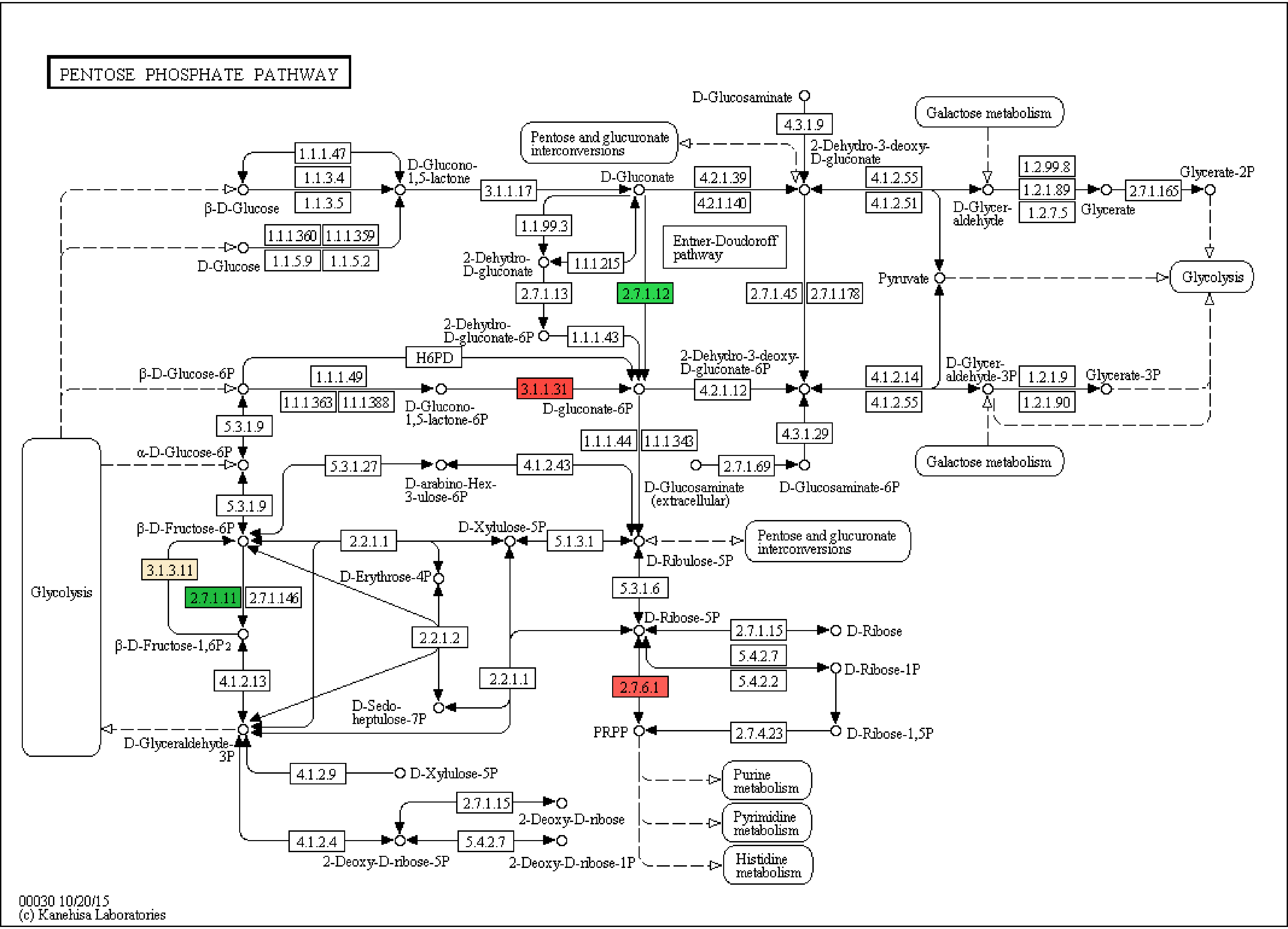
PENTOSE PHOSPHATE PATHWAY
D-Glucosaminate
4.3.1.9
Galactose metabolism
Pentose and glucuronate interconversions
2-Dehydro-3-deoxy-D-gluconate
1.1.1.47
1.1.3.4
1.1.3.5
D-Glucono-1,5-lactone
3.1.1.17
D-Gluconate
4.2.1.39
4.2.1.140
4.1.2.55
4.1.2.51
1.2.99.8
1.2.1.89
1.2.7.5
Glycerate-2P
2.7.1.165
Glycerate
D-Glycer-aldehyde
β-D-Glucose
1.1.1.360
1.1.1.359
1.1.5.9
1.1.5.2
D-Glucose
1.1.99.3
2-Dehydro-D-gluconate
1.1.1.215
Entner-Doudoroff pathway
2.7.1.12
2.7.1.13
2.7.1.45
2.7.1.178
Pyruvate
Glycolysis
2-Dehydro-D-gluconate-6P
1.1.1.43
H6PD
2-Dehydro-3-deoxy-D-gluconate-6P
D-Glycer-aldehyde-3P
1.2.1.9
Glycerate-3P
β-D-Glucose-6P
1.1.1.49
1.1.1.363
1.1.1.388
D-Glucono-1,5-lactone-6P
3.1.1.31
D-gluconate-6P
4.2.1.12
4.1.2.14
4.1.2.55
1.2.1.90
5.3.1.9
1.1.1.44
1.1.1.343
4.3.1.29
α-D-Glucose-6P
5.3.1.27
4.1.2.43
2.7.1.69
D-arabino-Hex-3-ulose-6P
D-Glucosaminate (extracellular)
D-Glucosamine-6P
5.3.1.9
β-D-Fructose-6P
D-Xylulose-5P
2.2.1.1
5.1.3.1
Pentose and glucuronate interconversions
D-Ribulose-5P
3.1.3.11
D-Erythrose-4P
Glycolysis
2.7.1.11
2.7.1.146
5.3.1.6
D-Ribose-5P
2.7.1.15
D-Ribose
2.2.1.2
β-D-Fructose-1,6P2
5.4.2.7
D-Ribose-1P
5.4.2.2
4.1.2.13
2.2.1.1
2.7.6.1
D-Sedo-heptulose-7P
PRPP
2.7.4.23
D-Ribose-1,5P
D-Glyceraldehyde-3P
4.1.2.9
D-Xylulose-5P
Purine metabolism
2.7.1.15
2-Deoxy-D-ribose
Pyrimidine metabolism
4.1.2.4
5.4.2.7
2-Deoxy-D-ribose-5P
2-Deoxy-D-ribose-1P
Histidine metabolism
00030 10/20/15
(c) Kanehisa Laboratories

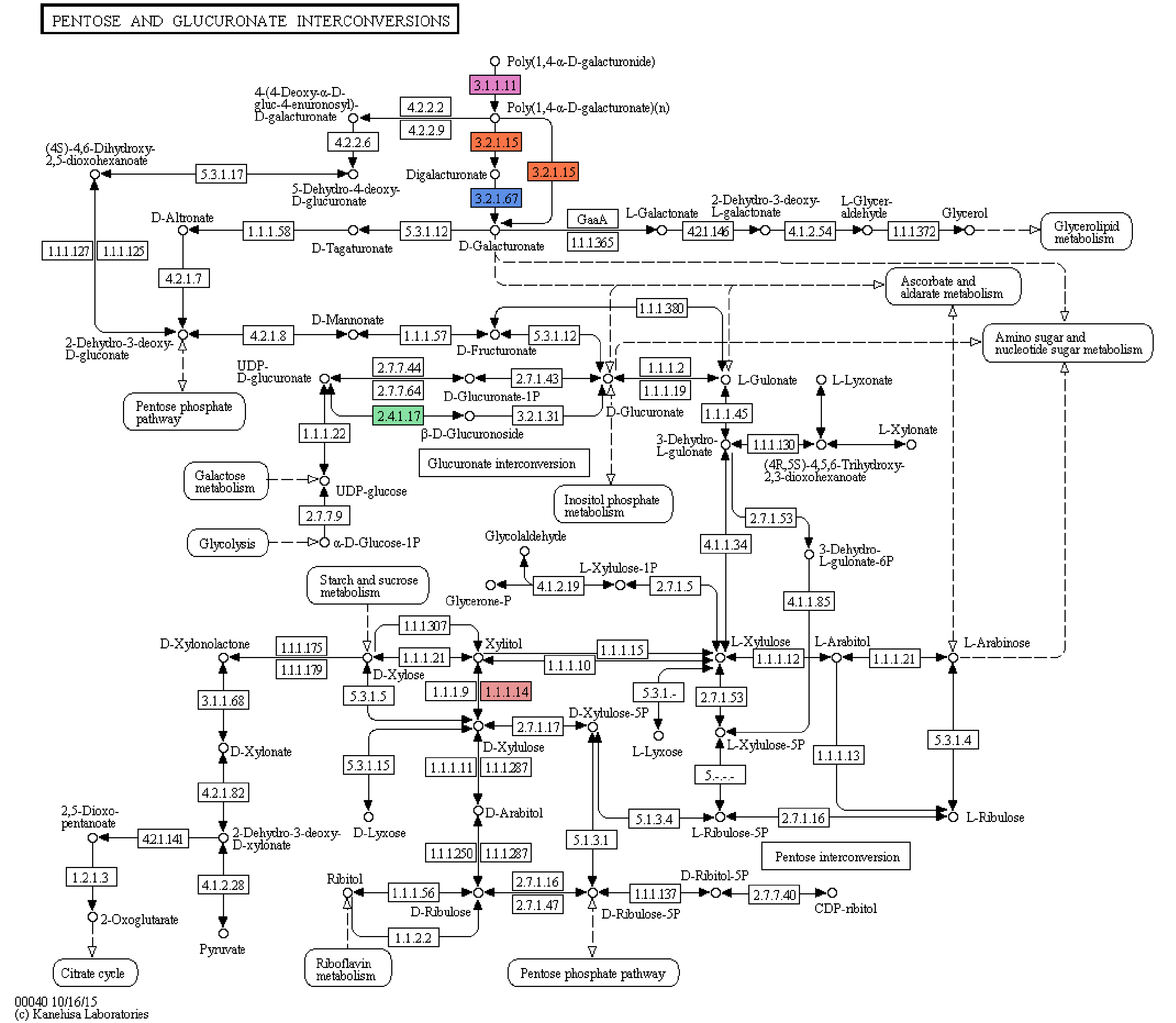
PENTOSE AND GLUCURONATE INTERCONVERSIONS
Poly(1,4-α-D-galacturonide)
3.1.1.11
Poly(1,4-α-D-galacturonate)(n)
4-(4-Deoxy-α-D-gluc-4-enuronosyl)-D-galacturonate
4.2.2.2
4.2.2.9
4.2.2.6
3.2.1.15
3.2.1.15
Digalacturonate
3.2.1.67
(4S)-4,6-Dihydroxy-2,5-dioxohexanoate
5.3.1.17
5-Dehydro-4-deoxy-D-glucuronate
D-Altronate
1.1.1.58
D-Tagaturonate
5.3.1.12
D-Galacturonate
GaaA
1.1.1.365
L-Galactonate
4.2.1.146
2-Dehydro-3-deoxy-L-galactonate
4.1.2.54
L-Glyceraldehyde
1.1.1.372
Glycerol
Glycerolipid metabolism
1.1.1.127
1.1.1.125
4.2.1.7
Ascorbate and aldarate metabolism
1.1.1.380
2-Dehydro-3-deoxy-D-gluconate
4.2.1.8
D-Mannonate
1.1.1.57
D-Fructuronate
5.3.1.12
Amino sugar and nucleotide sugar metabolism
UDP-D-glucuronate
2.7.7.44
2.7.7.64
D-Glucuronate-1P
2.7.1.43
1.1.1.2
1.1.1.19
L-Gulonate
L-Lyxonate
Pentose phosphate pathway
2.4.1.17
β-D-Glucuronoside
3.2.1.31
D-Glucuronate
1.1.1.45
1.1.1.22
3-Dehydro-L-gulonate
1.1.1.130
L-Xylonate
(4R,5S)-4,5,6-Trihydroxy-2,3-dioxohexanoate
Glucuronate interconversion
Galactose metabolism
UDP-glucose
Inositol phosphate metabolism
2.7.7.9
2.7.1.53
Glycolysis
α-D-Glucose-1P
Glycolaldehyde
4.1.1.34
3-Dehydro-L-gulonate-6P
L-Xylulose-1P
Starch and sucrose metabolism
4.1.2.19
2.7.1.5
Glycerone-P
4.1.1.85
1.1.1.307
D-Xylonolactone
1.1.1.175
1.1.1.179
Xylitol
1.1.1.15
L-Xylulose
L-Arabitol
L-Arabinose
1.1.1.21
D-Xylose
1.1.1.10
1.1.1.12
1.1.1.21
1.1.1.9
1.1.1.14
3.1.1.68
5.3.1.5
5.3.1.-
2.7.1.53
D-Xylulose-5P
2.7.1.17
D-Xylonate
L-Lyxose
L-Xylulose-5P
D-Xylulose
5.3.1.4
5.3.1.15
1.1.1.11
1.1.1.287
1.1.1.13
5.-.-.-
4.2.1.82
D-Arabitol
5.1.3.4
2.7.1.16
L-Ribulose
2,5-Dioxopentanoate
L-Ribulose-5P
4.2.1.41
2-Dehydro-3-deoxy-D-xylonate
D-Lyxose
5.1.3.1
Pentose interconversion
1.1.1.250
1.1.1.287
1.2.1.3
4.1.2.28
Ribitol
1.1.1.56
2.7.1.16
D-Ribitol-5P
1.1.1.137
2.7.7.40
CDP-ribitol
2.7.1.47
D-Ribulose
D-Ribulose-5P
2-Oxoglutarate
Pyruvate
1.1.2.2
Citrate cycle
Riboflavin metabolism
Pentose phosphate pathway
00040 10/16/15
(c) Kanehisa Laboratories

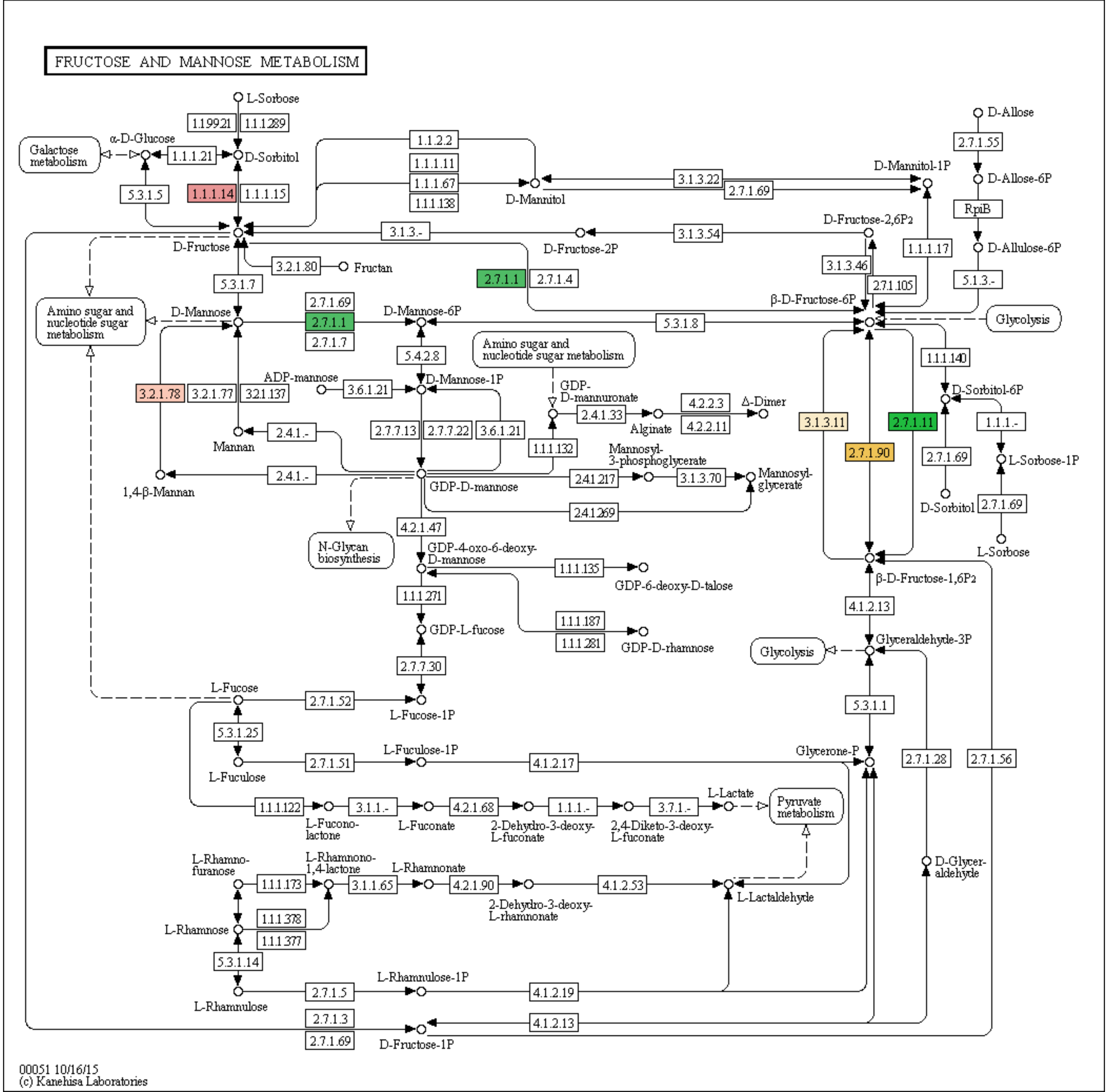
FRUCTOSE AND MANNOSE METABOLISM
L-Sorbose
1.1.99.21
1.1.1.289
Galactose metabolism
α-D-Glucose
1.1.1.21
D-Sorbitol
5.3.1.5
1.1.1.14
1.1.1.15
1.1.2.2
1.1.1.11
1.1.1.67
1.1.1.138
D-Mannitol
3.1.3.22
2.7.1.69
D-Mannitol-1P
D-Allose
2.7.1.55
D-Allose-6P
RpiB
D-Allulose-6P
5.1.3.-
3.1.3.-
D-Fructose
D-Fructose-2P
3.1.3.54
D-Fructose-2,6P2
1.1.1.17
3.2.1.80
Fructan
2.7.1.1
2.7.1.4
3.1.3.46
2.7.1.105
5.3.1.7
β-D-Fructose-6P
Glycolysis
Amino sugar and nucleotide sugar metabolism
D-Mannose
2.7.1.69
2.7.1.1
2.7.1.7
D-Mannose-6P
5.3.1.8
5.4.2.8
Amino sugar and nucleotide sugar metabolism
1.1.1.140
D-Sorbitol-6P
3.2.1.78
3.2.1.77
3.2.1.137
ADP-mannose
3.6.1.21
D-Mannose-1P
GDP-D-mannuronate
2.4.1.33
Alginate
4.2.2.3
4.2.2.11
Δ-Dimer
3.1.3.11
2.7.1.11
1.1.1.-
Mannan
2.4.1.-
2.7.7.13
2.7.7.22
3.6.1.21
1.1.1.132
2.7.1.90
2.7.1.69
L-Sorbose-1P
Mannosyl-3-phosphoglycerate
1,4-β-Mannan
2.4.1.-
GDP-D-mannose
2.4.1.217
3.1.3.70
Mannosyl-glycerate
D-Sorbitol
2.7.1.69
2.4.1.269
N-Glycan biosynthesis
4.2.1.47
GDP-4-oxo-6-deoxy-D-mannose
1.1.1.135
GDP-6-deoxy-D-talose
L-Sorbose
β-D-Fructose-1,6P2
1.1.1.271
1.1.1.187
1.1.1.281
GDP-D-rhamnose
4.1.2.13
GDP-L-fucose
Glycolysis
Glyceraldehyde-3P
2.7.7.30
L-Fucose
2.7.1.52
L-Fucose-1P
5.3.1.1
5.3.1.25
L-Fuculose-1P
Glycerone-P
2.7.1.28
2.7.1.56
L-Fuculose
2.7.1.51
4.1.2.17
1.1.1.122
3.1.1.-
4.2.1.68
1.1.1.-
3.7.1.-
L-Lactate
Pyruvate metabolism
L-Fucono-lactone
L-Fuconate
2-Dehydro-3-deoxy-L-fuconate
2,4-Diketo-3-deoxy-L-fuconate
L-Rhamno-furanose
L-Rhamnono-1,4-lactone
L-Rhamnonate
D-Glycer-aldehyde
1.1.1.173
3.1.1.65
4.2.1.90
4.1.2.53
L-Lactaldehyde
1.1.1.378
2-Dehydro-3-deoxy-L-rhamnonate
L-Rhamnose
1.1.1.377
5.3.1.14
L-Rhamnulose-1P
2.7.1.5
4.1.2.19
L-Rhamnulose
2.7.1.3
4.1.2.13
2.7.1.69
D-Fructose-1P
00051 10/16/15
(c) Kanehisa Laboratories

GALACTOSE METABOLISM

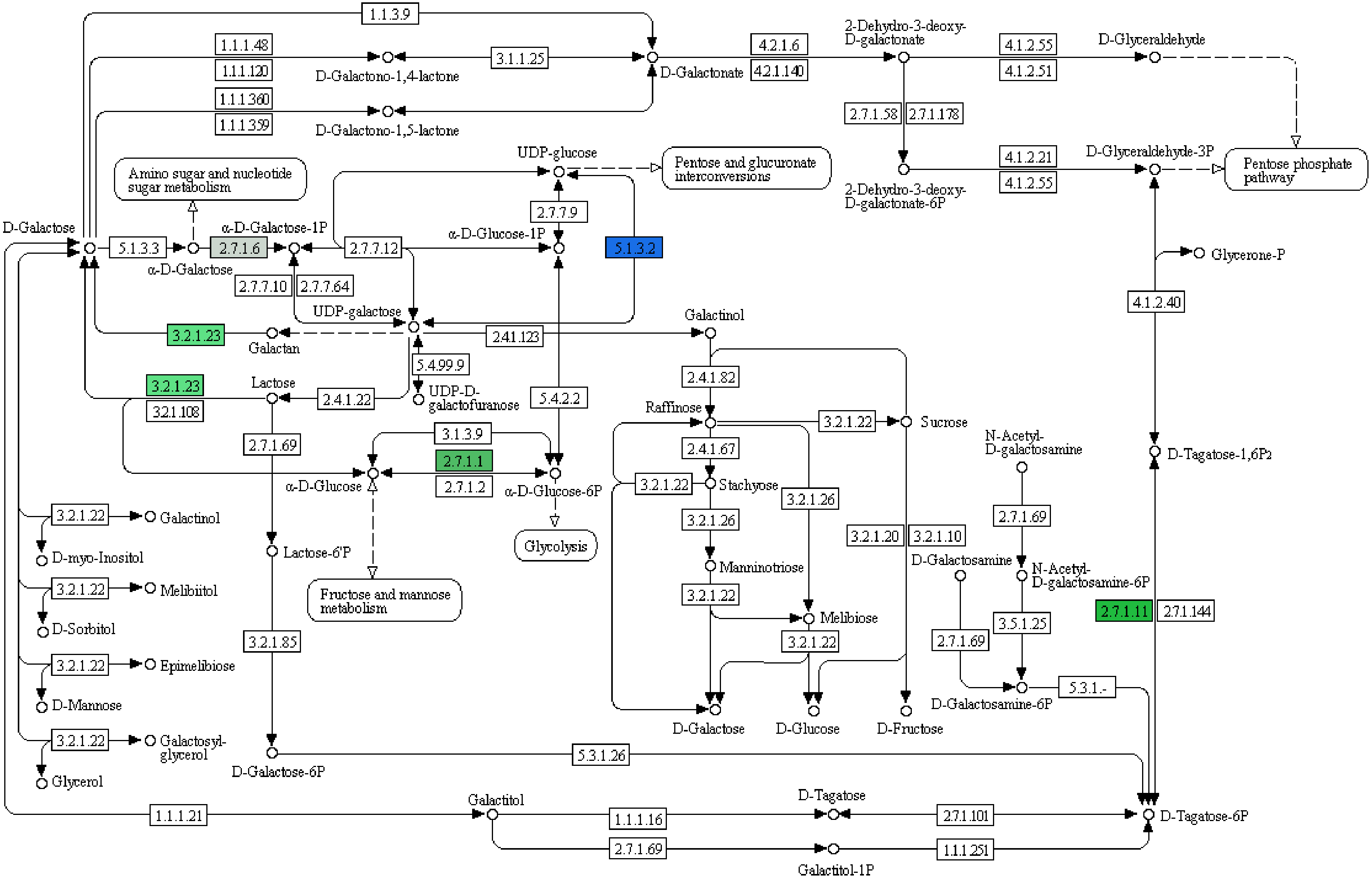

00052 12/16/14
(c) Kanehisa Laboratories

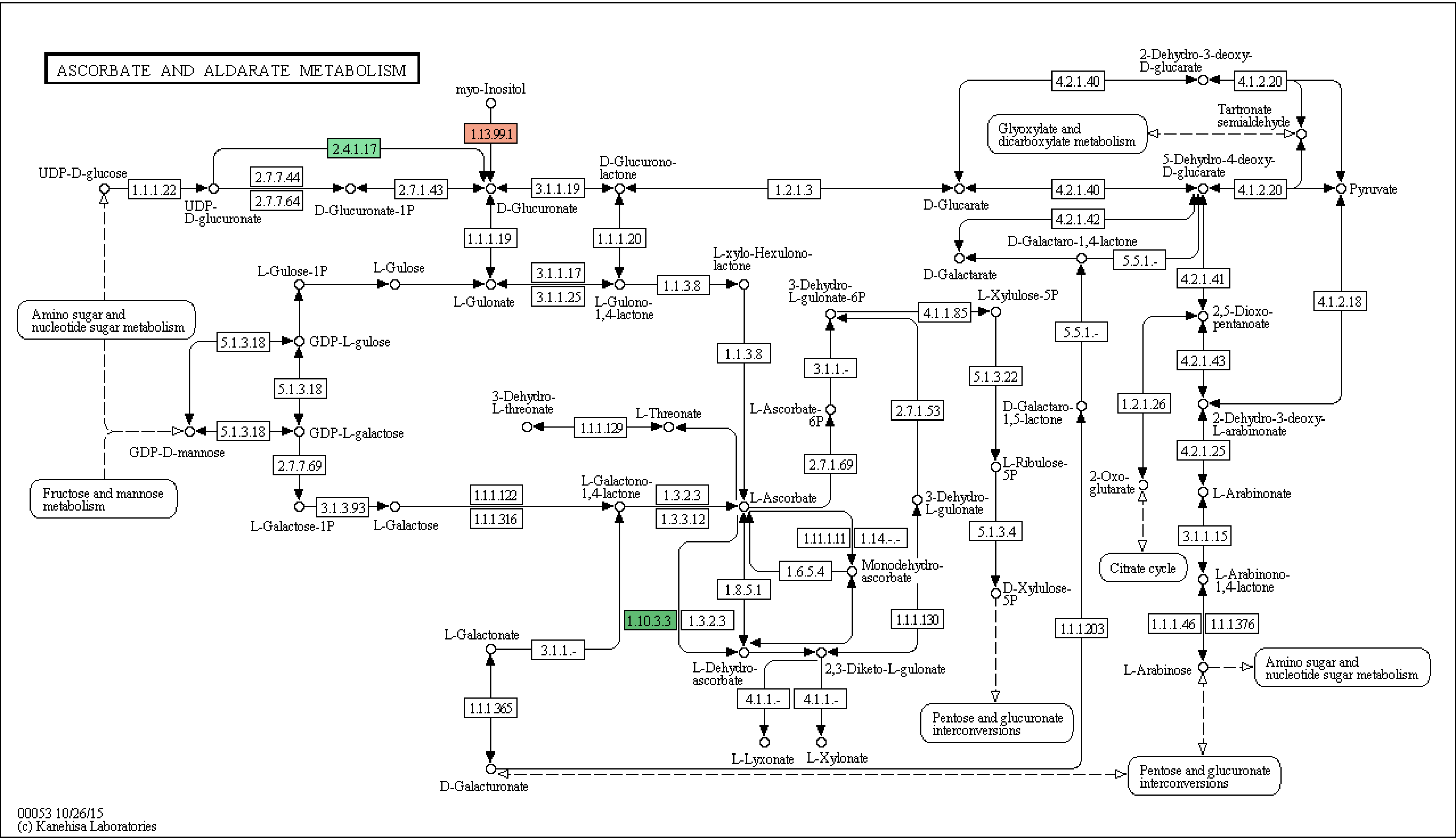

ASCORBATE AND ALDARATE METABOLISM
myo-Inositol
1.13.99.1
2.4.1.17
UDP-D-glucose
1.1.1.22
UDP-D-glucuronate
2.7.7.44
2.7.7.64
D-Glucuronate-1P
2.7.1.43
D-Glucuronate
3.1.1.19
D-Glucurono-lactone
1.2.1.3
D-Glucarate
4.2.1.40
2-Dehydro-3-deoxy-D-glucarate
4.1.2.20
Tartronate semialdehyde
Glyoxylate and dicarboxylate metabolism
5-Dehydro-4-deoxy-D-glucarate
Pyruvate
4.2.1.42
D-Galactaro-1,4-lactone
5.5.1.-
D-Galactarate
4.2.1.41
4.1.2.18
2,5-Dioxo-pentanoate
4.2.1.43
1.2.1.26
2-Dehydro-3-deoxy-L-arabinonate
4.2.1.25
2-Oxo-glutarate
L-Arabinonate
Citrate cycle
3.1.1.15
L-Arabinono-1,4-lactone
1.1.1.46
1.1.1.376
L-Arabinose
Amino sugar and nucleotide sugar metabolism
Pentose and glucuronate interconversions
1.1.1.19
1.1.1.20
L-Gulose-1P
L-Gulose
L-Gulonate
3.1.1.17
3.1.1.25
L-Gulono-1,4-lactone
1.1.3.8
L-xylo-Hexulono-lactone
3-Dehydro-L-gulonate-6P
4.1.1.85
L-Xylulose-5P
5.1.3.22
D-Galactaro-1,5-lactone
L-Ribulose-5P
5.1.3.4
D-Xylulose-5P
1.1.1.203
Amino sugar and nucleotide sugar metabolism
5.1.3.18
GDP-L-gulose
GDP-D-mannose
GDP-L-galactose
2.7.7.69
Fructose and mannose metabolism
L-Galactose-1P
3.1.3.93
L-Galactose
1.1.1.122
1.1.1.316
L-Galactono-1,4-lactone
1.3.2.3
1.3.3.12
L-Ascorbate
3-Dehydro-L-threonate
1.1.1.129
L-Threonate
3.1.1.-
L-Ascorbate-6P
2.7.1.69
2.7.1.53
3-Dehydro-L-gulonate
1.11.1.11
1.14.-.-
1.6.5.4
Monodehydro-ascorbate
1.8.5.1
1.10.3.3
1.3.2.3
1.1.1.130
L-Dehydro-ascorbate
2,3-Diketo-L-gulonate
4.1.1.-
L-Lyxonate
L-Xylonate
Pentose and glucuronate interconversions
L-Galactonate
3.1.1.-
1.1.1.365
D-Galacturonate
00053 10/26/15
(c) Kanehisa Laboratories

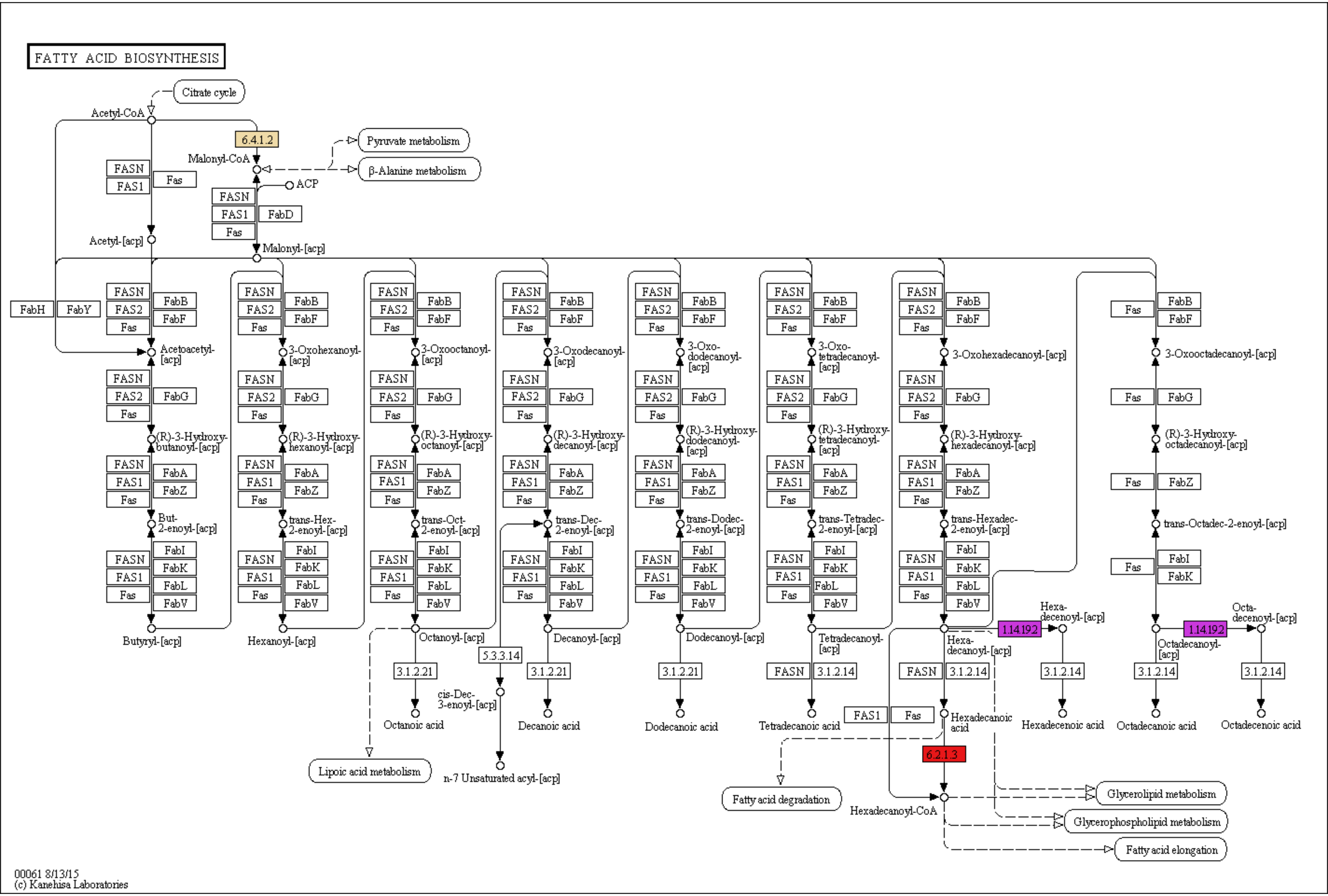
FATTY ACID BIOSYNTHESIS
Citrate cycle
Acetyl-CoA
6.4.1.2
Pyruvate metabolism
Malonyl-CoA
β-Alanine metabolism
ACP
FASN
FAS1
FAS2
Fas
FabD
FabH
FabY
FabB
FabF
FabG
FabA
FabZ
FabI
FabK
FabL
FabV
Acetyl-[acp]
Malonyl-[acp]
Acetoacetyl-[acp]
3-Oxohexanoyl-[acp]
3-Oxooctanoyl-[acp]
3-Oxodecanoyl-[acp]
3-Oxo-dodecanoyl-[acp]
3-Oxo-tetradecanoyl-[acp]
3-Oxohexadecanoyl-[acp]
3-Oxooctadecanoyl-[acp]
(R)-3-Hydroxy-butanoyl-[acp]
(R)-3-Hydroxy-hexanoyl-[acp]
(R)-3-Hydroxy-octanoyl-[acp]
(R)-3-Hydroxy-decanoyl-[acp]
(R)-3-Hydroxy-dodecanoyl-[acp]
(R)-3-Hydroxy-tetradecanoyl-[acp]
(R)-3-Hydroxy-hexadecanoyl-[acp]
(R)-3-Hydroxy-octadecanoyl-[acp]
But-2-enoyl-[acp]
trans-Hex-2-enoyl-[acp]
trans-Oct-2-enoyl-[acp]
trans-Dec-2-enoyl-[acp]
trans-Dodec-2-enoyl-[acp]
trans-Tetradec-2-enoyl-[acp]
trans-Hexadec-2-enoyl-[acp]
trans-Octadec-2-enoyl-[acp]
Butyryl-[acp]
Hexanoyl-[acp]
Octanoyl-[acp]
Decanoyl-[acp]
Dodecanoyl-[acp]
Tetradecanoyl-[acp]
Hexa-decanoyl-[acp]
Octadecanoyl-[acp]
Hexa-decenoyl-[acp]
Octa-decenoyl-[acp]
1.14.19.2
5.3.3.14
3.1.2.21
3.1.2.14
cis-Dec-3-enoyl-[acp]
n-7 Unsaturated acyl-[acp]
Octanoic acid
Decanoic acid
Dodecanoic acid
Tetradecanoic acid
Hexadecanoic acid
Hexadecenoic acid
Octadecanoic acid
Octadecenoic acid
6.2.1.3
Hexadecanoyl-CoA
Lipoic acid metabolism
Fatty acid degradation
Glycerolipid metabolism
Glycerophospholipid metabolism
Fatty acid elongation
00061 8/13/15
(c) Kanehisa Laboratories

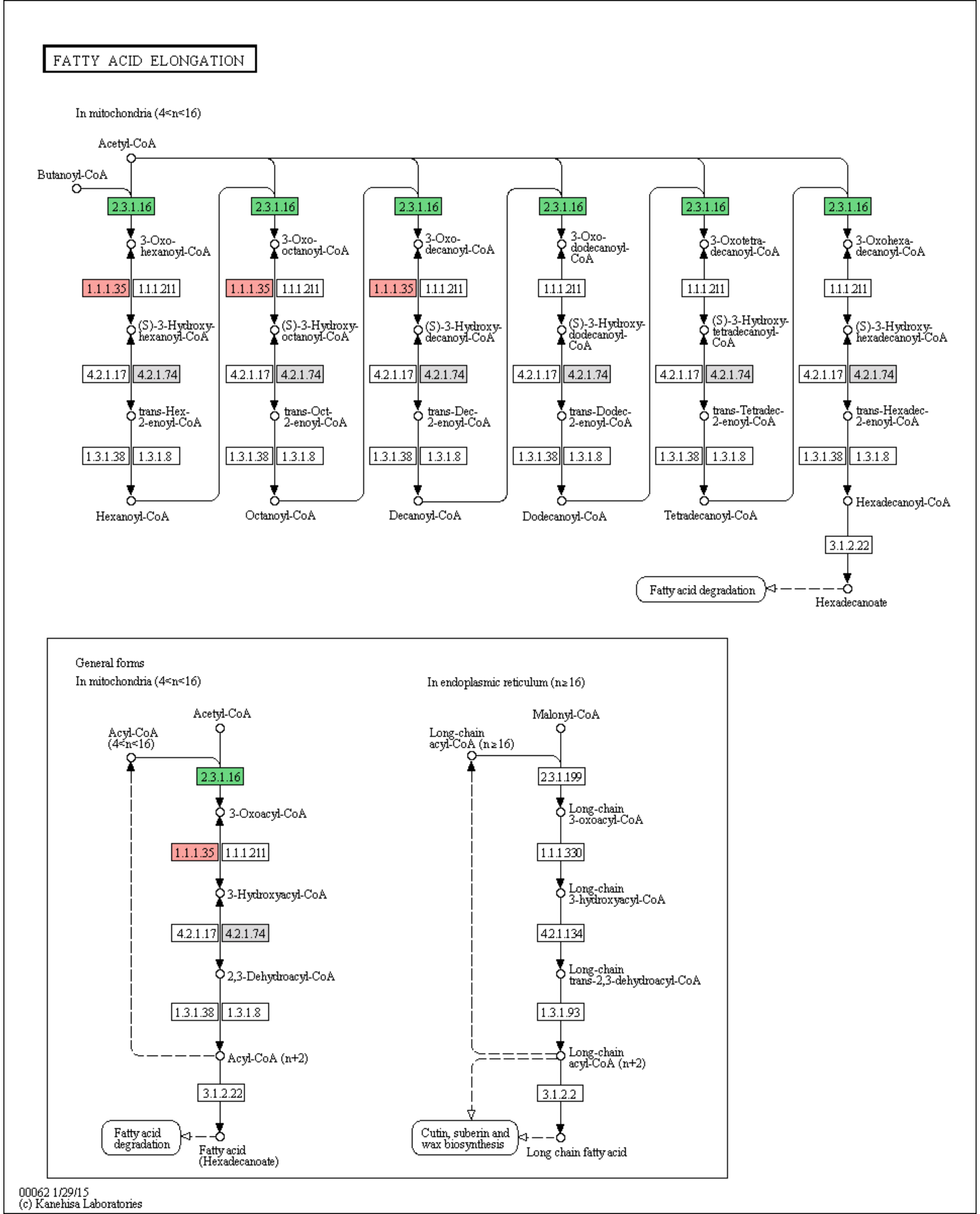
FATTY ACID ELONGATION
In mitochondria (4<n<16)
Acetyl-CoA
Butanoyl-CoA
2.3.1.16
3-Oxo-hexanoyl-CoA
1.1.1.35
1.1.1.211
(S)-3-Hydroxy-hexanoyl-CoA
4.2.1.17
4.2.1.74
trans-Hex-2-enoyl-CoA
1.3.1.38
1.3.1.8
Hexanoyl-CoA
3-Oxo-octanoyl-CoA
(S)-3-Hydroxy-octanoyl-CoA
trans-Oct-2-enoyl-CoA
Octanoyl-CoA
3-Oxo-decanoyl-CoA
(S)-3-Hydroxy-decanoyl-CoA
trans-Dec-2-enoyl-CoA
Decanoyl-CoA
3-Oxo-dodecanoyl-CoA
(S)-3-Hydroxy-dodecanoyl-CoA
trans-Dodec-2-enoyl-CoA
Dodecanoyl-CoA
3-Oxotetra-decanoyl-CoA
(S)-3-Hydroxy-tetradecanoyl-CoA
trans-Tetradec-2-enoyl-CoA
Tetradecanoyl-CoA
3-Oxohexa-decanoyl-CoA
(S)-3-Hydroxy-hexadecanoyl-CoA
trans-Hexadec-2-enoyl-CoA
Hexadecanoyl-CoA
3.1.2.22
Fatty acid degradation
Hexadecanoate
General forms
In mitochondria (4<n<16)
Acetyl-CoA
Acyl-CoA (4<n<16)
2.3.1.16
3-Oxoacyl-CoA
1.1.1.35
1.1.1.211
3-Hydroxyacyl-CoA
4.2.1.17
4.2.1.74
2,3-Dehydroacyl-CoA
1.3.1.38
1.3.1.8
Acyl-CoA (n+2)
3.1.2.22
Fatty acid degradation
Fatty acid (Hexadecanoate)
In endoplasmic reticulum (n≥16)
Malonyl-CoA
Long-chain acyl-CoA (n≥16)
2.3.1.199
Long-chain 3-oxoacyl-CoA
1.1.1.330
Long-chain 3-hydroxyacyl-CoA
4.2.1.134
Long-chain trans-2,3-dehydroacyl-CoA
1.3.1.93
Long-chain acyl-CoA (n+2)
3.1.2.2
Cutin, suberin and wax biosynthesis
Long chain fatty acid
00062 1/29/15
(c) Kanehisa Laboratories

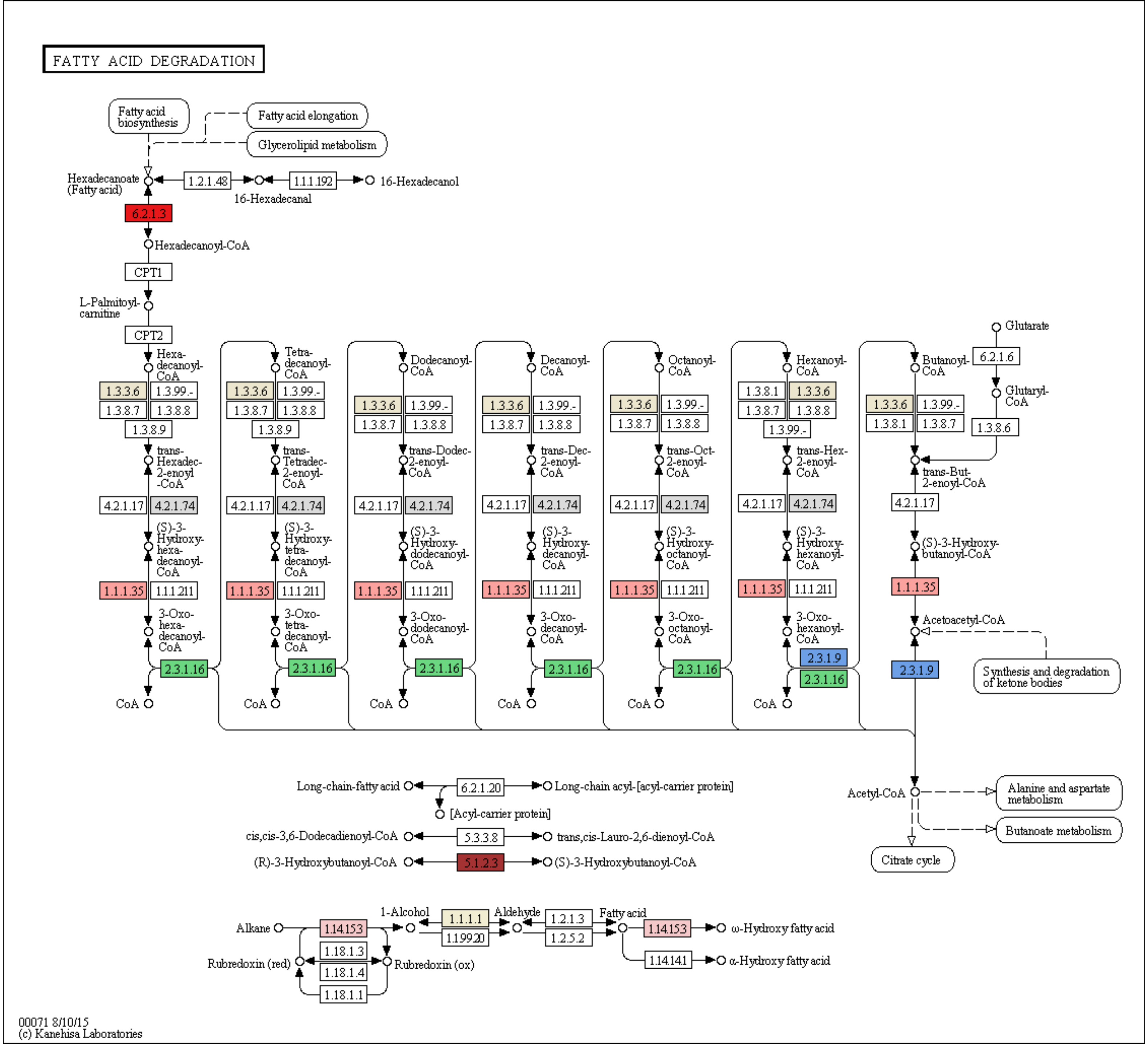
FATTY ACID DEGRADATION
Fatty acid biosynthesis
Fatty acid elongation
Glycerolipid metabolism
Hexadecanoate (Fatty acid)
1.2.1.48
1.1.1.192
16-Hexadecanol
16-Hexadecanal
6.2.1.3
Hexadecanoyl-CoA
CPT1
L-Palmitoyl-carnitine
CPT2
Hexa-decanoyl-CoA
Tetra-decanoyl-CoA
Dodecanoyl-CoA
Decanoyl-CoA
Octanoyl-CoA
Hexanoyl-CoA
Butanoyl-CoA
Glutarate
6.2.1.6
Glutaryl-CoA
1.3.8.6
1.3.3.6
1.3.99.-
1.3.8.7
1.3.8.8
1.3.8.9
1.3.8.1
trans-Hexadec-2-enoyl-CoA
trans-Tetradec-2-enoyl-CoA
trans-Dodec-2-enoyl-CoA
trans-Dec-2-enoyl-CoA
trans-Oct-2-enoyl-CoA
trans-Hex-2-enoyl-CoA
trans-But-2-enoyl-CoA
4.2.1.17
4.2.1.74
(S)-3-Hydroxy-hexa-decanoyl-CoA
(S)-3-Hydroxy-tetra-decanoyl-CoA
(S)-3-Hydroxy-dodecanoyl-CoA
(S)-3-Hydroxy-decanoyl-CoA
(S)-3-Hydroxy-octanoyl-CoA
(S)-3-Hydroxy-hexanoyl-CoA
(S)-3-Hydroxy-butanoyl-CoA
1.1.1.35
1.1.1.211
3-Oxo-hexa-decanoyl-CoA
3-Oxo-tetra-decanoyl-CoA
3-Oxo-dodecanoyl-CoA
3-Oxo-decanoyl-CoA
3-Oxo-octanoyl-CoA
3-Oxo-hexanoyl-CoA
Acetoacetyl-CoA
2.3.1.16
2.3.1.9
CoA
Synthesis and degradation of ketone bodies
Acetyl-CoA
Alanine and aspartate metabolism
Butanoate metabolism
Citrate cycle
Long-chain-fatty acid
6.2.1.20
Long-chain acyl-[acyl-carrier protein]
[Acyl-carrier protein]
cis,cis-3,6-Dodecadienoyl-CoA
5.3.3.8
trans,cis-Lauro-2,6-dienoyl-CoA
(R)-3-Hydroxybutanoyl-CoA
5.1.2.3
(S)-3-Hydroxybutanoyl-CoA
Alkane
1.14.15.3
1-Alcohol
1.1.1.1
1.1.99.20
Aldehyde
1.2.1.3
1.2.5.2
Fatty acid
ω-Hydroxy fatty acid
1.14.14.1
α-Hydroxy fatty acid
1.18.1.3
1.18.1.4
1.18.1.1
Rubredoxin (red)
Rubredoxin (ox)
00071 8/10/15
(c) Kanehisa Laboratories

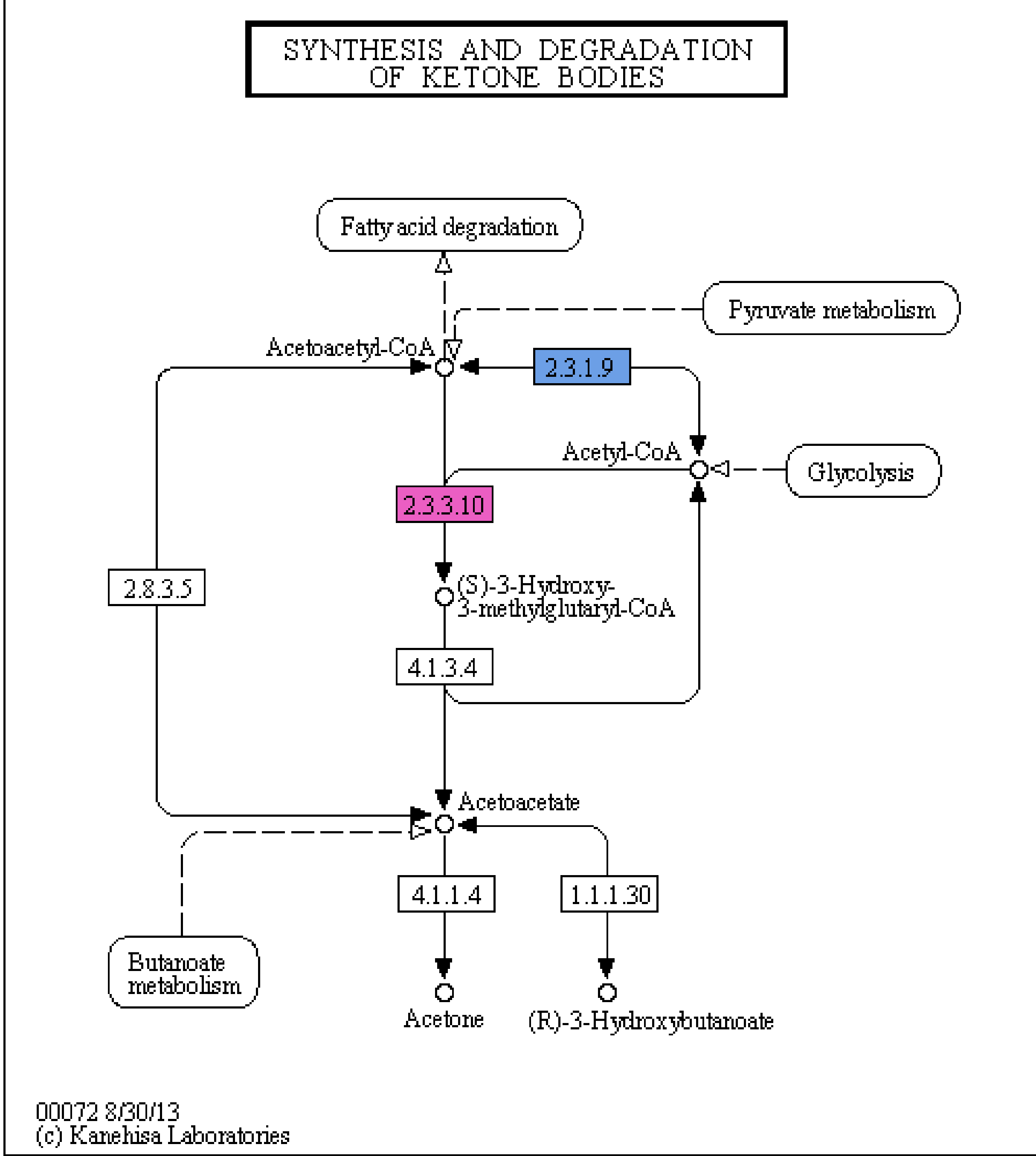
SYNTHESIS AND DEGRADATION OF KETONE BODIES
Fatty acid degradation
Pyruvate metabolism
Acetoacetyl-CoA
2.3.1.9
Acetyl-CoA
Glycolysis
2.3.3.10
2.8.3.5
(S)-3-Hydroxy-3-methylglutaryl-CoA
4.1.3.4
Acetoacetate
4.1.1.4
1.1.1.30
Butanoate metabolism
Acetone
(R)-3-Hydroxybutanoate
00072 8/30/13
(c) Kanehisa Laboratories

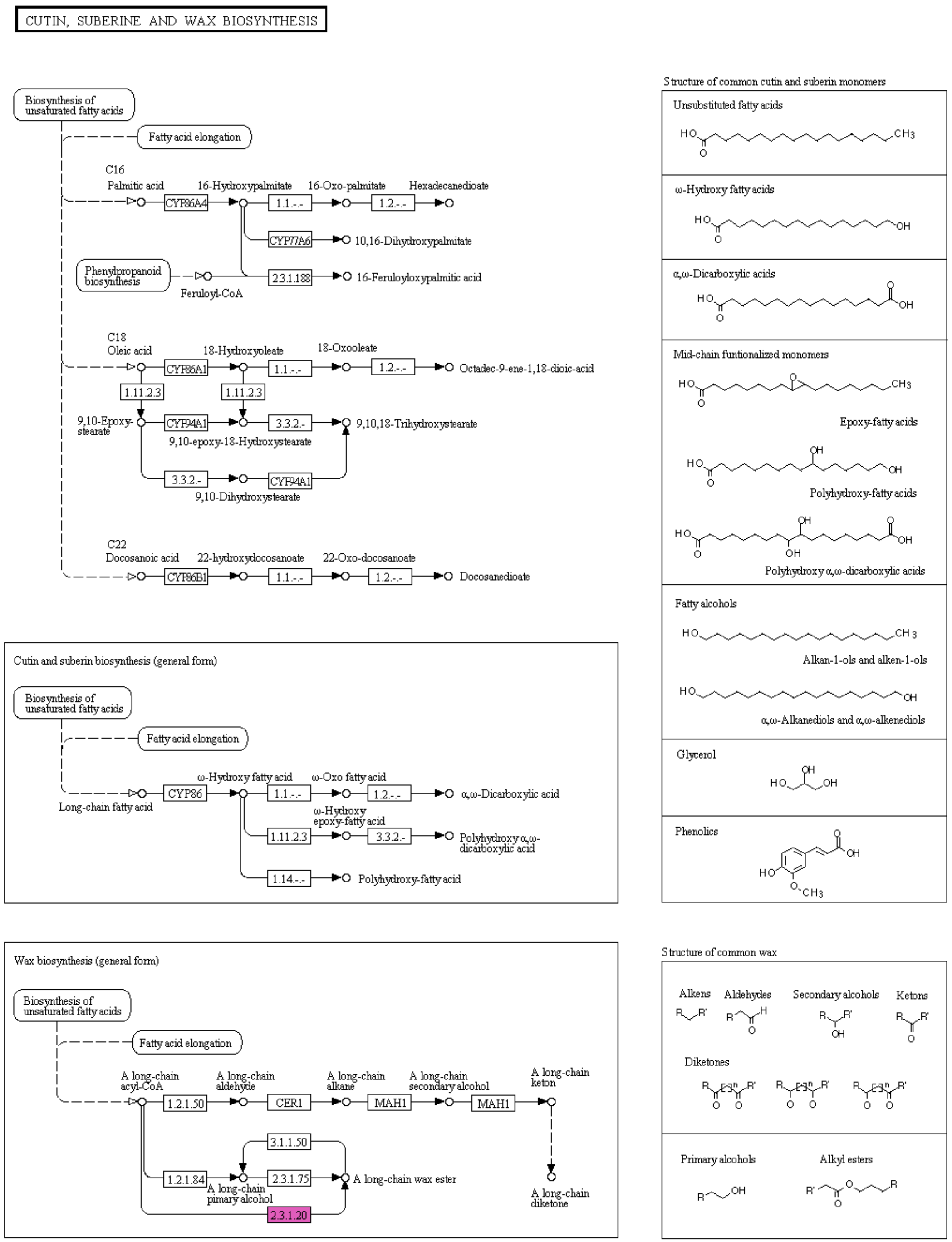

00073 11/20/15
(c) Kanehisa Laboratories

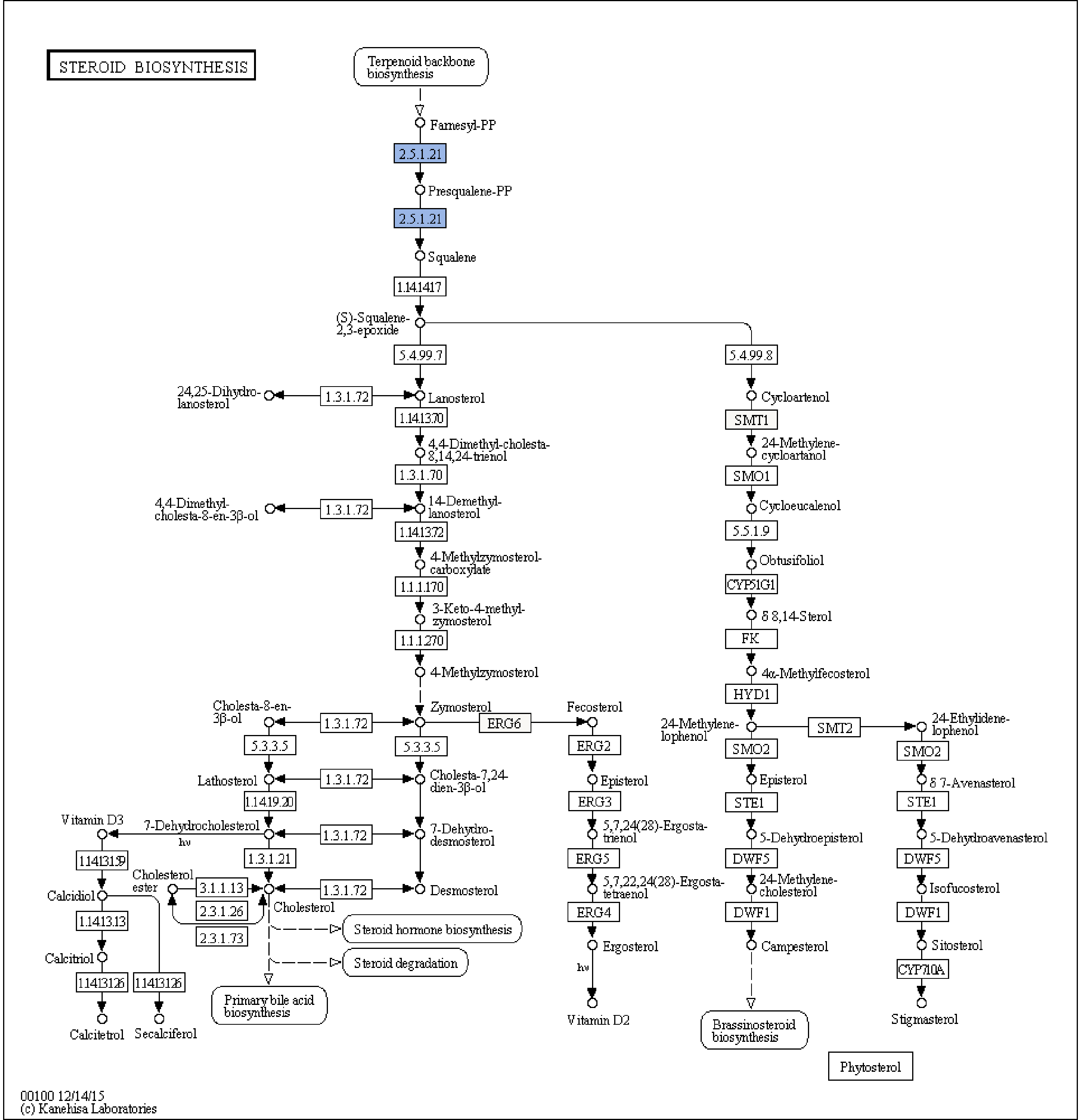
STEROID BIOSYNTHESIS
Terpenoid backbone biosynthesis
Farnesyl-PP
2.5.1.21
Presqualene-PP
2.5.1.21
Squalene
1.14.14.17
(S)-Squalene-2,3-epoxide
5.4.99.7
5.4.99.8
24,25-Dihydro-lanosterol
1.3.1.72
Lanosterol
1.14.13.70
4,4-Dimethyl-cholesta-8,14,24-trienol
1.3.1.70
4,4-Dimethyl-cholesta-8-en-3β-ol
1.3.1.72
14-Demethyl-lanosterol
1.14.13.72
4-Methylzymosterol-carboxylate
1.1.1.170
3-Keto-4-methyl-zymosterol
1.1.1.270
4-Methylzymosterol
Cholesta-8-en-3β-ol
1.3.1.72
Zymosterol
ERG6
Fecosterol
5.3.3.5
5.3.3.5
ERG2
Lathosterol
1.3.1.72
Cholesta-7,24-dien-3β-ol
Episterol
1.14.19.20
ERG3
Vitamin D3
7-Dehydrocholesterol
hν
1.3.1.72
7-Dehydro-desmosterol
5,7,24(28)-Ergosta-trienol
1.14.13.159
1.3.1.21
ERG5
Cholesterol ester
3.1.1.13
1.3.1.72
Desmosterol
5,7,22,24(28)-Ergosta-tetraenol
Calcidiol
2.3.1.26
Cholesterol
1.14.13.13
2.3.1.73
Steroid hormone biosynthesis
ERG4
Ergosterol
Calcitriol
Steroid degradation
hν
1.14.13.126
1.14.13.126
Primary bile acid biosynthesis
Vitamin D2
Calcitetrol
Secalciferol
Cycloartenol
SMT1
24-Methylene-cycloartanol
SMO1
Cycloeucalenol
5.5.1.9
Obtusifoliol
CYP51G1
δ 8,14-Sterol
FK
4α-Methylfecosterol
HYD1
24-Methylene-lophenol
SMT2
24-Ethylidene-lophenol
SMO2
SMO2
Episterol
δ 7-Avenasterol
STE1
STE1
5-Dehydroepisterol
5-Dehydroavenasterol
DWF5
DWF5
24-Methylene-cholesterol
Isofucosterol
DWF1
DWF1
Campesterol
Sitosterol
CYP710A
Brassinosteroid biosynthesis
Stigmasterol
Phytosterol
00100 12/14/15
(c) Kanehisa Laboratories

PRIMARY BILE ACID BIOSYNTHESIS

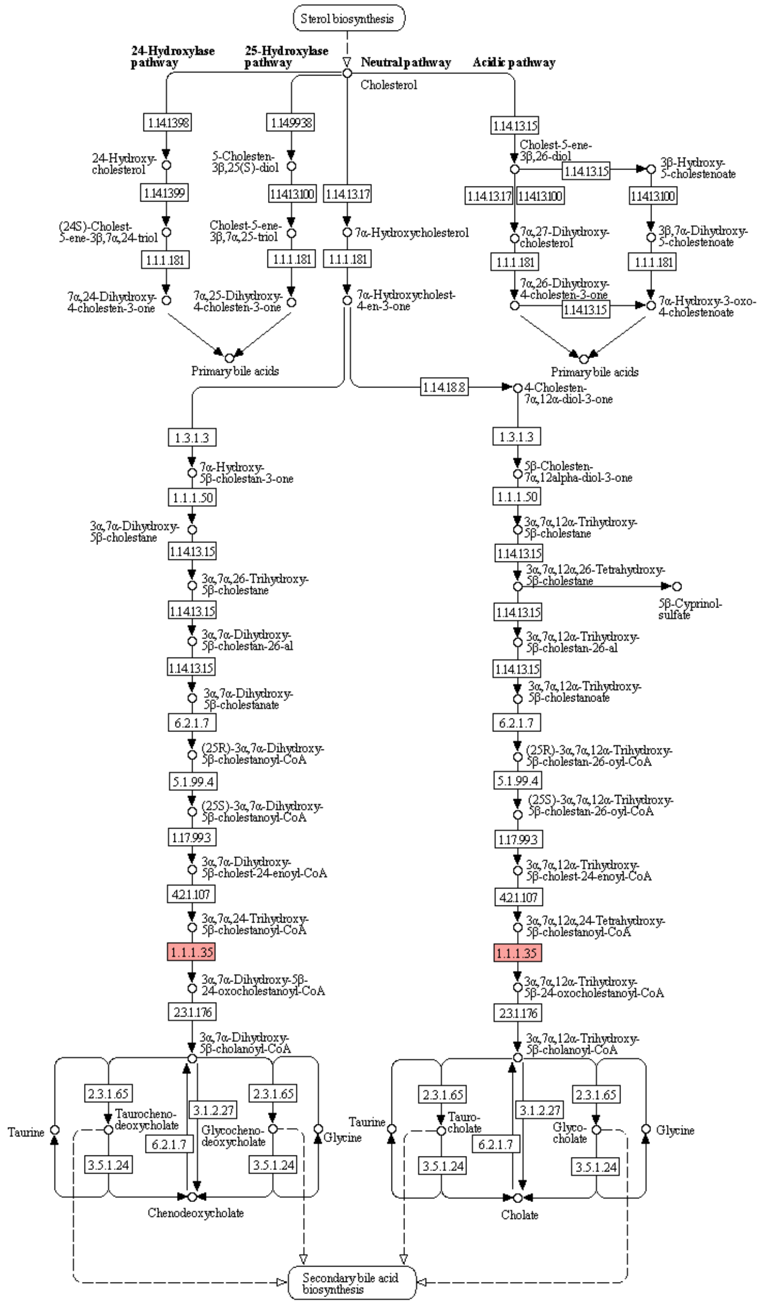

00120 12/14/15
(c) Kanehisa Laboratories

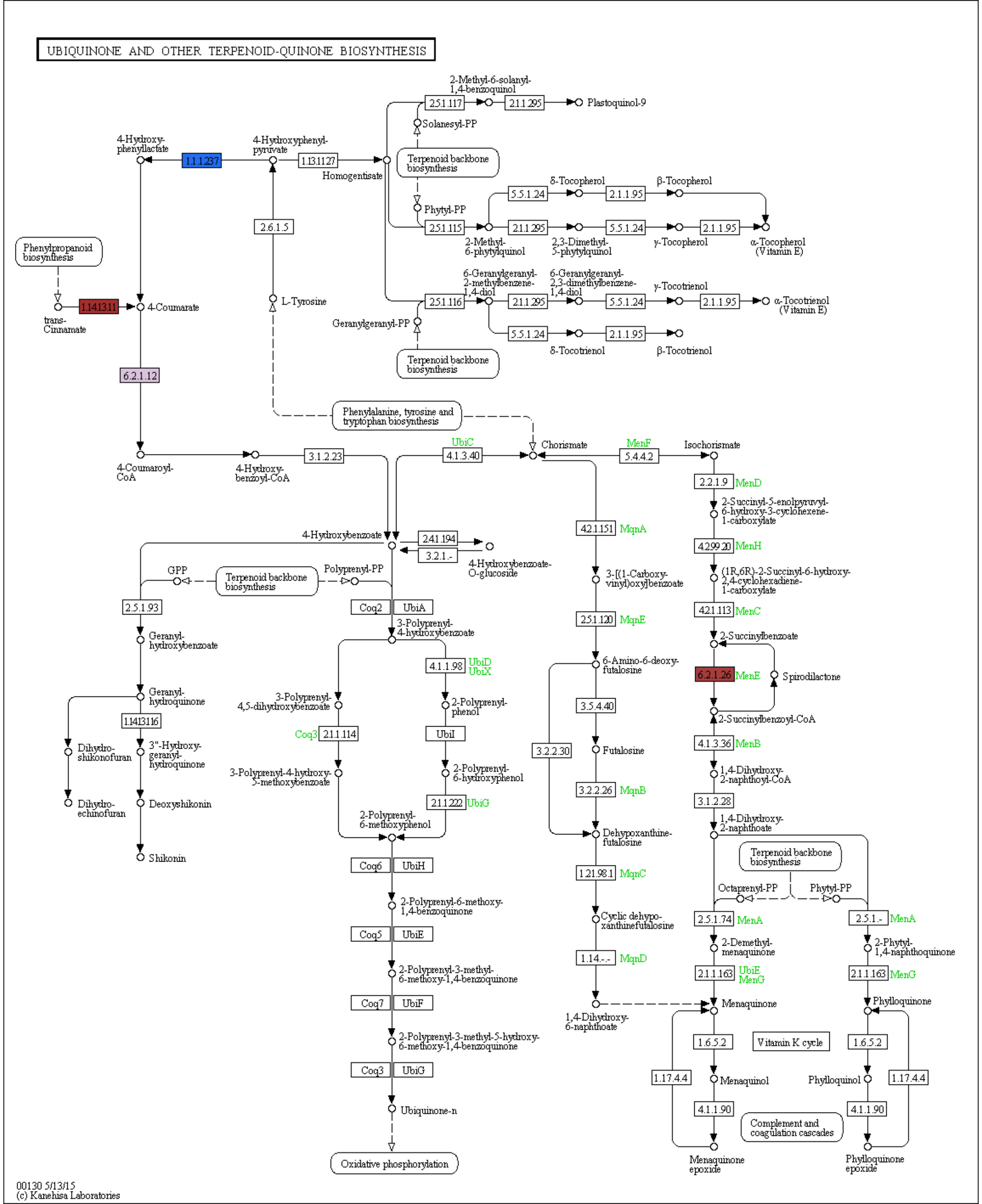
UBIQUINONE AND OTHER TERPENOID-QUINONE BIOSYNTHESIS
2-Methyl-6-solanyl-1,4-benzoquinol
2.5.1.117
2.1.1.295
Plastoquinol-9
Solanesyl-PP
4-Hydroxy-phenyllactate
1.1.1.237
4-Hydroxyphenyl-pyruvate
1.13.11.27
Homogentisate
Terpenoid backbone biosynthesis
δ-Tocopherol
β-Tocopherol
5.5.1.24
2.1.1.95
Phytyl-PP
2.5.1.115
2.1.1.295
5.5.1.24
2.1.1.95
2.6.1.5
2-Methyl-6-phytylquinol
2,3-Dimethyl-5-phytylquinol
γ-Tocopherol
α-Tocopherol (Vitamin E)
Phenylpropanoid biosynthesis
6-Geranylgeranyl-2-methylbenzene-1,4-diol
6-Geranylgeranyl-2,3-dimethylbenzene-1,4-diol
γ-Tocotrienol
2.5.1.116
2.1.1.295
5.5.1.24
2.1.1.95
α-Tocotrienol (Vitamin E)
1.14.13.11
4-Coumarate
L-Tyrosine
Geranylgeranyl-PP
trans-Cinnamate
5.5.1.24
2.1.1.95
δ-Tocotrienol
β-Tocotrienol
Terpenoid backbone biosynthesis
6.2.1.12
Phenylalanine, tyrosine and tryptophan biosynthesis
UbiC
MenF
4.1.3.40
Chorismate
5.4.4.2
Isochorismate
4-Coumaroyl-CoA
4-Hydroxy-benzoyl-CoA
3.1.2.23
2.2.1.9
MenD
2-Succinyl-5-enolpyruvyl-6-hydroxy-3-cyclohexene-1-carboxylate
4-Hydroxybenzoate
2.4.1.194
3.2.1.-
4.2.1.151
MqnA
4.2.99.20
MenH
4-Hydroxybenzoate-O-glucoside
GPP
Terpenoid backbone biosynthesis
Polyprenyl-PP
3-[(1-Carboxy-vinyl)oxy]benzoate
(1R,6R)-2-Succinyl-6-hydroxy-2,4-cyclohexadiene-1-carboxylate
2.5.1.93
Coq2
UbiA
2.5.1.120
MqnE
4.2.1.113
MenC
3-Polyprenyl-4-hydroxybenzoate
Geranyl-hydroxybenzoate
2-Succinylbenzoate
6-Amino-6-deoxy-futalosine
4.1.1.98
UbiD
UbiX
6.2.1.26
MenE
Spirodilactone
Geranyl-hydroquinone
3-Polyprenyl-4,5-dihydroxybenzoate
2-Polyprenyl-phenol
3.5.4.40
1.14.13.116
2-Succinylbenzoyl-CoA
Coq3
2.1.1.114
UbiI
3.2.2.30
Futalosine
4.1.3.36
MenB
Dihydro-shikonofuran
3''-Hydroxy-geranyl-hydroquinone
3-Polyprenyl-4-hydroxy-5-methoxybenzoate
2-Polyprenyl-6-hydroxyphenol
1,4-Dihydroxy-2-naphthoyl-CoA
3.2.2.26
MqnB
Dihydro-echinofuran
Deoxyshikonin
2.1.1.222
UbiG
3.1.2.28
2-Polyprenyl-6-methoxyphenol
1,4-Dihydroxy-2-naphthoate
Dehypoxanthine-futalosine
Shikonin
Terpenoid backbone biosynthesis
Coq6
UbiH
1.21.98.1
MqnC
Octaprenyl-PP
Phytyl-PP
2-Polyprenyl-6-methoxy-1,4-benzoquinone
Cyclic dehypo-xanthinefutalosine
2.5.1.74
MenA
2.5.1.-
MenA
Coq5
UbiE
2-Demethyl-menaquinone
2-Phytyl-1,4-naphthoquinone
2-Polyprenyl-3-methyl-6-methoxy-1,4-benzoquinone
1.14.-.-
MqnD
2.1.1.163
UbiE
MenG
2.1.1.163
MenG
Coq7
UbiF
Menaquinone
Phylloquinone
1,4-Dihydroxy-6-naphthoate
2-Polyprenyl-3-methyl-5-hydroxy-6-methoxy-1,4-benzoquinone
1.6.5.2
Vitamin K cycle
1.6.5.2
Coq3
UbiG
1.17.4.4
Menaquinol
Phylloquinol
1.17.4.4
4.1.1.90
4.1.1.90
Ubiquinone-n
Complement and coagulation cascades
Menaquinone epoxide
Phylloquinone epoxide
Oxidative phosphorylation
00130 5/13/15
(c) Kanehisa Laboratories

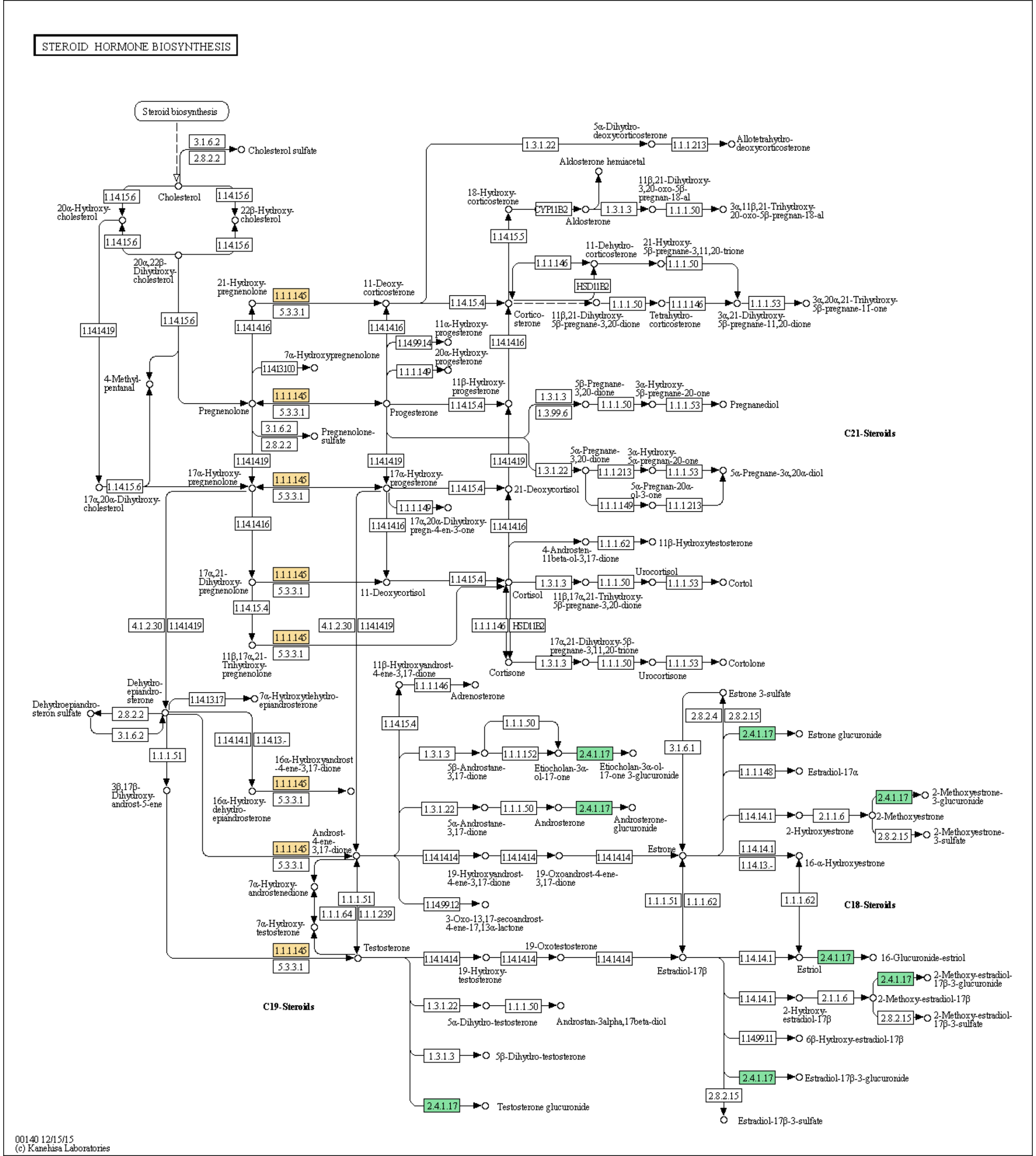
STEROID HORMONE BIOSYNTHESIS
Steroid biosynthesis
3.1.6.2
2.8.2.2
Cholesterol sulfate
1.14.15.6
Cholesterol
1.14.15.6
20α-Hydroxy-cholesterol
22β-Hydroxy-cholesterol
1.14.15.6
1.14.15.6
20α,22β-Dihydroxy-cholesterol
1.14.14.19
1.14.15.6
4-Methyl-pentanal
21-Hydroxy-pregnenolone
1.1.1.145
5.3.3.1
11-Deoxy-corticosterone
1.14.14.16
1.14.14.16
7α-Hydroxypregnenolone
1.14.13.100
11α-Hydroxy-progesterone
1.14.99.14
20α-Hydroxy-progesterone
1.1.1.149
11β-Hydroxy-progesterone
1.14.15.4
Pregnenolone
1.1.1.145
5.3.3.1
Progesterone
3.1.6.2
2.8.2.2
Pregnenolone-sulfate
1.14.14.19
1.14.14.19
17α-Hydroxy-pregnenolone
1.1.1.145
5.3.3.1
17α-Hydroxy-progesterone
1.14.15.4
1.14.15.6
17α,20α-Dihydroxy-cholesterol
1.1.1.149
17α,20α-Dihydroxy-pregn-4-en-3-one
1.14.14.16
1.14.14.16
17α,21-Dihydroxy-pregnenolone
1.1.1.145
5.3.3.1
11-Deoxycortisol
1.14.15.4
1.14.15.4
1.1.1.145
5.3.3.1
11β,17α,21-Trihydroxy-pregnenolone
4.1.2.30
1.14.14.19
4.1.2.30
1.14.14.19
1.3.1.22
5α-Dihydro-deoxycorticosterone
1.1.1.213
Allotetrahydro-deoxycorticosterone
Aldosterone hemiacetal
18-Hydroxy-corticosterone
CYP11B2
Aldosterone
11β,21-Dihydroxy-3,20-oxo-5β-pregnan-18-al
1.3.1.3
1.1.1.50
3α,11β,21-Trihydroxy-20-oxo-5β-pregnan-18-al
1.14.15.5
1.1.1.146
11-Dehydro-corticosterone
21-Hydroxy-5β-pregnane-3,11,20-trione
1.1.1.50
HSD11B2
1.14.15.4
Cortico-sterone
1.1.1.50
11β,21-Dihydroxy-5β-pregnane-3,20-dione
1.1.1.146
Tetrahydro-corticosterone
3α,21-Dihydroxy-5β-pregnane-11,20-dione
1.1.1.53
3α,20α,21-Trihydroxy-5β-pregnane-11-one
1.14.14.16
1.3.1.3
1.3.99.6
5β-Pregnane-3,20-dione
1.1.1.50
3α-Hydroxy-5β-pregnane-20-one
1.1.1.53
Pregnanediol
C21-Steroids
1.14.14.19
1.3.1.22
5α-Pregnane-3,20-dione
1.1.1.213
3α-Hydroxy-5α-pregnan-20-one
1.1.1.53
5α-Pregnane-3α,20α-diol
21-Deoxycortisol
5α-Pregnan-20α-ol-3-one
1.1.1.149
1.1.1.213
1.14.14.16
4-Androsten-11beta-ol-3,17-dione
1.1.1.62
11β-Hydroxytestosterone
Urocortisol
1.3.1.3
1.1.1.50
1.1.1.53
Cortol
Cortisol
11β,17α,21-Trihydroxy-5β-pregnane-3,20-dione
1.1.1.146
HSD11B2
17α,21-Dihydroxy-5β-pregnane-3,11,20-trione
Cortisone
1.3.1.3
1.1.1.50
1.1.1.53
Cortolone
Urocortisone
11β-Hydroxyandrost-4-ene-3,17-dione
1.1.1.146
Adrenosterone
Dehydro-epiandro-sterone
Dehydroepiandro-steron sulfate
2.8.2.2
3.1.6.2
1.14.13.17
7α-Hydroxydehydro-epiandrosterone
1.1.1.51
1.14.14.1
1.14.13.-
16α-Hydroxyandrost-4-ene-3,17-dione
1.1.1.145
5.3.3.1
16α-Hydroxy-dehydro-epiandrosterone
3β,17β-Dihydroxy-androst-5-ene
1.14.15.4
1.1.1.50
1.3.1.3
1.1.1.152
2.4.1.17
5β-Androstane-3,17-dione
Etiocholan-3α-ol-17-one
Etiocholan-3α-ol-17-one 3-glucuronide
1.3.1.22
1.1.1.50
2.4.1.17
5α-Androstane-3,17-dione
Androsterone
Androsterone-glucuronide
Androst-4-ene-3,17-dione
1.1.1.145
5.3.3.1
1.14.14.14
1.14.14.14
1.14.14.14
19-Hydroxyandrost-4-ene-3,17-dione
19-Oxoandrost-4-ene-3,17-dione
7α-Hydroxy-androstenedione
1.1.1.51
1.1.1.64
1.1.1.239
1.14.99.12
3-Oxo-13,17-secoandrost-4-ene-17,13α-lactone
7α-Hydroxy-testosterone
1.1.1.145
5.3.3.1
Testosterone
1.14.14.14
19-Hydroxy-testosterone
1.14.14.14
19-Oxotestosterone
1.14.14.14
C19-Steroids
1.3.1.22
1.1.1.50
5α-Dihydro-testosterone
Androstan-3alpha,17beta-diol
1.3.1.3
5β-Dihydro-testosterone
2.4.1.17
Testosterone glucuronide
Estrone 3-sulfate
2.8.2.4
2.8.2.15
3.1.6.1
2.4.1.17
Estrone glucuronide
1.1.1.148
Estradiol-17α
2.4.1.17
2-Methoxyestrone-3-glucuronide
1.14.14.1
2.1.1.6
2-Methoxyestrone
2-Hydroxyestrone
2.8.2.15
2-Methoxyestrone-3-sulfate
Estrone
1.14.14.1
1.14.13.-
16-α-Hydroxyestrone
1.1.1.51
1.1.1.62
1.1.1.62
C18-Steroids
Estradiol-17β
1.14.14.1
Estriol
2.4.1.17
16-Glucuronide-estriol
2.4.1.17
2-Methoxy-estradiol-17β-3-glucuronide
1.14.14.1
2.1.1.6
2-Methoxy-estradiol-17β
2-Hydroxy-estradiol-17β
2.8.2.15
2-Methoxy-estradiol-17β-3-sulfate
1.14.99.11
6β-Hydroxy-estradiol-17β
2.4.1.17
Estradiol-17β-3-glucuronide
2.8.2.15
Estradiol-17β-3-sulfate
00140 12/15/15
(c) Kanehisa Laboratories

OXIDATIVE PHOSPHORYLATION

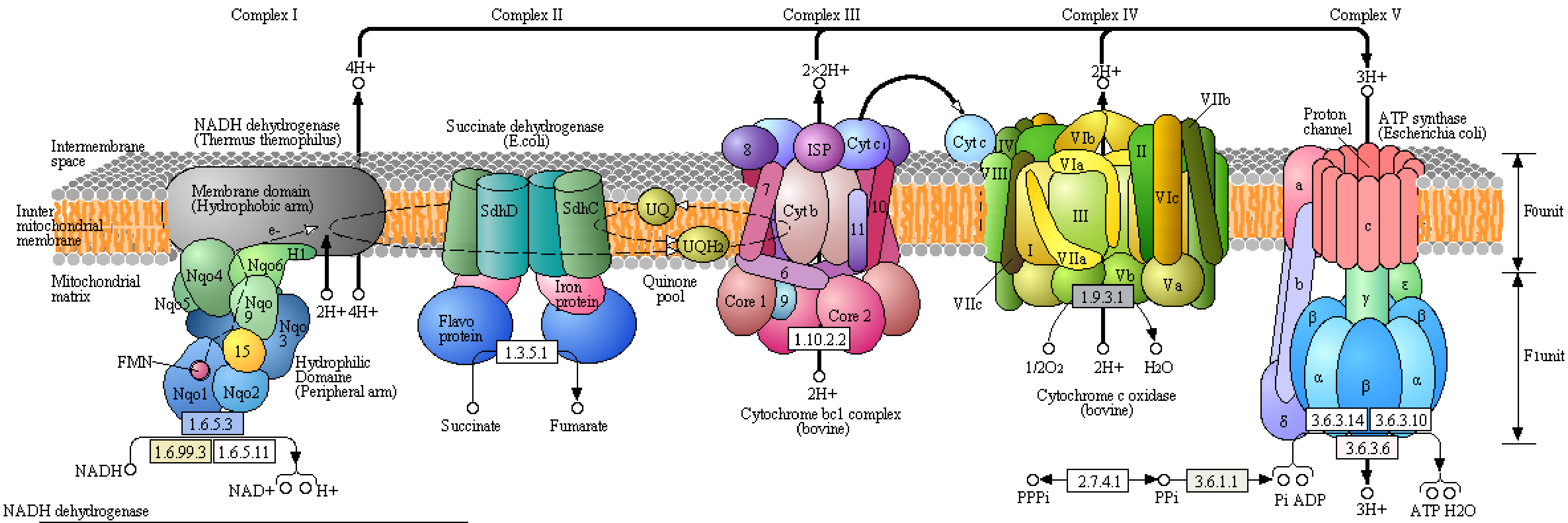

NADH dehydrogenase

E	ND1	ND2	ND3	ND4	ND4L	ND5	ND6										
E	Ndufs1	Ndufs2	Ndufs3	Ndufs4	Ndufs5	Ndufs6	Ndufs7	Ndufs8	Ndufv1	Ndufv2	Ndufv3						
B/A	NuoA	NuoB	NuoC	NuoD	NuoE	NuoF	NuoG	NuoH	NuoI	NuoJ	NuoK	NuoL	NuoM	NuoN			
B/A	NdhC	NdhK	NdhJ	NdhH	NdhA	NdhI	NdhG	NdhE	NdhF	NdhD	NdhB	NdhL	NdhM	NdhN	HoxE	HoxF	HoxU
E	Ndufa1	Ndufa2	Ndufa3	Ndufa4	Ndufa5	Ndufa6	Ndufa7	Ndufa8	Ndufa9	Ndufa10	Ndufab1	Ndufa11	Ndufa12	Ndufa13			
E	Ndufb1	Ndufb2	Ndufb3	Ndufb4	Ndufb5	Ndufb6	Ndufb7	Ndufb8	Ndufb9	Ndufb10	Ndufb11	Ndufc1	Ndufc2				

Succinate dehydrogenase / Fumarate reductase

E	SDHC	SDHD	SDHA	SDHB
B/A	SdhC	SdhD	SdhA	SdhB
		FrdA	FrdB	FrdC	FrdD

Cytochrome c reductase

E/B/A	ISP	Cyt b	Cyt 1						
E				COR1	QCR2	QCR6	QCR7	QCR8	QCR9	QCR10

Cytochrome c oxidase

E	COX10	COX3	COX1	COX2	COX4	COX5A	COX5B	COX6A	COX6B	COX6C	COX7A	COX7B	COX7C	COX8	E/B/A: COX11, COX15	COX17
B/A	CyoE	CyoD	CyoC	CyoB	CyoA											
		CoxD	CoxC	CoxA	CoxB											
		QoxD	QoxC	QoxB	QoxA											

Cytochrome c oxidase , cbb3-type

B	I	II	IV	III

Cytochrome bd complex

B/A	CydA	CydB

F-type ATPase (Bacteria)

alpha	beta	gamma	delta	epsilon
a	b	c		

F-type ATPase (Eukaryotes)

alpha	beta	gamma	delta	epsilon	
OSCP	a	b	c	d	e
f	g	f6/h	j	k	8

V/A-type ATPase (Bacteria, Archaeas)

A	B	C	D	E	F	G/H
I	K					

V-type ATPase (Eukaryotes)

A	B	C	D	E	F	G	H
a	c	d	e	S1			

00190 6/16/15
(c) Kanehisa Laboratories

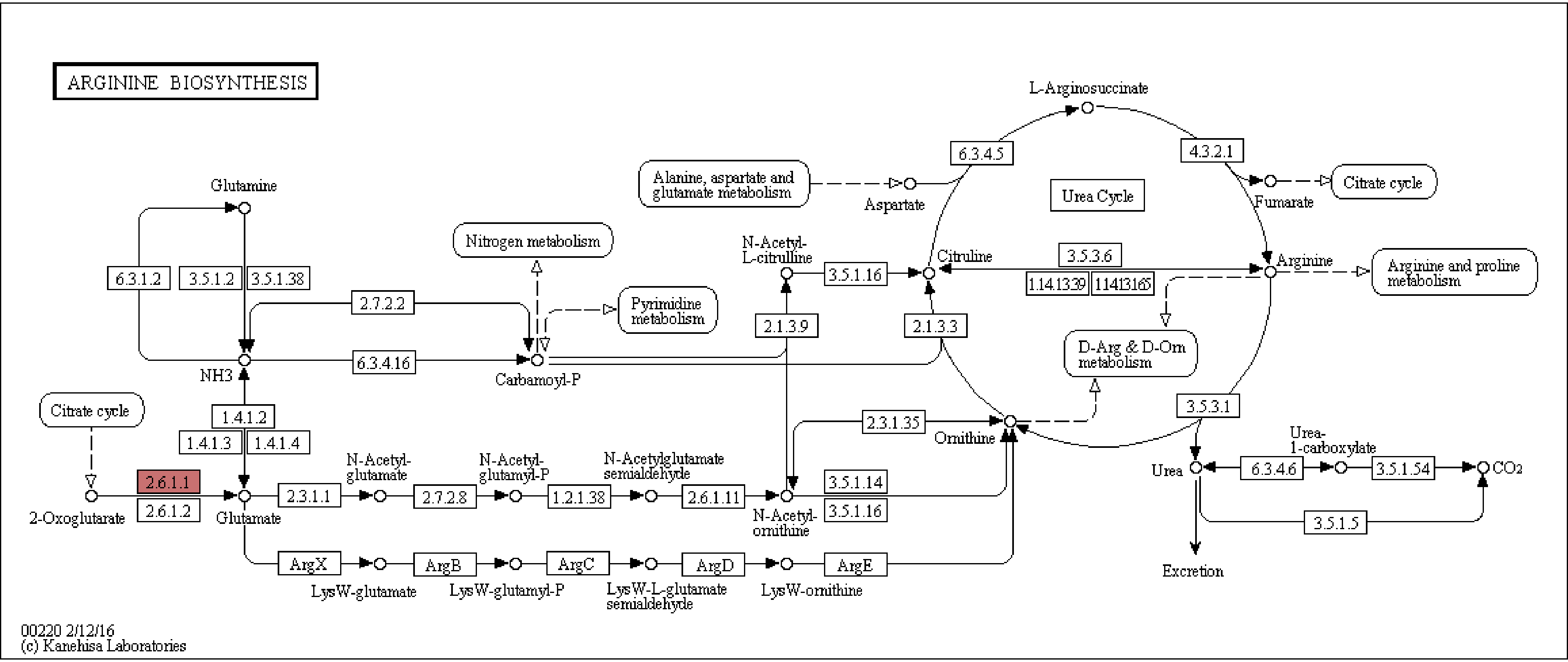
ARGININE BIOSYNTHESIS
L-Arginosuccinate
6.3.4.5
4.3.2.1
Alanine, aspartate and glutamate metabolism
Aspartate
Urea Cycle
Citrate cycle
Fumarate
Glutamine
Nitrogen metabolism
N-Acetyl-L-citrulline
Citruline
3.5.3.6
Arginine
Arginine and proline metabolism
6.3.1.2
3.5.1.2
3.5.1.38
3.5.1.16
1.14.13.39
1.14.13.165
2.7.2.2
Pyrimidine metabolism
2.1.3.9
2.1.3.3
D-Arg & D-Orn metabolism
NH3
6.3.4.16
Carbamoyl-P
Citrate cycle
1.4.1.2
1.4.1.3
1.4.1.4
2.3.1.35
Ornithine
3.5.3.1
Urea-1-carboxylate
N-Acetyl-glutamate
N-Acetyl-glutamyl-P
N-Acetylglutamate semialdehyde
2.6.1.1
2.3.1.1
2.7.2.8
1.2.1.38
2.6.1.11
3.5.1.14
Urea
6.3.4.6
3.5.1.54
CO_2
2-Oxoglutarate
2.6.1.2
Glutamate
N-Acetyl-ornithine
3.5.1.16
3.5.1.5
ArgX
ArgB
ArgC
ArgD
ArgE
Excretion
LysW-glutamate
LysW-glutamyl-P
LysW-L-glutamate semialdehyde
LysW-ornithine
00220 2/12/16
(c) Kanehisa Laboratories

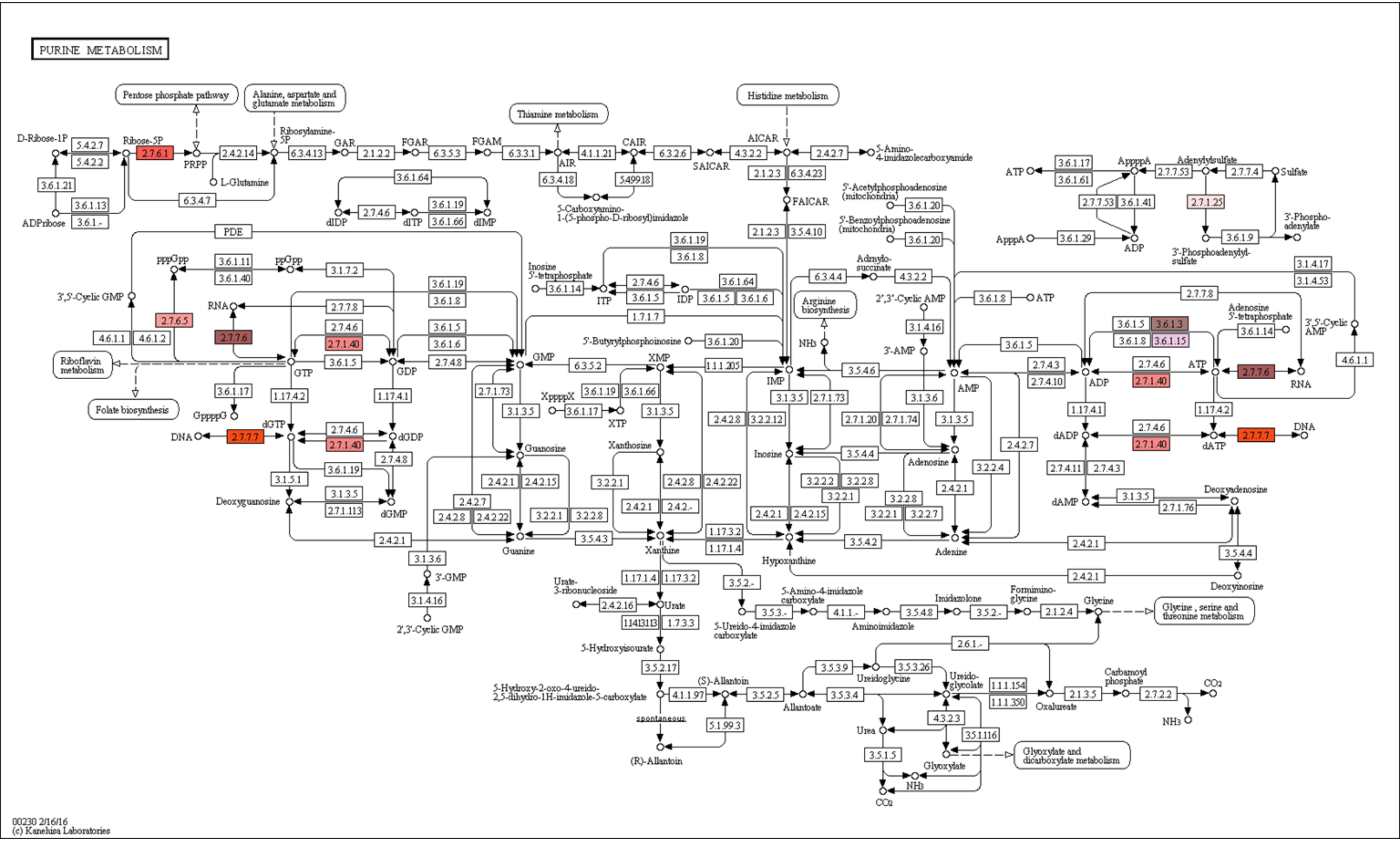
PURINE METABOLISM
Pentose phosphate pathway
Alanine, aspartate and glutamate metabolism
Thiamine metabolism
Histidine metabolism
D-Ribose-1P
Ribose-5P
PRPP
Ribosylamine-5P
GAR
FGAR
FGAM
AIR
CAIR
SAICAR
AICAR
FAICAR
5-Amino-4-imidazolecarboxyamide
L-Glutamine
ADPribose
5-Carboxyamino-1-(5-phospho-D-ribosyl)imidazole
dIDP
dITP
dIMP
PDE
pppGpp
ppGpp
3',5'-Cyclic GMP
RNA
GTP
GDP
GMP
Riboflavin metabolism
Folate biosynthesis
GppppG
dGTP
dGDP
dGMP
DNA
Deoxyguanosine
Guanosine
Guanine
3'-GMP
2',3'-Cyclic GMP
Inosine 5'-tetraphosphate
ITP
IDP
5'-Butyrylphosphoinosine
XMP
XppppX
XTP
Xanthosine
Xanthine
IMP
Inosine
Hypoxanthine
Urate-3-ribonucleoside
Urate
5-Hydroxyisourate
5-Hydroxy-2-oxo-4-ureido-2,5-dihydro-1H-imidazole-5-carboxylate
(S)-Allantoin
(R)-Allantoin
spontaneous
Allantoate
5-Ureido-4-imidazole carboxylate
5-Amino-4-imidazole carboxylate
Aminoimidazole
Imidazolone
Formimino-glycine
Glycine
Glycine, serine and threonine metabolism
Ureidoglycine
Ureido-glycolate
Urea
Glyoxylate
Glyoxylate and dicarboxylate metabolism
NH3
CO2
Oxalureate
Carbamoyl phosphate
Arginine biosynthesis
Adenylo-succinate
5'-Acetylphosphoadenosine (mitochondria)
5'-Benzoylphosphoadenosine (mitochondria)
2',3'-Cyclic AMP
3'-AMP
AMP
Adenosine
Adenine
ADP
ATP
dADP
dATP
dAMP
Deoxyadenosine
Deoxyinosine
ApppA
ApppA
Adenylylsulfate
Sulfate
3'-Phospho-adenylate
3'-Phosphoadenylyl-sulfate
Adenosine 5'-tetraphosphate
3',5'-Cyclic AMP
5.4.2.7
5.4.2.2
3.6.1.21
3.6.1.13
3.6.1.-
2.7.6.1
2.4.2.14
6.3.4.7
6.3.4.13
2.1.2.2
6.3.5.3
6.3.3.1
4.1.1.21
6.3.4.18
5.4.99.18
6.3.2.6
4.3.2.2
2.4.2.7
2.1.2.3
6.3.4.23
3.5.4.10
3.6.1.64
2.7.4.6
3.6.1.19
3.6.1.66
3.6.1.11
3.6.1.40
3.1.7.2
2.7.6.5
2.7.7.6
4.6.1.1
4.6.1.2
2.7.7.8
2.7.1.40
3.6.1.8
3.6.1.5
3.6.1.6
2.7.4.8
6.3.5.2
3.6.1.17
1.17.4.2
1.17.4.1
2.7.7.7
3.1.5.1
3.1.3.5
2.7.1.113
2.4.2.1
2.7.1.73
2.4.2.15
2.4.2.8
2.4.2.22
3.2.2.1
3.2.2.8
3.5.4.3
3.1.3.6
3.1.4.16
3.6.1.14
1.7.1.7
3.6.1.20
1.1.1.205
2.4.2.-
1.17.3.2
1.17.1.4
1.7.3.3
3.5.2.17
4.1.1.97
3.5.2.5
5.1.99.3
3.5.2.-
3.5.3.-
4.1.1.-
3.5.4.8
2.1.2.4
2.6.1.-
3.5.3.9
3.5.3.26
3.5.3.4
4.3.2.3
3.5.1.116
3.5.1.5
1.1.1.154
1.1.1.350
2.1.3.5
2.7.2.2
3.2.2.12
3.5.4.6
6.3.4.4
3.1.3.6
2.7.1.20
2.7.1.74
3.5.4.4
3.2.2.2
3.2.2.7
2.4.2.7
3.2.2.4
2.7.4.3
2.7.4.10
2.7.4.11
2.7.1.76
3.6.1.17
3.6.1.61
2.7.7.53
3.6.1.41
2.7.7.4
2.7.1.25
3.6.1.9
3.6.1.29
3.1.4.17
3.1.4.53
3.6.1.3
3.6.1.15
3.5.4.4
00230 2/16/16
(c) Kanehisa Laboratories

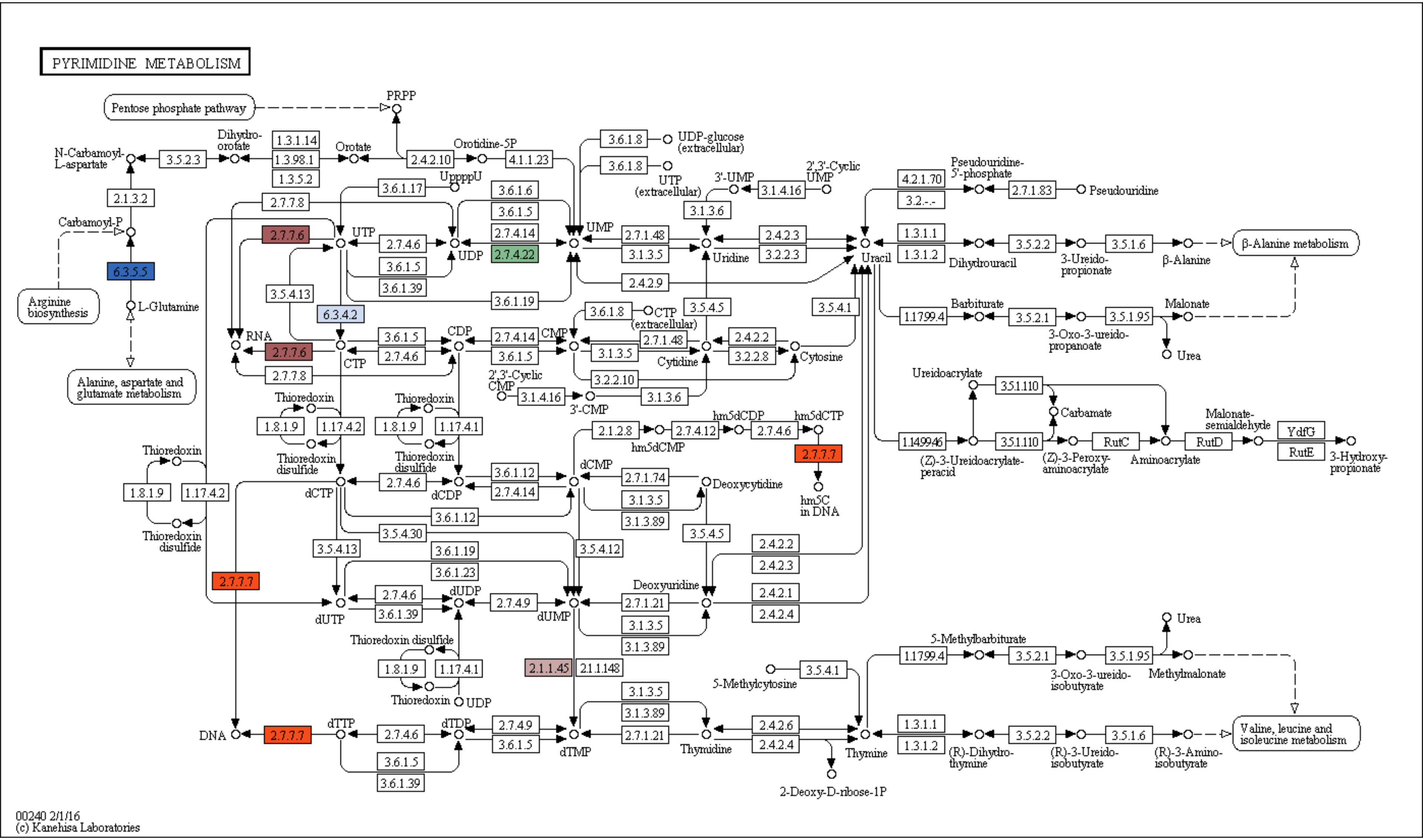

PYRIMIDINE METABOLISM
Pentose phosphate pathway
PRPP
N-Carbamoyl-L-aspartate
3.5.2.3
Dihydro-orotate
1.3.1.14
1.3.98.1
1.3.5.2
Orotate
2.4.2.10
Orotidine-5P
4.1.1.23
2.1.3.2
Carbamoyl-P
6.3.5.5
Arginine biosynthesis
L-Glutamine
Alanine, aspartate and glutamate metabolism
3.6.1.8
UDP-glucose (extracellular)
3.6.1.8
UTP (extracellular)
3'-UMP
3.1.4.16
2',3'-Cyclic UMP
3.1.3.6
UppppU
3.6.1.17
2.7.7.8
3.6.1.6
3.6.1.5
2.7.4.14
2.7.4.22
2.7.7.6
UTP
2.7.4.6
UDP
UMP
3.6.1.5
3.6.1.39
3.6.1.19
2.7.1.48
3.1.3.5
2.4.2.3
3.2.2.3
Uridine
2.4.2.9
Uracil
Pseudouridine-5'-phosphate
4.2.1.70
3.2.-.-
2.7.1.83
Pseudouridine
1.3.1.1
1.3.1.2
Dihydrouracil
3.5.2.2
3-Ureido-propionate
3.5.1.6
β-Alanine
β-Alanine metabolism
3.5.4.13
6.3.4.2
RNA
2.7.7.6
2.7.7.8
3.6.1.5
2.7.4.6
CTP
CDP
2.7.4.14
3.6.1.5
CMP
3.6.1.8
CTP (extracellular)
3.5.4.5
2.7.1.48
3.1.3.5
2.4.2.2
3.2.2.8
Cytidine
Cytosine
3.5.4.1
3.2.2.10
2',3'-Cyclic CMP
3.1.4.16
3'-CMP
3.1.3.6
Barbiturate
1.17.99.4
3.5.2.1
3-Oxo-3-ureido-propanoate
3.5.1.95
Malonate
Urea
Ureidoacrylate
3.5.1.110
Carbamate
1.14.99.46
3.5.1.110
(Z)-3-Ureidoacrylate-peracid
(Z)-3-Peroxy-aminoacrylate
RutC
Aminoacrylate
Malonate-semialdehyde
RutD
YdfG
RutE
3-Hydroxy-propionate
Thioredoxin
1.8.1.9
1.17.4.2
Thioredoxin disulfide
Thioredoxin
1.8.1.9
1.17.4.1
Thioredoxin disulfide
Thioredoxin
1.8.1.9
1.17.4.2
Thioredoxin disulfide
hm5dCDP
2.1.2.8
2.7.4.12
2.7.4.6
hm5dCTP
hm5dCMP
2.7.7.7
hm5C in DNA
dCTP
2.7.4.6
dCDP
3.6.1.12
2.7.4.14
dCMP
2.7.1.74
3.1.3.5
3.1.3.89
Deoxycytidine
3.6.1.12
3.5.4.30
3.5.4.13
3.6.1.19
3.6.1.23
3.5.4.12
3.5.4.5
2.4.2.2
2.4.2.3
2.7.7.7
dUTP
2.7.4.6
3.6.1.39
dUDP
2.7.4.9
dUMP
2.7.1.21
3.1.3.5
3.1.3.89
Deoxyuridine
2.4.2.1
2.4.2.4
Thioredoxin disulfide
1.8.1.9
1.17.4.1
Thioredoxin
UDP
2.1.1.45
2.1.1.148
5-Methylbarbiturate
1.17.99.4
3.5.2.1
3-Oxo-3-ureido-isobutyrate
3.5.1.95
Methylmalonate
Urea
5-Methylcytosine
3.5.4.1
3.1.3.5
3.1.3.89
DNA
2.7.7.7
dTTP
2.7.4.6
dTDP
2.7.4.9
3.6.1.5
dTMP
2.7.1.21
Thymidine
2.4.2.6
2.4.2.4
Thymine
1.3.1.1
1.3.1.2
(R)-Dihydro-thymine
3.5.2.2
(R)-3-Ureido-isobutyrate
3.5.1.6
(R)-3-Amino-isobutyrate
Valine, leucine and isoleucine metabolism
3.6.1.5
3.6.1.39
2-Deoxy-D-ribose-1P
00240 2/1/16
(c) Kanehisa Laboratories

ALANINE, ASPARTATE AND GLUTAMATE METABOLISM

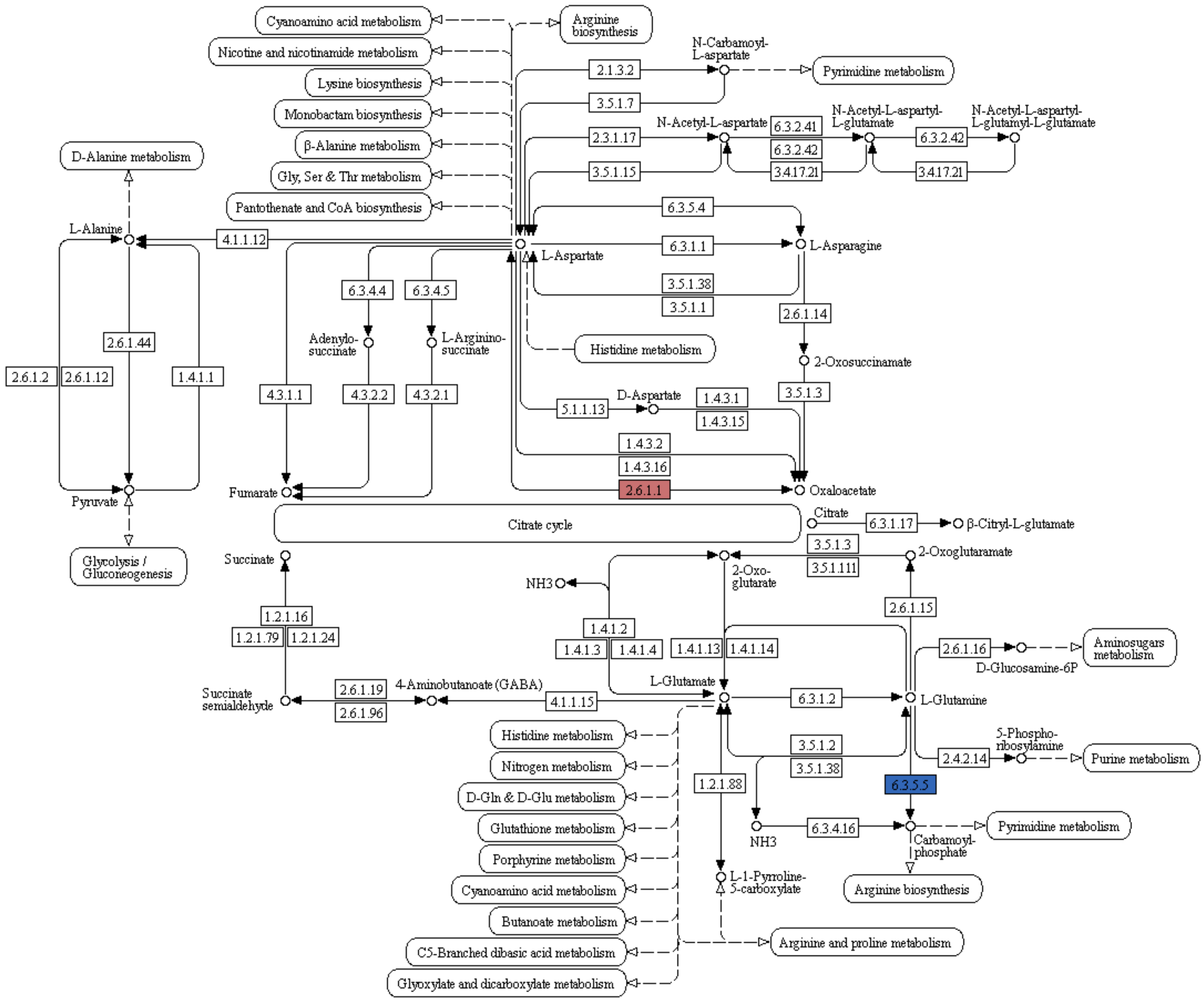

00250 10/30/15

(c) Kanehisa Laboratories

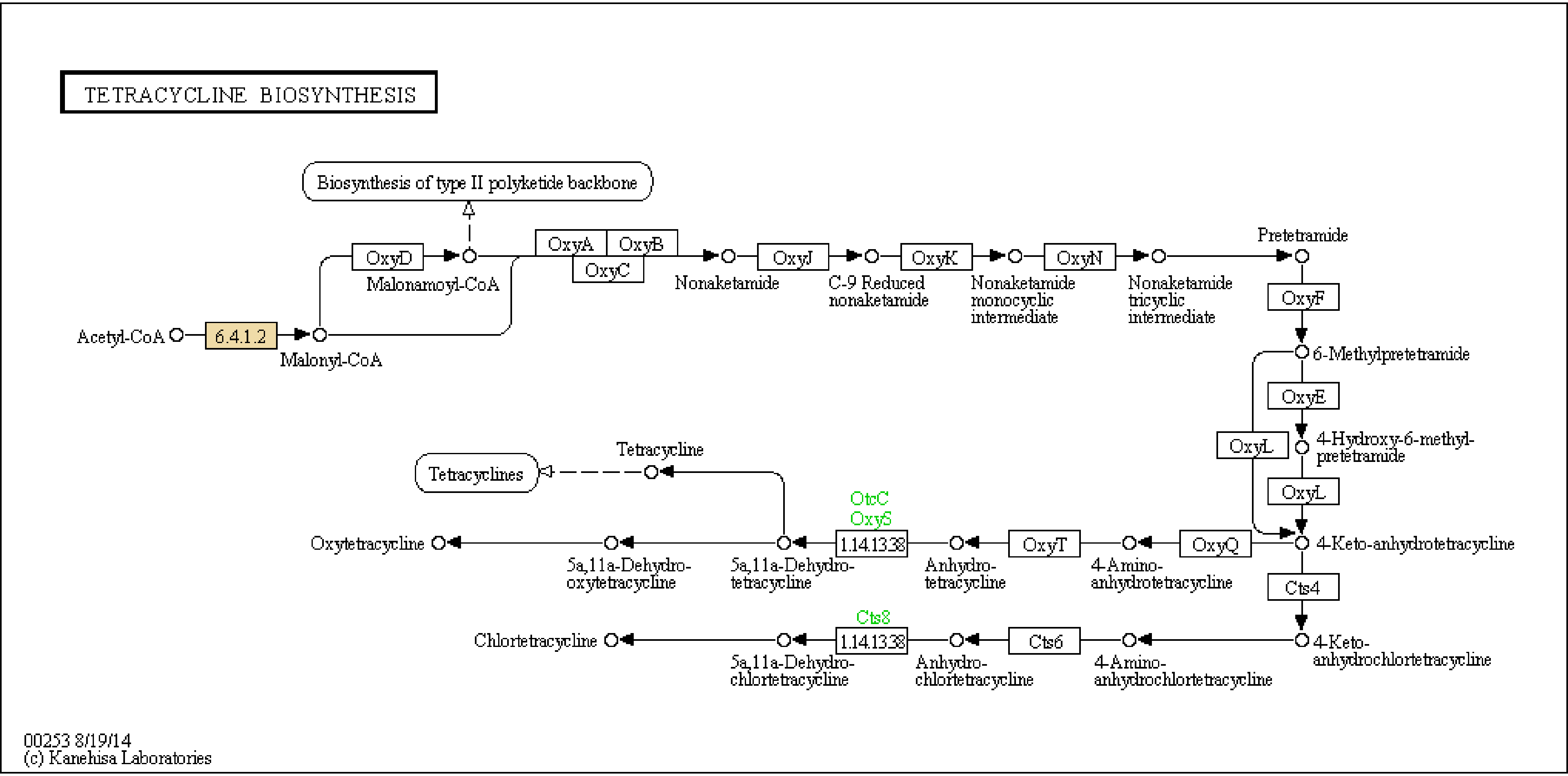
TETRACYCLINE BIOSYNTHESIS
Biosynthesis of type II polyketide backbone
Acetyl-CoA
6.4.1.2
Malonyl-CoA
OxyD
Malonamoyl-CoA
OxyA
OxyB
OxyC
Nonaketamide
OxyJ
C-9 Reduced nonaketamide
OxyK
Nonaketamide monocyclic intermediate
OxyN
Nonaketamide tricyclic intermediate
Pretetramide
OxyF
6-Methylpretetramide
OxyE
4-Hydroxy-6-methyl-pretetramide
OxyL
OxyL
4-Keto-anhydrotetracycline
Cts4
4-Keto-anhydrochlortetracycline
OxyQ
4-Amino-anhydrotetracycline
OxyT
Anhydro-tetracycline
OtcC
OxyS
1.14.13.38
5a,11a-Dehydro-tetracycline
Tetracycline
Tetracyclines
5a,11a-Dehydro-oxytetracycline
Oxytetracycline
Cts6
4-Amino-anhydrochlortetracycline
Anhydro-chlortetracycline
Cts8
1.14.13.38
5a,11a-Dehydro-chlortetracycline
Chlortetracycline
00253 8/19/14
(c) Kanehisa Laboratories

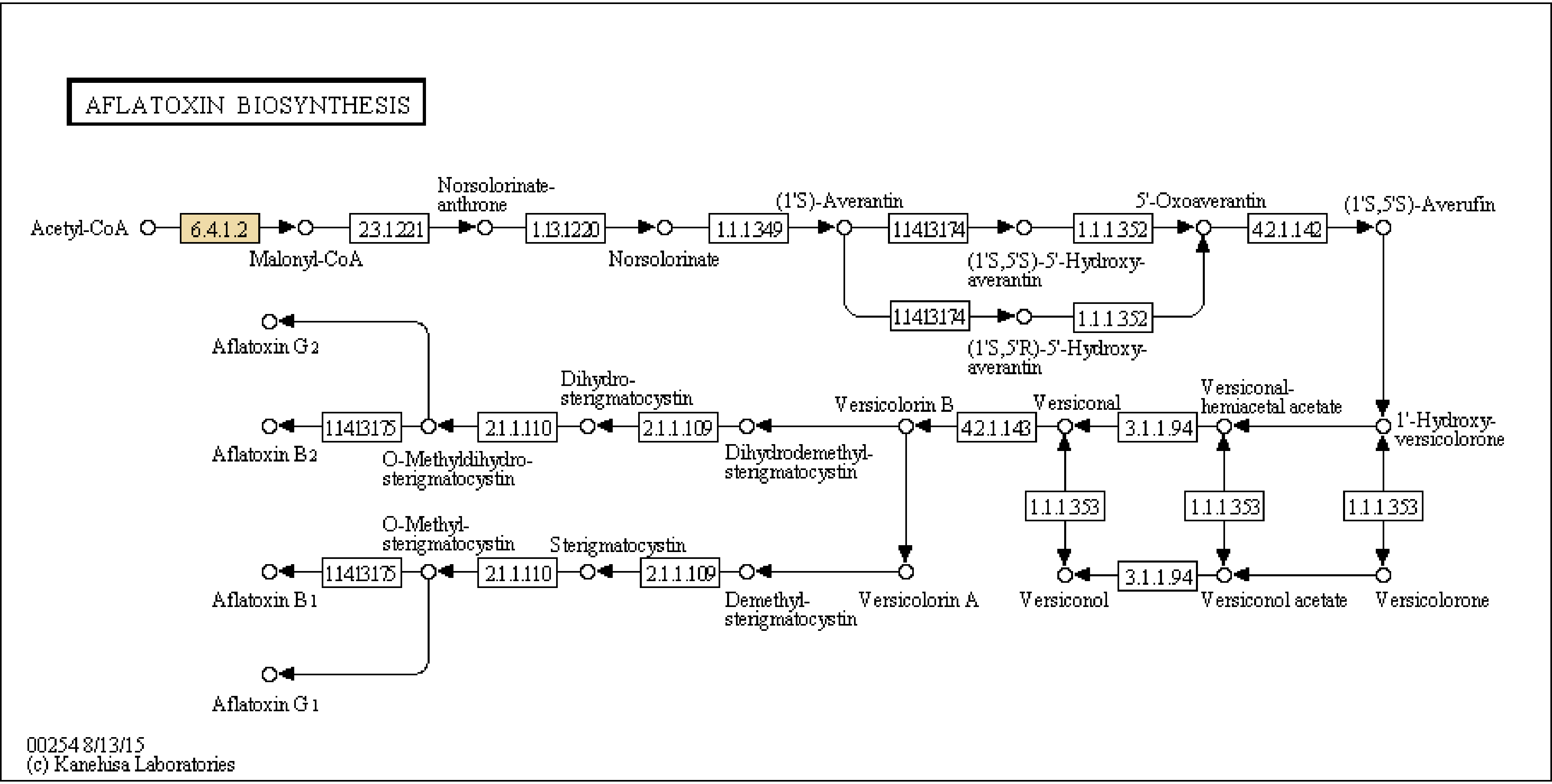
AFLATOXIN BIOSYNTHESIS
Acetyl-CoA
6.4.1.2
Malonyl-CoA
2.3.1.221
Norsolorinate-anthrone
1.13.12.20
Norsolorinate
1.1.1.349
(1'S)-Averantin
1.14.13.174
(1'S,5'S)-5'-Hydroxy-averantin
1.1.1.352
5'-Oxoaverantin
4.2.1.142
(1'S,5'S)-Averufin
1.14.13.174
(1'S,5'R)-5'-Hydroxy-averantin
1.1.1.352
Aflatoxin G2
Aflatoxin B2
1.14.13.175
O-Methyldihydro-sterigmatocystin
2.1.1.110
Dihydro-sterigmatocystin
2.1.1.109
Dihydrodemethyl-sterigmatocystin
Versicolorin B
4.2.1.143
Versiconal
3.1.1.94
Versiconal-hemiacetal acetate
1'-Hydroxy-versicolorone
1.1.1.353
1.1.1.353
1.1.1.353
Aflatoxin B1
1.14.13.175
O-Methyl-sterigmatocystin
2.1.1.110
Sterigmatocystin
2.1.1.109
Demethyl-sterigmatocystin
Versicolorin A
Versiconol
3.1.1.94
Versiconol acetate
Versicolorone
Aflatoxin G1
00254 8/13/15
(c) Kanehisa Laboratories

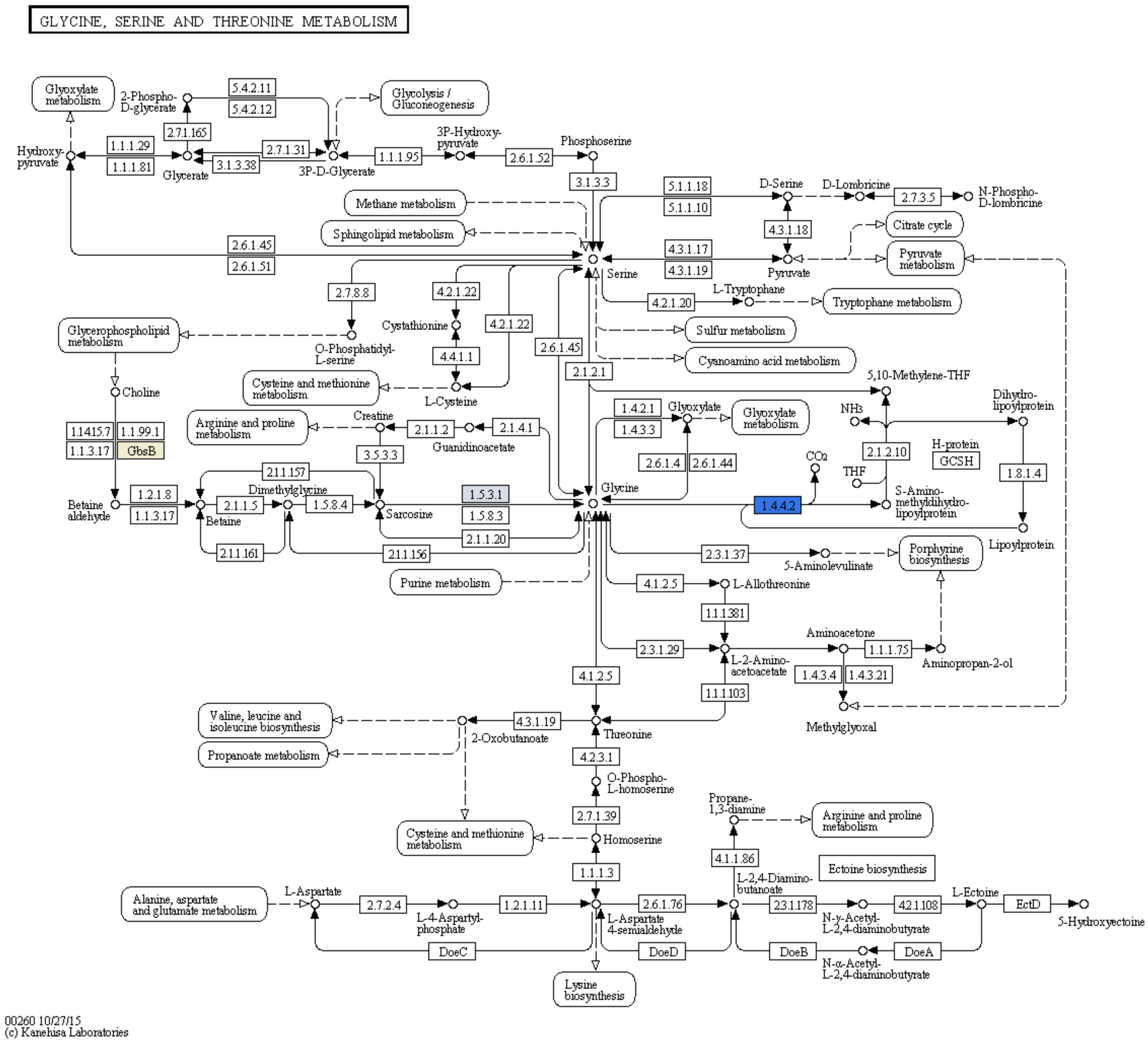
GLYCINE, SERINE AND THREONINE METABOLISM
Glyoxylate metabolism
2-Phospho-D-glycerate
5.4.2.11
5.4.2.12
Glycolysis / Gluconeogenesis
2.7.1.165
Hydroxy-pyruvate
1.1.1.29
1.1.1.81
Glycerate
2.7.1.31
3.1.3.38
3P-D-Glycerate
1.1.1.95
3P-Hydroxy-pyruvate
2.6.1.52
Phosphoserine
3.1.3.3
Methane metabolism
Sphingolipid metabolism
5.1.1.18
5.1.1.10
D-Serine
D-Lombricine
2.7.3.5
N-Phospho-D-lombricine
4.3.1.18
Citrate cycle
4.3.1.17
4.3.1.19
Pyruvate
Pyruvate metabolism
2.6.1.45
2.6.1.51
Serine
L-Tryptophane
4.2.1.20
Tryptophane metabolism
2.7.8.8
4.2.1.22
Cystathionine
4.2.1.22
Sulfur metabolism
Glycerophospholipid metabolism
O-Phosphatidyl-L-serine
4.4.1.1
2.6.1.45
Cyanoamino acid metabolism
2.1.2.1
5,10-Methylene-THF
Cysteine and methionine metabolism
L-Cysteine
Dihydro-lipoylprotein
Choline
1.4.2.1
1.4.3.3
Glyoxylate
Glyoxylate metabolism
NH3
Arginine and proline metabolism
Creatine
2.1.1.2
2.1.4.1
Guanidinoacetate
1.14.15.7
1.1.99.1
1.1.3.17
GbsB
3.5.3.3
H-protein
GCSH
2.1.2.10
2.1.1.157
2.6.1.4
2.6.1.44
CO2
THF
1.8.1.4
Dimethylglycine
Glycine
Betaine aldehyde
1.2.1.8
1.1.3.17
2.1.1.5
1.5.8.4
Sarcosine
1.5.3.1
1.5.8.3
1.4.4.2
S-Amino-methyldihydro-lipoylprotein
Betaine
2.1.1.20
Lipoylprotein
2.1.1.161
2.1.1.156
2.3.1.37
5-Aminolevulinate
Porphyrine biosynthesis
Purine metabolism
4.1.2.5
L-Allothreonine
1.1.1.381
Aminoacetone
2.3.1.29
1.1.1.75
L-2-Amino-acetoacetate
Aminopropan-2-ol
4.1.2.5
1.4.3.4
1.4.3.21
1.1.1.103
Methylglyoxal
Valine, leucine and isoleucine biosynthesis
4.3.1.19
Threonine
2-Oxobutanoate
Propanoate metabolism
4.2.3.1
O-Phospho-L-homoserine
2.7.1.39
Propane-1,3-diamine
Arginine and proline metabolism
Cysteine and methionine metabolism
Homoserine
4.1.1.86
Ectoine biosynthesis
1.1.1.3
L-2,4-Diamino-butanoate
L-Aspartate
Alanine, aspartate and glutamate metabolism
2.7.2.4
1.2.1.11
2.6.1.76
2.3.1.178
4.2.1.108
L-Ectoine
EctD
5-Hydroxyectoine
L-4-Aspartyl-phosphate
L-Aspartate 4-semialdehyde
N-γ-Acetyl-L-2,4-diaminobutyrate
DoeC
DoeD
DoeB
DoeA
N-α-Acetyl-L-2,4-diaminobutyrate
Lysine biosynthesis
00260 10/27/15
(c) Kanehisa Laboratories

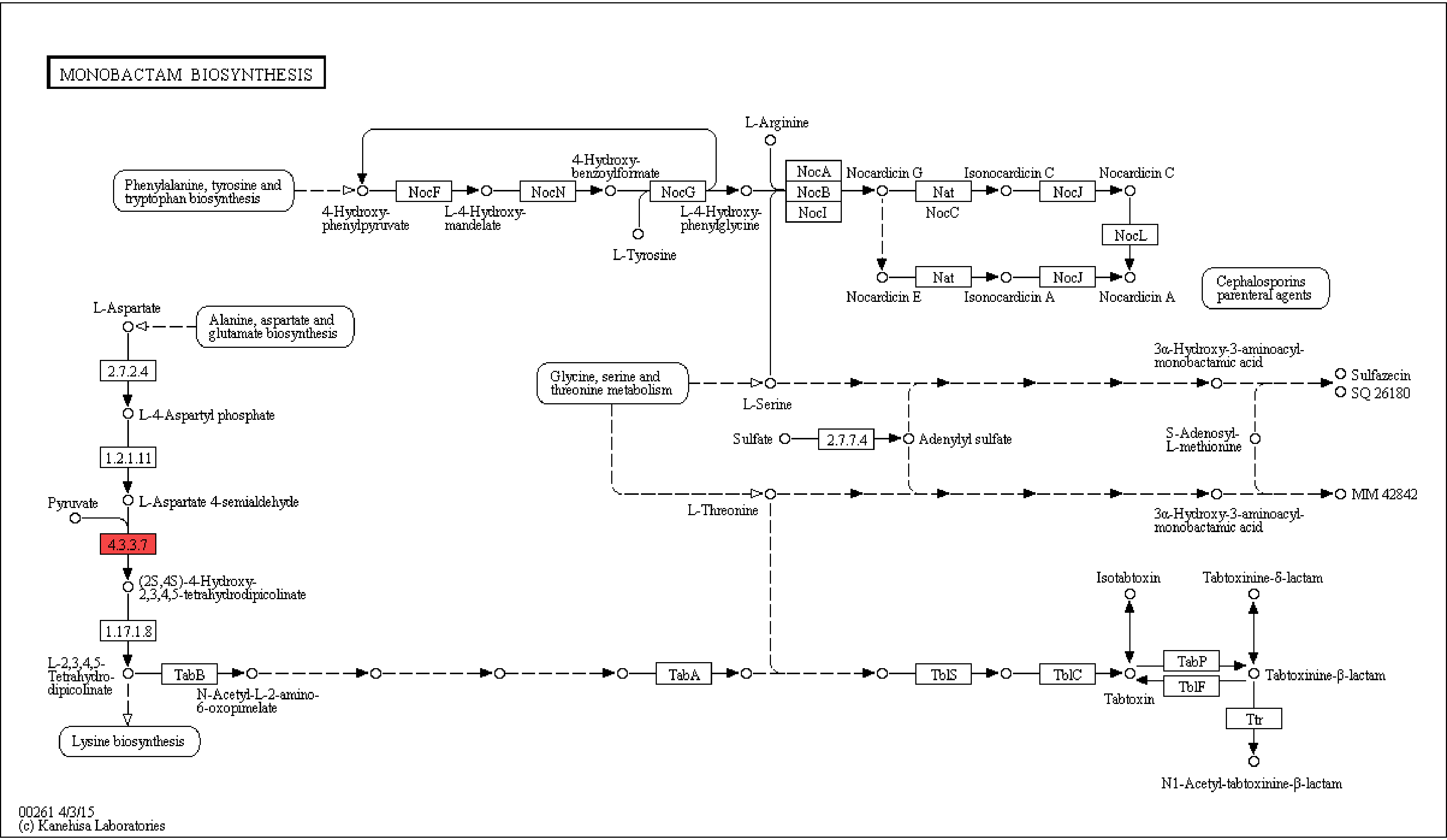
MONOBACTAM BIOSYNTHESIS
Phenylalanine, tyrosine and tryptophan biosynthesis
4-Hydroxy-phenylpyruvate
NocF
L-4-Hydroxy-mandelate
NocN
4-Hydroxy-benzoylformate
NocG
L-Tyrosine
L-4-Hydroxy-phenylglycine
L-Arginine
NocA
NocB
NocI
Nocardicin G
Nat
NocC
Isonocardicin C
NocJ
Nocardicin C
NocL
Nocardicin E
Nat
Isonocardicin A
NocJ
Nocardicin A
Cephalosporins parenteral agents
L-Aspartate
Alanine, aspartate and glutamate biosynthesis
2.7.2.4
L-4-Aspartyl phosphate
1.2.1.11
L-Aspartate 4-semialdehyde
Pyruvate
4.3.3.7
(2S,4S)-4-Hydroxy-2,3,4,5-tetrahydrodipicolinate
1.17.1.8
L-2,3,4,5-Tetrahydro-dipicolinate
TabB
N-Acetyl-L-2-amino-6-oxopimelate
TabA
Lysine biosynthesis
Glycine, serine and threonine metabolism
L-Serine
Sulfate
2.7.7.4
Adenylyl sulfate
3α-Hydroxy-3-aminoacyl-monobactamic acid
Sulfazecin
SQ 26180
S-Adenosyl-L-methionine
L-Threonine
MM 42842
3α-Hydroxy-3-aminoacyl-monobactamic acid
TblS
TblC
Isotabtoxin
Tabtoxin
TabP
TblF
Tabtoxinine-δ-lactam
Tabtoxinine-β-lactam
Ttr
N1-Acetyl-tabtoxinine-β-lactam
00261 4/3/15
(c) Kanehisa Laboratories

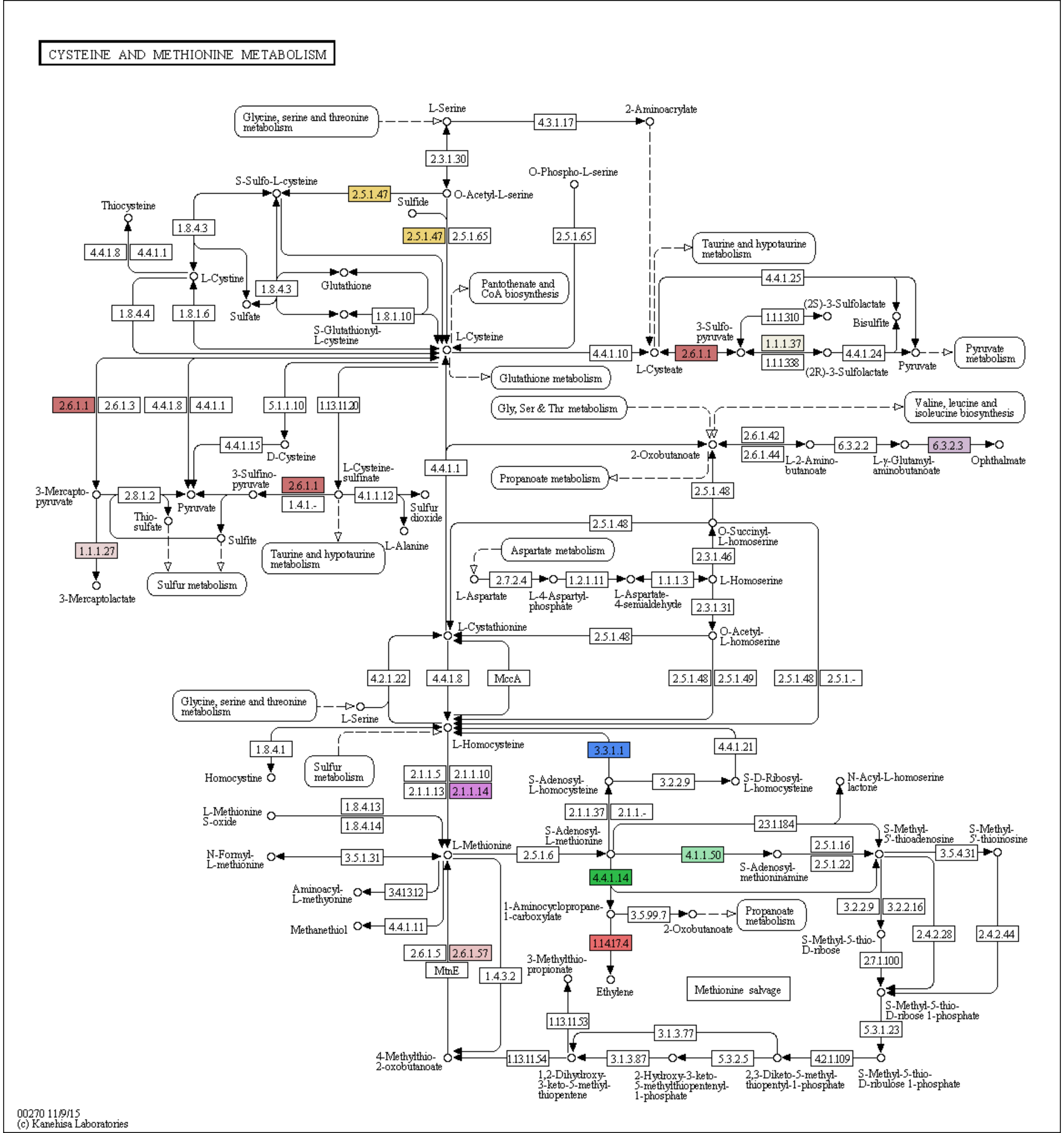
CYSTEINE AND METHIONINE METABOLISM
Glycine, serine and threonine metabolism
L-Serine
4.3.1.17
2-Aminoacrylate
2.3.1.30
O-Phospho-L-serine
S-Sulfo-L-cysteine
2.5.1.47
Sulfide
O-Acetyl-L-serine
Thiocysteine
1.8.4.3
2.5.1.47
2.5.1.65
2.5.1.65
4.4.1.8
4.4.1.1
Taurine and hypotaurine metabolism
L-Cystine
1.8.4.3
Glutathione
Pantothenate and CoA biosynthesis
4.4.1.25
1.8.4.4
1.8.1.6
Sulfate
S-Glutathionyl-L-cysteine
1.8.1.10
(2S)-3-Sulfolactate
1.1.1.310
Bisulfite
3-Sulfo-pyruvate
1.1.1.37
L-Cysteine
4.4.1.10
2.6.1.1
1.1.1.338
4.4.1.24
Pyruvate
Pyruvate metabolism
L-Cysteate
(2R)-3-Sulfolactate
Glutathione metabolism
2.6.1.1
2.6.1.3
4.4.1.8
4.4.1.1
5.1.1.10
1.13.11.20
Gly, Ser & Thr metabolism
Valine, leucine and isoleucine biosynthesis
2.6.1.42
2.6.1.44
6.3.2.2
6.3.2.3
4.4.1.15
D-Cysteine
2-Oxobutanoate
L-2-Amino-butanoate
L-γ-Glutamyl-aminobutanoate
Ophthalmate
3-Sulfino-pyruvate
L-Cysteine-sulfinate
4.4.1.1
Propanoate metabolism
3-Mercapto-pyruvate
2.8.1.2
Pyruvate
2.6.1.1
1.4.1.-
4.1.1.12
Sulfur dioxide
2.5.1.48
Thio-sulfate
Sulfite
L-Alanine
2.5.1.48
O-Succinyl-L-homoserine
1.1.1.27
Taurine and hypotaurine metabolism
Aspartate metabolism
2.3.1.46
Sulfur metabolism
2.7.2.4
1.2.1.11
1.1.1.3
L-Homoserine
3-Mercaptolactate
L-Aspartate
L-4-Aspartyl-phosphate
L-Aspartate-4-semialdehyde
2.3.1.31
L-Cystathionine
2.5.1.48
O-Acetyl-L-homoserine
4.2.1.22
4.4.1.8
MccA
2.5.1.48
2.5.1.49
2.5.1.48
2.5.1.-
Glycine, serine and threonine metabolism
L-Serine
L-Homocysteine
1.8.4.1
3.3.1.1
4.4.1.21
Sulfur metabolism
Homocystine
2.1.1.5
2.1.1.10
S-Adenosyl-L-homocysteine
3.2.2.9
S-D-Ribosyl-L-homocysteine
N-Acyl-L-homoserine lactone
2.1.1.13
2.1.1.14
L-Methionine S-oxide
1.8.4.13
2.1.1.37
2.1.1.-
1.8.4.14
2.3.1.184
S-Methyl-5'-thioadenosine
S-Methyl-5'-thioinosine
S-Adenosyl-L-methionine
2.5.1.16
N-Formyl-L-methionine
3.5.1.31
L-Methionine
2.5.1.6
4.1.1.50
3.5.4.31
2.5.1.22
S-Adenosyl-methioninamine
4.4.1.14
Aminoacyl-L-methionine
3.4.13.12
1-Aminocyclopropane-1-carboxylate
3.5.99.7
Propanoate metabolism
3.2.2.9
3.2.2.16
2-Oxobutanoate
Methanethiol
4.4.1.11
S-Methyl-5-thio-D-ribose
2.4.2.28
2.4.2.44
1.14.17.4
2.6.1.5
2.6.1.57
3-Methylthio-propionate
2.7.1.100
MtnE
1.4.3.2
Ethylene
Methionine salvage
S-Methyl-5-thio-D-ribose 1-phosphate
1.13.11.53
3.1.3.77
5.3.1.23
4-Methylthio-2-oxobutanoate
1.13.11.54
3.1.3.87
5.3.2.5
4.2.1.109
1,2-Dihydroxy-3-keto-5-methyl-thiopentene
2-Hydroxy-3-keto-5-methylthiopentenyl-1-phosphate
2,3-Diketo-5-methyl-thiopentyl-1-phosphate
S-Methyl-5-thio-D-ribulose 1-phosphate
00270 11/9/15
(c) Kanehisa Laboratories

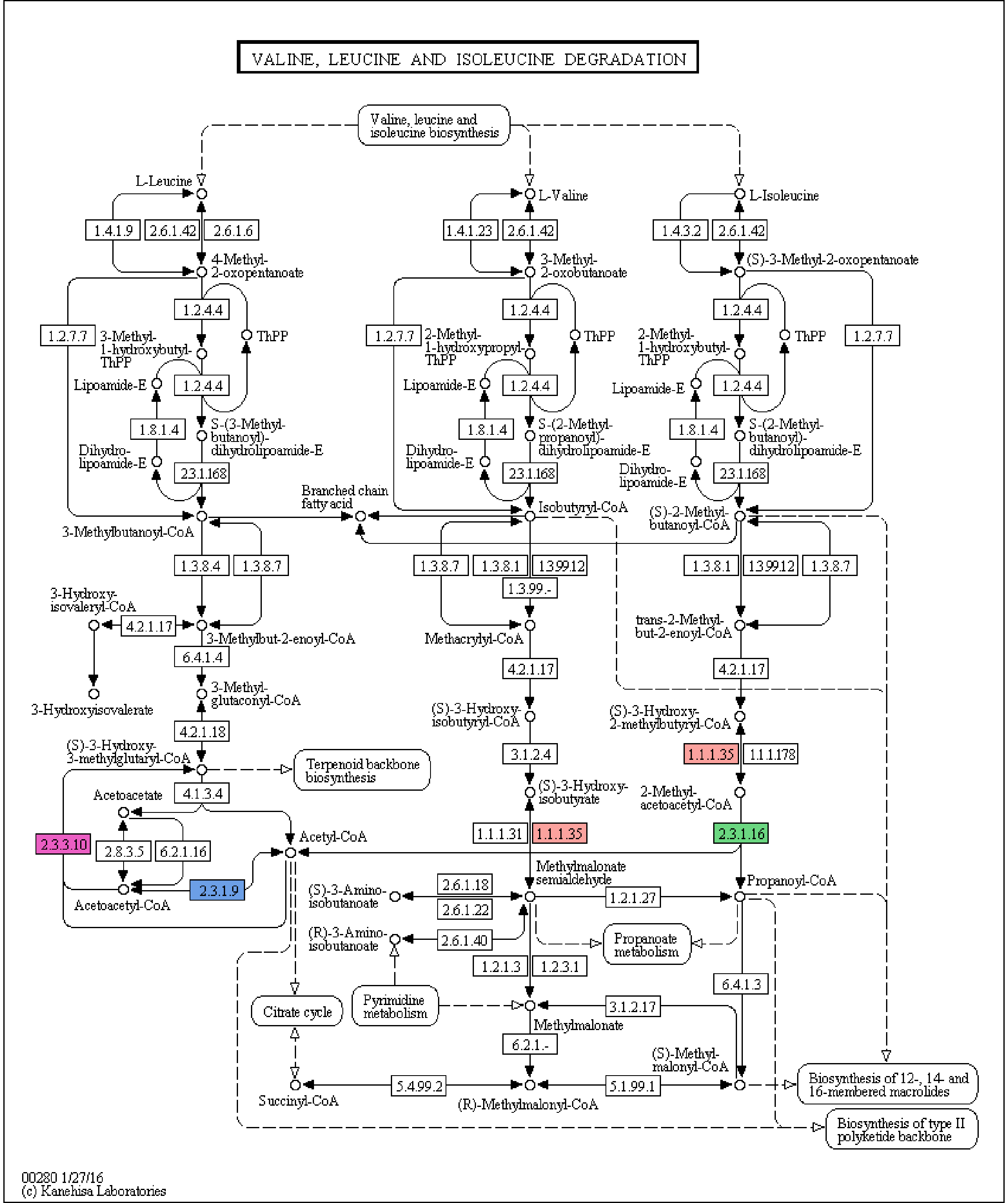
VALINE, LEUCINE AND ISOLEUCINE DEGRADATION
Valine, leucine and isoleucine biosynthesis
L-Leucine
L-Valine
L-Isoleucine
1.4.1.9
2.6.1.42
2.6.1.6
1.4.1.23
2.6.1.42
1.4.3.2
2.6.1.42
4-Methyl-2-oxopentanoate
3-Methyl-2-oxobutanoate
(S)-3-Methyl-2-oxopentanoate
1.2.4.4
1.2.4.4
1.2.4.4
1.2.7.7
1.2.7.7
1.2.7.7
ThPP
ThPP
ThPP
3-Methyl-1-hydroxybutyl-ThPP
2-Methyl-1-hydroxypropyl-ThPP
2-Methyl-1-hydroxybutyl-ThPP
Lipoamide-E
Lipoamide-E
Lipoamide-E
1.2.4.4
1.2.4.4
1.2.4.4
1.8.1.4
1.8.1.4
1.8.1.4
S-(3-Methylbutanoyl)-dihydrolipoamide-E
S-(2-Methylpropanoyl)-dihydrolipoamide-E
S-(2-Methylbutanoyl)-dihydrolipoamide-E
Dihydrolipoamide-E
Dihydrolipoamide-E
Dihydrolipoamide-E
2.3.1.168
2.3.1.168
2.3.1.168
Branched chain fatty acid
3-Methylbutanoyl-CoA
Isobutyryl-CoA
(S)-2-Methylbutanoyl-CoA
1.3.8.4
1.3.8.7
1.3.8.7
1.3.8.1
1.3.99.12
1.3.99.-
1.3.8.1
1.3.99.12
1.3.8.7
3-Hydroxyisovaleryl-CoA
4.2.1.17
3-Methylbut-2-enoyl-CoA
Methacrylyl-CoA
trans-2-Methylbut-2-enoyl-CoA
6.4.1.4
3-Hydroxyisovalerate
3-Methylglutaconyl-CoA
4.2.1.17
4.2.1.17
4.2.1.18
(S)-3-Hydroxyisobutyryl-CoA
(S)-3-Hydroxy-2-methylbutyryl-CoA
(S)-3-Hydroxy-3-methylglutaryl-CoA
Terpenoid backbone biosynthesis
3.1.2.4
1.1.1.35
1.1.1.178
4.1.3.4
(S)-3-Hydroxyisobutyrate
2-Methylacetoacetyl-CoA
Acetoacetate
Acetyl-CoA
1.1.1.31
1.1.1.35
2.3.1.16
2.3.3.10
2.8.3.5
6.2.1.16
Methylmalonate semialdehyde
Propanoyl-CoA
Acetoacetyl-CoA
2.3.1.9
(S)-3-Aminoisobutanoate
2.6.1.18
2.6.1.22
1.2.1.27
(R)-3-Aminoisobutanoate
2.6.1.40
Propanoate metabolism
1.2.1.3
1.2.3.1
6.4.1.3
Citrate cycle
Pyrimidine metabolism
3.1.2.17
Methylmalonate
6.2.1.-
(S)-Methylmalonyl-CoA
Biosynthesis of 12-, 14- and 16-membered macrolides
Succinyl-CoA
5.4.99.2
(R)-Methylmalonyl-CoA
5.1.99.1
Biosynthesis of type II polyketide backbone
00280 1/27/16
(c) Kanehisa Laboratories

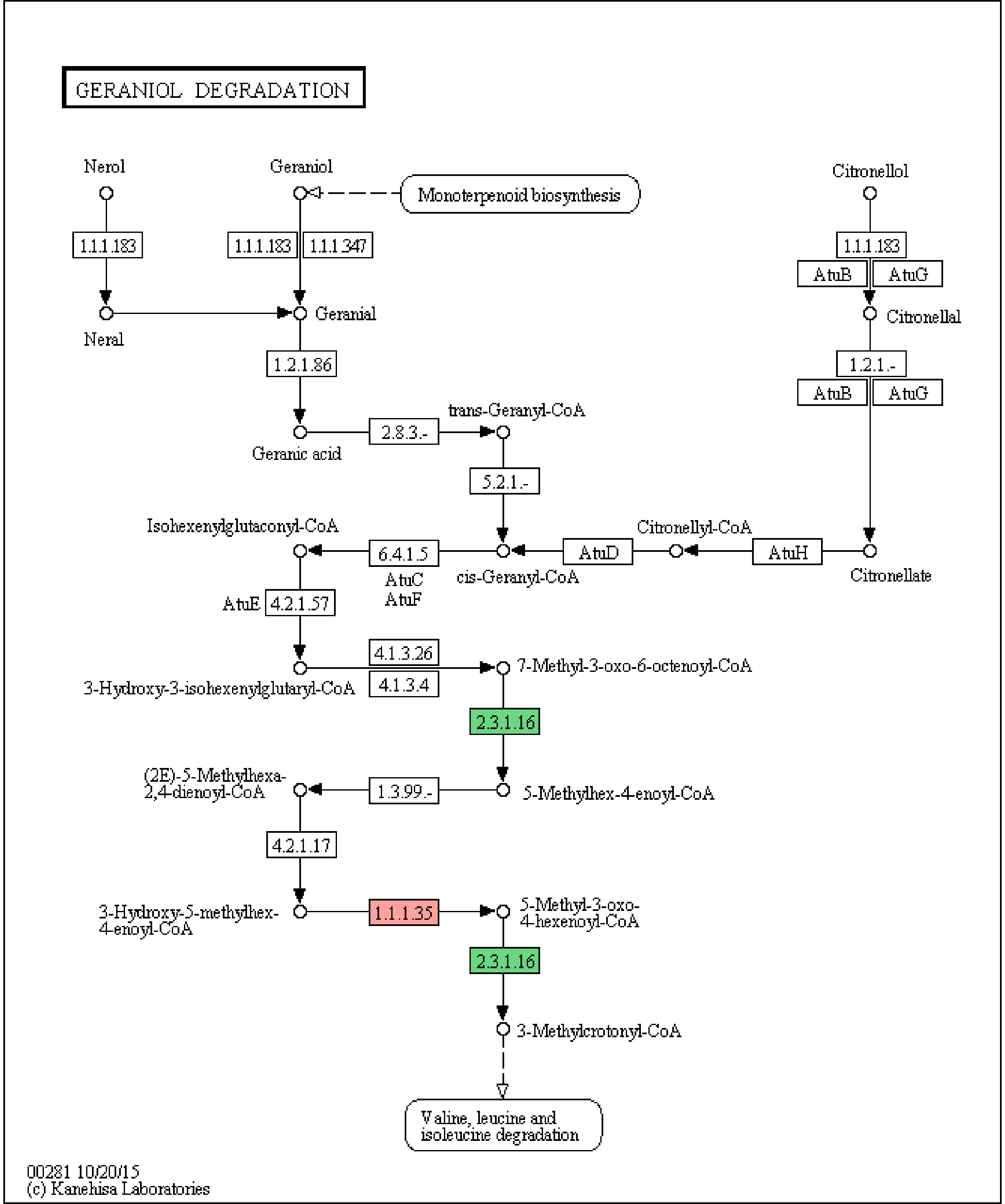
GERANIOL DEGRADATION
Nerol
Geraniol
Monoterpenoid biosynthesis
Citronellol
1.1.1.183
1.1.1.183
1.1.1.347
1.1.1.183
AtuB
AtuG
Neral
Geranial
Citronellal
1.2.1.86
1.2.1.-
AtuB
AtuG
trans-Geranyl-CoA
2.8.3.-
Geranic acid
5.2.1.-
Isohexenylglutaconyl-CoA
6.4.1.5
AtuC
AtuF
cis-Geranyl-CoA
AtuD
Citronellyl-CoA
AtuH
Citronellate
AtuE
4.2.1.57
4.1.3.26
4.1.3.4
7-Methyl-3-oxo-6-octenoyl-CoA
3-Hydroxy-3-isohexenylglutaryl-CoA
2.3.1.16
(2E)-5-Methylhexa-2,4-dienoyl-CoA
1.3.99.-
5-Methylhex-4-enoyl-CoA
4.2.1.17
3-Hydroxy-5-methylhex-4-enoyl-CoA
1.1.1.35
5-Methyl-3-oxo-4-hexenoyl-CoA
2.3.1.16
3-Methylcrotonyl-CoA
Valine, leucine and isoleucine degradation
00281 10/20/15
(c) Kanehisa Laboratories

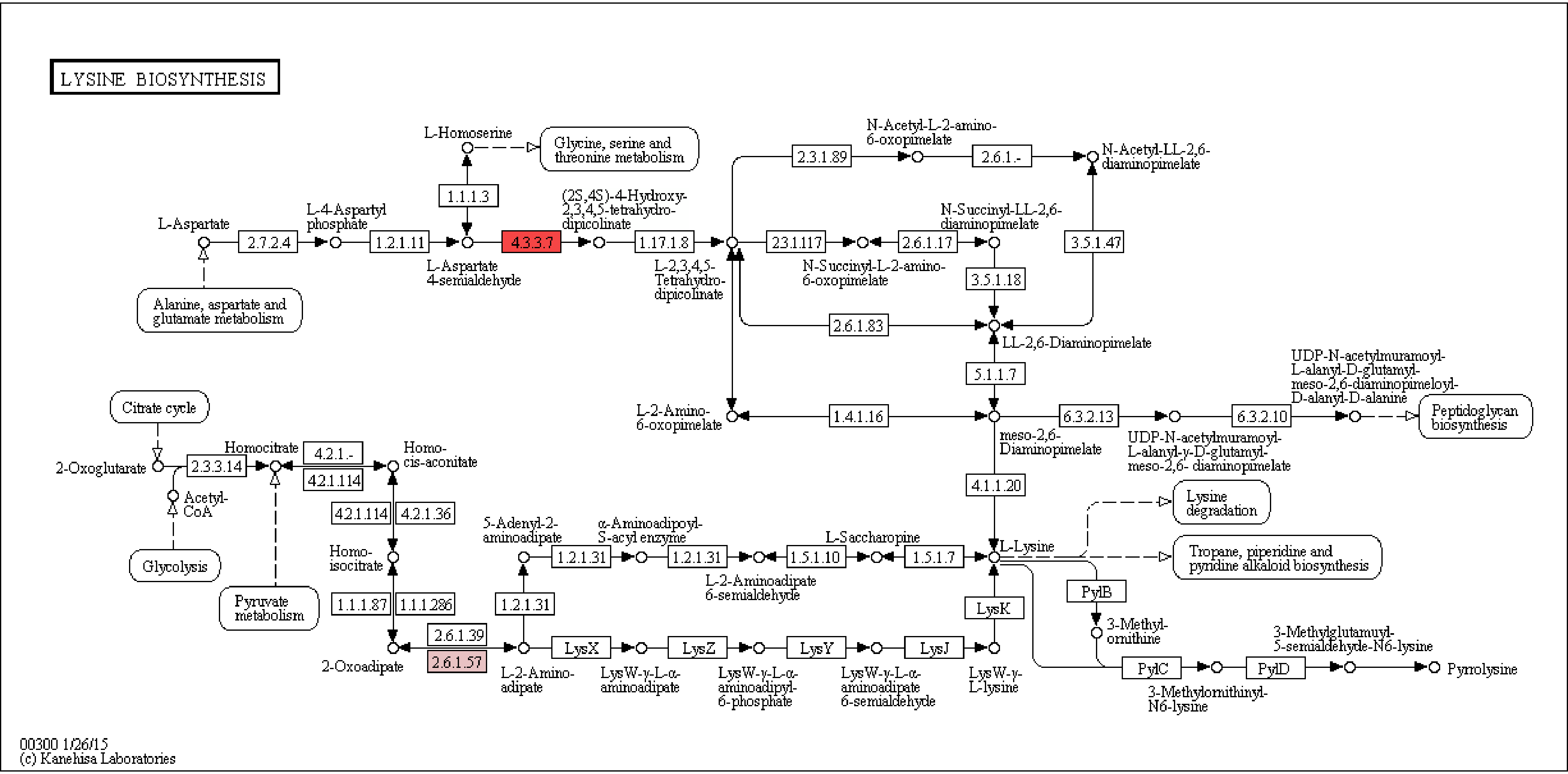
LYSINE BIOSYNTHESIS
L-Homoserine
Glycine, serine and threonine metabolism
1.1.1.3
(2S,4S)-4-Hydroxy-2,3,4,5-tetrahydro-dipicolinate
L-Aspartate
L-4-Aspartyl phosphate
2.7.2.4
1.2.1.11
4.3.3.7
1.17.1.8
L-Aspartate 4-semialdehyde
L-2,3,4,5-Tetrahydro-dipicolinate
Alanine, aspartate and glutamate metabolism
2.3.1.89
N-Acetyl-L-2-amino-6-oxopimelate
2.6.1.-
N-Acetyl-LL-2,6-diaminopimelate
N-Succinyl-LL-2,6-diaminopimelate
2.3.1.117
2.6.1.17
3.5.1.47
N-Succinyl-L-2-amino-6-oxopimelate
3.5.1.18
2.6.1.83
LL-2,6-Diaminopimelate
5.1.1.7
UDP-N-acetylmuramoyl-L-alanyl-D-glutamyl-meso-2,6-diaminopimeloyl-D-alanyl-D-alanine
L-2-Amino-6-oxopimelate
1.4.1.16
6.3.2.13
6.3.2.10
Peptidoglycan biosynthesis
meso-2,6-Diaminopimelate
UDP-N-acetylmuramoyl-L-alanyl-γ-D-glutamyl-meso-2,6-diaminopimelate
Citrate cycle
2-Oxoglutarate
2.3.3.14
Homocitrate
4.2.1.-
4.2.1.114
Homo-cis-aconitate
Acetyl-CoA
4.2.1.114
4.2.1.36
4.1.1.20
Lysine degradation
Glycolysis
Homo-isocitrate
5-Adenyl-2-aminoadipate
α-Aminoadipoyl-S-acyl enzyme
L-Saccharopine
L-Lysine
1.2.1.31
1.2.1.31
1.5.1.10
1.5.1.7
Tropane, piperidine and pyridine alkaloid biosynthesis
L-2-Aminoadipate 6-semialdehyde
Pyruvate metabolism
1.1.1.87
1.1.1.286
1.2.1.31
PylB
LysK
3-Methyl-ornithine
2.6.1.39
2.6.1.57
LysX
LysZ
LysY
LysJ
3-Methylglutamuyl-5-semialdehyde-N6-lysine
PylC
PylD
Pyrrolysine
2-Oxoadipate
L-2-Amino-adipate
LysW-γ-L-α-aminoadipate
LysW-γ-L-α-aminoadipyl-6-phosphate
LysW-γ-L-α-aminoadipate 6-semialdehyde
LysW-γ-L-lysine
3-Methylornithinyl-N6-lysine
00300 1/26/15
(c) Kanehisa Laboratories

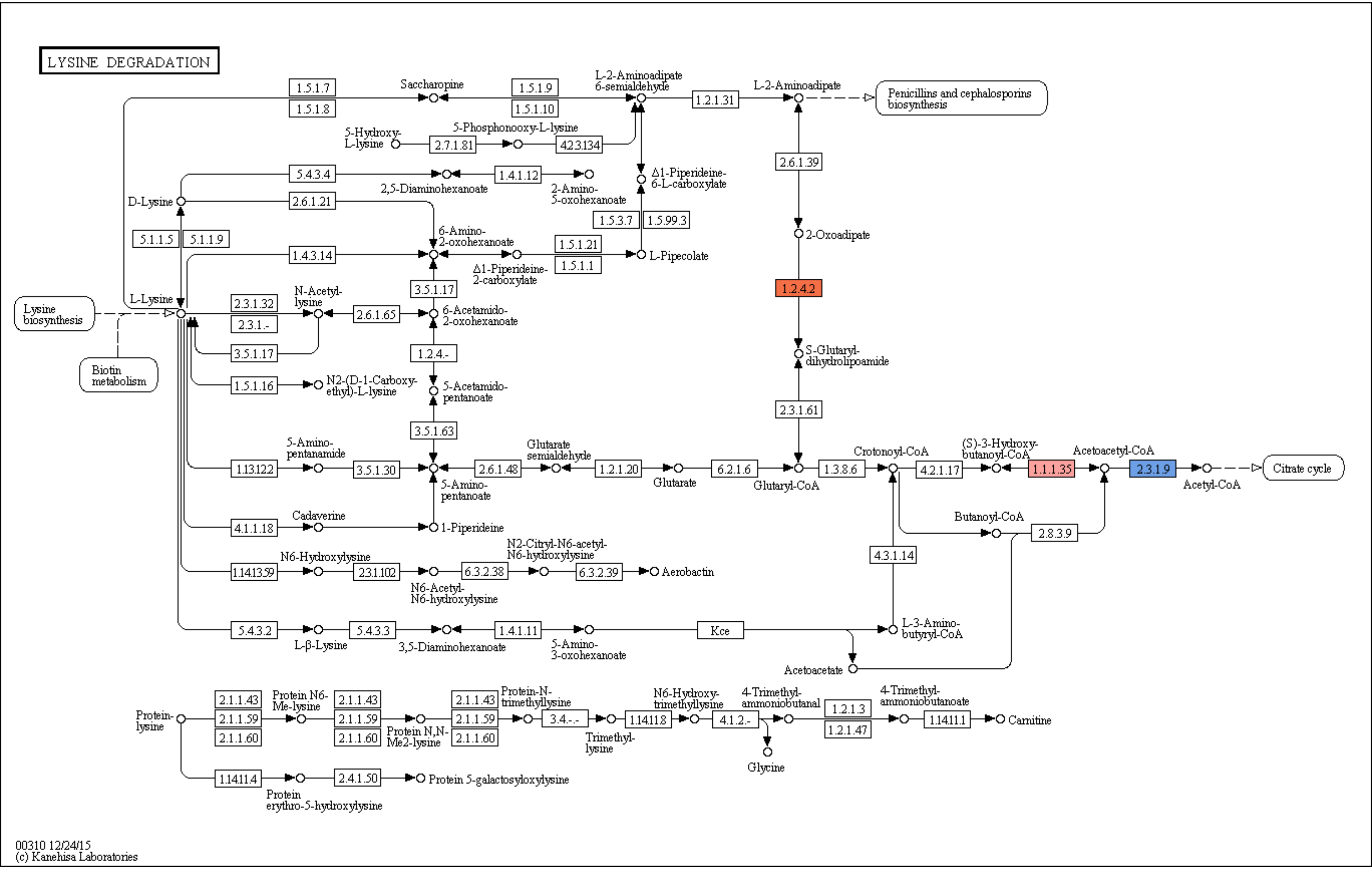
LYSINE DEGRADATION
1.5.1.7
1.5.1.8
Saccharopine
1.5.1.9
1.5.1.10
L-2-Aminoadipate 6-semialdehyde
1.2.1.31
L-2-Aminoadipate
Penicillins and cephalosporins biosynthesis
5-Hydroxy-L-lysine
2.7.1.81
5-Phosphonooxy-L-lysine
4.2.3.134
5.4.3.4
2,5-Diaminohexanoate
1.4.1.12
2-Amino-5-oxohexanoate
Δ1-Piperideine-6-L-carboxylate
2.6.1.39
D-Lysine
2.6.1.21
6-Amino-2-oxohexanoate
1.5.3.7
1.5.99.3
2-Oxoadipate
5.1.1.5
5.1.1.9
1.4.3.14
1.5.1.21
1.5.1.1
L-Pipecolate
Δ1-Piperideine-2-carboxylate
1.2.4.2
Lysine biosynthesis
L-Lysine
2.3.1.32
2.3.1.-
N-Acetyl-lysine
2.6.1.65
3.5.1.17
6-Acetamido-2-oxohexanoate
3.5.1.17
1.2.4.-
S-Glutaryl-dihydrolipoamide
Biotin metabolism
1.5.1.16
N2-(D-1-Carboxy-ethyl)-L-lysine
5-Acetamido-pentanoate
2.3.1.61
3.5.1.63
5-Amino-pentanamide
1.13.12.2
3.5.1.30
2.6.1.48
Glutarate semialdehyde
1.2.1.20
Glutarate
6.2.1.6
Glutaryl-CoA
1.3.8.6
Crotonoyl-CoA
4.2.1.17
(S)-3-Hydroxy-butanoyl-CoA
1.1.1.35
Acetoacetyl-CoA
2.3.1.9
Acetyl-CoA
Citrate cycle
5-Amino-pentanoate
4.1.1.18
Cadaverine
1-Piperideine
Butanoyl-CoA
2.8.3.9
N6-Hydroxylysine
1.14.13.59
2.3.1.102
N6-Acetyl-N6-hydroxylysine
6.3.2.38
N2-Citryl-N6-acetyl-N6-hydroxylysine
6.3.2.39
Aerobactin
4.3.1.14
5.4.3.2
L-β-Lysine
5.4.3.3
3,5-Diaminohexanoate
1.4.1.11
5-Amino-3-oxohexanoate
Kce
L-3-Amino-butyryl-CoA
Acetoacetate
Protein-lysine
2.1.1.43
2.1.1.59
2.1.1.60
Protein N6-Me-lysine
2.1.1.43
2.1.1.59
2.1.1.60
Protein N,N-Me2-lysine
2.1.1.43
2.1.1.59
2.1.1.60
Protein-N-trimethyllysine
3.4.-.-
Trimethyl-lysine
1.14.11.8
N6-Hydroxy-trimethyllysine
4.1.2.-
Glycine
4-Trimethyl-ammoniobutanal
1.2.1.3
1.2.1.47
4-Trimethyl-ammoniobutanoate
1.14.11.1
Carnitine
1.14.11.4
Protein erythro-5-hydroxylysine
2.4.1.50
Protein 5-galactosyloxylysine
00310 12/24/15
(c) Kanehisa Laboratories

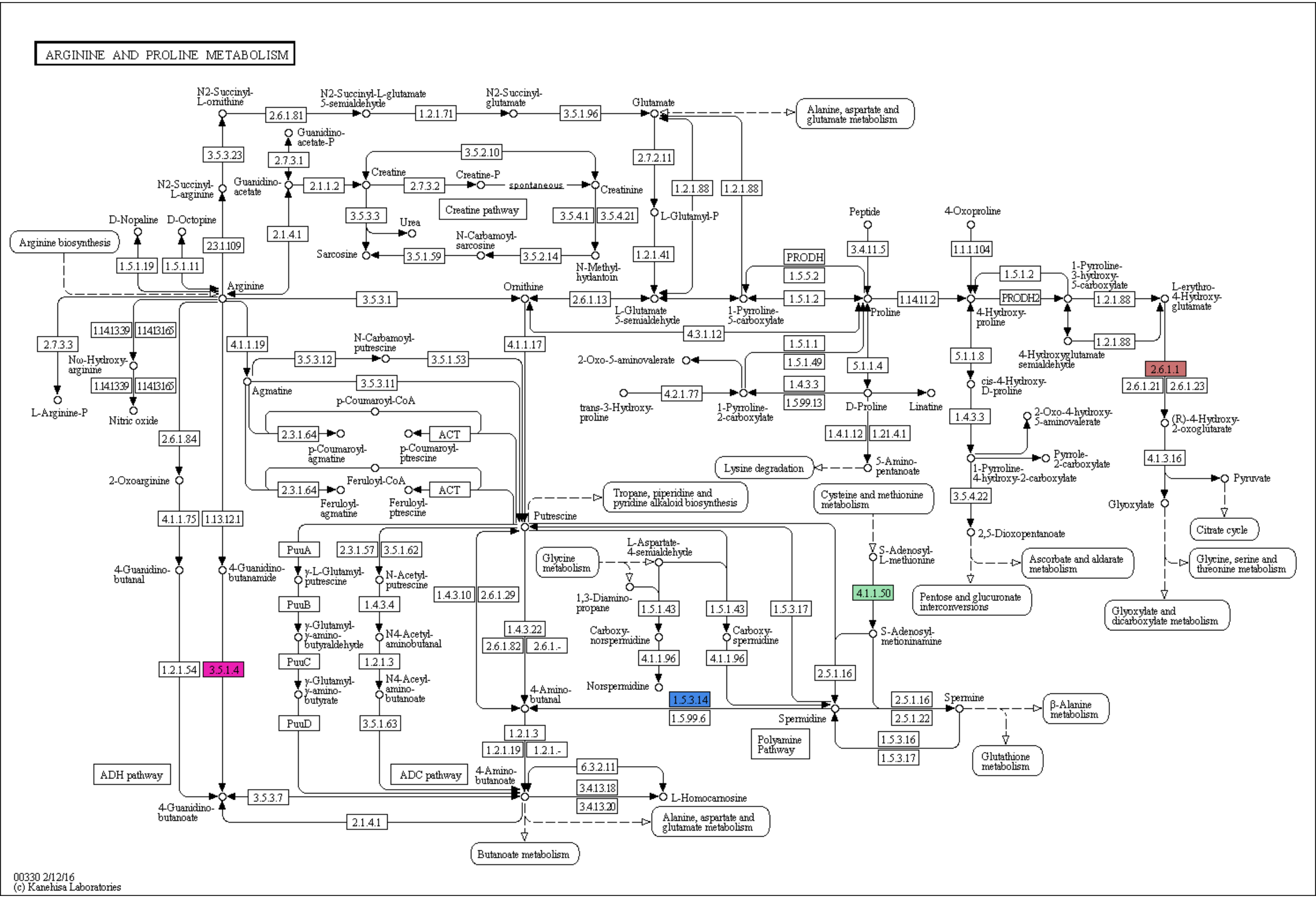
ARGININE AND PROLINE METABOLISM
N2-Succinyl-L-ornithine
2.6.1.81
N2-Succinyl-L-glutamate 5-semialdehyde
1.2.1.71
N2-Succinyl-glutamate
3.5.1.96
Glutamate
Alanine, aspartate and glutamate metabolism
3.5.3.23
Guanidino-acetate-P
2.7.3.1
3.5.2.10
N2-Succinyl-L-arginine
Guanidino-acetate
2.1.1.2
Creatine
2.7.3.2
Creatine-P
spontaneous
Creatinine
2.7.2.11
1.2.1.88
1.2.1.88
Creatine pathway
3.5.3.3
Urea
3.5.4.1
3.5.4.21
L-Glutamyl-P
Peptide
4-Oxoproline
D-Nopaline
D-Octopine
2.1.4.1
N-Carbamoyl-sarcosine
Arginine biosynthesis
2.3.1.109
Sarcosine
3.5.1.59
3.5.2.14
N-Methyl-hydantoin
1.2.1.41
PRODH
3.4.11.5
1.1.1.104
1.5.1.19
1.5.1.11
1.5.5.2
1.5.1.2
1-Pyrroline-3-hydroxy-5-carboxylate
L-erythro-4-Hydroxy-glutamate
Arginine
Ornithine
3.5.3.1
2.6.1.13
L-Glutamate 5-semialdehyde
1-Pyrroline-5-carboxylate
1.5.1.2
Proline
1.14.11.2
PRODH2
1.2.1.88
4-Hydroxy-proline
4.3.1.12
1.2.1.88
2.7.3.3
1.14.13.39
1.14.13.165
4.1.1.19
N-Carbamoyl-putrescine
4.1.1.17
1.5.1.1
4-Hydroxyglutamate semialdehyde
1.5.1.49
5.1.1.4
5.1.1.8
2.6.1.1
Nω-Hydroxy-arginine
3.5.3.12
3.5.1.53
2-Oxo-5-aminovalerate
2.6.1.21
2.6.1.23
1.14.13.39
1.14.13.165
Agmatine
3.5.3.11
trans-3-Hydroxy-proline
4.2.1.77
1.4.3.3
cis-4-Hydroxy-D-proline
L-Arginine-P
Nitric oxide
p-Coumaroyl-CoA
1-Pyrroline-2-carboxylate
1.5.99.13
D-Proline
Linatine
2-Oxo-4-hydroxy-5-aminovalerate
(R)-4-Hydroxy-2-oxoglutarate
2.3.1.64
ACT
1.4.3.3
2.6.1.84
p-Coumaroyl-agmatine
p-Coumaroyl-ptrescine
1.4.1.12
1.21.4.1
Pyrrole-2-carboxylate
4.1.3.16
Lysine degradation
5-Amino-pentanoate
1-Pyrroline-4-hydroxy-2-carboxylate
2-Oxoarginine
Feruloyl-CoA
Pyruvate
2.3.1.64
ACT
Tropane, piperidine and pyridine alkaloid biosynthesis
Cysteine and methionine metabolism
3.5.4.22
Glyoxylate
Feruloyl-agmatine
Feruloyl-ptrescine
Putrescine
4.1.1.75
1.13.12.1
Citrate cycle
2,5-Dioxopentanoate
L-Aspartate-4-semialdehyde
S-Adenosyl-L-methionine
PuuA
2.3.1.57
3.5.1.62
Glycine metabolism
Ascorbate and aldarate metabolism
Glycine, serine and threonine metabolism
4-Guanidino-butanal
4-Guanidino-butanamide
γ-L-Glutamyl-putrescine
N-Acetyl-putrescine
1,3-Diamino-propane
4.1.1.50
1.4.3.10
2.6.1.29
Pentose and glucuronate interconversions
Glyoxylate and dicarboxylate metabolism
PuuB
1.4.3.4
1.5.1.43
1.5.1.43
1.5.3.17
1.4.3.22
Carboxy-norspermidine
Carboxy-spermidine
S-Adenosyl-metioninamine
γ-Glutamyl-γ-amino-butyraldehyde
N4-Acetyl-aminobutanal
2.6.1.82
2.6.1.-
4.1.1.96
4.1.1.96
PuuC
1.2.1.3
2.5.1.16
1.2.1.54
3.5.1.4
Norspermidine
γ-Glutamyl-γ-amino-butyrate
N4-Aceyl-amino-butanoate
4-Amino-butanal
1.5.3.14
2.5.1.16
Spermine
β-Alanine metabolism
1.5.99.6
Spermidine
2.5.1.22
PuuD
3.5.1.63
1.2.1.3
Polyamine Pathway
1.5.3.16
1.2.1.19
1.2.1.-
1.5.3.17
Glutathione metabolism
ADH pathway
ADC pathway
4-Amino-butanoate
6.3.2.11
3.4.13.18
3.5.3.7
L-Homocarnosine
3.4.13.20
4-Guanidino-butanoate
2.1.4.1
Alanine, aspartate and glutamate metabolism
Butanoate metabolism
00330 2/12/16
(c) Kanehisa Laboratories

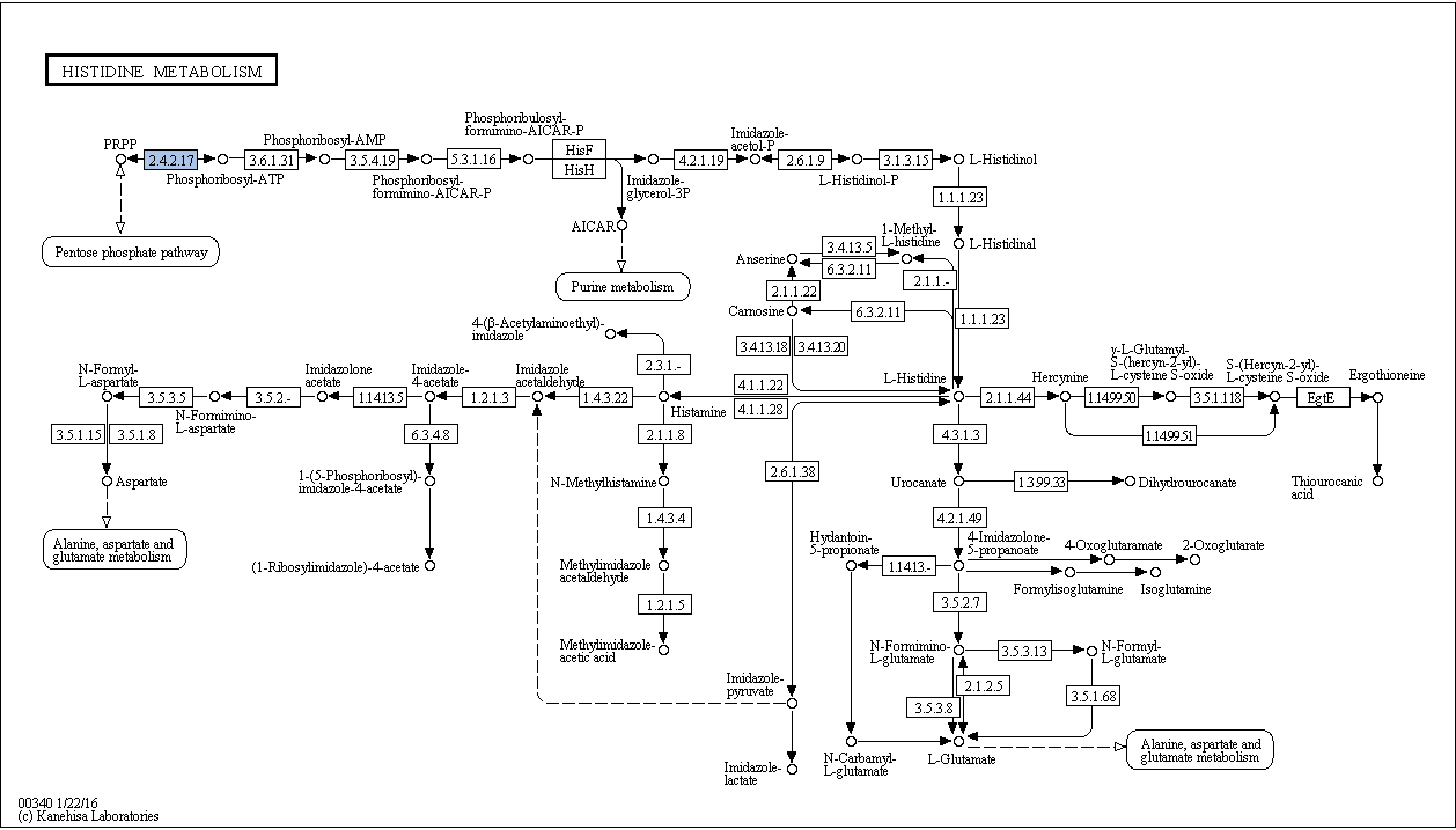
HISTIDINE METABOLISM
PRPP
2.4.2.17
Phosphoribosyl-ATP
3.6.1.31
Phosphoribosyl-AMP
3.5.4.19
Phosphoribosyl-formimino-AICAR-P
5.3.1.16
Phosphoribulosyl-formimino-AICAR-P
HisF
HisH
Imidazole-glycerol-3P
4.2.1.19
Imidazole-acetol-P
2.6.1.9
L-Histidinol-P
3.1.3.15
L-Histidinol
1.1.1.23
L-Histidinal
AICAR
Pentose phosphate pathway
Purine metabolism
1-Methyl-L-histidine
3.4.13.5
6.3.2.11
Anserine
2.1.1.-
2.1.1.22
Carnosine
6.3.2.11
1.1.1.23
3.4.13.18
3.4.13.20
4-(β-Acetylaminoethyl)-imidazole
2.3.1.-
4.1.1.22
4.1.1.28
L-Histidine
Histamine
N-Formyl-L-aspartate
3.5.3.5
N-Formimino-L-aspartate
3.5.2.-
Imidazolone acetate
1.14.13.5
Imidazole-4-acetate
1.2.1.3
Imidazole acetaldehyde
1.4.3.22
3.5.1.15
3.5.1.8
Aspartate
Alanine, aspartate and glutamate metabolism
6.3.4.8
1-(5-Phosphoribosyl)-imidazole-4-acetate
(1-Ribosylimidazole)-4-acetate
2.1.1.8
N-Methylhistamine
1.4.3.4
Methylimidazole acetaldehyde
1.2.1.5
Methylimidazole-acetic acid
2.6.1.38
Imidazole-pyruvate
Imidazole-lactate
2.1.1.44
Hercynine
1.14.99.50
γ-L-Glutamyl-S-(hercyn-2-yl)-L-cysteine S-oxide
3.5.1.118
S-(Hercyn-2-yl)-L-cysteine S-oxide
EgtE
Ergothioneine
1.14.99.51
Thiourocanic acid
4.3.1.3
Urocanate
1.3.99.33
Dihydrourocanate
4.2.1.49
4-Imidazolone-5-propanoate
1.14.13.-
Hydantoin-5-propionate
4-Oxoglutaramate
2-Oxoglutarate
Formylisoglutamine
Isoglutamine
3.5.2.7
N-Formimino-L-glutamate
3.5.3.13
N-Formyl-L-glutamate
2.1.2.5
3.5.3.8
3.5.1.68
N-Carbamyl-L-glutamate
L-Glutamate
Alanine, aspartate and glutamate metabolism
00340 1/22/16
(c) Kanehisa Laboratories

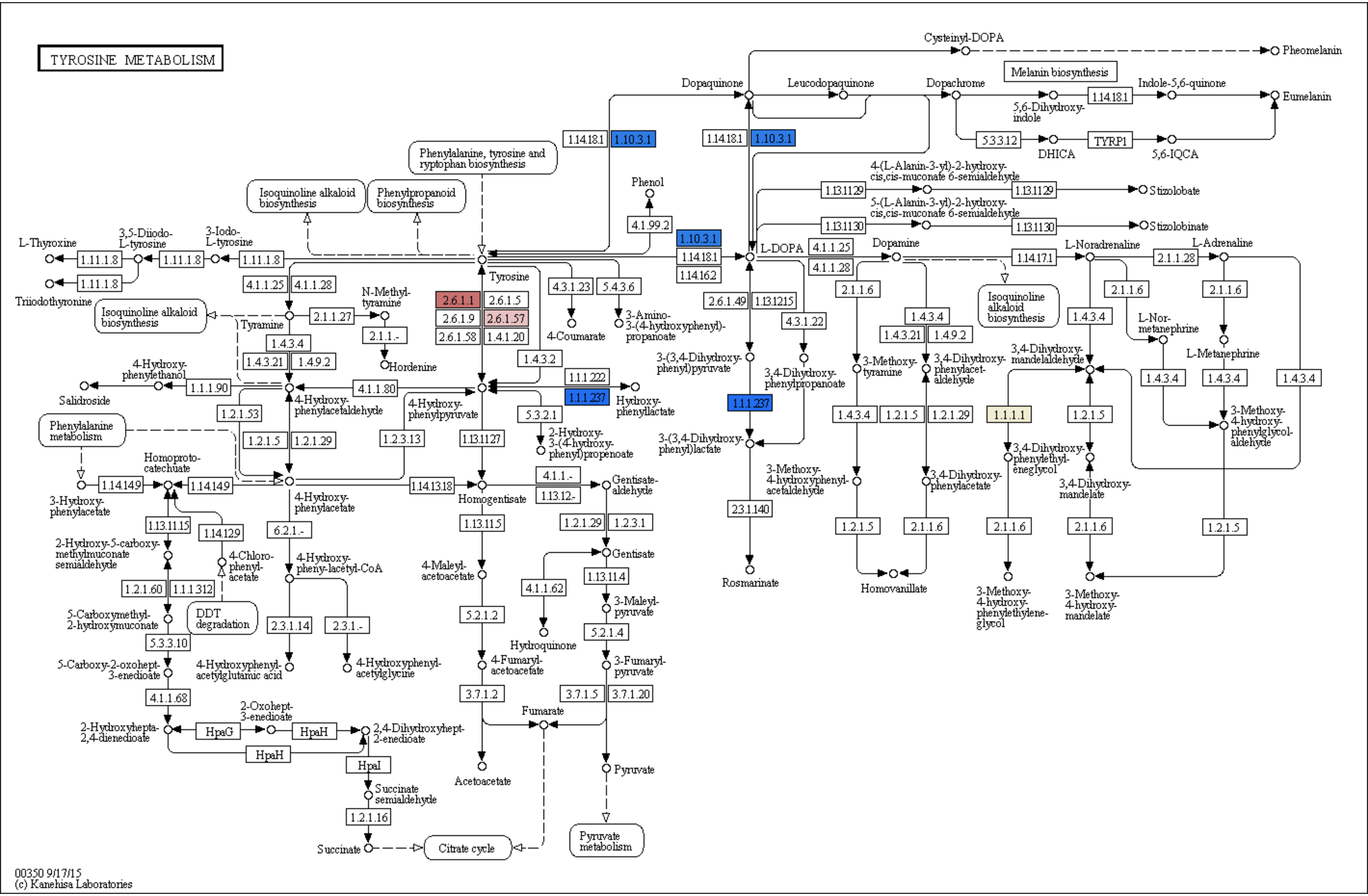
TYROSINE METABOLISM
Cysteinyl-DOPA
Pheomelanin
Melanin biosynthesis
Dopaquinone
Leucodopaquinone
Dopachrome
Indole-5,6-quinone
Eumelanin
5,6-Dihydroxy-indole
1.14.18.1
5.3.3.12
TYRP1
DHICA
5,6-IQCA
1.14.18.1
1.10.3.1
1.14.18.1
1.10.3.1
Phenylalanine, tyrosine and ryptophan biosynthesis
Isoquinoline alkaloid biosynthesis
Phenylpropanoid biosynthesis
Phenol
4.1.99.2
1.10.3.1
1.14.18.1
1.14.16.2
L-DOPA
4.1.1.25
4.1.1.28
Dopamine
1.14.17.1
L-Noradrenaline
2.1.1.28
L-Adrenaline
4-(L-Alanin-3-yl)-2-hydroxy-cis,cis-muconate 6-semialdehyde
1.13.11.29
1.13.11.29
Stizolobate
5-(L-Alanin-3-yl)-2-hydroxy-cis,cis-muconate 6-semialdehyde
1.13.11.30
1.13.11.30
Stizolobinate
L-Thyroxine
3,5-Diiodo-L-tyrosine
3-Iodo-L-tyrosine
1.11.1.8
1.11.1.8
1.11.1.8
1.11.1.8
Triiodothyronine
Tyrosine
4.1.1.25
4.1.1.28
N-Methyl-tyramine
2.6.1.1
2.6.1.5
2.6.1.9
2.6.1.57
2.6.1.58
1.4.1.20
4.3.1.23
5.4.3.6
4-Coumarate
3-Amino-3-(4-hydroxyphenyl)-propanoate
Isoquinoline alkaloid biosynthesis
Tyramine
2.1.1.27
2.1.1.-
Hordenine
1.4.3.4
1.4.3.21
1.4.9.2
1.4.3.2
4-Hydroxy-phenylethanol
1.1.1.90
Salidroside
4.1.1.80
4-Hydroxy-phenylacetaldehyde
4-Hydroxy-phenylpyruvate
1.1.1.222
1.1.1.237
Hydroxy-phenyllactate
5.3.2.1
2-Hydroxy-3-(4-hydroxy-phenyl)propenoate
Phenylalanine metabolism
1.2.1.53
1.2.1.5
1.2.1.29
1.2.3.13
1.13.11.27
Homoproto-catechuate
3-Hydroxy-phenylacetate
1.14.14.9
1.14.14.9
1.14.13.18
Homogentisate
4.1.1.-
1.13.12.-
Gentisate-aldehyde
4-Hydroxy-phenylacetate
1.13.11.15
1.14.12.9
6.2.1.-
1.13.11.5
1.2.1.29
1.2.3.1
Gentisate
2-Hydroxy-5-carboxy-methylmuconate semialdehyde
4-Chloro-phenyl-acetate
4-Hydroxy-pheny-lacetyl-CoA
4-Maleyl-acetoacetate
4.1.1.62
1.13.11.4
1.2.1.60
1.1.1.312
DDT degradation
3-Maleyl-pyruvate
5-Carboxymethyl-2-hydroxymuconate
2.3.1.14
2.3.1.-
5.2.1.2
Hydroquinone
5.2.1.4
5.3.3.10
5-Carboxy-2-oxohept-3-enedioate
4-Hydroxyphenyl-acetylglutamic acid
4-Hydroxyphenyl-acetylglycine
4-Fumaryl-acetoacetate
3-Fumaryl-pyruvate
4.1.1.68
3.7.1.2
3.7.1.5
3.7.1.20
2-Oxohept-3-enedioate
Fumarate
2-Hydroxyhepta-2,4-dienedioate
HpaG
HpaH
2,4-Dihydroxyhept-2-enedioate
HpaH
HpaI
Acetoacetate
Pyruvate
Succinate semialdehyde
1.2.1.16
Succinate
Citrate cycle
Pyruvate metabolism
2.6.1.49
1.13.11.5
3-(3,4-Dihydroxy-phenyl)pyruvate
4.3.1.22
3,4-Dihydroxy-phenylpropanoate
1.1.1.237
3-(3,4-Dihydroxy-phenyl)lactate
3-Methoxy-4-hydroxyphenyl-acetaldehyde
2.3.1.140
Rosmarinate
Isoquinoline alkaloid biosynthesis
2.1.1.6
1.4.3.4
1.4.3.21
1.4.9.2
3-Methoxy-tyramine
3,4-Dihydroxy-phenylacet-aldehyde
1.4.3.4
1.2.1.5
1.2.1.29
3,4-Dihydroxyphenylacetate
1.2.1.5
2.1.1.6
Homovanillate
2.1.1.6
1.4.3.4
L-Nor-metanephrine
2.1.1.6
L-Metanephrine
3,4-Dihydroxy-mandelaldehyde
1.4.3.4
1.4.3.4
1.4.3.4
1.1.1.1
1.2.1.5
3-Methoxy-4-hydroxy-phenylglycol-aldehyde
3,4-Dihydroxy-phenylethyl-eneglycol
3,4-Dihydroxy-mandelate
2.1.1.6
2.1.1.6
1.2.1.5
3-Methoxy-4-hydroxy-phenylethylene-glycol
3-Methoxy-4-hydroxy-mandelate
00350 9/17/15
(c) Kanehisa Laboratories

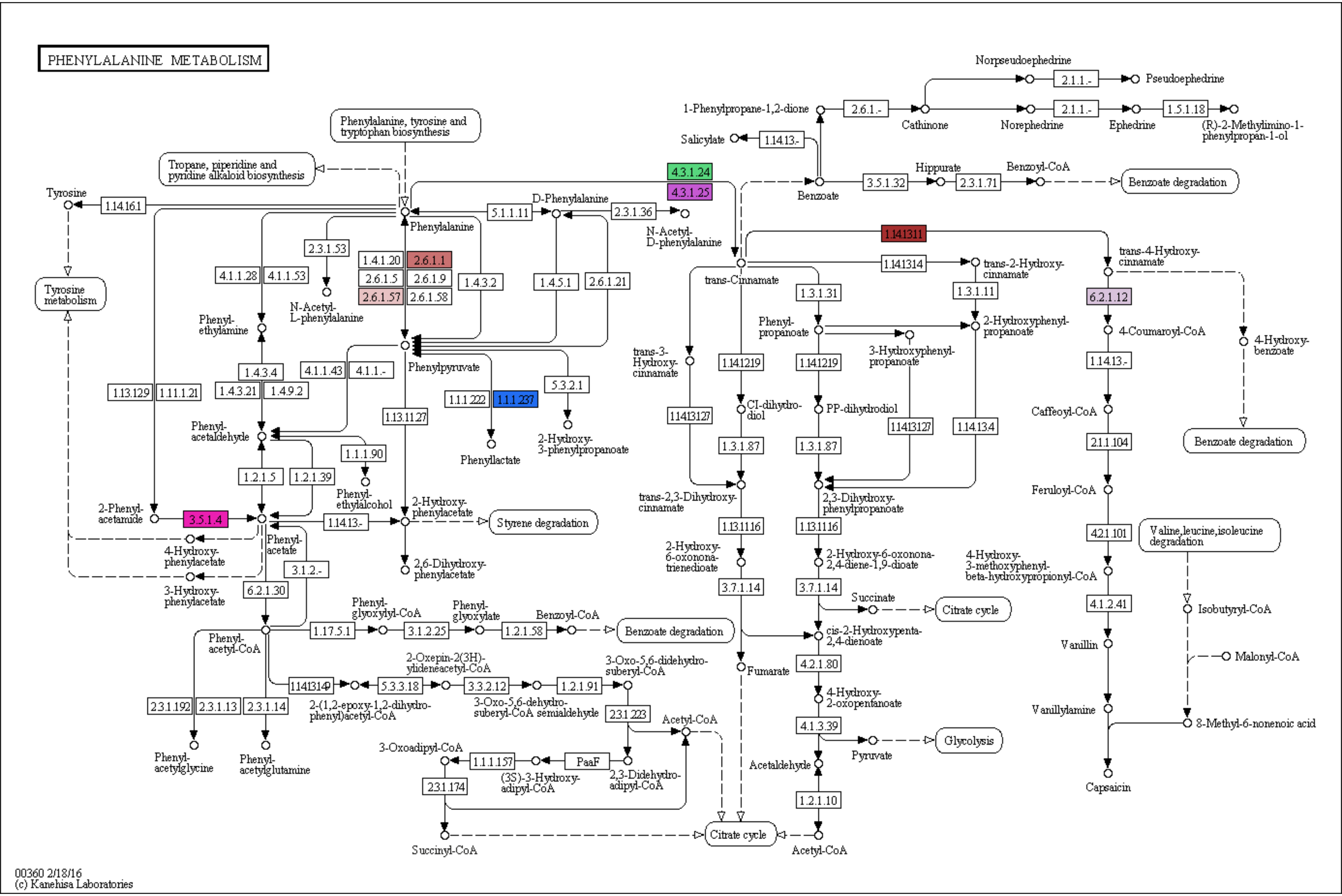

PHENYLALANINE METABOLISM
Phenylalanine, tyrosine and tryptophan biosynthesis
Tropane, piperidine and pyridine alkaloid biosynthesis
Tyrosine
1.14.16.1
Tyrosine metabolism
Phenylalanine
D-Phenylalanine
5.1.1.11
2.3.1.36
N-Acetyl-D-phenylalanine
4.3.1.24
4.3.1.25
2.3.1.53
N-Acetyl-L-phenylalanine
1.4.1.20
2.6.1.1
2.6.1.5
2.6.1.9
2.6.1.57
2.6.1.58
1.4.3.2
1.4.5.1
2.6.1.21
4.1.1.28
4.1.1.53
Phenyl-ethylamine
1.4.3.4
1.4.3.21
1.4.9.2
4.1.1.43
4.1.1.-
Phenylpyruvate
1.13.12.9
1.11.1.21
1.1.1.222
1.1.1.237
5.3.2.1
1.13.11.27
Phenyl-acetaldehyde
1.1.1.90
Phenyllactate
2-Hydroxy-3-phenylpropanoate
1.2.1.5
1.2.1.39
Phenyl-ethylalcohol
2-Phenyl-acetamide
3.5.1.4
1.14.13.-
2-Hydroxy-phenylacetate
Styrene degradation
Phenyl-acetate
4-Hydroxy-phenylacetate
3.1.2.-
2,6-Dihydroxy-phenylacetate
3-Hydroxy-phenylacetate
6.2.1.30
Phenyl-glyoxylyl-CoA
Phenyl-glyoxylate
Benzoyl-CoA
Phenyl-acetyl-CoA
1.17.5.1
3.1.2.25
1.2.1.58
Benzoate degradation
2-Oxepin-2(3H)-ylideneacetyl-CoA
3-Oxo-5,6-didehydro-suberyl-CoA
1.14.13.149
5.3.3.18
3.3.2.12
1.2.1.91
2-(1,2-epoxy-1,2-dihydro-phenyl)acetyl-CoA
3-Oxo-5,6-dehydro-suberyl-CoA semialdehyde
2.3.1.223
Acetyl-CoA
2.3.1.192
2.3.1.13
2.3.1.14
Phenyl-acetylglycine
Phenyl-acetylglutamine
3-Oxoadipyl-CoA
1.1.1.157
PaaF
(3S)-3-Hydroxy-adipyl-CoA
2,3-Didehydro-adipyl-CoA
2.3.1.174
Succinyl-CoA
Citrate cycle
Acetyl-CoA
Norpseudoephedrine
2.1.1.-
Pseudoephedrine
1-Phenylpropane-1,2-dione
2.6.1.-
Cathinone
Norephedrine
2.1.1.-
Ephedrine
1.5.1.18
(R)-2-Methylimino-1-phenylpropan-1-ol
Salicylate
1.14.13.-
Benzoate
3.5.1.32
Hippurate
2.3.1.71
Benzoyl-CoA
Benzoate degradation
1.14.13.11
trans-Cinnamate
1.14.13.14
trans-2-Hydroxy-cinnamate
trans-4-Hydroxy-cinnamate
1.3.1.31
1.3.1.11
6.2.1.12
Phenyl-propanoate
2-Hydroxyphenyl-propanoate
3-Hydroxyphenyl-propanoate
4-Coumaroyl-CoA
4-Hydroxy-benzoate
trans-3-Hydroxy-cinnamate
1.14.12.19
1.14.12.19
1.14.13.-
CI-dihydro-diol
PP-dihydrodiol
Caffeoyl-CoA
1.14.13.127
1.14.13.127
1.14.13.4
Benzoate degradation
1.3.1.87
1.3.1.87
2.1.1.104
trans-2,3-Dihydroxy-cinnamate
2,3-Dihydroxy-phenylpropanoate
Feruloyl-CoA
1.13.11.16
1.13.11.16
4.2.1.101
Valine, leucine, isoleucine degradation
2-Hydroxy-6-oxonona-trienedioate
2-Hydroxy-6-oxonona-2,4-diene-1,9-dioate
4-Hydroxy-3-methoxyphenyl-beta-hydroxypropionyl-CoA
3.7.1.14
3.7.1.14
Succinate
Citrate cycle
4.1.2.41
Isobutyryl-CoA
cis-2-Hydroxypenta-2,4-dienoate
Vanillin
Fumarate
Malonyl-CoA
4.2.1.80
4-Hydroxy-2-oxopentanoate
Vanillylamine
8-Methyl-6-nonenoic acid
4.1.3.39
Pyruvate
Glycolysis
Acetaldehyde
Capsaicin
1.2.1.10
Citrate cycle
Acetyl-CoA
00360 2/18/16
(c) Kanehisa Laboratories

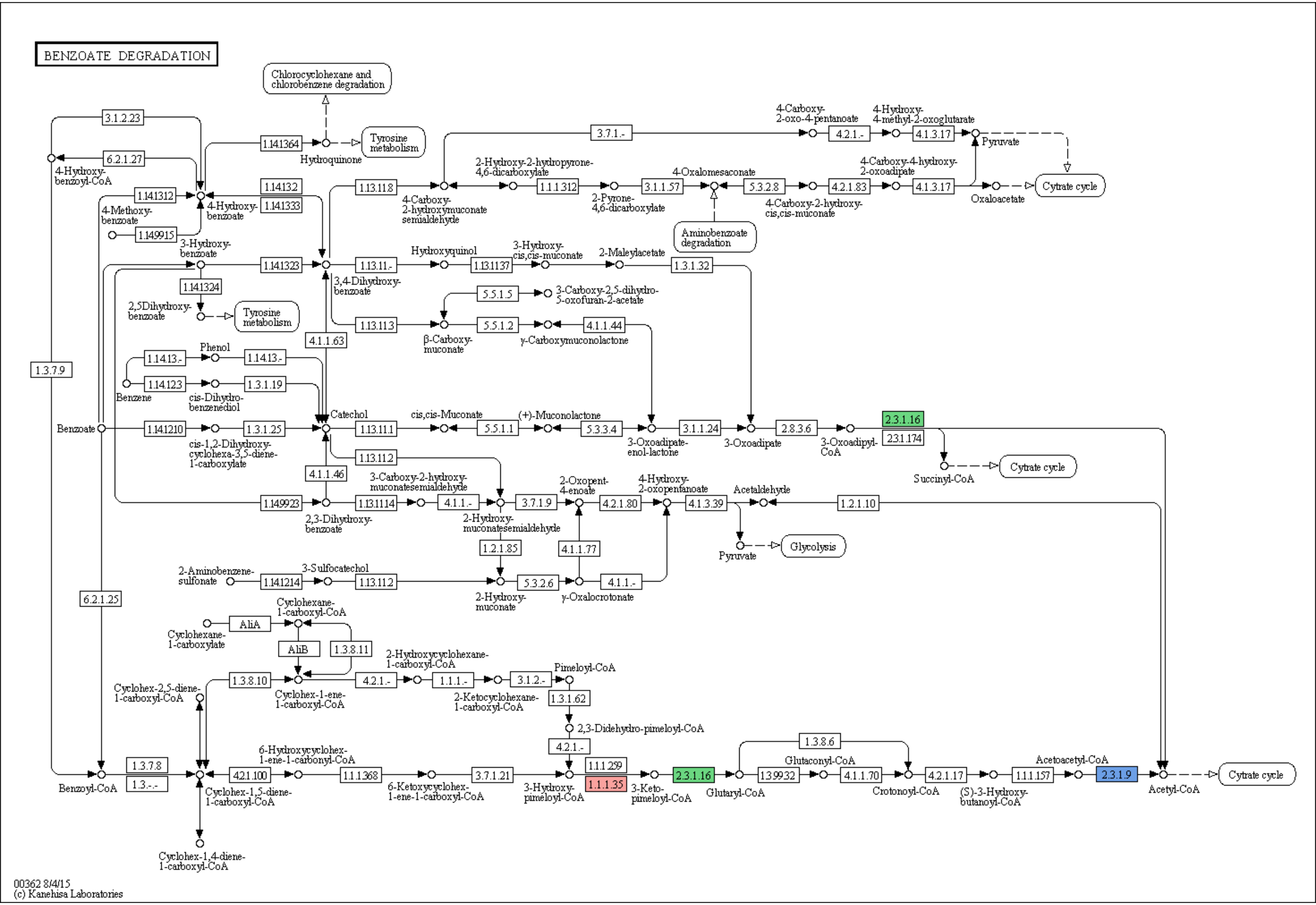
BENZOATE DEGRADATION
Chlorocyclohexane and chlorobenzene degradation
Tyrosine metabolism
Hydroquinone
3.1.2.23
6.2.1.27
4-Hydroxy-benzoyl-CoA
1.14.13.64
1.14.13.12
1.14.13.2
1.14.13.33
4-Methoxy-benzoate
4-Hydroxy-benzoate
1.14.99.15
3-Hydroxy-benzoate
1.14.13.23
1.14.13.24
2,5Dihydroxy-benzoate
Tyrosine metabolism
3.7.1.-
4-Carboxy-2-oxo-4-pentanoate
4.2.1.-
4-Hydroxy-4-methyl-2-oxoglutarate
4.1.3.17
Pyruvate
2-Hydroxy-2-hydropyrone-4,6-dicarboxylate
4-Oxalomesaconate
4-Carboxy-4-hydroxy-2-oxoadipate
1.13.11.8
1.1.1.312
3.1.1.57
5.3.2.8
4.2.1.83
4.1.3.17
Cytrate cycle
4-Carboxy-2-hydroxymuconate semialdehyde
2-Pyrone-4,6-dicarboxylate
4-Carboxy-2-hydroxy-cis,cis-muconate
Oxaloacetate
Aminobenzoate degradation
Hydroxyquinol
3-Hydroxy-cis,cis-muconate
2-Maleylacetate
1.13.11.-
1.13.11.37
1.3.1.32
3,4-Dihydroxy-benzoate
5.5.1.5
3-Carboxy-2,5-dihydro-5-oxofuran-2-acetate
1.13.11.3
5.5.1.2
4.1.1.44
β-Carboxy-muconate
γ-Carboxymuconolactone
4.1.1.63
Phenol
1.14.13.-
1.14.13.-
1.14.12.3
1.3.1.19
Benzene
cis-Dihydro-benzenediol
Catechol
1.3.7.9
Benzoate
1.14.12.10
1.3.1.25
cis-1,2-Dihydroxy-cyclohexa-3,5-diene-1-carboxylate
1.13.11.1
cis,cis-Muconate
5.5.1.1
(+)-Muconolactone
5.3.3.4
3.1.1.24
2.8.3.6
2.3.1.16
2.3.1.174
3-Oxoadipate-enol-lactone
3-Oxoadipate
3-Oxoadipyl-CoA
Succinyl-CoA
Cytrate cycle
1.13.11.2
4.1.1.46
3-Carboxy-2-hydroxy-muconatesemialdehyde
2-Oxopent-4-enoate
4-Hydroxy-2-oxopentanoate
Acetaldehyde
1.14.99.23
1.13.11.14
4.1.1.-
3.7.1.9
4.2.1.80
4.1.3.39
1.2.1.10
2,3-Dihydroxy-benzoate
2-Hydroxy-muconatesemialdehyde
Pyruvate
Glycolysis
1.2.1.85
4.1.1.77
2-Aminobenzene-sulfonate
3-Sulfocatechol
1.14.12.14
1.13.11.2
5.3.2.6
4.1.1.-
2-Hydroxy-muconate
γ-Oxalocrotonate
6.2.1.25
Cyclohexane-1-carboxyl-CoA
AliA
Cyclohexane-1-carboxylate
AliB
1.3.8.11
2-Hydroxycyclohexane-1-carboxyl-CoA
Cyclohex-2,5-diene-1-carboxyl-CoA
1.3.8.10
4.2.1.-
1.1.1.-
3.1.2.-
Pimeloyl-CoA
Cyclohex-1-ene-1-carboxyl-CoA
2-Ketocyclohexane-1-carboxyl-CoA
1.3.1.62
2,3-Didehydro-pimeloyl-CoA
4.2.1.-
1.3.8.6
6-Hydroxycyclohex-1-ene-1-carbonyl-CoA
Glutaconyl-CoA
Acetoacetyl-CoA
1.3.7.8
1.3.-.-
4.2.1.100
1.1.1.368
3.7.1.21
1.1.1.259
1.1.1.35
2.3.1.16
1.3.99.32
4.1.1.70
4.2.1.17
1.1.1.157
2.3.1.9
Cytrate cycle
Benzoyl-CoA
Cyclohex-1,5-diene-1-carboxyl-CoA
6-Ketoxycyclohex-1-ene-1-carboxyl-CoA
3-Hydroxy-pimeloyl-CoA
3-Keto-pimeloyl-CoA
Glutaryl-CoA
Crotonoyl-CoA
(S)-3-Hydroxy-butanoyl-CoA
Acetyl-CoA
Cyclohex-1,4-diene-1-carboxyl-CoA
00362 8/4/15
(c) Kanehisa Laboratories

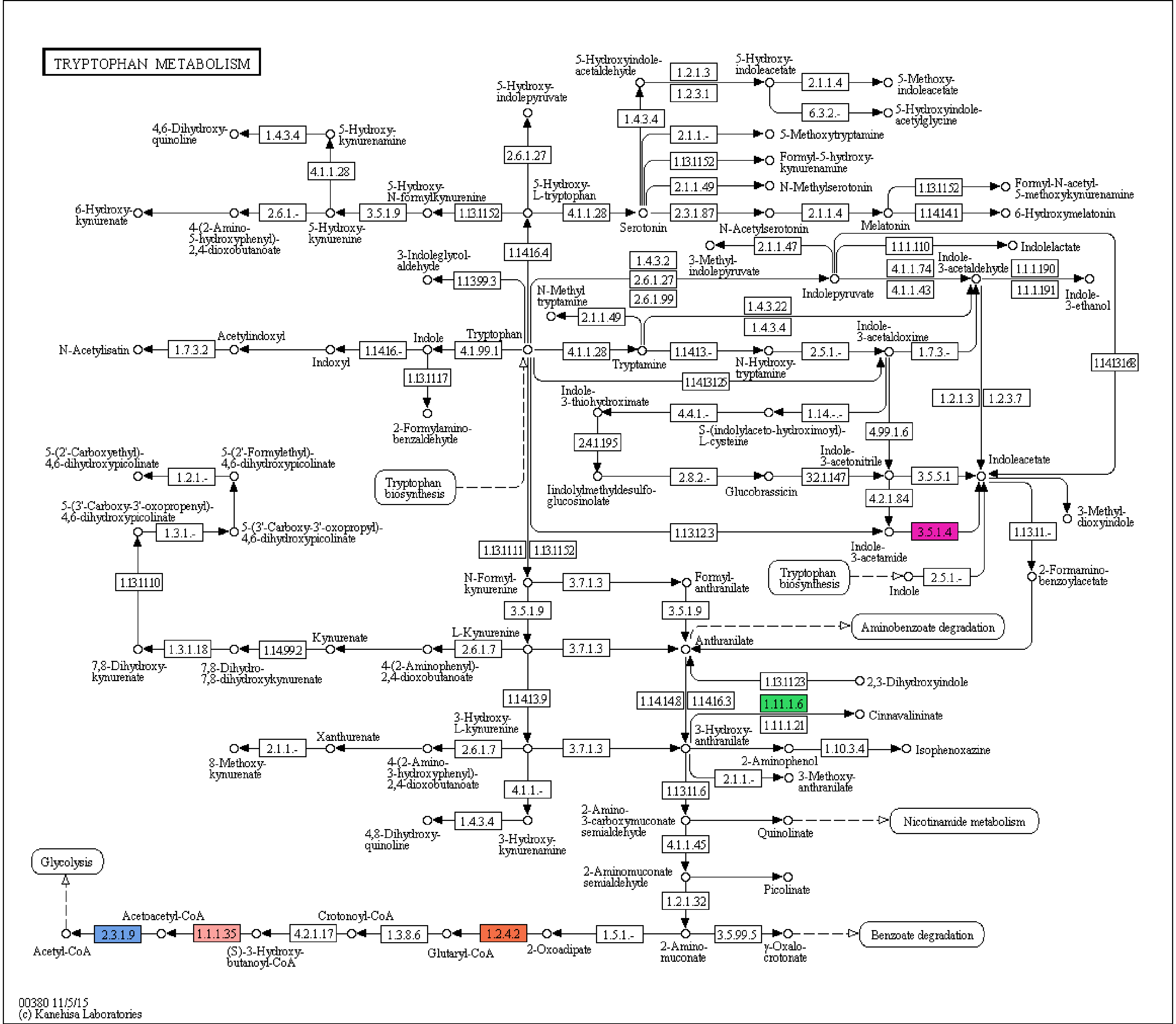
TRYPTOPHAN METABOLISM
5-Hydroxyindole-acetaldehyde
1.2.1.3
1.2.3.1
5-Hydroxy-indoleacetate
2.1.1.4
5-Methoxy-indoleacetate
6.3.2.-
5-Hydroxyindole-acetylglycine
5-Hydroxy-indolepyruvate
1.4.3.4
2.1.1.-
5-Methoxytryptamine
1.13.11.52
Formyl-5-hydroxy-kynurenamine
2.1.1.49
N-Methylserotonin
2.3.1.87
2.1.1.4
Melatonin
1.13.11.52
Formyl-N-acetyl-5-methoxykynurenamine
1.14.14.1
6-Hydroxymelatonin
4,6-Dihydroxy-quinoline
1.4.3.4
5-Hydroxy-kynurenamine
4.1.1.28
2.6.1.27
5-Hydroxy-N-formylkynurenine
5-Hydroxy-L-tryptophan
6-Hydroxy-kynurenate
2.6.1.-
4-(2-Amino-5-hydroxyphenyl)-2,4-dioxobutanoate
3.5.1.9
5-Hydroxy-kynurenine
1.13.11.52
4.1.1.28
Serotonin
N-Acetylserotonin
1.14.16.4
3-Indoleglycol-aldehyde
1.13.99.3
2.1.1.47
3-Methyl-indolepyruvate
1.4.3.2
2.6.1.27
2.6.1.99
N-Methyl tryptamine
2.1.1.49
1.4.3.22
1.4.3.4
Indolepyruvate
1.1.1.110
Indolelactate
4.1.1.74
4.1.1.43
Indole-3-acetaldehyde
1.1.1.190
1.1.1.191
Indole-3-ethanol
N-Acetylisatin
1.7.3.2
Acetylindoxyl
Indoxyl
1.14.16.-
Indole
Tryptophan
4.1.99.1
4.1.1.28
Tryptamine
1.14.13.-
N-Hydroxy-tryptamine
2.5.1.-
Indole-3-acetaldoxime
1.7.3.-
1.14.13.125
1.14.13.168
1.13.11.17
2-Formylamino-benzaldehyde
Indole-3-thiohydroximate
4.4.1.-
S-(indolylaceto-hydroximoyl)-L-cysteine
1.14.-.-
1.2.1.3
1.2.3.7
2.4.1.195
4.99.1.6
Indole-3-acetonitrile
5-(2'-Carboxyethyl)-4,6-dihydroxypicolinate
1.2.1.-
5-(2'-Formylethyl)-4,6-dihydroxypicolinate
Tryptophan biosynthesis
Indolylmethyldesulfo-glucosinolate
2.8.2.-
Glucobrassicin
3.2.1.147
3.5.5.1
Indoleacetate
4.2.1.84
5-(3'-Carboxy-3'-oxopropenyl)-4,6-dihydroxypicolinate
1.3.1.-
5-(3'-Carboxy-3'-oxopropyl)-4,6-dihydroxypicolinate
1.13.12.3
3.5.1.4
3-Methyl-dioxyindole
Indole-3-acetamide
1.13.11.-
1.13.11.11
1.13.11.52
1.13.11.10
N-Formyl-kynurenine
3.7.1.3
Formyl-anthranilate
Tryptophan biosynthesis
Indole
2.5.1.-
2-Formamino-benzoylacetate
3.5.1.9
3.5.1.9
Aminobenzoate degradation
Kynurenate
L-Kynurenine
Anthranilate
7,8-Dihydroxy-kynurenate
1.3.1.18
7,8-Dihydro-7,8-dihydroxykynurenate
1.14.99.2
4-(2-Aminophenyl)-2,4-dioxobutanoate
2.6.1.7
3.7.1.3
1.13.11.23
2,3-Dihydroxyindole
1.14.13.9
1.14.14.8
1.14.16.3
1.11.1.6
Cinnavalininate
1.11.1.21
3-Hydroxy-L-kynurenine
3-Hydroxy-anthranilate
8-Methoxy-kynurenate
2.1.1.-
Xanthurenate
4-(2-Amino-3-hydroxyphenyl)-2,4-dioxobutanoate
2.6.1.7
3.7.1.3
2-Aminophenol
1.10.3.4
Isophenoxazine
2.1.1.-
3-Methoxy-anthranilate
4.1.1.-
1.13.11.6
4,8-Dihydroxy-quinoline
1.4.3.4
3-Hydroxy-kynurenamine
2-Amino-3-carboxymuconate semialdehyde
Quinolinate
Nicotinamide metabolism
4.1.1.45
Glycolysis
2-Aminomuconate semialdehyde
Picolinate
1.2.1.32
Acetoacetyl-CoA
Crotonoyl-CoA
Acetyl-CoA
2.3.1.9
1.1.1.35
(S)-3-Hydroxy-butanoyl-CoA
4.2.1.17
1.3.8.6
Glutaryl-CoA
1.2.4.2
2-Oxoadipate
1.5.1.-
2-Amino-muconate
3.5.99.5
γ-Oxalo-crotonate
Benzoate degradation
00380 11/5/15
(c) Kanehisa Laboratories

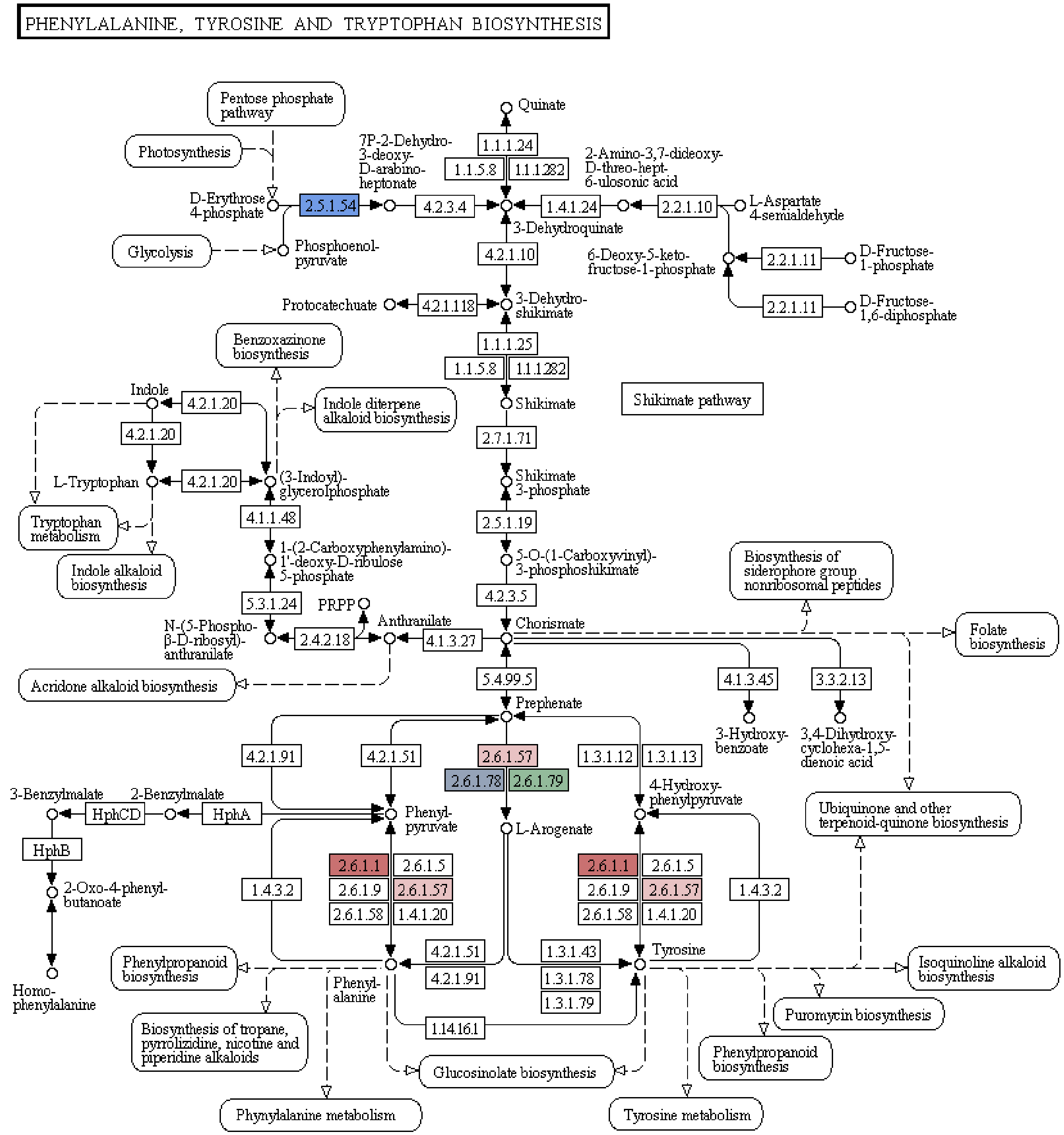

00400 7/3/14
(c) Kanehisa Laboratories

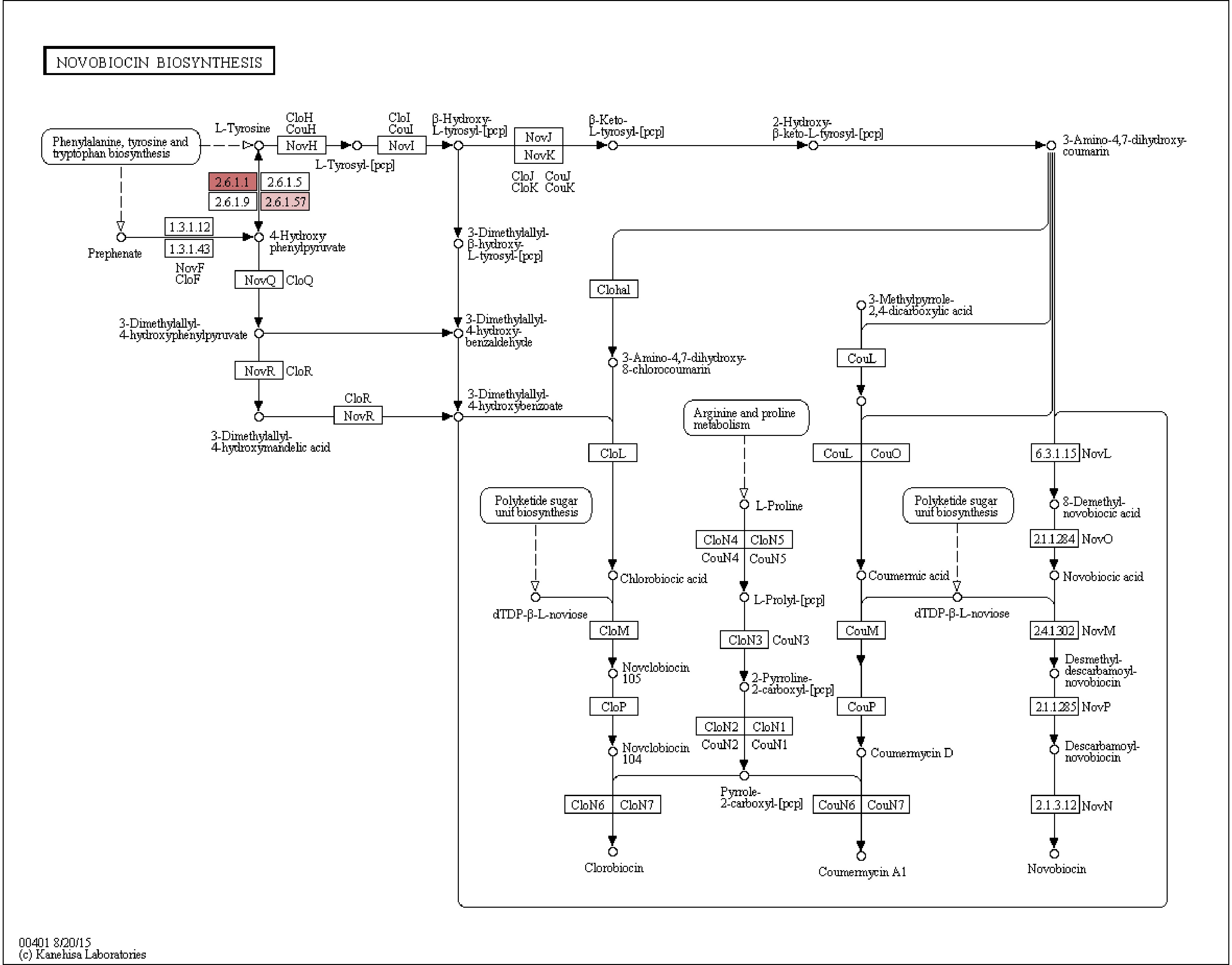
NOVOBIOCIN BIOSYNTHESIS
Phenylalanine, tyrosine and tryptophan biosynthesis
L-Tyrosine
CloH
CouH
NovH
L-Tyrosyl-[pcp]
CloI
CouI
NovI
β-Hydroxy-L-tyrosyl-[pcp]
NovJ
NovK
CloJ CouJ
CloK CouK
β-Keto-L-tyrosyl-[pcp]
2-Hydroxy-β-keto-L-tyrosyl-[pcp]
3-Amino-4,7-dihydroxy-coumarin
2.6.1.1
2.6.1.5
2.6.1.9
2.6.1.57
1.3.1.12
1.3.1.43
NovF
CloF
Prephenate
4-Hydroxy phenylpyruvate
NovQ CloQ
3-Dimethylallyl-β-hydroxy-L-tyrosyl-[pcp]
3-Dimethylallyl-4-hydroxyphenylpyruvate
3-Dimethylallyl-4-hydroxy-benzaldehyde
NovR CloR
CloR
NovR
3-Dimethylallyl-4-hydroxybenzoate
3-Dimethylallyl-4-hydroxymandelic acid
Clohal
3-Amino-4,7-dihydroxy-8-chlorocoumarin
3-Methylpyrrole-2,4-dicarboxylic acid
CouL
Arginine and proline metabolism
CloL
CouL CouO
6.3.1.15 NovL
Polyketide sugar unit biosynthesis
L-Proline
Polyketide sugar unit biosynthesis
8-Demethyl-novobiocic acid
CloN4 CloN5
CouN4 CouN5
2.1.1.284 NovO
Chlorobiocic acid
Coumermic acid
Novobiocic acid
L-Prolyl-[pcp]
dTDP-β-L-noviose
dTDP-β-L-noviose
CloM
CouM
2.4.1.302 NovM
CloN3 CouN3
Novclobiocin 105
Desmethyl-descarbamoyl-novobiocin
2-Pyrroline-2-carboxyl-[pcp]
CloP
CouP
2.1.1.285 NovP
CloN2 CloN1
CouN2 CouN1
Novclobiocin 104
Coumermycin D
Descarbamoyl-novobiocin
Pyrrole-2-carboxyl-[pcp]
CloN6 CloN7
CouN6 CouN7
2.1.3.12 NovN
Clorobiocin
Coumermycin A1
Novobiocin
00401 8/20/15
(c) Kanehisa Laboratories

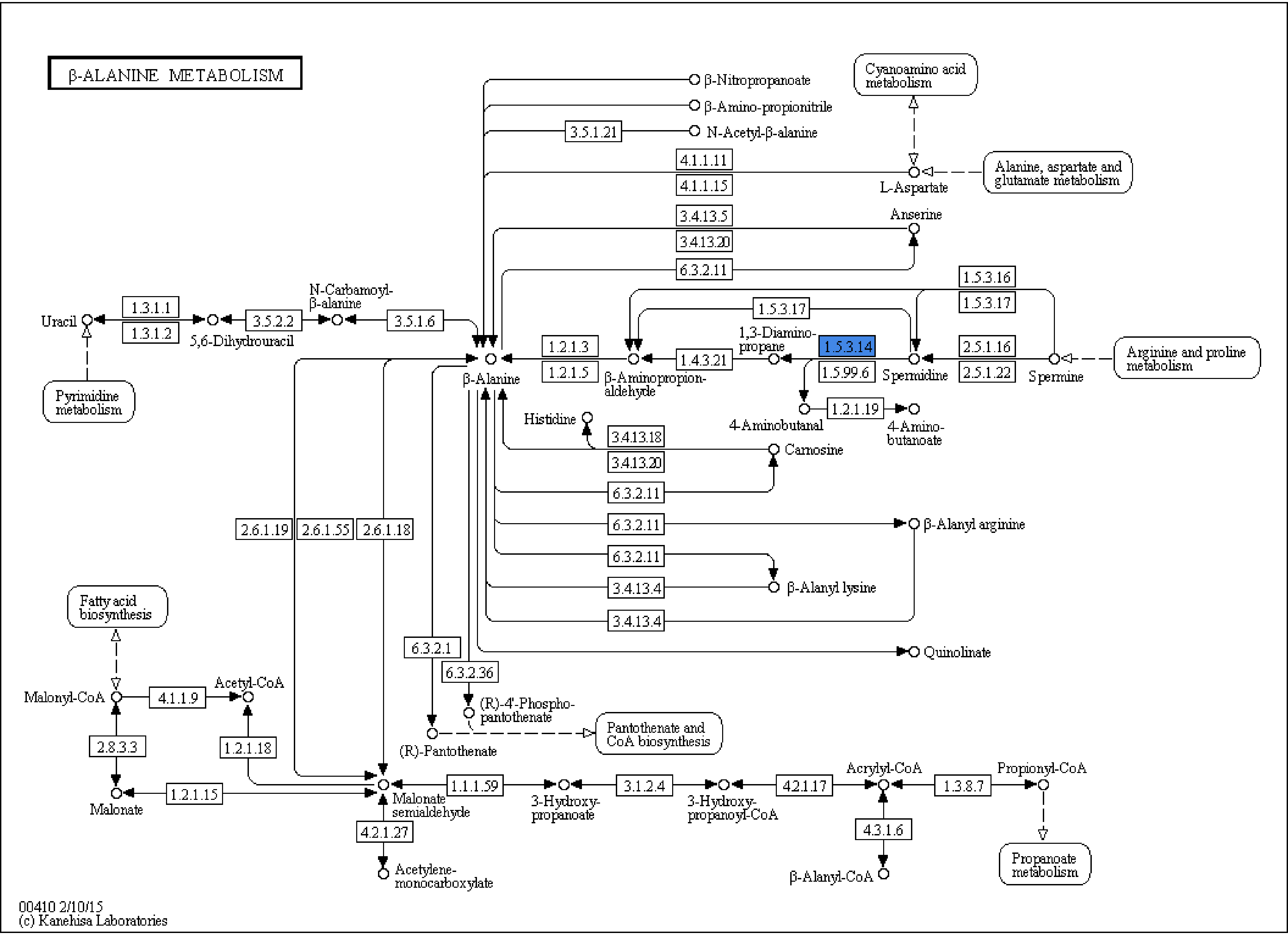
β-ALANINE METABOLISM
β-Nitropropanoate
β-Amino-propionitrile
3.5.1.21
N-Acetyl-β-alanine
Cyanoamino acid metabolism
4.1.1.11
4.1.1.15
L-Aspartate
Alanine, aspartate and glutamate metabolism
3.4.13.5
3.4.13.20
Anserine
6.3.2.11
1.5.3.16
1.5.3.17
1.5.3.17
Uracil
1.3.1.1
1.3.1.2
5,6-Dihydrouracil
3.5.2.2
N-Carbamoyl-β-alanine
3.5.1.6
1.2.1.3
1.2.1.5
1,3-Diamino-propane
1.5.3.14
1.5.99.6
2.5.1.16
2.5.1.22
1.4.3.21
β-Alanine
β-Aminopropion-aldehyde
Spermidine
Spermine
Arginine and proline metabolism
Pyrimidine metabolism
4-Aminobutanal
1.2.1.19
4-Amino-butanoate
Histidine
3.4.13.18
3.4.13.20
Carnosine
6.3.2.11
6.3.2.11
β-Alanyl arginine
2.6.1.19
2.6.1.55
2.6.1.18
6.3.2.11
3.4.13.4
β-Alanyl lysine
3.4.13.4
Quinolinate
Fatty acid biosynthesis
6.3.2.1
6.3.2.36
Malonyl-CoA
4.1.1.9
Acetyl-CoA
(R)-4'-Phospho-pantothenate
Pantothenate and CoA biosynthesis
(R)-Pantothenate
2.8.3.3
1.2.1.18
Acrylyl-CoA
Propionyl-CoA
1.1.1.59
3.1.2.4
4.2.1.17
1.3.8.7
Malonate
1.2.1.15
Malonate semialdehyde
3-Hydroxy-propanoate
3-Hydroxy-propanoyl-CoA
4.2.1.27
4.3.1.6
Acetylene-monocarboxylate
β-Alanyl-CoA
Propanoate metabolism
00410 2/10/15
(c) Kanehisa Laboratories

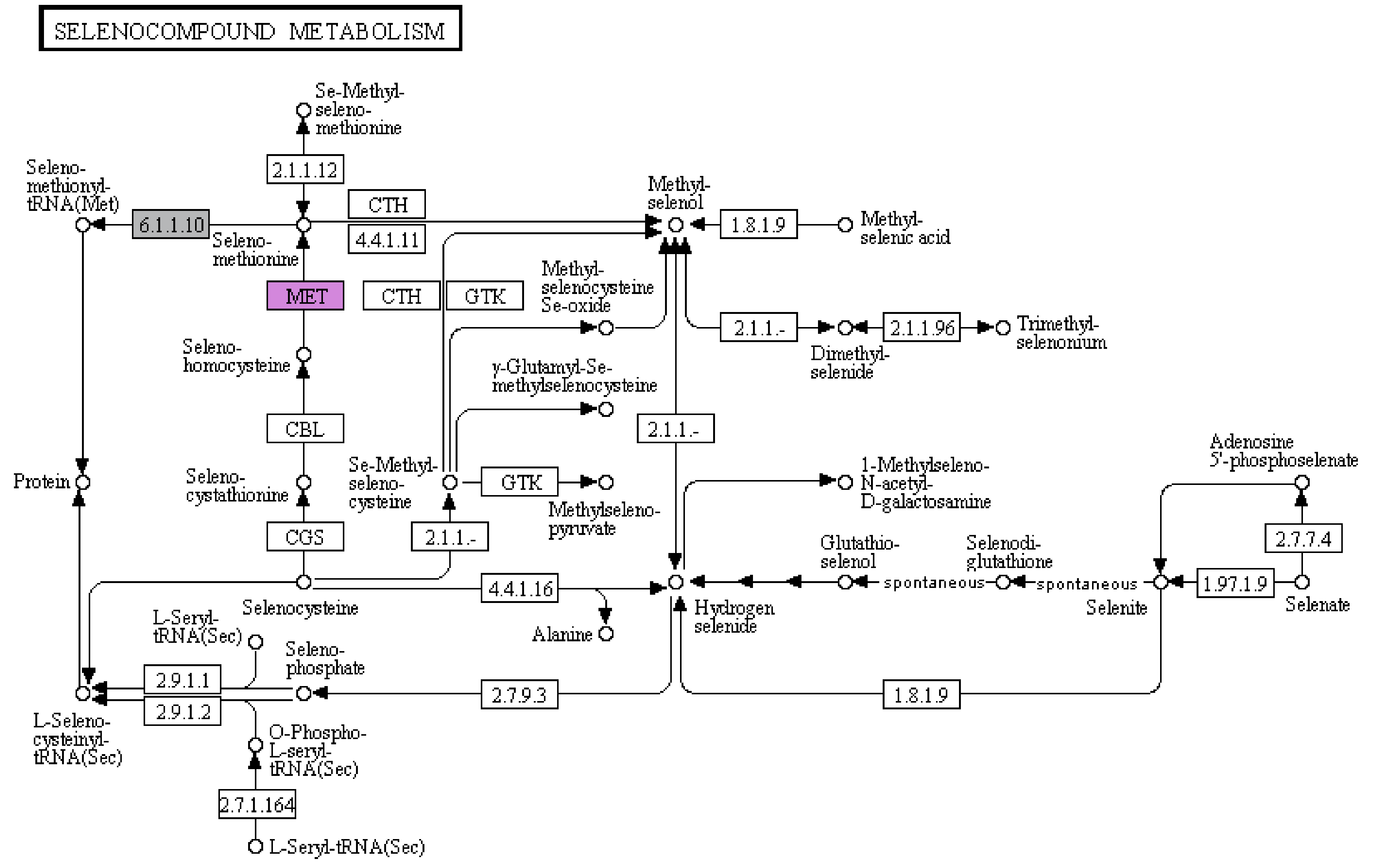

00450 3/3/14

(c) Kanehisa Laboratories

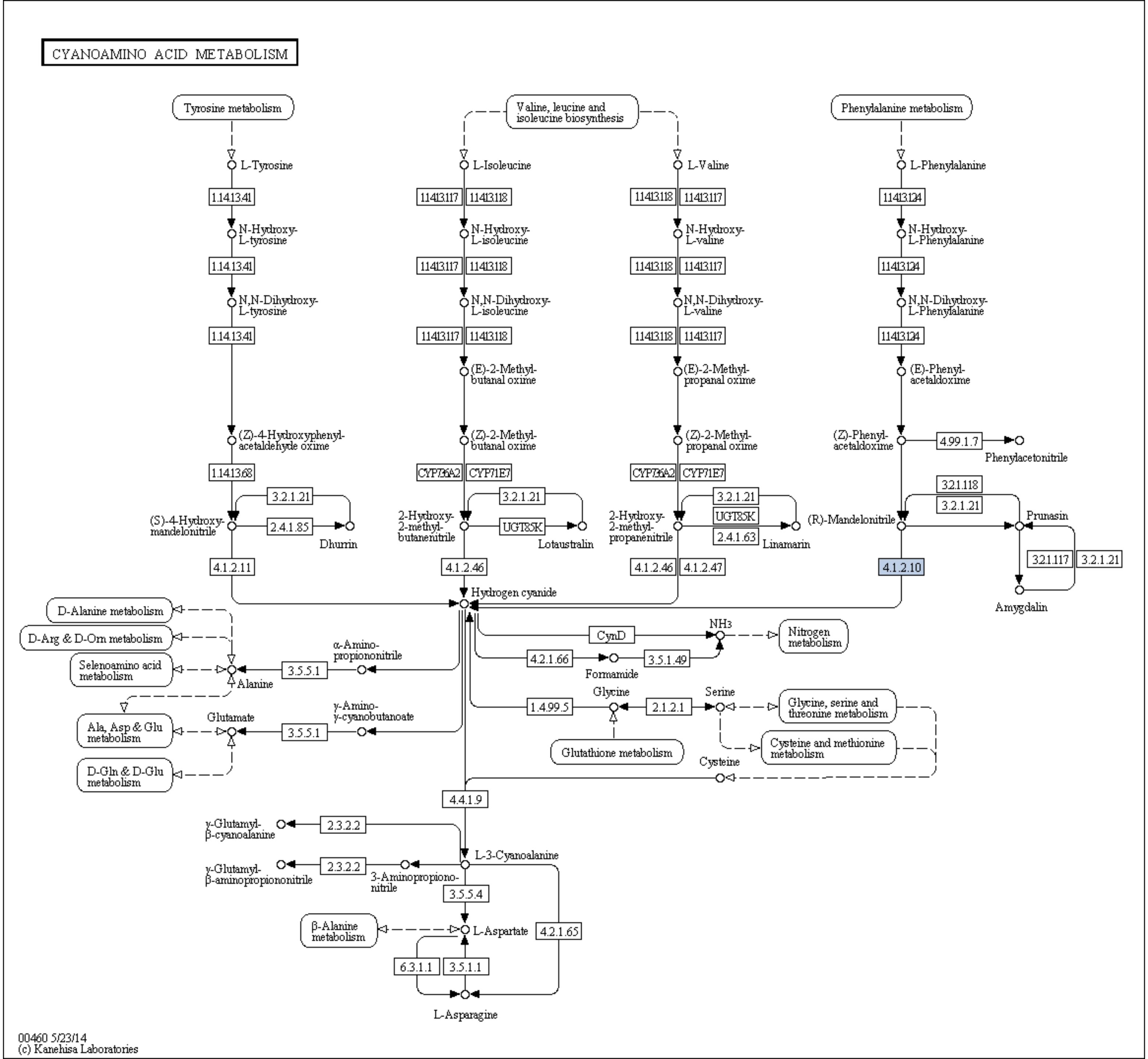
CYANOAMINO ACID METABOLISM
Tyrosine metabolism
Valine, leucine and isoleucine biosynthesis
Phenylalanine metabolism
L-Tyrosine
L-Isoleucine
L-Valine
L-Phenylalanine
1.14.13.41
1.14.13.117
1.14.13.118
1.14.13.124
N-Hydroxy-L-tyrosine
N-Hydroxy-L-isoleucine
N-Hydroxy-L-valine
N-Hydroxy-L-Phenylalanine
N,N-Dihydroxy-L-tyrosine
N,N-Dihydroxy-L-isoleucine
N,N-Dihydroxy-L-valine
N,N-Dihydroxy-L-Phenylalanine
(E)-2-Methyl-butanal oxime
(E)-2-Methyl-propanal oxime
(E)-Phenyl-acetaldoxime
(Z)-4-Hydroxyphenyl-acetaldehyde oxime
(Z)-2-Methyl-butanal oxime
(Z)-2-Methyl-propanal oxime
(Z)-Phenyl-acetaldoxime
4.99.1.7
Phenylacetonitrile
1.14.13.68
CYP736A2
CYP71E7
3.2.1.21
2.4.1.85
UGT85K
2.4.1.63
3.2.1.118
(S)-4-Hydroxy-mandelonitrile
2-Hydroxy-2-methyl-butanenitrile
2-Hydroxy-2-methyl-propanenitrile
(R)-Mandelonitrile
Dhurrin
Lotaustralin
Linamarin
Prunasin
3.2.1.117
Amygdalin
4.1.2.11
4.1.2.46
4.1.2.47
4.1.2.10
Hydrogen cyanide
D-Alanine metabolism
D-Arg & D-Orn metabolism
Selenoamino acid metabolism
Alanine
3.5.5.1
α-Amino-propiononitrile
CynD
NH3
Nitrogen metabolism
4.2.1.66
Formamide
3.5.1.49
Ala, Asp & Glu metabolism
Glutamate
γ-Amino-γ-cyanobutanoate
D-Gln & D-Glu metabolism
1.4.99.5
Glycine
2.1.2.1
Serine
Glycine, serine and threonine metabolism
Glutathione metabolism
Cysteine and methionine metabolism
Cysteine
4.4.1.9
γ-Glutamyl-β-cyanoalanine
2.3.2.2
L-3-Cyanoalanine
γ-Glutamyl-β-aminopropiononitrile
3-Aminopropiono-nitrile
3.5.5.4
β-Alanine metabolism
L-Aspartate
4.2.1.65
6.3.1.1
3.5.1.1
L-Asparagine
00460 5/23/14
(c) Kanehisa Laboratories

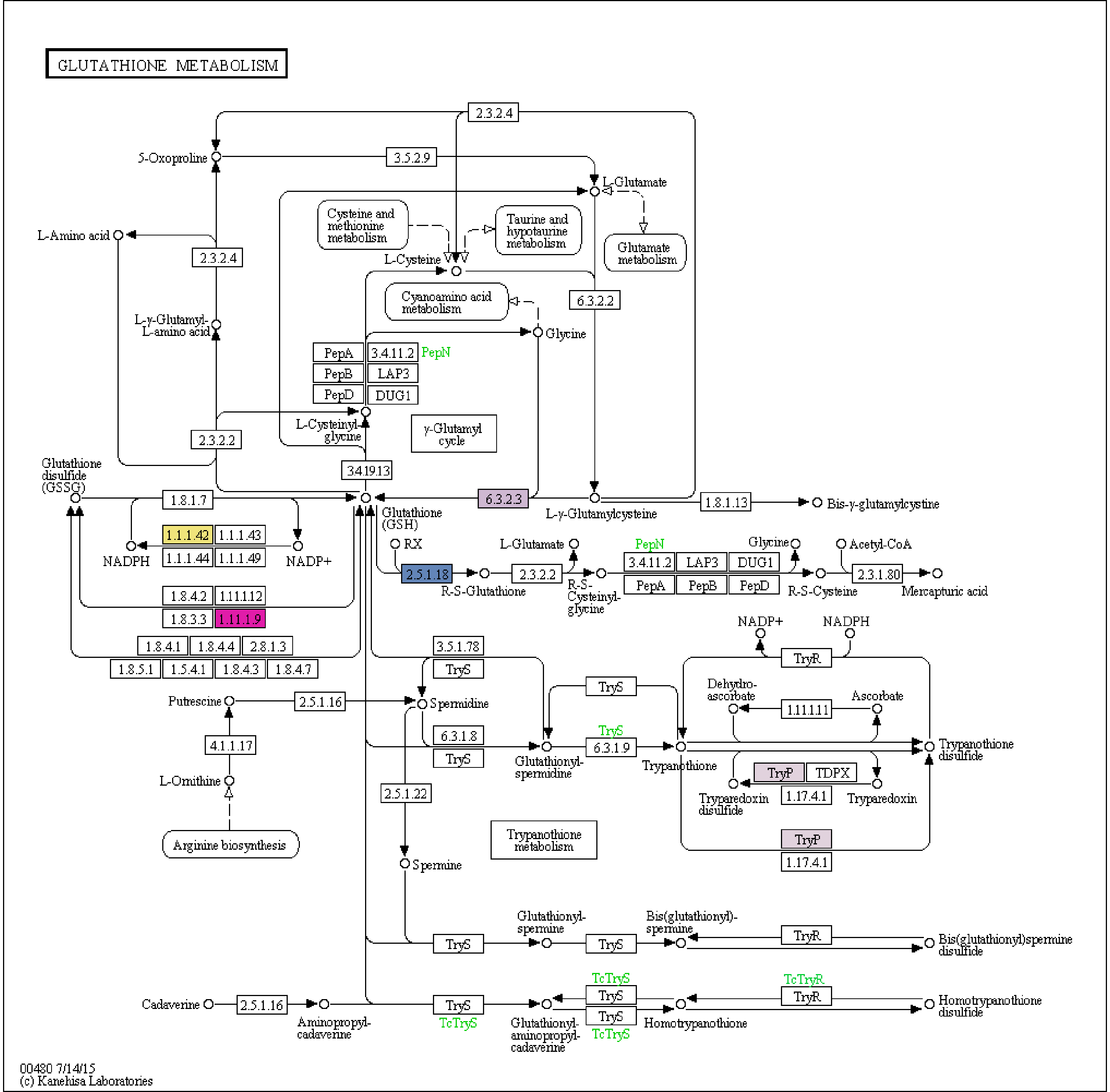
GLUTATHIONE METABOLISM
2.3.2.4
5-Oxoproline
3.5.2.9
L-Glutamate
L-Amino acid
2.3.2.4
Cysteine and methionine metabolism
Taurine and hypotaurine metabolism
Glutamate metabolism
L-Cysteine
Cyanoamino acid metabolism
6.3.2.2
L-γ-Glutamyl-L-amino acid
Glycine
PepA
3.4.11.2
PepN
PepB
LAP3
PepD
DUG1
L-Cysteinyl-glycine
γ-Glutamyl cycle
2.3.2.2
3.4.19.13
Glutathione disulfide (GSSG)
1.8.1.7
6.3.2.3
L-γ-Glutamylcysteine
1.8.1.13
Bis-γ-glutamylcystine
Glutathione (GSH)
1.1.1.42
1.1.1.43
1.1.1.44
1.1.1.49
NADPH
NADP+
RX
2.5.1.18
R-S-Glutathione
L-Glutamate
2.3.2.2
R-S-Cysteinyl-glycine
PepN
3.4.11.2
LAP3
DUG1
PepA
PepB
PepD
Glycine
Acetyl-CoA
R-S-Cysteine
2.3.1.80
Mercapturic acid
1.8.4.2
1.11.1.12
1.8.3.3
1.11.1.9
1.8.4.1
1.8.4.4
2.8.1.3
1.8.5.1
1.5.4.1
1.8.4.3
1.8.4.7
3.5.1.78
TryS
NADP+
NADPH
TryR
Putrescine
2.5.1.16
Spermidine
TryS
Dehydro-ascorbate
1.11.1.11
Ascorbate
4.1.1.17
6.3.1.8
TryS
6.3.1.9
TryS
Glutathionyl-spermidine
Trypanothione
Trypanothione disulfide
L-Ornithine
TryP
TDPX
Tryparedoxin disulfide
1.17.4.1
Tryparedoxin
2.5.1.22
Arginine biosynthesis
Trypanothione metabolism
TryP
1.17.4.1
Spermine
Glutathionyl-spermine
Bis(glutathionyl)-spermine
TryR
TryS
TryS
Bis(glutathionyl)spermine disulfide
TcTryS
TcTryR
TryS
TryR
Cadaverine
2.5.1.16
TryS
TryS
Homotrypanothione disulfide
Aminopropyl-cadaverine
TcTryS
Glutathionyl-aminopropyl-cadaverine
Homotrypanothione
TcTryS
00480 7/14/15
(c) Kanehisa Laboratories

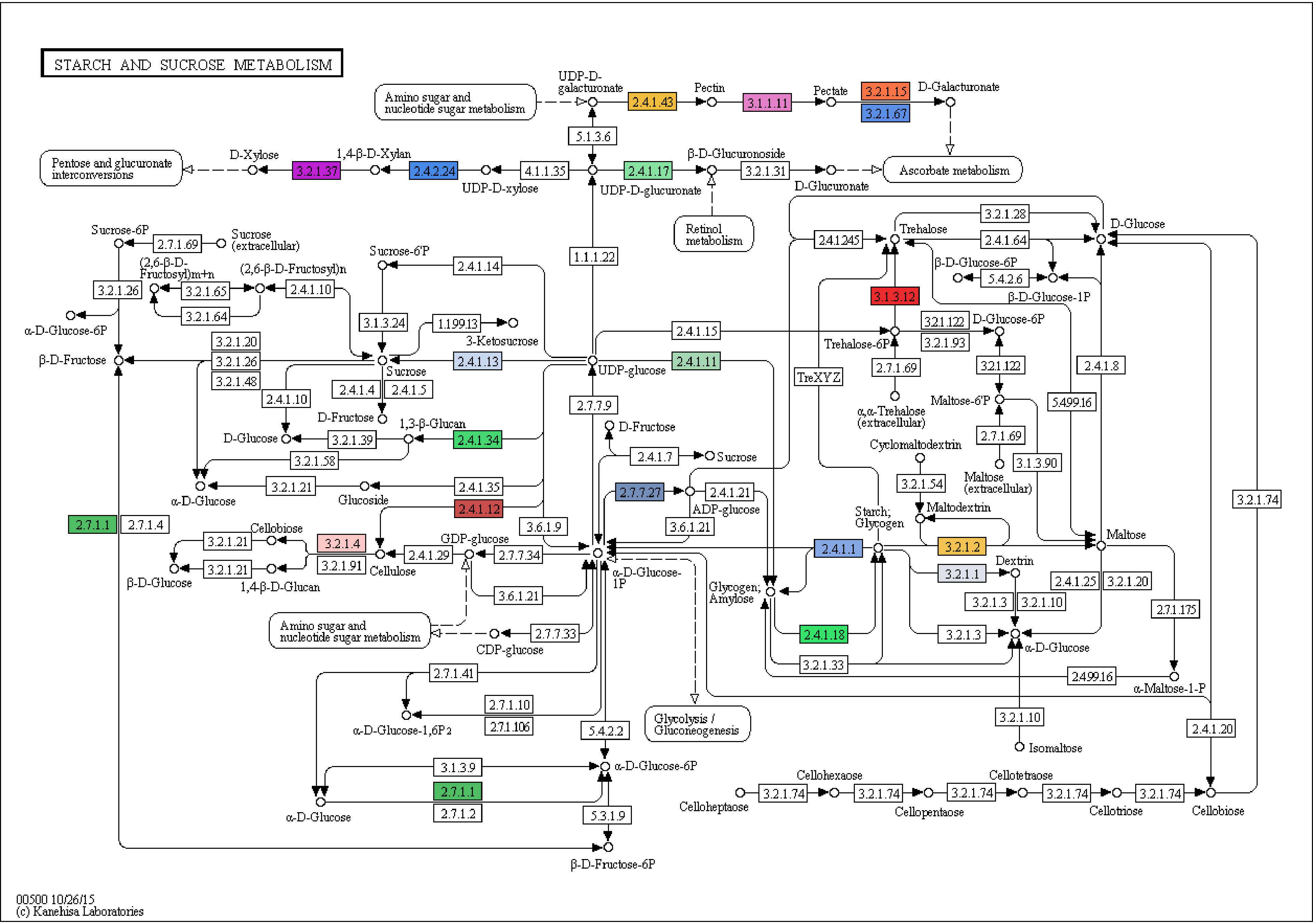
STARCH AND SUCROSE METABOLISM
Amino sugar and nucleotide sugar metabolism
UDP-D-galacturonate
2.4.1.43
Pectin
3.1.1.11
Pectate
3.2.1.15
3.2.1.67
D-Galacturonate
5.1.3.6
Pentose and glucuronate interconversions
D-Xylose
3.2.1.37
1,4-β-D-Xylan
2.4.2.24
UDP-D-xylose
4.1.1.35
2.4.1.17
UDP-D-glucuronate
β-D-Glucuronoside
3.2.1.31
D-Glucuronate
Ascorbate metabolism
Retinol metabolism
1.1.1.22
Sucrose-6P
2.7.1.69
Sucrose (extracellular)
(2,6-β-D-Fructosyl)m+n
(2,6-β-D-Fructosyl)n
3.2.1.26
3.2.1.65
2.4.1.10
3.2.1.64
α-D-Glucose-6P
Sucrose-6'P
2.4.1.14
3.1.3.24
1.1.99.13
3-Ketosucrose
3.2.1.20
3.2.1.26
3.2.1.48
β-D-Fructose
2.4.1.13
Sucrose
UDP-glucose
2.4.1.11
2.4.1.15
2.4.1.10
2.4.1.4
2.4.1.5
D-Fructose
D-Glucose
3.2.1.39
1,3-β-Glucan
2.4.1.34
3.2.1.58
α-D-Glucose
3.2.1.21
Glucoside
2.4.1.35
2.4.1.12
2.7.1.1
2.7.1.4
Cellobiose
3.2.1.21
3.2.1.4
3.2.1.21
3.2.1.91
β-D-Glucose
1,4-β-D-Glucan
Cellulose
2.4.1.29
GDP-glucose
2.7.7.34
3.6.1.9
2.7.7.9
D-Fructose
2.4.1.7
Sucrose
2.7.7.27
ADP-glucose
2.4.1.21
3.6.1.21
3.6.1.21
α-D-Glucose-1P
Amino sugar and nucleotide sugar metabolism
CDP-glucose
2.7.7.33
2.7.1.41
2.7.1.10
2.7.1.106
α-D-Glucose-1,6P2
5.4.2.2
Glycolysis / Gluconeogenesis
3.1.3.9
2.7.1.1
2.7.1.2
α-D-Glucose
α-D-Glucose-6P
5.3.1.9
β-D-Fructose-6P
3.2.1.28
Trehalose
2.4.1.245
2.4.1.64
D-Glucose
β-D-Glucose-6P
5.4.2.6
β-D-Glucose-1P
3.1.3.12
Trehalose-6P
3.2.1.122
D-Glucose-6P
3.2.1.93
3.2.1.122
TreXYZ
2.7.1.69
α,α-Trehalose (extracellular)
Maltose-6'P
2.7.1.69
Maltose (extracellular)
3.1.3.90
2.4.1.8
5.4.99.16
Cyclomaltodextrin
3.2.1.54
Maltodextrin
Starch; Glycogen
2.4.1.1
3.2.1.2
Maltose
Glycogen; Amylose
3.2.1.1
Dextrin
2.4.1.25
3.2.1.20
3.2.1.3
3.2.1.10
2.7.1.175
2.4.1.18
3.2.1.3
α-D-Glucose
3.2.1.33
2.4.99.16
α-Maltose-1-P
3.2.1.10
Isomaltose
2.4.1.20
3.2.1.74
Celloheptaose
3.2.1.74
Cellohexaose
3.2.1.74
Cellopentaose
3.2.1.74
Cellotetraose
3.2.1.74
Cellotriose
3.2.1.74
Cellobiose
00500 10/26/15
(c) Kanehisa Laboratories

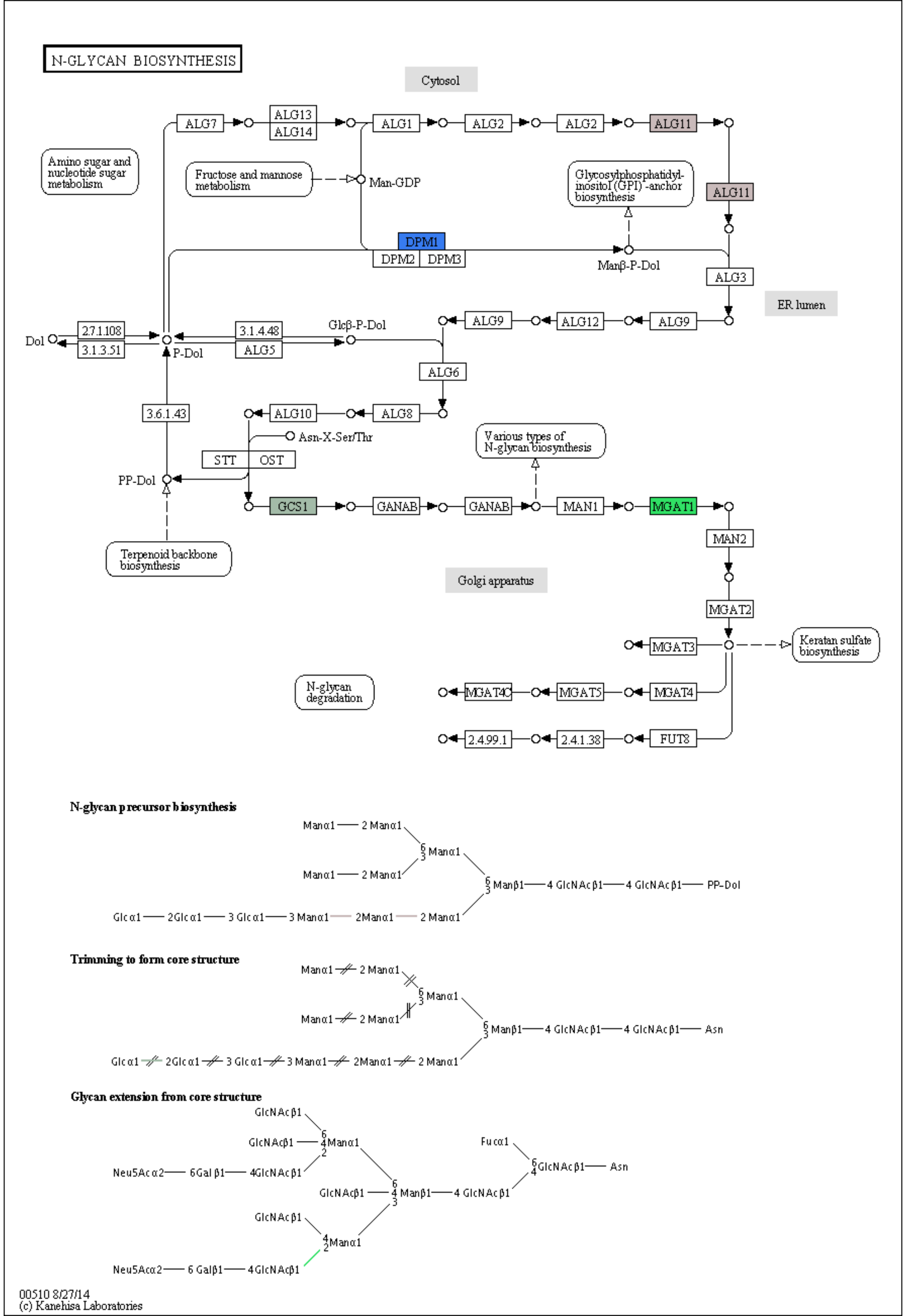
N-GLYCAN BIOSYNTHESIS
Cytosol
ER lumen
Golgi apparatus
ALG7
ALG13
ALG14
ALG1
ALG2
ALG11
ALG3
ALG9
ALG12
ALG6
ALG8
ALG10
ALG5
DPM1
DPM2
DPM3
Man-GDP
Manβ-P-Dol
Glcβ-P-Dol
Dol
P-Dol
PP-Dol
2.7.1.108
3.1.3.51
3.1.4.48
3.6.1.43
Asn-X-Ser/Thr
STT
OST
GCS1
GANAB
MAN1
MGAT1
MAN2
MGAT2
MGAT3
MGAT4
MGAT5
MGAT4C
FUT8
2.4.1.38
2.4.99.1
Amino sugar and nucleotide sugar metabolism
Fructose and mannose metabolism
Glycosylphosphatidyl-inositol (GPI) -anchor biosynthesis
Various types of N-glycan biosynthesis
Terpenoid backbone biosynthesis
Keratan sulfate biosynthesis
N-glycan degradation
N-glycan precursor biosynthesis
Manα1 — 2 Manα1
Manα1 — 2 Manα1
6 3 Manα1
6 3 Manβ1 — 4 GlcNAcβ1 — 4 GlcNAcβ1 — PP-Dol
Glcα1 — 2Glcα1 — 3 Glcα1 — 3 Manα1 — 2Manα1 — 2 Manα1
Trimming to form core structure
6 3 Manβ1 — 4 GlcNAcβ1 — 4 GlcNAcβ1 — Asn
Glycan extension from core structure
GlcNAcβ1
GlcNAcβ1
Neu5Acα2 — 6Galβ1 — 4GlcNAcβ1
6 4 2 Manα1
GlcNAcβ1
6 4 3 Manβ1 — 4 GlcNAcβ1
Fucα1
6 4 GlcNAcβ1 — Asn
GlcNAcβ1
4 2 Manα1
Neu5Acα2 — 6 Galβ1 — 4GlcNAcβ1
00510 8/27/14
(c) Kanehisa Laboratories

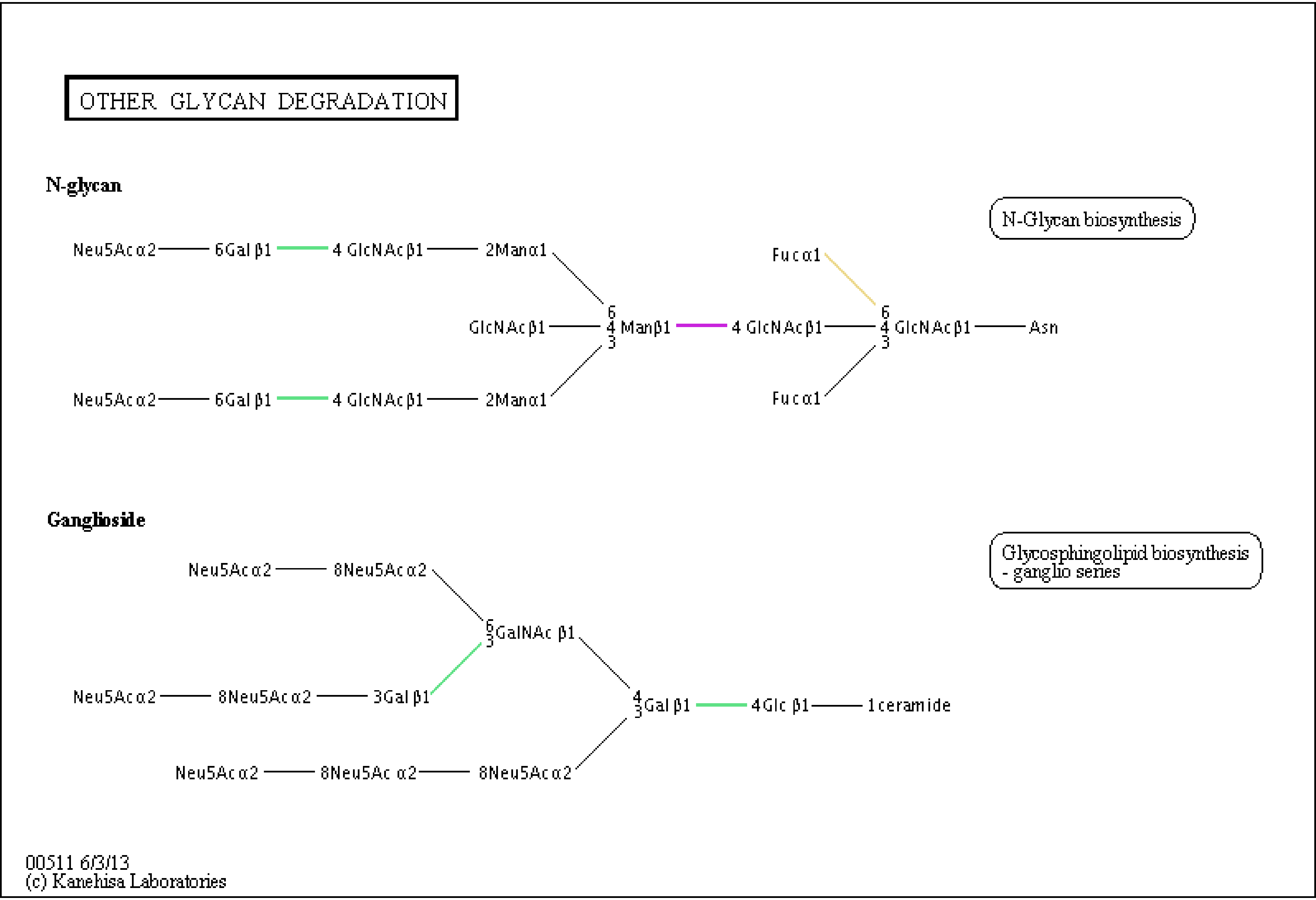
OTHER GLYCAN DEGRADATION
N-glycan
N-Glycan biosynthesis
Neu5Acα2 6Galβ1 4GlcNAcβ1 2Manα1
Fucα1
GlcNAcβ1 6 4 3 Manβ1 4 GlcNAcβ1 6 4 3 GlcNAcβ1 Asn
Neu5Acα2 6Galβ1 4GlcNAcβ1 2Manα1
Fucα1
Ganglioside
Glycosphingolipid biosynthesis - ganglio series
Neu5Acα2 8Neu5Acα2
6 3 GalNAcβ1
Neu5Acα2 8Neu5Acα2 3Galβ1
4 3 Galβ1 4Glcβ1 1ceramide
Neu5Acα2 8Neu5Acα2 8Neu5Acα2
00511 6/3/13
(c) Kanehisa Laboratories

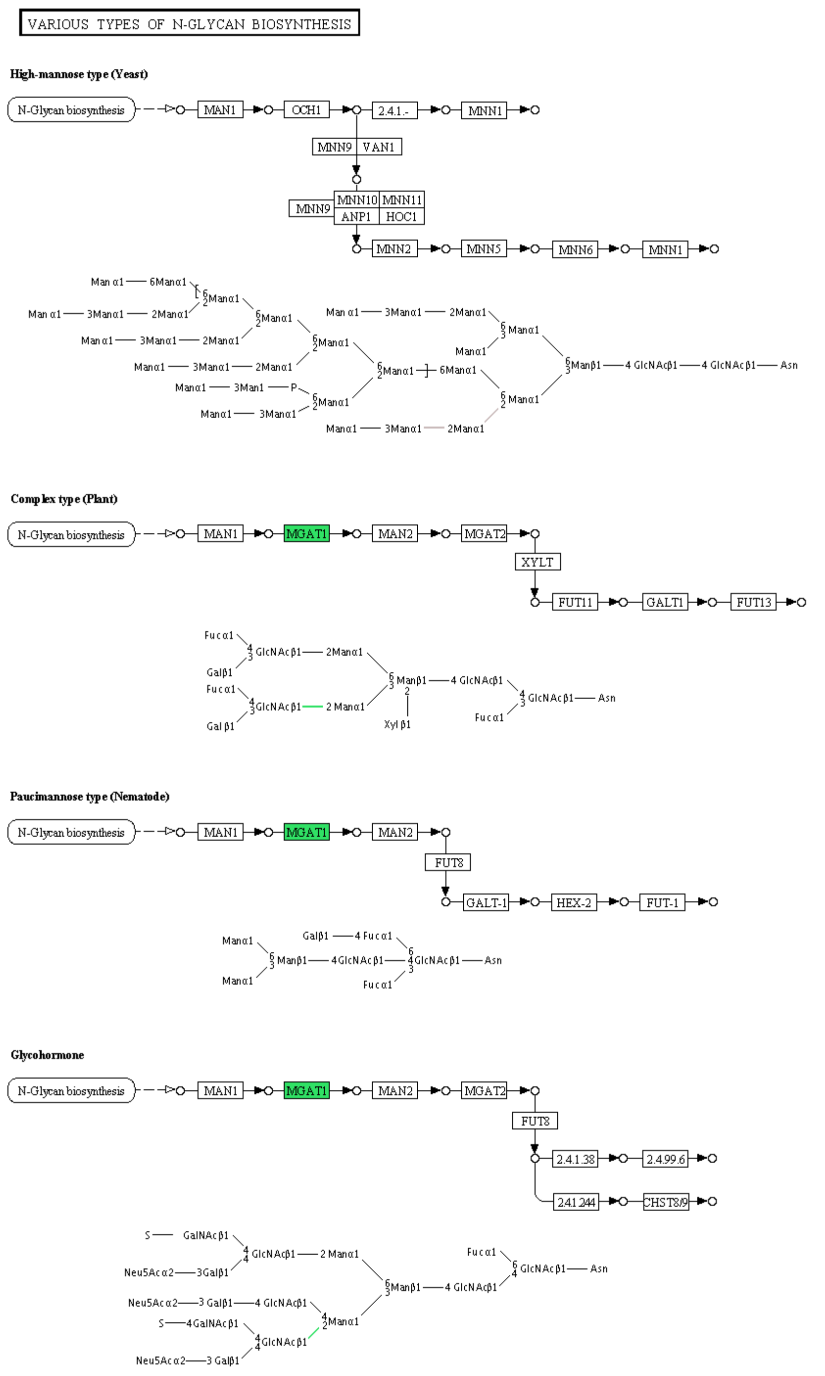

00513 11/20/12
(c) Kanehisa Laboratories

OTHER TYPES OF O-GLYCAN BIOSYNTHESIS

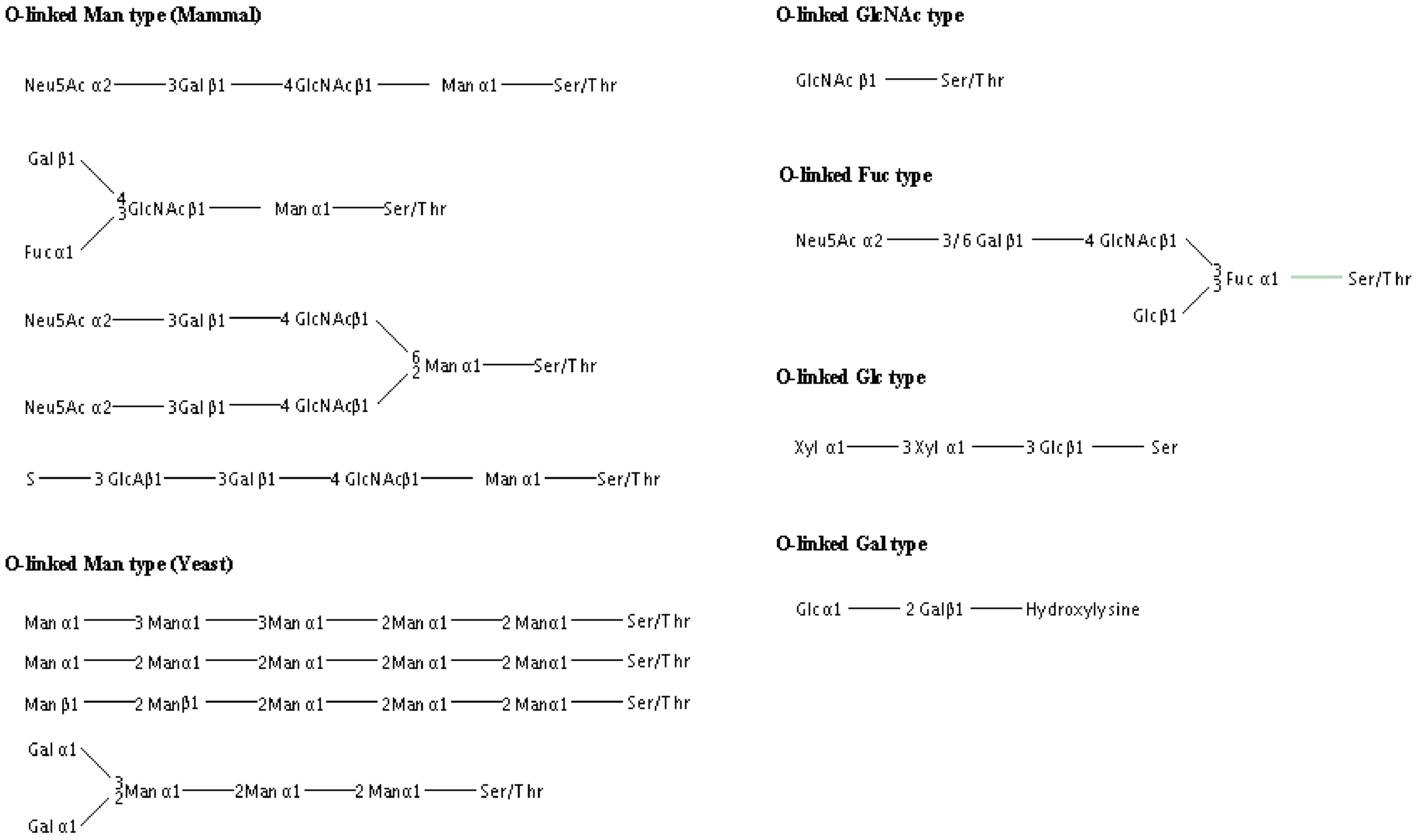

00514 11/20/12

(c) Kanehisa Laboratories

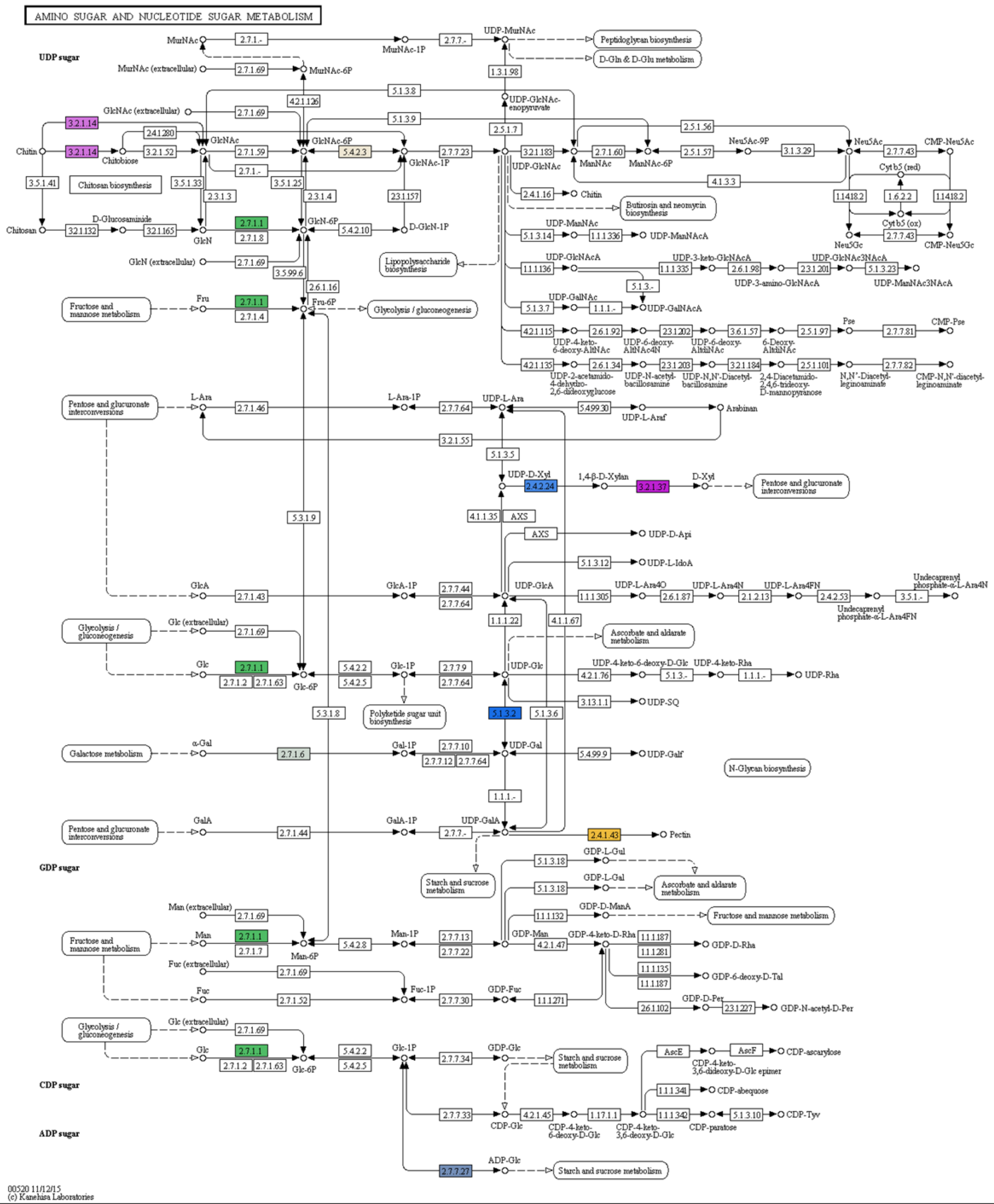
AMINO SUGAR AND NUCLEOTIDE SUGAR METABOLISM
UDP sugar
MurNAc
2.7.1.-
MurNAc-1P
2.7.7.-
UDP-MurNAc
Peptidoglycan biosynthesis
D-Gln & D-Glu metabolism
MurNAc (extracellular)
2.7.1.69
MurNAc-6P
1.3.1.98
5.1.3.8
4.2.1.126
UDP-GlcNAc-enopyruvate
GlcNAc (extracellular)
5.1.3.9
2.5.1.7
2.5.1.56
3.2.1.14
2.4.1.280
GlcNAc
GlcNAc-6P
Neu5Ac-9P
Neu5Ac
CMP-Neu5Ac
Chitin
Chitobiose
3.2.1.52
2.7.1.59
5.4.2.3
2.7.7.23
3.2.1.183
2.7.1.60
2.5.1.57
3.1.3.29
2.7.7.43
GlcNAc-1P
UDP-GlcNAc
ManNAc
ManNAc-6P
2.7.1.-
4.1.3.3
Cyt b5 (red)
3.5.1.41
Chitosan biosynthesis
3.5.1.33
3.5.1.25
2.3.1.157
2.4.1.16
Chitin
1.14.18.2
1.6.2.2
Cyt b5 (ox)
2.3.1.3
2.3.1.4
Butirosin and neomycin biosynthesis
Chitosan
D-Glucosaminide
3.2.1.132
3.2.1.165
2.7.1.1
2.7.1.8
GlcN-6P
5.4.2.10
D-GlcN-1P
UDP-ManNAc
5.1.3.14
1.1.1.336
Neu5Gc
CMP-Neu5Gc
GlcN
GlcN (extracellular)
Lipopolysaccharide biosynthesis
UDP-GlcNAcA
1.1.1.136
UDP-3-keto-GlcNAcA
1.1.1.335
2.6.1.98
UDP-GlcNAc3NAcA
2.3.1.201
5.1.3.23
UDP-3-amino-GlcNAcA
UDP-ManNAc3NAcA
3.5.99.6
2.6.1.16
5.1.3.-
Fructose and mannose metabolism
Fru
2.7.1.4
Fru-6P
Glycolysis / gluconeogenesis
UDP-GalNAc
5.1.3.7
1.1.1.-
UDP-GalNAcA
4.2.1.115
2.6.1.92
2.3.1.202
3.6.1.57
2.5.1.97
Pse
2.7.7.81
CMP-Pse
UDP-4-keto-6-deoxy-AltNAc
UDP-6-deoxy-AltNAc4N
UDP-6-deoxy-AltdiNAc
6-Deoxy-AltdiNAc
4.2.1.135
2.6.1.34
2.3.1.203
3.2.1.184
2.5.1.101
2.7.7.82
UDP-2-acetamido-4-dehydro-2,6-dideoxyglucose
UDP-N-acetyl-bacillosamine
UDP-N,N'-Diacetyl-bacillosamine
2,4-Diacetamido-2,4,6-trideoxy-D-mannopyranose
N,N'-Diacetyl-legionaminate
CMP-N,N'-diacetyl-legionaminate
Pentose and glucuronate interconversions
L-Ara
2.7.1.46
L-Ara-1P
2.7.7.64
UDP-L-Ara
5.4.99.30
UDP-L-Araf
Arabinan
3.2.1.55
5.1.3.5
UDP-D-Xyl
2.4.2.24
1,4-β-D-Xylan
3.2.1.37
D-Xyl
Pentose and glucuronate interconversions
5.3.1.9
4.1.1.35
AXS
AXS
UDP-D-Api
5.1.3.12
UDP-L-IdoA
GlcA
2.7.1.43
GlcA-1P
2.7.7.44
2.7.7.64
UDP-GlcA
1.1.1.305
UDP-L-Ara4O
2.6.1.87
UDP-L-Ara4N
2.1.2.13
UDP-L-Ara4FN
2.4.2.53
3.5.1.-
Undecaprenyl phosphate-α-L-Ara4N
Undecaprenyl phosphate-α-L-Ara4FN
1.1.1.22
4.1.1.67
Ascorbate and aldarate metabolism
Glycolysis / gluconeogenesis
Glc (extracellular)
2.7.1.69
Glc
2.7.1.1
2.7.1.2
2.7.1.63
Glc-6P
5.4.2.2
5.4.2.5
Glc-1P
2.7.7.9
2.7.7.64
UDP-Glc
4.2.1.76
UDP-4-keto-6-deoxy-D-Glc
5.1.3.-
UDP-4-keto-Rha
1.1.1.-
UDP-Rha
3.13.1.1
UDP-SQ
5.3.1.8
Polyketide sugar unit biosynthesis
5.1.3.2
5.1.3.6
Galactose metabolism
α-Gal
2.7.1.6
Gal-1P
2.7.7.10
2.7.7.12
2.7.7.64
UDP-Gal
5.4.99.9
UDP-Galf
N-Glycan biosynthesis
1.1.1.-
Pentose and glucuronate interconversions
GalA
2.7.1.44
GalA-1P
2.7.7.-
UDP-GalA
2.4.1.43
Pectin
GDP sugar
5.1.3.18
GDP-L-Gul
GDP-L-Gal
Starch and sucrose metabolism
Ascorbate and aldarate metabolism
1.1.1.132
GDP-D-ManA
Fructose and mannose metabolism
Man (extracellular)
2.7.1.69
Fructose and mannose metabolism
Man
2.7.1.1
2.7.1.7
Man-6P
5.4.2.8
Man-1P
2.7.7.13
2.7.7.22
GDP-Man
4.2.1.47
GDP-4-keto-D-Rha
1.1.1.187
1.1.1.281
GDP-D-Rha
1.1.1.135
1.1.1.187
GDP-6-deoxy-D-Tal
Fuc (extracellular)
2.7.1.69
Fuc
2.7.1.52
Fuc-1P
2.7.7.30
GDP-Fuc
1.1.1.271
2.6.1.102
GDP-D-Per
2.3.1.227
GDP-N-acetyl-D-Per
Glycolysis / gluconeogenesis
Glc (extracellular)
2.7.1.69
Glc
2.7.1.1
2.7.1.2
2.7.1.63
Glc-6P
5.4.2.2
5.4.2.5
Glc-1P
2.7.7.34
GDP-Glc
Starch and sucrose metabolism
AscE
AscF
CDP-ascarylose
CDP-4-keto-3,6-dideoxy-D-Glc epimer
CDP sugar
1.1.1.341
CDP-abequose
2.7.7.33
CDP-Glc
4.2.1.45
CDP-4-keto-6-deoxy-D-Glc
1.17.1.1
CDP-4-keto-3,6-deoxy-D-Glc
1.1.1.342
CDP-paratose
5.1.3.10
CDP-Tyv
ADP sugar
2.7.7.27
ADP-Glc
Starch and sucrose metabolism
00520 11/12/15
(c) Kanehisa Laboratories

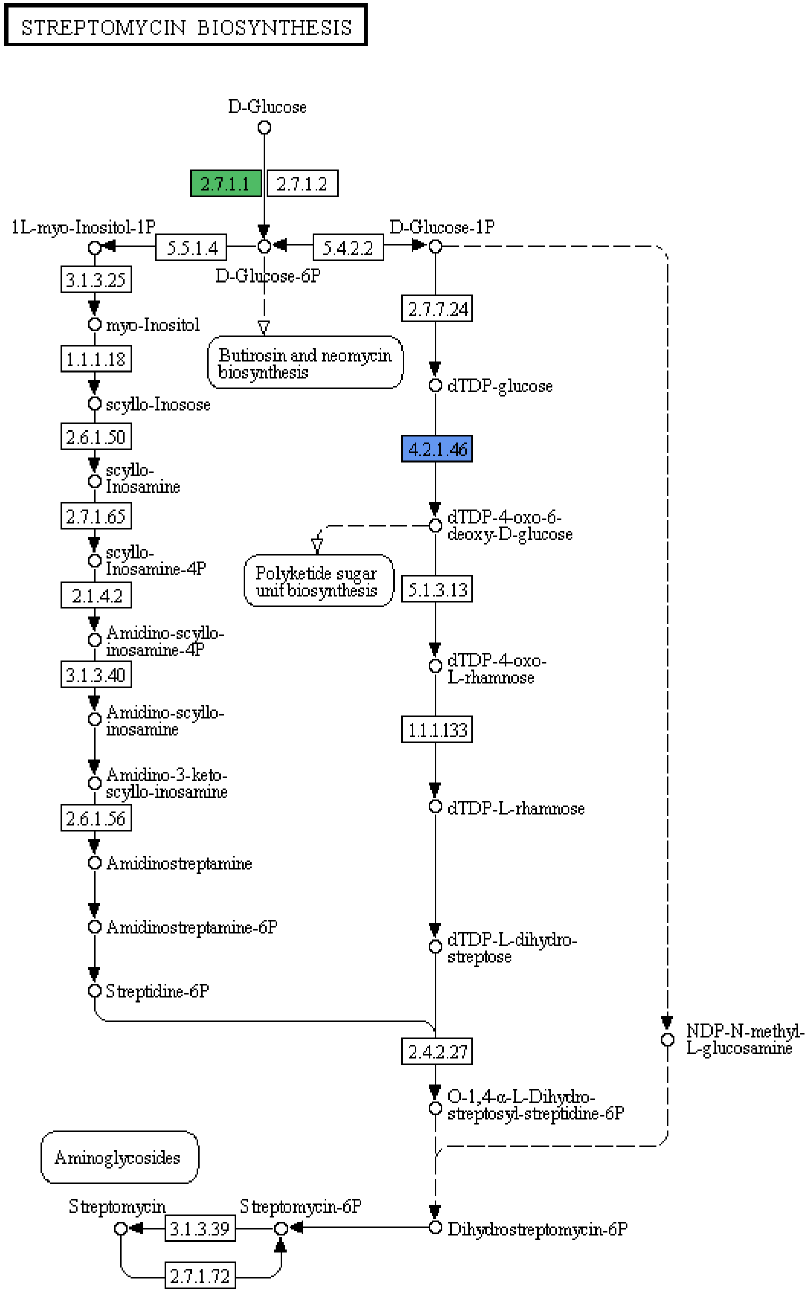

00521 12/27/10
(c) Kanehisa Laboratories

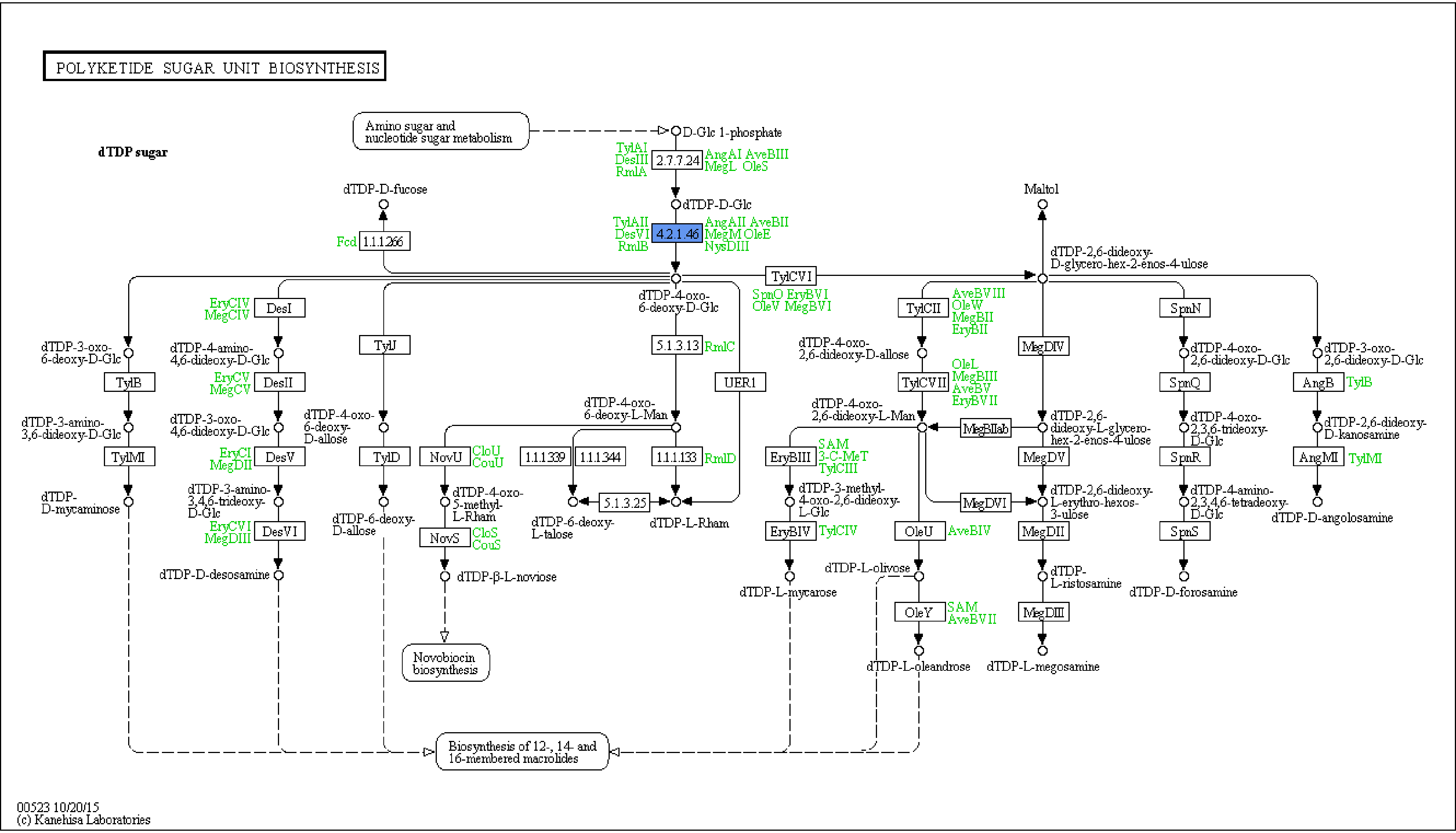
POLYKETIDE SUGAR UNIT BIOSYNTHESIS
dTDP sugar
Amino sugar and nucleotide sugar metabolism
D-Glc 1-phosphate
TylAI DesIII RmlA
2.7.7.24
AngAI AveBIII MegL OleS
dTDP-D-Glc
TylAII DesVI RmlB
4.2.1.46
AngAII AveBII MegM OleE NysDIII
dTDP-D-fucose
Fcd
1.1.1.266
Maltol
dTDP-2,6-dideoxy-D-glycero-hex-2-enos-4-ulose
TylCVI
SpnO EryBVI OleV MegBVI
dTDP-4-oxo-6-deoxy-D-Glc
EryCIV MegCIV
DesI
TylJ
5.1.3.13
RmlC
UER1
TylCII
AveBVIII OleW MegBII EryBII
MegDIV
SpnN
dTDP-3-oxo-6-deoxy-D-Glc
dTDP-4-amino-4,6-dideoxy-D-Glc
TylB
EryCV MegCV
DesII
dTDP-4-oxo-2,6-dideoxy-D-allose
TylCVII
OleL MegBIII AveBV EryBVII
dTDP-4-oxo-2,6-dideoxy-D-Glc
SpnQ
dTDP-3-oxo-2,6-dideoxy-D-Glc
AngB
TylB
dTDP-3-amino-3,6-dideoxy-D-Glc
dTDP-3-oxo-4,6-dideoxy-D-Glc
dTDP-4-oxo-6-deoxy-D-allose
dTDP-4-oxo-6-deoxy-L-Man
dTDP-4-oxo-2,6-dideoxy-L-Man
MegBIIab
dTDP-2,6-dideoxy-L-glycero-hex-2-enos-4-ulose
dTDP-4-oxo-2,3,6-trideoxy-D-Glc
dTDP-2,6-dideoxy-D-kanosamine
TylMI
EryCI MegDII
DesV
TylD
NovU
CloU CouU
1.1.1.339
1.1.1.344
1.1.1.133
RmlD
EryBIII
SAM 3-C-MeT TylCIII
MegDV
SpnR
AngMI
TylMI
dTDP-D-mycaminose
dTDP-3-amino-3,4,6-trideoxy-D-Glc
EryCVI MegDIII
DesVI
dTDP-6-deoxy-D-allose
dTDP-4-oxo-5-methyl-L-Rham
NovS
CloS CouS
dTDP-6-deoxy-L-talose
5.1.3.25
dTDP-L-Rham
dTDP-3-methyl-4-oxo-2,6-dideoxy-L-Glc
EryBIV
TylCIV
OleU
AveBIV
MegDVI
dTDP-2,6-dideoxy-L-erythro-hexos-3-ulose
MegDII
dTDP-4-amino-2,3,4,6-tetradeoxy-D-Glc
SpnS
dTDP-D-angolosamine
dTDP-D-desosamine
dTDP-β-L-noviose
dTDP-L-mycarose
dTDP-L-olivose
dTDP-L-ristosamine
dTDP-D-forosamine
OleY
SAM AveBVII
MegDIII
Novobiocin biosynthesis
dTDP-L-oleandrose
dTDP-L-megosamine
Biosynthesis of 12-, 14- and 16-membered macrolides
00523 10/20/15
(c) Kanehisa Laboratories

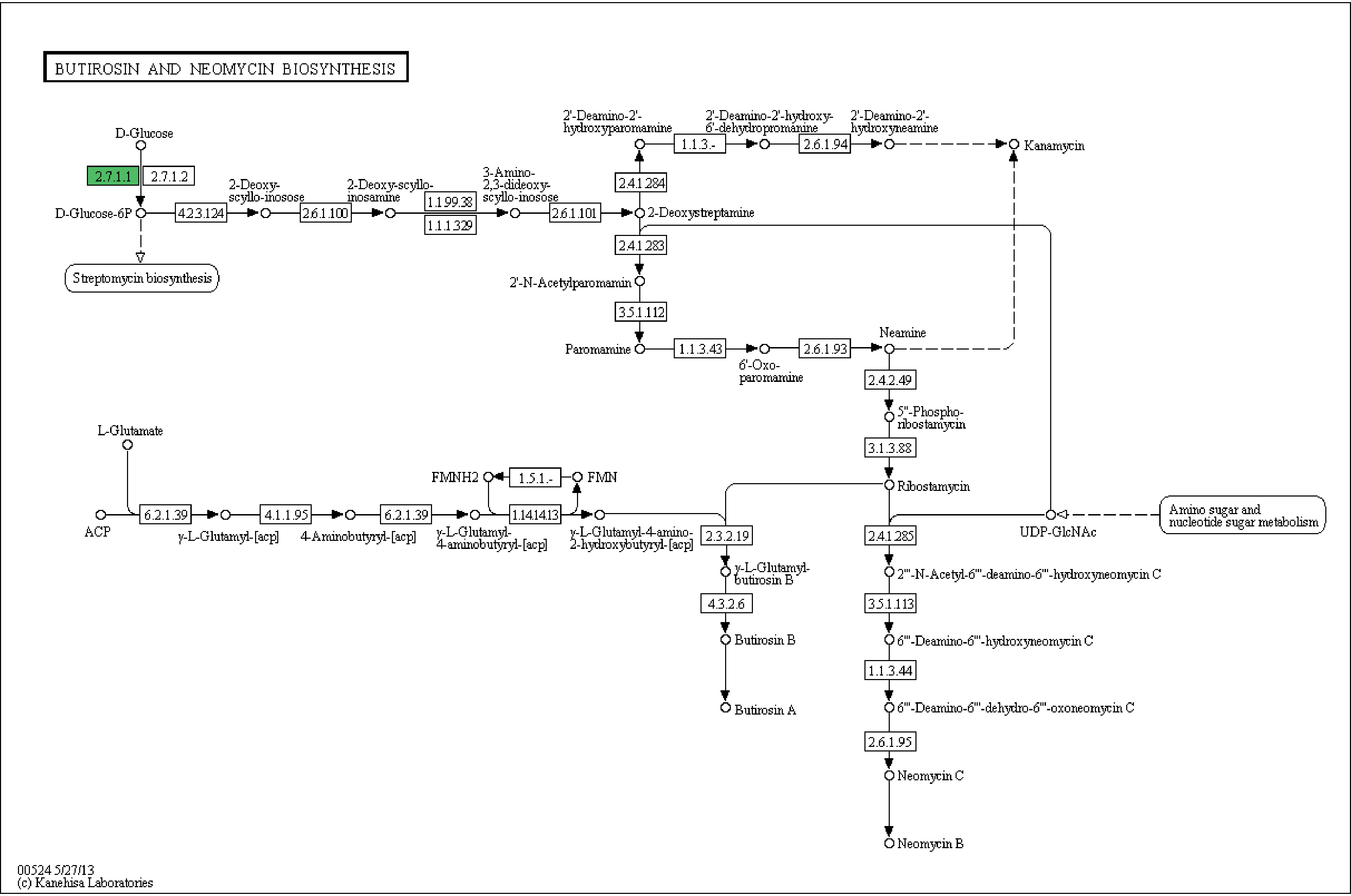
BUTIROSIN AND NEOMYCIN BIOSYNTHESIS
D-Glucose
2.7.1.1
2.7.1.2
D-Glucose-6P
Streptomycin biosynthesis
4.2.3.124
2-Deoxy-scyllo-inosose
2.6.1.100
2-Deoxy-scyllo-inosamine
1.1.99.38
1.1.1.329
3-Amino-2,3-dideoxy-scyllo-inosose
2.6.1.101
2-Deoxystreptamine
2.4.1.284
2'-Deamino-2'-hydroxyparomamine
1.1.3.-
2'-Deamino-2'-hydroxy-6'-dehydroparomamine
2.6.1.94
2'-Deamino-2'-hydroxyneamine
Kanamycin
2.4.1.283
2'-N-Acetylparomamin
3.5.1.112
Paromamine
1.1.3.43
6'-Oxo-paromamine
2.6.1.93
Neamine
2.4.2.49
5''-Phospho-ribostamycin
3.1.3.88
Ribostamycin
L-Glutamate
ACP
6.2.1.39
γ-L-Glutamyl-[acp]
4.1.1.95
4-Aminobutyryl-[acp]
6.2.1.39
γ-L-Glutamyl-4-aminobutyryl-[acp]
FMNH2
1.5.1.-
FMN
1.14.14.13
γ-L-Glutamyl-4-amino-2-hydroxybutyryl-[acp]
2.3.2.19
γ-L-Glutamyl-butirosin B
4.3.2.6
Butirosin B
Butirosin A
2.4.1.285
UDP-GlcNAc
Amino sugar and nucleotide sugar metabolism
2'''-N-Acetyl-6'''-deamino-6'''-hydroxyneomycin C
3.5.1.113
6'''-Deamino-6'''-hydroxyneomycin C
1.1.3.44
6'''-Deamino-6'''-dehydro-6'''-oxoneomycin C
2.6.1.95
Neomycin C
Neomycin B
00524 5/27/13
(c) Kanehisa Laboratories

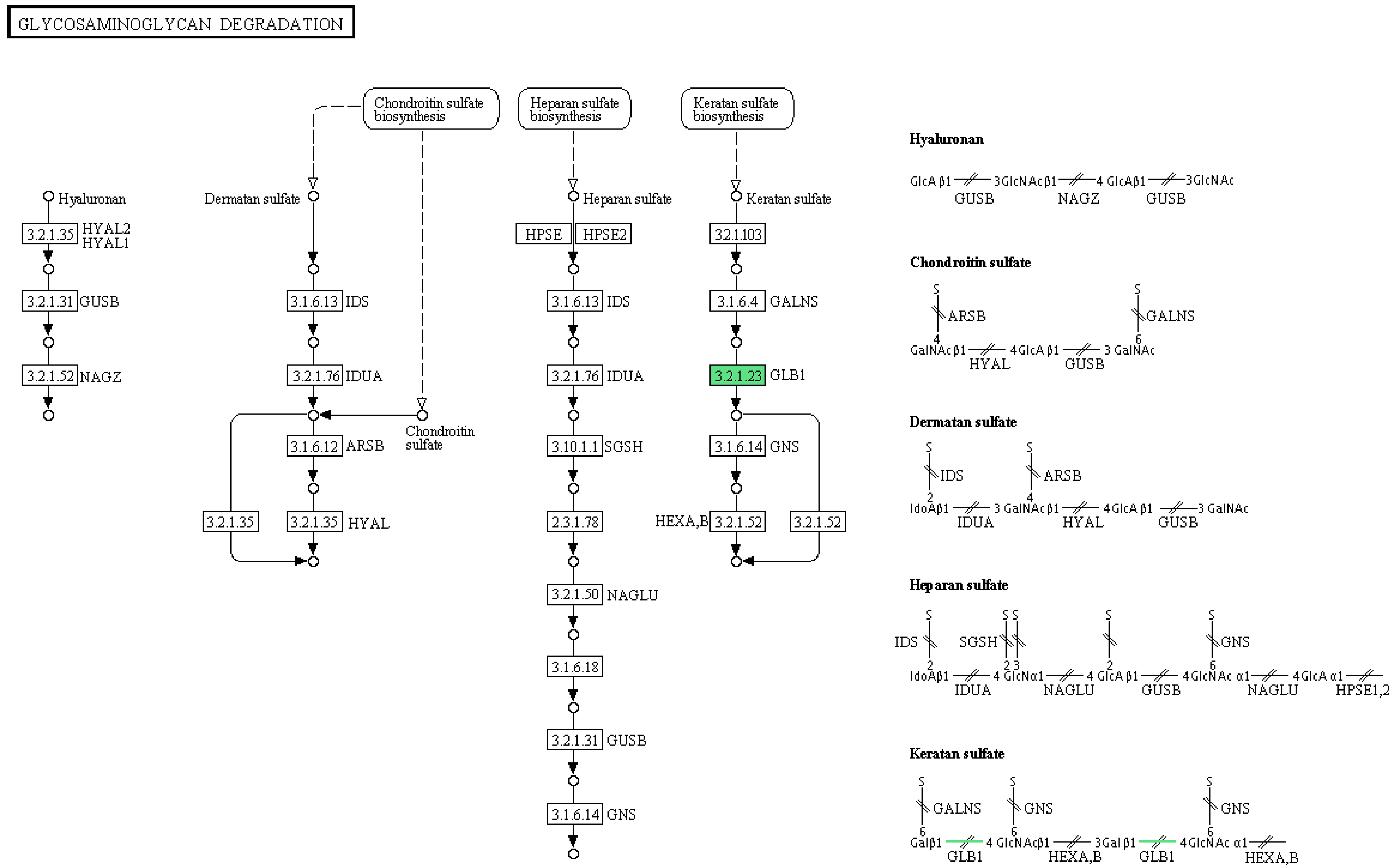

00531 5/22/15

(c) Kanehisa Laboratories

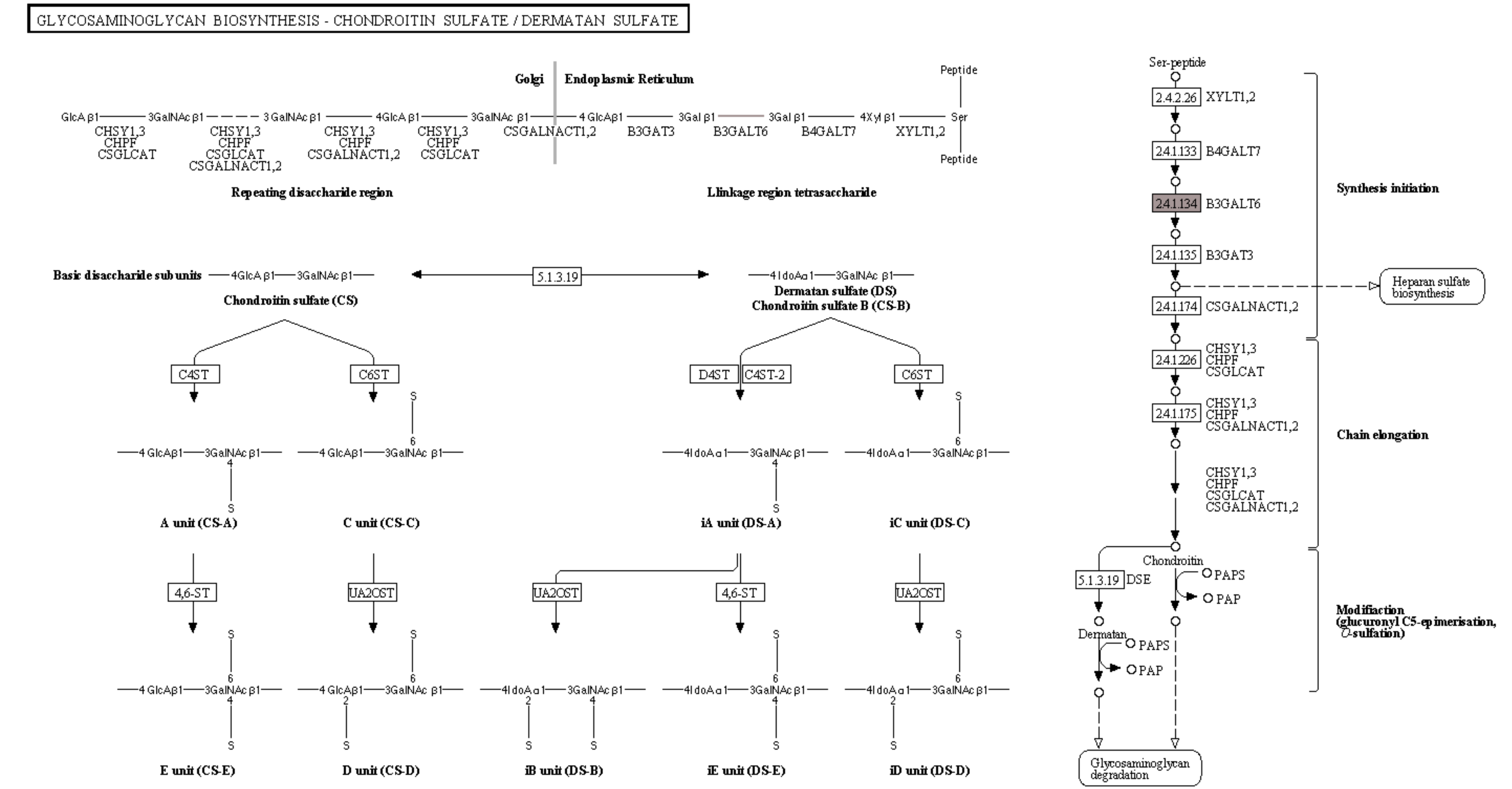

00532 1/8/13

(c) Kanehisa Laboratories

GLYCOSAMINOGLYCAN BIOSYNTHEISIS - HEPARAN SULFATE / HEPARIN

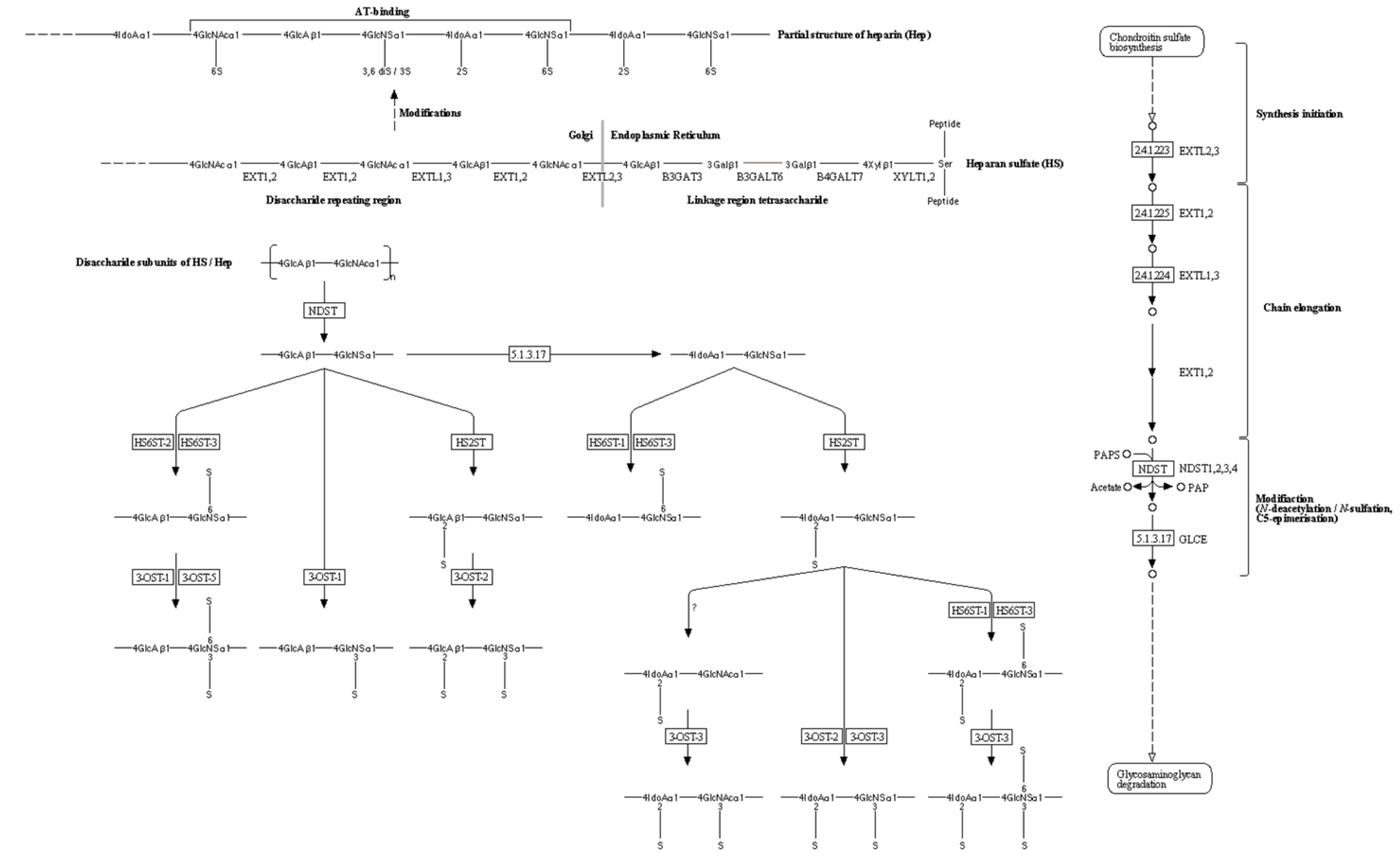

00534 1/8/13
(c) Kanehisa Laboratories

LIPOPOLYSACCHARIDE BIOSYNTHESIS

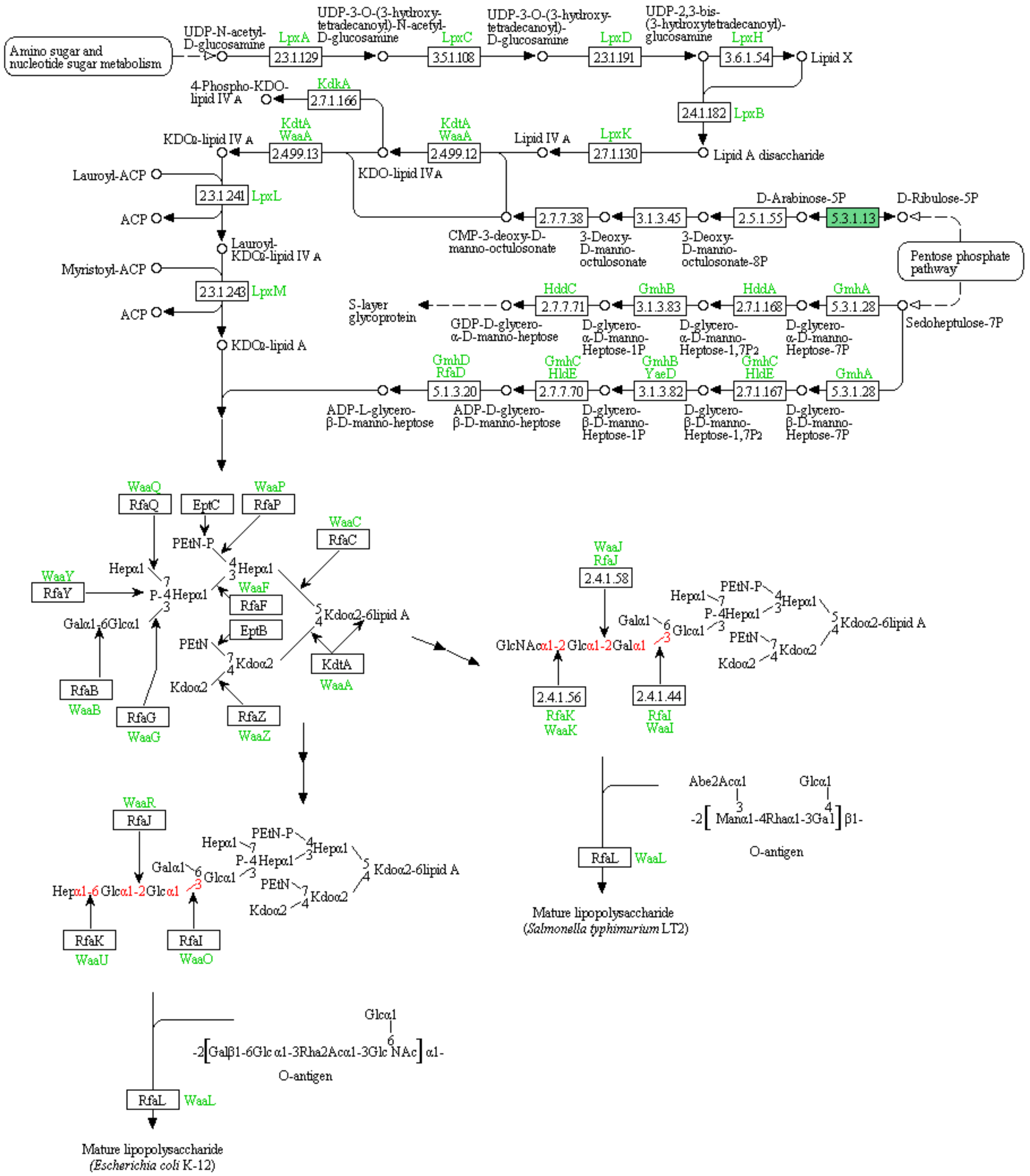

00540 6/16/15
(c) Kanehisa Laboratories

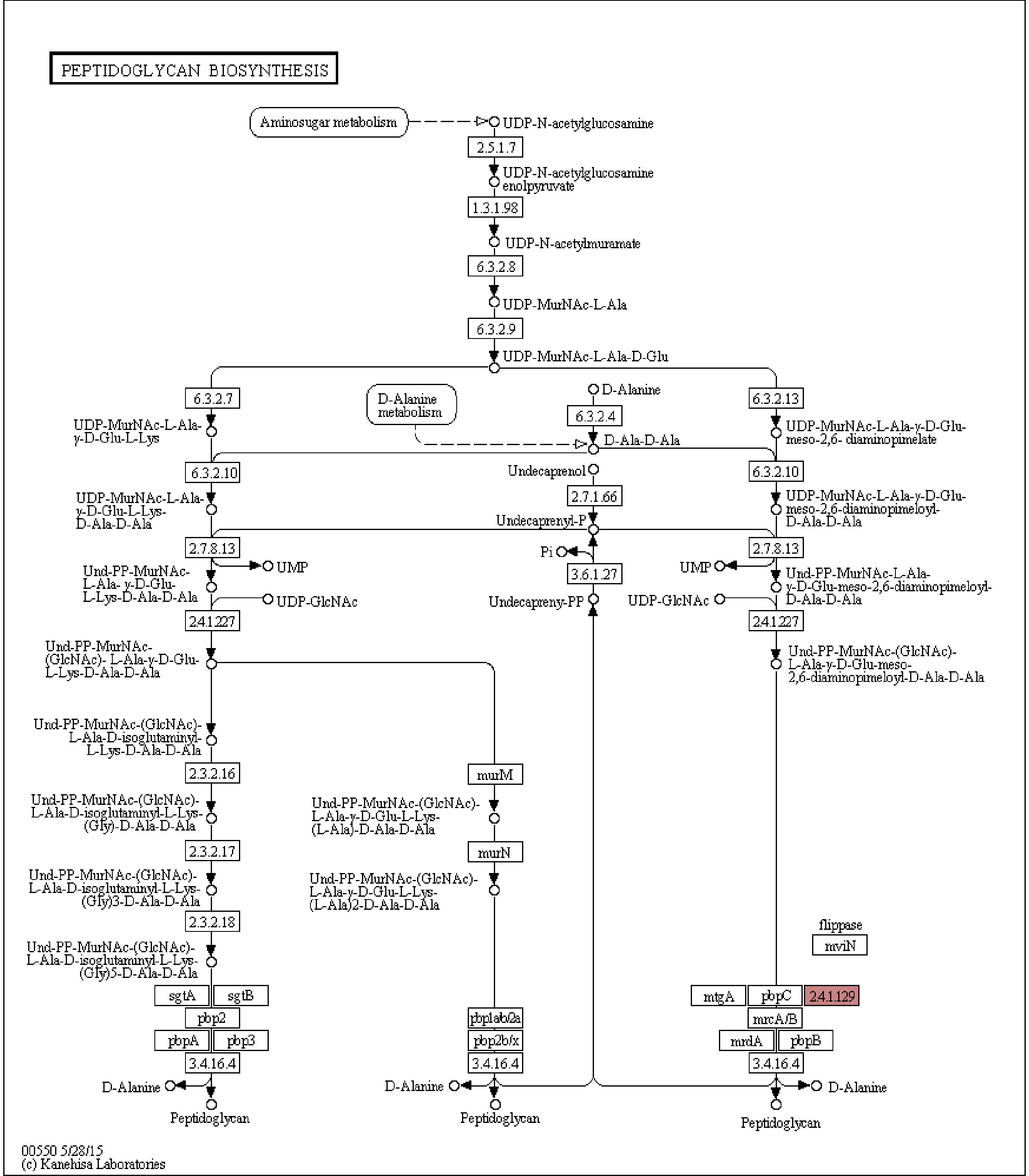
PEPTIDOGLYCAN BIOSYNTHESIS
Aminosugar metabolism
UDP-N-acetylglucosamine
2.5.1.7
UDP-N-acetylglucosamine enolpyruvate
1.3.1.98
UDP-N-acetylmuramate
6.3.2.8
UDP-MurNAc-L-Ala
6.3.2.9
UDP-MurNAc-L-Ala-D-Glu
6.3.2.7
D-Alanine metabolism
D-Alanine
6.3.2.4
D-Ala-D-Ala
6.3.2.13
UDP-MurNAc-L-Ala-γ-D-Glu-L-Lys
UDP-MurNAc-L-Ala-γ-D-Glu-meso-2,6- diaminopimelate
6.3.2.10
Undecaprenol
2.7.1.66
UDP-MurNAc-L-Ala-γ-D-Glu-L-Lys-D-Ala-D-Ala
UDP-MurNAc-L-Ala-γ-D-Glu-meso-2,6-diaminopimeloyl-D-Ala-D-Ala
Undecaprenyl-P
2.7.8.13
Pi
UMP
3.6.1.27
Und-PP-MurNAc-L-Ala- γ-D-Glu-L-Lys-D-Ala-D-Ala
Und-PP-MurNAc-L-Ala-γ-D-Glu-meso-2,6-diaminopimeloyl-D-Ala-D-Ala
UDP-GlcNAc
Undecapreny-PP
2.4.1.227
Und-PP-MurNAc-(GlcNAc)- L-Ala-γ-D-Glu-L-Lys-D-Ala-D-Ala
Und-PP-MurNAc-(GlcNAc)-L-Ala-γ-D-Glu-meso-2,6-diaminopimeloyl-D-Ala-D-Ala
Und-PP-MurNAc-(GlcNAc)-L-Ala-D-isoglutaminyl-L-Lys-D-Ala-D-Ala
2.3.2.16
Und-PP-MurNAc-(GlcNAc)-L-Ala-D-isoglutaminyl-L-Lys-(Gly)-D-Ala-D-Ala
murM
Und-PP-MurNAc-(GlcNAc)-L-Ala-γ-D-Glu-L-Lys-(L-Ala)-D-Ala-D-Ala
2.3.2.17
murN
Und-PP-MurNAc-(GlcNAc)-L-Ala-D-isoglutaminyl-L-Lys-(Gly)3-D-Ala-D-Ala
Und-PP-MurNAc-(GlcNAc)-L-Ala-γ-D-Glu-L-Lys-(L-Ala)2-D-Ala-D-Ala
2.3.2.18
Und-PP-MurNAc-(GlcNAc)-L-Ala-D-isoglutaminyl-L-Lys-(Gly)5-D-Ala-D-Ala
flippase
mviN
sgtA
sgtB
pbp2
pbpA
pbp3
3.4.16.4
pbp1a/b/2a
pbp2b/x
mtgA
pbpC
2.4.1.129
mrcA/B
mrdA
pbpB
Peptidoglycan
00550 5/28/15
(c) Kanehisa Laboratories

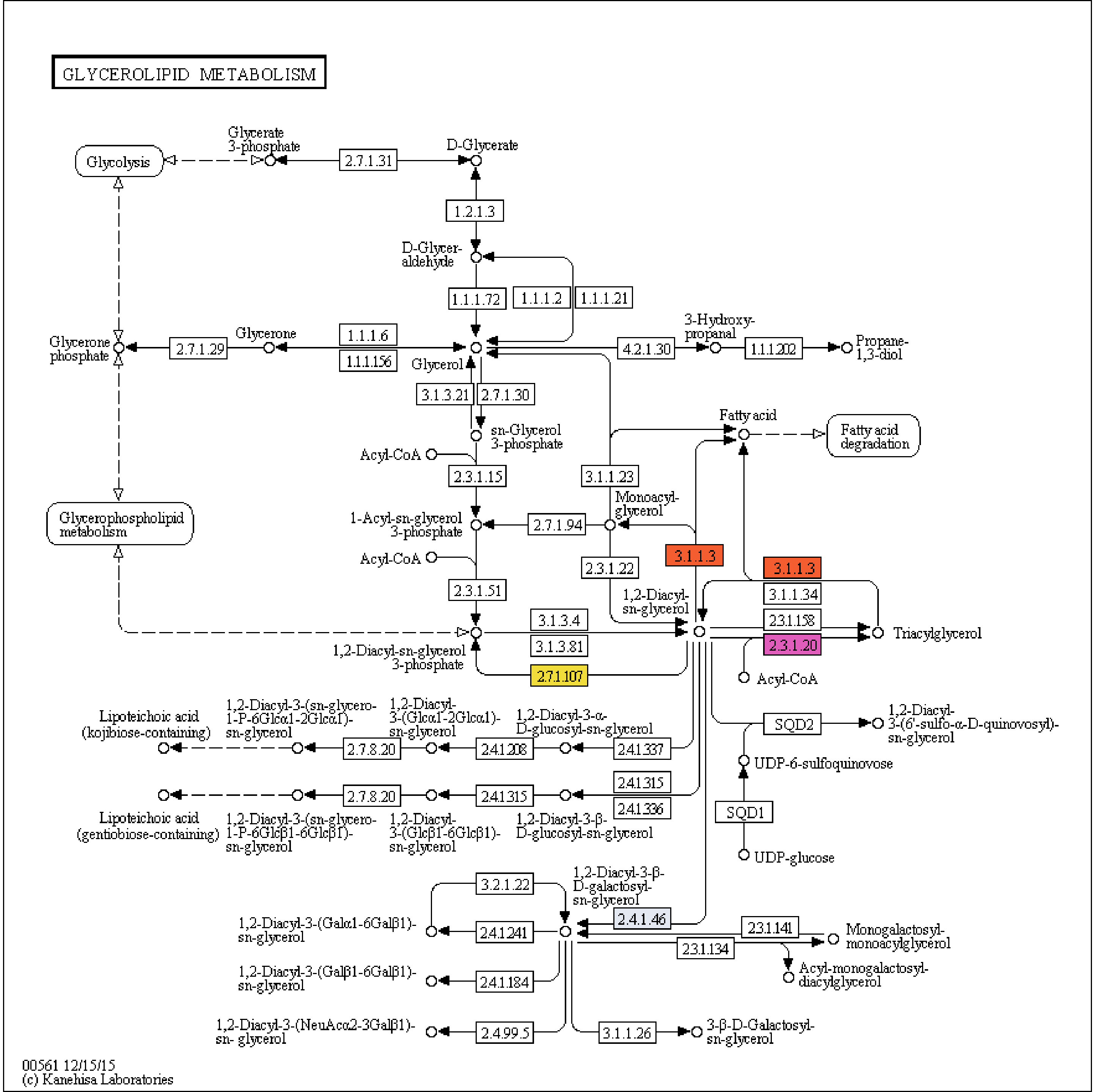
GLYCEROLIPID METABOLISM
Glycolysis
Glycerate 3-phosphate
2.7.1.31
D-Glycerate
1.2.1.3
D-Glycer-aldehyde
1.1.1.72
1.1.1.2
1.1.1.21
Glycerone phosphate
2.7.1.29
Glycerone
1.1.1.6
1.1.1.156
Glycerol
4.2.1.30
3-Hydroxy-propanal
1.1.1.202
Propane-1,3-diol
3.1.3.21
2.7.1.30
sn-Glycerol 3-phosphate
Fatty acid
Fatty acid degradation
Acyl-CoA
2.3.1.15
3.1.1.23
Monoacyl-glycerol
Glycerophospholipid metabolism
1-Acyl-sn-glycerol 3-phosphate
2.7.1.94
3.1.1.3
3.1.1.3
Acyl-CoA
2.3.1.22
2.3.1.51
1,2-Diacyl-sn-glycerol
3.1.1.34
3.1.3.4
2.3.1.158
3.1.3.81
2.3.1.20
Triacylglycerol
1,2-Diacyl-sn-glycerol 3-phosphate
2.7.1.107
Acyl-CoA
Lipoteichoic acid (kojibiose-containing)
1,2-Diacyl-3-(sn-glycero-1-P-6Glcα1-2Glcα1)-sn-glycerol
1,2-Diacyl-3-(Glcα1-2Glcα1)-sn-glycerol
1,2-Diacyl-3-α-D-glucosyl-sn-glycerol
SQD2
1,2-Diacyl-3-(6'-sulfo-α-D-quinovosyl)-sn-glycerol
2.7.8.20
2.4.1.208
2.4.1.337
UDP-6-sulfoquinovose
2.4.1.315
2.7.8.20
2.4.1.315
2.4.1.336
SQD1
Lipoteichoic acid (gentiobiose-containing)
1,2-Diacyl-3-(sn-glycero-1-P-6Glcβ1-6Glcβ1)-sn-glycerol
1,2-Diacyl-3-(Glcβ1-6Glcβ1)-sn-glycerol
1,2-Diacyl-3-β-D-glucosyl-sn-glycerol
UDP-glucose
3.2.1.22
1,2-Diacyl-3-β-D-galactosyl-sn-glycerol
1,2-Diacyl-3-(Galα1-6Galβ1)-sn-glycerol
2.4.1.241
2.4.1.46
2.3.1.141
Monogalactosyl-monoacylglycerol
2.3.1.134
Acyl-monogalactosyl-diacylglycerol
1,2-Diacyl-3-(Galβ1-6Galβ1)-sn-glycerol
2.4.1.184
1,2-Diacyl-3-(NeuAcα2-3Galβ1)-sn- glycerol
2.4.99.5
3.1.1.26
3-β-D-Galactosyl-sn-glycerol
00561 12/15/15
(c) Kanehisa Laboratories

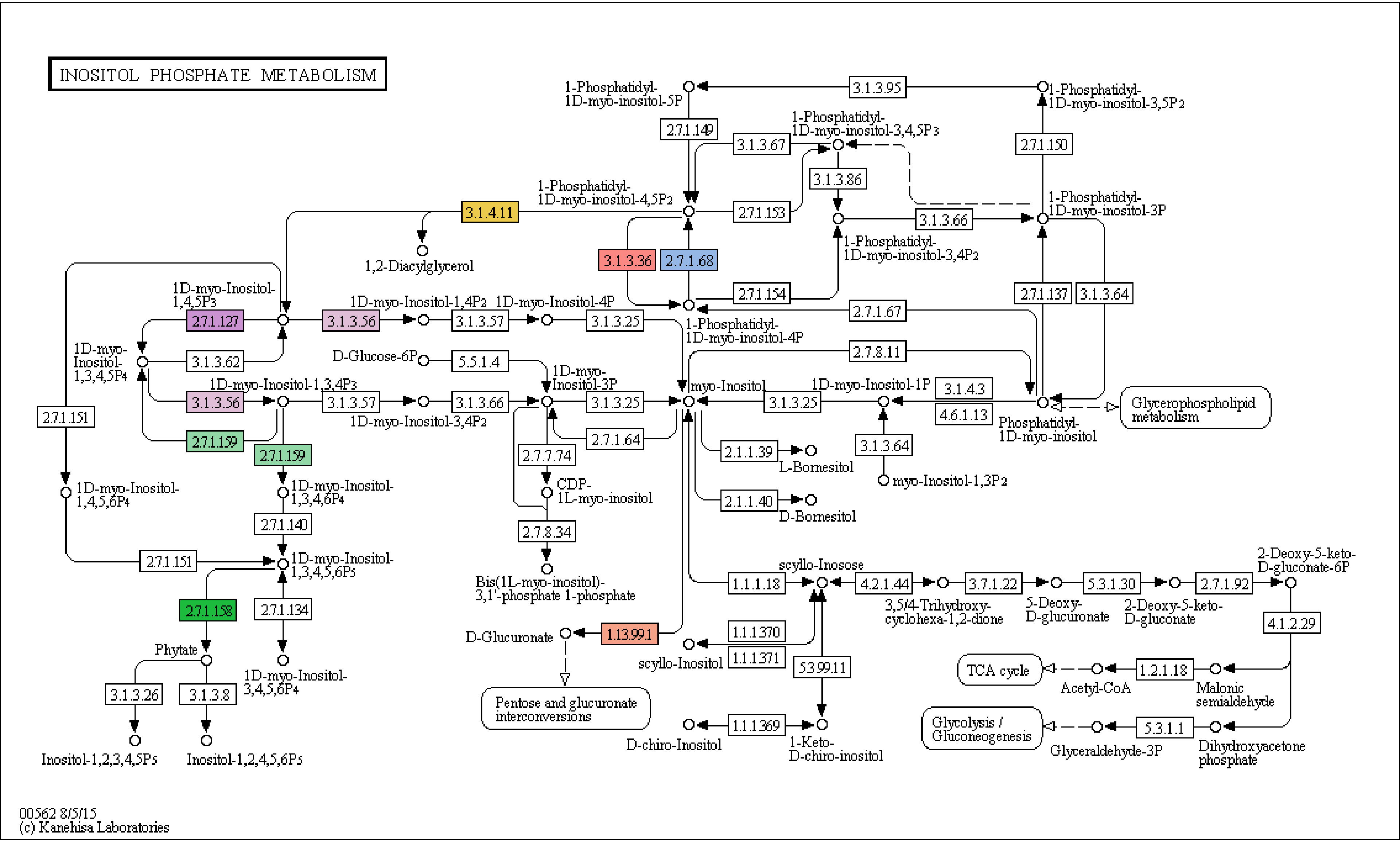
INOSITOL PHOSPHATE METABOLISM
1-Phosphatidyl-1D-myo-inositol-5P
3.1.3.95
1-Phosphatidyl-1D-myo-inositol-3,5P2
2.7.1.149
3.1.3.67
1-Phosphatidyl-1D-myo-inositol-3,4,5P3
2.7.1.150
3.1.3.86
1-Phosphatidyl-1D-myo-inositol-4,5P2
3.1.4.11
2.7.1.153
3.1.3.66
1-Phosphatidyl-1D-myo-inositol-3P
1,2-Diacylglycerol
3.1.3.36
2.7.1.68
1-Phosphatidyl-1D-myo-inositol-3,4P2
1D-myo-Inositol-1,4,5P3
2.7.1.154
2.7.1.137
3.1.3.64
1D-myo-Inositol-1,4P2
1D-myo-Inositol-4P
2.7.1.67
2.7.1.127
3.1.3.56
3.1.3.57
3.1.3.25
1-Phosphatidyl-1D-myo-inositol-4P
1D-myo-Inositol-1,3,4,5P4
3.1.3.62
D-Glucose-6P
5.5.1.4
2.7.8.11
1D-myo-Inositol-3P
myo-Inositol
1D-myo-Inositol-1P
3.1.4.3
1D-myo-Inositol-1,3,4P3
3.1.3.56
3.1.3.57
3.1.3.66
3.1.3.25
3.1.3.25
4.6.1.13
Glycerophospholipid metabolism
2.7.1.151
1D-myo-Inositol-3,4P2
Phosphatidyl-1D-myo-inositol
2.7.1.159
2.7.1.64
2.7.1.159
2.7.7.74
2.1.1.39
L-Bornesitol
3.1.3.64
myo-Inositol-1,3P2
1D-myo-Inositol-1,4,5,6P4
1D-myo-Inositol-1,3,4,6P4
CDP-1L-myo-inositol
2.1.1.40
D-Bornesitol
2.7.1.140
2.7.8.34
2.7.1.151
1D-myo-Inositol-1,3,4,5,6P5
scyllo-Inosose
2-Deoxy-5-keto-D-gluconate-6P
Bis(1L-myo-inositol)-3,1'-phosphate 1-phosphate
1.1.1.18
4.2.1.44
3.7.1.22
5.3.1.30
2.7.1.92
2.7.1.158
2.7.1.134
3,5/4-Trihydroxy-cyclohexa-1,2-dione
5-Deoxy-D-glucuronate
2-Deoxy-5-keto-D-gluconate
4.1.2.29
D-Glucuronate
1.13.99.1
1.1.1.370
Phytate
scyllo-Inositol
1.1.1.371
5.3.99.11
TCA cycle
Acetyl-CoA
1.2.1.18
Malonic semialdehyde
1D-myo-Inositol-3,4,5,6P4
3.1.3.26
3.1.3.8
Pentose and glucuronate interconversions
1.1.1.369
Glycolysis / Gluconeogenesis
5.3.1.1
D-chiro-Inositol
1-Keto-D-chiro-inositol
Glyceraldehyde-3P
Dihydroxyacetone phosphate
Inositol-1,2,3,4,5P5
Inositol-1,2,4,5,6P5
00562 8/5/15
(c) Kanehisa Laboratories

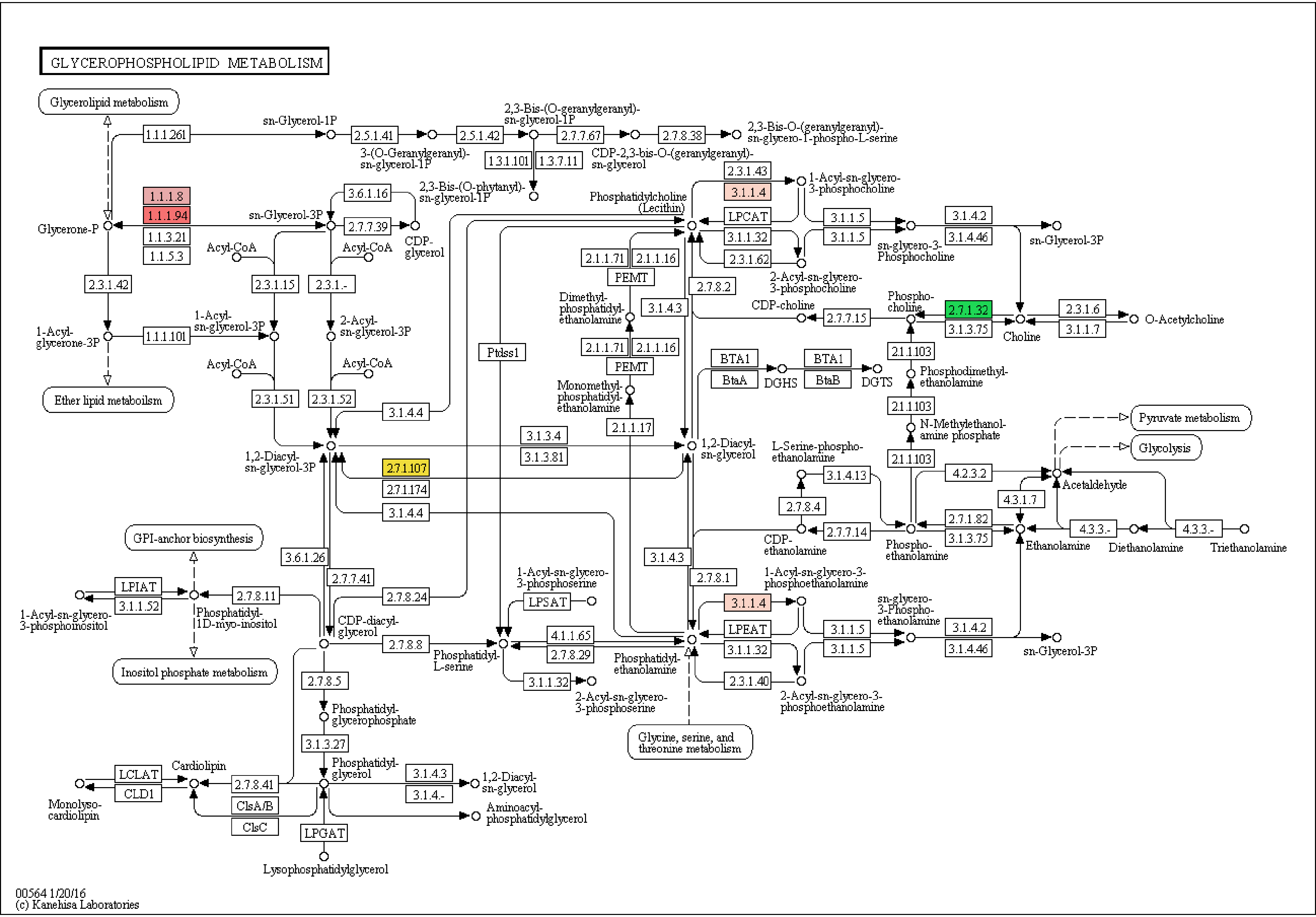
GLYCEROPHOSPHOLIPID METABOLISM
Glycerolipid metabolism
1.1.1.261
sn-Glycerol-1P
2.5.1.41
2.5.1.42
2,3-Bis-(O-geranylgeranyl)-sn-glycerol-1P
2.7.7.67
2.7.8.38
2,3-Bis-O-(geranylgeranyl)-sn-glycero-1-phospho-L-serine
3-(O-Geranylgeranyl)-sn-glycerol-1P
1.3.1.101
1.3.7.11
CDP-2,3-bis-O-(geranylgeranyl)-sn-glycerol
2,3-Bis-(O-phytanyl)-sn-glycerol-1P
1.1.1.8
1.1.1.94
1.1.3.21
1.1.5.3
Glycerone-P
sn-Glycerol-3P
3.6.1.16
2.7.7.39
CDP-glycerol
Acyl-CoA
Acyl-CoA
2.3.1.42
2.3.1.15
2.3.1.-
1-Acyl-glycerone-3P
1.1.1.101
1-Acyl-sn-glycerol-3P
2-Acyl-sn-glycerol-3P
Acyl-CoA
Acyl-CoA
Ether lipid metaboilsm
2.3.1.51
2.3.1.52
3.1.4.4
1,2-Diacyl-sn-glycerol-3P
3.1.3.4
3.1.3.81
2.7.1.107
2.7.1.174
3.1.4.4
Phosphatidylcholine (Lecithin)
2.3.1.43
3.1.1.4
1-Acyl-sn-glycero-3-phosphocholine
LPCAT
3.1.1.32
2.3.1.62
2-Acyl-sn-glycero-3-phosphocholine
3.1.1.5
3.1.1.5
sn-glycero-3-Phosphocholine
3.1.4.2
3.1.4.46
sn-Glycerol-3P
2.1.1.71
2.1.1.16
PEMT
Dimethyl-phosphatidyl-ethanolamine
3.1.4.3
2.7.8.2
CDP-choline
2.7.7.15
Phospho-choline
2.7.1.32
3.1.3.75
Choline
2.3.1.6
3.1.1.7
O-Acetylcholine
Ptdss1
2.1.1.71
2.1.1.16
PEMT
Monomethyl-phosphatidyl-ethanolamine
2.1.1.17
BTA1
BtaA
DGHS
BTA1
BtaB
DGTS
2.1.1.103
Phosphodimethyl-ethanolamine
2.1.1.103
N-Methylethanol-amine phosphate
2.1.1.103
1,2-Diacyl-sn-glycerol
L-Serine-phospho-ethanolamine
3.1.4.13
2.7.8.4
4.2.3.2
Pyruvate metabolism
Glycolysis
Acetaldehyde
4.3.1.7
2.7.1.82
3.1.3.75
CDP-ethanolamine
2.7.7.14
Phospho-ethanolamine
Ethanolamine
4.3.3.-
Diethanolamine
4.3.3.-
Triethanolamine
GPI-anchor biosynthesis
3.6.1.26
2.7.7.41
3.1.4.3
2.7.8.1
LPIAT
3.1.1.52
2.7.8.11
1-Acyl-sn-glycero-3-phosphoinositol
Phosphatidyl-1D-myo-inositol
Inositol phosphate metabolism
2.7.8.24
CDP-diacyl-glycerol
1-Acyl-sn-glycero-3-phosphoserine
LPSAT
2.7.8.8
Phosphatidyl-L-serine
4.1.1.65
2.7.8.29
Phosphatidyl-ethanolamine
3.1.1.32
2-Acyl-sn-glycero-3-phosphoserine
1-Acyl-sn-glycero-3-phosphoethanolamine
3.1.1.4
LPEAT
3.1.1.32
2.3.1.40
2-Acyl-sn-glycero-3-phosphoethanolamine
sn-glycero-3-Phospho-ethanolamine
3.1.1.5
3.1.1.5
3.1.4.2
3.1.4.46
sn-Glycerol-3P
2.7.8.5
Phosphatidyl-glycerophosphate
3.1.3.27
Glycine, serine, and threonine metabolism
LCLAT
CLD1
Cardiolipin
2.7.8.41
Monolyso-cardiolipin
ClsA/B
ClsC
Phosphatidyl-glycerol
3.1.4.3
3.1.4.-
1,2-Diacyl-sn-glycerol
Aminoacyl-phosphatidylglycerol
LPGAT
Lysophosphatidylglycerol
00564 1/20/16
(c) Kanehisa Laboratories

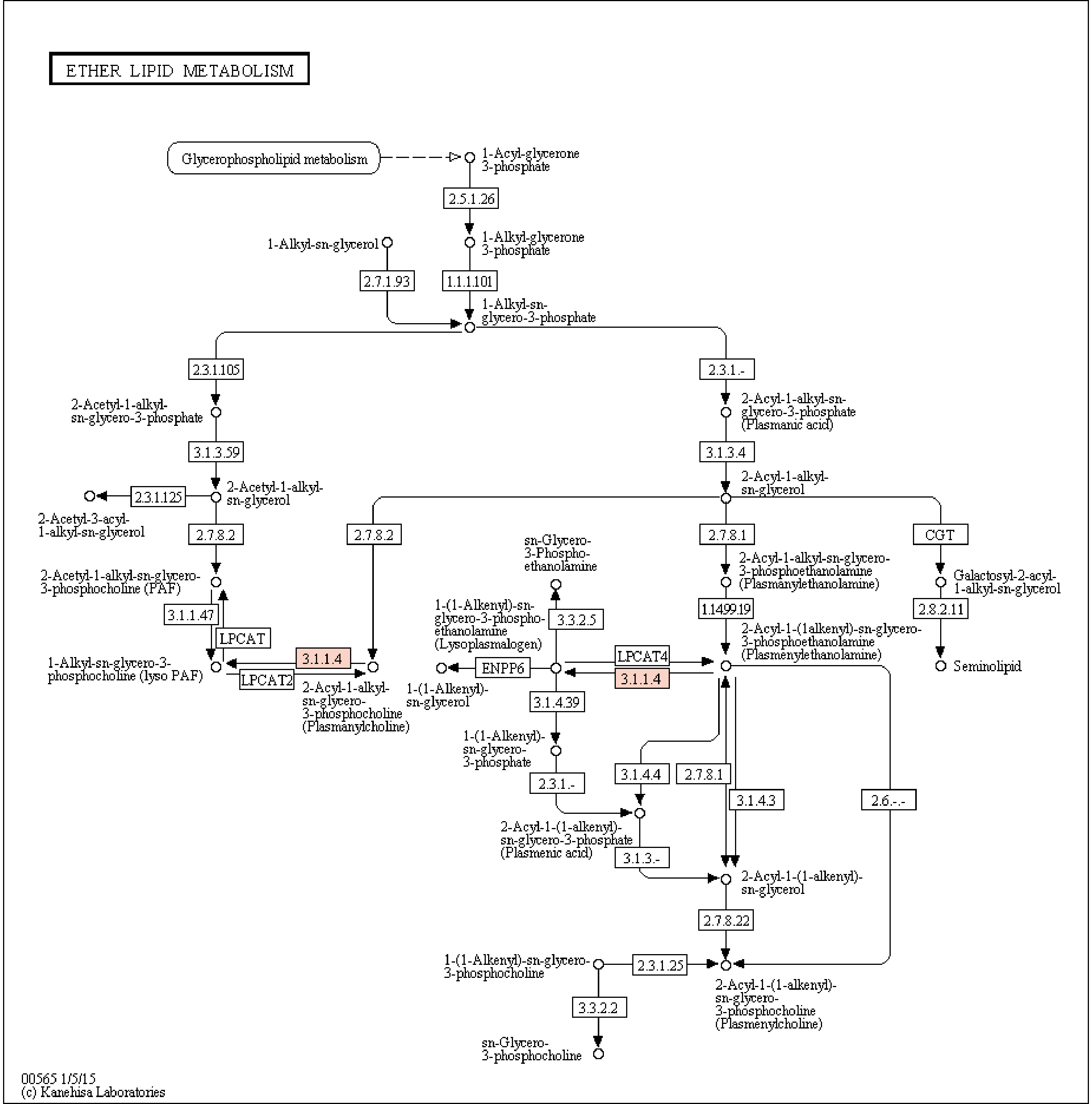

ETHER LIPID METABOLISM
Glycerophospholipid metabolism
1-Acyl-glycerone 3-phosphate
2.5.1.26
1-Alkyl-sn-glycerol
1-Alkyl-glycerone 3-phosphate
2.7.1.93
1.1.1.101
1-Alkyl-sn-glycero-3-phosphate
2.3.1.105
2.3.1.-
2-Acetyl-1-alkyl-sn-glycero-3-phosphate
2-Acyl-1-alkyl-sn-glycero-3-phosphate (Plasmanic acid)
3.1.3.59
3.1.3.4
2-Acetyl-1-alkyl-sn-glycerol
2-Acyl-1-alkyl-sn-glycerol
2.3.1.125
2-Acetyl-3-acyl-1-alkyl-sn-glycerol
2.7.8.2
2.7.8.2
2.7.8.1
CGT
sn-Glycero-3-Phospho-ethanolamine
2-Acyl-1-alkyl-sn-glycero-3-phosphoethanolamine (Plasmanylethanolamine)
Galactosyl-2-acyl-1-alkyl-sn-glycerol
2-Acetyl-1-alkyl-sn-glycero-3-phosphocholine (PAF)
3.1.1.47
LPCAT
1-(1-Alkenyl)-sn-glycero-3-phospho-ethanolamine (Lysoplasmalogen)
3.3.2.5
1.14.99.19
2.8.2.11
2-Acyl-1-(1alkenyl)-sn-glycero-3-phosphoethanolamine (Plasmenylethanolamine)
3.1.1.4
LPCAT4
1-Alkyl-sn-glycero-3-phosphocholine (lyso PAF)
ENPP6
3.1.1.4
Seminolipid
LPCAT2
2-Acyl-1-alkyl-sn-glycero-3-phosphocholine (Plasmanylcholine)
1-(1-Alkenyl)-sn-glycerol
3.1.4.39
1-(1-Alkenyl)-sn-glycero-3-phosphate
3.1.4.4
2.7.8.1
2.3.1.-
3.1.4.3
2.6.-.-
2-Acyl-1-(1-alkenyl)-sn-glycero-3-phosphate (Plasmenic acid)
3.1.3.-
2-Acyl-1-(1-alkenyl)-sn-glycerol
2.7.8.22
1-(1-Alkenyl)-sn-glycero-3-phosphocholine
2.3.1.25
3.3.2.2
2-Acyl-1-(1-alkenyl)-sn-glycero-3-phosphocholine (Plasmenylcholine)
sn-Glycero-3-phosphocholine
00565 1/5/15
(c) Kanehisa Laboratories

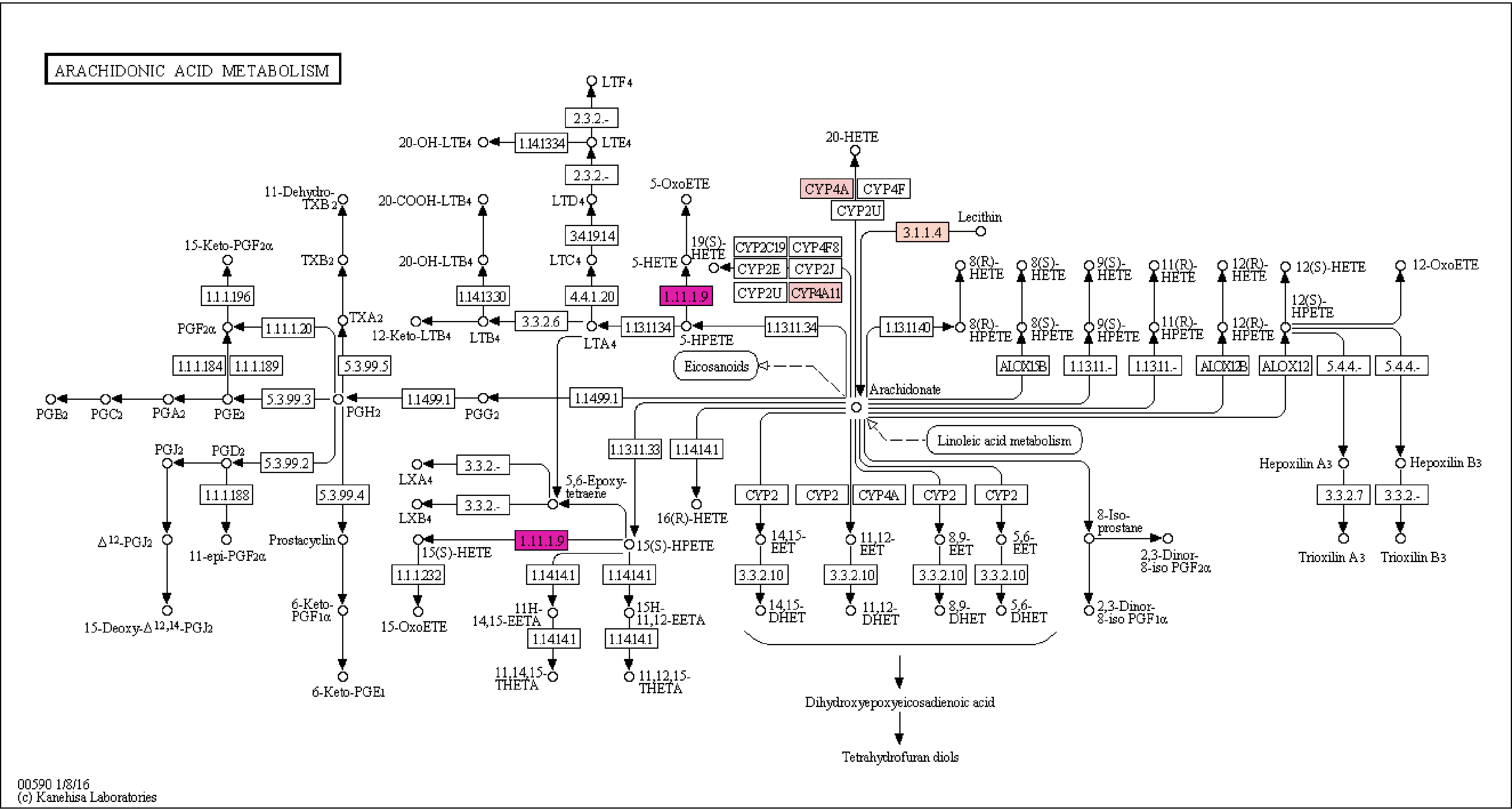
ARACHIDONIC ACID METABOLISM
LTF4
2.3.2.-
20-OH-LTE4
1.14.13.34
LTE4
2.3.2.-
LTD4
3.4.19.14
LTC4
4.4.1.20
20-COOH-LTB4
20-OH-LTB4
1.14.13.30
12-Keto-LTB4
3.3.2.6
LTB4
LTA4
1.13.11.34
5-OxoETE
5-HETE
1.11.1.9
5-HPETE
19(S)-HETE
CYP2C19
CYP4F8
CYP2E
CYP2J
CYP2U
CYP4A11
1.13.11.34
Eicosanoids
20-HETE
CYP4A
CYP4F
CYP2U
Lecithin
3.1.1.4
Arachidonate
Linoleic acid metabolism
11-Dehydro-TXB2
TXB2
TXA2
15-Keto-PGF2α
1.1.1.196
PGF2α
1.1.1.20
1.1.1.184
1.1.1.189
5.3.99.5
PGB2
PGC2
PGA2
PGE2
5.3.99.3
PGH2
1.14.99.1
PGG2
1.14.99.1
PGJ2
PGD2
5.3.99.2
1.1.1.188
5.3.99.4
Δ12-PGJ2
11-epi-PGF2α
Prostacyclin
15-Deoxy-Δ12,14-PGJ2
6-Keto-PGF1α
6-Keto-PGE1
LXA4
3.3.2.-
LXB4
3.3.2.-
5,6-Epoxy-tetraene
1.11.1.9
15(S)-HETE
15(S)-HPETE
1.1.1.232
15-OxoETE
1.14.14.1
1.14.14.1
11H-14,15-EETA
15H-11,12-EETA
1.14.14.1
1.14.14.1
11,14,15-THETA
11,12,15-THETA
1.13.11.33
1.14.14.1
16(R)-HETE
1.13.11.40
8(R)-HETE
8(R)-HPETE
8(S)-HETE
8(S)-HPETE
ALOX15B
9(S)-HETE
9(S)-HPETE
1.13.11.-
11(R)-HETE
11(R)-HPETE
1.13.11.-
12(R)-HETE
12(R)-HPETE
ALOX12B
12(S)-HETE
12(S)-HPETE
ALOX12
12-OxoETE
5.4.4.-
5.4.4.-
Hepoxilin A3
Hepoxilin B3
3.3.2.7
3.3.2.-
Trioxilin A3
Trioxilin B3
CYP2
CYP2
CYP4A
CYP2
CYP2
14,15-EET
11,12-EET
8,9-EET
5,6-EET
3.3.2.10
3.3.2.10
3.3.2.10
3.3.2.10
14,15-DHET
11,12-DHET
8,9-DHET
5,6-DHET
8-Iso-prostane
2,3-Dinor-8-iso PGF2α
2,3-Dinor-8-iso PGF1α
Dihydroxyepoxyeicosadienoic acid
Tetrahydrofuran diols
00590 1/8/16
(c) Kanehisa Laboratories

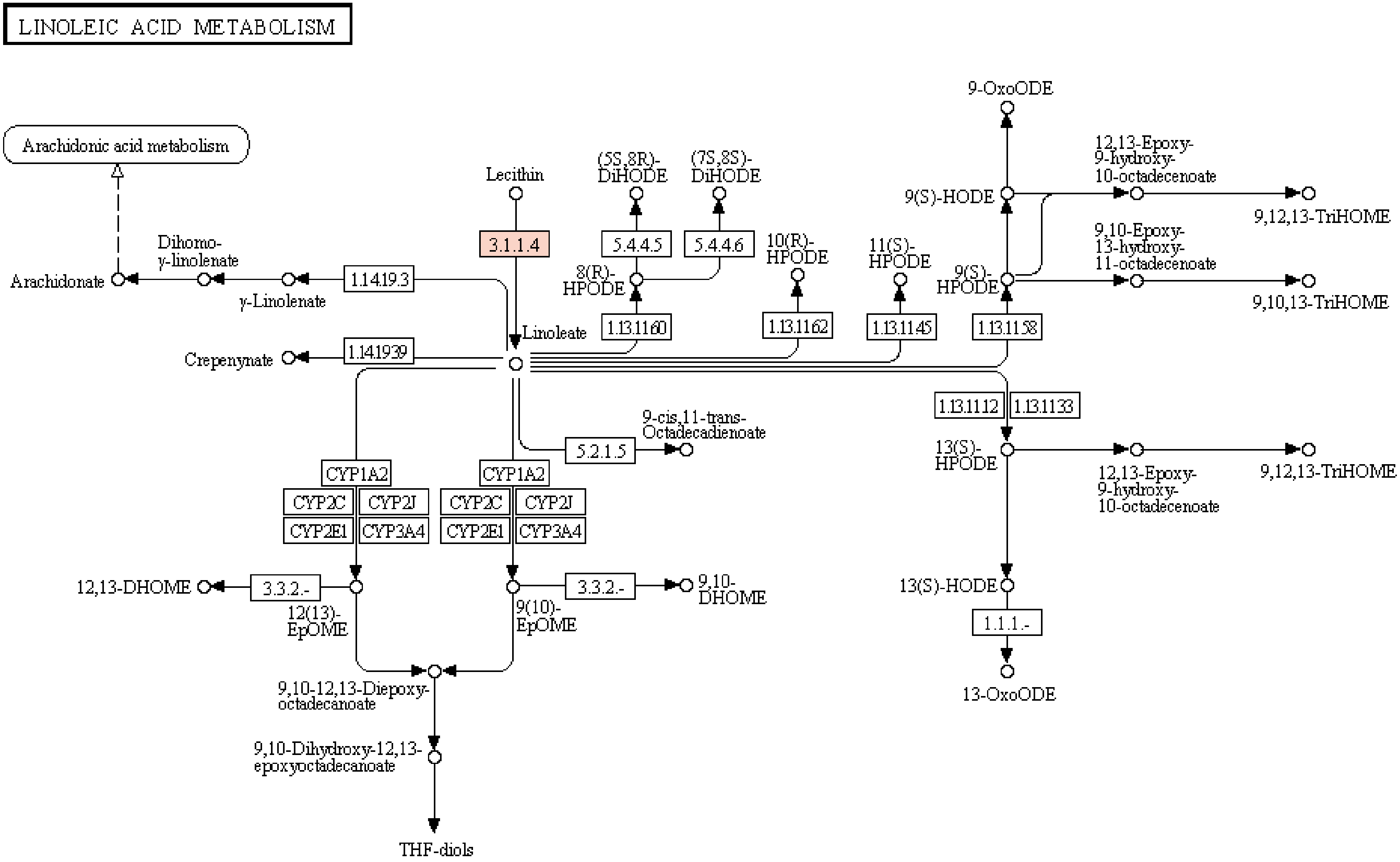

00591 12/14/15

(c) Kanehisa Laboratories

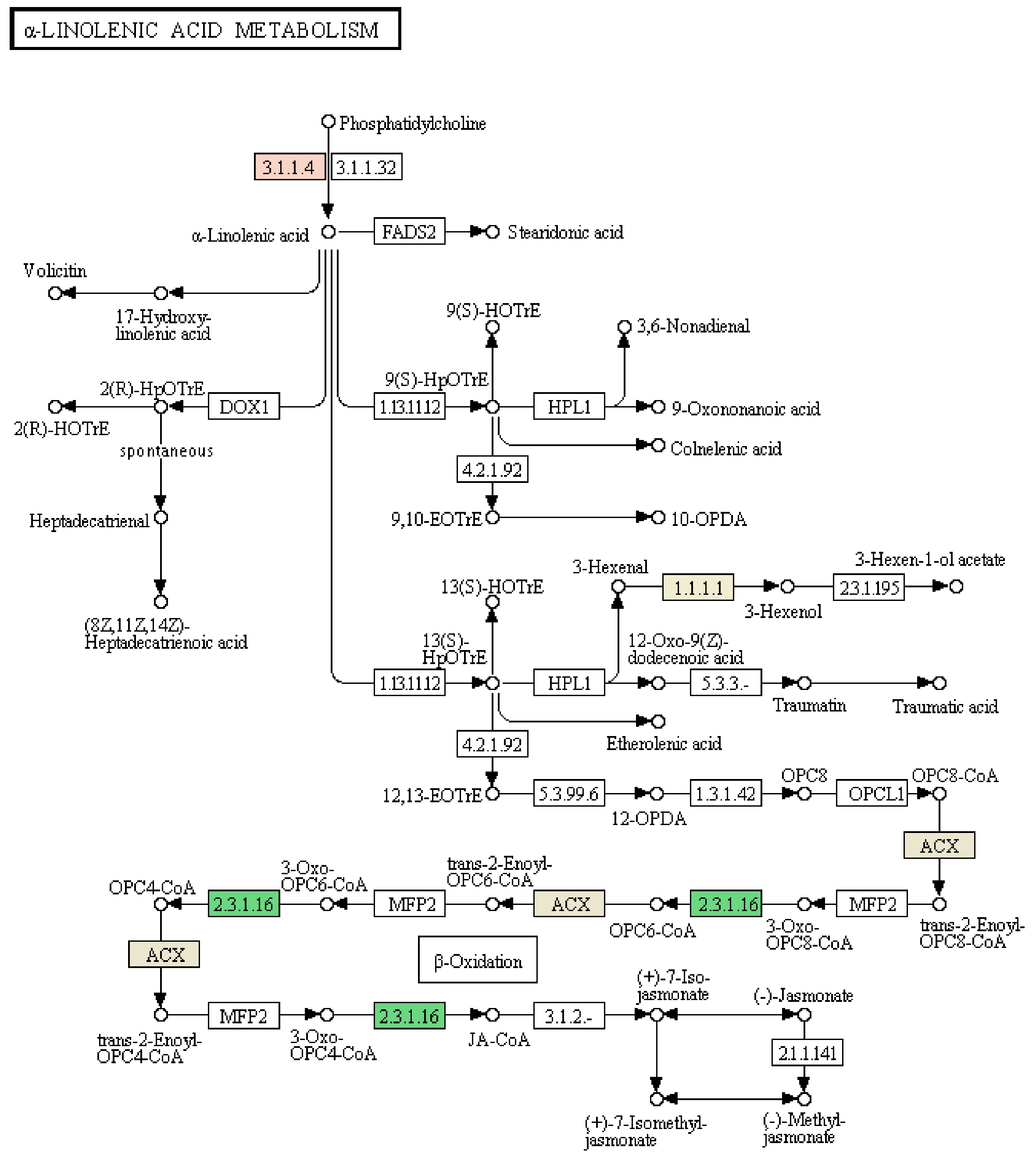

00592 1/6/15
(c) Kanehisa Laboratories

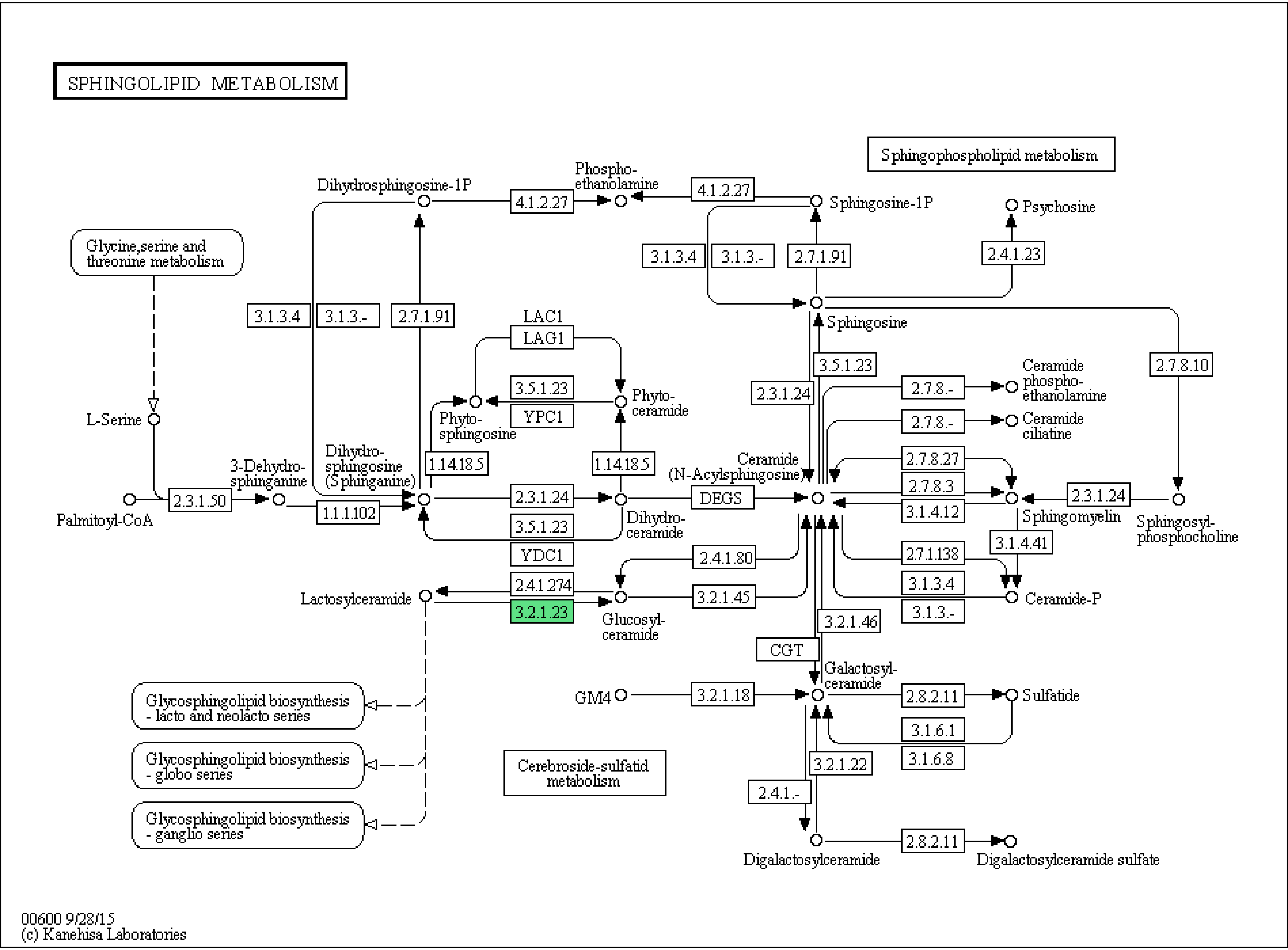
SPHINGOLIPID METABOLISM
Sphingophospholipid metabolism
Glycine,serine and threonine metabolism
L-Serine
Palmitoyl-CoA
2.3.1.50
3-Dehydro-sphinganine
1.1.1.102
Dihydro-sphingosine (Sphinganine)
Dihydrosphingosine-1P
3.1.3.4
3.1.3.-
2.7.1.91
4.1.2.27
Phospho-ethanolamine
4.1.2.27
Sphingosine-1P
Psychosine
3.1.3.4
3.1.3.-
2.7.1.91
2.4.1.23
Sphingosine
LAC1
LAG1
3.5.1.23
YPC1
Phyto-sphingosine
Phyto-ceramide
1.14.18.5
1.14.18.5
2.3.1.24
3.5.1.23
YDC1
Dihydro-ceramide
DEGS
Ceramide (N-Acylsphingosine)
3.5.1.23
2.3.1.24
2.7.8.-
Ceramide phospho-ethanolamine
2.7.8.-
Ceramide ciliatine
2.7.8.10
2.7.8.27
2.7.8.3
3.1.4.12
Sphingomyelin
2.3.1.24
Sphingosyl-phosphocholine
2.7.1.138
3.1.4.41
3.1.3.4
3.1.3.-
Ceramide-P
2.4.1.80
Lactosylceramide
2.4.1.274
3.2.1.23
Glucosyl-ceramide
3.2.1.45
3.2.1.46
CGT
Galactosyl-ceramide
GM4
3.2.1.18
2.8.2.11
Sulfatide
3.1.6.1
3.1.6.8
3.2.1.22
2.4.1.-
Digalactosylceramide
2.8.2.11
Digalactosylceramide sulfate
Glycosphingolipid biosynthesis - lacto and neolacto series
Glycosphingolipid biosynthesis - globo series
Glycosphingolipid biosynthesis - ganglio series
Cerebroside-sulfatid metabolism
00600 9/28/15
(c) Kanehisa Laboratories

GLYCOSPHINGOLIPID BIOSYNTHESIS - LACTO AND NEO-LACTOSERIES

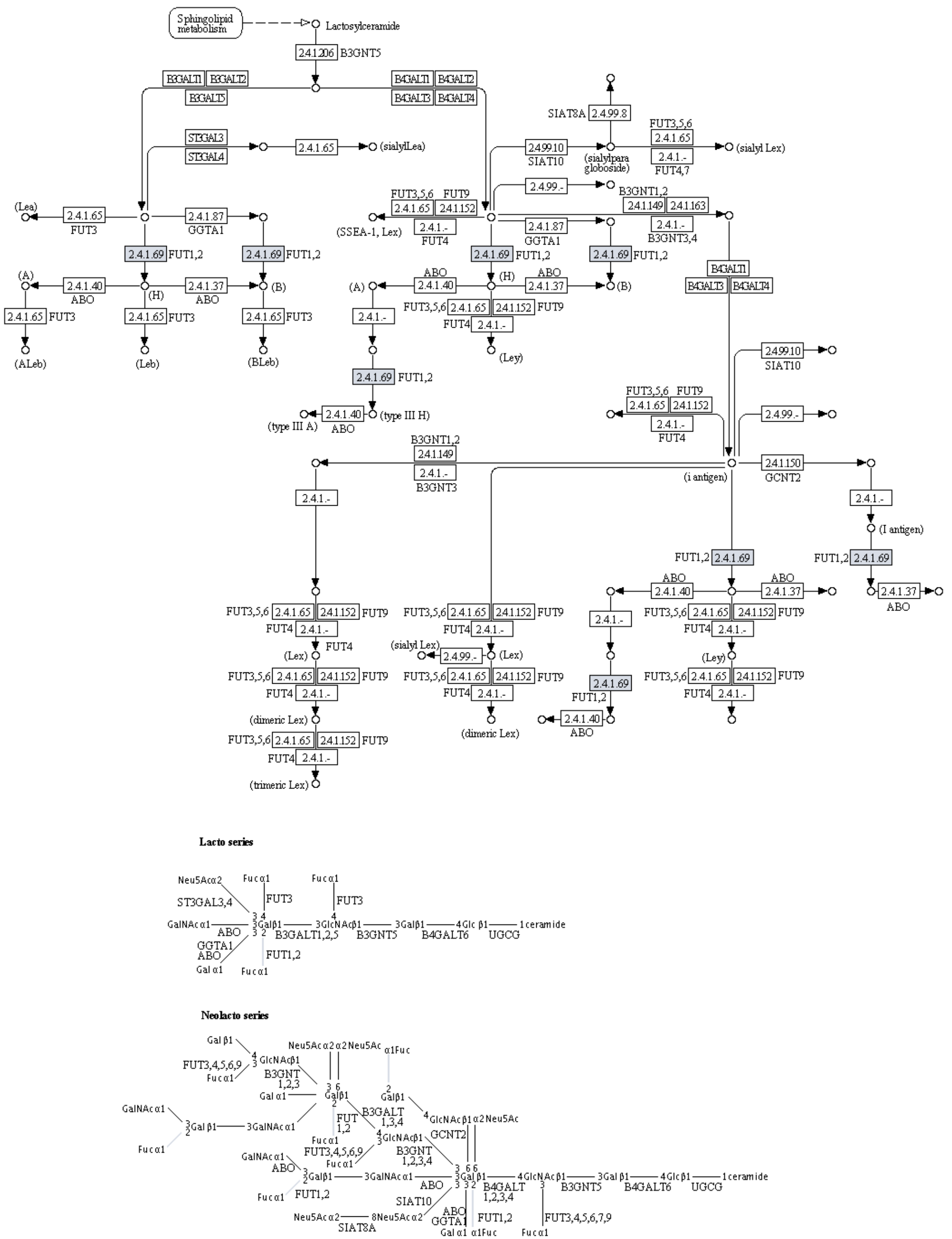

00601 11/14/14

(c) Kanehisa Laboratories

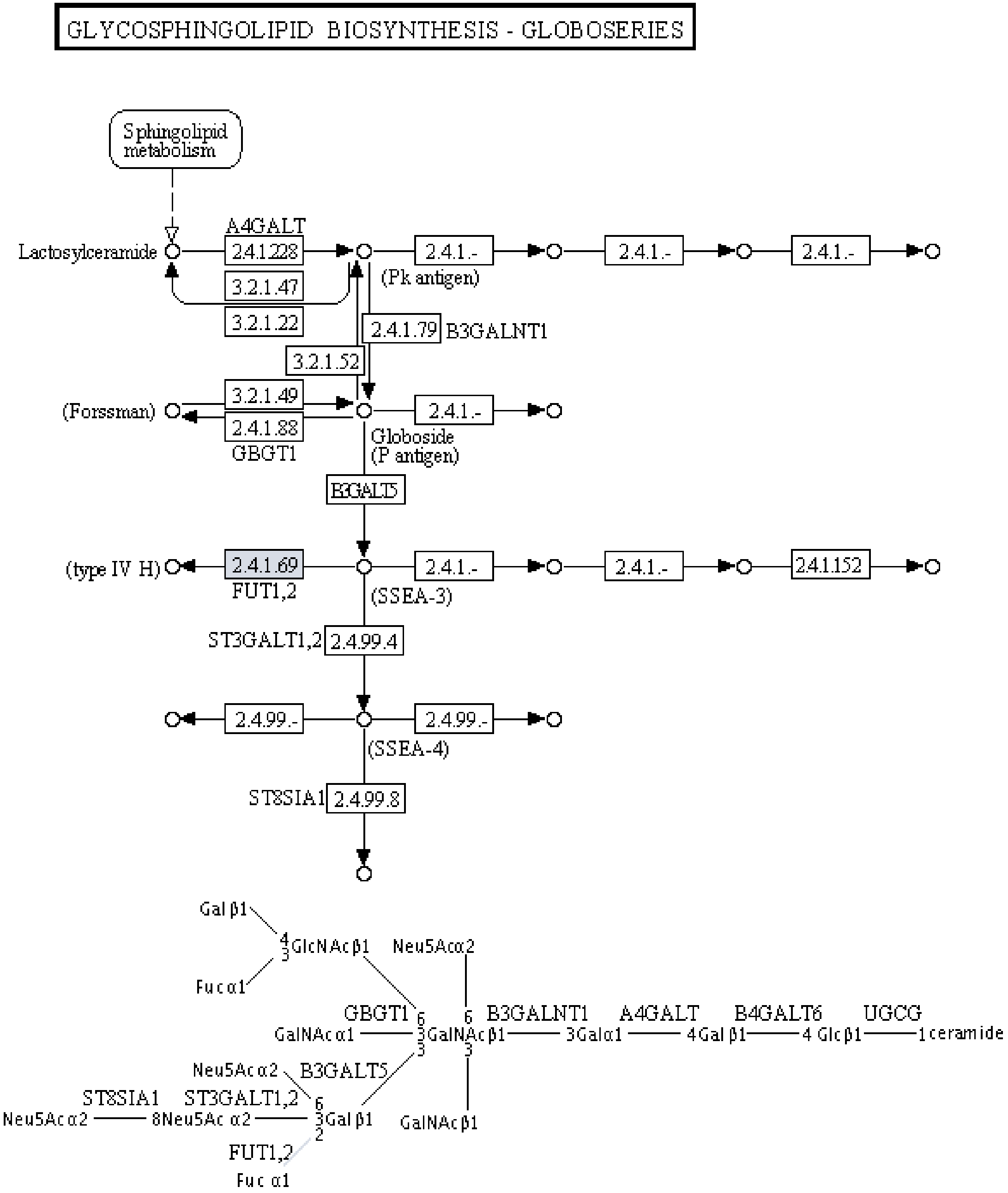

00603 6/6/12

(c) Kanehisa Laboratories

GLYCOSPHINGOLIPID BIOSYNTHESIS - GANGLIO SERIES

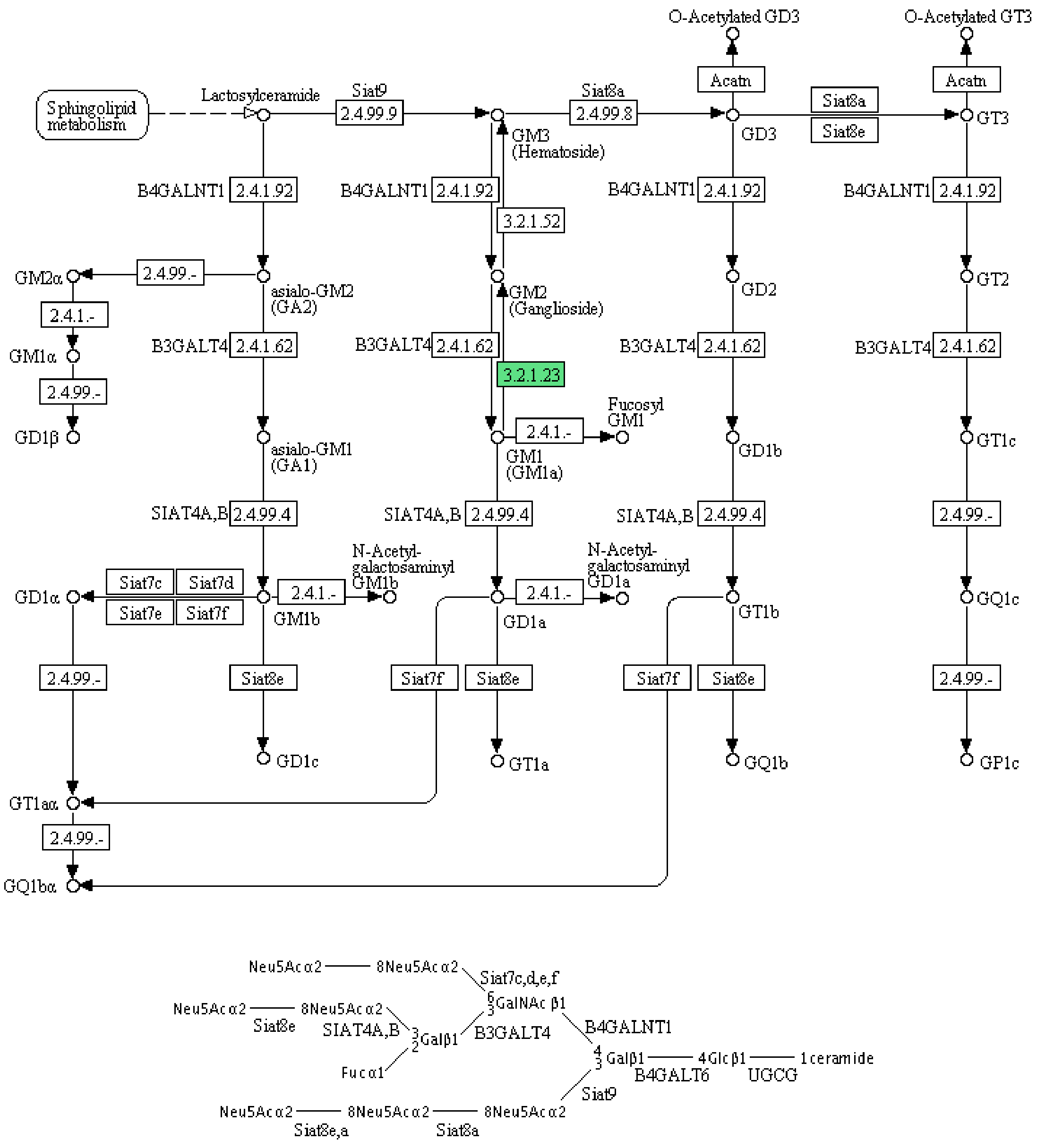

00604 7/2/12

(c) Kanehisa Laboratories

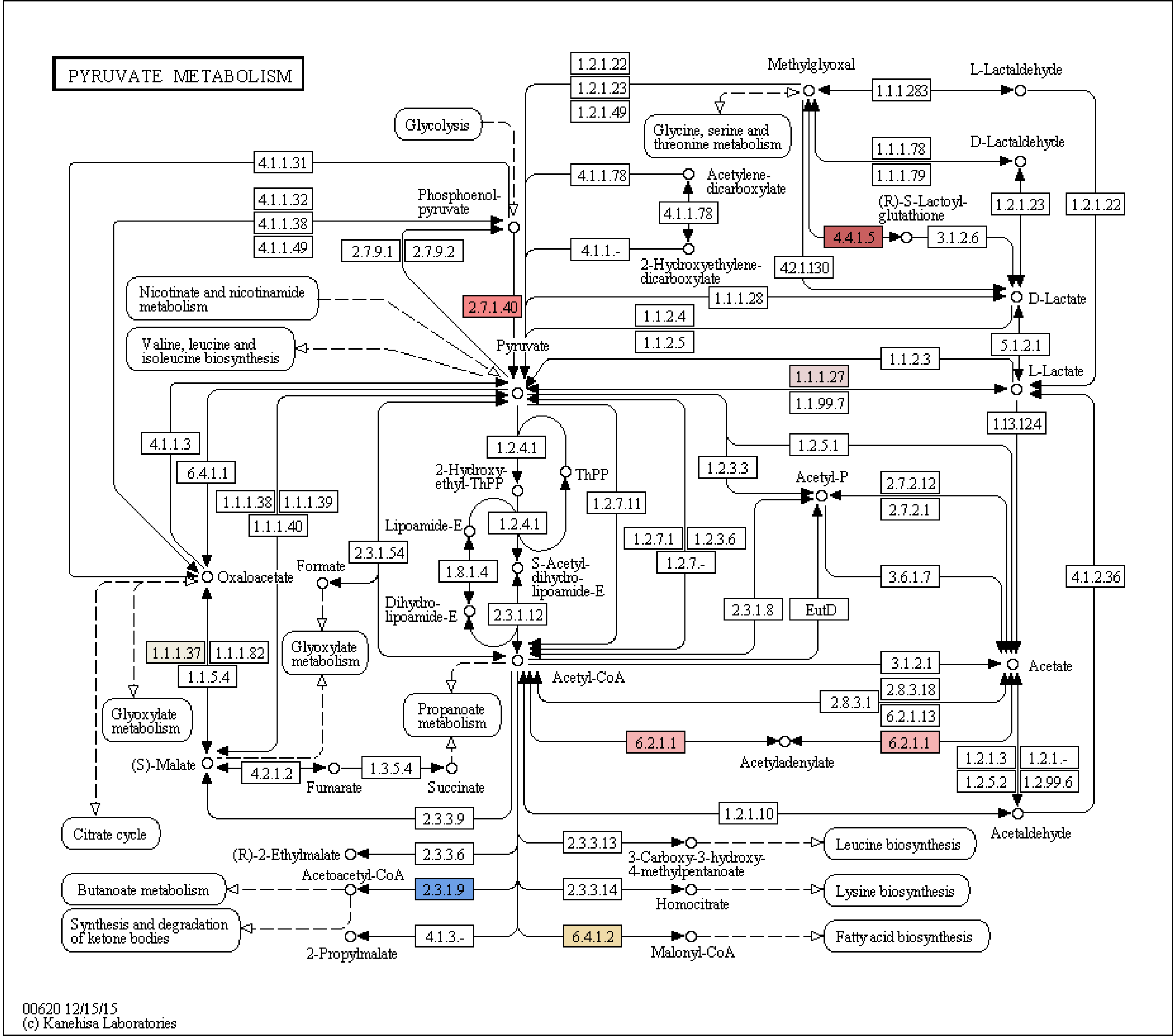
PYRUVATE METABOLISM
Glycolysis
Phosphoenol-pyruvate
Pyruvate
Methylglyoxal
L-Lactaldehyde
D-Lactaldehyde
Glycine, serine and threonine metabolism
Acetylene-dicarboxylate
2-Hydroxyethylene-dicarboxylate
(R)-S-Lactoyl-glutathione
D-Lactate
L-Lactate
Nicotinate and nicotinamide metabolism
Valine, leucine and isoleucine biosynthesis
Oxaloacetate
Formate
Glyoxylate metabolism
2-Hydroxy-ethyl-ThPP
ThPP
Lipoamide-E
S-Acetyl-dihydro-lipoamide-E
Dihydro-lipoamide-E
Acetyl-P
Acetyl-CoA
Acetate
Acetyladenylate
Acetaldehyde
Propanoate metabolism
(S)-Malate
Fumarate
Succinate
Citrate cycle
(R)-2-Ethylmalate
Acetoacetyl-CoA
2-Propylmalate
Butanoate metabolism
Synthesis and degradation of ketone bodies
3-Carboxy-3-hydroxy-4-methylpentanoate
Homocitrate
Malonyl-CoA
Leucine biosynthesis
Lysine biosynthesis
Fatty acid biosynthesis
1.2.1.22
1.2.1.23
1.2.1.49
1.1.1.283
1.1.1.78
1.1.1.79
4.1.1.31
4.1.1.32
4.1.1.38
4.1.1.49
4.1.1.78
4.1.1.-
2.7.9.1
2.7.9.2
2.7.1.40
4.4.1.5
3.1.2.6
4.2.1.130
1.1.1.28
1.1.2.4
1.1.2.5
1.1.2.3
5.1.2.1
1.1.1.27
1.1.99.7
1.13.12.4
4.1.1.3
6.4.1.1
1.1.1.38
1.1.1.39
1.1.1.40
1.2.4.1
1.2.5.1
1.2.3.3
2.7.2.12
2.7.2.1
1.2.7.11
1.2.7.1
1.2.3.6
1.2.7.-
2.3.1.54
1.8.1.4
2.3.1.12
3.6.1.7
4.1.2.36
2.3.1.8
EutD
1.1.1.37
1.1.1.82
1.1.5.4
3.1.2.1
2.8.3.18
2.8.3.1
6.2.1.13
6.2.1.1
1.2.1.3
1.2.1.-
1.2.5.2
1.2.99.6
1.2.1.10
4.2.1.2
1.3.5.4
2.3.3.9
2.3.3.6
2.3.1.9
4.1.3.-
2.3.3.13
2.3.3.14
6.4.1.2
00620 12/15/15
(c) Kanehisa Laboratories

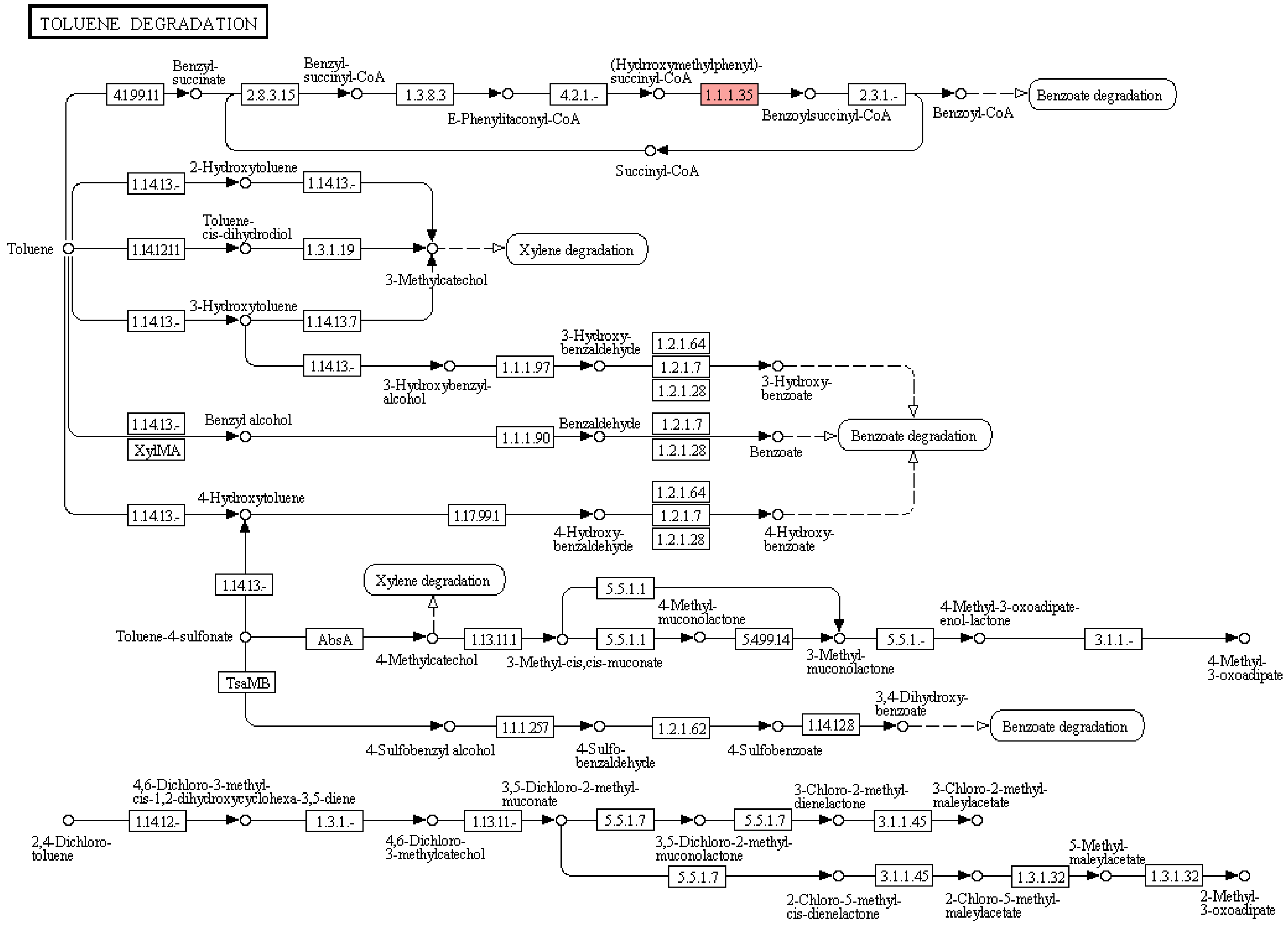

00623 10/15/15
(c) Kanehisa Laboratories

CHLOROALKANE AND CHLOROALKENE DEGRADATION

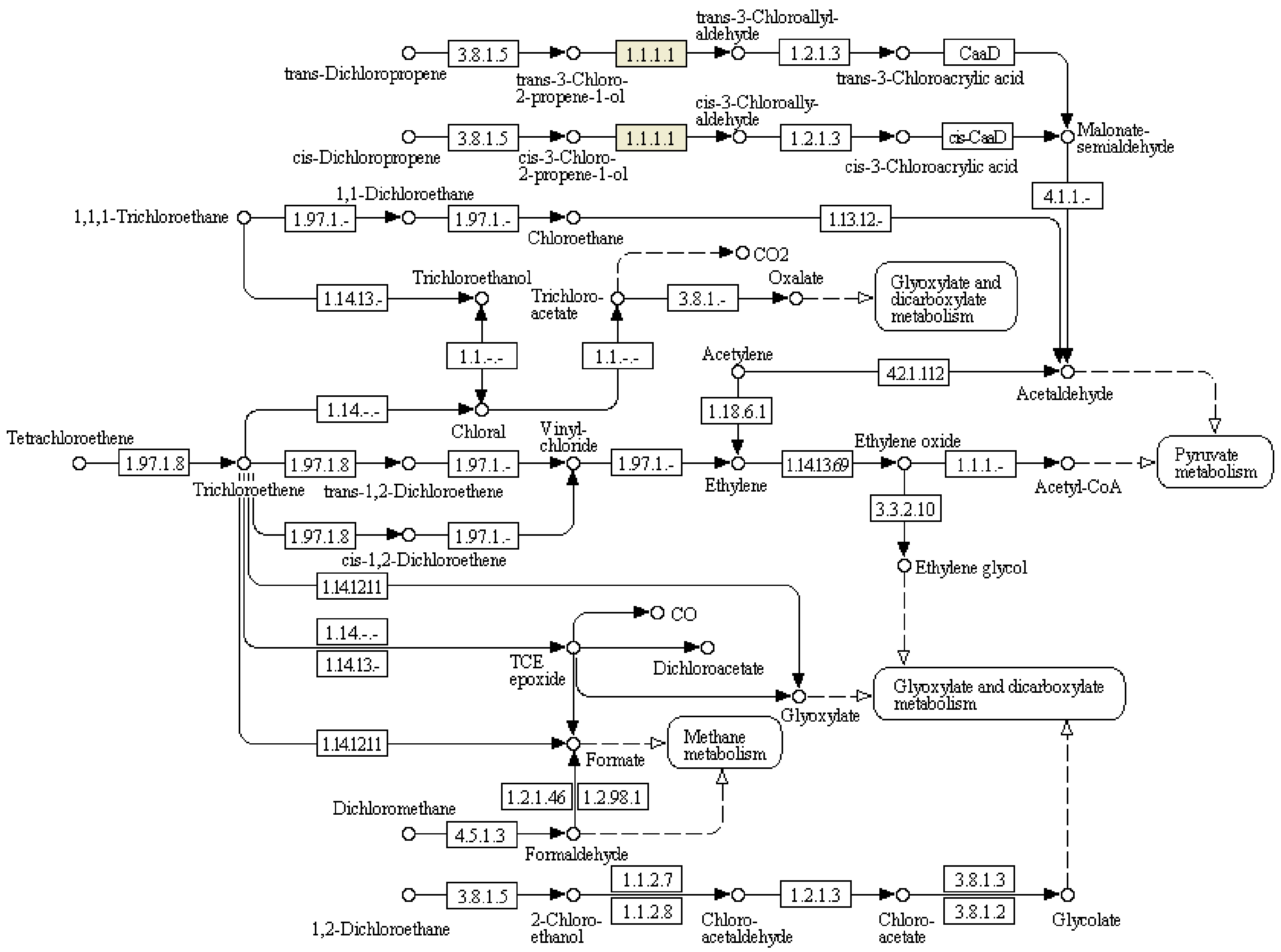

00625 6/8/15

(c) Kanehisa Laboratories

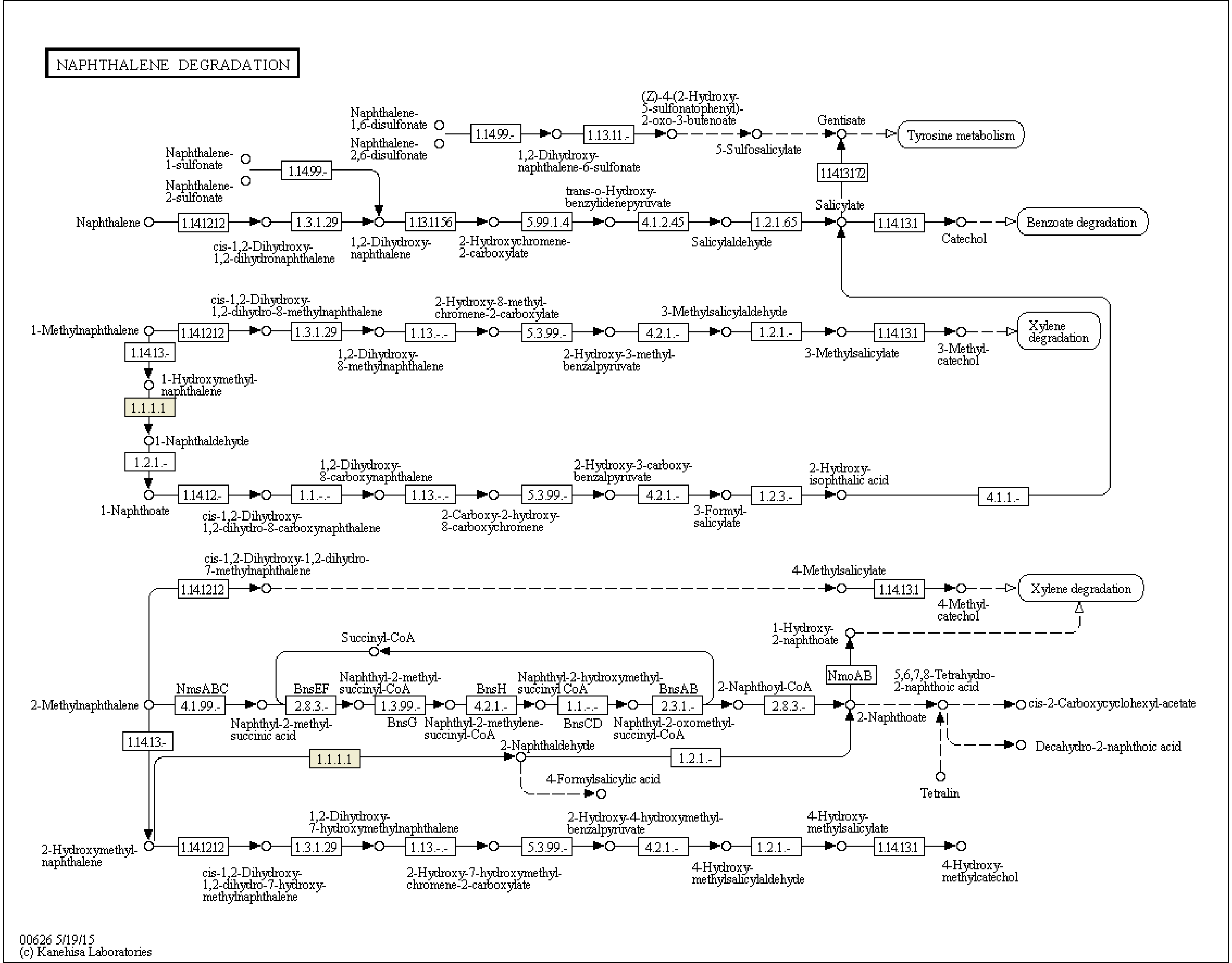
NAPHTHALENE DEGRADATION
Naphthalene-1,6-disulfonate
Naphthalene-2,6-disulfonate
1.14.99.-
1,2-Dihydroxy-naphthalene-6-sulfonate
1.13.11.-
(Z)-4-(2-Hydroxy-5-sulfonatophenyl)-2-oxo-3-butenoate
5-Sulfosalicylate
Gentisate
Tyrosine metabolism
1.14.13.172
Naphthalene-1-sulfonate
Naphthalene-2-sulfonate
1.14.99.-
Naphthalene
1.14.12.12
cis-1,2-Dihydroxy-1,2-dihydronaphthalene
1.3.1.29
1,2-Dihydroxy-naphthalene
1.13.11.56
2-Hydroxychromene-2-carboxylate
5.99.1.4
trans-o-Hydroxy-benzylidenepyruvate
4.1.2.45
Salicylaldehyde
1.2.1.65
Salicylate
1.14.13.1
Catechol
Benzoate degradation
1-Methylnaphthalene
1.14.12.12
cis-1,2-Dihydroxy-1,2-dihydro-8-methylnaphthalene
1.3.1.29
1,2-Dihydroxy-8-methylnaphthalene
1.13.-.-
2-Hydroxy-8-methyl-chromene-2-carboxylate
5.3.99.-
2-Hydroxy-3-methyl-benzalpyruvate
4.2.1.-
3-Methylsalicylaldehyde
1.2.1.-
3-Methylsalicylate
1.14.13.1
3-Methyl-catechol
Xylene degradation
1.14.13.-
1-Hydroxymethyl-naphthalene
1.1.1.1
1-Naphthaldehyde
1.2.1.-
1-Naphthoate
1.14.12.-
cis-1,2-Dihydroxy-1,2-dihydro-8-carboxynaphthalene
1.1.-.-
1,2-Dihydroxy-8-carboxynaphthalene
1.13.-.-
2-Carboxy-2-hydroxy-8-carboxychromene
5.3.99.-
2-Hydroxy-3-carboxy-benzalpyruvate
4.2.1.-
3-Formyl-salicylate
1.2.3.-
2-Hydroxy-isophthalic acid
4.1.1.-
1.14.12.12
cis-1,2-Dihydroxy-1,2-dihydro-7-methylnaphthalene
4-Methylsalicylate
1.14.13.1
4-Methyl-catechol
Xylene degradation
1-Hydroxy-2-naphthoate
Succinyl-CoA
2-Methylnaphthalene
NmsABC
4.1.99.-
Naphthyl-2-methyl-succinic acid
BnsEF
2.8.3.-
Naphthyl-2-methyl-succinyl-CoA
1.3.99.-
BnsG
Naphthyl-2-methylene-succinyl-CoA
BnsH
4.2.1.-
Naphthyl-2-hydroxymethyl-succinyl CoA
1.1.-.-
BnsCD
Naphthyl-2-oxomethyl-succinyl-CoA
BnsAB
2.3.1.-
2-Naphthoyl-CoA
2.8.3.-
NmoAB
2-Naphthoate
5,6,7,8-Tetrahydro-2-naphthoic acid
cis-2-Carboxycyclohexyl-acetate
Decahydro-2-naphthoic acid
Tetralin
1.14.13.-
1.1.1.1
2-Naphthaldehyde
1.2.1.-
4-Formylsalicylic acid
2-Hydroxymethyl-naphthalene
1.14.12.12
cis-1,2-Dihydroxy-1,2-dihydro-7-hydroxy-methylnaphthalene
1.3.1.29
1,2-Dihydroxy-7-hydroxymethylnaphthalene
1.13.-.-
2-Hydroxy-7-hydroxymethyl-chromene-2-carboxylate
5.3.99.-
2-Hydroxy-4-hydroxymethyl-benzalpyruvate
4.2.1.-
4-Hydroxy-methylsalicylaldehyde
1.2.1.-
4-Hydroxy-methylsalicylate
1.14.13.1
4-Hydroxy-methylcatechol
00626 5/19/15
(c) Kanehisa Laboratories

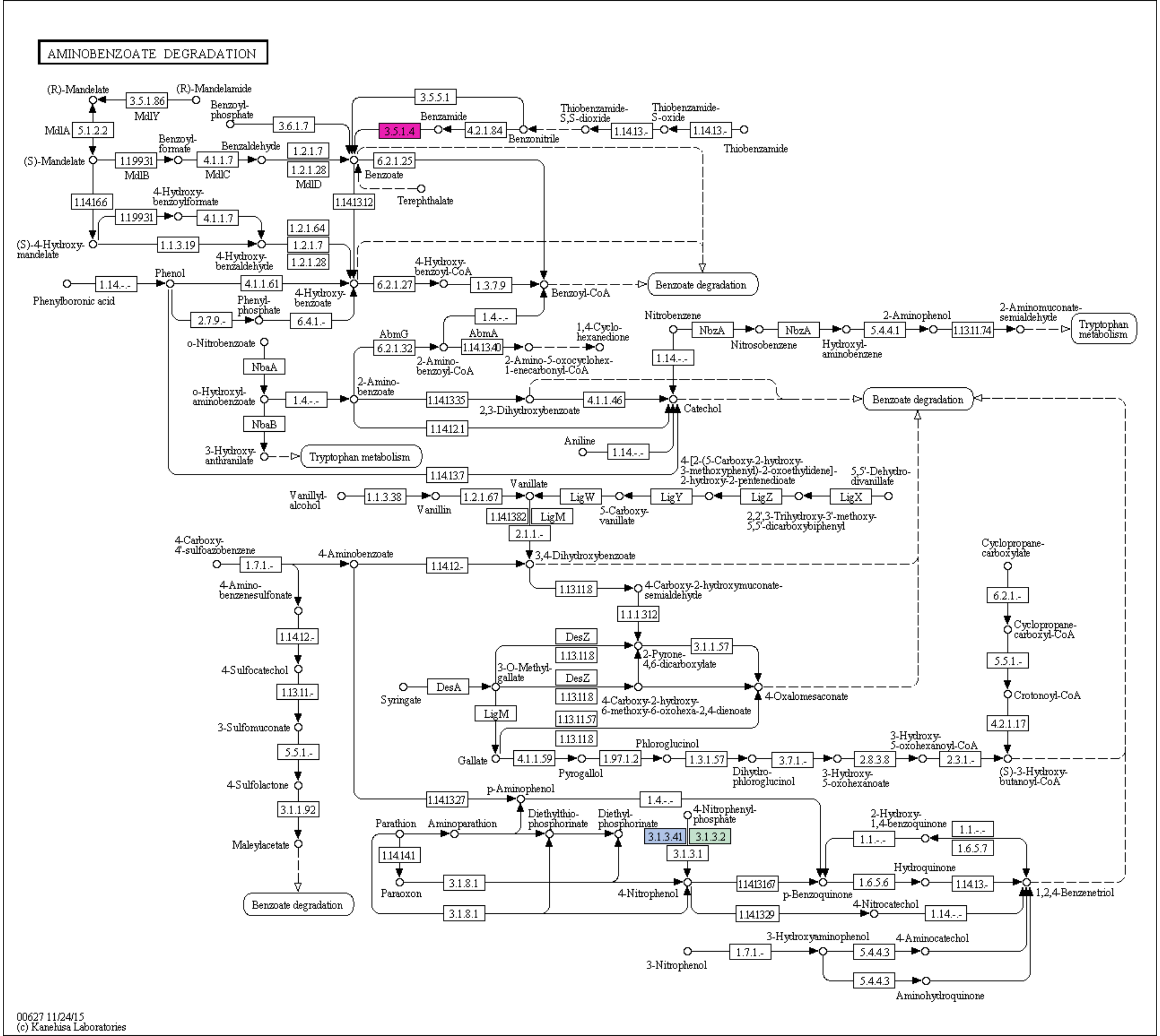
AMINOBENZOATE DEGRADATION
(R)-Mandelate
3.5.1.86
MdlY
(R)-Mandelamide
Benzoyl-phosphate
3.6.1.7
3.5.5.1
Thiobenzamide-S,S-dioxide
Thiobenzamide-S-oxide
3.5.1.4
Benzamide
4.2.1.84
Benzonitrile
1.14.13.-
1.14.13.-
Thiobenzamide
MdlA
5.1.2.2
Benzoyl-formate
Benzaldehyde
1.2.1.7
6.2.1.25
(S)-Mandelate
1.1.99.31
MdlB
4.1.1.7
MdlC
1.2.1.28
MdlD
Benzoate
Terephthalate
1.14.16.6
1.14.13.12
4-Hydroxy-benzoylformate
1.1.99.31
4.1.1.7
1.2.1.64
(S)-4-Hydroxy-mandelate
1.1.3.19
1.2.1.7
4-Hydroxy-benzaldehyde
1.2.1.28
4-Hydroxy-benzoyl-CoA
Phenol
1.14.-.-
4.1.1.61
6.2.1.27
1.3.7.9
Benzoyl-CoA
Benzoate degradation
Phenylboronic acid
Phenyl-phosphate
4-Hydroxy-benzoate
2.7.9.-
6.4.1.-
1.4.-.-
Nitrobenzene
NbzA
NbzA
2-Aminophenol
5.4.4.1
1.13.11.74
2-Aminomuconate-semialdehyde
Tryptophan metabolism
Nitrosobenzene
Hydroxyl-aminobenzene
AbmG
AbmA
1,4-Cyclo-hexanedione
o-Nitrobenzoate
6.2.1.32
1.14.13.40
2-Amino-benzoyl-CoA
2-Amino-5-oxocyclohex-1-enecarbonyl-CoA
1.14.-.-
NbaA
o-Hydroxyl-aminobenzoate
1.4.-.-
2-Amino-benzoate
1.14.13.35
2,3-Dihydroxybenzoate
4.1.1.46
Catechol
Benzoate degradation
NbaB
1.14.12.1
Aniline
1.14.-.-
3-Hydroxy-anthranilate
Tryptophan metabolism
1.14.13.7
4-[2-(5-Carboxy-2-hydroxy-3-methoxyphenyl)-2-oxoethylidene]-2-hydroxy-2-pentenedioate
5,5'-Dehydro-divanillate
Vanillyl-alcohol
1.1.3.38
Vanillin
1.2.1.67
Vanillate
LigW
5-Carboxy-vanillate
LigY
LigZ
LigX
1.14.13.82
LigM
2,2',3-Trihydroxy-3'-methoxy-5,5'-dicarboxybiphenyl
2.1.1.-
4-Carboxy-4'-sulfoazobenzene
1.7.1.-
4-Aminobenzoate
1.14.12.-
3,4-Dihydroxybenzoate
Cyclopropane-carboxylate
4-Amino-benzenesulfonate
1.13.11.8
4-Carboxy-2-hydroxymuconate-semialdehyde
6.2.1.-
1.1.1.312
Cyclopropane-carboxyl-CoA
1.14.12.-
DesZ
1.13.11.8
3.1.1.57
2-Pyrone-4,6-dicarboxylate
5.5.1.-
4-Sulfocatechol
3-O-Methyl-gallate
Syringate
DesA
DesZ
1.13.11.8
4-Oxalomesaconate
Crotonoyl-CoA
1.13.11.-
LigM
4-Carboxy-2-hydroxy-6-methoxy-6-oxohexa-2,4-dienoate
1.13.11.57
4.2.1.17
3-Sulfomuconate
1.13.11.8
Phloroglucinol
3-Hydroxy-5-oxohexanoyl-CoA
5.5.1.-
Gallate
4.1.1.59
1.97.1.2
1.3.1.57
3.7.1.-
2.8.3.8
2.3.1.-
Pyrogallol
Dihydro-phloroglucinol
3-Hydroxy-5-oxohexanoate
(S)-3-Hydroxy-butanoyl-CoA
4-Sulfolactone
1.14.13.27
p-Aminophenol
1.4.-.-
3.1.1.92
Parathion
Aminoparathion
Diethylthio-phosphorinate
Diethyl-phosphorinate
4-Nitrophenyl-phosphate
2-Hydroxy-1,4-benzoquinone
1.1.-.-
3.1.3.41
3.1.3.2
1.1.-.-
Maleylacetate
1.14.14.1
3.1.3.1
1.6.5.7
Hydroquinone
Paraoxon
3.1.8.1
1.14.13.167
1.6.5.6
1.14.13.-
4-Nitrophenol
p-Benzoquinone
1,2,4-Benzenetriol
Benzoate degradation
3.1.8.1
1.14.13.29
4-Nitrocatechol
1.14.-.-
3-Hydroxyaminophenol
4-Aminocatechol
3-Nitrophenol
1.7.1.-
5.4.4.3
5.4.4.3
Aminohydroquinone
00627 11/24/15
(c) Kanehisa Laboratories

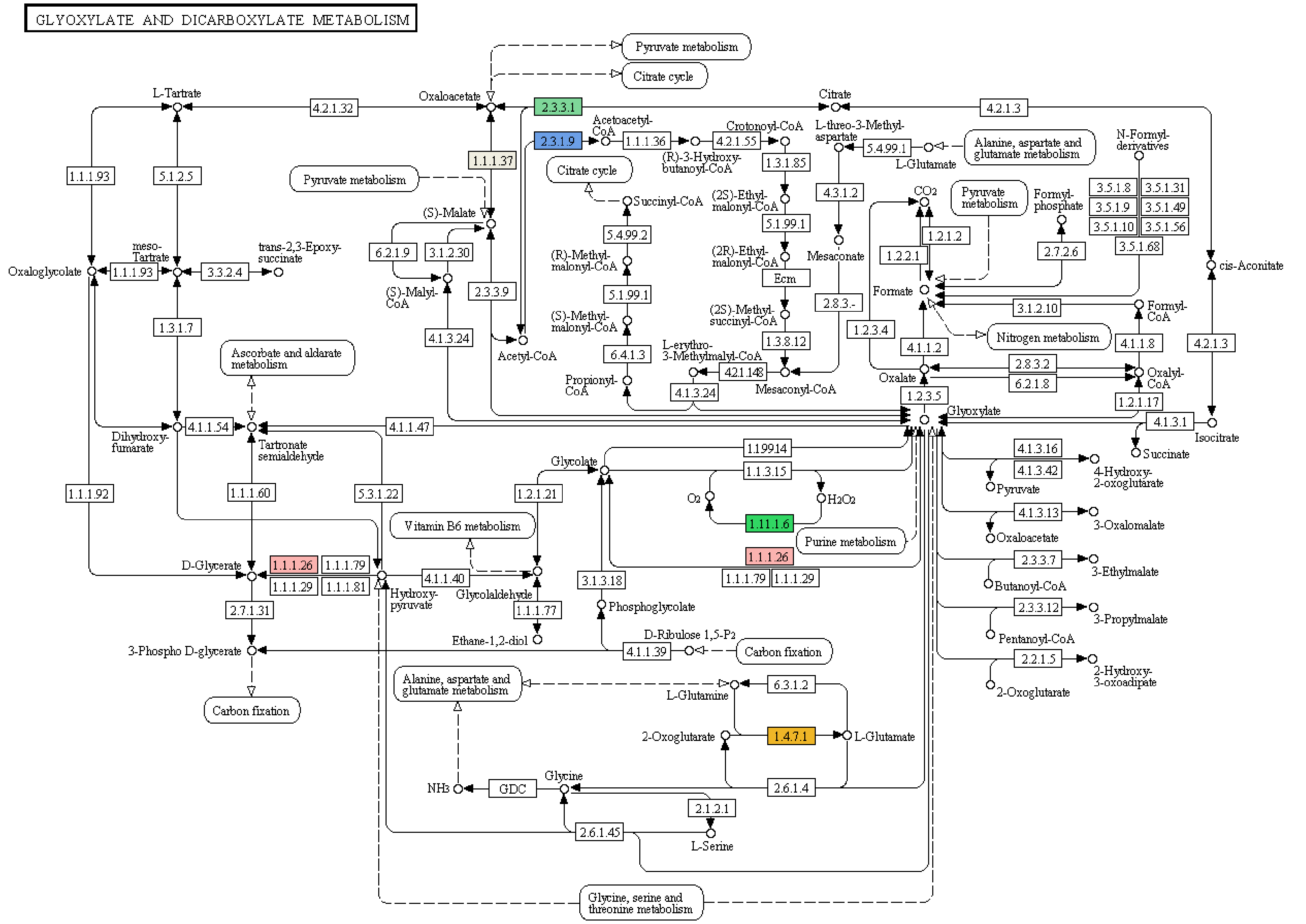

00630 9/10/15
(c) Kanehisa Laboratories

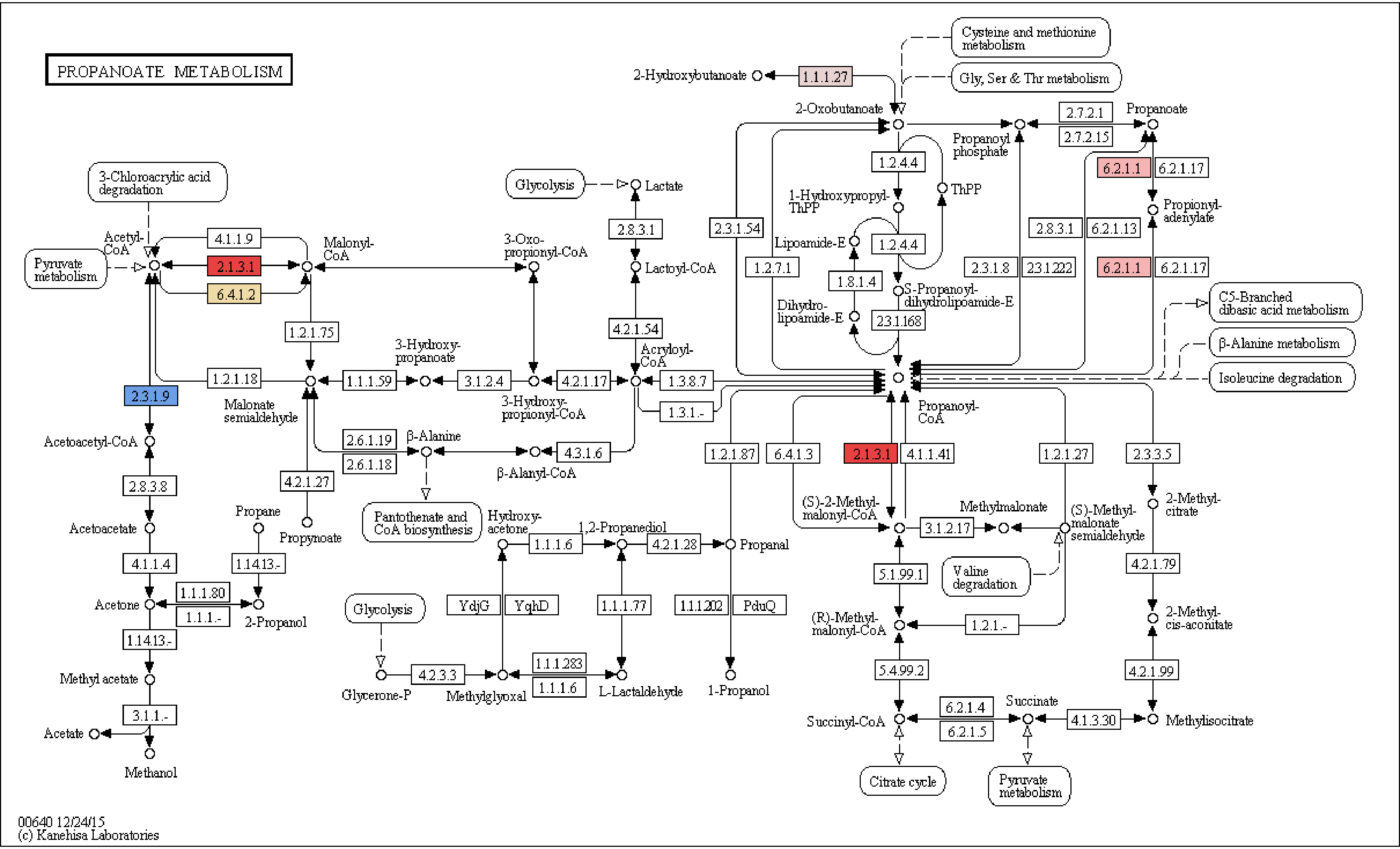

PROPANOATE METABOLISM
Cysteine and methionine metabolism
Gly, Ser & Thr metabolism
2-Hydroxybutanoate
1.1.1.27
2-Oxobutanoate
2.7.2.1
2.7.2.15
Propanoate
Propanoyl phosphate
1.2.4.4
ThPP
1-Hydroxypropyl-ThPP
6.2.1.1
6.2.1.17
Propionyl-adenylate
3-Chloroacrylic acid degradation
Glycolysis
Lactate
2.8.3.1
2.3.1.54
Lipoamide-E
1.2.4.4
2.8.3.1
6.2.1.13
Acetyl-CoA
4.1.1.9
Malonyl-CoA
3-Oxo-propionyl-CoA
Pyruvate metabolism
2.1.3.1
Lactoyl-CoA
1.2.7.1
1.8.1.4
S-Propanoyl-dihydrolipoamide-E
2.3.1.8
2.3.1.222
6.2.1.1
6.2.1.17
6.4.1.2
C5-Branched dibasic acid metabolism
Dihydro-lipoamide-E
2.3.1.168
1.2.1.75
4.2.1.54
β-Alanine metabolism
3-Hydroxy-propanoate
Acryloyl-CoA
1.2.1.18
1.1.1.59
3.1.2.4
4.2.1.17
1.3.8.7
Isoleucine degradation
2.3.1.9
Malonate semialdehyde
3-Hydroxy-propionyl-CoA
1.3.1.-
Propanoyl-CoA
2.6.1.19
β-Alanine
Acetoacetyl-CoA
2.6.1.18
4.3.1.6
1.2.1.87
6.4.1.3
2.1.3.1
4.1.1.41
1.2.1.27
2.3.3.5
β-Alanyl-CoA
2.8.3.8
4.2.1.27
Propane
Pantothenate and CoA biosynthesis
Hydroxy-acetone
(S)-2-Methyl-malonyl-CoA
Methylmalonate
(S)-Methyl-malonate semialdehyde
2-Methyl-citrate
Acetoacetate
Propynoate
1,2-Propanediol
3.1.2.17
1.1.1.6
4.2.1.28
Propanal
4.1.1.4
1.1.4.13.-
Valine degradation
5.1.99.1
4.2.1.79
1.1.1.80
Acetone
2-Propanol
1.1.1.-
Glycolysis
YdjG
YqhD
1.1.1.77
1.1.1.202
PduQ
(R)-Methyl-malonyl-CoA
1.2.1.-
2-Methyl-cis-aconitate
1.14.13.-
Methyl acetate
1.1.1.283
4.2.3.3
1.1.1.6
5.4.99.2
4.2.1.99
Glycerone-P
Methylglyoxal
L-Lactaldehyde
1-Propanol
6.2.1.4
Succinate
Succinyl-CoA
4.1.3.30
Methylisocitrate
3.1.1.-
6.2.1.5
Acetate
Methanol
Citrate cycle
Pyruvate metabolism
00640 12/24/15
(c) Kanehisa Laboratories

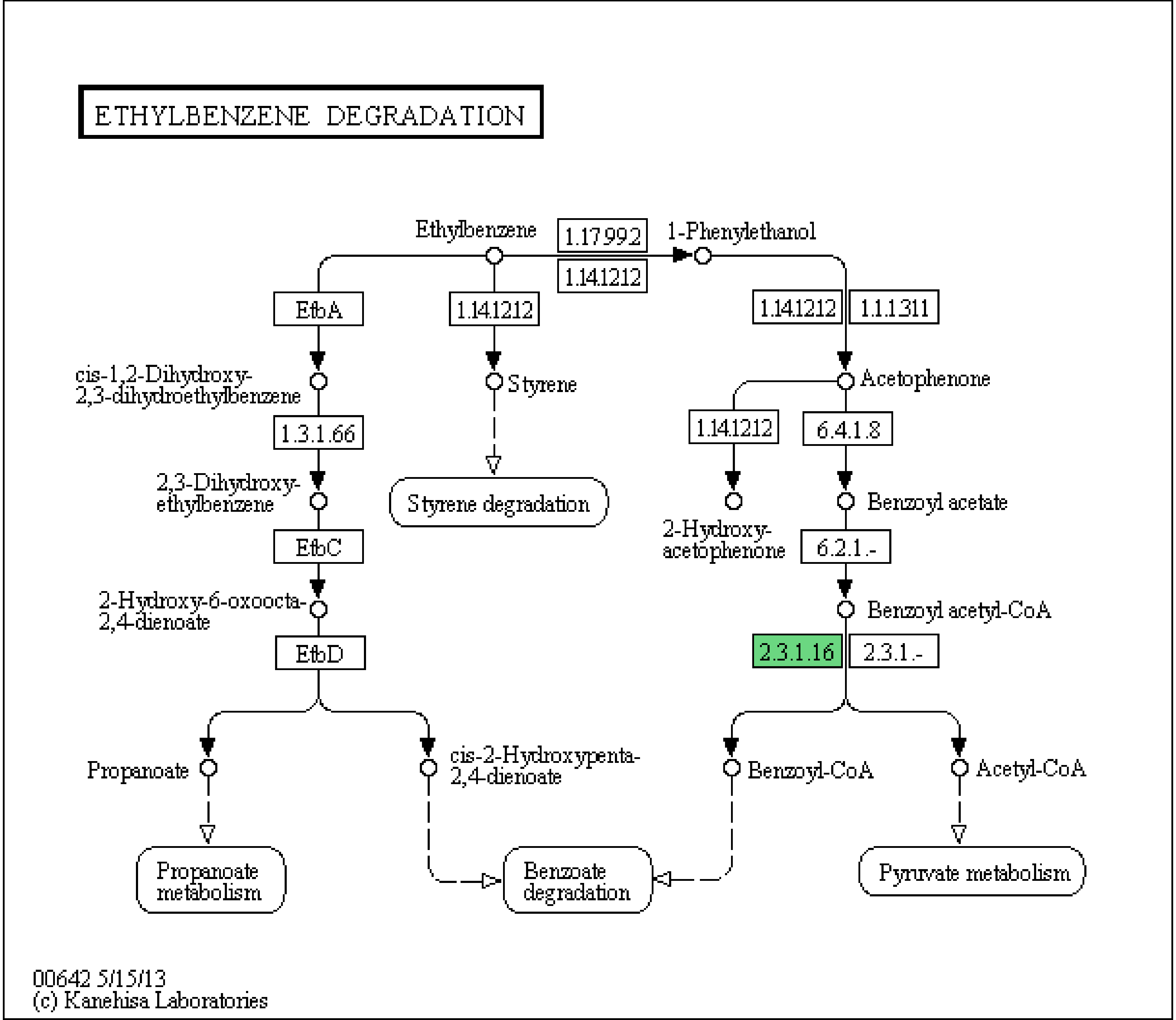
ETHYLBENZENE DEGRADATION
Ethylbenzene
1.17.99.2
1.14.12.12
1-Phenylethanol
EtbA
1.14.12.12
1.14.12.12
1.1.1.311
cis-1,2-Dihydroxy-2,3-dihydroethylbenzene
Styrene
Acetophenone
1.3.1.66
1.14.12.12
6.4.1.8
2,3-Dihydroxy-ethylbenzene
Styrene degradation
Benzoyl acetate
2-Hydroxy-acetophenone
EtbC
6.2.1.-
2-Hydroxy-6-oxoocta-2,4-dienoate
Benzoyl acetyl-CoA
EtbD
2.3.1.16
2.3.1.-
Propanoate
cis-2-Hydroxypenta-2,4-dienoate
Benzoyl-CoA
Acetyl-CoA
Propanoate metabolism
Benzoate degradation
Pyruvate metabolism
00642 5/15/13
(c) Kanehisa Laboratories

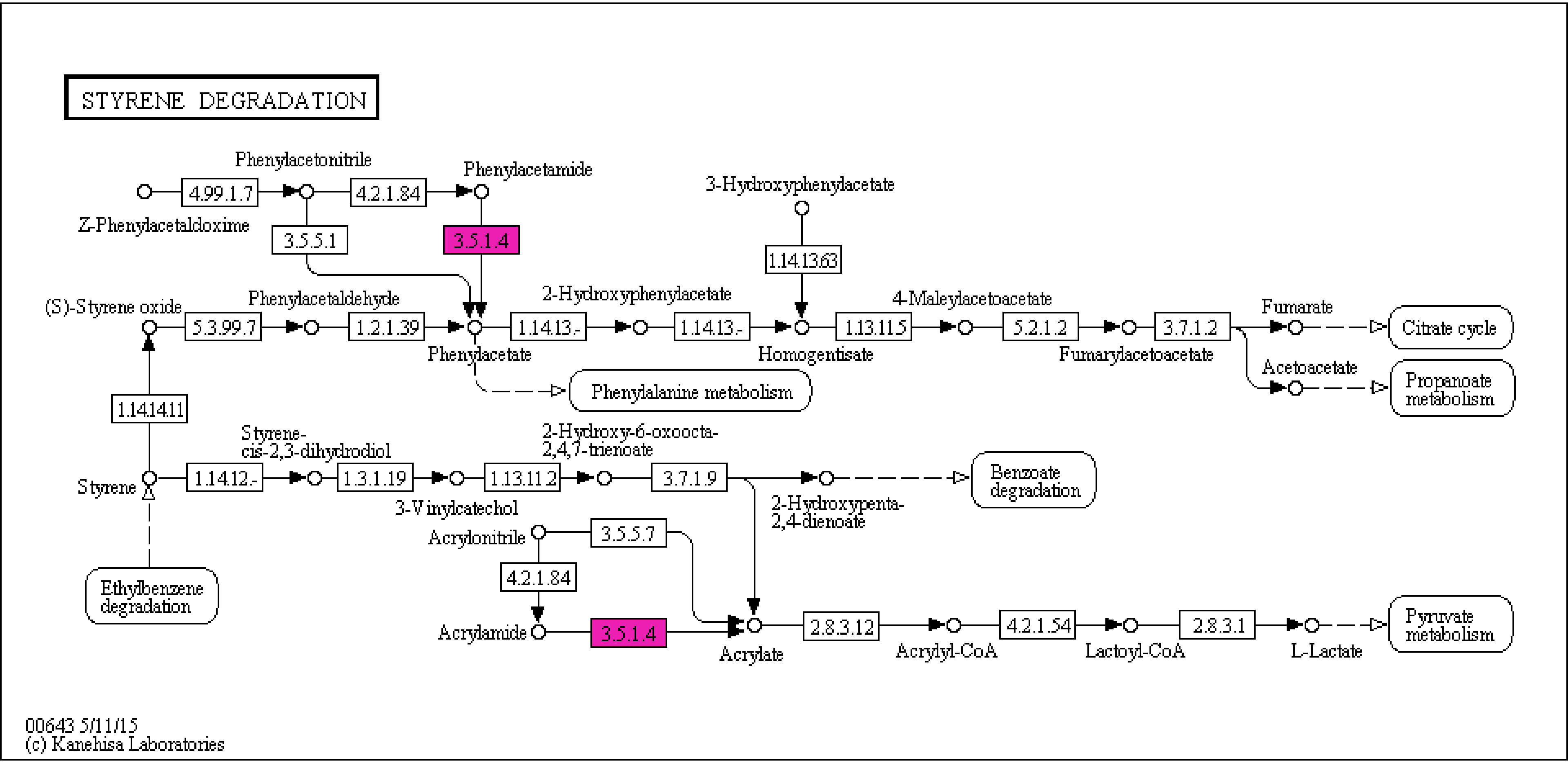
STYRENE DEGRADATION
Phenylacetonitrile
Phenylacetamide
4.99.1.7
4.2.1.84
Z-Phenylacetaldoxime
3.5.5.1
3.5.1.4
3-Hydroxyphenylacetate
1.14.13.63
(S)-Styrene oxide
Phenylacetaldehyde
5.3.99.7
1.2.1.39
2-Hydroxyphenylacetate
1.14.13.-
1.14.13.-
Phenylacetate
Homogentisate
1.13.11.5
4-Maleylacetoacetate
5.2.1.2
Fumarylacetoacetate
3.7.1.2
Fumarate
Citrate cycle
Acetoacetate
Propanoate metabolism
Phenylalanine metabolism
1.14.14.11
Styrene-cis-2,3-dihydrodiol
2-Hydroxy-6-oxoocta-2,4,7-trienoate
Styrene
1.14.12.-
1.3.1.19
1.13.11.2
3.7.1.9
3-Vinylcatechol
2-Hydroxypenta-2,4-dienoate
Benzoate degradation
Acrylonitrile
3.5.5.7
4.2.1.84
Ethylbenzene degradation
Acrylamide
3.5.1.4
Acrylate
2.8.3.12
Acrylyl-CoA
4.2.1.54
Lactoyl-CoA
2.8.3.1
L-Lactate
Pyruvate metabolism
00643 5/11/15
(c) Kanehisa Laboratories

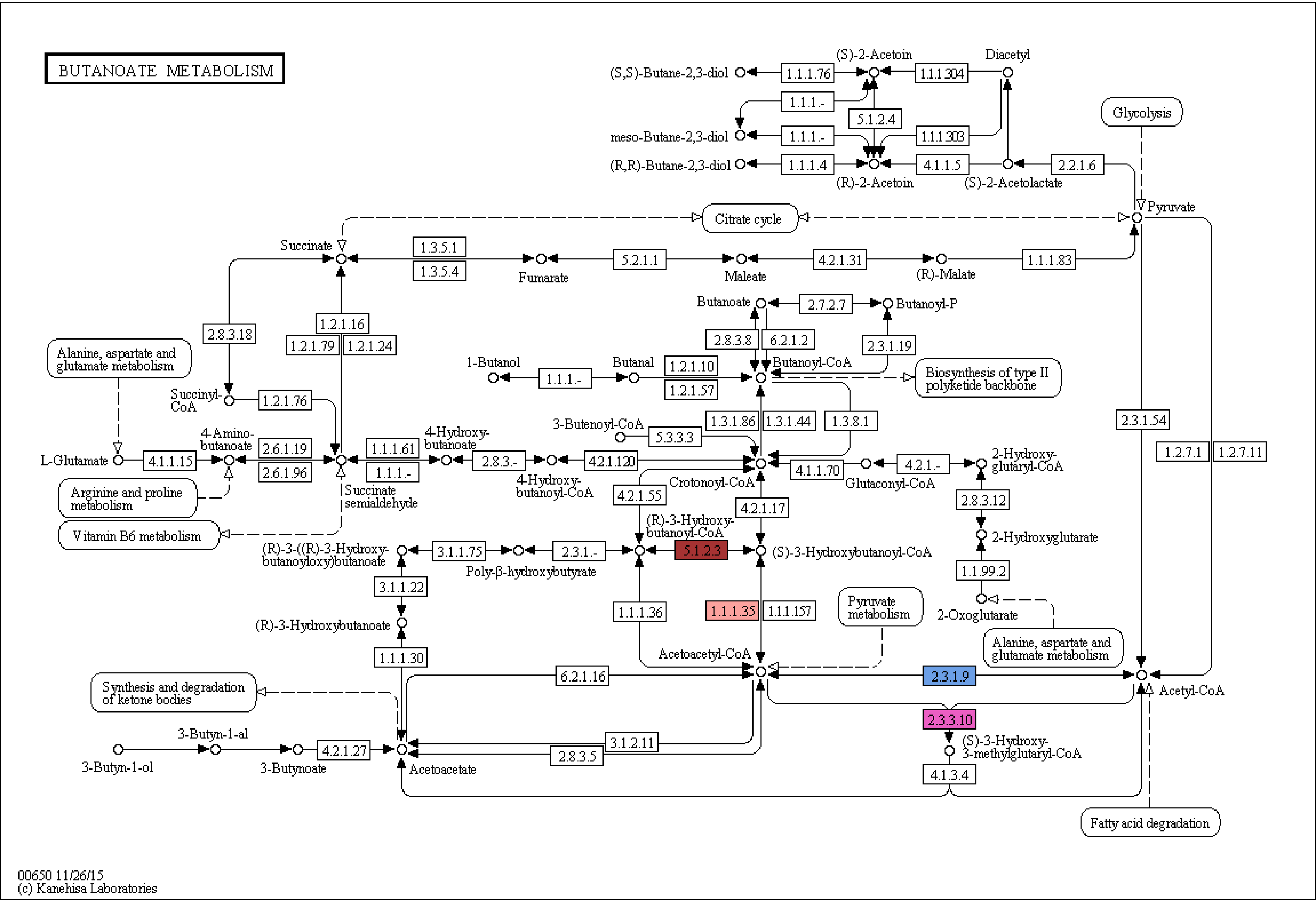

BUTANOATE METABOLISM
(S)-2-Acetoin
Diacetyl
(S,S)-Butane-2,3-diol
1.1.1.76
1.1.1.304
1.1.1.-
5.1.2.4
Glycolysis
meso-Butane-2,3-diol
1.1.1.-
1.1.1.303
(R,R)-Butane-2,3-diol
1.1.1.4
4.1.1.5
2.2.1.6
(R)-2-Acetoin
(S)-2-Acetolactate
Pyruvate
Citrate cycle
Succinate
1.3.5.1
1.3.5.4
Fumarate
5.2.1.1
Maleate
4.2.1.31
(R)-Malate
1.1.1.83
Butanoate
2.7.2.7
Butanoyl-P
2.8.3.18
1.2.1.16
2.8.3.8
6.2.1.2
2.3.1.19
Alanine, aspartate and glutamate metabolism
1.2.1.79
1.2.1.24
1-Butanol
Butanal
1.2.1.10
Butanoyl-CoA
Biosynthesis of type II polyketide backbone
1.1.1.-
1.2.1.57
Succinyl-CoA
1.2.1.76
3-Butenoyl-CoA
1.3.1.86
1.3.1.44
1.3.8.1
2.3.1.54
4-Amino-butanoate
4-Hydroxy-butanoate
5.3.3.3
1.2.7.1
1.2.7.11
L-Glutamate
4.1.1.15
2.6.1.19
1.1.1.61
2.8.3.-
4.2.1.120
4.2.1.-
2-Hydroxy-glutaryl-CoA
2.6.1.96
1.1.1.-
4.1.1.70
Glutaconyl-CoA
Arginine and proline metabolism
Succinate semialdehyde
4-Hydroxy-butanoyl-CoA
Crotonoyl-CoA
4.2.1.55
4.2.1.17
2.8.3.12
Vitamin B6 metabolism
(R)-3-Hydroxy-butanoyl-CoA
2-Hydroxyglutarate
(R)-3-((R)-3-Hydroxy-butanoyloxy)butanoate
3.1.1.75
2.3.1.-
5.1.2.3
(S)-3-Hydroxybutanoyl-CoA
Poly-β-hydroxybutyrate
1.1.99.2
3.1.1.22
Pyruvate metabolism
1.1.1.36
1.1.1.35
1.1.1.157
2-Oxoglutarate
(R)-3-Hydroxybutanoate
Alanine, aspartate and glutamate metabolism
1.1.1.30
Acetoacetyl-CoA
6.2.1.16
2.3.1.9
Acetyl-CoA
Synthesis and degradation of ketone bodies
2.3.3.10
(S)-3-Hydroxy-3-methylglutaryl-CoA
3-Butyn-1-al
3.1.2.11
3-Butyn-1-ol
3-Butynoate
4.2.1.27
2.8.3.5
Acetoacetate
4.1.3.4
Fatty acid degradation
00650 11/26/15
(c) Kanehisa Laboratories

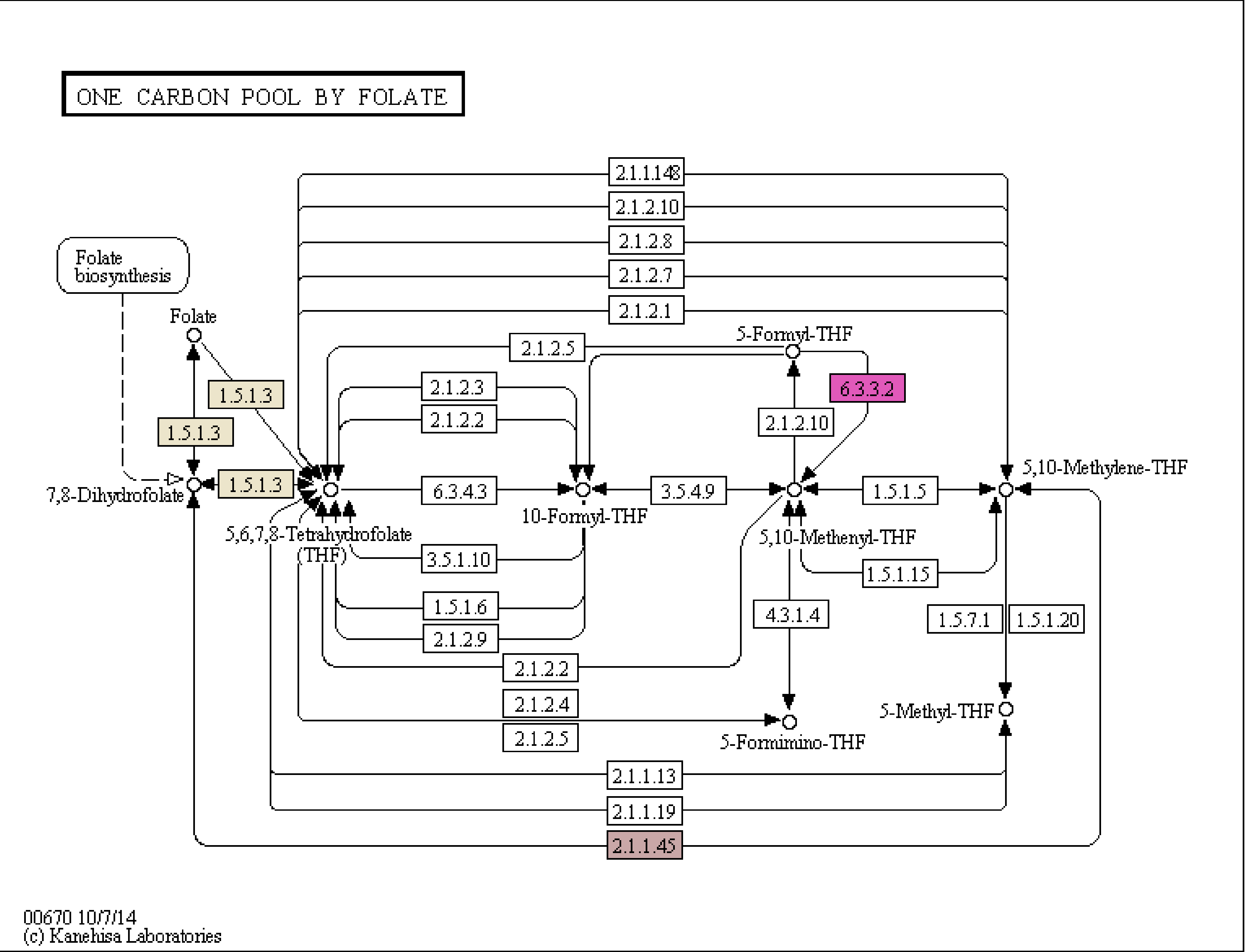
ONE CARBON POOL BY FOLATE
Folate biosynthesis
Folate
7,8-Dihydrofolate
5,6,7,8-Tetrahydrofolate (THF)
10-Formyl-THF
5-Formyl-THF
5,10-Methenyl-THF
5,10-Methylene-THF
5-Formimino-THF
5-Methyl-THF
2.1.1.148
2.1.2.10
2.1.2.8
2.1.2.7
2.1.2.1
2.1.2.5
2.1.2.3
2.1.2.2
1.5.1.3
1.5.1.3
1.5.1.3
6.3.4.3
3.5.4.9
1.5.1.5
6.3.3.2
2.1.2.10
3.5.1.10
1.5.1.6
2.1.2.9
1.5.1.15
4.3.1.4
1.5.7.1
1.5.1.20
2.1.2.2
2.1.2.4
2.1.2.5
2.1.1.13
2.1.1.19
2.1.1.45
00670 10/7/14
(c) Kanehisa Laboratories

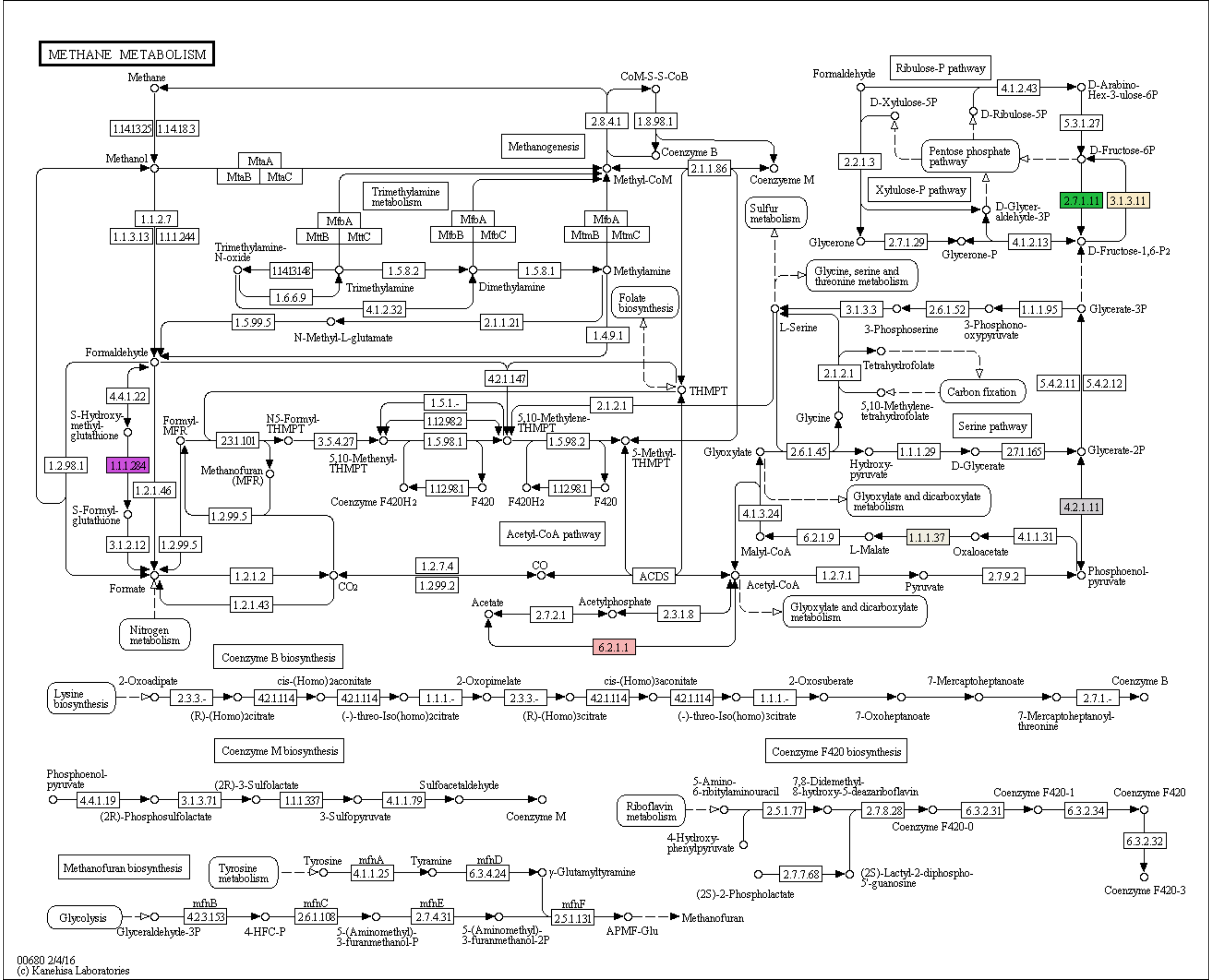
METHANE METABOLISM
Methane
CoM-S-S-CoB
1.14.13.25
1.14.18.3
2.8.4.1
1.8.98.1
Methanogenesis
Coenzyme B
Methanol
MtaA
MtaB
MtaC
2.1.1.86
Methyl-CoM
Coenzyme M
Trimethylamine metabolism
MtbA
MttB
MttC
MtbB
MtbC
MtmB
MtmC
1.1.2.7
1.1.3.13
1.1.1.244
Trimethylamine-N-oxide
1.14.13.148
1.5.8.2
1.5.8.1
Methylamine
Trimethylamine
Dimethylamine
1.6.6.9
4.1.2.32
Folate biosynthesis
1.5.99.5
N-Methyl-L-glutamate
2.1.1.21
1.4.9.1
Formaldehyde
4.2.1.147
THMPT
4.4.1.22
S-Hydroxy-methyl-glutathione
1.1.1.284
1.2.98.1
1.2.1.46
S-Formyl-glutathione
3.1.2.12
1.2.99.5
Formyl-MFR
2.3.1.101
N5-Formyl-THMPT
3.5.4.27
5,10-Methenyl-THMPT
1.5.1.-
1.12.98.2
1.5.98.1
5,10-Methylene-THMPT
1.5.98.2
5-Methyl-THMPT
2.1.2.1
Methanofuran (MFR)
1.2.99.5
1.12.98.1
Coenzyme F420H2
F420
F420H2
F420
Acetyl-CoA pathway
Formate
1.2.1.2
CO2
1.2.7.4
1.2.99.2
CO
ACDS
1.2.1.43
Nitrogen metabolism
Acetate
2.7.2.1
Acetylphosphate
2.3.1.8
6.2.1.1
Acetyl-CoA
Glyoxylate and dicarboxylate metabolism
Formaldehyde
Ribulose-P pathway
4.1.2.43
D-Arabino-Hex-3-ulose-6P
D-Xylulose-5P
D-Ribulose-5P
5.3.1.27
2.2.1.3
Pentose phosphate pathway
D-Fructose-6P
Xylulose-P pathway
2.7.1.11
3.1.3.11
Sulfur metabolism
D-Glyceraldehyde-3P
Glycerone
2.7.1.29
Glycerone-P
4.1.2.13
D-Fructose-1,6-P2
Glycine, serine and threonine metabolism
L-Serine
3.1.3.3
3-Phosphoserine
2.6.1.52
3-Phosphonooxypyruvate
1.1.1.95
Glycerate-3P
2.1.2.1
Tetrahydrofolate
Carbon fixation
5,10-Methylene-tetrahydrofolate
5.4.2.11
5.4.2.12
Glycine
Serine pathway
Glyoxylate
2.6.1.45
Hydroxy-pyruvate
1.1.1.29
D-Glycerate
2.7.1.165
Glycerate-2P
Glyoxylate and dicarboxylate metabolism
4.2.1.11
4.1.3.24
Malyl-CoA
6.2.1.9
L-Malate
1.1.1.37
Oxaloacetate
4.1.1.31
Phosphoenol-pyruvate
1.2.7.1
Pyruvate
2.7.9.2
Coenzyme B biosynthesis
Lysine biosynthesis
2-Oxoadipate
2.3.3.-
(R)-(Homo)2citrate
cis-(Homo)2aconitate
4.2.1.114
(-)-threo-Iso(homo)2citrate
1.1.1.-
2-Oxopimelate
(R)-(Homo)3citrate
cis-(Homo)3aconitate
(-)-threo-Iso(homo)3citrate
2-Oxosuberate
7-Oxoheptanoate
7-Mercaptoheptanoate
7-Mercaptoheptanoyl-threonine
2.7.1.-
Coenzyme B
Coenzyme M biosynthesis
Coenzyme F420 biosynthesis
Phosphoenol-pyruvate
4.4.1.19
(2R)-Phosphosulfolactate
(2R)-3-Sulfolactate
3.1.3.71
1.1.1.337
3-Sulfopyruvate
Sulfoacetaldehyde
4.1.1.79
Coenzyme M
Riboflavin metabolism
5-Amino-6-ribitylaminouracil
4-Hydroxy-phenylpyruvate
2.5.1.77
7,8-Didemethyl-8-hydroxy-5-deazariboflavin
2.7.8.28
Coenzyme F420-0
6.3.2.31
Coenzyme F420-1
6.3.2.34
Coenzyme F420
6.3.2.32
Coenzyme F420-3
2.7.7.68
(2S)-2-Phospholactate
(2S)-Lactyl-2-diphospho-5'-guanosine
Methanofuran biosynthesis
Tyrosine metabolism
Tyrosine
mfnA
4.1.1.25
Tyramine
mfnD
6.3.4.24
γ-Glutamyltyramine
Glycolysis
Glyceraldehyde-3P
mfnB
4.2.3.153
4-HFC-P
mfnC
2.6.1.108
5-(Aminomethyl)-3-furanmethanol-P
mfnE
2.7.4.31
5-(Aminomethyl)-3-furanmethanol-2P
mfnF
2.5.1.131
APMF-Glu
Methanofuran
00680 2/4/16
(c) Kanehisa Laboratories

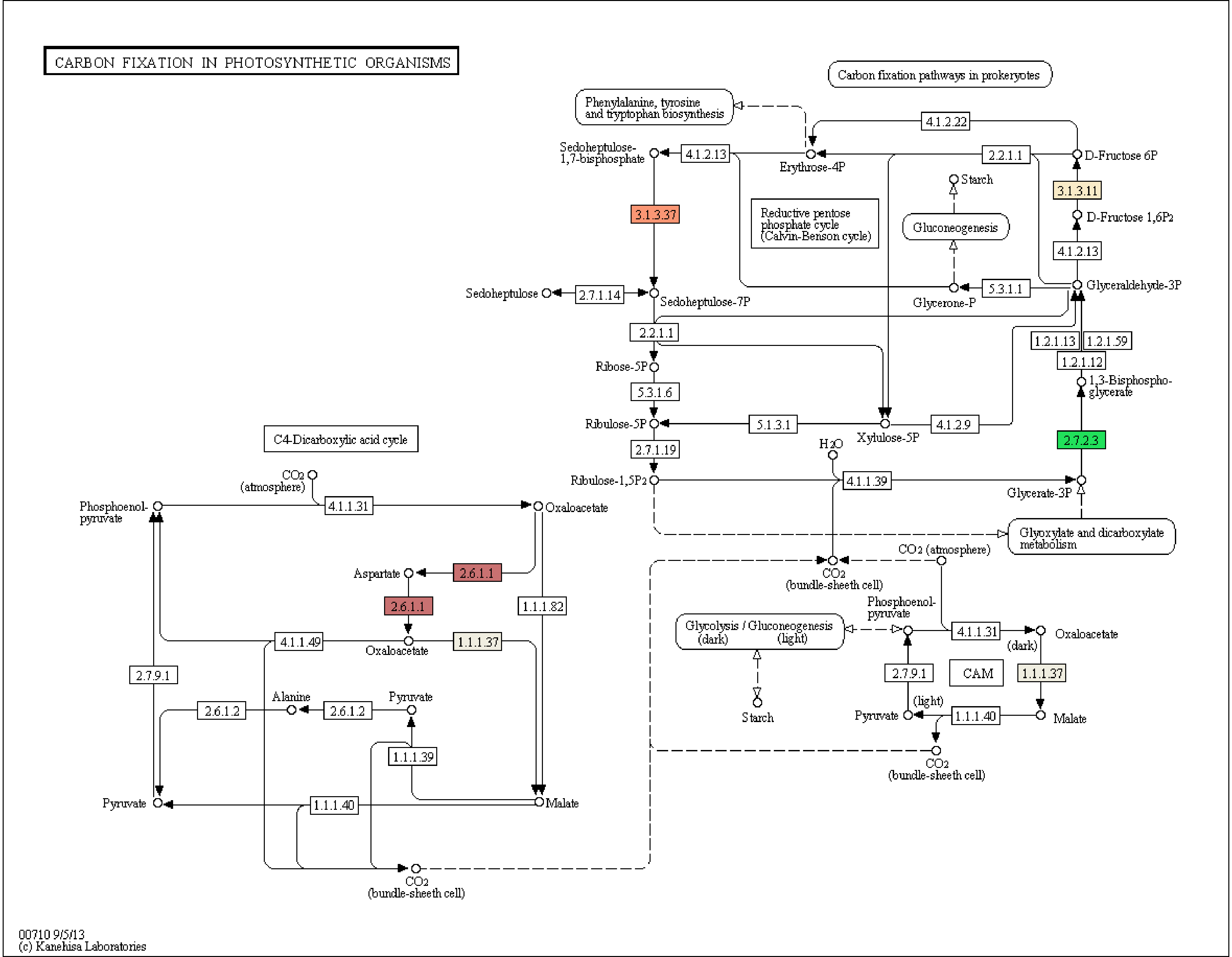

CARBON FIXATION IN PHOTOSYNTHETIC ORGANISMS
Carbon fixation pathways in prokeryotes
Phenylalanine, tyrosine and tryptophan biosynthesis
Sedoheptulose-1,7-bisphosphate
4.1.2.13
Erythrose-4P
4.1.2.22
2.2.1.1
D-Fructose 6P
3.1.3.37
Reductive pentose phosphate cycle (Calvin-Benson cycle)
Starch
Gluconeogenesis
3.1.3.11
D-Fructose 1,6P2
4.1.2.13
Sedoheptulose
2.7.1.14
Sedoheptulose-7P
Glycerone-P
5.3.1.1
Glyceraldehyde-3P
2.2.1.1
1.2.1.13
1.2.1.59
1.2.1.12
Ribose-5P
1,3-Bisphospho-glycerate
5.3.1.6
Ribulose-5P
5.1.3.1
Xylulose-5P
4.1.2.9
2.7.2.3
C4-Dicarboxylic acid cycle
H2O
2.7.1.19
Ribulose-1,5P2
4.1.1.39
Glycerate-3P
CO2 (atmosphere)
Phosphoenol-pyruvate
4.1.1.31
Oxaloacetate
Glyoxylate and dicarboxylate metabolism
CO2 (atmosphere)
CO2 (bundle-sheeth cell)
Aspartate
2.6.1.1
2.6.1.1
1.1.1.82
Phosphoenol-pyruvate
Glycolysis / Gluconeogenesis (dark) (light)
4.1.1.31
Oxaloacetate
(dark)
4.1.1.49
Oxaloacetate
1.1.1.37
2.7.9.1
2.7.9.1
CAM
1.1.1.37
(light)
Starch
Pyruvate
1.1.1.40
Malate
Alanine
2.6.1.2
2.6.1.2
Pyruvate
CO2 (bundle-sheeth cell)
1.1.1.39
Pyruvate
1.1.1.40
Malate
CO2 (bundle-sheeth cell)
00710 9/5/13
(c) Kanehisa Laboratories

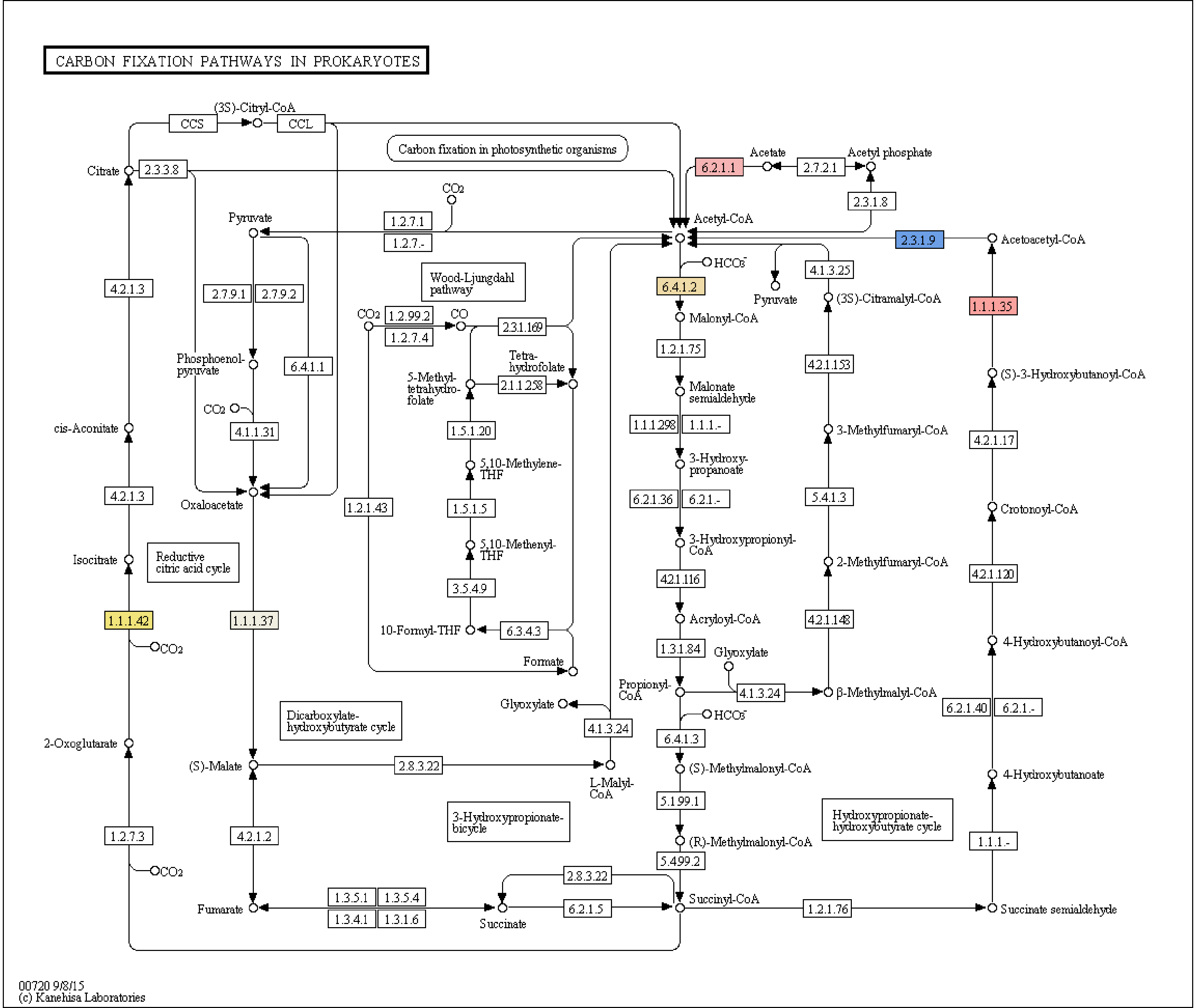
CARBON FIXATION PATHWAYS IN PROKARYOTES
(3S)-Citryl-CoA
CCS
CCL
Carbon fixation in photosynthetic organisms
Citrate
2.3.3.8
CO2
Pyruvate
1.2.7.1
1.2.7.-
Acetate
Acetyl phosphate
6.2.1.1
2.7.2.1
2.3.1.8
Acetyl-CoA
2.3.1.9
Acetoacetyl-CoA
HCO3-
4.1.3.25
Pyruvate
(3S)-Citramalyl-CoA
1.1.1.35
4.2.1.3
2.7.9.1
2.7.9.2
Wood-Ljungdahl pathway
6.4.1.2
Malonyl-CoA
CO2
1.2.99.2
CO
2.3.1.169
1.2.7.4
Tetra-hydrofolate
1.2.1.75
4.2.1.153
(S)-3-Hydroxybutanoyl-CoA
Phosphoenol-pyruvate
6.4.1.1
5-Methyl-tetrahydro-folate
2.1.1.258
Malonate semialdehyde
CO2
4.1.1.31
1.5.1.20
1.1.1.298
1.1.1.-
3-Methylfumaryl-CoA
cis-Aconitate
4.2.1.17
5,10-Methylene-THF
3-Hydroxy-propanoate
4.2.1.3
Oxaloacetate
1.2.1.43
1.5.1.5
6.2.1.36
6.2.1.-
5.4.1.3
Crotonoyl-CoA
5,10-Methenyl-THF
3-Hydroxypropionyl-CoA
Isocitrate
Reductive citric acid cycle
2-Methylfumaryl-CoA
4.2.1.116
4.2.1.120
3.5.4.9
1.1.1.42
1.1.1.37
10-Formyl-THF
6.3.4.3
Acryloyl-CoA
4.2.1.148
CO2
1.3.1.84
Glyoxylate
4-Hydroxybutanoyl-CoA
Formate
Propionyl-CoA
4.1.3.24
β-Methylmalyl-CoA
Glyoxylate
HCO3-
6.2.1.40
6.2.1.-
Dicarboxylate-hydroxybutyrate cycle
4.1.3.24
6.4.1.3
2-Oxoglutarate
(S)-Malate
2.8.3.22
L-Malyl-CoA
(S)-Methylmalonyl-CoA
4-Hydroxybutanoate
5.1.99.1
3-Hydroxypropionate-bicycle
Hydroxypropionate-hydroxybutyrate cycle
1.2.7.3
4.2.1.2
(R)-Methylmalonyl-CoA
1.1.1.-
CO2
5.4.99.2
2.8.3.22
1.3.5.1
1.3.5.4
Succinyl-CoA
Fumarate
1.3.4.1
1.3.1.6
6.2.1.5
1.2.1.76
Succinate semialdehyde
Succinate
00720 9/8/15
(c) Kanehisa Laboratories

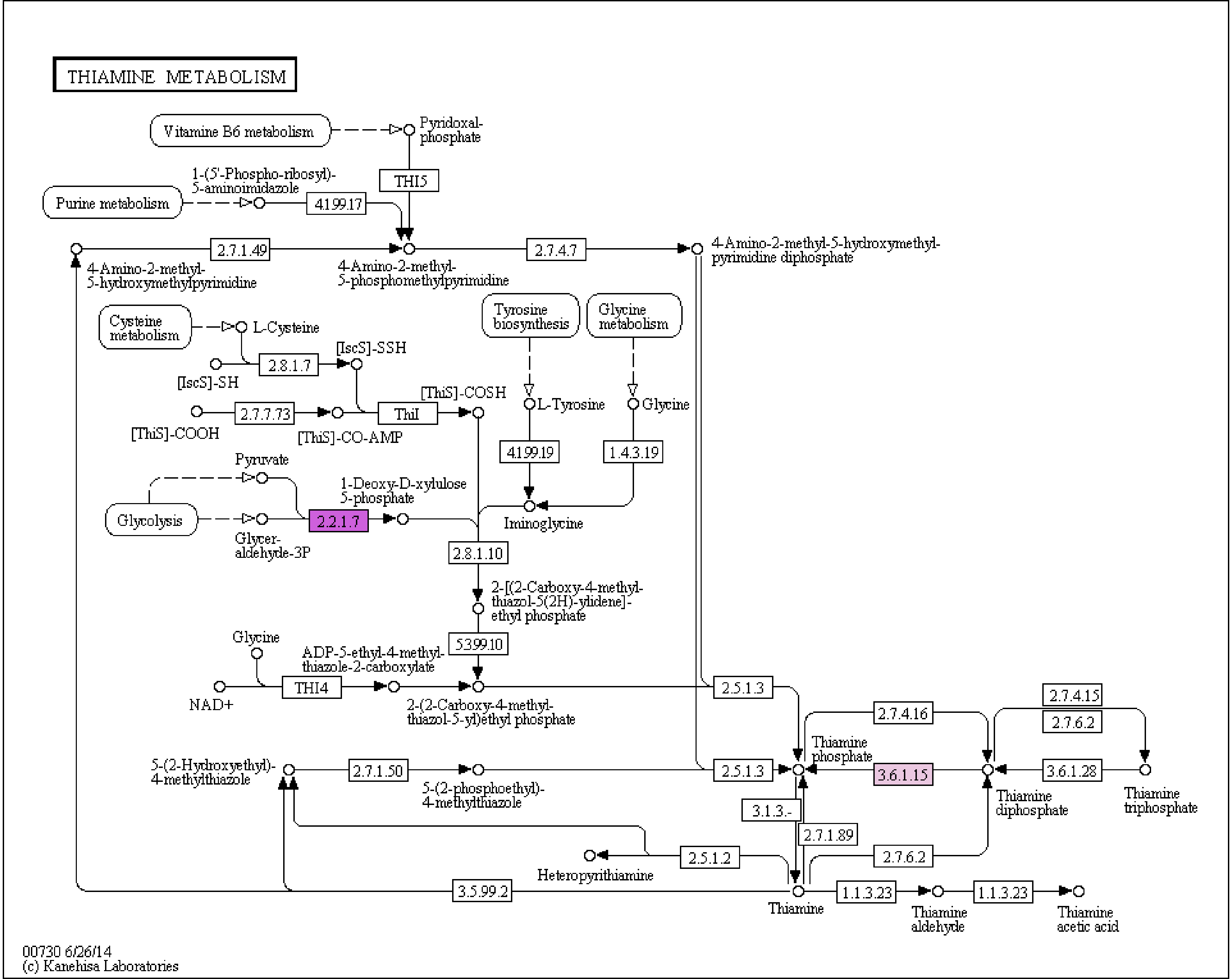
THIAMINE METABOLISM
Vitamine B6 metabolism
Pyridoxal-phosphate
THI5
Purine metabolism
1-(5'-Phospho-ribosyl)-5-aminoimidazole
4.1.99.17
2.7.1.49
4-Amino-2-methyl-5-hydroxymethylpyrimidine
4-Amino-2-methyl-5-phosphomethylpyrimidine
2.7.4.7
4-Amino-2-methyl-5-hydroxymethyl-pyrimidine diphosphate
Cysteine metabolism
L-Cysteine
[IscS]-SSH
2.8.1.7
[IscS]-SH
Tyrosine biosynthesis
Glycine metabolism
[ThiS]-COSH
2.7.7.73
ThiI
[ThiS]-COOH
[ThiS]-CO-AMP
L-Tyrosine
Glycine
4.1.99.19
1.4.3.19
Pyruvate
1-Deoxy-D-xylulose 5-phosphate
Glycolysis
2.2.1.7
Iminoglycine
Glycer-aldehyde-3P
2.8.1.10
2-[(2-Carboxy-4-methyl-thiazol-5(2H)-ylidene]-ethyl phosphate
Glycine
5.3.99.10
ADP-5-ethyl-4-methyl-thiazole-2-carboxylate
NAD+
THI4
2-(2-Carboxy-4-methyl-thiazol-5-yl)ethyl phosphate
2.5.1.3
2.7.4.15
2.7.4.16
2.7.6.2
Thiamine phosphate
5-(2-Hydroxyethyl)-4-methylthiazole
2.7.1.50
5-(2-phosphoethyl)-4-methylthiazole
2.5.1.3
3.6.1.15
3.6.1.28
Thiamine diphosphate
Thiamine triphosphate
3.1.3.-
2.7.1.89
Heteropyrithiamine
2.5.1.2
2.7.6.2
3.5.99.2
Thiamine
1.1.3.23
Thiamine aldehyde
1.1.3.23
Thiamine acetic acid
00730 6/26/14
(c) Kanehisa Laboratories

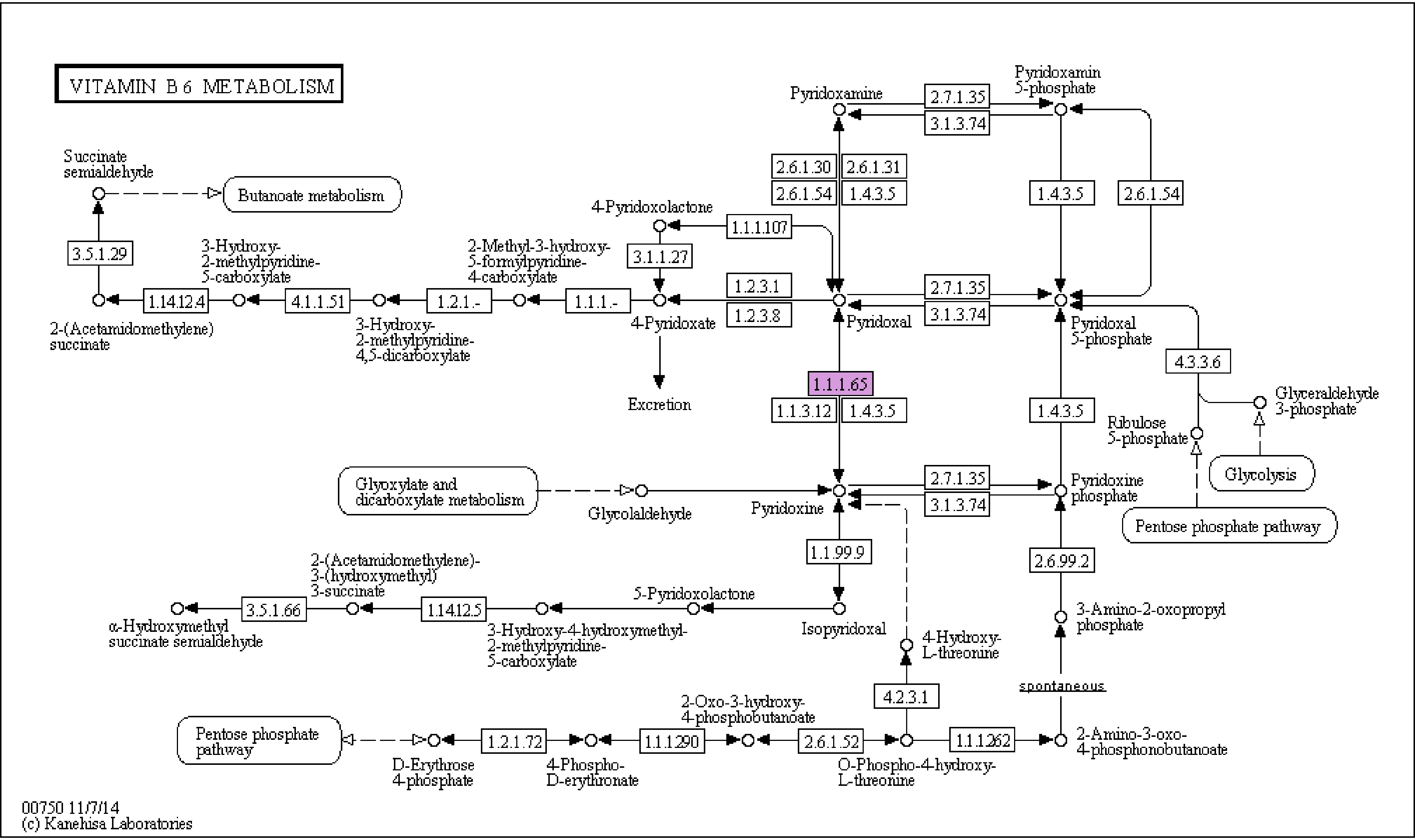

VITAMIN B6 METABOLISM
Succinate semialdehyde
Butanoate metabolism
3.5.1.29
2-(Acetamidomethylene) succinate
1.14.12.4
3-Hydroxy-2-methylpyridine-5-carboxylate
4.1.1.51
3-Hydroxy-2-methylpyridine-4,5-dicarboxylate
1.2.1.-
2-Methyl-3-hydroxy-5-formylpyridine-4-carboxylate
1.1.1.-
4-Pyridoxolactone
3.1.1.27
1.1.1.107
4-Pyridoxate
Excretion
1.2.3.1
1.2.3.8
Pyridoxamine
2.7.1.35
3.1.3.74
Pyridoxamin 5-phosphate
2.6.1.30
2.6.1.31
2.6.1.54
1.4.3.5
1.4.3.5
2.6.1.54
Pyridoxal
2.7.1.35
3.1.3.74
Pyridoxal 5-phosphate
1.1.1.65
1.1.3.12
1.4.3.5
1.4.3.5
4.3.3.6
Glyceraldehyde 3-phosphate
Ribulose 5-phosphate
Glycolysis
Glyoxylate and dicarboxylate metabolism
Glycolaldehyde
Pyridoxine
2.7.1.35
3.1.3.74
Pyridoxine phosphate
Pentose phosphate pathway
1.1.99.9
2.6.99.2
2-(Acetamidomethylene)-3-(hydroxymethyl) 3-succinate
5-Pyridoxolactone
α-Hydroxymethyl succinate semialdehyde
3.5.1.66
1.14.12.5
3-Hydroxy-4-hydroxymethyl-2-methylpyridine-5-carboxylate
Isopyridoxal
4-Hydroxy-L-threonine
3-Amino-2-oxopropyl phosphate
4.2.3.1
spontaneous
2-Oxo-3-hydroxy-4-phosphobutanoate
Pentose phosphate pathway
1.2.1.72
1.1.1.290
2.6.1.52
1.1.1.262
D-Erythrose 4-phosphate
4-Phospho-D-erythronate
O-Phospho-4-hydroxy-L-threonine
2-Amino-3-oxo-4-phosphonobutanoate
00750 11/7/14
(c) Kanehisa Laboratories

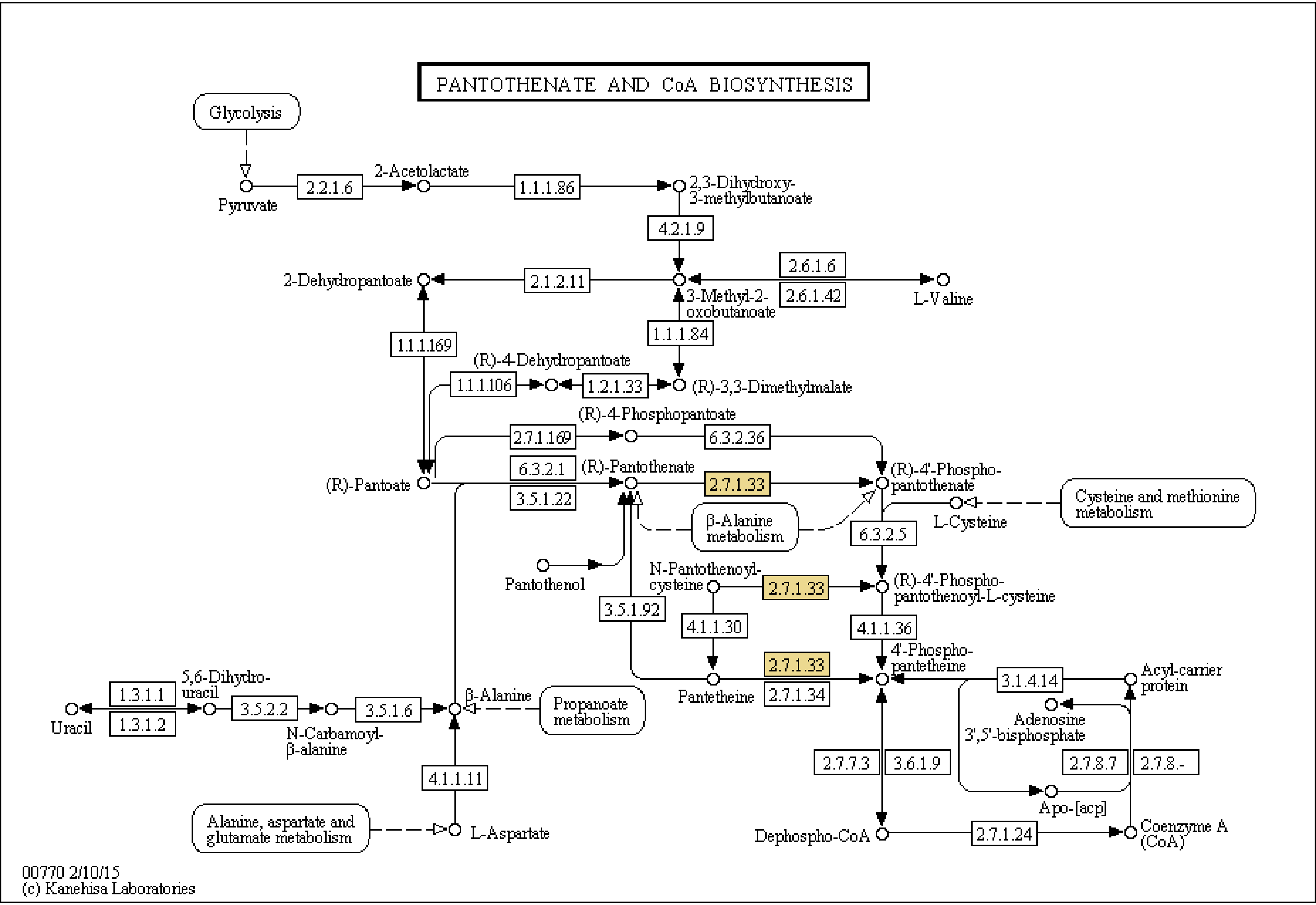
PANTOTHENATE AND CoA BIOSYNTHESIS
Glycolysis
Pyruvate
2.2.1.6
2-Acetolactate
1.1.1.86
2,3-Dihydroxy-3-methylbutanoate
4.2.1.9
2-Dehydropantoate
2.1.2.11
3-Methyl-2-oxobutanoate
2.6.1.6
2.6.1.42
L-Valine
1.1.1.169
1.1.1.84
(R)-4-Dehydropantoate
1.1.1.106
1.2.1.33
(R)-3,3-Dimethylmalate
(R)-4-Phosphopantoate
2.7.1.169
6.3.2.36
6.3.2.1
(R)-Pantothenate
(R)-Pantoate
3.5.1.22
2.7.1.33
(R)-4'-Phospho-pantothenate
Cysteine and methionine metabolism
L-Cysteine
β-Alanine metabolism
6.3.2.5
Pantothenol
N-Pantothenoyl-cysteine
2.7.1.33
(R)-4'-Phospho-pantothenoyl-L-cysteine
3.5.1.92
4.1.1.30
4.1.1.36
4'-Phospho-pantetheine
2.7.1.33
2.7.1.34
Pantetheine
3.1.4.14
Acyl-carrier protein
Uracil
1.3.1.1
1.3.1.2
5,6-Dihydro-uracil
3.5.2.2
N-Carbamoyl-β-alanine
3.5.1.6
β-Alanine
Propanoate metabolism
Adenosine 3',5'-bisphosphate
2.7.7.3
3.6.1.9
2.7.8.7
2.7.8.-
4.1.1.11
Apo-[acp]
Alanine, aspartate and glutamate metabolism
L-Aspartate
Dephospho-CoA
2.7.1.24
Coenzyme A (CoA)
00770 2/10/15
(c) Kanehisa Laboratories

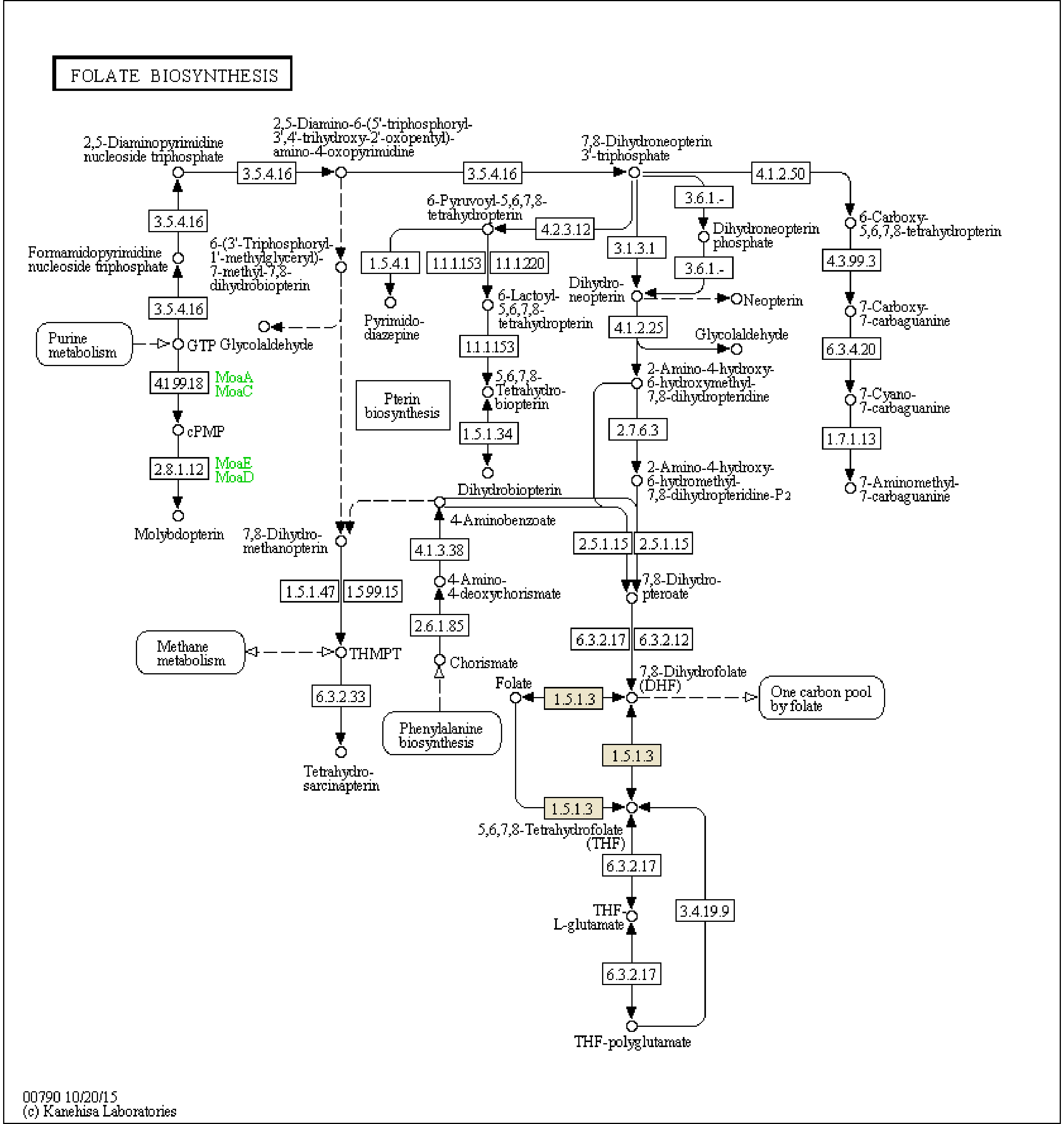

FOLATE BIOSYNTHESIS
2,5-Diaminopyrimidine nucleoside triphosphate
2,5-Diamino-6-(5'-triphosphoryl-3',4'-trihydroxy-2'-oxopentyl)-amino-4-oxopyrimidine
7,8-Dihydroneopterin 3'-triphosphate
3.5.4.16
3.5.4.16
4.1.2.50
3.6.1.-
3.5.4.16
6-Pyruvoyl-5,6,7,8-tetrahydropterin
4.2.3.12
Dihydroneopterin phosphate
6-Carboxy-5,6,7,8-tetrahydropterin
Formamidopyrimidine nucleoside triphosphate
6-(3'-Triphosphoryl-1'-methylglyceryl)-7-methyl-7,8-dihydrobiopterin
1.5.4.1
1.1.1.153
1.1.1.220
3.1.3.1
3.6.1.-
4.3.99.3
3.5.4.16
Dihydro-neopterin
Neopterin
6-Lactoyl-5,6,7,8-tetrahydropterin
Pyrimido-diazepine
7-Carboxy-7-carbaguanine
Purine metabolism
GTP
Glycolaldehyde
4.1.2.25
Glycolaldehyde
1.1.1.153
6.3.4.20
4.1.99.18
MoaA
MoaC
2-Amino-4-hydroxy-6-hydroxymethyl-7,8-dihydropteridine
5,6,7,8-Tetrahydro-biopterin
Pterin biosynthesis
7-Cyano-7-carbaguanine
cPMP
1.5.1.34
2.7.6.3
1.7.1.13
2.8.1.12
MoaE
MoaD
2-Amino-4-hydroxy-6-hydromethyl-7,8-dihydropteridine-P2
7-Aminomethyl-7-carbaguanine
Dihydrobiopterin
Molybdopterin
7,8-Dihydro-methanopterin
4-Aminobenzoate
4.1.3.38
2.5.1.15
2.5.1.15
4-Amino-4-deoxychorismate
7,8-Dihydro-pteroate
1.5.1.47
1.5.99.15
2.6.1.85
6.3.2.17
6.3.2.12
Methane metabolism
THMPT
Chorismate
Folate
7,8-Dihydrofolate (DHF)
1.5.1.3
One carbon pool by folate
6.3.2.33
Phenylalanine biosynthesis
1.5.1.3
Tetrahydro-sarcinapterin
1.5.1.3
5,6,7,8-Tetrahydrofolate (THF)
6.3.2.17
THF-L-glutamate
3.4.19.9
6.3.2.17
THF-polyglutamate
00790 10/20/15
(c) Kanehisa Laboratories

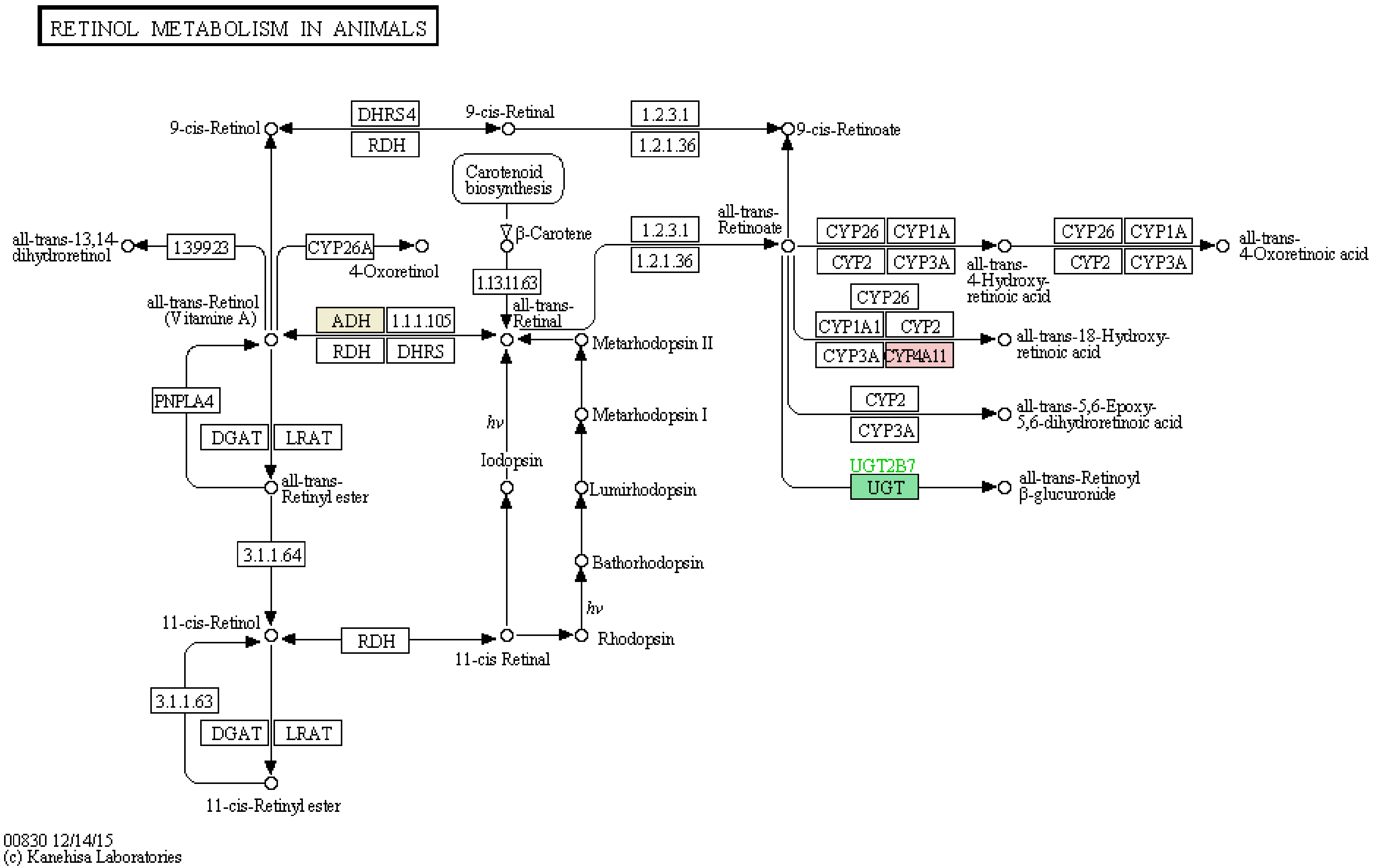

RETINOL METABOLISM IN ANIMALS
9-cis-Retinol
DHRS4
RDH
9-cis-Retinal
1.2.3.1
1.2.1.36
9-cis-Retinoate
Carotenoid biosynthesis
β-Carotene
1.13.11.63
all-trans-13,14-dihydroretinol
1.3.99.23
CYP26A
4-Oxoretinol
all-trans-Retinol (Vitamine A)
ADH
1.1.1.105
RDH
DHRS
all-trans-Retinal
all-trans-Retinoate
CYP26
CYP1A
CYP2
CYP3A
all-trans-4-Hydroxy-retinoic acid
all-trans-4-Oxoretinoic acid
CYP1A1
CYP4A11
all-trans-18-Hydroxy-retinoic acid
all-trans-5,6-Epoxy-5,6-dihydroretinoic acid
UGT2B7
UGT
all-trans-Retinoyl β-glucuronide
PNPLA4
DGAT
LRAT
all-trans-Retinyl ester
3.1.1.64
Metarhodopsin II
Metarhodopsin I
Lumirhodopsin
Bathorhodopsin
Rhodopsin
Iodopsin
hν
11-cis-Retinol
11-cis Retinal
3.1.1.63
11-cis-Retinyl ester
00830 12/14/15
(c) Kanehisa Laboratories

PORPHYRIN AND CHLOROPHYLL METABOLISM

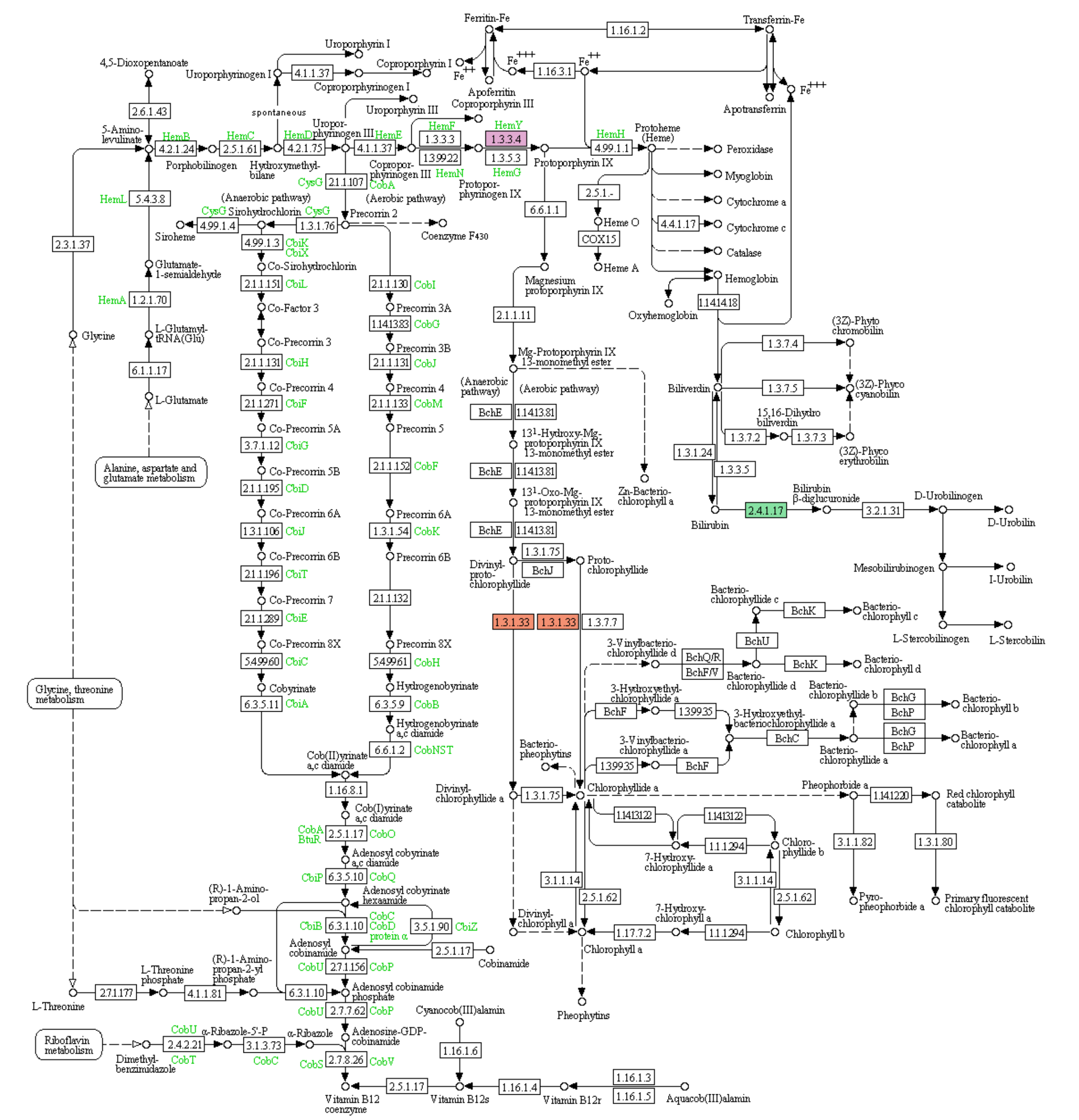

00860 12/14/15
(c) Kanehisa Laboratories

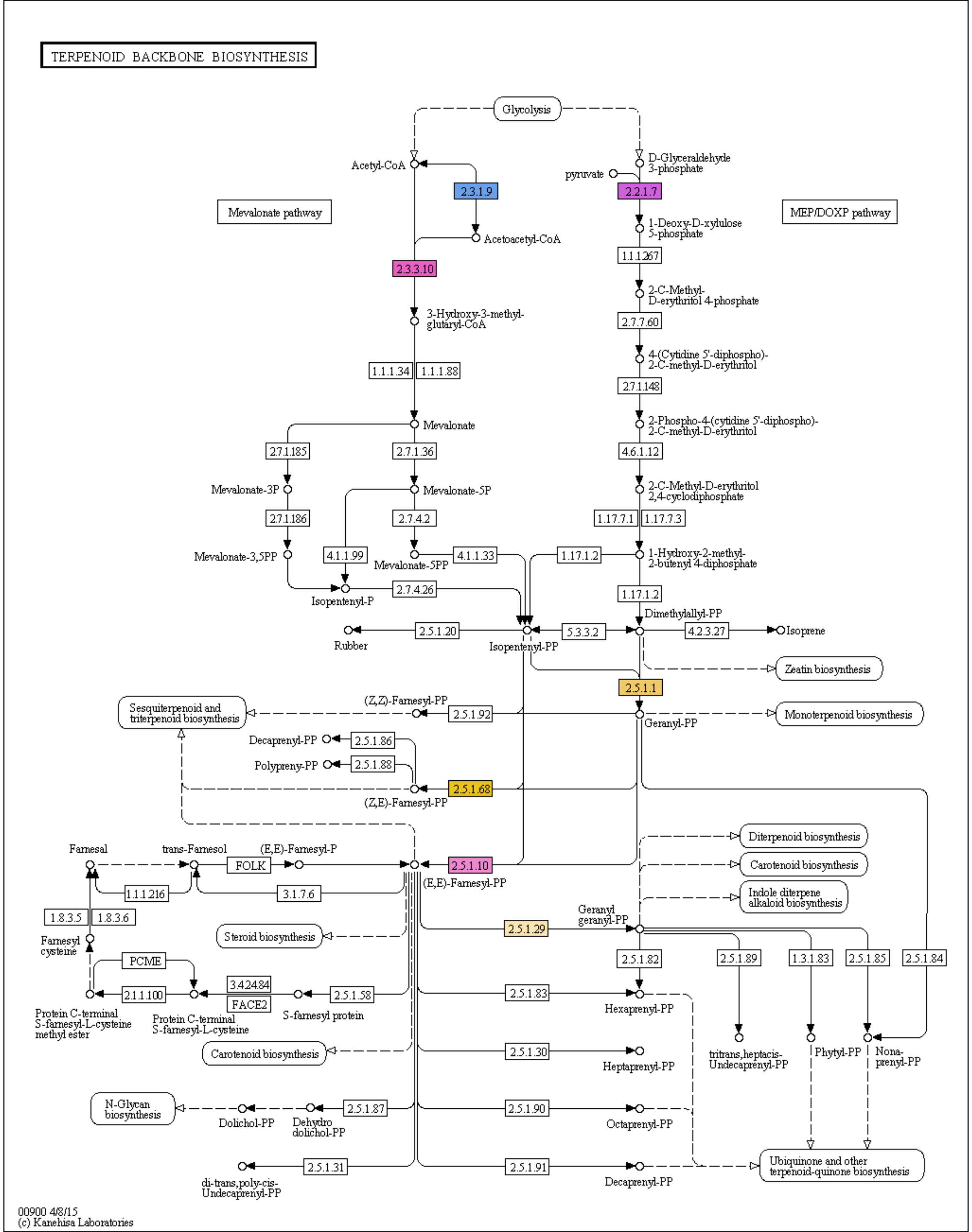
TERPENOID BACKBONE BIOSYNTHESIS
Glycolysis
Acetyl-CoA
D-Glyceraldehyde 3-phosphate
pyruvate
2.3.1.9
2.2.1.7
Mevalonate pathway
MEP/DOXP pathway
Acetoacetyl-CoA
1-Deoxy-D-xylulose 5-phosphate
2.3.3.10
1.1.1.267
2-C-Methyl-D-erythritol 4-phosphate
3-Hydroxy-3-methyl-glutaryl-CoA
2.7.7.60
4-(Cytidine 5'-diphospho)-2-C-methyl-D-erythritol
1.1.1.34
1.1.1.88
2.7.1.148
Mevalonate
2-Phospho-4-(cytidine 5'-diphospho)-2-C-methyl-D-erythritol
2.7.1.185
2.7.1.36
4.6.1.12
Mevalonate-3P
Mevalonate-5P
2-C-Methyl-D-erythritol 2,4-cyclodiphosphate
2.7.1.186
2.7.4.2
1.17.7.1
1.17.7.3
Mevalonate-3,5PP
4.1.1.99
4.1.1.33
1.17.1.2
1-Hydroxy-2-methyl-2-butenyl 4-diphosphate
Mevalonate-5PP
2.7.4.26
1.17.1.2
Isopentenyl-P
Dimethylallyl-PP
2.5.1.20
5.3.3.2
4.2.3.27
Isoprene
Rubber
Isopentenyl-PP
Zeatin biosynthesis
2.5.1.1
(Z,Z)-Farnesyl-PP
Sesquiterpenoid and triterpenoid biosynthesis
2.5.1.92
Geranyl-PP
Monoterpenoid biosynthesis
Decaprenyl-PP
2.5.1.86
Polypreny-PP
2.5.1.88
2.5.1.68
(Z,E)-Farnesyl-PP
Diterpenoid biosynthesis
Farnesal
trans-Farnesol
(E,E)-Farnesyl-P
FOLK
2.5.1.10
Carotenoid biosynthesis
(E,E)-Farnesyl-PP
1.1.1.216
3.1.7.6
Indole diterpene alkaloid biosynthesis
1.8.3.5
1.8.3.6
Geranyl geranyl-PP
2.5.1.29
Farnesyl cysteine
Steroid biosynthesis
PCME
2.5.1.82
2.5.1.89
1.3.1.83
2.5.1.85
2.5.1.84
3.4.24.84
2.1.1.100
2.5.1.58
2.5.1.83
FACE2
Protein C-terminal S-farnesyl-L-cysteine methyl ester
Protein C-terminal S-farnesyl-L-cysteine
S-farnesyl protein
Hexaprenyl-PP
tritrans,heptacis-Undecaprenyl-PP
Phytyl-PP
Nona-prenyl-PP
Carotenoid biosynthesis
2.5.1.30
Heptaprenyl-PP
N-Glycan biosynthesis
2.5.1.87
2.5.1.90
Dolichol-PP
Dehydro dolichol-PP
Octaprenyl-PP
2.5.1.31
2.5.1.91
Ubiquinone and other terpenoid-quinone biosynthesis
di-trans,poly-cis-Undecaprenyl-PP
Decaprenyl-PP
00900 4/8/15
(c) Kanehisa Laboratories

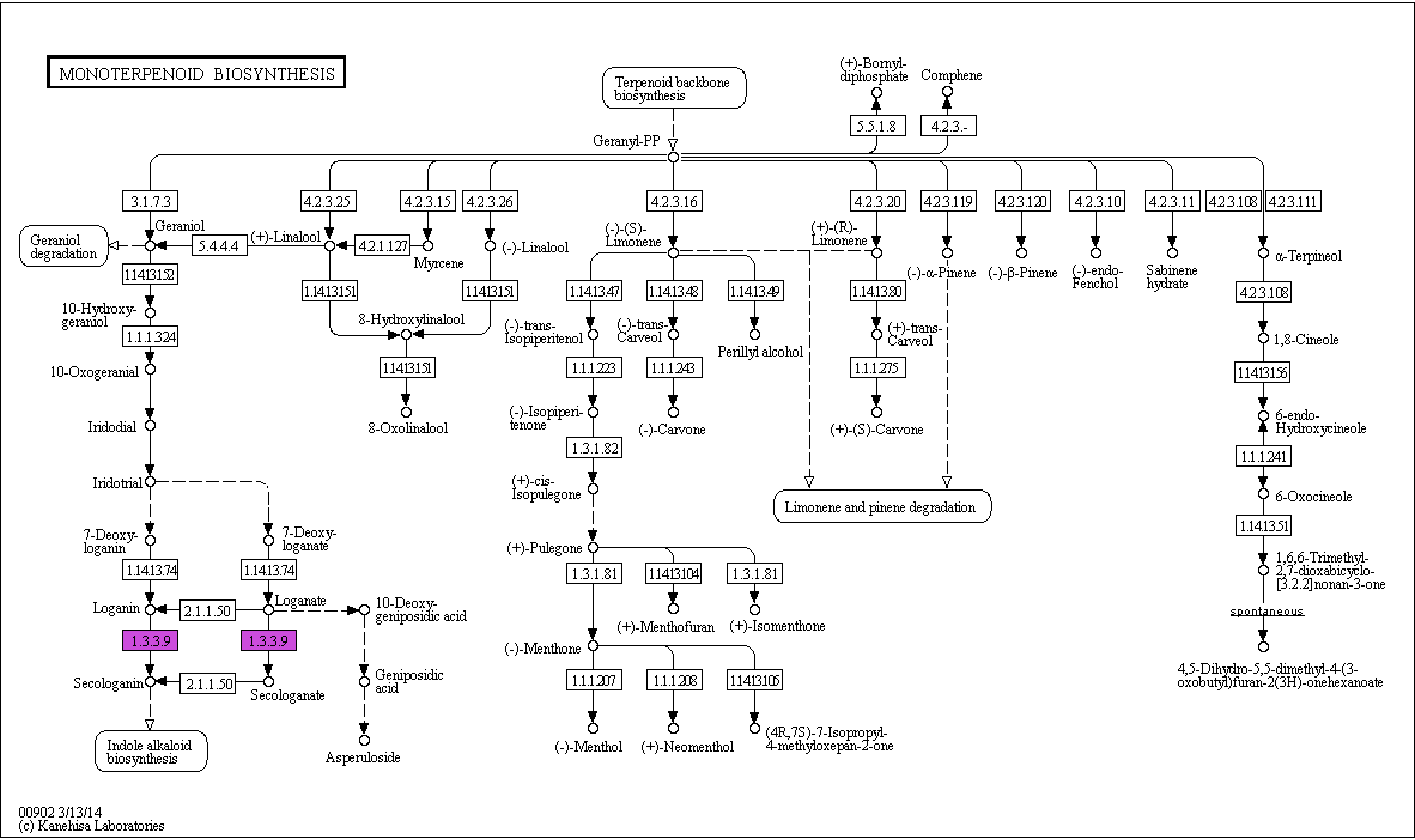

MONOTERPENOID BIOSYNTHESIS
Terpenoid backbone biosynthesis
Geranyl-PP
(+)-Bornyl-diphosphate
Comphene
5.5.1.8
4.2.3.-
3.1.7.3
4.2.3.25
4.2.3.15
4.2.3.26
4.2.3.16
4.2.3.20
4.2.3.119
4.2.3.120
4.2.3.10
4.2.3.11
4.2.3.108
4.2.3.111
Geraniol degradation
Geraniol
5.4.4.4
(+)-Linalool
4.2.1.127
Myrcene
(-)-Linalool
1.14.13.152
10-Hydroxy-geraniol
1.1.1.324
10-Oxogeranial
Iridodial
Iridotrial
7-Deoxy-loganin
7-Deoxy-loganate
1.14.13.74
1.14.13.74
Loganin
2.1.1.50
Loganate
10-Deoxy-geniposidic acid
1.3.3.9
1.3.3.9
Secologanin
2.1.1.50
Secologanate
Geniposidic acid
Asperuloside
Indole alkaloid biosynthesis
1.14.13.151
1.14.13.151
8-Hydroxylinalool
1.14.13.151
8-Oxolinalool
(-)-(S)-Limonene
(+)-(R)-Limonene
1.14.13.47
1.14.13.48
1.14.13.49
1.14.13.80
(-)-trans-Isopiperitenol
(-)-trans-Carveol
Perillyl alcohol
(+)-trans-Carveol
1.1.1.223
1.1.1.243
1.1.1.275
(-)-Isopiperi-tenone
(-)-Carvone
(+)-(S)-Carvone
1.3.1.82
(+)-cis-Isopulegone
(+)-Pulegone
1.3.1.81
1.14.13.104
1.3.1.81
(+)-Menthofuran
(+)-Isomenthone
(-)-Menthone
1.1.1.207
1.1.1.208
1.14.13.105
(-)-Menthol
(+)-Neomenthol
(4R,7S)-7-Isopropyl-4-methyloxepan-2-one
(-)-α-Pinene
(-)-β-Pinene
(-)-endo-Fenchol
Sabinene hydrate
Limonene and pinene degradation
α-Terpineol
4.2.3.108
1,8-Cineole
1.14.13.156
6-endo-Hydroxycineole
1.1.1.241
6-Oxocineole
1.14.13.51
1,6,6-Trimethyl-2,7-dioxabicyclo-[3.2.2]nonan-3-one
spontaneous
4,5-Dihydro-5,5-dimethyl-4-(3-oxobutyl)furan-2(3H)-onehexanoate
00902 3/13/14
(c) Kanehisa Laboratories

CAROTENOID BIOSYNTHESIS

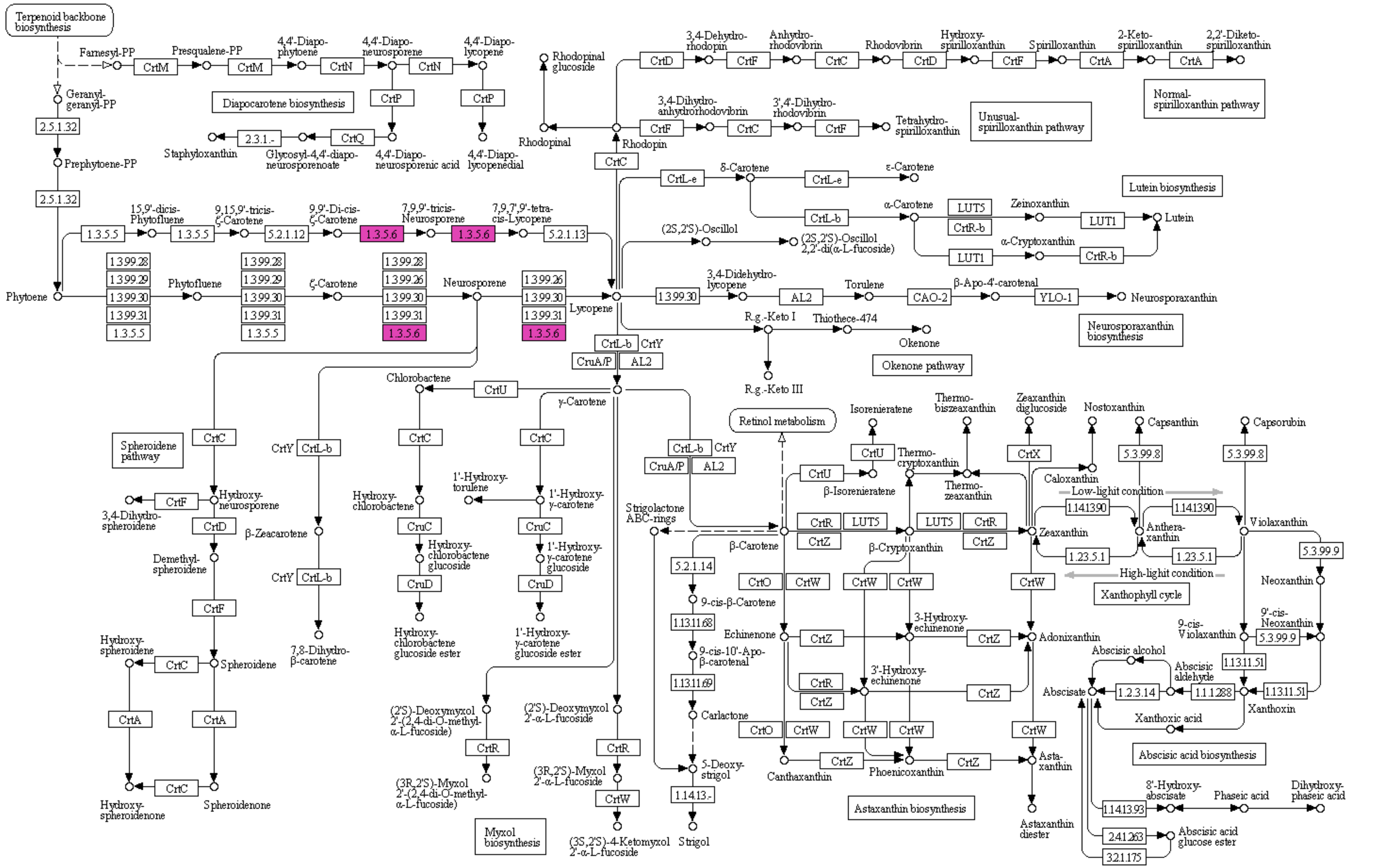

00906 6/8/15
(c) Kanehisa Laboratories

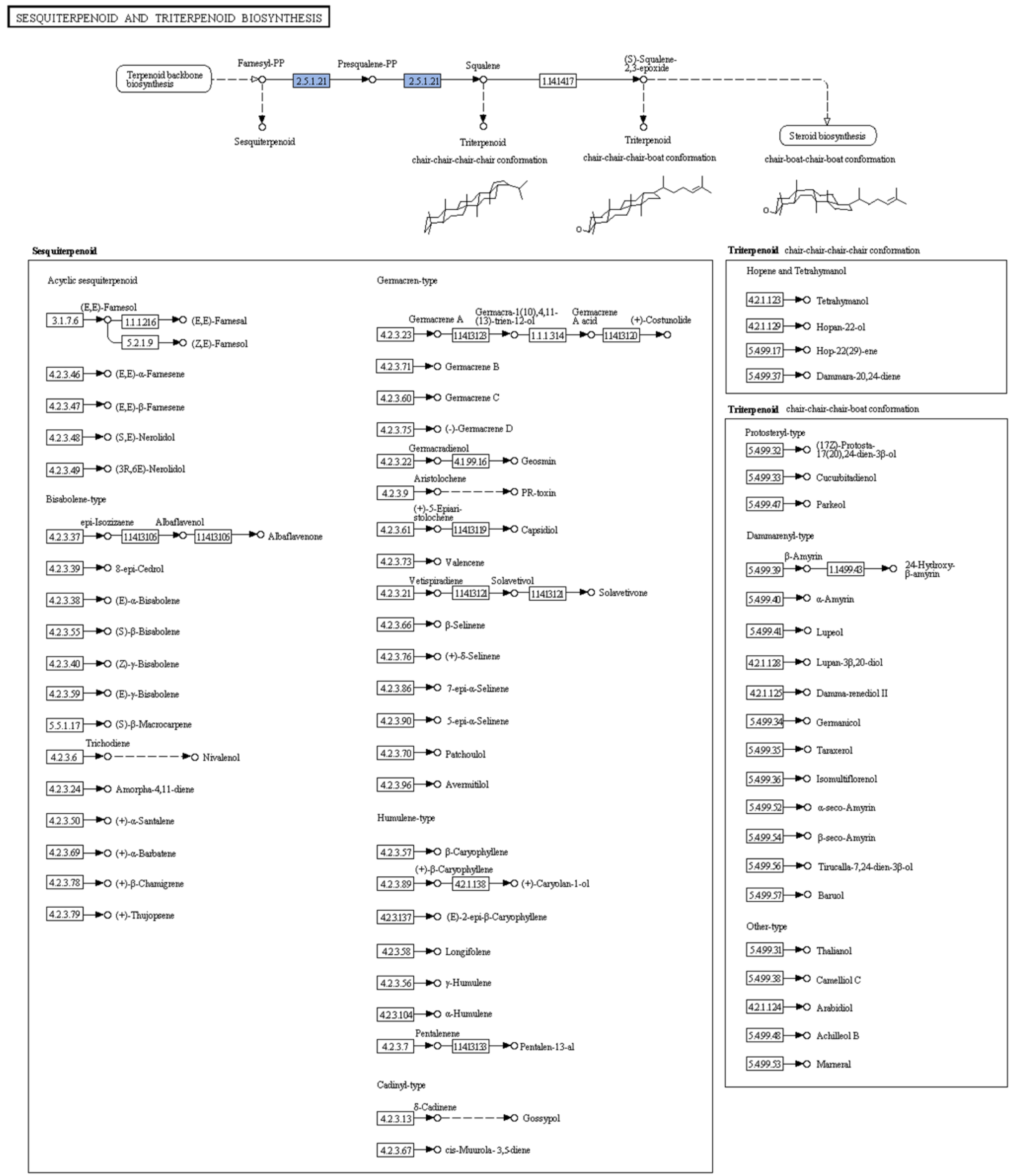

00909 12/14/15
(c) Kanehisa Laboratories

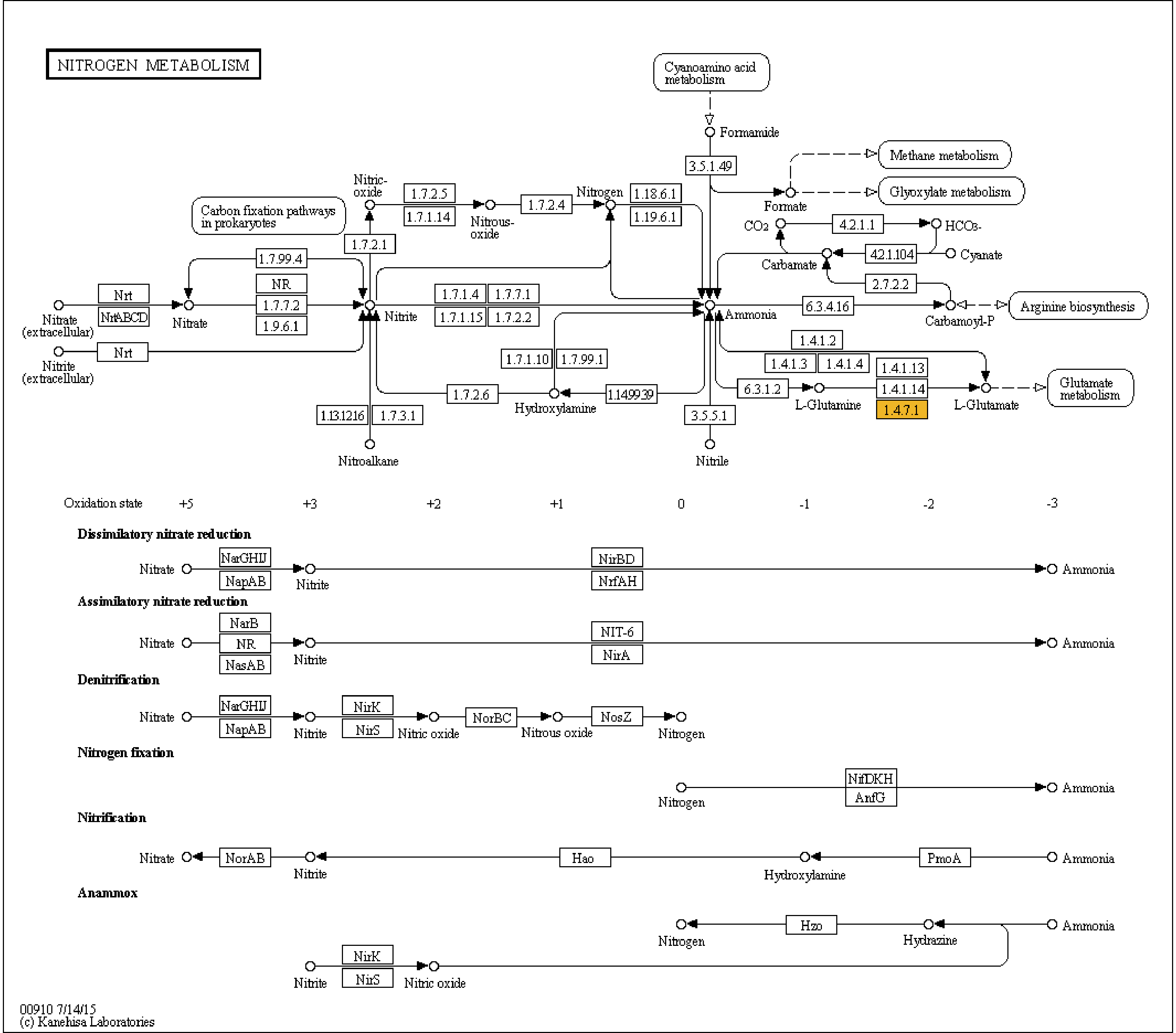
NITROGEN METABOLISM
Cyanoamino acid metabolism
Formamide
3.5.1.49
Methane metabolism
Glyoxylate metabolism
Formate
Nitric-oxide
1.7.2.5
1.7.1.14
Nitrous-oxide
1.7.2.4
Nitrogen
1.18.6.1
1.19.6.1
CO2
4.2.1.1
HCO3-
Carbon fixation pathways in prokaryotes
1.7.2.1
1.7.99.4
NR
1.7.7.2
1.9.6.1
Nrt
NrtABCD
Nitrate (extracellular)
Nitrate
Nitrite (extracellular)
Nitrite
1.7.1.4
1.7.7.1
1.7.1.15
1.7.2.2
Carbamate
4.2.1.104
Cyanate
2.7.2.2
Ammonia
6.3.4.16
Carbamoyl-P
Arginine biosynthesis
1.4.1.2
1.4.1.3
1.4.1.4
1.4.1.13
1.4.1.14
1.4.7.1
1.7.1.10
1.7.99.1
1.7.2.6
Hydroxylamine
1.14.99.39
6.3.1.2
L-Glutamine
L-Glutamate
Glutamate metabolism
1.13.12.16
1.7.3.1
3.5.5.1
Nitroalkane
Nitrile
Oxidation state
+5
+3
+2
+1
0
-1
-2
-3
Dissimilatory nitrate reduction
NarGHIJ
NapAB
NirBD
NrfAH
Nitrate
Nitrite
Ammonia
Assimilatory nitrate reduction
NarB
NR
NasAB
NIT-6
NirA
Nitrate
Nitrite
Ammonia
Denitrification
NarGHIJ
NapAB
NirK
NirS
NorBC
NosZ
Nitrate
Nitrite
Nitric oxide
Nitrous oxide
Nitrogen
Nitrogen fixation
NifDKH
AnfG
Nitrogen
Ammonia
Nitrification
NorAB
Hao
PmoA
Nitrate
Nitrite
Hydroxylamine
Ammonia
Anammox
Hzo
Nitrogen
Hydrazine
Ammonia
NirK
NirS
Nitrite
Nitric oxide
00910 7/14/15
(c) Kanehisa Laboratories

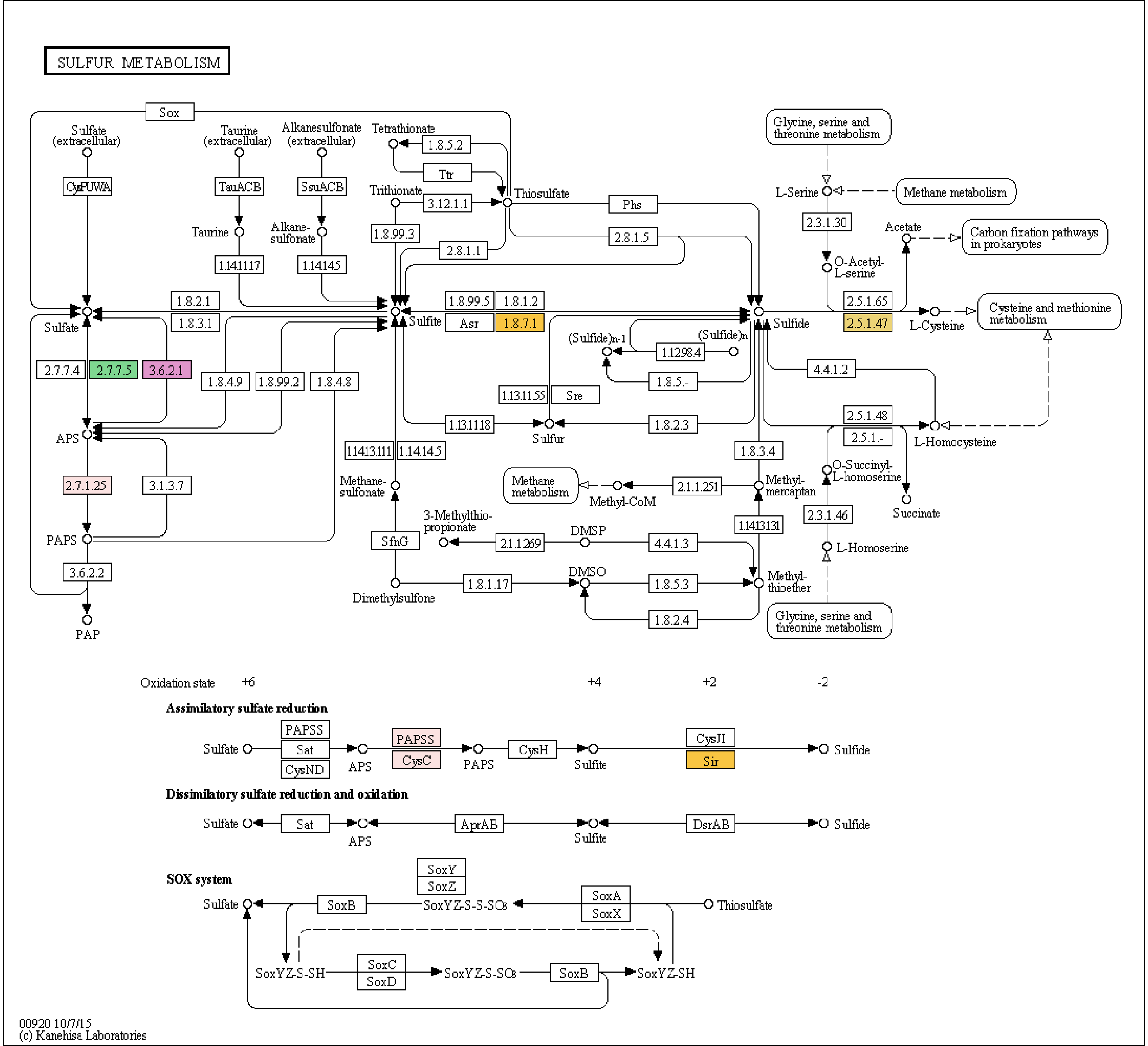
SULFUR METABOLISM
Sox
Sulfate (extracellular)
Taurine (extracellular)
Alkanesulfonate (extracellular)
Tetrathionate
1.8.5.2
Ttr
Trithionate
Thiosulfate
3.12.1.1
Phs
2.8.1.5
CysPUWA
TauACB
SsuACB
Taurine
Alkane-sulfonate
1.8.99.3
2.8.1.1
1.14.11.17
1.14.14.5
Glycine, serine and threonine metabolism
L-Serine
Methane metabolism
2.3.1.30
Acetate
Carbon fixation pathways in prokaryotes
O-Acetyl-L-serine
1.8.2.1
1.8.3.1
Sulfate
Sulfite
1.8.99.5
1.8.1.2
Asr
1.8.7.1
Sulfide
2.5.1.65
2.5.1.47
L-Cysteine
Cysteine and methionine metabolism
(Sulfide)n-1
(Sulfide)n
1.12.98.4
1.8.5.-
2.7.7.4
2.7.7.5
3.6.2.1
1.8.4.9
1.8.99.2
1.8.4.8
4.4.1.2
1.13.11.55
Sre
2.5.1.48
1.13.11.18
Sulfur
1.8.2.3
L-Homocysteine
APS
2.5.1.-
1.14.13.111
1.14.14.5
1.8.3.4
O-Succinyl-L-homoserine
2.7.1.25
3.1.3.7
Methane-sulfonate
Methane metabolism
Methyl-CoM
2.1.1.251
Methyl-mercaptan
Succinate
3-Methylthio-propionate
DMSP
1.14.13.131
2.3.1.46
PAPS
SfnG
2.1.1.269
4.4.1.3
L-Homoserine
3.6.2.2
DMSO
Methyl-thioether
Dimethylsulfone
1.8.1.17
1.8.5.3
1.8.2.4
Glycine, serine and threonine metabolism
PAP
Oxidation state
+6
+4
+2
-2
Assimilatory sulfate reduction
Sulfate
PAPSS
Sat
CysND
APS
PAPSS
CysC
PAPS
CysH
Sulfite
CysJI
Sir
Sulfide
Dissimilatory sulfate reduction and oxidation
Sulfate
Sat
APS
AprAB
Sulfite
DsrAB
Sulfide
SOX system
SoxY
SoxZ
Sulfate
SoxB
SoxYZ-S-S-SO3
SoxA
SoxX
Thiosulfate
SoxYZ-S-SH
SoxC
SoxD
SoxYZ-S-SO3
SoxB
SoxYZ-SH
00920 10/7/15
(c) Kanehisa Laboratories

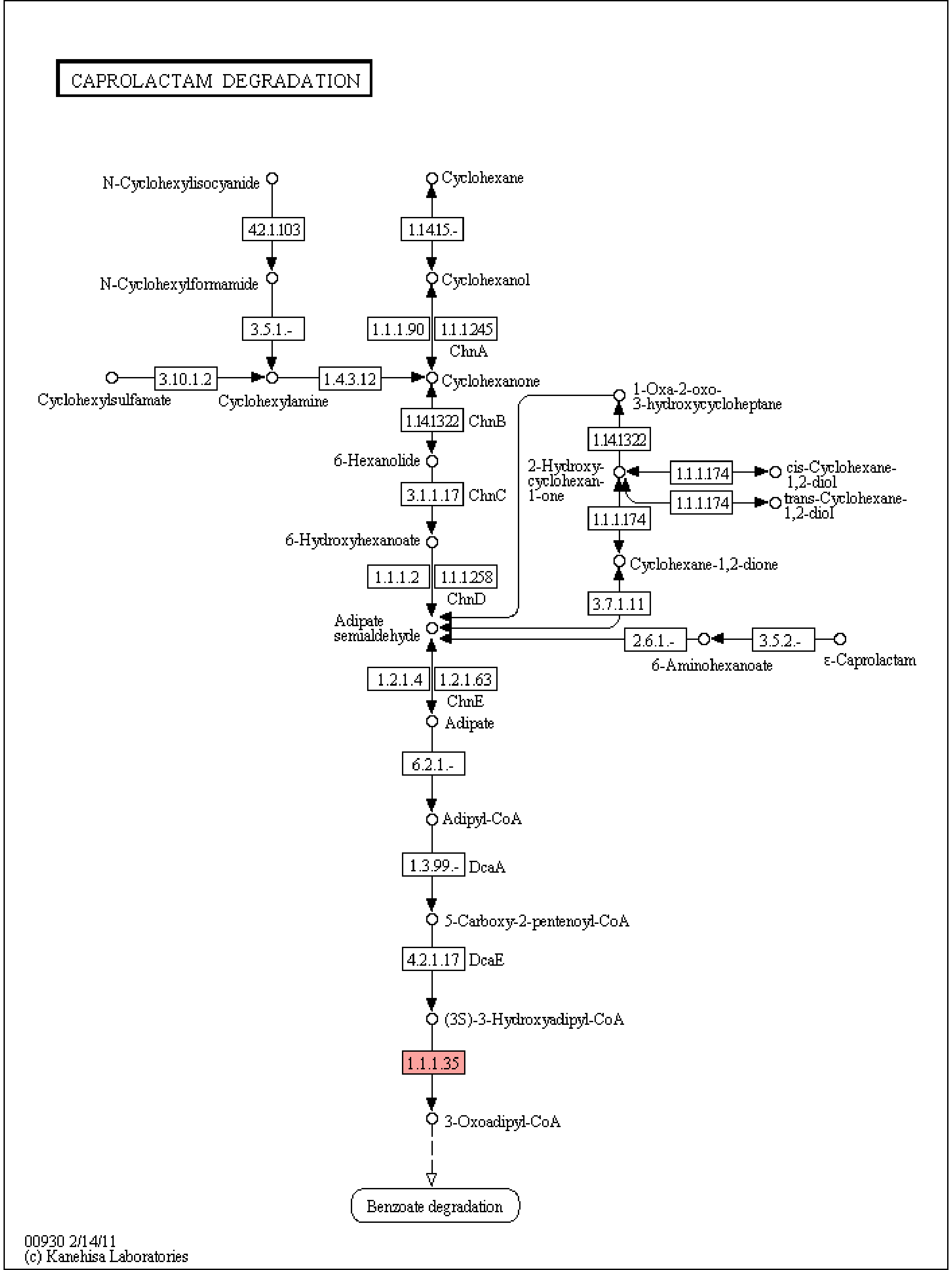
CAPROLACTAM DEGRADATION
N-Cyclohexylisocyanide
4.2.1.103
N-Cyclohexylformamide
3.5.1.-
Cyclohexylsulfamate
3.10.1.2
Cyclohexylamine
1.4.3.12
Cyclohexane
1.14.15.-
Cyclohexanol
1.1.1.90
1.1.1.245
ChnA
Cyclohexanone
1.14.13.22
ChnB
6-Hexanolide
3.1.1.17
ChnC
6-Hydroxyhexanoate
1.1.1.2
1.1.1.258
ChnD
Adipate semialdehyde
1.2.1.4
1.2.1.63
ChnE
Adipate
6.2.1.-
Adipyl-CoA
1.3.99.-
DcaA
5-Carboxy-2-pentenoyl-CoA
4.2.1.17
DcaE
(3S)-3-Hydroxyadipyl-CoA
1.1.1.35
3-Oxoadipyl-CoA
Benzoate degradation
1-Oxa-2-oxo-3-hydroxycycloheptane
1.14.13.22
2-Hydroxy-cyclohexan-1-one
1.1.1.174
cis-Cyclohexane-1,2-diol
1.1.1.174
trans-Cyclohexane-1,2-diol
1.1.1.174
Cyclohexane-1,2-dione
3.7.1.11
2.6.1.-
6-Aminohexanoate
3.5.2.-
ε-Caprolactam
00930 2/14/11
(c) Kanehisa Laboratories

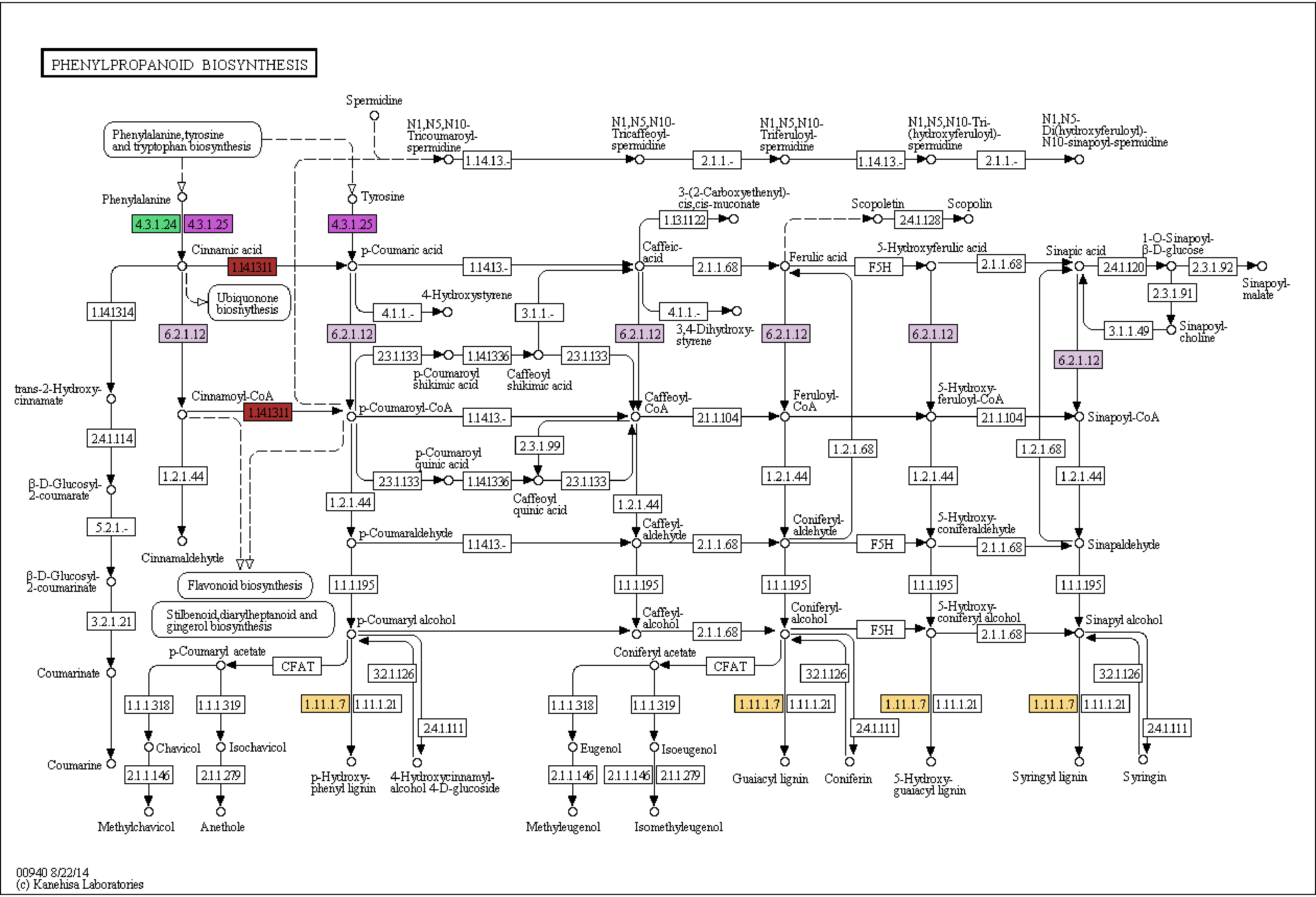
PHENYLPROPANOID BIOSYNTHESIS
Phenylalanine, tyrosine and tryptophan biosynthesis
Spermidine
N1,N5,N10-Tricoumaroyl-spermidine
1.14.13.-
N1,N5,N10-Tricaffeoyl-spermidine
2.1.1.-
N1,N5,N10-Triferuloyl-spermidine
1.14.13.-
N1,N5,N10-Tri-(hydroxyferuloyl)-spermidine
2.1.1.-
N1,N5-Di(hydroxyferuloyl)-N10-sinapoyl-spermidine
Phenylalanine
4.3.1.24
4.3.1.25
Tyrosine
4.3.1.25
Cinnamic acid
1.14.13.11
p-Coumaric acid
1.14.13.-
Caffeic-acid
1.13.11.22
3-(2-Carboxyethenyl)-cis,cis-muconate
2.1.1.68
Ferulic acid
Scopoletin
2.4.1.128
Scopolin
F5H
5-Hydroxyferulic acid
2.1.1.68
Sinapic acid
2.4.1.120
1-O-Sinapoyl-β-D-glucose
2.3.1.92
Sinapoyl-malate
2.3.1.91
3.1.1.49
Sinapoyl-choline
Ubiquonone biosynthesis
1.14.13.14
4.1.1.-
4-Hydroxystyrene
3.1.1.-
4.1.1.-
3,4-Dihydroxy-styrene
6.2.1.12
6.2.1.12
6.2.1.12
6.2.1.12
6.2.1.12
6.2.1.12
2.3.1.133
p-Coumaroyl shikimic acid
1.14.13.36
Caffeoyl shikimic acid
2.3.1.133
trans-2-Hydroxy-cinnamate
Cinnamoyl-CoA
1.14.13.11
p-Coumaroyl-CoA
1.14.13.-
Caffeoyl-CoA
2.1.1.104
Feruloyl-CoA
5-Hydroxy-feruloyl-CoA
2.1.1.104
Sinapoyl-CoA
2.4.1.114
2.3.1.99
p-Coumaroyl quinic acid
1.2.1.68
1.2.1.68
β-D-Glucosyl-2-coumarate
1.2.1.44
2.3.1.133
1.14.13.36
2.3.1.133
Caffeoyl quinic acid
1.2.1.44
1.2.1.44
1.2.1.44
1.2.1.44
1.2.1.44
5.2.1.-
Cinnamaldehyde
p-Coumaraldehyde
1.14.13.-
Caffeyl-aldehyde
2.1.1.68
Coniferyl-aldehyde
F5H
5-Hydroxy-coniferaldehyde
2.1.1.68
Sinapaldehyde
β-D-Glucosyl-2-coumarinate
Flavonoid biosynthesis
1.1.1.195
1.1.1.195
1.1.1.195
1.1.1.195
1.1.1.195
3.2.1.21
Stilbenoid, diarylheptanoid and gingerol biosynthesis
p-Coumaryl alcohol
Caffeyl-alcohol
2.1.1.68
Coniferyl-alcohol
F5H
5-Hydroxy-coniferyl alcohol
2.1.1.68
Sinapyl alcohol
p-Coumaryl acetate
CFAT
Coniferyl acetate
CFAT
Coumarinate
3.2.1.126
3.2.1.126
3.2.1.126
1.1.1.318
1.1.1.319
1.11.1.7
1.11.1.21
1.1.1.318
1.1.1.319
1.11.1.7
1.11.1.21
1.11.1.7
1.11.1.21
1.11.1.7
1.11.1.21
2.4.1.111
2.4.1.111
2.4.1.111
Chavicol
Isochavicol
Eugenol
Isoeugenol
Coumarine
2.1.1.146
2.1.1.279
2.1.1.146
2.1.1.146
2.1.1.279
p-Hydroxy-phenyl lignin
4-Hydroxycinnamyl-alcohol 4-D-glucoside
Guaiacyl lignin
Coniferin
5-Hydroxy-guaiacyl lignin
Syringyl lignin
Syringin
Methylchavicol
Anethole
Methyleugenol
Isomethyleugenol
00940 8/22/14
(c) Kanehisa Laboratories

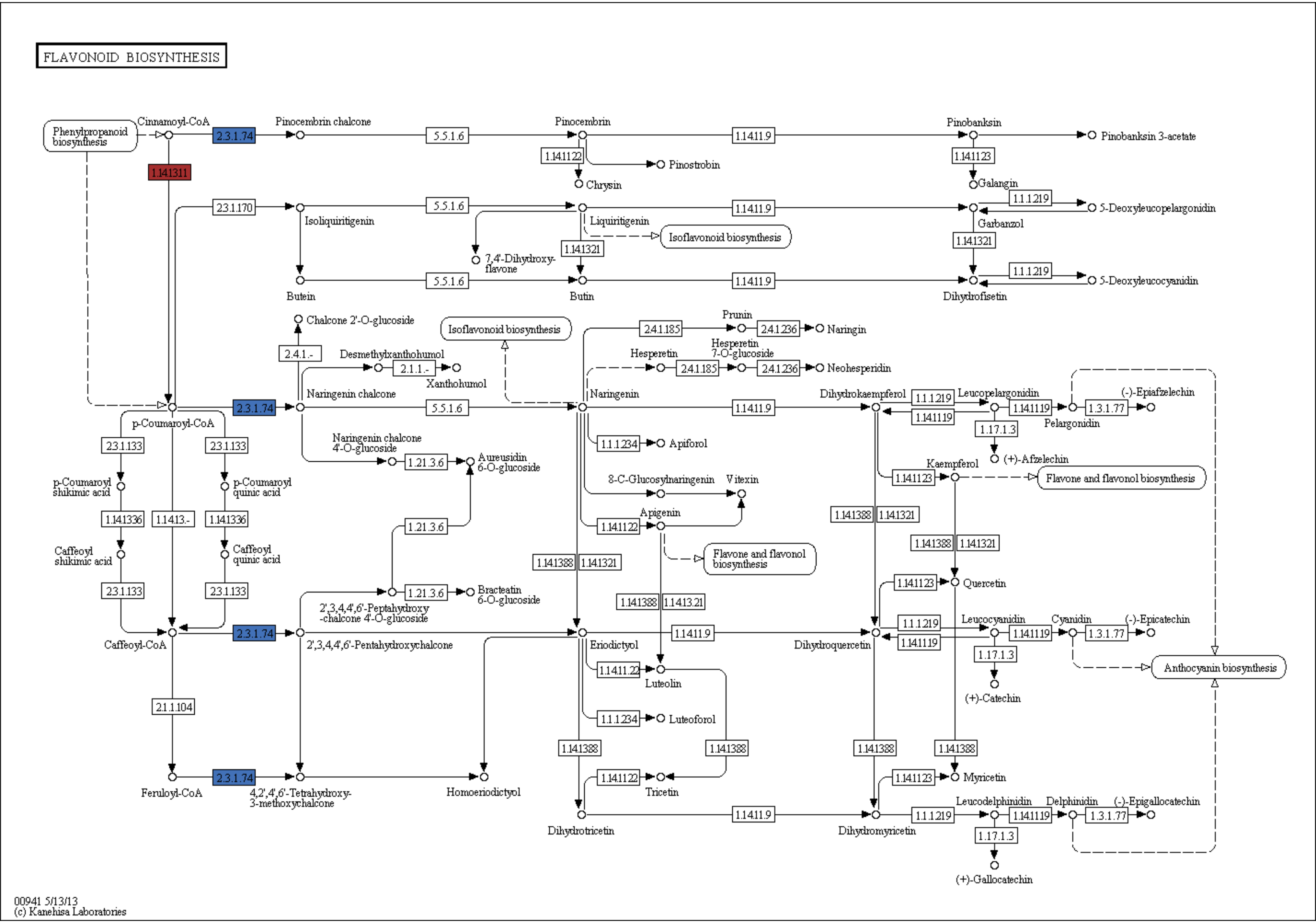
FLAVONOID BIOSYNTHESIS
Phenylpropanoid biosynthesis
Cinnamoyl-CoA
2.3.1.74
Pinocembrin chalcone
5.5.1.6
Pinocembrin
1.14.11.9
Pinobanksin
Pinobanksin 3-acetate
1.14.13.11
1.14.11.22
Pinostrobin
Chrysin
1.14.11.23
Galangin
2.3.1.170
Isoliquiritigenin
Liquiritigenin
Isoflavonoid biosynthesis
1.1.1.219
5-Deoxyleucopelargonidin
Garbanzol
1.14.13.21
7,4'-Dihydroxy-flavone
Butein
Butin
Dihydrofisetin
5-Deoxyleucocyanidin
Chalcone 2'-O-glucoside
2.4.1.-
Desmethylxanthohumol
2.1.1.-
Xanthohumol
Naringenin chalcone
Naringenin
Prunin
2.4.1.185
2.4.1.236
Naringin
Hesperetin
Hesperetin 7-O-glucoside
Neohesperidin
Dihydrokaempferol
Leucopelargonidin
1.14.11.19
Pelargonidin
1.3.1.77
(-)-Epiafzelechin
1.17.1.3
(+)-Afzelechin
Kaempferol
Flavone and flavonol biosynthesis
p-Coumaroyl-CoA
2.3.1.133
p-Coumaroyl shikimic acid
p-Coumaroyl quinic acid
1.14.13.36
1.14.13.-
Caffeoyl shikimic acid
Caffeoyl quinic acid
Naringenin chalcone 4'-O-glucoside
1.21.3.6
Aureusidin 6-O-glucoside
1.1.1.234
Apiforol
8-C-Glucosylnaringenin
Vitexin
Apigenin
1.14.13.88
Quercetin
Bracteatin 6-O-glucoside
2',3,4,4',6'-Peptahydroxy-chalcone 4'-O-glucoside
Caffeoyl-CoA
2',3,4,4',6'-Pentahydroxychalcone
Eriodictyol
Dihydroquercetin
Leucocyanidin
Cyanidin
(-)-Epicatechin
Anthocyanin biosynthesis
(+)-Catechin
Luteolin
Luteoforol
2.1.1.104
Feruloyl-CoA
4,2',4',6'-Tetrahydroxy-3-methoxychalcone
Homoeriodictyol
Tricetin
Myricetin
Dihydrotricetin
Dihydromyricetin
Leucodelphinidin
Delphinidin
(-)-Epigallocatechin
(+)-Gallocatechin
00941 5/13/13
(c) Kanehisa Laboratories

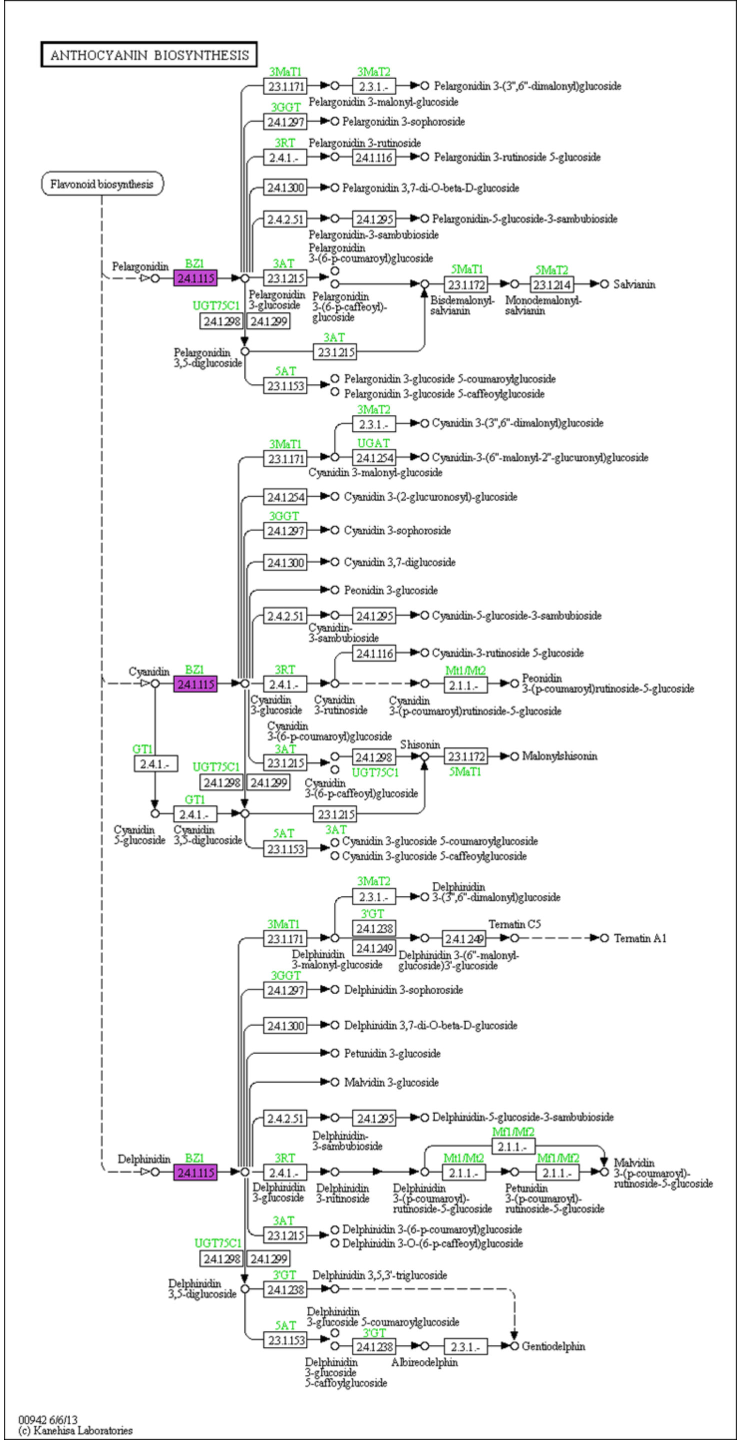
ANTHOCYANIN BIOSYNTHESIS
Flavonoid biosynthesis
Pelargonidin
BZ1
2.4.1.115
3MaT1
2.3.1.171
3MaT2
2.3.1.-
Pelargonidin 3-(3",6"-dimalonyl)glucoside
Pelargonidin 3-malonyl-glucoside
3GGT
2.4.1.297
Pelargonidin 3-sophoroside
3RT
2.4.1.-
Pelargonidin 3-rutinoside
2.4.1.116
Pelargonidin 3-rutinoside 5-glucoside
2.4.1.300
Pelargonidin 3,7-di-O-beta-D-glucoside
2.4.2.51
2.4.1.295
Pelargonidin-5-glucoside-3-sambubioside
Pelargonidin-3-sambubioside
Pelargonidin 3-(6-p-coumaroyl)glucoside
3AT
2.3.1.215
5MaT1
2.3.1.172
5MaT2
2.3.1.214
Salvianin
Pelargonidin 3-glucoside
Pelargonidin 3-(6-p-caffeoyl)-glucoside
Bisdemalonyl-salvianin
Monodemalonyl-salvianin
UGT75C1
2.4.1.298
2.4.1.299
Pelargonidin 3,5-diglucoside
5AT
2.3.1.153
Pelargonidin 3-glucoside 5-coumaroylglucoside
Pelargonidin 3-glucoside 5-caffeoylglucoside
Cyanidin 3-(3",6"-dimalonyl)glucoside
UGAT
2.4.1.254
Cyanidin-3-(6"-malonyl-2"-glucuronyl)glucoside
Cyanidin 3-malonyl-glucoside
Cyanidin 3-(2-glucuronosyl)-glucoside
Cyanidin 3-sophoroside
Cyanidin 3,7-diglucoside
Peonidin 3-glucoside
Cyanidin-5-glucoside-3-sambubioside
Cyanidin-3-sambubioside
Cyanidin-3-rutinoside 5-glucoside
Cyanidin
Mt1/Mt2
2.1.1.-
Peonidin 3-(p-coumaroyl)rutinoside-5-glucoside
Cyanidin 3-glucoside
Cyanidin 3-rutinoside
Cyanidin 3-(p-coumaroyl)rutinoside-5-glucoside
Cyanidin 3-(6-p-coumaroyl)glucoside
GT1
2.4.1.-
Shisonin
Malonylshisonin
Cyanidin 3-(6-p-caffeoyl)glucoside
Cyanidin 5-glucoside
Cyanidin 3,5-diglucoside
Cyanidin 3-glucoside 5-coumaroylglucoside
Cyanidin 3-glucoside 5-caffeoylglucoside
Delphinidin 3-(3",6"-dimalonyl)glucoside
3'GT
2.4.1.238
2.4.1.249
Ternatin C5
Ternatin A1
Delphinidin 3-malonyl-glucoside
Delphinidin 3-(6"-malonyl-glucoside)3'-glucoside
Delphinidin 3-sophoroside
Delphinidin 3,7-di-O-beta-D-glucoside
Petunidin 3-glucoside
Malvidin 3-glucoside
Delphinidin-5-glucoside-3-sambubioside
Delphinidin-3-sambubioside
Mf1/Mf2
Delphinidin
Malvidin 3-(p-coumaroyl)-rutinoside-5-glucoside
Delphinidin 3-glucoside
Delphinidin 3-rutinoside
Delphinidin 3-(p-coumaroyl)-rutinoside-5-glucoside
Petunidin 3-(p-coumaroyl)-rutinoside-5-glucoside
Delphinidin 3-(6-p-coumaroyl)glucoside
Delphinidin 3-O-(6-p-caffeoyl)glucoside
Delphinidin 3,5,3'-triglucoside
Delphinidin 3,5-diglucoside
Delphinidin 3-glucoside 5-coumaroylglucoside
Gentiodelphin
Delphinidin 3-glucoside 5-caffoylglucoside
Albireodelphin
00942 6/6/13
(c) Kanehisa Laboratories

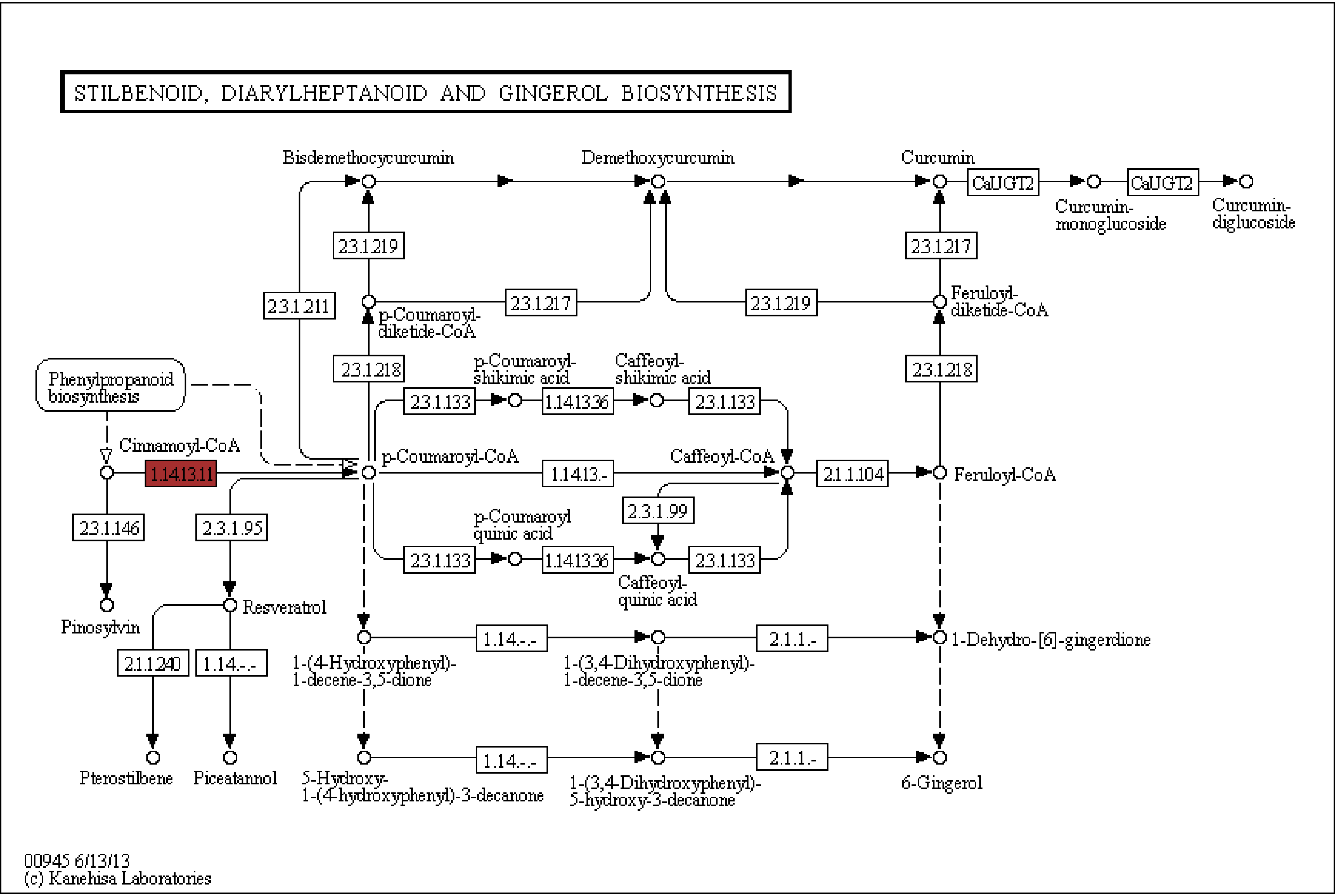
STILBENOID, DIARYLHEPTANOID AND GINGEROL BIOSYNTHESIS
Bisdemethoxycurcumin
Demethoxycurcumin
Curcumin
CaUGT2
CaUGT2
Curcumin-monoglucoside
Curcumin-diglucoside
2.3.1.219
2.3.1.217
2.3.1.211
2.3.1.217
2.3.1.219
p-Coumaroyl-diketide-CoA
Feruloyl-diketide-CoA
2.3.1.218
2.3.1.218
Phenylpropanoid biosynthesis
p-Coumaroyl-shikimic acid
Caffeoyl-shikimic acid
2.3.1.133
1.14.13.36
2.3.1.133
Cinnamoyl-CoA
p-Coumaroyl-CoA
Caffeoyl-CoA
1.14.13.11
1.14.13.-
2.1.1.104
Feruloyl-CoA
2.3.1.146
2.3.1.95
2.3.1.99
p-Coumaroyl quinic acid
2.3.1.133
1.14.13.36
2.3.1.133
Caffeoyl-quinic acid
Pinosylvin
Resveratrol
2.1.1.240
1.14.-.-
1.14.-.-
2.1.1.-
1-Dehydro-[6]-gingerdione
1-(4-Hydroxyphenyl)-1-decene-3,5-dione
1-(3,4-Dihydroxyphenyl)-1-decene-3,5-dione
Pterostilbene
Piceatannol
1.14.-.-
2.1.1.-
5-Hydroxy-1-(4-hydroxyphenyl)-3-decanone
1-(3,4-Dihydroxyphenyl)-5-hydroxy-3-decanone
6-Gingerol
00945 6/13/13
(c) Kanehisa Laboratories

ISOQUINOLINE ALKALOID BIOSYNTHESIS

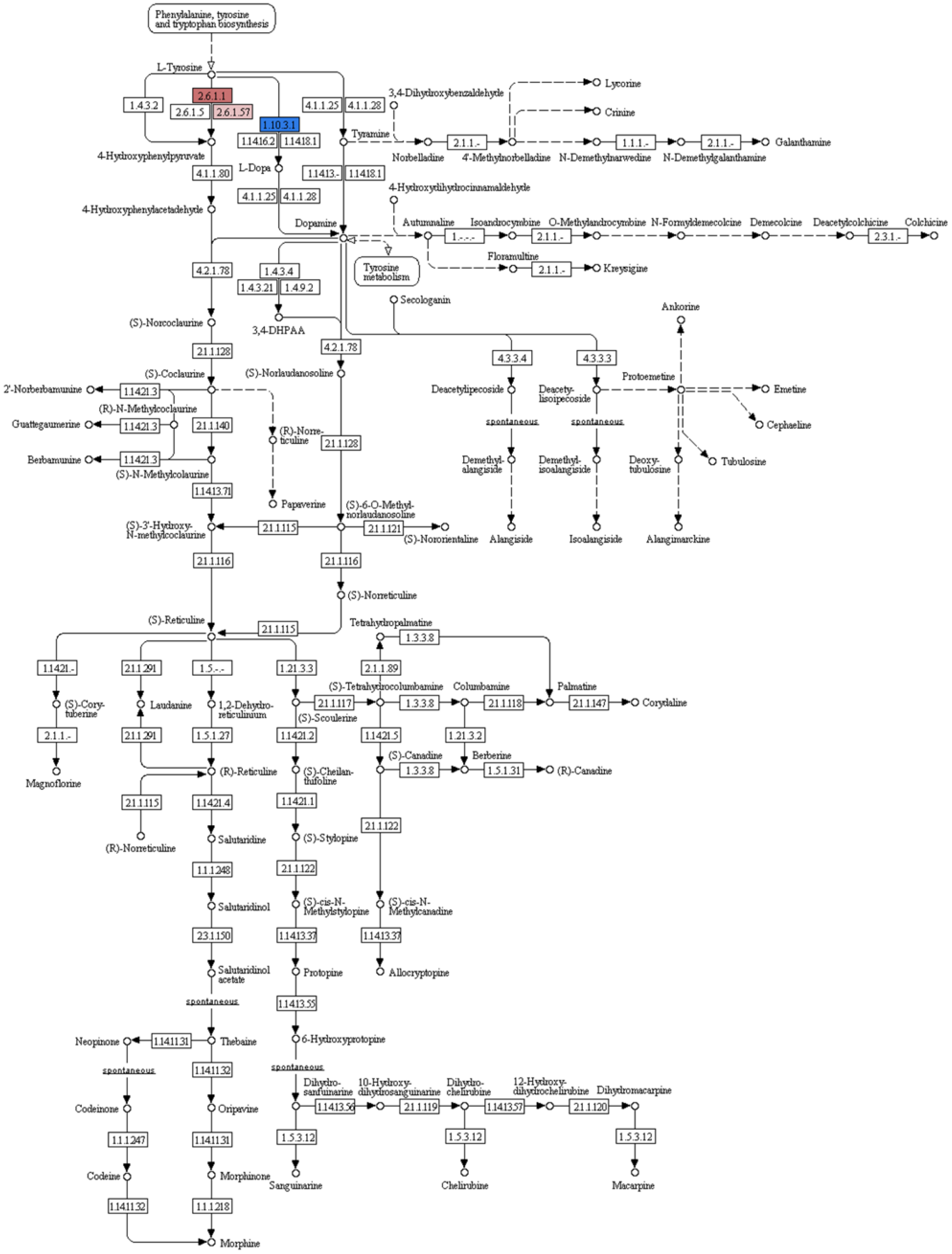

00950 3/14/14
(c) Kanehisa Laboratories

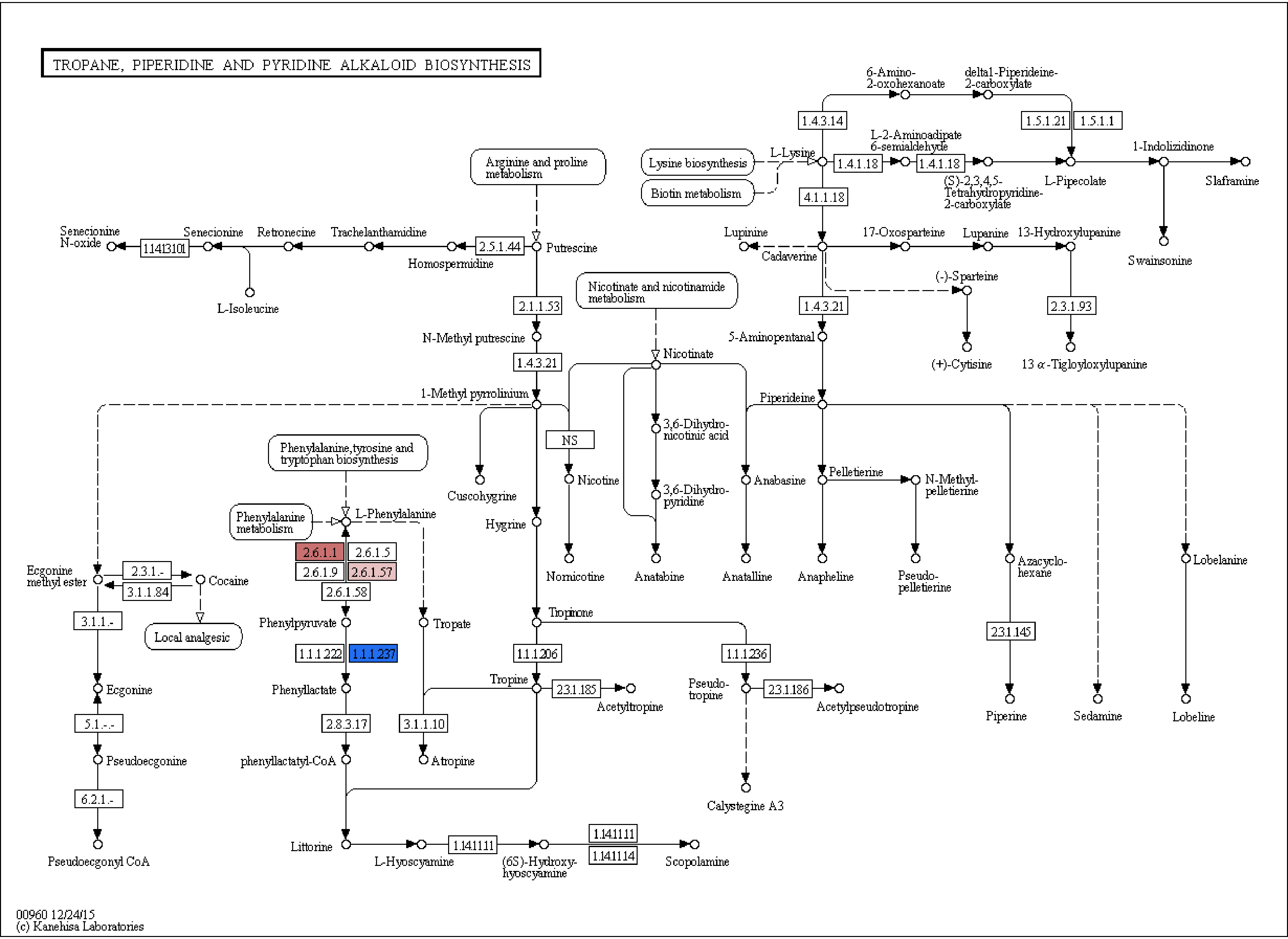
TROPANE, PIPERIDINE AND PYRIDINE ALKALOID BIOSYNTHESIS
Arginine and proline metabolism
Lysine biosynthesis
Biotin metabolism
L-Lysine
6-Amino-2-oxohexanoate
delta1-Piperideine-2-carboxylate
1.4.3.14
1.5.1.21
1.5.1.1
L-2-Aminoadipate 6-semialdehyde
1.4.1.18
1.4.1.18
(S)-2,3,4,5-Tetrahydropyridine-2-carboxylate
L-Pipecolate
1-Indolizidinone
Slaframine
Swainsonine
4.1.1.18
Senecionine N-oxide
1.14.13.101
Senecionine
Retronecine
Trachelanthamidine
2.5.1.44
Putrescine
Homospermidine
L-Isoleucine
Lupinine
Cadaverine
17-Oxosparteine
Lupanine
13-Hydroxylupanine
(-)-Sparteine
2.3.1.93
1.4.3.21
(+)-Cytisine
13 α-Tigloyloxylupanine
Nicotinate and nicotinamide metabolism
2.1.1.53
N-Methyl putrescine
1.4.3.21
5-Aminopentanal
Nicotinate
1-Methyl pyrrolinium
Piperideine
NS
3,6-Dihydro-nicotinic acid
Phenylalanine,tyrosine and tryptophan biosynthesis
Cuscohygrine
Nicotine
3,6-Dihydro-pyridine
Anabasine
Pelletierine
N-Methyl-pelletierine
Phenylalanine metabolism
L-Phenylalanine
Hygrine
2.6.1.1
2.6.1.5
2.6.1.9
2.6.1.57
2.6.1.58
Nornicotine
Anatabine
Anatalline
Anapheline
Pseudo-pelletierine
Azacyclo-hexane
Lobelanine
Ecgonine methyl ester
2.3.1.-
3.1.1.84
Cocaine
Local analgesic
3.1.1.-
Phenylpyruvate
Tropate
Tropinone
1.1.1.222
1.1.1.237
1.1.1.206
1.1.1.236
2.3.1.145
Ecgonine
Phenyllactate
Tropine
2.3.1.185
Acetyltropine
Pseudo-tropine
2.3.1.186
Acetylpseudotropine
Piperine
Sedamine
Lobeline
5.1.-.-
2.8.3.17
3.1.1.10
Pseudoecgonine
phenyllactatyl-CoA
Atropine
6.2.1.-
Calystegine A3
Littorine
1.14.11.11
1.14.11.11
1.14.11.14
Pseudoecgonyl CoA
L-Hyoscyamine
(6S)-Hydroxy-hyoscyamine
Scopolamine
00960 12/24/15
(c) Kanehisa Laboratories

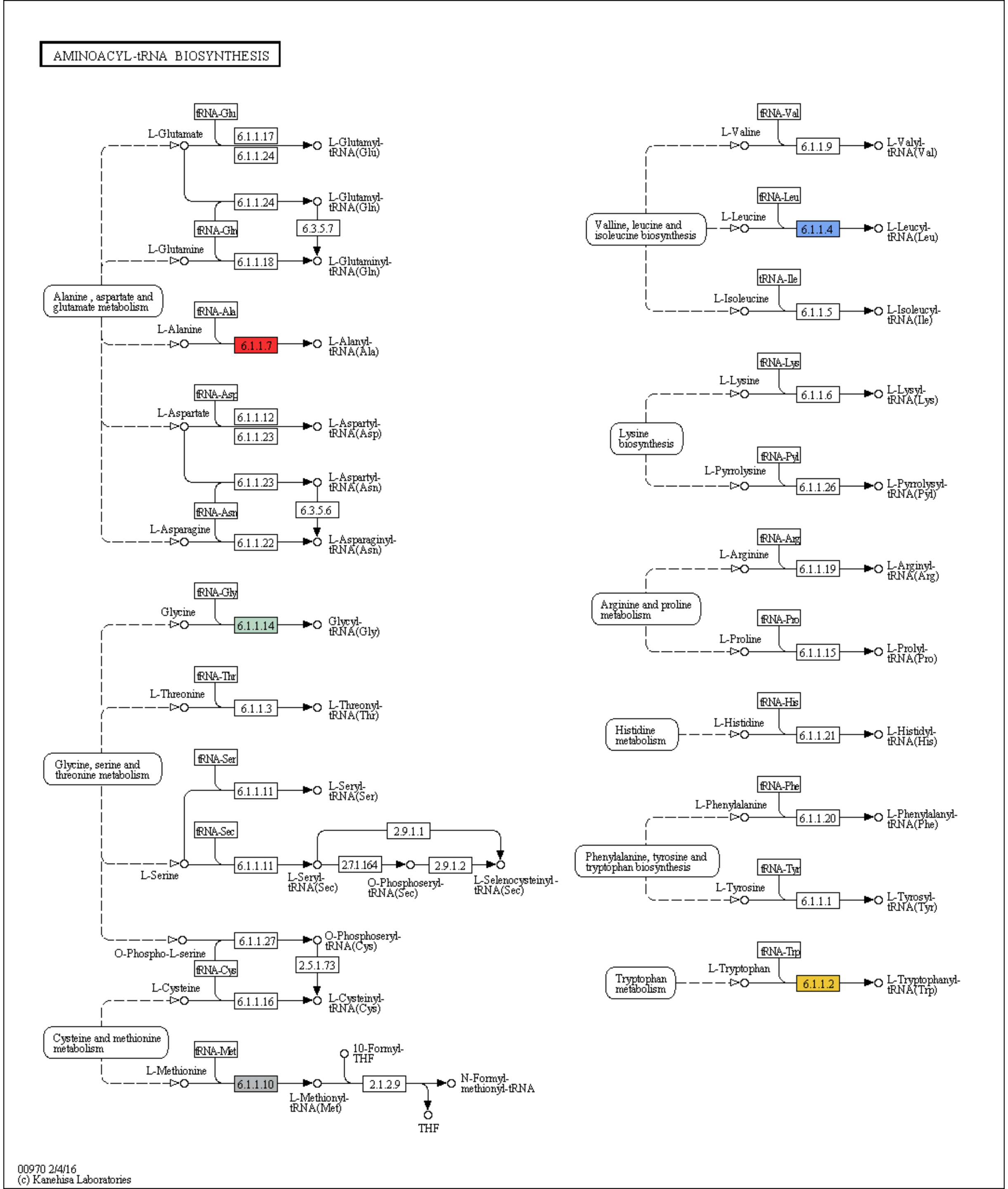
AMINOACYL-tRNA BIOSYNTHESIS
tRNA-Glu
L-Glutamate
6.1.1.17
6.1.1.24
L-Glutamyl-tRNA(Glu)
6.1.1.24
L-Glutamyl-tRNA(Gln)
tRNA-Gln
6.3.5.7
L-Glutamine
6.1.1.18
L-Glutaminyl-tRNA(Gln)
Alanine, aspartate and glutamate metabolism
tRNA-Ala
L-Alanine
6.1.1.7
L-Alanyl-tRNA(Ala)
tRNA-Asp
L-Aspartate
6.1.1.12
6.1.1.23
L-Aspartyl-tRNA(Asp)
6.1.1.23
L-Aspartyl-tRNA(Asn)
tRNA-Asn
6.3.5.6
L-Asparagine
6.1.1.22
L-Asparaginyl-tRNA(Asn)
tRNA-Gly
Glycine
6.1.1.14
Glycyl-tRNA(Gly)
tRNA-Thr
L-Threonine
6.1.1.3
L-Threonyl-tRNA(Thr)
Glycine, serine and threonine metabolism
tRNA-Ser
6.1.1.11
L-Seryl-tRNA(Ser)
tRNA-Sec
2.9.1.1
L-Serine
6.1.1.11
L-Seryl-tRNA(Sec)
2.7.1.164
O-Phosphoseryl-tRNA(Sec)
2.9.1.2
L-Selenocysteinyl-tRNA(Sec)
O-Phospho-L-serine
6.1.1.27
O-Phosphoseryl-tRNA(Cys)
tRNA-Cys
2.5.1.73
L-Cysteine
6.1.1.16
L-Cysteinyl-tRNA(Cys)
Cysteine and methionine metabolism
tRNA-Met
L-Methionine
6.1.1.10
L-Methionyl-tRNA(Met)
10-Formyl-THF
2.1.2.9
N-Formyl-methionyl-tRNA
THF
tRNA-Val
L-Valine
6.1.1.9
L-Valyl-tRNA(Val)
Valine, leucine and isoleucine biosynthesis
tRNA-Leu
L-Leucine
6.1.1.4
L-Leucyl-tRNA(Leu)
tRNA-Ile
L-Isoleucine
6.1.1.5
L-Isoleucyl-tRNA(Ile)
tRNA-Lys
L-Lysine
6.1.1.6
L-Lysyl-tRNA(Lys)
Lysine biosynthesis
tRNA-Pyl
L-Pyrrolysine
6.1.1.26
L-Pyrrolysyl-tRNA(Pyl)
tRNA-Arg
L-Arginine
6.1.1.19
L-Arginyl-tRNA(Arg)
Arginine and proline metabolism
tRNA-Pro
L-Proline
6.1.1.15
L-Prolyl-tRNA(Pro)
tRNA-His
Histidine metabolism
L-Histidine
6.1.1.21
L-Histidyl-tRNA(His)
tRNA-Phe
L-Phenylalanine
6.1.1.20
L-Phenylalanyl-tRNA(Phe)
Phenylalanine, tyrosine and tryptophan biosynthesis
tRNA-Tyr
L-Tyrosine
6.1.1.1
L-Tyrosyl-tRNA(Tyr)
tRNA-Trp
Tryptophan metabolism
L-Tryptophan
6.1.1.2
L-Tryptophanyl-tRNA(Trp)
00970 2/4/16
(c) Kanehisa Laboratories

METABOLISM OF XENOBIOTICS BY CYTOCHROME P450

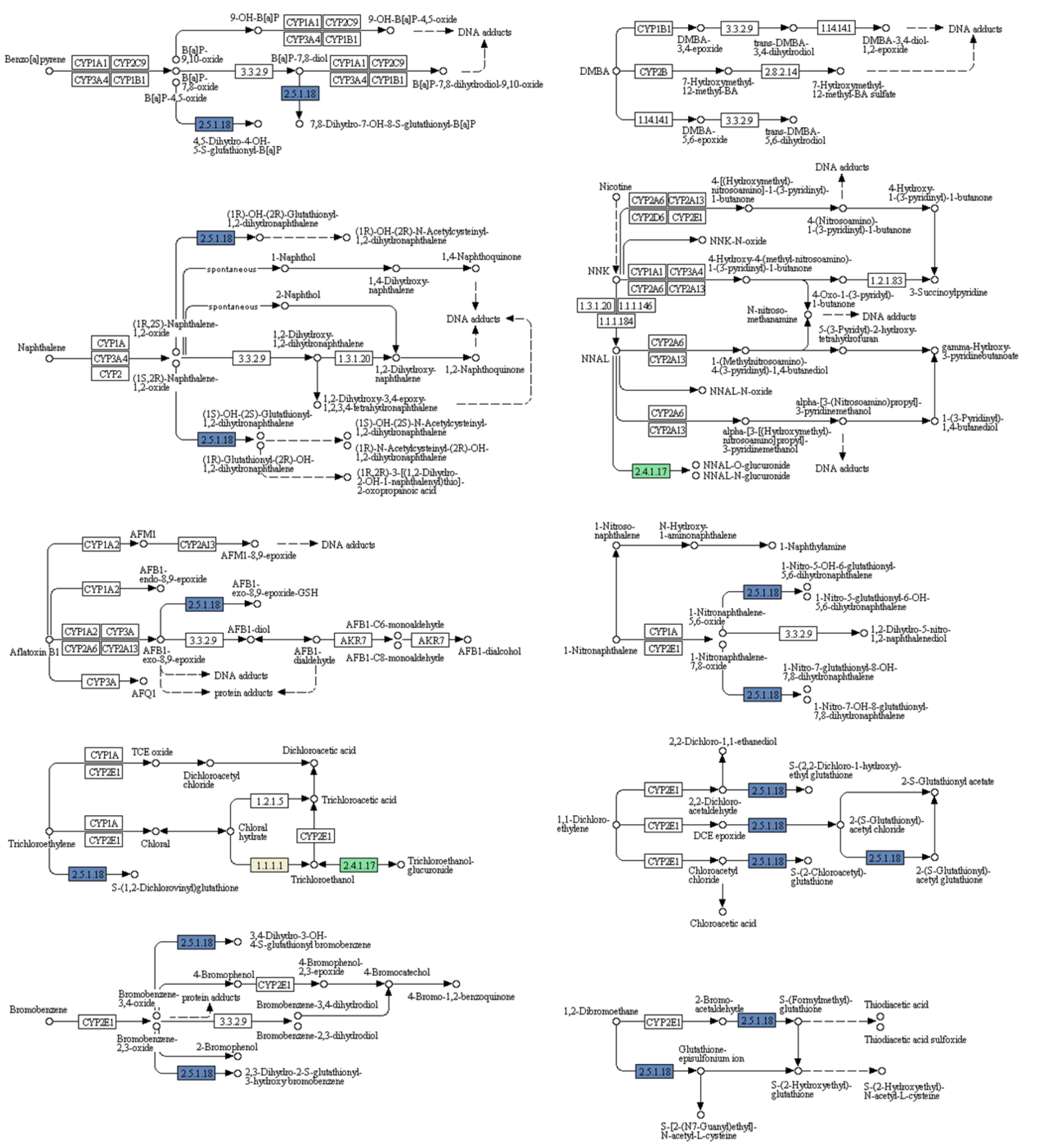

00980 1/8/16

(c) Kanehisa Laboratories

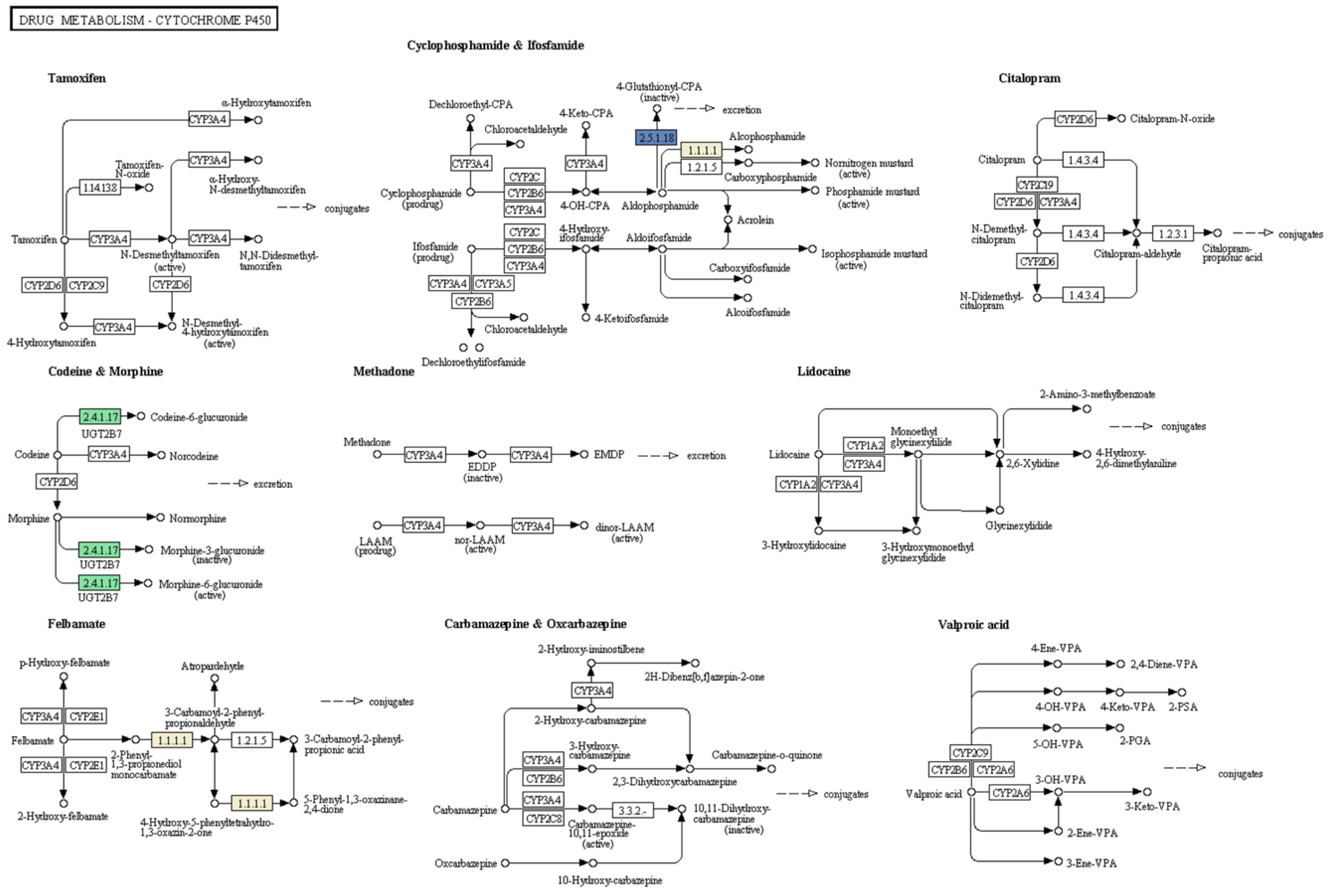

00982 1/8/16
(c) Kanehisa Laboratories

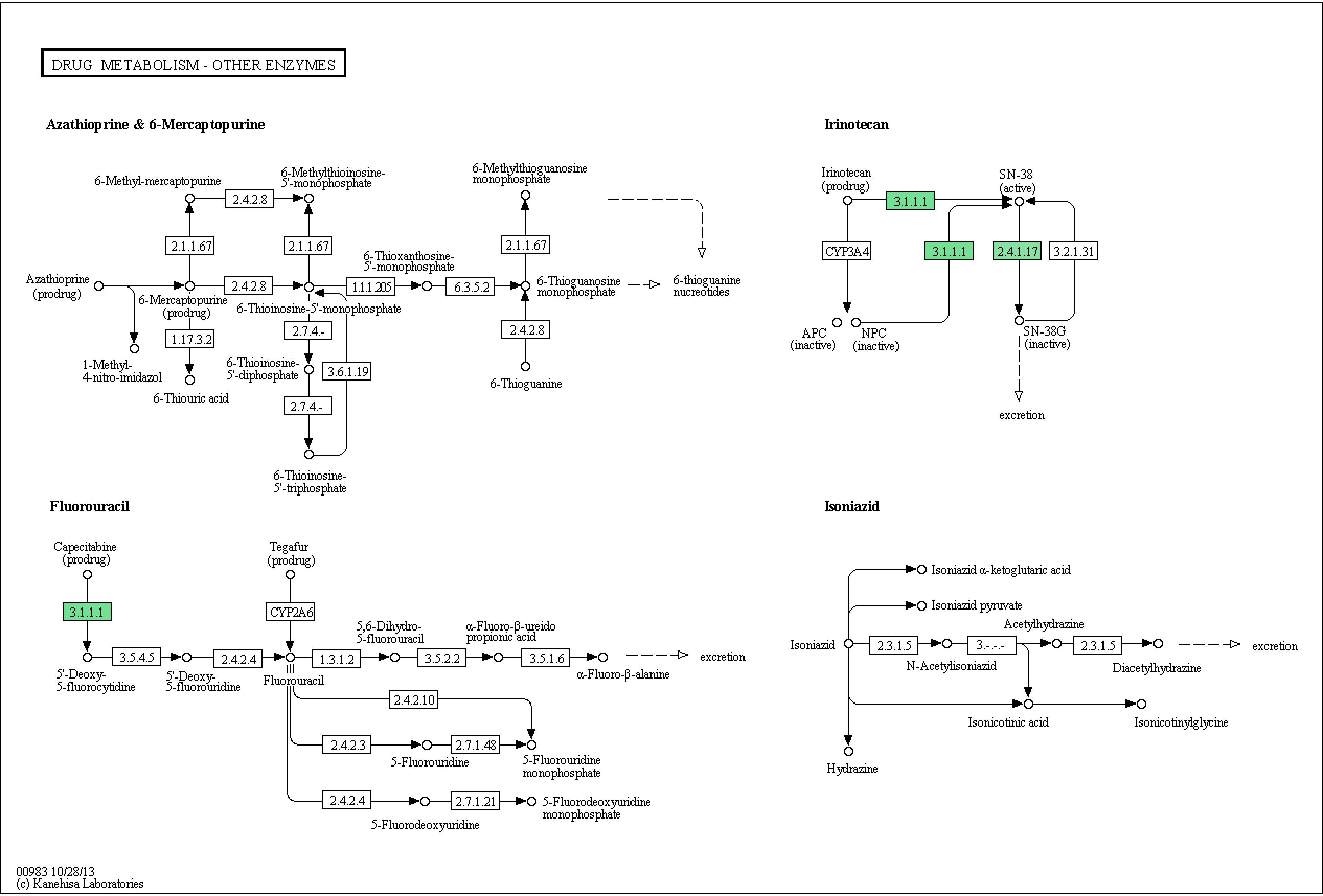
DRUG METABOLISM - OTHER ENZYMES
Azathioprine & 6-Mercaptopurine
6-Methyl-mercaptopurine
2.4.2.8
6-Methylthioinosine-5'-monophosphate
6-Methylthioguanosine monophosphate
2.1.1.67
2.1.1.67
6-Thioxanthosine-5'-monophosphate
2.1.1.67
Azathioprine (prodrug)
2.4.2.8
1.1.1.205
6.3.5.2
6-Thioguanosine monophosphate
6-thioguanine nucreotides
6-Mercaptopurine (prodrug)
6-Thioinosine-5'-monophosphate
2.7.4.-
1.17.3.2
2.4.2.8
1-Methyl-4-nitro-imidazol
6-Thiouric acid
6-Thioinosine-5'-diphosphate
3.6.1.19
6-Thioguanine
2.7.4.-
6-Thioinosine-5'-triphosphate
Irinotecan
Irinotecan (prodrug)
3.1.1.1
SN-38 (active)
CYP3A4
3.1.1.1
2.4.1.17
3.2.1.31
APC (inactive)
NPC (inactive)
SN-38G (inactive)
excretion
Fluorouracil
Capecitabine (prodrug)
Tegafur (prodrug)
3.1.1.1
CYP2A6
5,6-Dihydro-5-fluorouracil
α-Fluoro-β-ureido propionic acid
3.5.4.5
2.4.2.4
1.3.1.2
3.5.2.2
3.5.1.6
excretion
5'-Deoxy-5-fluorocytidine
5'-Deoxy-5-fluorouridine
Fluorouracil
α-Fluoro-β-alanine
2.4.2.10
2.4.2.3
2.7.1.48
5-Fluorouridine
5-Fluorouridine monophosphate
2.4.2.4
2.7.1.21
5-Fluorodeoxyuridine monophosphate
5-Fluorodeoxyuridine
Isoniazid
Isoniazid α-ketoglutaric acid
Isoniazid pyruvate
Acetylhydrazine
Isoniazid
2.3.1.5
3.-.-.-
2.3.1.5
excretion
N-Acetylisoniazid
Diacetylhydrazine
Isonicotinic acid
Isonicotinylglycine
Hydrazine
00983 10/28/13
(c) Kanehisa Laboratories

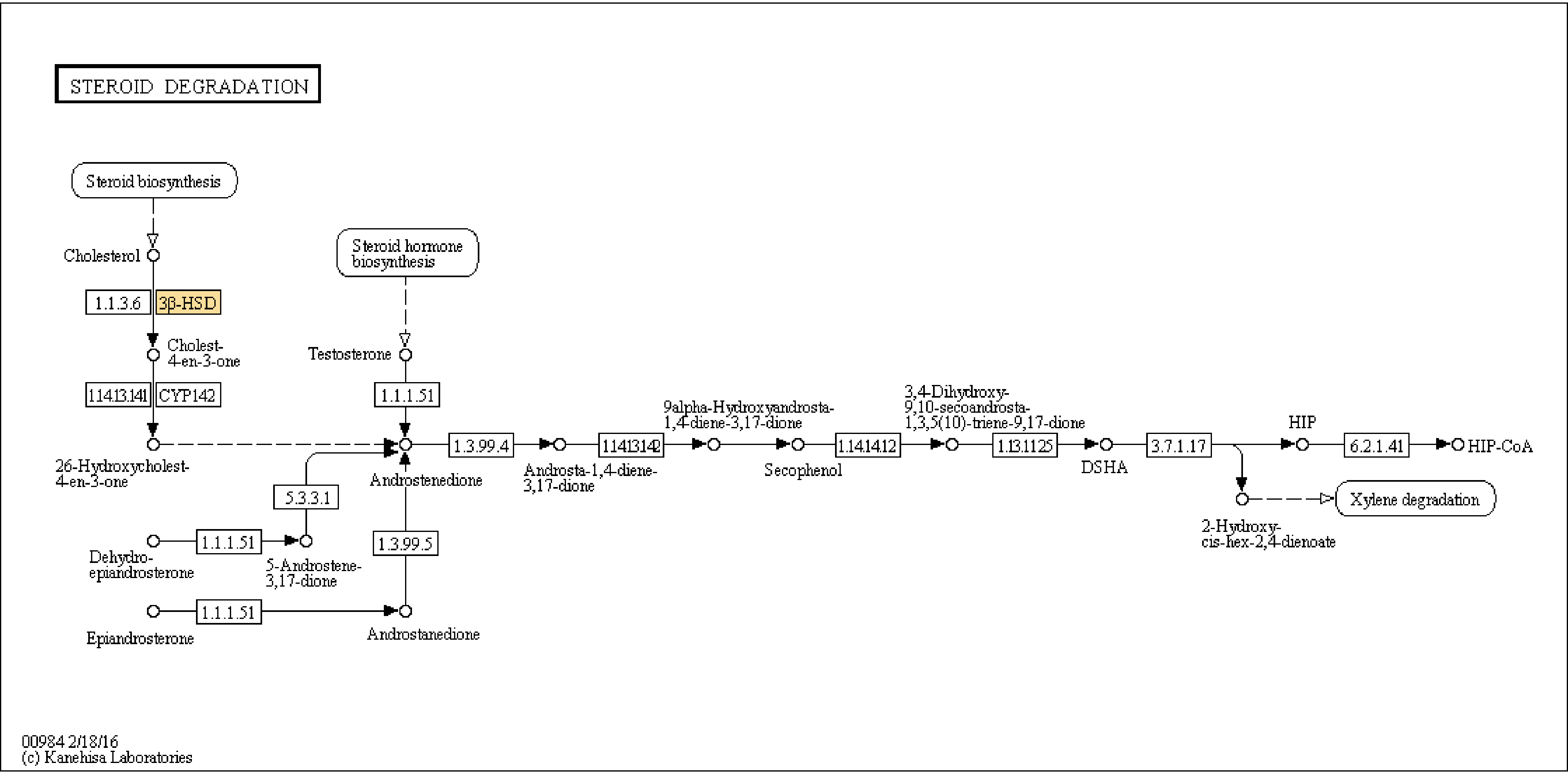
STEROID DEGRADATION
Steroid biosynthesis
Cholesterol
1.1.3.6
3β-HSD
Cholest-4-en-3-one
1.14.13.141
CYP142
26-Hydroxycholest-4-en-3-one
Steroid hormone biosynthesis
Testosterone
1.1.1.51
Androstenedione
5.3.3.1
Dehydro-epiandrosterone
1.1.1.51
5-Androstene-3,17-dione
1.3.99.5
Epiandrosterone
1.1.1.51
Androstanedione
1.3.99.4
Androsta-1,4-diene-3,17-dione
1.14.13.142
9alpha-Hydroxyandrosta-1,4-diene-3,17-dione
Secophenol
1.14.14.12
3,4-Dihydroxy-9,10-secoandrosta-1,3,5(10)-triene-9,17-dione
1.13.11.25
DSHA
3.7.1.17
HIP
6.2.1.41
HIP-CoA
2-Hydroxy-cis-hex-2,4-dienoate
Xylene degradation
00984 2/18/16
(c) Kanehisa Laboratories

BIOSYNTHESIS OF UNSATURATED FATTY ACIDS

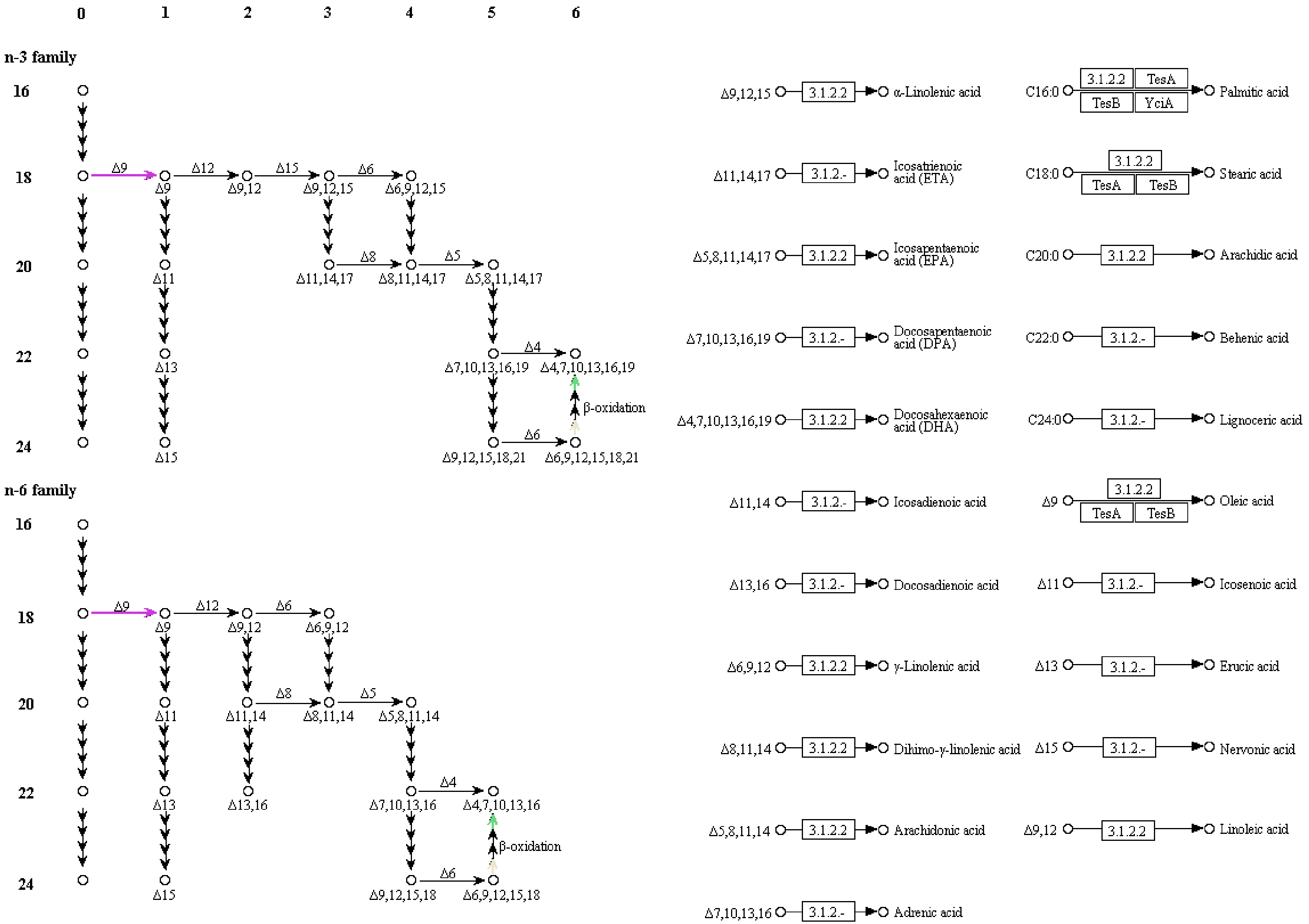

01040 5/19/15

(c) Kanehisa Laboratories

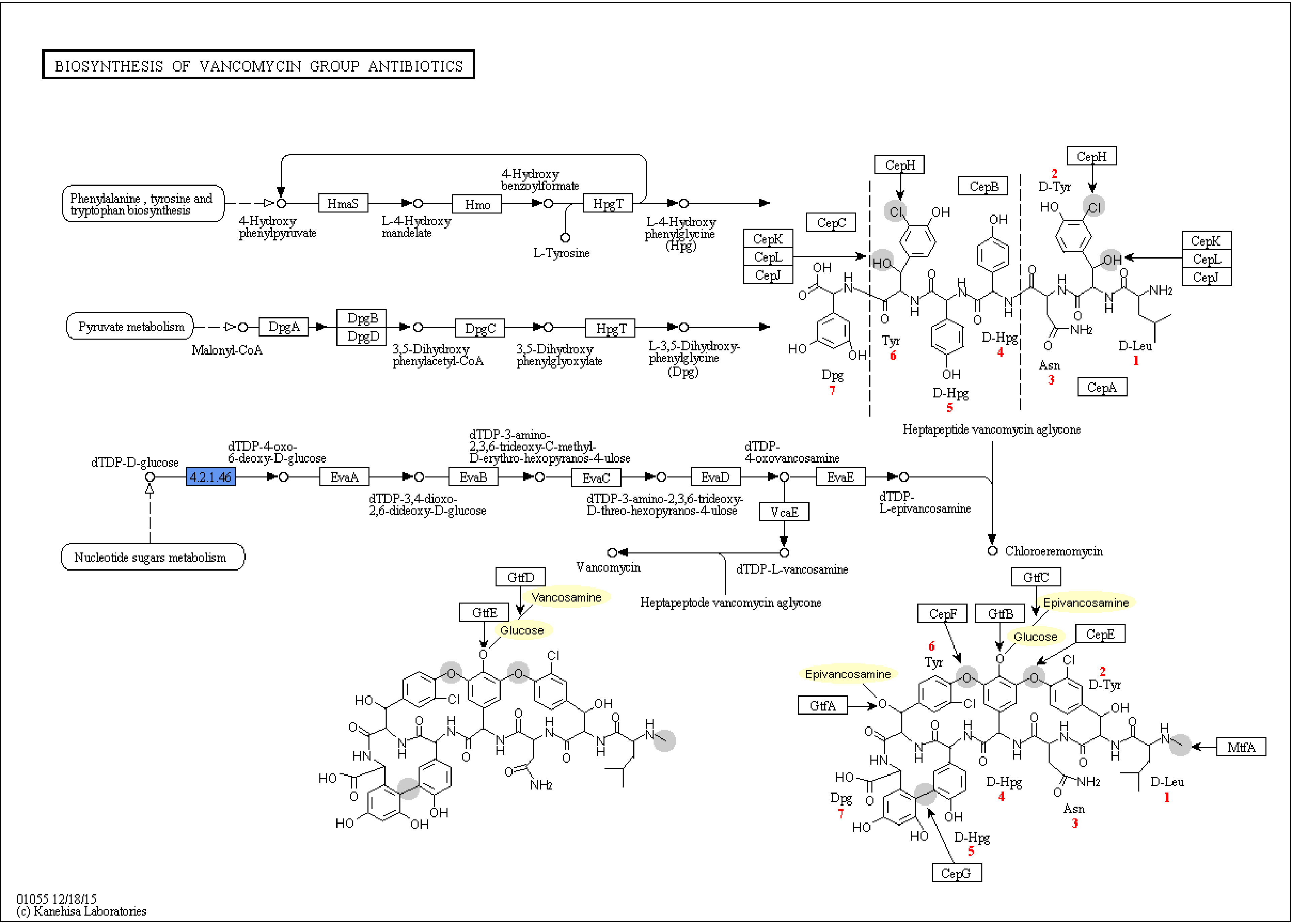

BIOSYNTHESIS OF VANCOMYCIN GROUP ANTIBIOTICS
Phenylalanine, tyrosine and tryptophan biosynthesis
4-Hydroxy phenylpyruvate
HmaS
L-4-Hydroxy mandelate
Hmo
4-Hydroxy benzoylformate
HpgT
L-Tyrosine
L-4-Hydroxy phenylglycine (Hpg)
Pyruvate metabolism
Malonyl-CoA
DpgA
DpgB
DpgD
3,5-Dihydroxy phenylacetyl-CoA
DpgC
3,5-Dihydroxy phenylglyoxylate
HpgT
L-3,5-Dihydroxy-phenylglycine (Dpg)
CepH
CepB
CepC
CepK
CepL
CepJ
CepA
Dpg 7
Tyr 6
D-Hpg 5
D-Hpg 4
Asn 3
D-Tyr 2
D-Leu 1
Heptapeptide vancomycin aglycone
dTDP-D-glucose
4.2.1.46
dTDP-4-oxo-6-deoxy-D-glucose
EvaA
dTDP-3,4-dioxo-2,6-dideoxy-D-glucose
EvaB
dTDP-3-amino-2,3,6-trideoxy-C-methyl-D-erythro-hexopyranos-4-ulose
EvaC
dTDP-3-amino-2,3,6-trideoxy-D-threo-hexopyranos-4-ulose
EvaD
dTDP-4-oxovancosamine
EvaE
dTDP-L-epivancosamine
VcaE
dTDP-L-vancosamine
Nucleotide sugars metabolism
Vancomycin
Heptapeptode vancomycin aglycone
Chloroeremomycin
GtfD
GtfE
Vancosamine
Glucose
GtfC
CepF
GtfB
CepE
Epivancosamine
Glucose
Epivancosamine
GtfA
MtfA
CepG
01055 12/18/15
(c) Kanehisa Laboratories

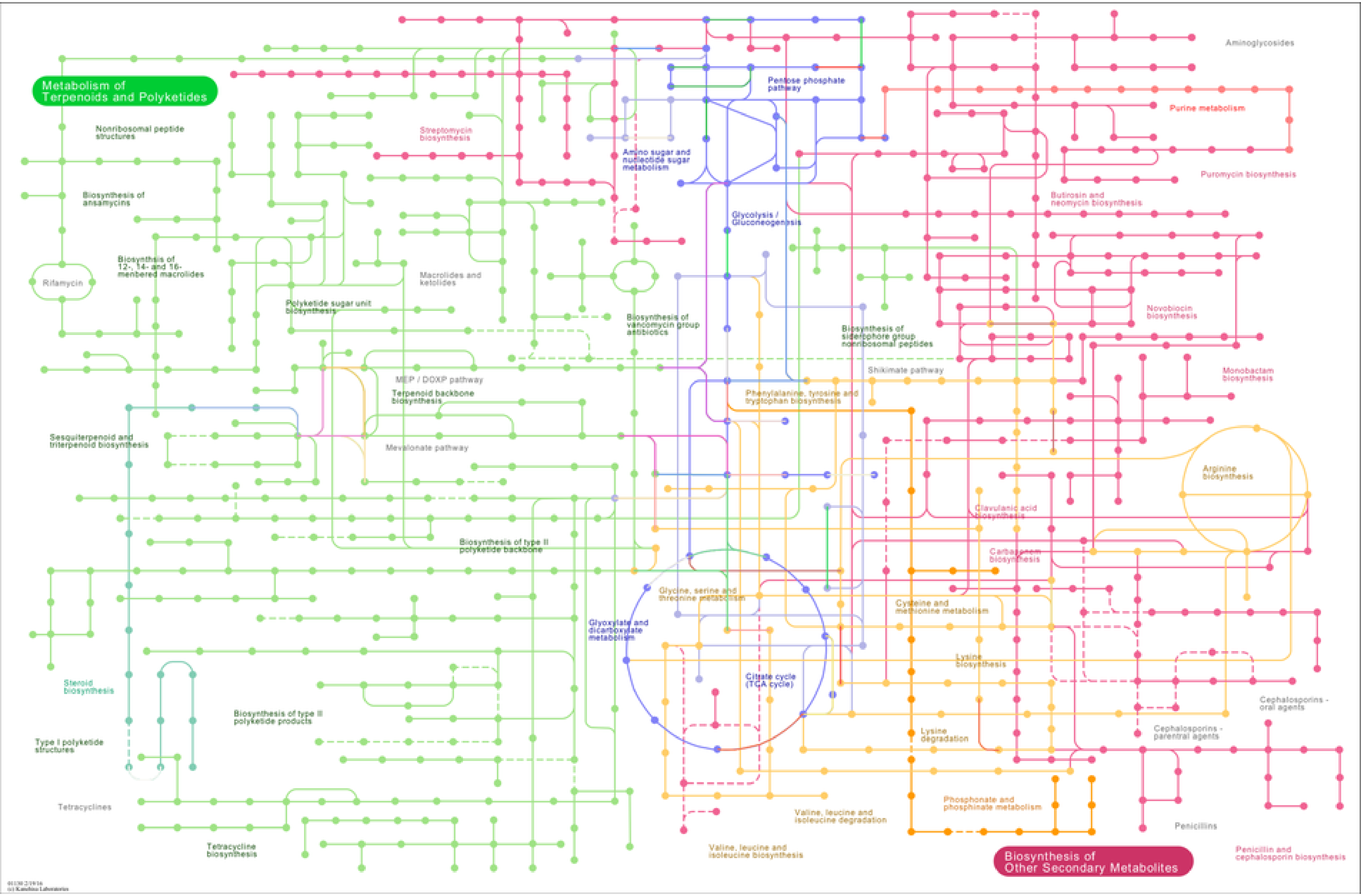
Metabolism of Terpenoids and Polyketides
Nonribosomal peptide structures
Biosynthesis of ansamycins
Rifamycin
Biosynthesis of 12-, 14- and 16-membered macrolides
Streptomycin biosynthesis
Amino sugar and nucleotide sugar metabolism
Pentose phosphate pathway
Aminoglycosides
Purine metabolism
Puromycin biosynthesis
Butirosin and neomycin biosynthesis
Glycolysis / Gluconeogenesis
Macrolides and ketolides
Polyketide sugar unit biosynthesis
Biosynthesis of vancomycin group antibiotics
Biosynthesis of siderophore group nonribosomal peptides
Novobiocin biosynthesis
Monobactam biosynthesis
Shikimate pathway
MEP / DOXP pathway
Terpenoid backbone biosynthesis
Phenylalanine, tyrosine and tryptophan biosynthesis
Sesquiterpenoid and triterpenoid biosynthesis
Mevalonate pathway
Arginine biosynthesis
Clavulanic acid biosynthesis
Biosynthesis of type II polyketide backbone
Carbapenem biosynthesis
Glycine, serine and threonine metabolism
Cysteine and methionine metabolism
Glyoxylate and dicarboxylate metabolism
Lysine biosynthesis
Steroid biosynthesis
Citrate cycle (TCA cycle)
Cephalosporins - oral agents
Biosynthesis of type II polyketide products
Cephalosporins - parental agents
Type I polyketide structures
Lysine degradation
Phosphonate and phosphinate metabolism
Tetracyclines
Valine, leucine and isoleucine degradation
Penicillins
Tetracycline biosynthesis
Valine, leucine and isoleucine biosynthesis
Penicillin and cephalosporin biosynthesis
Biosynthesis of Other Secondary Metabolites

PHOSPHATIDYLINOSITOL SIGNALING SYSTEM

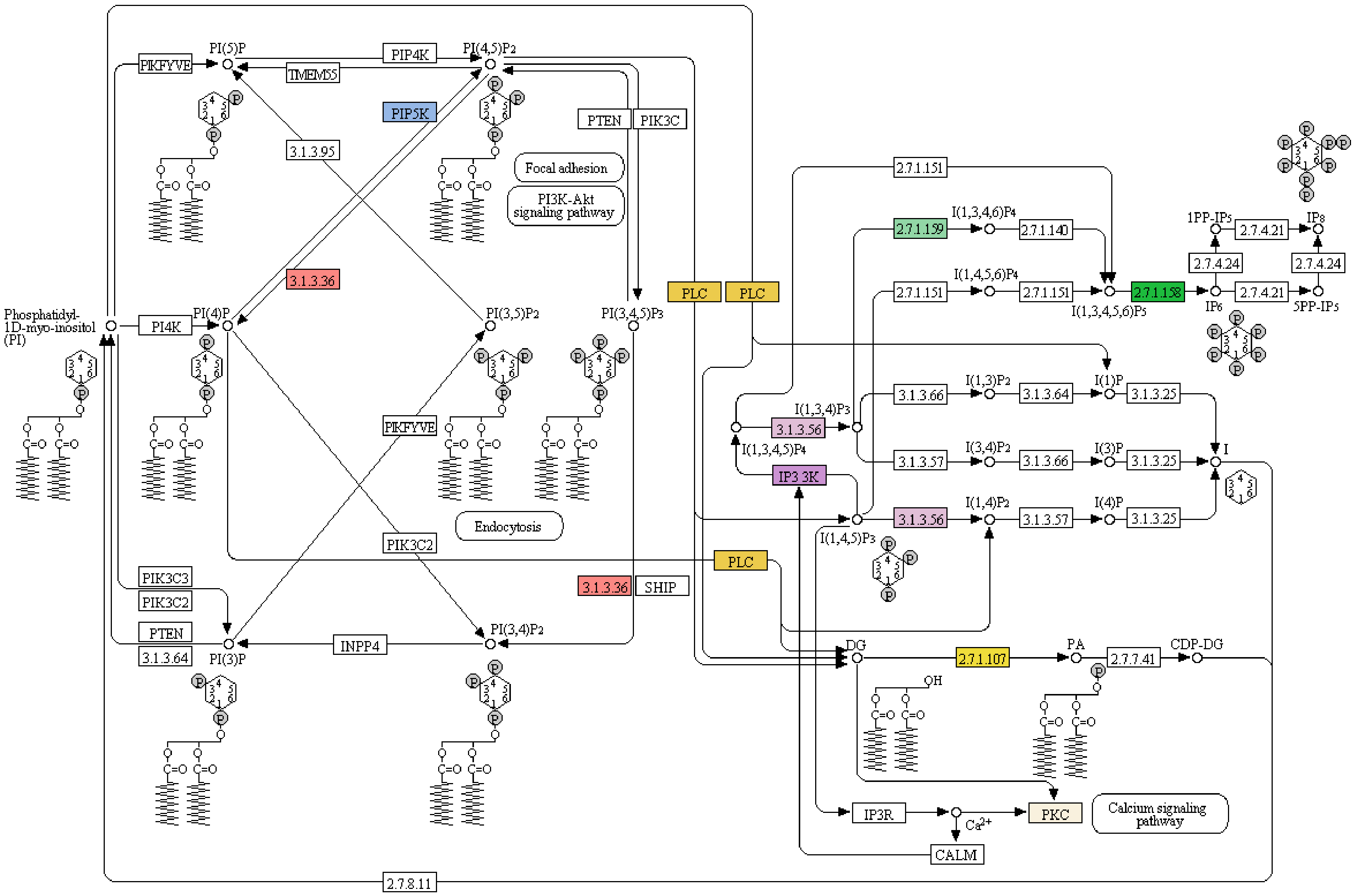

04070 10/23/15

(c) Kanehisa Laboratories

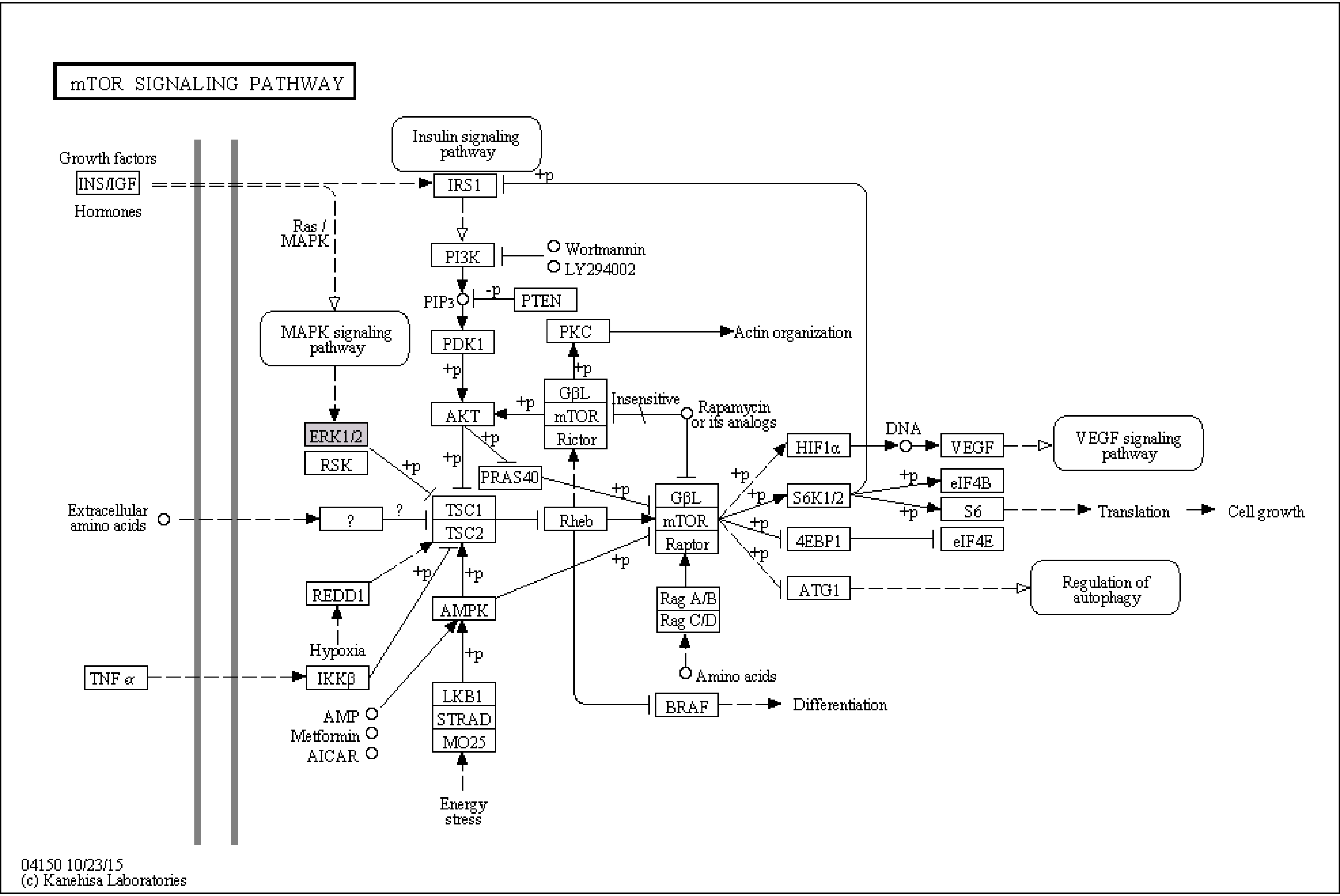
mTOR SIGNALING PATHWAY
Insulin signaling pathway
Growth factors
INS/IGF
Hormones
IRS1
+p
Ras / MAPK
PI3K
Wortmannin
LY294002
PIP3
-p
PTEN
MAPK signaling pathway
PKC
Actin organization
PDK1
+p
GβL
mTOR
Rictor
+p
AKT
Insensitive
Rapamycin or its analogs
ERK1/2
+p
RSK
+p
PRAS40
DNA
HIF1α
VEGF
VEGF signaling pathway
eIF4B
S6K1/2
Extracellular amino acids
?
?
TSC1
TSC2
Rheb
GβL
mTOR
Raptor
S6
Translation
Cell growth
4EBP1
eIF4E
ATG1
Regulation of autophagy
REDD1
AMPK
Rag A/B
Rag C/D
Hypoxia
TNF α
IKKβ
Amino acids
LKB1
STRAD
MO25
BRAF
Differentiation
AMP
Metformin
AICAR
Energy stress
04150 10/23/15
(c) Kanehisa Laboratories

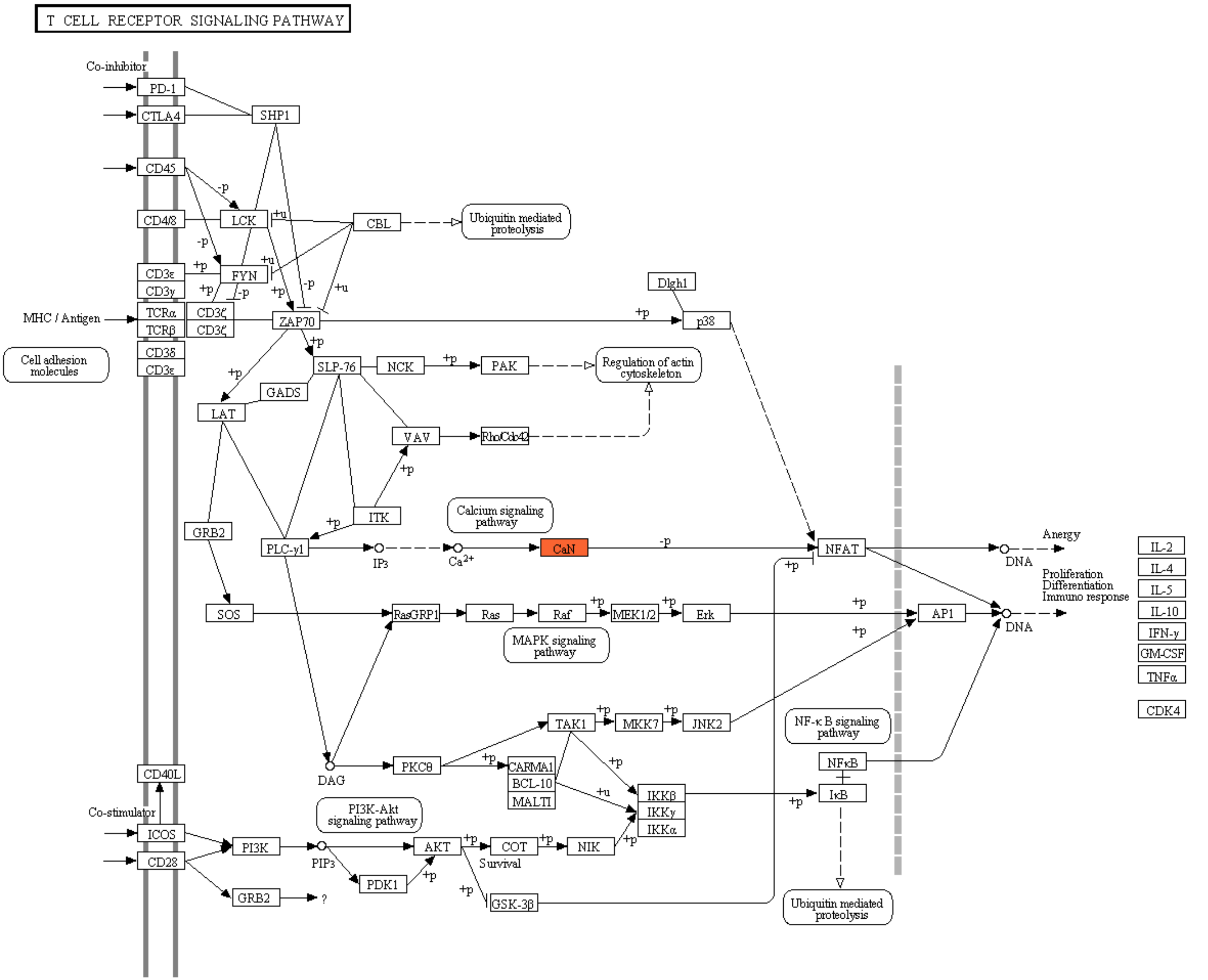

04660 2/21/14
(c) Kanehisa Laboratories
